# Possible introgression of the *VRTN* mutation increasing vertebral number, carcass length and teat number from Chinese pigs into European pigs

**DOI:** 10.1038/srep19240

**Published:** 2016-01-19

**Authors:** Jie Yang, Lusheng Huang, Ming Yang, Yin Fan, Lin Li, Shaoming Fang, Wenjiang Deng, Leilei Cui, Zhen Zhang, Huashui Ai, Zhenfang Wu, Jun Gao, Jun Ren

**Affiliations:** 1 College of Animal Science and National Engineering Research Center for Breeding Swine Industry, South China Agricultural University, Guangdong, P.R. China; 2 State Key Laboratory for Pig Genetic Improvement and Production Technology, Jiangxi Agricultural University, Nanchang, P.R. China; 3 National Engineering Research Center for Breeding Swine Industry, Guangdong Wens Foodstuffs Group Co., Ltd, Guangdong, P.R. China

## Abstract

*Vertnin* (*VRTN*) variants have been associated with the number of thoracic vertebrae in European pigs, but the association has not been evidenced in Chinese indigenous pigs. In this study, we first performed a genome-wide association study in Chinese Erhualian pigs using one *VRTN* candidate causative mutation and the Illumina Porcine 60K SNP Beadchips. The *VRTN* mutation is significantly associated with thoracic vertebral number in this population. We further show that the *VRTN* mutation has pleiotropic and desirable effects on teat number and carcass (body) length across four diverse populations, including Erhualian, White Duroc × Erhualian F_2_ population, Duroc and Landrace pigs. No association was observed between *VRTN* genotype and growth and fatness traits in these populations. Therefore, testing for the *VRTN* mutation in pig breeding schemes would not only increase the number of vertebrae and nipples, but also enlarge body size without undesirable effects on growth and fatness traits, consequently improving pork production. Further, by using whole-genome sequence data, we show that the *VRTN* mutation was possibly introgressed from Chinese pigs into European pigs. Our results provide another example showing that introgressed Chinese genes greatly contributed to the development and production of modern European pig breeds.

The number of vertebrae including cervical, thoracic, lumbar, sacral and caudal vertebrae shows developmental constraint in most vertebrates^1^. For instance, the numbers of cervical and sacral vertebrae are respectively fixed at 7 and 4 in mammals. However, the number of thoracic and lumbar vertebrae varies considerably in pigs^2^. European commercial breeds like Large White, Landrace and Duroc have more (n = 21–23) thoracic and lumbar vertebrae than Chinese indigenous breeds (n = 19-20)^3^,^4^.

The total number of thoracic and lumbar vertebrae is an economically important trait that affects meat production in pigs^5^. Thus, deciphering the molecular basis of thoracic and lumbar vertebral number in pigs would not only provide insight into our understanding of vertebral development in mammals, but also benefit the pig industry by selecting for more vertebrae using molecular breeding technology. To date, two major quantitative trait locus (QTL) for vertebral number have been consistently identified in multiple pig populations: one for the number of lumbar vertebrae on *Sus scrofa* chromosome 1 (SSC1)^6^,^7^, and the other for the number of thoracic vertebrae on SSC7^7^,^8^,^9^,^10^. The Pro192Leu mutation in the *NR6A1* gene is known to be the causal variant (QTN) underlying the SSC1 QTL effect. The favorable allele at the QTN that increases vertebral number is fixed in the European commercial breeds^11^. Interestingly, a recent study of whole-genome sequencing shows that the *NR6A1* locus is one of the strongest selective sweep regions in European domestic pigs^12^.

*Vertnin* (*VRTN*) has been proposed to be the gene responsible for the SSC7 QTL affecting thoracic vertebral number^10^. Unlike the alleles at the SSC1 QTL, the SSC7 QTL alleles are segregating in the European commercial breeds, and are thus of significant interest for the pig industry by selecting the favorable allele to increase thoracic vertebral number and pork production. In our previous study, we provided additional evidence that *VRTN* is the underlying gene for the SSC7 QTL. By a battery of genetic analyses, we identified two *VRTN* variants as strong candidate QTN for this QTL effect. One of the two variants is a SNP in the promoter region, and the other is an Indel in intron 1 of the *VRTN* gene. Both variants reside in conserved functional elements and possibly affect the expression of *VRTN*^13^. We further showed that the favorable allele for increased thoracic vertebrae at the QTN is also segregating in some of Chinese indigenous breeds and is possibly of Chinese origin^13^. Recently, Kensuke *et al.* (2013) reported that the *VRTN* mutation is significantly associated with body length and intramuscular fat content (IMF) in a Duroc population^14^. However, two subsequent reports indicated that there was no significant association between this mutation and IMF in Duroc pigs^15^,^16^. Therefore, the associations of *VRTN* mutations with production traits related to vertebral number need further investigations.

In this study, we demonstrate that the *VRTN* candidate QTNs are significantly associated with the number of thoracic vertebrae in Chinese Erhualian pigs. We further show that the *VRTN* candidate QTNs are associated with thoracic vertebrae, carcass/body length and teat number in divergent populations. Further, we resequenced the *VRTN* region using representative individuals from Chinese and European pig breeds. We illustrate that the *VRTN* mutation was possibly introgressed from Chinese pigs into European pigs. These findings advance our understanding of the molecular basis of vertebral number, and have immediate transition into breeding practices to improve meat production in both European commercial pigs and Chinese indigenous pigs. Moreover, our results provide another example showing that introgressed Chinese (Asian) genes greatly contributed to the development and production of modern European pig breeds^17^,^18^,^19^.

## Methods

### Ethics statement

All procedures involving animals followed the guidelines for the care and use of experimental animals that were approved by the State Council of the People’s Republic of China. The ethics committee of Jiangxi Agricultural University specifically approved this study.

### Animals and phenotype recording

In this study, experimental animals were from five populations, including one White Duroc × Erhualian F_2_ intercross (referred hereafter as the F_2_ cross), one Chinese purebred (Erhualian) population, and three European purebred populations (Duroc, Landrace and Large White).

The F_2_ cross was developed and managed as described previously^20^. Briefly, two White Duroc boars were mated to 17 Erhualian sows. Nine F_1_ boars and 59 F_1_ sows were then intercrossed to produce a total of 1,912 F_2_ animals in 6 batches. Of the 1,912 F_2_ animals, 1,034 individuals were slaughtered for phenotype recording at the age of 240 ± 3 days. A total of 928 F_2_ individuals with phenotypic data of thoracic vertebral number, carcass length, teat number, body weight, average daily gain, intramuscular fat content and backfat thickness were used in this study.

Erhualian, a Chinese indigenous pig breed, was originally distributed in Jiangsu Province. This breed is characterized by its prolificacy (litter size >16), excellent maternity and favorable meat quality^21^. We purchased 332 Erhualian pigs from Jiangsu Province. The 322 pigs included 166 barrows and 166 gilts from 9 sire families. All Erhualian pigs were fed with consistent diet under a standardized feeding and management regimen, and given free access to water, and then slaughtered at 300 ± 3 days of age in the same commercial abattoir. Phenotypes including thoracic vertebral number, lumber vertebral number, carcass length, teat number, body weight at slaughter, intramuscular fat content and average backfat thickness at the shoulder, first rib and hip were measured in the Erhualian population as described recently^22^,^23^.

A total of 3,495 European purebred pig samples were collected from 3 pig breeding farms, including 1,050 Duroc boars, 1,097 Landrace sows, and 1,348 Large White sows. Of the 3,495 animals, 1,348 Large White sows were not recorded for any phenotypic traits, while 833 Duroc pigs and 596 Landrace pigs were recorded for teat number, average daily gain, body length, backfat thickness and intramuscular fat content at the weight of 100 ± 5 kg. Backfat thickness and intramuscular fat content were measured between the 10th and 11th rib using a Preg-Alert Pro B-ultrasound machine (Renco Corporation, USA). The average daily gains were calculated as linear regressions of body weight from 30 ± 5 kg to 100 ± 5 kg.

### SNP genotyping

Genomic DNA was extracted from ear tissue of each pig using a standard phenol/chloroform method. DNA quality was determined by a Nanodrop-100 spectrophotometer (Thermo Fisher, USA). The Erhualian (n = 332) and Duroc (n = 833) pigs were genotyped for 62,163 SNPs on the Porcine SNP 60K Beadchips (Illumina, USA) according to the supplier’s protocol. The quality control criteria were applied for the SNP data by the check.marker function of GenABEL^24^. Animals with SNP call rates ≥95% and familial Mendelian error rates ≤0.1, and SNPs with call rates ≥95%, minor allele frequencies (MAF) ≥0.1 and significance levels of deviation from Hardy-Weinberg equilibrium ≥10^−6^ were included for further statistical analysis. The 60K SNP data of the F_2_ cross (n = 1,015) that passed quality control are available from our previous studies^23^,^25^.

A total of 4,832 pigs including 1,015 individuals from the F_2_ cross (19 F_0_, 68 F_1_ and 928 F_2_ pigs), 322 Erhualian pigs, 1,050 Duroc boars, 1,097 Landrace sows and 1,348 Large White sows were genotyped for the *VRTN* g.20311_20312ins291 mutation (referred hereafter as the *VRTN* mutation), a strong candidate QTN underlying the SSC7 QTL effect on thoracic vertebral number^13^. The genotypes of this mutation were judged using a PCR-based test. Primer pairs (*VRTN*-FP: GGC AGG GAA GGT GTT TGT TA and *VRTN*-RP: GAC TGG CCT CTG TCC CTT G) were designed using Primer Premier 5.0 based on the *VRTN* sequence (GenBank accession no. AB554652.1). The PCR reaction was performed using a reaction mix of 25 μL containing 40 ng of genomic DNA, 2.5 μL Buffer, 1.5 μL MgCl_2_, forward and reverse primers (2 pM each), and 2.5 U Taq. PCR products were separated by 2% agarose gel electrophoresis and the genotypes were visually recorded according to the length of amplicon. The mutant allele (*ins*) was represented by amplicons of 411 bp and the wild-type allele (*del*) by amplicons of 120 bp.

### Association analysis

Prior to the association analysis, we checked the distribution of all phenotypes with the Shapiro test^26^. All phenotypic data conformed to the Gaussian distribution. In genome-wide association studies (GWAS) on the number of vertebrae and teats in the Erhualian and Duroc populations, the allelic effect of each SNP on phenotypic traits was tested by using a general linear mixed model^27^ that included a random polygenic effect and a variance-covariance matrix proportionate to genome-wide identity-by-state^28^. The formula of the model is: **y** = **μ** + **Xb** + **s**c + **Za** + **e**, where **y** is the vector of phenotypes; **μ** is the overall mean; **b** is the vector of fixed effects including sex and batch effects; c is the effect of each SNP; a is the vector of random additive genetic effects with **a**~N(0, **G**σ_α_^2^), where **G** is the genomic relationship matrix calculated from the Illumina Porcine 60K SNP Beadchips and σ_α_^2^ is the polygenetic additive variance; **e** is the vector of residual errors with **e**~N(0, **I**σ_e_^2^), where **I** is the identity matrix and σ_e_^2^ is the residual variance;. **X** and **Z** are incidence matrices for b and a, respectively; **s** is the vector representing the SNP genotype for each individual. The GWAS were conducted by using the GenABEL package^24^. The genome-wide significance threshold was determined by the Bonferroni method, in which conventional *P*-value was divided by the number of tests performed^29^. A SNP was considered to have genome-wide significance at *P* < 0.05/N and chromosome-wide significance at *P* < 1/N, where N is the number of SNPs tested in the analyses. Quantile-quantile plots with genome control λ_GC_ values are shown in Supplementary Fig. 1. We found no evidence of systematic inflation of the GWAS results.

Associations between the *VRTN* mutation and phenotypes were evaluated using the following model identical to the GWAS model: **y** = **μ** + **Xb** + **s**c + **Za** + **e**, where **y** is the vector of phenotypic value of each trait, **μ** is the overall mean for each trait, **b** is the vector of fixed effects including sex and batch effects; c is the additive effect of the *VRTN* mutation; **a** is the vector of random additive genetic effects with **a**~N(0, **G**σ_α_^2^), where **G** is the genomic relationship matrix calculated from the 60K SNP markers in the F_2_, Erhualian and Duroc populations and σ_α_^2^ is the polygenetic additive variance; for the Landrace population that was not genotyped for the 60K Beadchips, **a** is the vector of random additive genetic effects with **a**~N(0, **A**σ_α_^2^), where **A** is the relationship matrix based on the pedigree of the Landrace population and σ_α_^2^ is the polygenetic additive variance; **e** is the vector of residual errors with **e**~N(0, **I**σ_e_^2^), where **I** is the identity matrix and σ_e_^2^ is the residual variance; **s** is the vector representing the genotype of the *VRTN* mutation for each individual; **X** and **Z** are incidence matrices for **b** and **a**, respectively.

### Introgression analysis

Two publicly available whole-genome sequence data sets were explored in the introgression analysis. One included whole-genome sequences (~ 25 × depth) of 69 Chinese pigs from 3 populations of wild boars and 11 geographically diverse breeds, including Bamaxiang, Luchuan, Wuzhishan, Erhualian, Laiwu, Min, Hetao, Tibetan (Gansu), Tibetan (Sichuan), Tibetan (Yunnan) and Tibetan (Tibet)^21^. The other contained whole-genome data (~ 8 × depth) of 55 European and Asian pigs from wild boars, Duroc, Hampshire, Pietrain, Landrace, Large White, Meishan, Jiangquhai, Xiang and Bearded pigs (*Sus barbatus*)^30^. The sequence reads of the two data sets are publicly available at the NCBI Sequence Read Archive under accession numbers SRA096093 and ERP001813. We first retrieved a 200 kb genomic sequence (Chr7: 103,357,506-103,567,075) flanking (100 kb up- and downstream) the *VRTN* gene from the *Sscrofa* 10.2 assembly ( http://www.animalgenome.org/repository/pig/Genome_build_10.2_mappings/). Then, we mapped clean pair-end sequence reads of the 69 Chinese pigs^21^ and the 55 European and Asian pigs^30^ to the 200 kb sequence using the Bowtie2 software^31^ with the parameters of “–fr–no-discordant–no-mixed–no-contain–no-overlap–no-unal”. Next, we selected mapped sam files by allowing at most 4 mismatches per read using in-house Perl scripts. Population based genotypes were further called by the ANGSD software^32^ under the parameters of “-GL 1 -doMaf 2 -doMajorMinor 1 -doGeno 5 -doPost 1 -postCutoff 0.95”. “-GL 1” represents that the SAMtools model was used to call SNPs. “-doMaf 2” implicates that the major allele was assumed to be known (inferred or given by user) while the minor allele was not determined. “-doMajorMinor 1” means that the major and minor allele can be inferred directly from likelihoods using a maximum likelihood approach to choose the major and minor alleles. “-doGeno 5” indicates that the major and minor alleles followed by the genotypes (AA, AC …) for each individual. “-doPost 1” means that the posterior probability of each genotype was estimated based on the allele frequency as a prior. “-postCutoff 0.95” implicates that a genotype with a posterior above this threshold would be called. The final set of SNP data were obtained by filtering with parameters of maf (minor allele frequencies) >0.01 and geno (genotype call rates) >0.9. Haplotypes were inferred for the *VRTN* gene (9 kb) and the 200 kb region excluding the *VRTN* gene via the SHAPEIT2 program^33^, respectively. First, haplotypes were reconstructed for the 69 Chinese pigs and 55 European and Asian pigs with all 1,695 SNPs in the 200 kb region including the *VRTN* gene (see supplementary file). Haplotypes harboring the mutant allele (*ins*) at the *VRTN* mutation site were defined as Q-type haplotypes, and those harboring the wild-type allele were defined as q-type haplotypes. Two sets of haplotypes were further determined from each inferred haplotype: one corresponded to the 9 kb region containing 25 SNPs, and the other associated with the 200 kb flanking region containing 1,670 SNPs. Major haplotypes with frequencies of greater than 0.04 (10/248) were finally explored to construct maximum likelihood (ML) phylogenetic trees using 1,000 bootstraps via MEGA 6.0^34^.

## Results and Discussion

### The *VRTN* mutation is segregating in both Chinese Erhualian and European commercial breeds

In our previous study, we reported the frequencies of the *VRTN* mutation on 1,371 pigs representing 20 diverse breeds and wild boars^13^. Here we genotyped this mutation in a larger sample of 3,827 pigs from the Chinese Erhualian breed and 3 European commercial breeds (i.e. Duroc, Large White and Landrace). As expected, we observed a similar distribution pattern of the *VRTN* allele frequencies in the present study compared to the previous report (Table 1). We found that both Chinese Erhualian and European breeds are segregating for this mutation, while the mutant (*ins*) allele associated with more vertebral number predominantly exist in European commercial breeds (Duroc: 0.59; Large White: 0.65; Landrace: 0.82). Interestingly, the Landrace breed that is known for long body length has the highest frequency (82%) of the mutant (*ins*) allele. This indicates that strong selection for body length in Landrace pigs likely enhanced the frequency of the mutant allele of *VRTN* that is significantly associated with thoracic vertebral number.

### The *VRTN* mutation is associated with thoracic vertebral number in Erhualian pigs

**Figure 1:**
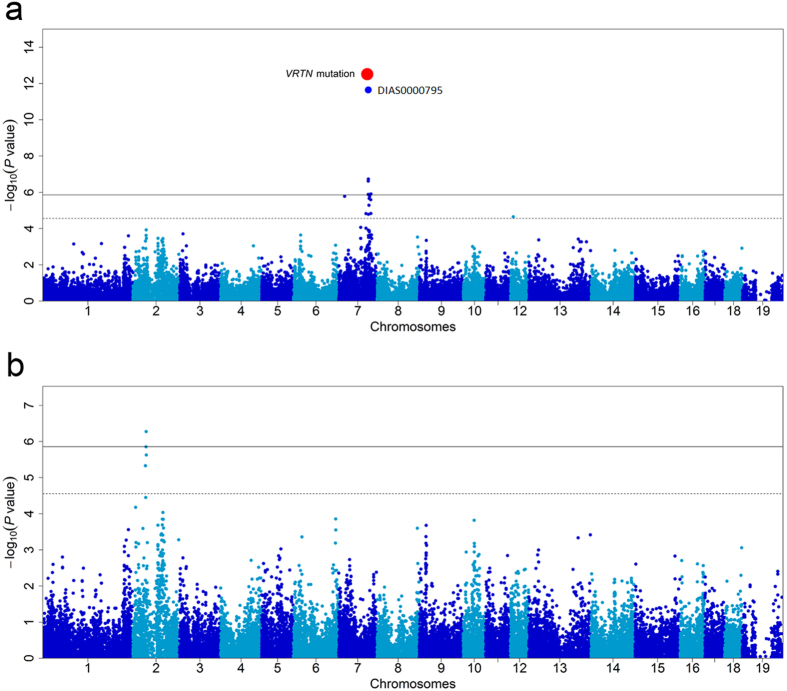
GWAS mapping for the number of thoracic vertebrae in the Erhualian population. (**a**) Manhattan plots of the GWAS for the number of thoracic vertebrae in the Erhualian population. In the Manhattan plots, negative log_10_ *P* values of the quantified SNPs were plotted against their genomic positions. Different colors indicate different chromosomes. The red dot represents the *VRTN* mutation, and the top GWAS SNP (DLAS0000795) on the Illumina Porcine 60K Beadchips is indicated. The solid and dashed lines indicate the 5% genome-wide and chromosome-wide Bonferroni-corrected thresholds, respectively. (**b**) When the *VRTN* mutation was include as a fixed effect in the GWAS model, no other SNP on SSC7 showed association signal.

To test if the *VRTN* mutation is associated with vertebral number in Chinese indigenous pigs, we performed a genome-wide association study (GWAS) on vertebral number in Erhualian pigs. After quality control, a total of 35,974 SNPs were included for the GWAS on 332 Erhualian pigs. The genome-wide and chromosome-wide significant thresholds were 1.39E-06 (0.05/35,974) and 2.78E-05 (1/35,974), respectively. SSC7 contained the most significant locus (*P* = 1.80E-12) for thoracic vertebral number, with the top SNP (DIAS0000795) at 103.6 Mb (Fig. 1a), 127.7 kb downstream of the *VRTN* gene. We then genotyped all 332 individuals for the *VRTN* mutation and included the *VRTN* genotypes in the GWAS model. The *VRTN* mutation appeared to be the top marker associated with the number of thoracic vertebrae (*P* = 3.14E-13, Fig. 1a), having a stronger strength of association than the original GWAS top SNP (DIAS0000795). The heterozygous (*ins/del*) pigs (14.34 ± 0.07) have more vertebrae than homozygous (*del/del*) animals (13.91 ± 0.02) (Table 2). However, this locus was not associated with lumbar vertebral number (Supplementary Fig. 2). When the *VRTN* mutation was included as a fixed effect in the GWAS model, the SSC7 QTL effects on thoracic vertebral number vanished in the Erhualian population (Fig. 1b). This finding favors the assumption that the *VRTN* mutation has a causative effect on thoracic vertebral number in the Erhualian breed.

### The *VRTN* mutation is associated with teat number in divergent pig populations

In this study, we found a significantly positive correlation (*r* = 0.32) between teat number and the number of thoracic vertebrae in the F_2_ cross (Supplementary Fig. 3). We have previously identified a significant QTL for teat number around the *VRTN* region in the F_2_ intercross^35^. To test if the *VRTN* mutation has pleiotropic effects on teat number, we investigated the association between the *VRTN* mutation and the number of teats in the F_2_, Erhualian, Duroc and Landrace populations (Table 2).

We observed a significant (*P* = 1.61E-04) association between *VRTN* genotype and teat number in the F_2_ intercross. The average teat number of *ins/ins* animals (17.59 ± 0.07) is greater than that of *del/del* animals (16.59 ± 0.11). When we included the *VRTN* mutation in the GWAS, it was the most significant marker for teat number on SSC7 and exhibited the same strength of association with the top SNP on the Illumina 60K Beadchips (data not shown).

In the Erhualian population, the *ins/del* pigs had more (*P* = 0.03) teats than *del/del* pigs (20.81 ± 0.23 vs. 20.31 ± 0.10). In the Landrace pigs, the number of teats in this population was also significantly associated with the *VRTN* mutation (*P* = 7.21E-04), and the *ins/ins* pigs (12.71 ± 0.04) have more teats than *ins/del* pigs (12.35 ± 0.08).

**Figure 2:**
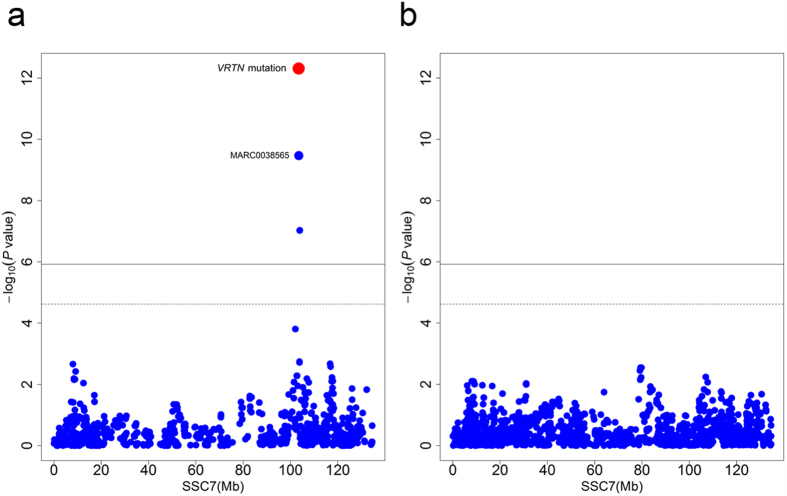
Regional association plot for the number of teats in the Duroc population. (**a**) Association of the *VRTN* mutation and 60K chip SNPs on SSC7 with the number of teats in the Duroc population. Negative log_10_ *P* values of the quantified SNPs were plotted against their genomic positions. The red dot represents the *VRTN* mutation, and the top GWAS chip SNP (MARC0038565) is indicated. The solid and dashed lines indicate the 5% genome-wide and chromosome-wide Bonferroni-corrected thresholds, respectively. (**b**) When the *VRTN* mutation was include as a fixed effect in the GWAS model, no other SNP on SSC7 showed association signal.

We performed GWAS for teat number in the Duroc population. After quality control, a total of 41,793 SNPs were included for the GWAS on 830 Duroc pigs. The genome-wide and chromosome-wide significant thresholds were 1.20E-06 (0.05/41,793) and 2.39E-05 (1/41,793), respectively. A genome-wide significant association with teat number was observed for SNP MARC0038565 at 103.49 Mb on SSC7 (*P* = 3.40E-10, Fig. 2a), which was 28 kb downstream of the *VRTN* gene. When we included the *VRTN* mutation in the GWAS, the mutation stood out to be the most significantly associated marker (Fig. 2a). The average teat number of *ins/ins* animals (11.21 ± 0.09) is greater than that of *del/del* animals (10.42 ± 0.06). When *VRTN* genotype was included as a fixed effect in the GWAS model, the association signal on SSC7 disappeared in the Duroc population (Fig. 2b). This supports the conclusion that the *VRTN* mutation has pleiotropic effects on teat number.

### The *VRTN* mutation is also significantly associated with body length

The significant association of the *VRTN* variation with body length and its related traits has been reported in two Duroc populations^14^,^16^. Here we tested the association between the *VRTN* mutation and body (carcass) length in the F_2_, Erhualian, Duroc and Landrace populations (Table 2).

In the F_2_ cross population, we detected a significant (*P* = 0.04) difference in carcass length between animals with the *ins* allele and those with the wild-type allele. In the Erhualian population, significant associations of the *VRTN* mutation with body length were also observed (*P* = 0.03). The heterozygous (*ins/del*) pigs have 44.4 mm longer body length than homozygous (*del/del*) animals (115.55 ± 1.71 vs. 111.11 ± 0.62). In the Duroc population, the *VRTN* mutation was strongly (*P* = 8.95E-35) associated with body length, with an additive effect of 7.5 mm. In the Landrace population, a tendency towards longer body length was found in *ins/ins* pigs compared to *ins/del* individuals, although the difference did not reach statistical significance (*P* = 0.14), possibly due to a low frequency (7.8%) of the *del* allele in the population.

### The *VRTN* mutation has no effect on fatness and production traits

There are contradictory reports about the association of the *VRTN* mutation with intramuscular fat content^14^,^15^,^16^. We herein analyzed the relationship between *VRTN* genotype and two fatness traits (IMF and backfat thickness) and one production trait (ADG) that were recorded in 928 F_2_ individuals from the White Duroc × Erhualian intercross, 332 Erhualian pigs, 666 Duroc pigs and 596 Landrace pigs. We did not detect any significant association between the *VRTN* mutation and IMF, backfat thickness and ADG in the tested populations (Table 2). This indicates that selection for the favorable allele at the *VRTN* mutation site would not have undesirable effects on growth and fatness traits in pigs.

### The *VRTN* mutation was possibly introgressed from Chinese pigs into European pigs

**Figure 3:**
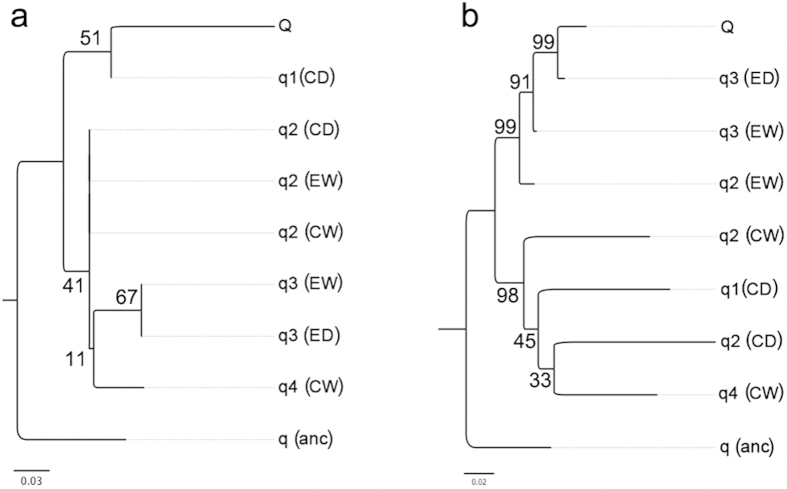
Possible introgression of the *VRTN* haplotype from Chinese pigs into European pigs. (**a**) Maximum likelihood (ML) phylogenic tree for the 9 kb region containing the *VRTN* gene. (**b**) ML phylogenic tree for the 200 kb region flanking (not including) the *VRTN* gene. Haplotypes of the two regions were first inferred, and then the two ML phylogenic trees were built for major haplotypes with frequencies of greater than 0.04 (see Methods). Q denotes the major haplotype harboring the Q allele (the mutant allele, *ins*), q denotes major haplotypes containing the q allele (the wild-type allele, *del*). EW, European wild boar; ED, European domestic pig; CW, Chinese wild boar; CD, Chinese domestic pig; anc, Beared pigs (*Sus barbatus*) as an outgroup to European and Chinese pigs. Scale bars represent the number of nucleotide substitutions per SNP site. Values in the tree indicate percentages (%) of observations in 1,000 bootstrap replicates.

To test the hypothesis that the *VRTN* mutation has been introgressed from Chinese pigs into European pigs, we performed the ML phylogenetic analysis for the *VRTN* gene and its flanking region using whole-genome sequence data for 124 Chinese and European pigs. We first inferred haplotypes of the two regions for the 124 pigs (see Methods). Only major haplotypes with frequencies of greater than 0.04 (10/248) were then explored to construct ML phylogenetic trees (Fig. 3). For the 200 kb region flanking (not including) the *VRTN* gene, all haplotypes of Chinese origin formed a branch, while all haplotypes of European origin defined another branch in the ML tree (Fig. 3b). This is consistent with the evolutionary split between Chinese and European pigs^30^. In the *VRTN* region, the Q-type haplotype containing the mutant allele (*ins*) at the *VRTN* mutation site was mainly from European domestic pigs (n = 22) and few from Chinese domestic pigs (n = 3). Surprisingly, the Q-type haplotype clustered with a q-type (wild-type) from Chinese domestic pigs (n = 51). All other three q-type haplotypes including q2 (n = 62), q3 (n = 63) and q4 (n = 11) from European and Chinese pigs defined another clade, which was separated with a long branch from the clade containing the Q-type haplotype. The haplotype of Beared pigs (*Sus barbatus*) appeared as a clear outgroup to all European and Chinese haplotypes (Fig. 3a). This observation suggests the introgression hypothesis, i.e, the *VRTN* Q-haplotype that carries the QTN allele increasing vertebral number was originated from Chinese pigs and may have been historically introgressed into European pigs. After the introgression event, the Q haplotype could have been selected for pork production, leading to significantly higher frequencies of the Q haplotype in European commercial breeds like Landrace, Large White and Duroc compared to Chinese indigenous pigs. It should be mentioned that the number of SNPs (n = 25) within the *VRTN* gene was much lower than that (n = 1,670) in the 200 kb flanking region due to the small size of the *VRTN* gene (9 kb). This may contributed to lower bootstrap values in the ML tree for the *VRTN* region compared with those in the ML tree for the 200 kb flanking region (Fig. 3). The low bootstrap support indicated that the two ML trees were not so definitive. Further investigations are required to confirm the introgression hypothesis.

## Additional Information

**How to cite this article**: Yang, J. *et al.* Possible introgression of the *VRTN* mutation increasing vertebral number, carcass length and teat number from Chinese pigs into European pigs. *Sci. Rep.* **6**, 19240; doi: 10.1038/srep19240 (2016).

## Supplementary Material

Supplementary Information

## Figures and Tables

**Table 1:** The frequencies of the *VRTN* mutation (g.20311_20312ins291) in four purebred pigs.

Breed		Genotype frequency		Allele frequency	
	No. ofanimals	−/−	*ins*/−	*ins*/*ins*	*ins*
Erhualian	332	0.87 (288)	0.13 (44)	0.00 (0)	0.07
Duroc	1050	0.30 (319)	0.49 (510)	0.21 (221)	0.59
Large White	1348	0.13 (170)	0.44 (595)	0.43 (583)	0.65
Landrace	1097	0.05 (51)	0.26 (282)	0.70 (764)	0.82

Frequency of each genotype and the mutant allele (*ins*) associated with increased vertebral number at the *VRTN* mutation site are shown in this table. The number of pigs within each genotype is given in parentheses.

**Table 2:** Association of the *VRTN* mutation (g.20311_20312ins291) with economically important traits in four pig populations.

Trait	No.	Phenotypic value			*P* value
		*del/del*	*ins/del*	*ins/ins*	
White Duroc × Erhualian F_2_ intercross					
TVN	927	14.17 ± 0.04(130)	14.91 ± 0.02(454)	15.49 ± 0.03(343)	2.08E-16
CL	925	94.82 ± 0.66(130)	96.10 ± 0.32(453)	96.64 ± 0.42(342)	0.04
TN	925	16.59 ± 0.11(129)	17.18 ± 0.07(452)	17.59 ± 0.07(344)	1.61E-04
BW1	927	95.14 ± 1.72(130)	97.66 ± 0.80(454)	97.66 ± 0.98(343)	0.40
ADG	925	433.65 ± 8.29(129)	447.74 ± 3.90(453)	447.46 ± 4.85(343)	0.38
IMF	852	2.32 ± 0.11(117)	2.14 ± 0.05(418)	2.14 ± 0.06(317)	0.06
BF	928	2.83 ± 0.08(130)	3.00 ± 0.04(454)	3.12 ± 0.05(344)	0.85
Erhualian					
TVN	332	13.91 ± 0.02(288)	14.34 ± 0.07(44)	—	1.48E-17
BL	319	111.11 ± 0.62(277)	115.55 ± 1.71(42)	—	0.03
TN	320	20.31 ± 0.10(277)	20.81 ± 0.23(43)	—	0.03
BW2	332	83.098 ± 0.91(288)	89.245 ± 2.27(44)	—	0.02
IMF	276	0.031 ± 0.00(236)	0.035 ± 0.00(40)	—	0.56
BF	276	3.372 ± 0.05(236)	3.543 ± 0.09(40)	—	0.65
Duroc					
BL	666	115.95 ± 0.19(247)	116.81 ± 0.17(329)	117.44 ± 0.33(90)	8.95E-35
TN	833	10.42 ± 0.06(268)	10.69 ± 0.05(410)	11.21 ± 0.09(155)	3.40E-10
ADG	833	975.20 ± 6.66(268)	959.71 ± 5.31(410)	964.28 ± 7.72(155)	0.90
IMF	833	1.65 ± 0.04(268)	1.69 ± 0.03(410)	1.67 ± 0.05(155)	0.14
BF	833	10.75 ± 0.12(268)	10.69 ± 0.10(410)	10.62 ± 0.13(155)	0.36
Landrace					
BL	596	—	116.66 ± 0.37(93)	117.24 ± 0.14(503)	0.14
TN	538	—	12.35 ± 0.08(84)	12.71 ± 0.04(454)	7.21E-04
ADG	595	—	852.60 ± 8.13(93)	847.32 ± 3.23(502)	0.45
BF	595	—	13.44 ± 0.14(93)	13.48 ± 0.07(502)	0.68

TVN, thoracic vertebral number; CL, carcass length; TN, teat number; ADG, average daily gain; IMF, intramuscular fat content; BF, backfat thickness; BL, body length; BW1, body weight at 240 ± 3 days; BW2, body weight at 300 ± 3 days. Not all traits have been measured in four pig populations. Phenotypic values are shown in mean ± standard error. The number of individuals within each genotype is given in brackets.

## References

[b1] Narita Y. & Kuratani S. Evolution of the vertebral formulae in mammals: a perspective on developmental constraints. J Exp Zool B Mol Dev Evol 304, 91–106 (2005). 15660398 10.1002/jez.b.21029

[b2] King J. & Roberts R. Carcass length in the bacon pig: its association with vertebrae numbers and prediction from radiographs of the young pig. Anim Prod 2, 59–65 (1960).

[b3] Wang L. *et al.* In Animal genetic resources in China: pigs . (eds China National Commission of Animal Genetic Resources) 2–16 (China Agricultural Press, 2011).

[b4] Borchers N., Reinsch N. & Kalm E. The number of ribs and vertebrae in a Pietrain cross: variation, heritability and effects on performance traits. J Anim Breed Genet 121, 392–403 (2004).

[b5] Ciobanu D. C., Lonergan S. M. & Huff-Lonergan E. J. Genetics of meat quality and carcass traits In The genetics of the pig 2nd edn (eds Rothschild M. & Ruvinsky A. ) Ch. **15**, 355–389 (CABI, 2011).

[b6] Wada Y. *et al.* Quantitative trait loci (QTL) analysis in a Meishan x Gottingen cross population. Anim Genet 31, 376–384 (2000). 11167524 10.1046/j.1365-2052.2000.00696.x

[b7] Ren D. R. *et al.* Mapping and fine mapping of quantitative trait loci for the number of vertebrae in a White Duroc x Chinese Erhualian intercross resource population. Anim Genet 43, 545–551 (2012). 22497517 10.1111/j.1365-2052.2011.02313.x

[b8] Edwards D. B. *et al.* Quantitative trait locus mapping in an F2 Duroc x Pietrain resource population: II. Carcass and meat quality traits. J Anim Sci 86, 254–266 (2008). 17965326 10.2527/jas.2006-626

[b9] Uemoto Y. *et al.* Quantitative trait loci analysis on Sus scrofa chromosome 7 for meat production, meat quality, and carcass traits within a Duroc purebred population. J Anim Sci 86, 2833–2839 (2008). 18567733 10.2527/jas.2007-0293

[b10] Mikawa S. *et al.* Identification of a second gene associated with variation in vertebral number in domestic pigs. BMC Genet 12, 5 (2011). 21232157 10.1186/1471-2156-12-5 PMC3024977

[b11] Mikawa S. *et al.* Fine mapping of a swine quantitative trait locus for number of vertebrae and analysis of an orphan nuclear receptor, germ cell nuclear factor (NR6A1). Genome Res 17, 586–593 (2007). 17416745 10.1101/gr.6085507 PMC1855175

[b12] Rubin C. J. *et al.* Strong signatures of selection in the domestic pig genome. Proc Natl Acad Sci USA 109, 19529–19536 (2012). 23151514 10.1073/pnas.1217149109 PMC3511700

[b13] Fan Y. *et al.* A further look at porcine chromosome 7 reveals *VRTN* variants associated with vertebral number in Chinese and Western pigs. PLoS One 8, e62534 (2013). 23638110 10.1371/journal.pone.0062534 PMC3634791

[b14] Hirose K. *et al.* Association of swine vertnin (*VRTN*) gene with production traits in Duroc pigs improved using a closed nucleus breeding system. Anim Sci J 84, 213–221 (2013). 23480701 10.1111/j.1740-0929.2012.01066.x

[b15] Hirose K. *et al.* Evaluation of effects of multiple candidate genes (LEP, LEPR, MC4R, PIK3C3, and *VRTN*) on production traits in Duroc pigs. Anim Sci J 85, 198–206 (2014). 24128088 10.1111/asj.12134

[b16] Nakano H. *et al.* Effect of *VRTN* gene polymorphisms on Duroc pig production and carcass traits, and their genetic relationships. Anim Sci J 86, 125–131 (2015). 25187328 10.1111/asj.12260

[b17] Ojeda A. *et al.* Evolutionary study of a potential selection target region in the pig. Heredity 106, 330–338 (2011). 20502482 10.1038/hdy.2010.61 PMC3183885

[b18] Wilkinson S. *et al.* Signatures of diversifying selection in European pig breeds. PLoS Genet 9, e1003453 (2013). 23637623 10.1371/journal.pgen.1003453 PMC3636142

[b19] Bosse M. *et al.* Genomic analysis reveals selection for Asian genes in European pigs following human-mediated introgression. Nat Commun 5, 4392 (2014). 25025832 10.1038/ncomms5392 PMC4225517

[b20] Guo Y. *et al.* A linkage map of the porcine genome from a large-scale White Duroc x Erhualian resource population and evaluation of factors affecting recombination rates. Anim Genet 40, 47–52 (2009). 19178432 10.1111/j.1365-2052.2008.01802.x

[b21] Ai H. *et al.* Adaptation and possible ancient interspecies introgression in pigs identified by whole-genome sequencing. Nat Genet 47, 217–225 (2015). 25621459 10.1038/ng.3199

[b22] Liu X. *et al.* Genome-wide association analyses for meat quality traits in Chinese Erhualian pigs and a Western Duroc x (Landrace x Yorkshire) commercial population. Genet Sel Evol 47, 44 (2015). 25962760 10.1186/s12711-015-0120-x PMC4427942

[b23] Qiao R. *et al.* Genome-wide association analyses reveal significant loci and strong candidate genes for growth and fatness traits in two pig populations. Genet Sel Evol 47, 17 (2015). 25885760 10.1186/s12711-015-0089-5 PMC4358731

[b24] Aulchenko Y. S., Ripke S., Isaacs A. & van Duijn C. M. GenABEL: an R library for genome-wide association analysis. Bioinformatics 23, 1294–1296 (2007). 17384015 10.1093/bioinformatics/btm108

[b25] Yang B. *et al.* Genome-wide association analyses for fatty acid composition in porcine muscle and abdominal fat tissues. PLoS One 8, e65554 (2013). 23762394 10.1371/journal.pone.0065554 PMC3676363

[b26] Shapiro S. S. & Wilk M. B. An analysis of variance test for normality (complete samples). Biometrika , 52, 591–611 (1965).

[b27] Yu J. *et al.* A unified mixed-model method for association mapping that accounts for multiple levels of relatedness. Nat Genet 38, 203–208 (2006). 16380716 10.1038/ng1702

[b28] Hayes B. J. & Goddard M. E. Technical note: prediction of breeding values using marker-derived relationship matrices. J Anim Sci 86, 2089–2092 (2008). 18407982 10.2527/jas.2007-0733

[b29] Yang Q., Cui J., Chazaro I., Cupples L. A. & Demissie S. Power and type I error rate of false discovery rate approaches in genome-wide association studies. BMC Genet 6 Suppl 1, S134 (2005). 16451593 10.1186/1471-2156-6-S1-S134 PMC1866802

[b30] Groenen M. A. *et al.* Analyses of pig genomes provide insight into porcine demography and evolution. Nature 491, 393–398 (2012). 23151582 10.1038/nature11622 PMC3566564

[b31] Langmead B. & Salzberg S. L. Fast gapped-read alignment with Bowtie 2. Nat Methods 9, 357–359 (2012). 22388286 10.1038/nmeth.1923 PMC3322381

[b32] Korneliussen T., Albrechtsen A. & Nielsen R. ANGSD: Analysis of Next Generation Sequencing Data. BMC Bioinformatics 15, 356 (2014). 25420514 10.1186/s12859-014-0356-4 PMC4248462

[b33] Delaneau O., Zagury J. F. & Marchini J. Improved whole-chromosome phasing for disease and population genetic studies. Nat Methods 10, 5–6 (2013). 23269371 10.1038/nmeth.2307

[b34] Tamura K., Stecher G., Peterson D., Filipski A. & Kumar S. MEGA6: Molecular Evolutionary Genetics Analysis version 6.0. Mol Biol Evol 30, 2725–2729 (2013). 24132122 10.1093/molbev/mst197 PMC3840312

[b35] Ding N. *et al.* Genome-wide QTL mapping for three traits related to teat number in a White Duroc x Erhualian pig resource population. BMC Genet 10, 6 (2009). 19226448 10.1186/1471-2156-10-6 PMC2672953

