# Supplementary material for: Possible introgression of the VRTN mutation increasing vertebral number, carcass length and teat number from Chinese pigs into European pigs
Source: Sci Rep. 2016 Jan 19;6:19240. doi: 10.1038/srep19240 (PMC4726066; doi:10.1038/srep19240)
Supplement: Supplementary Information [file srep19240-s1.pdf]

Supplementary Information of:

Possible introgression of the *VRTN* mutation increasing vertebral number, carcass length and teat number from Chinese pigs into European pigs

Jie Yang[1,2], Lusheng Huang[2], Ming Yang[3], Yin Fan[2], Lin Li[2], Shaoming Fang[2], Wenjiang Deng[2], Leilei Cui[2], Zhen Zhang[2], Huashui Ai[2], Zhenfang Wu[1,3], Jun Gao[2]*, Jun Ren [2]*

[1] National Engineering Research Center for Breeding Swine Industry, South China Agricultural University, Guangdong, P.R. China

[2] State Key Laboratory for Pig Genetic Improvement and Production Technology, Jiangxi Agricultural University, Nanchang, P.R. China

[3] National Engineering Research Center for Breeding Swine Industry, Guangdong Wens Foodstuffs Group Co.,Ltd, Guangdong, P.R. China

*Correspondence to: Jun Ren and Jun Gao.

E-mail: renjunjxau@hotmail.com; jungaochina@hotmail.com

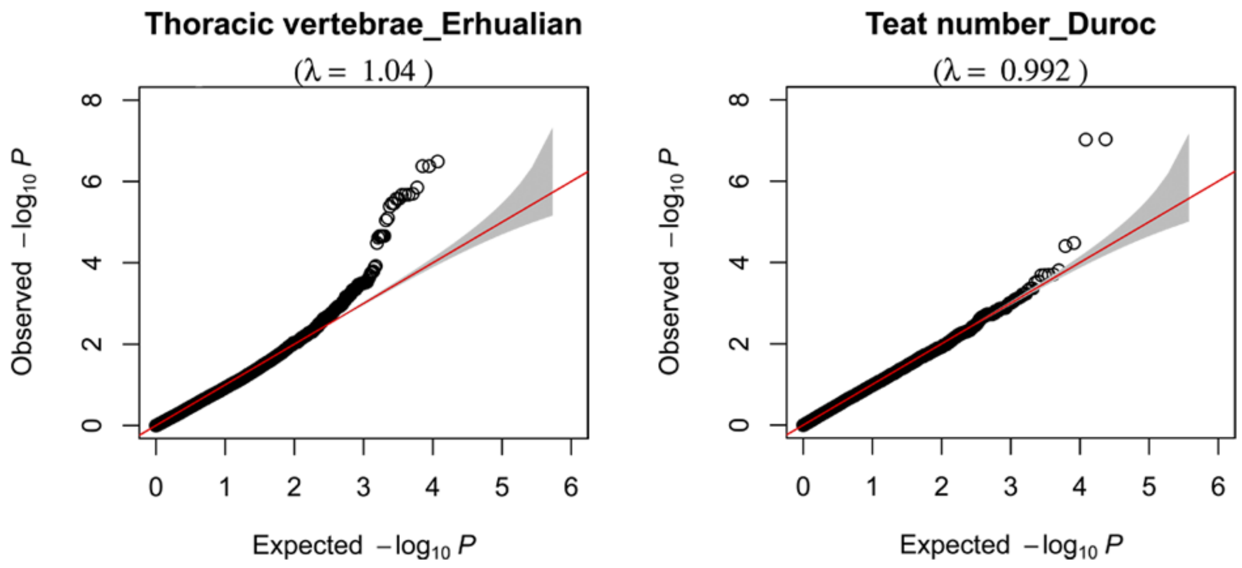

Supplementary Fig. 1

Quantile-quantile plot of SNPs after quality control in genome-wide association studies on the Erhualian and Duroc populations.

Yang *et al.*, Supplementary Fig. 1

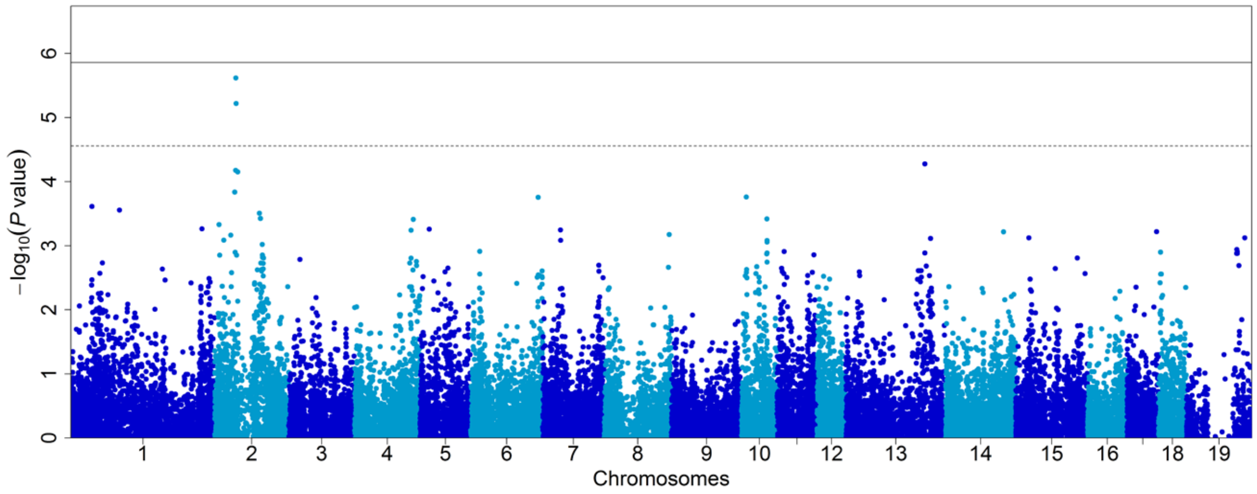

Supplementary Fig. 2

GWAS mapping for the number of lumbar vertebrae in the Erhualian population. SNPs on different chromosomes are denoted by different colors. The solid and dashed lines indicate the 5% genome-wide and suggestive Bonferroni-corrected thresholds, respectively.

Yang *et al.*, Supplementary Fig. 2

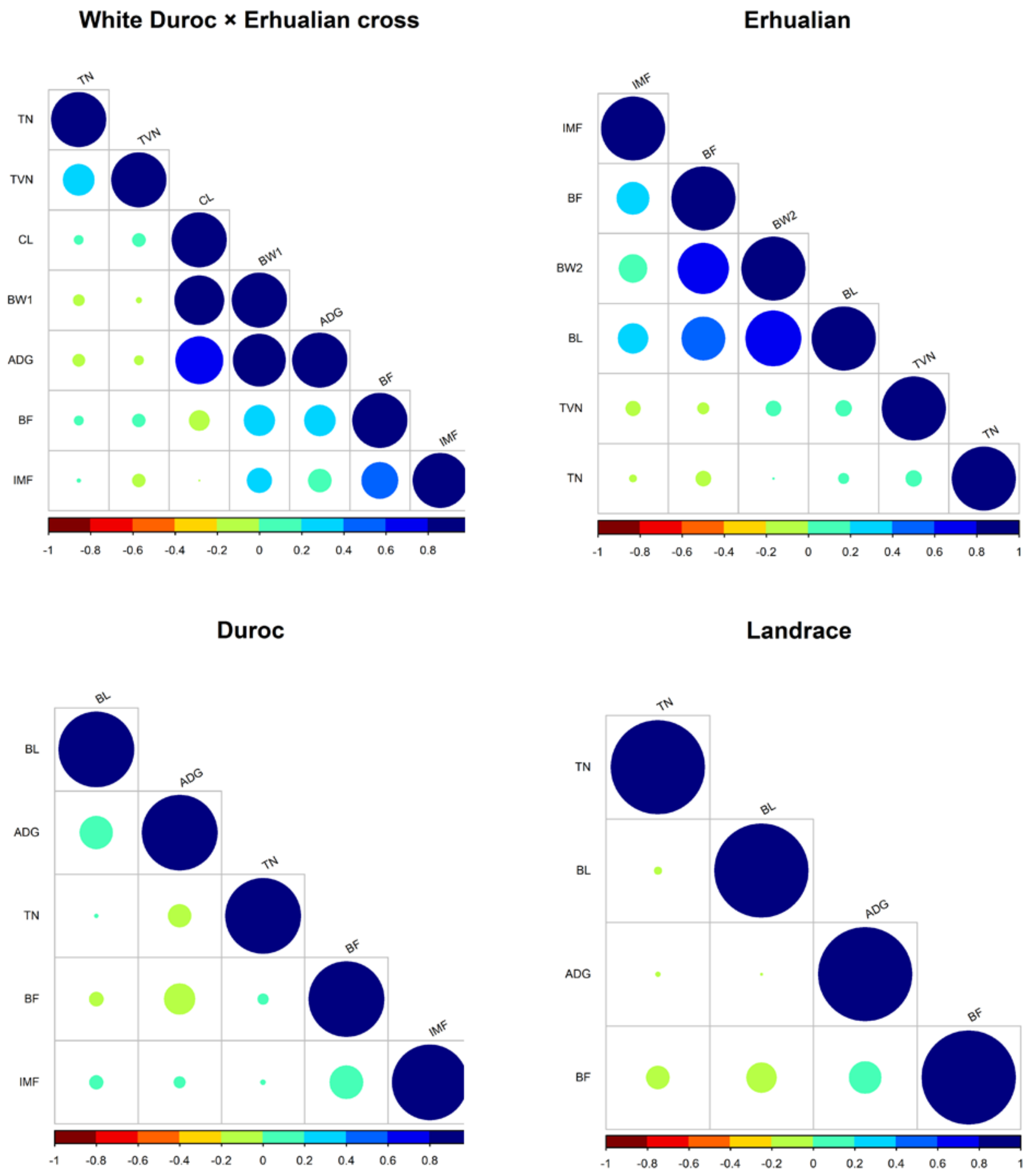

Supplementary Fig. 3

Correlation plots between economically important traits in four Chinese and European pig populations. The sizes and colors of the dots, respectively, represent the degree and direction (positive or negative) of the correlation coefficient. The larger the dot, the greater the correlation coefficient.

Yang *et al.*, Supplementary Fig. 3

Supplementary dataset

Haplotypes of 124 pigs in the *VRTN* gene and its 200 kb flanking region. The 25 nucleotides at positions 425 to 449 in the following haplotypes correspond to SNPs of the *VRTN* gene, and the remaining 1,670 nucleotides represent SNPs of the 200 kb region flanking the *VRTN* gene. Each individuals have two haplotypes. Two sets of haplotypes were further determined from each inferred haplotype for phylogenetic analysis: one corresponded to the *VRTN* gene containing the 25 SNPs, and the other corresponded to the 200 kb region excluding the *VRTN* gene containing the 1,670 SNPs.

>EHL_ER_CS0234.1

GTCGGGATGCCTTCCCAACTCCGGCACGGCGCGGCGCCTTCGCGCACGGATCGGATAA
GCTTACCCGTGGATGGCTCGTTCTCAGAAATATCTCGTAGTCTTCTCGTTGGTTACTCCA
ATGAAGCGCCTCGTCTGCGGGGGCACTGAACCGCGAGAGCTATTCAAGTGCTCTACTA
CTCGACCGGGGGCAGCTTACAACCGCGGGATTGGCACGGTCTACCTCCGTCCAACAGG
GGGTACGACGACCCGGTCCCCCCTCCCGCGTCGAAGGCTGCCAACACGATAGTAAGCG
AACTGGCCCAAACGTATTAATACGTACAGGGGGCGGGCCTCTGGCGCGCCACTGGATC
AGGCCCGTGGCGTGCCCGCCTCGTCAGCGCCACCCATTGCTAAGCGCTGACAGTAATA
GACCCCTCCATAGTAGTTGCCGATGTTGATTCGGTCACCGGCCGAAACGCGCGCGCTC
AGCACAAGGCAGGTACCACGGAGCGAAAGGTGGATGATTGGAAGGGGCTGCTGACGC
GCCTATCAGCCCGTTCCCCCGCGCCTGCTGTGGCGACCAACTACGCCCCGCAGCGTCCA
GTACCCGAGCAGTCTCTCAACTGGTCCGATTAGACTGTATACACCGCCGTTGGGACGC
GGACTAAACAACTCCCTCATACCCATCCGCCCGTCCGGAGCGGAACGACTCGGCGGTA
CCGGCGTCAGGCCCCCTCGCCTAGACTGCACCATTATGTTGGGAGGTGCGTCGACTGG
GGGCCGGCGACTTGATCCTGGTCGGCCCGTGAAGCCCCTCAGTCCCATGGAGACGTTC
TCTCCCATCTATTGGCCTCCGGGGCTCTCCACCAATCGCACCGGAGTCTTGTCTAATAT
GCAATTTATTCATCGTGAGGTATAAACCGCCCCCGAGTGGGTGCGGCTTGAAGTCCAG
CCCCCCAGGCCGCCTGCTAACACGGAAGTGTACCCGGTTCCACTCAGAGGGCATGAGG
CAGTACCTACGGATCTCAACAAGCCTAGTCCCCATGATACGTACAGCTGGGCCAGGTG
GGACTAATACGCCGGGGTCTCGTTGCGGAACGGCTAGAAGGTAAATACGCCCTGGCCA
TATACTCTCTGTCCGCATCCCTTGGGGACATCCATTATGCTGCCGTTGACGTCGTCAAC
GTCACGCCACTAACACCGAACTAGGGGGACCAAACTATACCATGTTATCCGTATACTT
ACCCACTGTTGCAGGTCAAGGTATGGGGCTGCAAGTAATGTACATACTCCTGGTGGGA
ACTTGTCCTCCGGACTTGAATGGCTACCTGCCGGGCTGGGACTCAGCAAACCACCCTG
ACGCATCCCAGGCCTTTGATCGGATCACAATTTTGATTCCACCAGTTCCCAATTACGGT
TCCGCATCGTTGGGAGGCCTTTGGCTAGATCTACCTGCATGGTTCGAGCGGTAATATCG
GGTCTGAAATCCCCTGACCCAACGGGAGGCAGTATGTGTGGTCGGAATCTGCACCTTG
CGAGAGATAATCGTAATGACGATGGACCTTCTACAAGTGAGTGCTGGGGCACGAGCGG
CGGACCAGATGGTCTTCTGAATGTTCCTTCCCGTGCCGAAGGCTGAACTCGAGGCGGA
GCAGTGGGCGCGTACCGTCGACATGCAGGTGCACCGCGAGGTCCCTTGAAGATCCCGT
CATCAAC

>EHL_ER_CS0234.2

GTCGGGACGCCCTCCCGGCTCTGGCACGGCGCGGTGGCTTTGCGCACGGATCGGATAA
ACTTTCCATTGGACGGCTCGTTCTCAGAAATATCTCGTAGTCTTCTCGTTGGTTACTCCA
ATGAAACGCCTCGTCTGCGGGGGCACTGAACCGCGAGAGCTATTCAAATGCTCTACTA

CTCGACCGGGGGCAGCTTACAACCGTGGGATCGGCGCGGCCCACCTCCGGTCAACGAG
AGTTACGAGTTCCCGGCCCGTTTTCTCGCGTCGGAGGCTGCCAACACGATAGTAACTGA
ACTGGCCCAAACGTATTAATACGCCCCGGGGGCGGGCCTCTGGCGCGCCACTGGATCA
GGCCCGTGGCGTGCCCGCCTCGTCAGCGCCACCCATTGCTAAGCGCTGACAGTAATAG
ACCCCTCCATAGTAGTTGCCGATGTTGATTTGGTCACCGGCCGAAACGTATGCACTTAG
CACAGGGCAGGTACTACAAAGCGAGAGGGGGATGATTGGCAGGGGCTGCTGACGCGC
CTATCAGCCCGTTCCCCCGCGCCTGCTGTGGCGACCAACTACGCCCCGCAGTGACCGGT
ACCCGAGCAGTCTCTCAACTGGGTCAATTAGGTTGTATACATCCTTGTTGGGTCGCTGC
CTAAATAACCCCTTTACGCACATCCGCCCATCCGGAGCGGAACGACTTGGCGGAACCG
GCGTCAGACCCCCTCGCCTAGGCTGCACCATTATATTAGGCGGTGCGTCGACTGAGGG
CTAGCGACTTGATCCCGGTCGGCCCGTGAAGCCCATCAGTTCCATGGAGACGTTCGCTC
CCATCCGTTGGCCTCCGGGGCTCTCCACCAATCGCACCGGAGTCTTGTCAGATGTGAAA
TTTATTCATCGTGAGGTATAAACCGCCCCCGCGTGGGTGCGGCTCGATGTCTGGCCTCC
CAGGCTGCCTGCTAACACGAAAGTGTACCCGGTTCCACTCAGAAGGGACGAGACACTC
TCGACGGCTCTCAACAAGCCTAGTCCCCATGATACGTACAGCTGGGCCAGGTGGGATT
AATACGTCGGGGTCCCATCGCGGGATGTTTGAGGGGGAGATACGTTCCGGCCATATAC
TCTTTGTCCGCATCCCTTGGGGAAATCCATTACGCTGCCGTTGCCGTCGTTAACGTCAC
GCCACTAACACCGAACTAGGGGGACCAAACTATACCATGTTATCCGTATACTTACCCA
CTGTTGCAGTTCAGGGTACGGGGGTGTGAGTCATGGACGCGCTCCTGGTGGGAGCTTG
GCCTCTGAACTTGAATAACAGCTCACTGGAATGGGATTTAGCAAACCATCCCAACATA
TTCCAGGCCTCTGATCAGATCACAATTTTGCTTTCACCAGTTCCCAATTATGATTCCGCA
TCGTCGGGAGGCCTTCGACTAGATCTGCTCGCATGGTTCGAGCGGTAATATCGGGTCTG
AAATCCCCTGACCCAACGGGAGGCGGTATGCGTGATCGGAATCTGCACCTTGCGAGAG
CTAATCGTGATGACGATGGCTCTTCCACAAGTGAGTGCGGGGGCGCGAGCGGCGAACC
AGATGGTCCTCTGAATGTTCCCCCCCGTGCCGAAGGCTGAACTCGAGGCGGGGCCGTG
GTCGCGCACCGTTACCACGCAGGTGCGTCGCGAGGCCCCTTGAAGGTCCCGTCATGAA
G

>EHL_ER_CS3544.1

GTCGGGACGCCCTCCCGGCTCTGGCATGGCGCGGTGGCTTTGCGCACGGATCGGATAA
ACTTTCCATTGGACGGCTCGTTCTCAGAAATATCTCGTAGTCTTCTCGTTGGTTACTCCA
ATGAAACGCCTCGTCTGCGGGGGCACTGAACCGCGAGAGCTATTCAAGTGCTCTACTA
CTCGACCGGGGGCAGCTTACAACCGTGGGATCGGCGCGGCCCACCTCCGGTCAACGAG
AGTTACGAGTACCTGGTCCGTTTTCCCGCGTCGGAAGCTACCAACACGATAGTAACTG
AACCAGCGCAAACGTATTGATACGCCTCGGGGGCGGGCCTCTGGCGCACCGCTGGATC
AGGCCCGTGGCGTGCCCGCCTCGTCAGCGCCACCCATTGCTAAGCGCTGACAGTAATA
GACCCCTCCATAGTAGTTGCCGATGTTGATTCGGTCACCGGCCGAAACGTATGCACTTA
GCACAGGGCAGGTACTACAAAGCGAGAGGGGGATGATTGGCAGGGGCTGCTGACGCG
CCTATCAGCCCGTTCCCCCGCGCCTGCTGTGGCGACCAACTACGCCCCGCAGCGTCCAG
TACCCGAGCAGTCTCTCAACTGGTCCGATTAGACTGTATACACCGCCGTTGGGACGCG
GACTAAACAACTCCCTCATACCCATCCGCCCGTCCGGAGCGGAACGACTCGGCGGTAC
CGGCGTCAGGCCCCCTCGCCTAGACTGCACCATTATGTTGGGAGGTGCGTCGACTGGG
GGCCGGCGACTTGATCCTGGTCGGCCCGTGAAGCCCATCAGTCCCATGGAGACGTTCT
CTCCCATCTATTGGCCTCCGGGGCTCTCCACCAATCGCACCGGAGTCTTGTCTAATATG
AAATTTAAACATCGTGAGGTATAAACCGCCCCCGAGTGGGTGCGGCTTGAAGTCCAGC
CCCCCAGGCCGCCTGCTAACACGGAAGTGTACCCGGTTCCACTCAGAGGGCATGAGGC
AGTACCTACGGATCTCAACAAGCCTAGTCCCCATGATACGTACAGCTGGGCCAGGTGG
GATTAATACGCCGGGGTCCCGCCGCGAAACGGCTAGAAGGTAAATACGCCCTGGCCAT
ATACTCTTTGTCCGCATCCCTTGGGGACATCAATTATGCTGCCGTTGACGTCGTCAACG
TCACGCCACTAACACCGAACTAGGGGGACCAAACTATACCATGTTATCCGTATACTTA
CCCACTGTTGCAGGTCAAGGTATGGGGCTGCAAGTAATGTACATACTCCTGGTGGGAA

CTTGTCCTCCGGACTTGAATGGCTACCTGCCGGGCTGGGACTTAGCAAACCACCCTGAC
GCATCCCAGGCCTTTGATCGGATCACAATTTTGATTCCACCAGTTCCCAATTACGGTTC
CGCATCGTTGGGAGGCCTTTGGCTAGATCTACCTGCATGGTTCGATCGGTAATATCGGG
TCTGAAATCCCCTGACCCAACGGGAGGCAGTATGTGTGGTCGGAATCTGCACCTTGCG
AGAGATAATCGTAATGACGATGGACCTTCTACAAGTGAGTGCTGGGGCACGAGCGGCG
GACCAGATGGTCTTCTGAATGTTCCTTCCCGTGCCGACGGCTGAACTCGAGGCGGAGC
AGTGGGCGCGTACCGTCGACATGCAGGTGCACCGCGAGGTCCCTTGAAGATCCCGTCA
TCAAC

>EHL_ER_CS3544.2

GTCGGGACGCCCTCCCGGCTCTGGCATGGCGCGGTGGCTTTGCGCACGGATCGGATAA
ACTTTCCATTGGACGGCTCGTTCTCAGAAATATCTCGTAGTCTTCTCGTTGGTTACTCCA
ATGAAACGCCTCGTCTGCGGGGGCACTGAACCGCGAGAGCTATTCAAGTGCTCTACTA
CTCGACCGGGGGCAGCTTACAACCGTGGGATCGGCGCGGCCCACCTCCGGTCAACGAG
AGTTACGAGTACCTGGTCCGTTTTCCCGCGTCGGAAGCCACCAACACGATCGTAACTG
AACCAGCGCAAACGTATTGATACGCCTCGGGGGCGGGCCTCTGGCGCACCGCTGGATC
AGGCCCGTGGCGTGCCCGCCTCGTCAGCGCCACCCATTGCTAAGCGCTGACAGTAATA
GACCCCTCCATAGTAGTTGCCGATGTTGATTCGGTCACCGGCCGAAACGTATGCACTTA
GCACAGGGCAGGTACTACAAAGCGAGAGGGGGATGATTGGCAGGGGCTGCTGACGCG
CCTATCAGCCCGTTCCCCCGCGCCTGCTGTGGCGACCAACTACGCCCCGCAGCGTCCAG
TACCCGAGCAGTCTCTCAACTGGTCCGATTAGACTGTATACACCGCCGTTGGGACGCG
GACTAAACAACTCCCTCATACCCATCCGCCCGTCCGGAGCGGAACGACTCGGCGGTAC
CGGCGTCAGGCCCCCTCGCCTAGACTGCACCATTATGTTGGGAGGTGCGTCGACTGGG
GGCCGGCGACTTGATCCTGGTCGGCCCGTGAAGCCCATCAGTCCCATGGAGACGTTCT
CTCCCATCTATTGGCCTCCGGGGCTCTCCACCAATCGCACCGGAGTCTTGTCTAATATG
CAATTTATTCATCGTGAGGTATAAACCGCCCCCGAGTGGGTGCGGCTTGAAGTCCAGC
CCCCCAGGCCGCCTGCTAACACGGAAGTGTACCCGGTTCCACTCAGAGGGCATGAGGC
AGTACCTACGGATCTCAACAAGCCTAGTCCCCATGATACGTACAGCTGGGCCAGGTGG
GATTAATACGCCGGGGTCCCGTTGCGGGATGTCTAGGGGGGAGATACGTTCCGGTCAT
ATACTCTTTGTCCGCATCCCTTGGGGAAATCCATTATGCTCCCGTTGACGTCGTCAACG
TCACGCCACTAACACCGAACTAGGGGGACCAAACTATACTATGTTATCCGTATACTTAC
CCACTGTTGCAGGTCAAGGTATGGGGCTGCAAGTAATGTACATACTCCTGGTGGGAGC
TTGTCCTCCGGACTCGAATGGCTACTCGCCGGGCTGGGACTTAGCAAACCACTCTGACG
CATCTACGGCCTCAGATTAGATCACAATTTTGCTTTCTCCATTTCCTAATTACCGTTCCG
CATCGTCGGGAGGCCTTCGACTAGATTTACTCGCATGGTTTAAGCGATAATATCAAGTC
TGGAATCCTCTGACCCAACGTGAAGCGGTATGCGCAATTGGGGTCTGTTCCTTGCAAG
AGCTAATCGTAATGACGATGGACCTTCTACAAGTGAGTGCTGTAGTGCCAGCAGCGGA
CCAGATTGTTTTCCGGATGTTCCCCCTCGTGCCGAAGGCTGAACTCAGGGCGGGGCCGT
GGGCGCGCATCGTTGCCACGCAGGTACACCGTGAGGCACCTTGAAGGTCCCGTCATGA
AG

>SCWB_NCYZ0010.1

GTCGGGATGCCCTCTCGATACTGGCACGGCGCGGCGCCTTCGCATACGGATCGGATAA
GCTTTCCCGTAGATGGCTCGTCCTCGGAAATATCTCGTAGTCTTCTCGTTGGTTGCTCCA
ACGAAGCGACGAATCTACTTGGATACTGAACCGCGAGAGCTATTCAAGTGCTCTACTA
CTCGACCGGGGGCAGCATACAGCCGTGGGATCGGCGCGGCCCACCTCCGGCCAACGAG
GGGTACAACGTTTCGGCGCGTTTTCCTGCGTCAGAGGCTGCCAACACGATAGTAACCG
AACTGGCCCAAACGTATTAATACGCACAGGGGGCGGACCTCTAACGCGCCACTGGATC
AGGCCTGTGGCGTGCCCGCCTCGTCAGCACCACCCATTGCTAAGCGCTGACAGTAATA
AACCCCTCCATAGTAGTTGCCGATGTTGATTCGGTCACCGGCCGAAACGCGCGCGCTC

AGCACAAGGCAGGTACCACGGAGCGAAAGGTGGATGACGGGCAAAGGCTGTTGGCGC
GCCGATCAGCCCGTTCCCCCGCGCCTGTTGGAGCAATCACCTACGCCTCGCAACGACC
GGTACTTGAGCTGTCTCTCAACTGGTCCGATTAGATTGTATAAACAGTCGTTGAGACGC
GGACTAAACAACCTCCTCATATAAATCCGCCCGTCCGGAGCGGAACGACTTGGCGGTA
CCGGCGTCGGGCCCACTCGCCTAGATTGCACCATTATGTTGGGAGATGCGTCGACTGG
GGGCTGGCAACCTGAGGTCGGCCGTTTCGTGAAGCCCATTAGTCCCATGGAGACGTCC
TCTCCCATCCATTGGCCTCCGGGGCTCTTCATCAATCGCGTCGGAGTATCGTCTAATGT
GAAATTTATTCATCGTGAGGTATACACCGCCCCCGCGTGGGTGCGGCTCGAAGTCCGG
CCCCCCAGGCTGCCTGCTAACACGGAAGTATGCCTCGTTTAGCTCAGAAGGGACGAGA
CACTCTCGACGGCTCTCAACAAGCCTAGTCCCCACGGTACATACTACCGTGCCAGGTG
GGATTGGTACGCCGGGGTCCCGTCGCAGGATGTCTAGGGGGGAGATACGTTCCGGTCA
TATACTCTTTGTCCGCATCCCTTGGGAAAATCCATTACGCTGCCGTTGACGTCGGTAAT
GCCGCGCGATTAACACCGAGCTAATGGGACCAAAACATACCATGTAGTCCGCGCGCCT
ACCCATTGTTGCGGTTCAGGGAACGGGGGTGTGAGTAATGTACGCGCTCCTGGTGGGA
ACTTGTCCTCCGAACTTGAATAACAGCTCACTGGGATGGGATTTAGCAAACCACTCTGA
CGCATTCCAGGCCTCTGATCAGATCACAATTTTGCTTTCACCAGTTCCCAATTACGGTT
CCGCATCGTCGGGAGGCCTTCGACTGGACCTACTTCCATGGTTCGAGCGGTAATATCGG
GTCTGAAATCCCCTGACCCAACGGGAGGCGGTATGCGCGGTCGGAATCTGTTCCTTGC
AAGAGCTAATCGTAATGACGATGGACCTTCTAGAAGTGAGTTGTGTGGCGCGAGCGGC
GGATCAGATGGCCTTCCGAATGTTCCCCCTCGTGCCGAAGGCTGAACTCGAGGCGGAG
GAATGGGCGCGCACCGTTGCCACGCAGGTGCACCGCGCAGCACCTTGAGGGTCCAGTC
ATGAAG

>SCWB_NCYZ0010.2

GTCGGGATGCCCTCTCGATACTGGCACGGCGCGGCGCCTTCGCGCACGGATCGTATAA
GCTTTCCCGTAGATGGCTCGTCCTCGGAAATATCTCGTAGTCTGCTCGTTGGTTGCTCC
AACGAAGCGACGAATCTACTTGGATACTGAACCGCGAGAGCTATTCAAGTGCTCTACT
ACTCGACCGGGGGCAGCATACAGCCGTGGGATCGGCGCGGCCCACCTCCGGCCAACGA
GGGGTACGACGTTTCGGCGCGTTTTCCCGCGTCGGAGGCTGCCAACACGATAGTAACT
GAAATGGCCCAAACGTATTAATACGCACAGGGGGCGGGCAGTTGGCGCGCCACTGGAT
CAGGCCCGTGGCGTACTCGACTCGTCAGCGCCACCTATTGCTAAGCGCTGACAGTAAT
AGACCCCTCCATAGTAGTTGTTAATGTTGATTCGGTTAACGGCCGAAACGCGCGCGCTC
AGCACAAGCCAGATACCACGGAGCGAAAGGTGGATGATTGGCAGGGGCCTCTGGCGC
ACCTACAAAACTATTCCTCCGCTCCTGCTGGAGCGACTAACTACGCCCCGCAGCGACC
GGTATCCGAGCAGTCTCCTAACCGGTCCGATTCTATTGTATAAACAGTCGTTGGGACGC
GGACTAAATAACCCCCTTATACACATCCGCCCGTCCGGAGCGGAACGACTTGGTGGAA
CTGGCGTCAGGCCCCCTCGCCTAGGCTGCACCATTATGTTGGGAGGTGCGTCGACTGG
GGGCCGGCGACTTGAGCTCGGTCGGCCCGTGAAGTCCATCAGTCCCATAAAGACGTCC
TTTCCAGTCCATTGGCCTCCGGGGCTCTCCACCAATCGCACCGGAGTCTTGTCTAATGT
GAAATTTATTCATCGTGAGGTATAAACCGCCTTCGCGTGGGTGCGGCTCGAAGTTCGGC
CCCCCAGACTGCCTGCTAACACGGAAGTGTACCCGGTTCCACTCAGAGGGCATGAGGC
AGTACCTACGGATCTCAACAAGCCTAGTTCGCGCAATACACATAACCATGCCCAATGA
GATTAATACGTCGGGGTCCCATCGCGGGATGTTTGAGGGGGAGATACGTTCCGGCCAT
ATACTCCCTGTCCGCATCCCTTGGGGAAATCCATTATGCTGCCGTTGACGTCGTCAACG
TCACGCCACTAACACCGAACTAGGGGGACCAAACTATACTATGTTATCCGTATACTTAC
CCACTGTTGCAGGTCAAGGTATGGGGCTGCAAGTAATGTACATACTCCTGGTGGGAAC
TTGTCCTCCGGACTTGAATGGCTACCTGCCGGGCTGGGACTTAGCAAACCACCCTGACG
CATCCCAGGCCTTTGATCGGATCACAATTTTGATTCCACCAGTTCCCAATTACGGTTCC
GCATCGTTGGGAGGCCTTTGGCTAGATCTACCTGCATGGGTTAAGTGAGAATATCGAG
TCTGGAATCCCCCGACCCGGCGGGAGGCGGTACGTGTGATCGGAATCTGCACCTTGCG
AGAGCTAAGCGTAATGACGATGACTCTTCCACAAGTGAGTGCTGGGGCGTGAACGGGG

GACCAGATTGTCTTCCGAGCGTTCCCCCCCGTGCCGAACGATGAATTCGAGGCGGAGG
AATGGGCGCGCACCGTTGCCACGCAGGTGCACCGCGAGGTCCCTTGAAGATCCCGTCA
TCAAC

>SCWB_NCYZ0013.1

GTCGGGTTGCCTCCTCGGCTCTGGCACGGCGCGGCGCTTTCGCGCACGGATCGGATAA
ACTTTCCCGTGGGCGGCTCGTCCTCAGAAACATTTCGTAGTCTTCTCGTTGGTTACTCCA
CTGCCGCGCCGCGTCTGCTTGGATGCTGAACCACGAGATTTATTAGAGTACTCTACTAC
TCGACCGGGGGCAGCATACAACCGTGGGGTCGGCGCGGCCCACCTCCGGCCAACGAG
AGTTACGAGTACCCGGCGCGTTTTCTCACGTCGGAGACTGTTAACACGATAGTAACTG
AAATGACCCAAACGTATTAATACGCACAGGGGGCGGGCCTCTGGCGCGCCACTGGATC
AGGCCCGTGGCGTGCCCGCCTCGTCAGCGCCACCCATTGCTAAGCGCTGACAGTAATA
GACCCCTCTATAGTAGTTGCCGAAGTTGATTCGGTCACCGGCTGAAACGTATGCACTTA
GCACAGGGCAGGTACTACAAAGCGAGAGGGGGATGATTGGCAGGGGCTGCTGACGCG
CCGATCAGCCCGTTCCTCCGCGCCTGCTGGAGCGACCAACTACGCTCTGTAGCGTCCAG
TACCCGAGCAGTCTCTCAACTGGTCCGATTAGATTGTATAAACAGTCGTTGGGACGCG
GACTAAACAACCCCCTTATATACATCCGCCCGTCCGGAGCGGAACGACTTCGCGGTAC
CGGCGTCAGGCCCCCTTGCCTAGATTGCACTAATATGTTGGGAGATGCGTCGACTGGG
ATCTGGCAACCTGAGCTCGGTCGGCCCGTGAAGCCCATCAGTCCCATGGACACGTTCTC
TCCCATCCATTGGCCTCCGGGGCTCTTCATCAATCGCGTCGGAGTATCGTCTAATGTGA
AATTTATTCATCGTGAGGTATAAACCGCCCCCGCGTGGGTGCGGCTCGAAATCCGGCC
CCCTGGGCTGCTAGCTATCGCGGAAGTGTGCCTCGTTTCGCTCGGAAAAGACGAAATG
CTCTAGGCGGCTCTCAACAAGCCTATTCCCCACGATACGTACAACCGTGCCCGGTGGG
ATTAATACGCCGGGGTCCCATCGCGGGATGTTTGAGGGGGAGATACGTTCCGGCCATA
TACTCTTTGTCCGCATCCCTTGGGGAAATCCATTACGCTGCCGTTGACGTCGTTAACGT
CACGCCGCTAACACCGAACTAGGGGGACAAAACTATACCATGTTATCCGTATGTCTAC
TCGTTGTCGCGGTTCAGAGTACGGGGCTGTGAGTAATGTACGCGCTCCTGGTGGGAAC
TTGTCCTCTGAACTTGAATAACAGCTCACTGGGATGGGATTTAAAAAATCATCCCAACA
TATTCCAGGCCTCTGATCAGATCACAATTTTGCTTTCACCAGTTCCCAATTACGGTTCCG
CATCGTCGGGAGGCCTTCGACTAAATCTACTCGCAAGGTTCGAGCGGTAATATCGGGT
CTGAAATCCCCTGACCCAACGGGAGGCGGTACGTGTGATCGGAGTCTGCACCTTGCGG
GAGCTAATCGTAATGACGATGACTCTCCCACAAGTGAGTGGTGTGGCGCGGGCGGCGG
ACCAGATGGCCTTCCGAATGGTCCCCCCCGCGCCAAAGGCTGCACTCGAGGCGCGACC
GTGGGCGCGCACCGTTGCCACATAGGTGTACCGCAAGGTCCCTTGAAGGTCCCGTCAT
GAAG

>SCWB_NCYZ0013.2

GTCGGGTTGCCTCCTCGGCTCTGGCACGGCGCGGCGCTTTCGCGCACGGATCGGATAA
ACTTTCCCGTGGACGGCTCGTCCTCAGAAACATTTCGTAGTCTTCTCGTTGGTTACTCCA
CTGCCGCGCCGCGTCTGCTTGGATGCTGAACCACGAGATTTATTAGAGTACTCTACTAC
TCGACCGGGGGCAGCATACAACCGTGGGGTCGGCGCGGCCCACCTCCGGCCAACGAG
AGTTACGAGTACCCGGCGCGTTTTCTCACGTCGGAGACTGTTAACACGATAGTAACTG
AAATGACCCAAACGTATTAATACGCACAGGGGGCGGGCCTCTGGCGCGCCACTGGATC
AGGCCCGTGGCGTGCCCGCCTCGTCAGCGCCACCCATTGCTAAGCGCTGACAGTAATA
GACCCCTCTATAGTAGTTGCCGAAGTTGATTCGGTCACCGGCCGAAACGTATGCACTTA
GCACAGGGCAGGTACTACAAGGCGAGAGGGGGATGATTGGCAGGGGCTGCTGACGCG
CCTATCAGCCCGTTCCTCCGCGCCTGCTGGAGCGACCAACTACGCTCTGTAGCGTCCAG
TACCCGAGCAGTCTCTCAACTGGTCCGATTAGATTGTATAAACAGTCGTTGGGACGCG
GACTAAACAACCCCCTTATATACATCCGCCCGTCCGGAGCGGAACGACTTCGCGGTAC
CGGCGTCAGGCCCCCTTGCCTAGATTGCACTAATATGTTGGGAGATGCGTCGACTGGG

ATCTGGCAACCTGAGCTCGGTCGGCCCGTGAAGCCCATCAGTCCCATGGACACGTTCTC
TCCCATCCATTGGCCTCCGGGGCTCTTCATCAATCGCGTCGGAGTATCGTCTAATGTGA
AATTTATTCATCGTGAGGTATAAACCGCCCCCGCGTGGGTGCGGCTCGAAATCCGGCC
CCCTGGGCTGCTAGCTAATGCGGAAGTGTGCCTCGTTTCGCTCGGAAAGGACGAAATG
CTCTAGGCGGCTCTCAACAAGCCTATTCCCCACGATACGTACAACCGTGCCCGGTGGG
ATTAATACGCCGGGGTCCCATCGCGGGATGTTTGAGGGGGAGATACGTTCCGGCCATA
TACTCTTTGTCCGCATCCCTTGGGGAAATCCATTACGCTGCCGTTGACGTCGTTAACGT
CACGCCGCTAACACCGAACTAGGGGGACCAAACTATACCATGTTATCCGTATGTCTAC
TCGTTGTCGCGGTTCAGAGTACGGGGCTGTGAGTAATGTACGCGCTCCTGGTGGGAAC
TTGTCCTCTGAACTTGAATAACAGCTCACTGGGATGGGATTTCGCAAACCATCCCAACA
TATTCCAGGCCTCTGATCAGATCACAATTTTGCTTTCACCAGTTCCCAATTACGGTTCCG
CATCGTCGGGAGGCCTTCGACTAAATCTACTCGCAAGGTTCGAGCGGTAATATCGGGT
CTGAAATCCCCTGACCCAACGGGAGGCGGTACGTGTGATCGGAGTCTGCACCTTGCGG
GAGCTAATCGTAATGACGATGACTCTCCCACAAGTGAGTGGTGTGGCGCGGGCGGCGG
ACCAGATGGCCTTCCGAATGGTCCCCCCCGCGCCAAAGGCTGACCTCGAGGCGCGACC
GTGGGCGCGCACCGTTGCCACATAGGTGTACCGCAAGGTCCCTTGAAGGTCCCGTCAT
GAAG

>SCWB_SYWB103.1

TTCGGGATGCCCTTTCGACTGTGCCGCGGCGCGGCGCCTCCGCGCACGGATCAGATAA
GCTTTCCCGTGGACGGCTCGTCCTCAGAAACATTTCGTAGTCTTCTCGTTGGTTACTCCA
CTGCCGCGCCGCGTCTGCTTGGATGCTGAACCACGAGATTTATTAAAGTACTCTACTAC
TCGACCGGGGGCAGCATACAACCGTGGGATCGGTGCGACCCTCCTCCGGCCAGCAGGG
AGTACGACGTCCCGGCCTGTTTTCCTGCGTCAGAGGCTGCCAACACGATAGTAACTGA
AATGGCCAAAACGTACTAATACGCCCCGGTTGCGGGCCTCTGGCGCGCCACTAAACTG
AGTCCAGGATGTGCCCGCCTCGCCATCGTCATTCATCGCTGTATATTAGAAAAATCAGA
TTCTCTCATAGTAGTTGTCGATGTTGATTCGGTCGCCGGCCGAAACGTATGCACTTAGC
ACAGGGCAGGTACTACAAAGCGAGAGGGGGATGATTGGCAGGGGCTGCTGACGCGCC
TACCAACTTGTTCCTCCGCGCTTGCTGGAGCGACCAACTACGCCCCGCAGCAACCGGTA
CTTGAGCTGTCTCCCAACTGGTCCGGGGAGACTGTATACATCCTTGTTGGGTCGTTGCA
TAAACAACCCACTCTTACCCATCCGCCCGTCCGGAGCAAAACAACTTGGTAGTACCGG
CGTCAGGCCCCCTCGCCTAGGCTGCACCATTATGTTGGGAGGTGCGTCGATTGGGGGTT
GGCGACTTGAGCTCGGTCGGCCCGTGAAGTCCCTCAGGCCCATGGAGACGTTCTCTCCC
ATCCATGGGTCTCCTGGGCTTTCCACGAATTGCGTCGAAGTAATGCCAGATGTTAAGCG
TATTCATCATAGGGCACAAGCCGCGCCTTCGTGGGTGCGGCTCGAAGCCCGACCCCCC
AGGCTGCCTAATGACACGGAAGCGTACCTGGTTCCACTCAAAAGGCATGAGGCAGTCC
CTACAGATCTTAGCAAGCTAAGTCCCCACGATACATACAACCGGGCCAGGTGGGATTA
AGACGCCGGGGTCCCGTCGCGGGATGTTTGAGGGGGAGATACGTTCCGGCCATATACT
CTTTGTCCGCATCCCTTGGGGAAATCCATTACGCTGTCGTTGACGTCGTTAACGTCACG
CCACTAACACCGAACTAGGGGGACCAAACTATACCATGTTATCCGTATGCCTACTCGTT
GTCACGATTCAGGGTACGGGGCTGTGAGTAATGTACACACTCCTGGTGGGAACTGGTC
CTCCGGACTTGAATGGTTACTCGCCGAGCTGGAACTTAGCAAACCACCCTGATGCATTC
CAGGTATCAGATTAGAACACAATTTTGCTTTCACCAGTTCCCAATTACGGTGCCGTATC
GTTGGGAGGCCTTTGGCTAGATTTACTCCCATGGTTCGAGCGGTAATATCGGGTCTGAA
ATCCCCTGACCCAACGGGAGGCGGTATGTGTGGTCGCGATCTGCACCTCGCGAGAGCC
GAGCGTAATGACGATGACTCTCCCACAAGTGAGTGGTGTGGCGCGGGCGGCGGACCAG
ATGGCCTTCCGAATGGTCCCCCCCGCGCCAAAGGCTGACCTCGAGGCGCGACCGGGGG
CGCGCACCGTTGCCACATAGGTGTACCGCAAGGTCCCTTGAAGGTCCCGTCGTGAAG

>SCWB_SYWB103.2

GTCGGGATGCCCTCCCGACACCAGCACGGCGCGGCGCTCTCGCGCATGTACCAGATGG
GCTTTCCCGTGGATGGCTCGTCCTCAGAAATATCTCGTAGTCTTCTCGTTGGTTGCTCCA
ACGAAGCGACGAATCTACTTGGATACTGACCCGCGAGAGCTATTCAAGTGCTCTACTA
CTCGACCGGGGGCAGCTTACAACCGTGGGATCGACGCGGCTCATCTCCGGCCAACGAA
AGTTACGACGTCCCGGCCCGTTTTCCCGCGTCGGAGGCTGCCAATACGATAGTCACTGA
AATGGCCAAAACGTACTAATACGCCCCGGTTGCGGGCCTCTGGCGCGCCACTGGATCA
GGCCCGTGGCGTGCCCGCCTCGTCAGCACCACCCATTGCTAAGCGCTGACAGTAATAG
ACCCCTCCATAGTAGTTGCCGATGTTGATTCGGTCACCAGCCGAAACGCGCGCGCTCA
GCACAAGGCAGGTACCACGGAGCGAAAGGTGGATGACGGGCAAAGGCTGTTGGCACG
CCTACAAAACTATTTCTCCGCTCCTGCTGGAGCGACCAACTACGGTTTGTAGCGTCCAG
CACCCGAGCAAGCTCTCAACTGATCCGATGAGACGTTATACACCGCCGGTGGGATACG
GACTAAACAACCCCCTCATACCCATTCGCCCGACCGGAGCGGAACGACTCCGCGATAC
CGACGTCAGGGCCCCTCGACTAGACTGCGCCGTTATATTGGGCGGTGCGTCGACTGAG
GGCTAGCGACTTGATCCCGGTCGGCCCGTGAAGCCCATCAGTTCCATGGAGACGTCCG
TTCCAGTCCATTGGCTTCCGGGGCTCTCCACCAATCGCACCGGAGTCTTGTCTAATGTG
AAATTTATTCATCGTGAGGTATAAACCGCCCCCGCGTGGGTGCGGCTCGAAGTCTGGC
CTCCCAGGCTGCCTGCTAACACGGAAGTGTACCCGGTTCCACTCAGAGGGCATGAGGC
AGTACCTACGGATCTCAACAAGCCTAGTTCGCGCAATACACATAACCATGCCCAAAGA
GATTAAGACGCCGGGGTCTCATCGCGGGATGTTTGAGGGGGAGATACGTTCCGGCCAT
ATACTCCCGGTCCGCGCCCCTCGGAGAAATCCATTACACTGCCGTTGACGTCGTTAACG
TCACGCCACTAACACCGAACTAGGGGGACCAAACTATACCATGTTATCCGTATGCCCA
CTTGTTGTTGCAGGTCAAGGTACGGGGCTGCAAGTAATGTACACACTCTTGGTGGGAG
CTTATCCTCCGGACTTGAATGGCTACTCGCCGGGCTGGGACTTAGCAAACCACCCTGAC
GCATTCCAGGCCTCTGATCAGATCACAATTTTGCTTTCACCAGTTCCCAATTACGGTTC
CGCATCGTCGGGAGGCCTTCGACTAGATCTACTCGCATGGTTCGAGCGGTAATATCGG
GTCTGAAATCCCCTGACCCAACGGGAGGCGGTACGTGTGGTCGGAATCTGCACTTTGT
GGGAGCTAATCGTAATGACGATGGCTCTTCCACAAGTGAGTGGTGTGGCGCGAGCGGC
GGACCAGATTGTCTTCCGAATGTTCACCCCCGTGCCGAAGGCTGAACTCAAGGCGGGG
CCGTGGGTGCGCACCGTTACCACGCAGGTGCATCGCGAGGCACCTTGAAGGTCCCGTC
ATGAAG

>SCWB_SYWB205.1

GGCGGGATGCCTTCTCGACTCCGGGACGGCGTGGCGCCTTCGTGCACGGATGGGATAA
GTTTTCCCGTGGACGGCTCGTCCTCAGAAATATCTCGTAGTCTTCTCGTTGGTTACTCCA
ATGAAGTTCCTCGTCTGCGGGGATGCTGACCCGCGAGAGCCATTCGAGTGCTCTACTAC
TCGACCGGGGGCAGCACGCAGCCGCGGGAACGGCGCGGTCTACCTCCGGCCAGCAGG
GGGGACGACGTCCCGGCCCGTTTTCCCGCGTCGGAGGCTGCCAACACGATAGTAACTG
ACCCAGCGCAAACGTATTGATACGCACAGGGGGCGGGCCTCTGGGGCGCCACTGGATC
AGGCCTGTGGCGTGCCCGCCTCGTCAGCACCACCCATTGCTAAGCGCTGACAGTAATA
AACCACTCCATAGTAGTGGCCGATGTTGATTCGGTCACCGGCCGAAACGTATGCGCTC
AGCACAGGGCAGGTACTACGGAGCGAAAGGTGGATGATTGGCAGGGGCCGCTGGCGC
ACCTACAAAACTATTCGTCCGCGCCTGCTGGAGCGACCAACTACGCTCTATAGCGTCCA
GTACCCGAGCAGTCTCCCAACCGGTCCGGTGAGACTGTATAAACAGTCGTTGAGACGC
GGACTAAACAACCTCCTCATACCAATCCGCCCGTCCGGAGCGGACCGACTCGGCGGTA
CCGGCGTCAGGCCCCCTCGCCTAGACTGCACCATTATGTTGGGAGGTGCGTCGACTGG
GGGCCGGCGACTTGATCCTGGTCGGCCCGTGAGGCCCATCAGGCCTATGGAGACGGTC
TCTCCCATCCATTGGCCCCCGGGGTCCTCTACCGATTGCGTCGGAGTCTTGTATAATAT
GAAATTTATTCATCGTGAGGCATAAACCGCCCCCGCGTGGGTGCGGCTCGAAGCCCGG
CTTCCCAGGTTGGCAGCTAACACGGAAGTGTGCCTCGTTTCGCTCAGGAGGGACGGGA
CACTCTCGACGGCTCTCAACAAGCCTAGTCCCCACGATACATACAACCGTGCCCGGTG
GAATTAAGATGCCTGGAGCCCGCCGCGGGATGTCTAGGGGGGAGATACGTTCCGGTCA

TATACTCTTTGTCCGCATCCCTTGGGAAAATCCATTACGCTGTCATTGACGTCGTCAAC
GTCATGCCACTAACACCGAACTAGGGGGACCAAACTATACCATGTTATCCGTATGCCT
ACTCGTTGTCGCGGTTCAGAGTACGGGCCTGTGAGTAATGTACGCGCTCCGGGTGGGA
ACGTGTCCTCCGGACTTGAATGGTTACTCGCCGAGCTGGAACTTAGCAAACCACCCTG
ATGCATTCCAGGTATCAGATTAGAACACAATTTTGCTTTCACCAGTTCCCAATTGCGGG
GCCGTATCGTCGGGAGGCCTTCGACCGGGTCTACTCGCATAGTTCGAGCGGTAATATTG
GATCTGAAATCTCCTGACCCAACGGGGGGCGGTACGTGTGATCGGAATCTGCACCTTG
CGAGATCTAATCGTAATGACGATGGCTCTTCCACAAGTGAGTGCTGGGGCACGAGCGG
CGGACCAGATGGCCTTCCGAATGTTCCCCCCCGTGCCGAAGGCTTAACTCGAGGCGGA
GCCGTGGGCTCGCACCGTTGCCGCGCAGGTACACCGCGAGGTCCCTTGAAGGTCCCAT
CATGAAG

>SCWB_SYWB205.2

GTCGGGATGCCCTCTCGATACTGGCACGGCGCGGCGCCTTCGCGCACGGATCGGATAA
GCTTTCCCGTAGATGGCTCGTCCTCGGAAATATCTCGTAGTCTGCTCGTTGGTTGCTCC
AACGAAGCGACGAATCTACTTGGATACTGAACCGCGAGAGCTATTCAAGTGCTCTACT
ACTCGACCGGGGGCAGCATACAGCCGTGGGATCGGCGCGGCCCACCTCCGGCCAACGA
GGGGTACAACGTTTCGGCGCGTTTTCCTGCGTCAGAGGCTGCCAACACGATAGTAACC
GAACTGGCCCAAACGTATTAATACGCCCAGGGGGCGGGCCTCTGGCGCGCCACTGGAT
CAGGCCCGTGGCGTGCCCGCCTCGTCAGCGCCACCCATTGCTAAGCGCTGACAGTAAT
AGACCCCTCCATCGTAGTTGCCGATGTTGATTCGGTCACCGGCCGAAACGTGCGCGCTC
AGCACAAGGCAGGTACCACGGAGCGAAAGGTGGATGACGGGCAAAGGCTGTTGGCGC
GCCGATCAGCCCGTTCCCCCGCGCCTGTTGGAGCGACCAACTACGCCCCGCAGCGACC
GGTACCCGAGCAGTCTCTCAACTGGTCCGAGTAGATTGTAAACATCCTTGTTGGGTCGT
TGCATAAACAACCCACTCTTATACATCCGCCCGTCCGGAGCGGAACGACTTGGCGGTA
CCGGCGTCAGGCCCACTCGCCTAGACTGCACCATTATGTTGGGAGATGCGTCGACTGG
GGGCCGGCAACCTGAGGTCGGCCGTTTCGTGAAGCCCATTAGTCCCATAAAGACGTCC
TCTCCAATCCATTGGCCTCCGGGGCTCTCCACCAATCGCACCGGAGTCTTGTCTAATGT
GAAATTTATTCATCGTGAGGTATAAACCGCCTTCGCGTGGGTGCGGCTCGAAGTTCGGC
CCCCCAGACTGCCTGCTAACACGGAAGTGTACCCGGTTCCACTCAGAGGGCATGAGGC
AGTACCTACGGATCTCAACAAGCCTAGTTCGCACAATACACATAACCATGCCAAATGA
GATTAATACGTCGGGGTCCCGTCGCGGGATGTTTGAGGGGGAGATACGTTCCGGCCAT
ATACTCCCTGTTCGCGCCCCTCAGAGAAGTCCATTACGCTGCCGTTGACGTCGTCAATG
CCGCGCCACTAACACCGGACTAGGGGGACCAAACCATACCATGTAATCCGTGTACTTG
CCCATTGTCGCGGTTCAGAGTACTGGGCTGTGAGTAATGTACGCGTTCCTGGTGGGAAC
TTGTCCTCTGAACTTGAATAACAACTCACTGGAATGGGATTTAGCGAACCATCCCAACA
TATTCCAGGCCTCTGATCAGATCACAATTTTGCTTTCACCAGTTCTCAATTATGATTCCG
CATCGTCGGGAGGCCTTCGACTAGATCTGCTCGCATGGTTCGAGCGGTAATATCGGGTC
TGAAATCCCCTGACCCAACGGGAGGCGGTATGCGTGATCGGAATCTGCACCTTGCGAG
AGCTAATCGTGATGACGATGGCTCTTCCACAAGTGAGTGCGGGGGCGCGAGCGGCGAA
CCAGATGGTCCTCTGAATGTTCCCCCCCGTGCCGAAGGCTGAACTCGAGGCGGGGCCG
TGGTCGCGCACCGTTACCACGCAGGTGCGTCGCGAGGCCCCTTGAAGGTCCCGTCGTG
AAG

>SCWB_ZJWB01.1

GTCGGGACGCCCTCCCGGCTCTGGCATGGCGCGGTGGCTTTGCGCACGGATCGGATAA
ACTTTCCATTGGACGGCTCGTTCTCAGAAATATCTCGTAGTCTTCTCGTTGGTTACTCCA
ATGAAACGCCTCGTCTGCGGGGGCACTGAACCGCGAGAGCTATTCAAGTGCTCTACTA
CTCGACCGGGGGCAGCTTACAACCGTGGGATCGGCGCGGCCCACCTCCGGTCAACGAG
AGTTACGAGTACCCGGTCCGTTTTCCCGCGTCGGAGGCTACCAACACGATAGTAACTG

AACCAGCGCGAGAACATTGATACGCCTCGGGGGCGGGCCTCTGGCGCGCCACTGGATC
AGGCCCGTAGCGTGCCCGCCTAGTCAGCGCCACCCATTGCTAAGCGCTGACAGTAATA
GACCCCTCCATAGTAGTTGTCGATGTTGATTCGGTCACCAGCCGAAACGTGCGCGCTCA
GCGCAGGGCAGGTACTGTAGAACGAAAGGTGGATATTTGGCAAAGGCTGCTGGCGCG
CCTACCAACCTGTTCCTCCGCGCCTGCTGGAGCGACCAACTACGCCCCGCAGCGACCG
GTACTTGAGCTGTCCCTCAACTGGTCCGATGAGACTGTATACACCGCCGTTGGGACGCG
GACTAAACAACCCCCTCATACCCATCTGCCCGTCTGCAGCAGAACGACTCGACGGTAC
CGGCGTCAGGCGCCTCCGCCTAGGCTGCATCATTATGTTGGGAGATGCGTCGACTGGG
GGCCGGCGACCTGAGCTCGGTCGTCCCGTGAAGCCACTCAGGCTCATGGAGCCGTTCT
CTCCCATCCATTGGCCTCCTGGGCTTTCCACCAATTGCGTCGAAGTATTTTCTAATGTTA
AGTGTGTTTGTCGCGGGACACAAACTGCCCCCGCGCGGGTGCGGCGCGTAATCCGGCC
CCCCAGGCCGCCTGCTAACACGGAAGTGTACCCGGTTCCACTCAGAGGGCATGAGGCA
GTACCTACGGATCTCAACAAGCCTAGTCCCCACGATACGTACAGCTGGGCCAGGTGGG
ACTAATACGCCGGGGTCCCGTCGCGGGACATCTGGGATGGGAACACGTTCCGGTCAGA
TACTCTTTGTCCGCATCCCTTGGGGAAATCCATTACGCTGTCATTGACGTCGTCAACGT
CATGCCACTAACACCGAACTAGGGGGACCAAACTATACCATGTTATCCGTATGCCTAC
TCGTTGTCGCGGTTCAGAGTACGGGCCTGTGAGTAATGTACGCGCTCCTGGTGGGAACT
TGTCCTCCGGACTTGAATGGTTACTCGCCGAGCTGGAACTTAGCAAACCACCCTGATGC
ATTCCAGGTATCAGATTAGAACACAATTTTGCTTTCACCAGTTCCCAATTGCGGGGCCG
TATCGTCGGGAGGCCTTCGACCGGGTCTACTCGCATAGTTCGAGCGGTAATATTGGATC
TGAAATCTCCTGACCCAACGGGAGACGGTGTGCTCGACCGGGATCTGCACCTTGCGAG
AGCTAATCGTAATGACGATGGCTCTTCCACAAGTGCGTGCTGGGGCGCGAACGGCGGA
TCATATGGTCTTCCGAATGTTCCCCCCCGTGCCGAAGGCTGCACTCGAGGCGGAGCAGT
GGGCGCGTACCGTCGACATGCAGGTGCACCGCGAGGTCCCTTGAAGATCCCGTCATGA
AG

>SCWB_ZJWB01.2

GTCGGGATGCCCTCCCGGCTCTGGCACCGCGCGGCGCCTTCGCATGCGTATCGGATAA
GCTTTCCCGTGGACCAATCGTCCTCAGAAATATCTCGTAGTCTTCTCGTTGGTTATTCCA
ATGAAGCGCCTCGTCTGCGGGGGCACTGAACCGCTAGAGCTATTCGAGTGCTCTACTA
CTCCACCGGGGGCAGCACGCAGCCGTGGGATCGGCACGGCCCACCTCCAGCCAACGAG
AGTTACGAGTACCCGACGCGTTTTCCCGCGTCGGAGGCTGCCAACACGATAGTAACTA
CACTGGCCCAAACGTATTAATACGCACAGGGGGCGGGCATTTGGCGCGCCACTGGATC
AGGCCCGTGGCGTACTCGACTCGTCAGCGCCACCTATTGCTAAGCGCTGACAGTAATA
GACCCCTCCATAGTAGTTGCTAATGTTGATTCGGTTGACGGCCGAAACGCGCGCGCTCA
GCACAGGCCAGGTACCACGGAGCGAAAGGTGGATGATTGGCAGGGGCCTCTGGCGCA
CCTACAAAACTATTTCTCCGCTCCTGCTGGAGCAACCAACTACGCCCCGCAGCGACTGG
TATCCGAACAGTCTCTCAACTTGGCCGATTCTATTGTATAAACAGTCGTTGGGACGCGG
ACTAAACAACCTCCTCATACCCATCCTCCCGTTCGCAGCAGAACGACTCGACGGTACC
GGCGGCAGGCCCCTCCGCCTAGGCTGCATCATTATGTTGGGAGATGCGTCGACTGGGG
GCCGGCGACCTGAGCTCGGTCGTCCCGTGAAGCCAATCAGTCTCATGGAGCCGTTCTCT
CCCATCCATTGGCCTCCTGGGCTTTCCACCAATTGCGTCGAAGTATTTTCTAATGTTAA
GTGTGTTTGTCGCGGGACACAAACTGCCCCCGCGCGGGTGCGGAGCGTAATCCGGCCC
CCCAGGCCGCCTGCTAACACGGAAGTGTACCCGGTTCCACTCAGAGGGCATGAGGCAG
TACCTACGGATCTCAACAAGCCTAGTCCCCATGATACGTACAGCTGGGCCAGGTGGGA
TTAATACGCCGGGGTCCCGTCGCGGGATGTTTGAGGGGGAGATACGTTCCGGCCATAT
ACCCCCTGTCCGCATCCCGTGGGGAAATCCATTACGCTGCTGTTGCCGTCGTCAACGTC
ACGCCGCTAACACCGAACTAGGGGGACCAAAACATACCATGTAATCCGTGTACTTGCC
CATTGTCGCGGTTCAGAGTACGGGGCTGTGAGTAATGTACGCGTTCCTGGTGGGAACTT
GTCCTCTGAACTTGAATAACAGCTCACTGGAATGGGATTTAGCAAACCATCCCAACAT
ATTCCAGGCCTCTGATCAGATCACAATTTTGCTTTCACCAGTTCCCAATTATGATTCCGC

ATCGTTGGGAGGCCTTCGACTAGATCTGCTCGCATGGTTCGAGCGGGAATATCGGGTCT
GAAAACCCCTGACCCAACGGGAGGCGGCATATGTGATCGCAATCTGCACCTTGCGAGA
GCTGATCGTATTCATGATGACTCCTTCACAAGTGAGTGGTGTGACGCGAGCAGCGGAC
CAGATGATTTTCCGAATGTTCCCCTCCGTACCGAAGGCTTAACTTGAGGCGGAGCCGTG
GGCTCGCACTCCTGCTACGCAGGTGCACCGCGAGGCAACTTGAAGGTCTCGTCATGAA
G

>SCWB_ZJWB02.1

GTCGGGATGCCCTCCCGGCTCTGGCACCGCGCGGCGCCTTCGCATGCGTATCGGATAA
GCTTTCCCGTGGACCAATCGTCCTCAGAAATATCTCGTAGTCTTCTCGTTGGTTATTCCA
ATGAAGCGCCTCGTCTGTGGGGGCACTGAACCGCTAGAGCTAGTCGAGTGCTCTACTA
CTCCACCGGGGGCAGCACGCAGCCGTGGGATCGGCACGGCCCACCTCCAGCCAACGAG
AGTTACGAGTACCCGACGCGTTTTCCCGCGTCGGAGGCTGCCAACACGATAGTCACTG
AACTGGCCCAAACGTATTAATACGCACAGGGGGCGGGCATTTGGCGCGCCACTGGATC
AGGCCCGTGGCGTACTCGACTCGTCAGCGCCACCTATTGCTAAGCGCTGACAGTAATA
GACCCCTCCATAGTAGTTGCTAATGTTGATTCGGTTAACGGCCGAAACGCGCGCGCTCA
GCACAGGCCAGGTACCACGGAGCGAAAGGTGGATGATTGGCAGGGGCCTCTGGCGCA
CCTACAAAACTATTTCTCCGCTCCTGCTGGAGCAACCAACTACGCCCCGCAGCGACTGG
TATCCGAACAGTCTCTCAACTTGGCCGATTCTATTGTATAAACAGTCGTTGGGACGCGG
ACTAAACAACCTCCTCATACCCATCCTCCCGTTCGCAGCAGAACGACTCGACGGTACC
GGCGTCAGGCGCCTCCGCCTAGGCTGCATCATTATGTTGGGAGATGCGTCGACTGGGG
GCCGGCGACCTGAGCTCGGTCGTCCCGTGAAGCCACTCAGTCTCATGGAGCCGTTCTCT
CCCATACATTGGCCTCCTGGGCTTTCCACCAAGTGCGTCGAAGTATTTTCTAATGTTAA
GTGTGTTTGTCGCGGGACACAAACTGCCCCCGCGCGGGTGCGGAGCGTAATCCGGCCC
CCCAGGCCGCCTGCTAACACGGAAGTGTACCCGGTTCCACTCAGAGGGCATGAGGCAG
TACCTACGGATCTCAACAAGCCTAGTCCCCATGATACGTACAGCTGGGCCAGGTGGGA
TTAATACGCCGGGGTCCCGTCGCGGGATGTTTGAGGGGGAGATACGTTCCGGCCATAG
ACCCCCTGTCCGCATCCCTTGGGGAAATCCAGTACGCTGCTGTTGCCGTCATCAACGTC
ACGCCGCTAACACCGAACTAGGGGGACCAAAACATACCATGTAATCCGTGTACTTGCC
CATTGTCGCGGTTCAGAGTACGGGGCTGTGAGTAATGTACGCGTTCCGGGTGGGAACT
TGTCCTCTGAACTTGAATAACAGCTCACTGGAATGGGATTTAGCAAACCATCCCAACAT
ATTCCAGGCCTCTGATCAGATCACAATTTTGCTTTCACCAGTTCCCAATTATGATTCCGC
ATCGTCGGGAGGCCTTCGACTAGATCTGCTCGCATGGTTCGAGCGGGAATATCGGGGC
TGAAAACCCCTGACCCAACGGGAGGCGGCATATGTGATCGCAATCTGCACCTTGCGAG
AGCTGATCGTATTCATGATGACTCCTTCACAAGTGAGTGGTGTGACGCGAGCAGCGGA
CCAGATGATTTTCCGAATGTTCCCCTCCGTACCGAAGGCTTACCTTGAGGCGGAGCCGT
GGGCTCGCACTCCTGCTACGCAGGTGCACCGCGAGGCAACTTGAAGGTCTCGTCATGA
AG

>SCWB_ZJWB02.2

GTCGGGATGCCTTCCCAACTCCGGCACGGCGCGGCGCCTTCGCGCACGGATCGGATAA
GCTTACCCGTGGACGGCTCGACCCCGGAGATATCTCGTATTCTTCTTGTTGGTTACTCT
GATGCCGCGCCGCGTCTGCTTGGATGCTGAACCGCGAGAGCCATTCGAGTGCTCTACT
ACTCGACCGGGGGCAGCACGCAGCCGCGGGAACGGCGCGGTCTACCTCCGGCCAGCA
GGGGGGACGACGTCCCGGCCCGTTTTCCCGCGTCGGAGGCTGCCCACACGATAGTAAC
TGAACTGGCCCAAACGTATTAATACGCACAGGGGGCGGGCCTCTGGCGCGCCACTGGA
TCAGGCCCGTGGCGTGCCCGCCTCGTCAGCGCCACCTATTGCAAAGCGCTGACAGTAA
TAGACCCCTCCATAGTAGTTGCCGATTTTGATTCGGTCACCGGCTGAAACGTATGCACT
TAGCACAGGGCAGGTACTACAAAGCGAGAGGGGGATGATTGGCAGGGGCTGCTGACG
CGCCTATCAGCCCGTTCCCCCGCGCCTGCTGTGGCGATCGCCTACGCCCCGCAGTGACC

GGTACCCGAGCAGTCTCCCATCTGGTCTGATGAGACTGTATACACCGCCGTTGGGATGC
GGACTAAACAACCCCCTCATACCCATCCGCTTGTCCGCAGCAGAACGACTCGGCGGTA
CTGGCGTCAGGCCCCCTCGCCTAGACTGCACCATTATGTTGGGAGGTGCGTCGACTGG
GGGCTGGCAACCTGAGGTCGGCCGTTTCGTGAAGCCCATTAGTCCCATGGAGACGTTC
TCTCCCATCCATTGGCCTCCGGGGCTCTTCATCAATCGCGTCGGAGTATCGTCTAATGT
GAAATTTATTCATCGTGAGGTATACACCGCCCCCGCGTGGGTGCGGCTCGAAGCCCGG
CTTCCCAGGCTGGCAGCTAACACGGAAGTGTGCCTCGTTCCACTCAGAGGGCATGAGG
CAGTACCTACGGATCTCAACAAGCCTAGTCCCCATGATACGTTCAGCTGGGCCAGGTG
GGATTAATACGCCGGGGTCCCGTCGCGGGATGTTTGAGGGGGAGATCCGTTCCGGCCA
TATACTCTCTGTCCGCATCCCTTGGGGAAAGCCATTACGCTGCCGTTGACGTCGTTAAC
GCCGCTTCATTAACGCCGAACTAGGGAGACCAAACTACATCATGTTATCTGTATGCCCA
GTTGTTGTTGCGGTTCAGGGTACGGGGCTGTGAGGCACGGGCGCGCTCCTGGTGGGAA
CTTGTCCTCCGGACTTTAATGGCTACTCGCCGGGCTGAGACTTAGCAGGCCACCCTGAC
GCATTCCAGGCCTCAGATTAGATCACAATTTTGCTTTCATTAGTTCCCAATTACGGTTCC
GCATCGTCAGGAGGCCTTCGACTAGATCTACTCGTATGGTTCGAGCGGTGATATCGGGT
CTAAAATCCCCTGACCCAATGGGGGGCGGTACGTGTGATCGGAATCTGCACCTTGCGA
GATCTAATCGTAATGACGATGGCTCTTCCACAAGTGAGTGCTGGGGCGCGAGCGGCGG
ACCAGATGGCCTTCCGAATGTTCCCCCCCGTGCCGAAGGCTTAACTCGAGGCGGAGCC
GTGGGCTCGCACCGTTGCCGCGCAGGTACACCGCGAGGTCCCTTGAAGGTCCCATCAT
GAAG

>WZS_WZS149.1

GTTGGGATCCCTTCTCGACTCTGGCACGGCGCGGTGGCTTCGCGCGCGTATCAGCCAAG
CTTTCCCGTGGACGGCTCGTTCTCAGAAATATCTCGTAGTCTTCTCGTTGGTTACTCCAA
TGAAACGCCTCGTCTGCGGGGGCACTGAACCGCGAGAGCTATTCAAGTGCTCTACTAC
TCGACCGGAGGCAGCTTACAACCGTGGGATCGGCGCGGCCCACCTCCGGTCAACGAGA
GTTACGAGTACCTGGTCCGTTTTCCCGCGTCGGAAGCTACCAACACGATAGTAACTGA
ACCAGCGCGAGAGCATTGATACGCCTCGGGGGCGGGCCTCTGGCGCGCCACTGGATCA
GGCCCGTGGCGTGCCCGCCTCGTCAGCGCCACCCATTGCTAAGCGCTGACAGTAATAG
ACCCCTCCATAGTAGTTGCCGATGTTGATTTGGTCACCGGCCGAAACGTATGCGCTCAG
CACAGGGCAGGTACTACGGAGCGAAAGGTGGATGATTGGCAGGGGCCGCTGGCGCAC
CTACAAAACTATTCGTCCGCGCCTGCTGGAGCGACCAACTACGCTCTATAGCGTCCAGT
ACCCGAGCAGTCCCTCAATTGGTCCGATGAGACTGTATACGCCGCCGTTGGGACGCGG
ACTAAACAACCCCCTCATACCCATTCGCCCGTCCAGAGCGGAACGACTCCGCGGTGCC
GGCGTCAGGCCTCCTCGCCTAGGCTGCGCCATTATGTTGGGAGGTGCGTTGACTGGGG
GCCGGCGACCTGAGCTTGGTCGGCCCGTGAAGCCCATCAGTCTCATGGAGACGTTCTCT
CCCATCCAGTGGCCTCCTAGGCTTTCCACCAATTGCGTCAGGGTAATGCCAGATGTTAG
ATGTATTCATCGTGAGGTATACACCGCCCCCGCGTGGGTGCGGCTCGAAGCCCGGCCT
CCCAGGCTGGCTGCTAACACGGAAGTGTACCCGGTTCCACTCAGAGGGCATGAGGCAG
TACCTACGGATATCAACAAGCCTAGTCCCCATGATACGTACAGCTGGGCCAGGTGGGA
TCAATACGTCGGAAGCCCGTCGCGGGATGTCCAGGGGGGAGATACGTTCCGGCCATAT
ACTCTCTGTCCACATCCCTTGAGGAAATCCATTACGCTGCCGTTGACGTCGTTAACGCC
ACGCCACTAACACCGAACTAGGGGGACCAAACTATACCATGTTATCCGTATGCCCAGT
TGTTGTTGCGGTTCAGGGTACGGGGCTGTGAGGCACGGGCGCGCTCCTGGTGGGAACT
GGTCCTCCGGACTTTAATGGCTACTCGCCGGGCTGAGACTTAGCAGACCACCCTGACG
CATTCCAGGCCTCTGATCAGATCACAATTTTGCTGTCACCAGTTTCCAATTACGGTTCC
GCATCGCCGGGAGGCCTGCGATTAGATCTACTCGCATGGGTCGAGCGGTAATATCGGG
TTTGAAATCCCCTGACCCAACGGGGGACGGTATGCGCAATCAGGATCTGCACCTTTCG
GGAGCTAATCATAATGACGATGGCTCTTCCACAAGTGAGTGGTGTAGCGCCAGCGGCG
GACCAGACGGTCTTCCGAATGTTCCCCCCCGTGCCGAAGGCTGAACTCAAGGCGGGGC

CGTGGGCGTGCCCCGTTGCCACGCAGGTGCACCGCGAGGCACCTTGAAGGTCCCGTCA
TGAAG

>WZS_WZS149.2

GTCGGGATGCCTTCCCAACTCTGGCACGGCGCGGCGCCTTCGCGCACGGATCAGATAA
GCTTTCCCGTGGACGGCTCGACCCCGGAGATATCTCATATTCTTCTTGTTGGTTACTCCA
ATGAAGCGCCTCGTCTGCGGGGGCACTGAACCGCGAGAGCTATTCAAGTGCTCTACTA
TTCCACCGGGGGCAGCACGCAGCCGTGGGATCGGTGCGACCCACCTCCGGCCAACGAG
AGTTACGAGTACCCGGCCCGTTTCCCCGCATCGGAGGCTGCCAACACGATAGTAACTG
AACTGGCCCAAACGTATTAACACGCACAGGGGGCGGGCCTCTGGCGCGCCACTGGATC
AGGCCCGTGGCGTGCCCGCCTCGTCAGCGCCACCCATTGCTAAGCGCTGACAGTAATA
GACCACTCCATATAAGTTGCCGATGTTGATTCGGTCACCGGCCGAAACGTATGCACTTA
GCACAGGGCAGGTACTACAAAGCGAGAGGGGGATGATTGGCAGGGGCTGCTGACGCG
CCTATCAGCCCGTTCCCCCGCGCCTGCTGTGGCGACCAACTACGCCCCGCAGCGTCCAG
TACCCGAGCAGTCTCTCAACTGGTCCGATTAGACTGTATACACCGCCGTTGGGACGCG
GACTAAACAACTCCCTCATACCCATCCGCCCGTCCGGAGCGGAACGACTCGGCGGTAC
CGGCGTCAGGCCCCCTCGCCTAGACGGCACCATTATGTTGGGAGGTGCGTCGACTGGG
GGCCGGCGACTTGATCCTGGTCGGCCCGTGAAGCCCATCAGTCCCATGGAGACGTTCT
CTCCCATCTATTGGCCTCCGGGGCTCTCCACCAATCGCACCGGAGTCTTGTCTAATATG
AAATTTATTCATCGTGAGGTATAAACCGCCCCCGAGTGGGTGCGGCTTGAAGTCCAGC
CCCCCAGGCCGCCTGCTAACACGGATGTGTACCCGGTTCCACTCAGAGGGCATGAGGC
AGTACCTACGGATCTCGACAAGCCTAGTCCCCATGATACGTACAGCTGGGCCAGGTGG
GATTAATACGCCGGGGTTCCGTTGCGGAACGGCTAGAAGGGAAATACGCCTTGGCCAT
ATACTCTTTGTCCGCATCCCTTGGGGAAATCCATTATGCTGCCGTTGACGTCGTCAACG
TCACGCCACTAACACCGAACTAGGGGGACCAAACTATACCATGTTATCCGTATACTTA
CCCACTGTTGCAGGTCAAGGTATGGGGCTGCAAGTAATGTACATACTCCTGGTGGGAA
CTTGTCCTCCGGACGTGAATGGCTACCTGCCGGGCTGGGACTTAGCAAACCACCCTGA
CGCATCCCAGGCCTTTGATCGGATCACAATTTTGCTTTCACCAGTTCCCAATTACGGTT
CCGCATCGTCGGGAGGCCTTCGACTAGATCTACTCGCATGGTTCGAGCAGTAATATCG
GGTCTGAAATCCCCTGACCCAACGGGGGGCAGTATGTGTGGTCGGAATCTGCACCTTG
CGAGAGATAATCGTAATGACGATGGACCTTCTACAAGTGAGTGCTGGGGCACGAGCGG
CTGACCAGATTGTCTTCCGAATGTTCCCCCCCGTGCCGAAGGCTGAACCCGAGGCGGA
GCCGTGGGCGTGCACCGTTGCCACGCAGGTGCACCGCGAGGCACCTTGAAGGTCCCGT
CATGAAG

>LUC_LUC201.1

GTCGGGATGCCCTTTCGACACTGGCACGGCACGGCGCCTTCGCGCACGGATCGGATAA
ACTTTCCCGTGGACGGCTCGTCCTCAGAAACATTTCGTAGTCTTCTCGTTGGTTACTCCA
CTGCCGCGCCGCGTCTGCTTGGATGCTGACCCACGAGATTTATTAGAGTACTCTACTAC
TCGACCGGGGGCAGCATAGAACCGTGGGGTCGGCGCGGCCCACCGCCGGCCAACGAG
AGTTACGAGTACCCGGCCCGTTTCCCCGCATCGGAGGCTGCCAACACGATAGTAACTG
AACCAGCCCGAGAGCATTGATACGCCTCGGGGGCGGGCCTCTGGCGCGCCACTGGATC
AGGCCCGTGGCGTGCCCGCCTCGTTAGCGCCACCCATTGCTAAGCGCTGACAGTAATA
GACCCCTCCATAGTAGTTGCCGATGTTGATTCGGTCACCGGCCGAAACGTGCGCGCTCA
GTGCAGGGCAGGTACTGTAGAGCGGAGGGTGGATGATTGGCAGGGGTTGTTGGCACGC
CTACAAAACTACTTCTCCGCTCCCGCTGGAGCGACCAACTGCGCCTCGCAGCGACTGGT
ATCCGAACAGTCTATCAACTTGGCCGATTCTATTGTATAAACAGTCTTTGGGACGCGGA
CCAAACAACCCCCTCATACCCATCCGCCCGTCCGGAGTGGAACGACTTGGCGGTACTG
GCCTCAGGCCCACTCGCCTAGATTGCATCATTATGTTGGGAGGTACATCGACTGGGGG
CTGGCGACTTGATCCCGGTCGGCCCGTAAAGCCCATCAGTCCCATGGACACGTTCTCTC

CCATCCATTGGTCTCCTGGGCTTTCCACGAATTGCGTCGAAGTAATGCCAGATGTTAAG
CGTATTCATCATAGGGCACAAGCCGCGCCTTCGTGGGTGCGGCTCGAAGCCCGACCCC
CCAGGCTGCCTAATGACACGGAAGCGTACCTGGTTCCACTCAGGAGGGACGGGACACT
CTCGACGCCTCTCAACAAGCCTCGTCCCCACGATACATACAACCGTGCCAGGTGGAAT
TAAGATGCCGGGGTCTCGTCGCGAGCTGTCCAGGGGGGAGATACGTTCCGGCCAGATA
CTCTCGGTCCACATCCCTTGAGGAAATCCAGTACGCTGCCGTTGACGTCGTTAACGCCG
CTTCATTAACGTCGAACTAGGGAGACCAAACTATACCATGTTATCCGTATGCCCACCCA
TTTTTGCGGTTCAGAGTACGGGCCTGTGAGTAATGTACGCGCCCCTGGTGGGAACTTGT
CCTCCGGACTTGAATGGTTACTCGCCGAGCTGGAACTTAGCAAACCACCCTGATGCATT
CCAGGTATCAGATTAGAACACAATTTTGCTTTCACCAGTTCCCAATTGCGGGGCCGTAT
CGTCGGGAGGCCTTCGACTAGATCTGCTCGCATGGTTCGAGCGGTAATATCGGGTCTG
AAAACCCCTGACCCAACGGGAGGCGGTATGCGTGATCGGAATCTGCACCTTGCGAGAG
CTAATCGTGATGACGATGGCTCTTCCACAAGTGAGTGCTGGGGCGCGAGCGGCGAACC
AGATGGTCCTCTGAATGTTCCCCCCCGTGCCGAAGGCTGAACTCGAGGCGGGGCCGTG
GTCGCGCACCGTTACCACGCAGGTGCGTCGCGAGTCAACTTGAAGGTCCAGTCATGAA
G

>LUC_LUC201.2

GTCGGGATGCCTTCCCAACTCCGGCACGGCGCGGCGCCTTCGCGCACGGATCGGATAA
GCTTACCCGTGGACGGCTCGTTCTCAGAAATATCTCGTAGTCTTCTCGTTGGTTACTCC
AATGAAACGCCTCGTCTGCGGGGGCACTGAACCGCGAGAGCTATTCAAGTGCTCTACT
ACTCGACCGGGGGCAGCTTACAACCGTGGGATCGGCGCGGCCCACCTCCGGTCAACGA
GAGTTACGAGTACCTGGTCCGTTTTCCCGCGTCGGAAGCCGCCAACACGATAGTAACT
GAACTGGCCCAAACGTATTAACACGCACAGGGGACGGGCCTCTGGCGCGCCACTGGAT
CAGGCCCGTGGCGTGCCCGCCTCGTCAGCGCCACCCATTGCTAAGCGCTGACAGTAAT
AGACCCCTCCATAGTAGTTGCCGATGTTAATTCGGTCACCGGCCGAAACGTGCGCGCTC
AGTGCAGGGCAGGTCCTGTAGAACGGAGGATGGATATTTGGCAAAGGCTGCTGGTGCG
CCTACCAACTTGTTCCTCCGCGCCTGCTAGAGCGACCAACTACGCTCTGTAACGTCCAG
TACCCGAGCAGTCTCTCAACTTGGCCGATGAGACTGTATACACCGCCGTTGGTACGCG
GACTAAACAACTCCCTCATACCCATCCGCCCGTCCGGAGCAGAACGGTTTGGTGGTAC
CAGCGTCAGGCCCCTTCGCCTAGGCTGCACCATTATATTGGGAAACGCGACGGCCAGA
GGCCGGCGACCTAATCTCAGCTATTCTTCGGAGCCCATCACTCCCATGGAGACGTTTTC
TCCCACCCATTGGCCCCCGGGGTCCTCCACCAATTGCGTCGGAGTCTTGTCTAATATGA
AATTTATTCATCGTAAGGTATAAACCGCCCCCGCGTGGGTGCGGCTCGAAGTCCGGCTT
CCCAAGCTGGCTGCTAACACGGAAGTGTACCCGGTTCCACTCAGAGGGCCTGAGGCAG
TACCTACGGATCTCAAAAACCCTAGTCCCCATGATACGTACAGCTGGGCTAGGTGGGA
TTAATACGTCGGGGTCCCGTCGCGGGATGTCTAGGGGGGAGATACGTTCCGGCCATCT
ACTCTCTGTCCACATCCCTTGAGGAAATCCATTGCGCTGCCGTTGACGTCGTTAACGCC
GCTTCATTAACGTCGAACTAGGGAGACCAAACTACATCATGTTATCTGTATGCCCAGTT
GTTGTTGCGGTACAGGGTACGGGGCCGTGAGGCACGGGCGCGCTCCTGGTGGAAACTT
GTCCTCCGGACTTTAATGGCTACTCGCCGGGCTGAGACTTAGCAGACCACCCTGACGC
ATTCCAGGCCTCAGATTAGATCGCAATTTTGCTTTCATTAGTTCCCAATTACGGTTCCGC
ATCGTCAGGAGGCCTTCGACTAGATCTACTCGTATGGTTCGAGCGGTGATATCGGGTCT
AAAATCCCCTGACCCAATGGGGGGCGGTACGTGTGATCGGAATCTGCACCTTGCGAAA
TCTAATCGTAATGACGATGGCTCTTCCACAAGTGAGTGCTGGGGCGCGAGCGGCGGAC
CAGATGGCCTTCCGAATGTTCCCCCCCGTGCCGAAGGCTTAACTCGAGGCGGAGCCGT
GGGCTCGCACCGTTGCCGCGCAGGTACACCGCGAGGTCCCTTGAAGGTCCCATCATGA
AG

>LUC_LUC202.1

TTCAAGATGCCCTCCTGACTCTGCCACGACGCGGTGGCTTCACGTGTGGACCGGATAA
GTTTTCCCGTGGACCGCTCAACCCCGGAAATACCCCGAGGTTTTCTCAGTGGTTACCCT
GATGCCGCGCAGCGTCTGCTTGGATGCTGAACCGCGAGAGCTATTCAGGTGCTCGACT
ACTCGACCGGGAGCAGCACGCAGCCGCGGGAACGACGCGGTCTACCTCCGGCCAGCA
GGGGGTACGACGTCCCGGCCCGTTTTCCCGCGTCGGAGGCTGCCAATACGATAGTAAC
TGCAATGGCCCAAACGTATTAATACGCACAGGGGGCGGGCCTCTGGCGCGCCACTGGA
TCAGGCCCGTGGCGTGCCCGCCTCGTCAGCGCCACCCATTGCTAAGCGCTGACAGTAA
TAGACCACTCCATATAAGTTGCCGATTTTGATTCGGTCACCGGCCGAAACGTATGCACT
TAGCACAGGGCAGGTACTACAAAGCGAGAGGGGGATGATTGGCAGGGGCTGCTGACG
CGCCTATCAGCCCGTTCCCCCGCGCCTGCTGTGGCGACCAACTACGCCCCGCAGCGTCC
AGTACCCGAGCAGTCTCTCAACTGGTCCGATTAGACTGTATACACCGCCGTTGGGACG
CGGACTAAACAACTCCCTCATACCCATCCGCCCGTCCGGAGCGGAACGACTTGGCGGT
ACCGGCGTCAGGCCCCCTCGCCTAGACTGCGCCATTATGTTGGGCGGTGCGTCGACTG
AGGGCTAGCGACTTGATCCCGGTCGGCCCGTGAAGCCCATCACTCCCATGGAGACGTT
TTCTCCCACCCATTGGCCCCCGGGGTCCTCCACCAATTGCGTCGGAGTCTTGTCTAATA
TGAAATTTATTCATCGTAAGGTATAAACCGCCCCCGCGTGGGTGCGGCTCGAAGTCCG
GCTTCCCAAGCTGGCTGCTAACACGGAAGTGTACCCGGTTCCACTCAGAGGGCCTGAG
GCAGTACCTACGGATCTCAAAAACCCTAGTCCCCATGATACGTACAGCTGGGCTAGGT
GGGATTAATACGTCGGGGTCCCGTCGCGGGATGTCTAGGGGGGAGATACGTTCCGGCC
ATCTACTCTCTGTCCACATCCCTTGAGGAAATCCATTGCGCTGCCGTTGACGTCGTTAA
CGCCGCTTCATTAACGTCGAACTAGGGAGACCAAACTACATCATGTTATCTGTATGCCC
AGTTGTTGTTGCGGTTCAGGGTACGGGGCCGTGAGGCACGGGCGCGCTCCTGGTGGGA
ACTTGTCCTCCGGACTTTAATGGCTACTCGCCGGGCTGAGACTTAGCAGACCACCCTGA
CGCATTCCAGGCCTCAGATTAGATCGCAATTTTGCTTTCATTAGTTCCCAATTACGGTTC
CGCATCGTCAGGAGGCCTTCGACTAGATCTACTCGTATGGTTCGAGCGGTAATATCGG
GTCTGAAATCCCCTGACCTAACGGGAGGTGGCACGCGCGATCGGAATCTGCAGTTTGT
GGGAGCTAATCGTAATGACGATGGACCTTCTACAAGTGAGTGCTGTAGTGCCAGCAGC
GGACCAGATTGTTTTCCGGATGTTCCCCCTCGTGCCGAAGGCTGAACTCAGGGCGGGG
CCGTGGGCGCGCACCGTTGCCACGCAGGTACACCGTGAGGCACCTTGAAGGTCCCGTA
ATGAAC

>LUC_LUC202.2

GGCGGGATGCCTTCCCAACTCCGGCACGGCGCGGCGCCTTCGCGCACGGATCGGATAA
GCTTACCCGTGGACGGCTCGTTCTCAGAAATATCTCGTAGTCTTCTCGTTGGTTACTCC
AATGAAACGCCTCGTCTGCGGGGGCACTGAACCGCGAGAGCTATTCAAGTGCTCTACT
ACTCGACCGGGGGCAGCTTACAACCGTGGGATCGGCGCGGCCCACCTCCGGTCAACGA
GAGTTACGAGTACCTGGTCCGTTTTCCCGCGTCGGAAGCTACCAACACGATAGTAACT
GAACCAGCGCGAGAGCATTGATACGCCTCGGGGGCGGGCCTCTGGCGCGCCACTGGAT
CAGGCCCGTGGCGTGCCCGCCTCGTCAGCGCTACCCATTGCTAAGCGCTGACAGTAAT
AGACCCCTCCATAGTAGTTGCCGATGTTGATTCGGTCACCGGCCGAAACGTGCGCGCTC
AGTGCAGGGCAGGTACTGTAGAGCGGAAGGTGGATGATTGGCAGGGGTTGTTGGCAC
GCCTACAAAACTATTTCTCCGCTCCCGCTGGAGCGACCAACTGCGCCTCGCAGCGACTG
GTATCCGAACAGTCTATCAACTTGGCCGATTCTATTGTATAAACAGTCTTTGGGACGCG
GACCAAACAACCCCCTCATACCCATCCGCCCGTTCGGAGTGGAACGACTTGGCAGTAT
TGGCCTCAGGCCCACTCGCCTAGATTGCATCATTATGTTGGGAGGTACATCGACTGGGG
GCTGGCGACTTGATCCCGGTCGGCCCGTAAAGCCCATCAGTCCCATGGACACGTTCTCT
CCCATCCATTGGTCTCCTGGGCTTTCCACGAATTGCGTCGAAGTAATGCCAGATGTTAA
GCGTATTCATCATAGGGCACAAGCCGCGCCTTCGTGGGTGCGGCTCGAAGCCCGACCC
CCCAGGCTGCCTAATGACACAGAAGCGTACCTGGTTCCGCTCAGGAGGGACGGACAC
TCTCGACGCCTCTCAACAAGCCTAGTCCCCACGATACATACAACCGTGCCAGGTGGAA
TTAAGATGCCGGGGTCTCGTCGCGAGCTGTCCAGGGGGGAGATACGTTCCGGCCATAT

ACTCTCTGTCCACATCCCTTGAGGAAATCCATTACGCTGCCGTTGACGTCGTTAACGCC
GCTTCATTAACGTCGAACCAGGGAGACCAAACTATACCATGTTATCCGTATGCCCACCC
ATTTTTGCGGTTCAGAGTACGGGCCTGTGAGTAATGTACGCGCTCCTGGTGGGAACTTG
TCCTCCGGACTTGAATGGTTACTCGCCGAGCTGGAACTTAGCAAACCACCCTGATGCAT
TCCAGGTATCAGATTAGAACACAATTTTGCTTTCACCAGTTCCCAATTGCGGGGCCGTA
TCGTCGGGAGGCCTTCGACTAGATCCGCTCGCATGGTTCGAGCGGTAATATCGGGTCTG
AAATCCCCTGACCCAACGGGAGGCGGTATGCGTGATCGGAATCTGCACCTTGCGAGAG
CTAATCGTGATGACGATGGCTCTTCCACAAGTGAGTGCTGGGGCGCGAGCGGCGAACC
AGATGGTCCTCTGAATGTTCCCCCCCGTGCCGAAGGCTGAACTCGAGGCGGGGCCGTG
GTCGCGCACCGTTACCACGCAGGTGCGTCGCGAGTCAACTTGAAGGTCCAGTCATGAA
G

>LUC_LUC206.1

GTCGGGATGCCTTCCCAACTCCGGCACGGCGCGGCGCCTTCGCGCACGGATCGGATAA
GCTTACCCGTGGACGGCTCGTTCTCAGAAATATCTCGTAGTCTTCTCGTTGGTTACTCC
AATGCCGCGCCGCGTCTGCTTGGATGCTGAACCACGAGATTTATTAGAGTACTCTACTA
CTCGACCGGGGGCAGCATACAACCGTGGGGTCGGCGCGGCCCACCTCCGGCCAACGAG
AGTTACGAGTACCCGGCGCGTTTTCTCACGTCGGAGACTGTTAACACGATAGTAACTG
AAATGACCCAAACGTATTAATACGCACAGGGGGCGGGCCTCTGGCGCGCCACTGGATC
AGGCCCGTGGCGTGCCCGCCTCGTCAGCGCCACCCATTGCTAAGCGCTGACAGTAATA
GACCCCTCCATAGTAGTTGCCGATGTTGATTCGGTCACCGGCCGAAACGTGCGCGCTCA
GTGCAGGGCAGGTACTGTAGAGCGGAGGGTGGATGATTGGCAGGGGTTGTTGGCACGC
CTACAAAACTATTTCTCCGCTCCCGCTGGAGCGACCAACTGCGCCTCGCAGCGACTGGT
ATCCGAACAGTCTATCAACTTGGCCGATTCTATTGTATAAACAGTCTTTGGGACGCGGA
CCAAACAACCCCCTCATACCCATCCGCCCGTCCGGAGTGGAACGACTTGGCGGTATTG
GCCTCAGGCCCACTCGCCTAGATTGCATCATTATGTTGGGAGGTACATCGACTGGGGG
CTGGCGACTTGATCCCGGTCGGCCCGTAAAGCCCATCAGTCCCATGGACACGTTCTCTC
CCATCCATTGGTCTCCTGGGCTTTCCACGAATTGCGTCGAAGTAATGCCAGATGTTAAG
CGTATTCATCATAGGGCACAAGCCGCGCCTTCGTGGGTACGGCTCGAAGCCCGACCCC
CCAGGCTGCCTAATGACACGGAAGTGTACCTGGTTCCACTCAAAAGGCATGAGGCAGT
CCCTACAGATCGTAGCAAGCTAAGTCCCCACGATACATACAACCGGGCCAGGTGGGAT
TAAGACGCCGGGGTCCCGTCGCGGGACATCTGGGATGGGAACACGTTCCGGTCATATA
CTCTTTGTCCGCATCCCTTGGGGAAATCCATTACGCTGTCATTGACGTCGTCAACGTCA
TGCCACTAACACCGAACTAGGGGGACCAAACTATACCATGTTATCCGTATGCCTACTC
GTTGTCGCGGTTCAGAGTACGGGCCTGTGAGTAATGTACGCGCTCCTGGTGGGAACTT
GTCCTCCGGACTTGAATGGTTACTCGCCGAGCTGGAACTTAGCAAACCACCCCAACAT
ATTCCAGGCCTCTGATCAGATCACAATTTTGCTTTCACCAGTTCCCAATTACGGTTCCG
CATCGTCGGGAGGCCTTCGACTAAATCTACTCGCAAGGTTCGAGCGGTAATATCGGGT
CTGAAATCCCCTGCCCCAACGGGAGGCGGTACGTGTGATCGGAAGCTGCACCTCGCGA
GAGCCAAGCGGAATGACGATGGCTCTTCCACAAGTGAGTGGTGTAGCGCCAGCGGCGG
ACCAGATGGTCTTCCGAATGTTCCCCCCCGTGCCGAAGGCTGAATTCGAGATGGAGCA
GTGGGCTCGCACCGTTGCCACGCAGGTGCACCGCGAGGCAACTTGAAGGTCCAGTCAT
GAAG

>LUC_LUC206.2

GTCGGGATGCCTTCCCAACTCCGGCACGGCGCGGCGCCTTCGCGCACGGATCGGATAA
GCTTACCCGTGGACGGCTCGTTCTCAGAAATATCTCGTAGTCTTCTCGTTGGTTACTCC
AATGAAACGCCTCGTCTGCGGGGGCACTGAACCGCGAGAGCTATTCAAGTGCTCTACT
ACTCGACCGGGGGCAGCTTACAACCGTGGGATCGGCGCGGCCCACCTCCGGTCAACGA
GAGTTACGAGTACCTGGTCCGTTTTCCCGCGTCGGAAGCTACCAACACGATCGTAACTG

AACCAGCGCAAACGCATTGATACGCCTCGGGGGCGGGCCGCTGGCGCGCCACTGGATC
AGGCCCGTGGCGTGCCCGCCTCGTCAGCGCCACCCATTACTAAGCGCTGACAGTAATT
GACCCCTCCATAGTAGTTGCCGATGCTAATTCGGTCACCGGCCGAAACGTATGCACTTA
GCACAGGGCAGGTACTACAAAGCGAGAGGGGGATGATTGGCAGGGGCTGCTGACGCG
CCTATCAGCCCGTTCCCCCGCGCCTGCTGTGGCGACCAACTATGCCCCGCAGCGTCCAG
TACCCGAGCAGTCTCTCAACTGGTCCGATTAGACTGTATACACCGCCGTTGGGACGCG
GACTAAACAACTCCCTCATACCCATCCGCCCGTTCGGAGCGGAACGACTTGGCAGTAC
CGGCGTCAGGCCCCCTCGCCTAGACTGCGCCATTATGTTGGGCGGTGCGTCGACTGAG
GGCTAGCGACTTGATCCCGGTCGGCCCGTGAAGCCCATCACTCCCATGGAGACGTTTTC
TCCCACCCATTGGCCCCCGGGGTCCTCCACCAATTGCGTCGAAGTAATGCCAGATGTTA
AGCGTATTCATCATAGGGCACAAGCCGCGCCTTCGTGGGTGCGGCTCGAAGCCCGACC
CCCCAGGCTGCCTAATGACACAGAAGCGTACCTGGTTCCACTCAGGAGGGACGGGACA
CTCTCGACGCCTCTCAACAAGCCTCGTCCCCACGATACATACAACCGTGCCAGGTGGA
ATTAAGATGCCGGGGTCTCGTCGCGAGCTGTCCAGGGGGGAGATACGTTCCGGCCATA
TACTCTTTGTCCACATCCCTTGAGGAAATCCATTACGCTGCCGTTGACGTCGTTAACGC
CGCTTCATTAACGTCGAACTAGGGAGACCAAACTATACCATGTTATCCGTATGCCCACC
CATTTTTGCGGTTCAGAGTACTGGCCTGTGAGTAATGTACGCGCTCCTGGTGGGAACTT
GTCCTCCGGACTTGATAGGTTACTCGCCGAGCTGGAACTTAGCAAACCACCCTGATGC
ATTCCAGGTATCAGATTAGAACACAATTTTGCTTTCACCAGTTCCCAATTGCGGGGCCG
TATCGTCGGGAGGCCTTCGACTAGATCCGCTCGCATGGTTCGAGCGGTAATATCGGGTC
TGAAATCCCCTGACCCAACGGGAGGCGGTATGCGTGATCGGAATCTGCACCTTGCGAG
AGCTAATCGTGATGACGATGGCTCTTCCACAAGTGAGTGCTGGGGCGCGAGCGGCGAA
CCAGATGGTCCTCTGAATGTTCCCCCCCGTGCCGAAGGCTGAACTCGAGGCGGGGCCG
TGGTCGCGCACCGTTACCACGCAGGTGCGTCGCGAGTCAACTTGAAGGTCCAGTCATG
AAG

>LUC_LUC207.1

GTCGGGATGCCCTTTCGACACTGGCACGGCACGGCGCCTTCGCGCACGGATCGGATAA
ACTTTCCCGCGGACGGCTCGTCCTCAGAAACATTTCGTAGTCTTCTCGTTGGTTACTCC
ACTGCCGCGCCGCGTCTGCTTGGATGCTGAACCACGAGATTTATTAGAGTACTCTACTA
CTCGACCGGGGGCAGCATACAACCGTGGGGTCGGCGCGGCCCACCTCCGGCCAACGAG
AGTTACGAGTACCCGGCCCGTTTCCCCGCATCGGAGGCTGCCAACACGATAGTAACTG
AACTGGCCCAAACGTATTAACACGCACAGGGGACGGGCCTCTGGCGCGCCACTGGATC
AGGCCCGTGGCGTGCCCGCCTCGTCAGCGCCACCCATTGCTAAGCGCTGACAGTAATA
GACCCCTCCATAGTAGTTGCCGATGTTAATTCGGTCACCGGCCGAAACGTGCGCGCTCA
GTGCAGGGCAGGTCCTGTAGAACGGAGGATGGATATTTGGCAAAGGCTGCTGGTGCGC
CTACCAACTTGTTCCTCCGCGCCTGCTGGAGCGACCAACTACGCTCTGTAACGTCCAGT
ACCCGAGCAGTCTCTCAACTTGGCCGATGAGACTGTATACACCGCCGTTGGTACGCGG
ACTAAACAACTCCCTCATACCCATCCGCCCGTCCGGAGCAGAACGGTTTGGTGGTACC
AGCGTCAGGCCCCTTCGCCTAGGCTGCACCATTATATTGGGAAACGCGACGGCCAGAG
GCCGGCGACCTAATCTCAGCTATTCTTTGAAGCCCATCACTCCCAGGGAGACGTTTTCT
CCCACCCATTGGCCCCCGGGGTCCTCCACCAATTGCGTCGGAGTCTTGTCTAATATGAA
ATTTATTCATCGTAAGGTATAAACCGCCCCCGCGTGGGTGCGGCTCGAAGTCCGGCTTC
CCAAGCTGGCTGCTAACACGGAAGTGTACCCGGTTCCACTCAGAGGGCCTGAGGCAGT
ACCTACGGATCTCAAAATCCCTAGTCCCCATGATACGTACAGCTGGGCTAGGTGGGAT
TAATACGTCGGGGTCCCGTCGCGGGATGTCTAGGGGGGAGATACGTTCCGGCCATCTA
CTCTCTGTCCACATCCCTTGAGGAAATCCATTGCGCTGCCGTTGACGTCGTTAACGCCG
CTTCATTAACGTCGAACTAGGGAGACCAAACTACATCATGTTATCTGTATGCCCAGTTG
TTGTTGCGGTACAGGGTACGGGGCCGTGAGGCACGGGCGCGCTCCTGGTGGAAACTTG
TCCTCCGGACTTTAATGGCTACTCGCCGGGCTGAGACTTAGCAGGCCACCCTGACGCAT
TCCAGGCCTCAGATTAGATCGCAATTTTGCTTTCATTAGTTCCCAATTACGGTTCCGCAT

CGTCAGGAGGCCTTCGACTAGATCTACTCGTATGGTTCGAGCGGTGATATCGGGTCTAA
AATCCCCTGACCCAATGGGGGGCGGTACGTGTGATCGGAATCTGCACCTTGCGAGATC
TGATCGTAATGACGATGGCTCTTCCACAAGTGAGTGCTGGGGCGCGAGCGGCGGACCA
GATGGCCTTCCGAATGTTCCCCCCCGTGCCGACGGCTTAACTCGAGGCGGAGCCGTGG
GCTCGCACCGTTGCCGCGCAGGTACACCGCGAGGTCCCTTGAAGGTCCCATCATGAAG

>LUC_LUC207.2

GTCGGGATGCCCTTTCGACACTGGCACGGCACGGCGCCTTCGCGCACGGATCGGATAA
ACTTTCCCGTGGACGGCTCGTCCTCAGAAACATTTCGTAGTCTTCTCGTTGGTTACTCCA
CTGCCGCGCCGCGTCTGCTTGGATGCTGAACCACGAGATTTATTAGAGTACTCTACTAC
TCGACCGGGGGCAGCATACAACCGTGGGTCGGCGCGGCCCACCTCCGGCCAACGAG
AGTTACGAGTACCCGGCCCGTTTCCCCGCATCGGAGGCTGCCAACACGATAGTAACTG
AACTGGCCCAAACGTATTAACACGCACAGGGGACGGGCCTCTGGCGCGCCACTGGATC
AGGCCCGTGGCGTGCCCGCCTCGTCAGCGCCACCCATTGCTAAGCGCTGACAGTAATA
GACCCCTCCATAGTAGTTGCCGATGTTAATTCGGTCACCGGCCGAAACGTGCGCGCTCA
GTGCAGGGCAGGTCCTGTAGAACGGAGGGTGGATATTTGGCAAAGGCTGCTGGTGCGT
CTACCAACTTGTTCCTCCGCGCCTGCTAGAGCGACCAACTACGCTCTGTAACGTCCAGT
ACCCGAGCAGTCTCTCAACTTGGCCGATGAGACTGTATACACCGCCGTTGGTACGCGG
ACTAAACAACTCCCTCATACCCATCCGCCCGTCCGGAGCAGAACGGTTTGGTGGTACC
AGCGTCAGGCCCCTTCGCCTAGGCTGCACCATTATATTGGGAAACGCGACGGCCAGAG
GCCGGCGACCTAATCTCAGCTATTCTTCGGAGCCCATCACTCCCATGGAGACGTTTTCT
CCCACCCATTGGCCCCCGGGGTCCTCCACCAATTGCGTCGGAGTCTTGTCTAATATGAA
ATTTATTCATCGTAAGGTATAAACCGCCCCCGCGTGGGTGCGGCTCGAAGTCCGGCTTC
CCAAGCTGGCTGCTAACACGGAAGTGTACCCGGTTCCACTCAGAGGGCCTGAGGCAGT
ACCTACGGATCTCAAAAACCCTAGTCCCCATGATACGTACAGCTGGGCTAGGTGGGAT
TAATACGTCGGGGTCCCGTCGCGGGATGTCTAAGGGGGAGATACGTTCCGGCCATCTA
CTCTCTGTCCACATCCCTTGAGGAAATCCATTGCGCTGCCGTTGACGTCGTTAACGCCG
CTTCATTAACGTCGAACTAGGGAGACCAAACTACATCATGTTATCTGTATGCCCAGTTG
TTGTTGCGGTACAGGGTACGGGGCCGTGAGGCACGGGCGCGCTCCTGGTGGAAACTTG
TCCTCCGGACTTTAATGGCTACTCGCCGGGCTGAGACTTAGCAGACCACCCTGACGCAT
TCCAGGCCTCAGATTAGATCGCAATTTTGCTTTCATTAGTTCCCAATTACGGTTCCGCAT
CGTCAGGAGGCCTTCGACTAGATCTACTCGTATGGTTCGAGCGGTGATATCGGGTCTAA
AATCCCCTGACCCAATGGGGGGCGGTACGTGTGATCGGAATCTGCACCTTGCGAGATC
TAATCGTAATGACGATGGCTCTTCCACAAGTGAGTGCTGGGGCGCGAGCGGCGGACCA
GATGGCCTTCCGAATGTTCCCCCCCGTGCCGAAGGCTTAACTCGAGGCGGAGCCGTGG
GCTCGCACCGTTGCCGCGCAGGTACACCGCGAGGTCCCTTGAAGGTCCCATCATGAAG

>LUC_LUC208.1

GTCGGGATGCCCTCTCGATACTGGCACGGCGCGGCGCCTTCGCGCACGGATCAGATAA
GTTTTCTCGTGGACCGCTCAACCTCGGAAATATCTCATAGTCTTCTCGTTGGTTACTCCA
ATGAAGCGCCTCGTCTGCGGGGGCACTGCACCGCGAGAGCTATTCAAGTGCTCTACTA
CTCGACCGGGGGCAGCTTACAACCGCGGGATCGGCACGGTCTACCTCCGTCCAACAGG
GGGTACGAGTACCCGACGCGTTTTCCCGCGTCGGAGGCTGCCAACACGATAGTAACTG
AACTGGCCCAAACGTATTATTACGCCCCGGGGGCGGGCCTCTGGCGCGCCACTGGATC
AGGCCCGTGGCGTGCCCGCCTCGTCAGCGCCACCCATTGCTAAGCGCTGACAGTAATA
GACCCCTCCATAGTAGCTGCCGATGTTAATTCGGTCACCGGCCGAAACGTGCGCGCTC
AGTGCAGGGCAGGTCCTGTAGAACGGAGGGGGAATATTTGGCAAAGGCTGCTGGTGC
GCCTACCAACTTGTCCCTCCGCGCTTGCTGGAGCGACCAACTACGCTCTGTAACGTCCA
GTACCCGAGCAGTCTCTCAACTTGGCCGATGAGACTGTATACACCGCCGTTGGTACGC
GGACTAAACAACTCCCTCATACCCATCCGCCCGTCCGGCGCAGAACGGTTTGGTGGTA

CCAGCGTCAGGCCCCTTCGCCTAGGCTGCACCATTATATTGGGAAACGCGACGGCCAG
AGGCCGGCGACCTAATCTCAGCTATTCTTTGAAGCCCATCACTCCCAGGGAGACGTTTT
CTCCCACCCATTGGCCCCCGGGGTCCTCCACCAATTGCGTCGGAGTCTTGTCTAATATG
AAATTTATTCATCGTAAGGTATAAACCGCCCCCGCGTGGGTGCGGCTCGAAGTCCGGC
TTCCCAAGCTGGCTGCTAACACGGAAGTGTACCCGGTTCCACTCAGAGGGCCTGAGGC
AGTACCTACGGATCTCAAAAACCCTAGTCCCCATGATACGTACAGCTGGGCTAGGTGG
GATTAATACGTCGGGGTCCCGTCGCGGGATGTCTAGGGGGGAGATACGTTCCGGCCAT
CTACTCTCTGTCCACATCCCTTGAGGAAATCCATTGCGCTGCCGTTGACGTCGTTAACG
CCGCTTCATTAACGTCGAACTAGGGAGACCAAACTACATCATGTTATCTGTATGCCCAG
TTGTTGTTGCGGTACAGGGTACGGGGCCGTGAGGCACGGGCGCGCTCCTGGTGGGAAC
TGGTCCTCCGGACTTTAATGGCTACTCGCCGGGCTGAGACTTAGCAGGCCACCCTGACG
CATTCCAGGCCTCAGATTAGATCGCAATTTTGCTTTCATTAGTTCCCAATTACGGTTCCG
CATCGTCAGGAGGCCTTCGACTAGATCTACTCGTATGGTTCGAGCGGTGATATCGGGTC
TAAAATCCCCTGACTCAATGGGGGGCGGTACGTGTGATCGGAATCTGCACCTTGCGAG
ATCTAATCGTAATGACGATGGCTCTTCCACAAGTGAGTGCTGGGGCGCGAGCGGCGGA
CCAGATGGCCTTCCGAATGTTCCCCCCCGTGCCGAAGGCTTAACTCGAGGCGGAGCCG
TGGGCTCGCACCGTTGCCGCGCAGGTACACCGCGAGGTCCCTTGAAGGTCCCATCATG
AAG

>LUC_LUC208.2

GTCGGGATGCCTTCCCAACTCCGGCACGGCGCGGCGCCTTCGCGCACGGATCGGATAA
GCTTACCCGTGGACGGCTCGTTCTCAGCAATATCTCGAGGTCTTCCCAGTGGTTACTCT
GATGCCGCGCCGCGTCTGCTTGGATGCTGAACCGAGAGAGCCATTCGAGTGCTCTACA
ACTCGACTGGGGACAGCACACAATTGTGGGATCGGCGCGGCCCACCTCCGGCCAACGA
GAGTTACGAGTACCCGGCCCGTTTCCCCGCATCGGAGGCTGCCAACACGATAGTAACT
GAACTGGCCCAAACGTATTAACACGCACAGGGGACGGGCCTCTGGCGCGCCACTGGAT
CAGGCCCGTGGCGTGCCCGCCTCGTCAGCGCCACCCATTGCTAAGCGCTGACAGTAAT
AGACCCCTCCATATAAGTTGCCGATTTTGATTCGGTCACCGACCGAAACGTATGCACTT
AGCACAGGGCAGGTACTACAAAGCGAGAGGGGGATGATTGGCAGGGGCTGCCGACGC
GCCTATCAGCCCGTTCCCCCGCGCCTGCTGTGGCGACCGACTACGCCCCGCAGTGACCG
GTACCCGAGCAGTCTCCCATCTGGTCTGATGAGACTGTATACACCGCCGTTGGGATGCG
GACTAAACAACCCCCTCATACCCATCCGCTTGTCCGCAGCAGAACGACTCGGCGGTAC
TGGCGTCAGGCCCCCTCGCCTAGACTGCACCATTATGTTGGGAGGTGCGTCGACTGGG
GGCTGGCAACCTGAGGTCGGCCGTTTCGTGAAGCCCATCAGTCCCATGGAGACGTTCT
CTCCCATCCATTGGCCTCCGGGGCTCTTCATCAATCGCGTCGGAGTATCGTCTAATGTG
AAATTTATTCATCGTGAGGTATACACCGCCCCCGCGTGGGTGCGGCTCGAAGCCCGGC
TTCCCAGGCTGGCAGCTAACACGGAAATGTGCCTCGTTCCACTCAGAGGGCATGAGGC
AGTACCTACGGATCTCAACAAGCCTAGTCCCCATGATACGTTCAGCTGGGCCAGGTGG
GATTAATACGCCGGGGTCCCGTCGCGGAACGGCTAGAAGGTAAATACGCCTTGGCCAT
ATACTCTTTGTCCGCATCCCTTGGGGAAATCCATTATGCTGCCGTTGACGGCGTCAACG
TCACGCCACTAACACCGAACTAGGGGGACCAAACTATACCATGTTATCCGTATACTTA
CCCACTGTTGCAGGTCAAGGTTTGGGGCTGCAAGTAATGTACATACTCCTGGTGGGAA
CTTGTCCTCCGGACTTGAATGGCTACCTGCCGGGCTGGGACTTAGCAAACCACCCTGAC
GCATCCCAGGCCTTTGATCGGATCACAATTTTGCTTTCACCAGTTCCCAATTACGGTTC
CGCATCGTCGGGAGGCCTTCGACTAGATCTACTCGCATGGTTCGAGCAGTAATATCGG
GTCTGAAATCCCCTGACCCAACGGGGGGCAGTATGTGTGGTCGGAATCTGCACCTTGC
GAGAGATAATCGTAATGACGATGGACCTTCCACAAGTGAGTGCTGGGGCACGAGCGGC
TGACCAGATTGTCTTCCGAATGTTCCCCCCCGTGCCGAAGGCTGAACCCGAGGCGGAG
CCGTGGGCGTGCACCGTTGCCACGCAGGTGCACCGCGAGGCACCTTGAAGGTCCCGTC
ATGAAG

>LUC_LUC2016.1

GTCGGGATGCCCTTTCGACACTGGCACGGCACGGCGCCTTCGCGCACGGATCGGATAA
ACTTTCCCGTGGACGGCTCGTCCTCAGAAACATTTCGTAGTCTTCTCGTTGGTTACTCCA
CTGCCGCGCCGCGTCTGCTTGGATGCTGAACCACGAGATTTATTAGAGTACTCTACTAC
TCGACCGGGGGCAGCATACAACCGTGGGGTCGGCGCGGCCCACCTCCGGCCAACGAG
AGTTACGAGTACCCGGCCCGTTTCCCCGCATCGGAGGCTGCCAACACGATAGTAACTG
AACTGGCCCAAACGTATTAACACGCACAGGGGACGGGCCTCTGGCGCGCCACTGGATC
AGGCCCGTGGCGTGCCCGCCTCGTCAGCGCCACCCATTGCTAAGCGCTGACAGTAATA
GACCCCTCCATAGTAGTTGCCGATGTTAATTCGGTCACCGGCCGAAACGTGCGCGCTCA
GTGCAGGGCAGGTCCTGTAGAACGGAGGGTGAATATTTGGCAAAGGCTGCTGGTGCGC
CTACCAACTTGTTCCTCCGCGCCTGCTAGAGCGACCAACTACGCTCTGTAACGTCCAGT
ACCCGAGCAGTCTCTCAACTTGGCCGATGAGACTGGATACACCGCCGTTGGTACGCGG
ACTAAACAACTCCCTCATACCCATCCGCCCGTCCGGAGCAGAACGGTTTGGTGGTACC
AGCGTCAGGCCCCTTCGCCTAGGCTGCACCATTATATTGGGAAACGCGACGGCCAGAG
GCCGGCGACCTAATCTCAGCTATTCTTTGAAGCCCATCACGCCCATGGAGACGTTTTCT
CCCACCCATTGGCCCCCGGGGTCCTCCACCAATTGCGTCGGAGTCTTGTCTAATATGAA
ATTTATTCATCGTAAGGTATAAACCGCCCCCGCGTGGGTGCGGCTCGAAGTCCGGCTTC
CCAAGCTGGCTGCTAACACGGAAGTGTACCCGGTTCCACTCAGAGGGCCTGAGGCAGT
ACCTACGGATCTCAAAAACCCTAGTCCCCATGATACGTACAGCTGGGCTAGGTGGGAT
TAATACGTCGGGGTCCCGTCGCGGGATGTCTAGGGGGGAGATACGTTCCGGCCATATA
CTCCCTGTCCACATCCCTTGAGGACATCCATTGCGCTGCCGTTGACGGCGTTAACGCCG
CTTCATTAACGTCGAACTAGGGAGACCAAACTACATCATGTTATCCGTATGCCCAGTTG
TTGTTGCGGTACAGGGTACGGGGCCGTGAGGCACGGGCGCGCTCCTGGTGGGAACTTG
TCCTCCGGACTTTAATGGCTACTCGCCGGGCTGAGACTTAGCAGACCACCCTGACGCAT
TCCAGGCCTCAGATTAGATCGCAATTTTGCTTTCATTAGTTCCCAATTACGGTTCCGCAT
CGTCAGGAGGCCTTCGACTAGATCTACTCGTATGGTTCGAGCGGTGATATCGGGTCTAA
AATCCCCTGACTCAATGGGGGGCGGTACGTGTGATCGGAATCTGCACCTTGCGAGATC
TAATCGTAATGACGATGGCTCTTCCACAAGTGAGTGCTGGGGCGCGAGCGGCGGACCA
GATGGCCTTCCGAATGTTCCCCCCCGTGCCGAAGGCTTAACTCGAGGCGGAGCCGTGG
GCTCGCACCGTTGCCGCGCAGGTACACCGCGAGGTCCCTTGAAGGTCCCATCATGGAG

>LUC_LUC2016.2

GTCGGGATGCCCTCTCGATACTGGCACGGCGCGGCGCCTTCGCGCACGGATCAGATAA
GTTTTCCCGTGGACCGCTCAACCTCGGAAATACCCCGAGGTTTTCCCGTTGGTTACTCT
GATGCCGCGCAGCGTCTGCTTGGATGCTGAACCGCGAGAGCTATTCAAGTGCTCTACT
ACTCGACCGGGGGCAGCTTACAACCGCGGGATCGGCACGGTCTACCTCCGTCCAACAG
GGGGTACGACGACCCGGTCCCCCCTCCCGCGTCGAAGGCTGCCAACACGATAGTAAGC
GCCCTGGCCCAAACGTATGAATACGTACAGGGGGCGGGCCGCTGGCGCGCCACTGGAT
CAGGCCCGTGGCGTGCCCGCCTCGTCAGCGCTACCCATTGCTAAGCGCTGACAGTAATT
GACCCCTCCATAGTAGTTGCCGATGTTAATTCGGTCACCGGCCGAAACGTGCGCGCTCA
GTGCAGGGCAGGTCCTGTAGAACGGAGGGTGAATATTTGGCAAAGGCTGCTGGTGCGC
CTACCAACTTGTTCCTCCGCGCTTGCTGGAGCGACCAACTACGCTCTGTAACGTCCAGT
ACCCGAGCAGTCTCTCAACTTGGCCGATGAGACGGTATACACCGCCGTTGGGACGCGG
ACTAAACAACTCCCTCATACCCATCCGCCCGTCCGGAGCAGAACGACACGGCGGTACC
GGCGTCAGGCCCCCTCGCCTGAACTGCATCATTATGATGGGAGGTGCGTCGACTGGGG
GCCGGCGACTTGATCCCGGTCGGCCCGTGAAGCCCATCAGTCCCATGGGGACGTTCTC
CCCCATCCATTGGCCTCTGGGGCTTTCCACCAATTGCACCAGAATAATGCCAGATGTTA
GATGTATTCATCGTGGGGCACGAGCCTTCCCCGCGTGAGTGCGGCTCGAAATCCGGTC
CCCCAGGCTGCCAGCTAACACGGAAGTGTGCCTCGTTTCGCTCAGAGGGGACGAGACA
CTCTCGATGGCTCTCAACAAGCCTAGTCTCCACGATACATACAATCGTGCCAGGTGGG

ATTAATACGCCGGAAGCCCATCGCGAGATGTTTGAGGGAGAGATACGTTCCGGCCATA
TACTCCCTGTCCGCATCCCTTGGGGAAATCCGTTACACTGCCGTTGACGTCGTTAACGC
CACGCCACTAACACCGAACTAGGAGGACCAAACTATACCATGTTATCCGTATGCCCAG
TTGTTGTTGCGGTTCAGGGTACGGGGCTGTGAGGCACGTACGCGTTCCTGGTGGGAACT
TGTCCTCTGAACTTGAATAACAGCTCACTGGAATGGGATTTAGCAAACCATCCCAACAT
ATTCCAGGCCTCTGATCAGATCACAATTTTGCTTTCACCAGTTCCCAATTATGATTCCGC
ATCGTCGGGAGGCCTTCGACTAGATCTACTCGCGTGGTTCGAGCGGTAATATCGGGTCT
GAAATCCCCTGACCTAACGGGAGGTGGCACGCGCGATCGGAATCTGCAGTGTGTGGGA
GCTAATCGTAATGACGATGGACCTTCTACAAGTGAGTGCTGTAGCGCCAGCAGCGGAC
CAGATTGTTTTCCGGATGTTCCCCCTCGTGCCGAAGGCTGAACTCAGGGCGGGGCCGTG
GGCGCGCACCGTTGCCACGCTGGTACACCGTGAGGCACCTTGAAGGTCCCGTAATGAA
C

>WZS_WZS809.1

GTCGGGATGCCCTCCCGACACCAGCACGGCGCGGCGCTCTCGCGCATGTACCAGATGG
GCTTTCCCGTGGATGGCTCGTCCTCGGAGATATCTCGTAGTCTTCTCGTGGGTTACTCC
AATGAAGTGCCTCGTCTGCGTGGACACTGAACCGCGAGATTTATTAGAGTACTCTACTA
CTCGACCGGGGGCAGCATACAACCGTGGGATCGGCGCGGCCCACCTCCGGCCAACGAG
AGTTACGAGTACCCGGCCCGTTTTCCCGCGTCGGAGGCTGCCAACACGATAGTAACTG
AACTGGCCCAAACGTATTAATACGCCCCGGGGGCGGGCCTCTGGCGCGCCACTGGATC
AGGCCCGTGGCGTGCCCGCCTCGTCAGCGCCACCCATTGCTAAGCGCTGACAGTAATA
GACCCCTCCATAGTAGTTGCCGATGTTGATTCGGTCACCGGCCGAAACGTATGCACTTA
ACACAGGGCAGGTACTACAAAGCGAGAGGGGGATGATTGGCAGGGGCTGCTGACGCG
CCTATCAACTTGTTCCTCCGCGCCTGCTGGAGCGACCAACTACGCTCTGTAACGTCCAG
TACCCGAGCAGTCTCTCAATTGGTCCGATGAGACGGTATACGCCGCCGTTGGGACGCG
GACTAAACAACCCCCTCATACCCATTCGCCCGTCCAGAGCGGAACGACTCCGCGGTGC
CGGCGTCAGGCCTCCTCGCCTAGGCGGCGCCATTATATTGGGAGGTGCGTCAACTGGG
GGCCGGCAACCTGAGGTTGGTCGGCCCGTGAAGCCCATCAGTCCCATGGACACGTTCT
CTCCCATCCATTGGTCTCCTGGGCTTTCCACGAATTGCGTCGAAGTAATGCCAGATGTT
AAGCGTATTCATCATAGGGCACAAGCCGCGCCTTCGTGGGTGCGGCTCGAAGCCCGAC
CCCCCAGGCTGCCTAATGACACAGAAGCGTACCTGGTTCCACTCAAAAGGCATGAGGC
AGTCCCTACAGATCGTAGCAAGCTAAGTCCCCACGATACATACAACCGGGCCAGGTGG
GATTAAGACGCCGGGGTCCCGTCGCGGGACATCTGGGATGGGAACACGTTCCGGTCAT
ATACTCTTTGTCCGCATCCCTTGGGGAAATCCATTACGCTGTCATTGACGTCGTCAACG
TCATGCCACTAACACCGAACTAGGGGGACCAAACTATACCATGTTATCCGTATGCCTA
CTCGTTGTCGCGGTTCAGAGTACGGGCCTGTGAGTAATGTACGCGCTCCTGGTGGAAA
CTTGTCCTCCGGACTTGAATGGTTACTCGCCGAGCTGGAACTTAGCAAACCACCCCAAC
ATATTCCAGGCCTCTGATCAGATCACAATTTTGCTTTCACCAGTTCCCAATTACGGTTCC
GCATCGTCGGGAGGCCTTCGACTAAATCTACTCGCAAGGTTCGAGCGGTAATATCGGG
TCTGAAATCCCCTGACCCAACGGGAGGCGGTACGTGTGATCGGAAGCTGCACCTCGCG
AGAGCCAAGCGGAATGACGATGGCTCTTCCACAAGTGAGTGGTGTAGCGCCAGCGGCG
GACCAGATGGTCTTCCGAATGTTCCCCCCCGTGCCGAAGGCTGAATTCGAGATGGAGC
AGTGGGCTCACACCGTTGCCACGCAGGTGCACCGCGAGGCAACTTGAAGGTCCAGTCA
TGAAG

>WZS_WZS809.2

GTCGGGATGCCTTCCCAACTCCGGCACGGCGCGGCGCCTTCGCGCACGGATCGGATAA
GCTTACCCGTGGACGGCTCGTCCTCAGAAACACCTCGTAGTCTTCTCGTTGGTTACTCC
ACTGCCGCGCCGCGTCTGCTTGGATGCTGAACCACGAGATTTATTAGAGTACTCTACTA
CTCGACCGGGGGCAGCATACAACCGTGGGGTCGGCGCGGCCCACCTCCGGCCAACGAG

AGTTACGAGTACCCGGCCCGTTTTCCCGCGTCGGAGGCTGCCAATACGATAGTAACTG
AAATGGCCCAAACGTACTAATACGCCCCGGTTGCGGGCCTCTGGCGCGCCACTGGATC
AGGCCCGTAGCGTGCCCGCCTAGTCAGCGCCACCCATTGCTAAGCGCTGACAGTAATA
GACCCCTCCATAGTAGTTGCCGATGTTGATTCGGTCACCGGCCGAAACGTATGCACTCA
GCACAGGGCAGGTACCACGGAGCGAAAGGTGGATGATTGGCAGGGGCCGCTTGCGCA
CCTACATAACTATTCGTCCGCGCCTGCTGGAGCGACCAACTACGCTCTATAGCGTTCAG
TACCCGAGCAGTCTCCCAACTTGGCCGATTCTATTGTATAAACAGTCGTTGGGACGCGG
ACTAAACAACCTCCTCATACCCATCCTCCCGTTCGCAGCAGAACGACTCGACGGTACC
GGCGTCAAGCGCCTCCGCCTAGGCTGCATCATTATGTTGGGAGATGCGTCGACTGGGG
GCCGGCGACCTGAGCTCGGTCGTCCCGTGAAGCCAATCAGTCTCATGGAGCCGTTCTCT
CCCATACATTGGCCTCCTGGGCTTCCCATCAATTGTGTCGAAATATTGTCTAATGTGAA
ATTTATTCATCGTGAGGTGTAAACCGCCCCCGCGTGACTGTAGAGCGTAATCCGGCCTT
CTGGGCTAGCAGCTAACGAGGAAGTGTGCTTCATTTCGTTCGGAAAGGACGAAATGCT
CTAGGCGGCTCTCAACCAGCCTAGTCCCCACGATACATACAACCGTACCAGGTCGAAT
TAATCCGCCGGGGTCCCGTCGCGGGATGTTTGAGGGGGAGATACGTTCCGGCCATATA
CTTTTTGTCCGCATCCCTTGGGGAAATCCATTACGCTGCCGTTGATGTCGTTAACGTCA
CGCCACTAACACCGAACTAGGGGGACCAAACTATACCATGTTATCCGTATACTTACCC
ACTGCCGTGGTTCGGAGTACGCTGCTGTGAGTAATGTACGCGCTCCTCGTGGGGATTTG
TCGTCTGAACTTGAATAACAGCTCACTCGGATGGGATTTAGCAAACCATCCCAACATAT
TCCAGGCCTCTGATCAGATCACAATTTTGCTGTCACCAGTTTCCAATTACGGTTGTGCA
TCGCCGGGAGGCTTGCGATTAGATCTACTCGCATGATTCGATCGGTAATATCGGGTCTG
AAATCTCCTGACCTAACGGGAGGTGGCACGCGCGATCGGAATCTGCAGTTTGCGGGAG
CTAATCGTATTCATGATGACTCCTTCACAAGTGAGTGGTGTGACGCGAGCAGCGGACC
AGATGATTTTCCGAATGTTCCCCTCCGTACCGAAGGCTTAACTTGAGGCGGAGCCGTGG
GCTCGCACTCCTGCTACGCAGGTGCACCGCGAGGCAACTTGAAGGTCTCGTCATGAAG

>WZS_WZS889.1

GTCGGGATGCCCTTTCGACACTGGCACGGCACGGCGCCTTCGCGCACGGATCGGATAA
ACTTTCCCGTGGACGGCTCGTCCTCAGAAACATTTCGTAGTCTTCTCGTTGGTTACTCCA
CTGCCGCGCCGCGTCTGCTTGGATGCTGAACCACGAGATTTATTAGAGTACTCTACTAC
TCGACCGGGGGCAGCATACAACCGTGGGGTCGGCGCGGCCCACCTCCGGCCAACGAG
AGTTACGAGTACCCGGCGCGTTTTCTCACGTCGGAGACTGTTAACACGATAGTAACTG
AAATGACCCAAACGTATTAATACGCACCGGGGGCGGGCCTCTGGCGCGCCACTGGATC
AGGCCCGTGGCGTGCCCGCGTCGTCAGCGCCACCCATTGCTAAGCGCTGACAGTAATA
GACCCCTCCATAGTATTTGCCGATGTTAATTCGGTCACCGGCCGAAACGTGCGCGCTCA
GTGCAGGGCAGGTCCTGTAGAACGGAGGATGGATATTTGGCAAAGGCTGCTGGTGCGC
CTACCAACTTGTTCCTCCGCGCCTGCTAGAGCGACCAACTACGCTCTGTAACGTCCAGT
ACCCGAGCAGTCTCTCAACTGGTCCGAGTAGACTGTATACACCGCCGTTGGGACGCGG
ACTAAACAACTCCCTCATACCCATCCGCCCGTCCGGAGCAGAACGACTTGGTGGTACC
GGCGTGAAGCGCCTCCGCCTAGGCTGCATCATTATGTTGGGAGATGCGTCAACTGGGG
GCCGGCAACCTGAGGTCGGCCGTTTCGTGAAGCCCATTAGTCCCATGGAGACGTCCTCT
CCAATCCATTGGCTTCCGGGGCTCTCCACCAATCGCACCGGAGTCTTGTCTAATGTGAA
ATTTATTCAACGTGAGGTATAAACCGCCCCCGCGTGGGTGCGGCTCGAAGTCTGGCCTC
CCAGGCTGCCTGCTAACACGGAAGTGTACCCGGTTCCACTCAGAGGGCATGAGGCAGT
ACCTACGGATCTCAACAAGCCTAGTTCGCGCAATACACATAACCATGCCCAATGAGAT
TAAGACGCCGGGGTTCCATCGCGGGATGTCTAGGGGGGAGATACGTTCCGGCCATATA
CTCTCTGTCCACATCCCTTGAGGAAATCCATTACGCTGCCGTTGACGTCGGTAACGCCG
CTTCATTAACGTCGAACTAGGGAGACCAAACCTTGCCATGTAATCCGTGTACTTACCCA
TTGTTGCGGTTTAGGGAACGGGGCTGTGGCTAATAGACGCGCTCCTGGTGGGAGCTTG
TCCTCCGGACTCGAATGGCTACTCGCCGGGCTGGGACTTAGCAAACCACTCTGACGCA
TCTACGGCCTCAGATTAGATCACAATTTTGCTTTCACCATTTCCCCATTACCGTTCCGCA

TCGTCGGGAGGTCTTCGACTAGATCTACTCGCATGATTCGAGCGGTAATATCGGGTCTG
AAATCTCCTGACCTAACGGGAGGTGGCACGCGCGATCGGAATCTGCACTTTGTGGGAG
CTAATCGTATTCATGATGACTCCTTCACAAGTGAGTGGTGTGACGCGAGCAGCGGACC
AGCTGATTTTCCGAATGTTCCCCTCCGTACCGAAGGCTTAACTTGAGGCGGAGCCGTGG
GCTCGCACTCCTGCTACGCAGGTGCACCGCGAGGCAACTTGAAGGTCTCGTCATGAAG

>WZS_WZS889.2

GTCGGGATGCCTTCCCAACTCTGGCACGGCGCGGCGCCTTCGCGCACGGATCGGATAA
GCTTACCCGTGGACGGCTCGTTCTCAGAAATATCTCGTAGTCTTCTCGTTGGTTACTCC
AATGAAACGCCTCGTCTGCGGGGGCACTGAACCGCGAGAGCTATTCAAGTGCTCTACT
ACTCGACCGGGGGCAGCTTACAACCGTGGGATCGGCGCGGCCCACCTCCGGTCAACGA
GAGTTACGAGTACCTGGTCCGTTTTCCCGCGTCGGAAGCTACCAACACGATAGTAACT
GAACCAGCGCGAGAACATTGATACGCCTCGGGGGCGGGCCTCTGGCGCGCCACTGGAT
CAGGCCCGTGGCGTGCCCGCCTCGTCAGCGCCACCCATTGCTAAGCGCTGACAGTAAT
AGACCCCTCCATAGTAGTTGCCGATGTTGATTCAGACACCGGCCGAAACGTGCGCGCT
CAGTGCAGGGCAGGTACTGTAGAGCGGAGGATGGATATTTGGCAAAGGCTGCTGGCGC
GCCTACCAACTTGTTCCTCCGCGCTTGCTGGAGCGACCAACTACGCTCTGTAGCGTCCA
GTACCCGAGCAGTCTCTCAACTGGTCCGATTCTATTGTATAAACAGTCGTTGGGACGCG
GACTAAACAACCTCCTCATACCCATCCGCCCGTCCGGAGCAGAACGACTCGGCGGTAC
CGGCGTCAGGCCCCCTCGCCTAGGCTGCACCATTATGTTGGGAGGTGCGTCGACTGGG
GGCCGGCGACTTGAGCTCGGTCGTCCCGTGAAGCCAATCAGTCCCATGGGGACATTCG
TTCCCGTCCATTGGCCTCTGGGGCTTTCCACCAATTGCACCAGAATAATGCCAGATGTT
AGATGTATTCATCGTGGGGCACGAGCCTTCCCCGCGTGAGTGCGGCTCGAAATCCGGT
CCCCCAGGCTGCCAGCTAACACGGAAGTGTGCCCGGTTCCACTCAGAGGGCATGAGGC
AGTACCTACGGATCTCAACAAGCCTAGTTCGTGCGATACACATAACCATGCCCGGTGG
GATTAAGACGCTGGGGTCTCATCGCGGGATGTTTGAGGGGGAGATACGTTCCGGTCAG
ATACTCTTTGTCCGCATCCCTTGGGGAAATCCATTACGCTGCCGTTGACGTCGTCAACG
TCACGCCACTAACACCGAACTAGGGGGACCAAACTATACCATGTTATCCGTATACTTA
CCCACTGTTGCAGGTCAAGGTATGGGGCTGCAAGTAATGTACATACTCCTGGTGGGAA
CTTGTCCTCCGGACTTGAATGGCTACCTGCCGGGCTGGGACTTAGCAAACCACCCTGAC
GCATCCCAGGCCTTTGATCGGATCACAATTTTGCTTTCACCAGTTCCCAATTACGGTTC
CGCATCGTCGGGAGGCCTTCGACTAGATCTACTCGCATGGTTCGAGCAGTAATATCGG
GTCTGAAATCCCCTGACCCAACGGGGGGCAGTATGTGTGGTCGGAATCTGCACCTTGC
GAGAGATAATCGTAATGACGATGGACCTTCTAGAAGTGAGTGCTGGGGCACGAGCGGC
TGACCAGATTGTCTTCCGAATGTTCCCCCCCGTGCCGAAGGCTGAACCCGAGGCGGAG
CCGTGGGCGTGCACCGTTGCCACGCAGGTGCACCGCGAGGCACCTTGAAGGTCCCGTC
ATGAAG

>WZS_WZS947.1

GTCGGGACGCCCTCCCGGCTCTGGCATGGCGCGGTGGCTTTGCGCACGGATCGGATAA
ACTTTCCATTGGACGGCTCGTTCTCAGAAATATCTCGTAGTCTTCTCGTTGGTTATTCCA
ATGAAGCGCCTCGTCTGTGGGGGCACTGAACCGCTAGAGCTATTCGAGTGCTCTACTA
CTCCACCGGGGGCAGCACGCAGCCGTGGGATCGGCACGGCCCACCTCCAGCCAACGAG
AGTTACGAGTACCCGACGCGTTTTCCCGCGTCGGAGGCTGCCAACACAATAGTAACTG
AACTGGCCCAAACGTATTAATACGCCCCGGGGGCGGGCCTCTGGCGCGCCACTGGATC
AGGCCCGTGGCGTGCCCGCCTCGTCAGCGCTACCCATTGCTAAGCGCTGACAGTAATA
GACCCCTCCATAGTAGTTGCCGATGTTGATTCGGTCACCGGCCGAAACGTGCGCGCTCA
GTGCAGGGCAGGTACTGTAGAGCGGAGGATGGATGATTGGCAGGGGTTGTTGGCACGC
CTACAAAACTATTTCTCCGCTCCCGCTGGAGCGACCAACTGCGCCTCGCAGCGACTGGT
ATCCGAACAGTCTATCAACTTGGCCGATTCTATTGTATAAACAGTCGTTGGGACGCGGA

CCAAACAACCCCCTCATACCCATCCGCCCGTCCGGAGTGGAACGACTTGGCGGTATTG
GCCTCAGGCCCACTCGCCTAGATTGCATCATTATGTTGGGAGGTACATCGACTGGGGG
CTGGCGACTTGATCCCGGTCGGCCCGTAAAGCCCATCAGTCCCATGGACACGTTCTCTC
CCATCCATTGGTCTCCTGGGCTTTCCACGAATTGCGTCGAAGTAATGCCAGATGTTAAG
CGTATTCATCATAGGGCACAAGCCGCGCCTTCGTGGGTACGGCTCGAAGCCCGACCCC
CCAGGCTGCCTAATGACACGGAAGTGTACCTGGTTCCACTCAAAAGGCATGAGGCAGT
CCCTACAGATCTTAGCAAGCTAAGTCCCCACGATACATACAACCGGGCCAGGTGGGAT
TAAGACGCCGGGGTCCCGTCGCGGGACATCTGGGATGGGAACACGTTCCGGTCATATA
CTCTTTGTCCGCATCCCTTGGGGAAATCCATTACGCTGTCATTGACGTCGTCAACGTCA
TGCCACTAACACCGAACTAGGGGGACCAAACTATACCATGTTATCCGTATGCCTACTC
GTTGTCGCGGTTCAGAGTACGGGCCTGTGAGTAATGTACGCGCTCCTGGTGGGAACTT
GTCCTCCGGACTTGAATGGTTACTCGCCGAGCTGGAACTTAGCAAACCACCCCAACAT
ATTCCAGGCCTCTGATCAGATCACAATTTTGCTTTCACCAGTTCCCAATTACGGTTCCG
CATCGTCGGGAGGCCTTCGACTAAATCTACTCGCAAGGTTCGAGCGGTAATATCGGGT
CTGAAATCCCCTGACCCAACGGGAGGCGGTACGTGTGATCGGAAGCTGCACCTCGCGA
GAGCCAAGCGGAATGACGATGGCTCTTCCACAAGTGAGTGGTGTAGTGCCAGCGGCGG
ACCAGATGGTCTTCCGAATGTTCCCCCCCGTGCCGAAGGCTGAATTCGAGATGGAGCA
GTGGGCTCGCACCGTTGCCACGCAGGTGCACCGCGAGGCAACTTGAAGGTCCAGTCAT
GAAG

>WZS_WZS947.2

GTCGGGATGCCTTCCCAACTCTGGCACGGCGCGGCGCCTTCGCGCACGGATCGGATAA
GCTTTCCCGCGGACGGCTCGTCCTCGGAAATATCTCGTAGTCTTCTCGTTGGTTGCTCC
AACGAAGCGACGAATCTACTTGGATACTGAACCGCGAGAGCTATTCAAGTGCTCTACT
ACTCGACCGGGGGAAGCATACAGCCGTGGGATCGGCGCGGCCCACCTCCGGCCAACG
AGAGTTACGAGTACCCGGCCCGTTTTCCCGCGTCGGAGGCTGCCAACACGATAGTAAC
TGAACTGGCCCAAACGTATTGATACGCCCCGGGGGCGGGCCTCTGGCGCGCCACTGGA
TCAGGCCCGTGGCGTGCCCGCCTCGTCAGCGCCACCCATTGCTAAGCGCTGACAGTAA
TAGACCCCTCCATAGTAGTTGCCGATGTTAATTCGGTCACCGGCCGAAACGTATGAAC
GCAGCACAGGGCAGGTACTACGGAGCGAAAGGTAGCTGATTGGCAGGGGCTGCTGGC
GCGCCTACCAACCTGTTCCTCCGCGCCTGCTGGAGCGACCAACTACGCCCCGCAGCGA
CCGGTACTTGAGCTGTCTCTCAACTGGTTCGATGAGACTGTATACACCGTCGTTGGGAC
GCGGACTAAACAACCTCCTCATACCAATCCGCCCGTCCGGAGCGGAACGACTCGGCGG
TACCGGCGTCAGGCCCCCTCGCCTAGGCTGCACCATTATGTTGGGAGGTGCGTCGACTG
GGGGCCGGCGACTTGAGCTCGGTCGTCCCGTGAAGCCCATCAGTCCCATGGAGACGTT
CTCTCCCATCCATTAGCCTCCGGGGCTCTCCACCAATCGCACCGGAGTCTTGTCTAGTG
TTAAATGTATTCATCGTGGGGTATAAACCGCCCCCGCGTGGGTGCGGCTCGAAGTCTG
GCCTCCCAGGCTGCTAGCTAACACGGAAGTGTGCCTCGTTTCGCTCAGAGGGGACGAG
ACACTCTCGACGGCTCTCAACAAGCCTAGTCCCCACGATACATACAACCGTGCCCGGT
GGGGTTAATACACCGGGGTCCCGTCGCGGGACGTCTGGGATGGACCATGTTCCGGCC
GTATACTCCCTGTTCGTGCCCCTCGGAGAAATCCATTACGCTGCCGTTGACGTCGTTAA
CGTCACGCCACTAACACCGAACTAGGGGGACCAAACTATACCATGTAATCCGTGCGCC
TACCCACTGTTGCGATTCAGGGTACGGGGCTGTGAGTAATGTACACACTCCTGGTGTGA
GCTTGTCCTCCGGACTTGAATGGCTACTCGCCGGGCTGGGACTTAGCAAACCACCCTGA
CGCATTCCAGGCCTCTGATCAGATCACAATTTTGCTTTCACCAGTTCCCAATTACGGTT
CCGCATCGTCGGGAGGCCTTCGACTAGATCTACTCGCATGGTTCGAGCGGTAATATCG
GGTCTGAAATCCCCTGACCCAACGGGAGGCGGTACGTGTGATCAGGATCTGCACCTTG
CGAGAGCCAAGCGTAATGACGGTGGCTCTTCCACAAGTAAAAGCTCGGGCGCGAGCG
GCGGGCTGGATGGTCCCCCGAATGTTCCCCCCTATGCCGAAGGCTGAACTCGAGGCTG
AGCCGTGGGCGCGCACCGTTGCCACGCAGGTGCACCGCGAGGTCCCTTGAAGGTCCCG
TCACGAAG

>WZS_WZS955.1

GTCGGGATGCCTTCCCAACTCCGGCACGGCGCGGCGCCTTCGCGCACGGATCGGATAA
GCTTACCCGTGGACGGCTCGTTCTCAGAAATATCTCGTAGTCTTCTCGTTGGTTACTCC
AATGAAACGCCTCGTCTGCGGGGGCACTGAACCGCGAGAGCTATTCAAGTGCTCTACT
ACTCGACCGGGGGCAGCTTACAACCGTGGGATCGGCGCGGCCCACCTCCGGTCAACGA
GAGTTACGAGTACCTGGTCCGTTTTCCCGCGTCGGAAGCTACCAACACGATAGTAACT
GAACCAGCGCGAGAACATTGATACGCCTCGGGGGCGGGCCTCTGGCGCGCCACTGGAT
CAGGCCCGTGGCGTGCCCGCCTCGTCAGCGCCACCCATTACTAAGCGCTGACAGTAAT
AGACCCCTCCATAGTAGTTGCCGATTTTGATTCGGTCACCGGCCGAAACGTATGCACTC
AGCACAGGGCAGGTACCACGGAGCGAAAGGTGGATGATTGGCAGGGGCCGCTTGCGC
ACCTACATAACTATTCGTCCGCGCCTGCTGGAGCGACCAACTACGCTCTATAGCGTTCA
GTACCCGAGCAGTCTCCTAACTGGTCCGGTGAGACTGTAAACATCCTTGTTGGGTCGTT
GCATAAACAACCCACTCTTACCCATCCGTCCGTCCGGAGCAGAACGACTCGGCGGTAC
CGGCGTCAGGCCCCCTCGCCTAGACTGCACCATTATGTTGGGAGGTGCGTCGACTGGG
GGCTGGCAACCTGAGGTCGGCCGTTTCGTGAAGCCCATTAGTCCCATGGACACGTTCTC
TCCCATACATTGGCCTCCTGGGCTTTCCACCAATTGCGTCGAAGTATTTTCTAATGTTAA
GTGTGTTTGTCGCGGGACACAAACTGCCCCCGCGCGGGTGCGGAGCGTAATCCGGCCC
CCCAGGCCGCCTGCTAACACGGAAGTGTACCCGGTTCCACTCAGAGGGCATGAGGCAG
TACCTACGGATCTCAACAAGCCTAGTCCCCATGATACGTACAGCTGGGCCAGGTGGGA
TTAATACGCCGGGGTCCCGTCGCGGGATGTTTGAGGGGGAGATACATTCCGGCCGTAT
ACTCCCTGTCCGCATCCCTTGGGGTAATCCATTACGCCGTCGTTGACGTCATCAATGCC
GCGCGATTAACACCGAGCCAATGGGACCAAAACATACCATGTAATCCGTGTACTTACC
CATCGTTGCGGTACAGGATACGGGGCTGTGAGTAATGTACACACTCCTGGAGGGAGCT
TGTCCTCCAGACTTGAATGGCTACCTGCCGGGCTGGGACTCAGCAAACCACCCTGACG
CATCCCAGGCCTTTGATCGGATCACAATTTTGCTTTCACCAGTTCCCCATTACGGTTCCG
CATCGTCGGGAGGTCTTCGACTAGATCTACTCGCATGGTTCGAGCAGTAATATCGGGTC
TGAAATCCCCTGACCCAACGGGGGGCAGTATGTGTGGTCGGAATCTGCACCTTGCGAG
AGATAATCGTAATGACGATGGACCTTCTACAAGTGAGTGCTGGGGCACGAGCGGCGGA
CCAGATTGTCTTCCGAATGTTCCCCCCCGTGCCGAAGGCTGCACCCGAGGCGGAGCCG
TGGGCGTGCACCGTTGCCACGCAGGTGCACCGCGAGGCACCTTGAAGGTCCCGTCATG
AAG

>WZS_WZS955.2

GTCGGGATGCCTTCCCAACTCTGGCACGGCGCGGCGCCTTCGCGCACGGATCGGATAA
GCTTTCCCGTGGACGGCTCGTCCTCGGAAATATCTCGTAGTCTTCTCGTTGGTTGCTCCA
ACGAAGCGACGAATCTACTTGGATACTGAACCGCGAGAGCTATTCAAGTGCTCTACTA
CTCGACCGGGGGCAGCATACAGCCGTGGGATCGGCGCGGCCCACCTCCGGCCAACGAG
AGTTACGAGTACCCGGCCCGTTTTCCCGCGTCGGAGGCTGCCAACACGATAGTAACTG
AACTGGCCCAAACGTATTGATACGCCCCGGGGGCGGGCCTCTGGCGCGCCACTGGATC
AGGCCCGTGGCGTGCCCGCCTCGTCAGCGCCACCCATTGCTAAGCGCTGACAGTAATA
GACCCCTCCATAGTAGTTGCCGATGTTAATTCGGTCACCGGCCGAAACGTATGAACGC
AGCACAGGGCAGGTACTACGGAGCGAAAGGTAGCTGATTGGCAGGGGCTGCTGGCGC
GCCTACCAACCTGTTCCTCCGCGCCTGCTGGAGCGACCAACTACGCCCCGCAGCGACC
GGTACTTGAGCTGTCTCTCAACTGGTTCGATGAGACTGTATACACCGTCGTTGGGACGC
GGACTAAACAACCTCCTCATACCAATCCGCCCGTCCGGAGCGGAACGACTCGGCGGTA
CCGGCGTCAGGCCCCCTCGCCTAGGCTGCACCATTATGTTGGGAGGTGCGTCGACTGG
GGGCCGGCGACTTGAGCTCGGTCGTCCCGTGAAGCCCATCAGTCCCATGGAGACGTTC
TCTCCCATCCATTAGCCTCCGGGGCTCTCCACCAATCGCACCGGAGTCTTGTCTAGTGT
TAAATGTATTCATCGTGGGGTATAAACCGCCCCCGCGTGGGTGCGGCTCGAAGTCTGG
CCTCCCAGGCTGCTAGCTAACACGGAAGTGTGCCTCGTTTCGCTCAGAGGGGACGAGA

CACTCTCGACGGCTCTCAACAAGCCTAGTCCCCACGATACATACAACCGTGCCCGGTG
GGATTAATACACCGGGGTCCCGTCGCGGGACGTCTGGGATGGGACCATGTTCCGGCCG
TATACTCCCTGTTCGTGCCCCTCGGAGAAATCCATTACGCTGCCGTTGACGTCGTTAAC
GTCACGCCACTAACACCGAACTAGGGGGACCAAACTATACCATGTAATCCGTGCGCCT
ACCCACTGTTGCGATTCAGGGTACGGGGCTGTGAGTAATGTACACACTCCTGGTGTGA
GCTTGTCCTCCGGACTTGAATGGCTACTCGCCGGGCTGGGACTTAGCAAACCACCCTGA
CGCATTCCAGGCCTCTGATCAGATCACAATTTTGCTTTCACCAGTTCCCAATTACGGTT
CCGCATCGTCGGGAGGCCTTCGACTAGATCTACTCGCATGGTTCGAGCGGTAATATCG
GGTCTGAAATCCCCTGACCCAACGGGAGGCGGTACGTGTGATCAGGATCTGCACCTTG
CGAGAGCCAAGCGTAATGACGGTGGCTCTTCCACAAGTAAAAGCTCGGGCGCGAGCG
GCGGGCTGGATGGTCCCCCGAATGTTCCCCCCTATGCCGAAGGCTGAACTCGAGGCTG
AGCCGTGGGCGCGCACCGTTGCCACGCAGGTGCACCGCGAGGTCCCTTGAAGGTCCCG
TCACGAAG

>WZS_WZS973.1

GTCGGGACGCCCTCCCGGCTCTGGCATGGCGCGGTGGCTTTGCGCACGGATCGGATAA
ACTTTCCATTGGACGGCTCGTTCTCAGAAATATCTCGTAGTCTTCTCGTTGGTTACTCCA
ATGAAACGCCTCGTCTGCGGGGGCACTGAACCGCGAGAGCTATTCAAGTGCTCTACTA
CTCGACCGGGGGCAGCTTACAACCGTGGGATCGGCGCGGCCCACCTCCGGTCAACGAG
AGTTACGAGTACCTGGTCCGTTTTCCCGCGTCGGAAGCTACCAACACGATAGTAACTG
AACCAGCGCGAGAGTATTGATACGCCTCGGGGGCGGGCCTCTGGCGCGCCACTGGATC
AGGCCCGTGGCGTGCCCGCCTCGTCAGCGCCACCCATTGCTAAGCGCTGACAGTAATA
GACCCCTCCATAGTAGTTGCCGATGTTGATTCGGTCACCAACCGAAACGTGCGCGCTCA
GCGCAGGGCAGATACTGTAGAACGGAGGATGGATGATTGGCAGGGGTTGTTGGCACG
CCTACAAAACTATTTCTCCGCTCCCGCTGGAGCGACCAACTGCGCCTCGCAGCGACTGG
TATCCGAACAGTCTATCAACTTGGCCGATTCTATTGTATAAACAGTCTTTGGGACGCGG
AACAAACAACCCCCTCATACCCATCCGCCCGTCCGGAGTGGAACGACTTGGCGGTATT
GGCCTCAGGCCCACTCGCCTAGATTGCATCATTATGTTGGGAGGTACATCGACTGGGG
GCTGGCGACTTGATCCCGGTCGGCCCGTAAAGCCCATCAGTCCCATGGAGACGTTCTCT
CCCATCCATTGGCCTCTGGGGCTTTCCATCAATTGCATCGAAGTATTGCCTAGGGTGAG
ATGTATTCATCGTGGGGCACAAGCCGCCCCCGCGTGAGTGCGGCTCGAAGTCTGGCCC
CCCAGGCTGCCTGCTAACACGGAAGTGTACCCGGTTCCACTCAGAGGGCATGAGGCAG
TACCTACGGATCTCAACAAGCCTAGTTCGCGCGATACACATAACCATGCCCGGTGGGA
TTAAGACGCTGGGGTCTCATCGCGGGATGTTTGAGGGGGAGATACGTTCCGGTCATAT
ACTCTTTGTCCGCATCCCTTGGGGAAATCCATTACGCTGCCGTTGACGTCGTTAACGTC
ATGCCACTAACACCGAACTAGGGGGACCAAACTATACCATGCTATCCGTATGCCTACT
CGTTGTCGCGGTTCAGAGTACGGGGCTGTGAGTAATGTACGCATTCCTGGTGGGAACTT
GTCCTCTGAACTTGAATAACAGCTCACTGGAATGGGATTTAGCAAACCATCCCAACAT
ATTCCAGGCCTCTGATCAGATCACAATTTTGCTGTCACCAGTTCCCAATTACGGTTCCG
CATCGTCGGGAGGCCTTCGACTAGATCTACTCGCATGGTTCGAGTGGTAATATCGGGTC
TGAAATCCCCTGACCCAACGGGAGGCGGTATACGCAATCAGGATCTGCACTTTTCGGG
AGCTAATCGTAATGACGATGGACCTTCTAGAAGTGAGTGCTGGGGCGCGAACAGCGGA
CCAGACGGTCTTCCGAATGTTCCCCCCCGTGTCGAACGATGAATTCGAGGCGGAGGAA
TGGGCGCGCACCGTTGCCACGCAGGTACACCGCGAGGTCCCTTGAAGATCCCGTCATC
AAC

>WZS_WZS973.2

GTCGGGATGCCTTCCCAACTCCGGCACGGCGCGGCGCCTTCGCGCACGGATCGGATAA
GCTTACCCGTGGACGGCTCGTTCTCAGAAATATCTCGTAGTCTTCTCGTTGGTTATTCCA
ATGAAGCGCCTCGTCTGTGGGGGCACTGAACCGCTAGAGCTATTCGAGTGCTCTACTA

CTCCACCGGGGGCAGCACGCAGCCGTGGGATCGGCACGGCCCACCTCCAGCCAACGAG
AGTTACGAGTACCCGACGCGTTTTCCCGCGTCGGAGGCTGCCCACACAATAGTAACTG
AACTGGCCCAAACGTATTAATACGCCCCGGGGGCGGGCCTCTGGCGCGCCACTGGATC
AGGCCCGTGGCGTGCCCGCCTCGTCAGCGCCACCCATTGCTAAGCGCTGACAGTAATA
GACCCCTCCATAGTAGTTGCCGATGTTGATTCGGTCACCGGCCGAAACGTGCGCGCTCA
GTGCAGGGCAGGTACTGTAGAGCGGAGGATGGATATTTGGCAAAGGCTGCTGGCGCGC
CTACCAACTTGTTCCTCCGCGCTTGCTGGAGCGACCAACTACGCTCTGTAGCGTCCAGC
ACCCGAGCAGTCTCCCAACCGGTCCGGTGAGACTGTAAACATCCTTGTTGGGTTGTTGC
ATAAACAACCCACTCTTACCCATCCGTCCGTCCGGAGCAGACCGGTTTGGTGGTACCA
GCGTCAGGCCCCTTCGCCTAGGCTGCACCATTATATTGGGAAACGCGACGGCCAGAGG
CCGGCGACCTAATCTCAGCTATTCTTCGGAGCCCATCAGTCCCATGGAGACGTTCTCTC
CCATCTATTGGCCTCCGGGGCTCTCCACCAATCGCACCGGAGTCTTGTCTAATATGAAA
TTTATTCATCGTGAGGTATAAACCGCCCCCGAGTGGGTGCGGCTTGAAGTCCAGCCCCC
CAGGCCGCCTGCTAACACGGAAGTGTACCCGGTTCCACTCAGAGGGCATGAGGCAGTA
CCTACGGATCTCGACAAGCCTAGTCCCCATGATACGTACAGCTGGGCCAGGTGGGACT
AATACGCCGGGGTTCCGTTGCGGAACGGCTAGAAGGTAAATACGCCTTGGCCATATAC
TCTTTGTCCGCATCCCTTGGGGAAATCCATTATGCTGCCGTTGACGTCGTCAACGTCAC
GCCACTAACACCGAACTAGGGGGACCAAACTATACCATGTTATCCGTATACTTACCCA
CTGTTGCAGGTCAAGGTATGGGGCTGCAAGTAATGTACATACTCCTGGTGGGAACTTG
TCCTCCGGACTTGAATGGCTACCTGCCGGGCTGGGACTTAGCAAACCACCCTGACGCA
TCCCAGGCCTTTGATCGGATCACAATTTTGCTTTCACCAGTTCCCAATTACGGTTCCGC
ATCGTCGGGAGGCCTTCGACCGGATCTACTCGCATGGTTCGAGCAGTAATATCGGGTCT
GAAATCCCCTGACCCAACGGGGGGCAGTATGTGTGGTCGGAATCTGCACCTTGCGAGC
GATAATCGTAATGACGATGGACCTTCTACAAGTGAGTGCTGGGGCACGAGCGGCTGAC
CAGATTGTCTTCCGAATGTCCCCCCCCGTGCCGAAGGCTGAACCCGAGGCGGAGCCGT
GGGCGTGCACCGTTGCCACGCAGGTGCACCGCGAGGCACCTTGAAGGTCCCGTCATGA
AG

>TT_B12.1

GTCGGGACGCCCTCCCGGCTCTGGCACGGCGCGGTGGCTTTGCGCACGGATCGGATAA
ACTTTCCATTGGACGGCTCGTTCTCAGAAATATCTCGTAGTCTTCTCGTTGGTTACTCCA
ATGAAGCGCCTCGTCTGCGGGGGCACTGAACCGCGAGAGCTATTCAAGTGCTCTACTA
CTCGACCGGGGGCAGCTTACAACCGTGGGATCGGCGCGGCCCACCTCCGGTCAACGAG
AGTTACGAGTTCCCGGCCCGTTTTCTCGCGTCGGAGGCTGCCAACACGATAGTAACTGA
ACTGGCCCAAACGTATTAATACGCCCCGGGGGCGGGCCTCTGGCGCGCCACTGGATCA
GGCCCGTGGCGTGCCCGCCTCGTCAGCGCCACCCATTGCTAAGCGCTGACAGTAATAG
ACCCCTCCATAGTAGTTGCCGATGTTGATTTGGTCACCGGCCGAAACGTATGCGCTCAG
CACAGGGCAGGTACTACGGAGCGAAAGGTGGATGATTGGCAGGGGCCGCTGGCGCAC
CTACAAAACTATTCGTCCGCGCCTGCTGGAGCGACCAACTACGCTCTATAGCGTCCAGT
ACCCGAGCAGTCCCTCAATTGGTCCGATGAGACGGTATACGCCGCCGTTGGGACGCGG
ACTAAACAACCCCCTCATACCCATTCGCCCGTCCAGAGCGGAACGACTCCGCGGTGCC
GGCGTCAGGCCTCCTCGCCTAGGCTGCGCCATTATGTTGGGAGGTGCGTTGACTGGGG
GCCGGCGACCTGAGCTTGGTCGGCCCGTGAAGCCCATCACTCCCATGGAGACGTTTTCT
CCCACCCATTGGCCCCCGGGGTCCTCCACCAATTGCGTCGGAGTCTTGTCTAATATGAA
ATTTATTCATCGTAAGGTATAAACCGCCCCCGCGTGGGTGCGGCTCGAAGTCCGGCTTC
CCAAGCTGGCTGCTAACACGGAAGTGTACCCGGTTCCACTCAGAGGGCCTGAGGCAGT
ACCTACGGATCTCAAAAACCCTAGTCCCCATGATACGTACAGCTGGGCTAGGTGGGAT
TAATACGTCGGGGTCCCATCGCGGGATGTTTGAGGGGGAGATACGTTCCAGCCATATA
CTCCCTGATCGCGCCCCTCAGAGAAGTCAATCACGCTGCCGTTGACGTCGTCAATGCCG
CGCCACTAACACCGGACTAGGGGGACCAAACCATACCATGTAATACGTGTACTTGCCC
ATTGTCGCGGTTCAGAGTACGGGGCTGTGAGTAATGTACGCGTTCCTGGTGGGAACTT

GTCCTCTGAACTTGAATAACAGCTCACTGGAATGGGATTTAGCAAACCATCCCAACAT
ATTCCAGGCCTCTGATCAGATCACAATTTTGCTTTCACCAGTTCCCAATTATGATTCCGC
ATCGTCGGGAGGCCTTCGACTAGATCTGCTCGCATGGTTCGAGCGGTAATATCGGGTCT
GAAATCCCCTGACCCAACGGGAGGCGGTATGCGTGATCGGAATCTGCACCTTGCGAGA
GCTAATCGTGATGACAATGGCTCTTCCACAAGTGAGTGCGGGGGCGCGAGCGGCGAAC
CAGCTGGTCCTCTGAATATTCCCCCCCGTGCCGAAGGCTGAACTCGAGGCGGGGCCGT
GGTCGCGCACCGTTACCACGCAGGTGCGTCGCGAGGCCCCTTGAAGGTCCCGTCATGA
AG

>TT_B12.2

GTCGGGATGCCTTCCCAACTCTGGCACGGCGCGGCGCCTTCGCGCACGGATCGGATAA
GCTTTCCCGTGGACGGCTCGTCCTCGGAAATATCTCGTAGTCTTCTCGTTGGTTGCTCCA
ACGAAGCGACGAATCTACTTGGATACTGAACCGCGAGAGCTATTCAAGTGCTCTACTA
CTCGACCGGGGGCAGCATACAGCCGTGGGATCGGCGCGGCCCACCTCCGGCCAACGAG
AGTTACGAGTACCCGGCCCGTTTTCCCGCGTCGGAGGCTGCCAACACGATAGTAACTG
AACTGGCCCAAACGTATTGATACGCCCCGGGGGCGGGCCTCTGGCGCGCCACTGGATC
AGGCCCGTGGCGTGCCCGCCTCGTCAGCGCCACCCGTTGCTAAGCGCTGACAGTAATA
GACCCCTCCATAGTAGTTGCCGATGTAGGTCTGGTCACCGGCCGAAACGTATGAACGC
AGCACAGGGCAGGTACTACGGAGCGAAAGGTAGCTGATTGGCAGGGGCTGCTGGCGC
GCCTACCAACCTGTTCCTCCGCGCCTGCTGGAGCGACCAACTACGCCCCGCAGCGACC
GGTCCCCGAGCAGTCTCTCAACTGGTTCGATGAGACTGTATACACCGTCGTTGGGACGC
GGACTAAACCGCCCCCTCATACCCACCCGCCCGTCCGGAGCGGAACGACTCGGCGGTA
CCGGCGTCAGGCCCCCTCGCCTAGGCTGCACCATTATGTTGGGAGGTGCGTCGACTGG
GGGCCGGCGACTTGAGCTCGGTCGTCCCGTGAAGCCCATCAGTCCCATGGAGACGTTC
TCTCCCATCCATTGGCCTCCGGGGCTCTCCACCAATCGCACCGGAGTCTTGTCTAGTGT
TAAATGTATTCATCGTGGGGTATAAACCGCCCCCGCGTAAGTGCGGCTCGAAGTCTGG
CCTCCCAGGCTGCTAGCAAACACGGAAGTGCGCCTCGTCTCGCTCAGAGGGGACAAGA
CACTCTCGACGGCTCTCAACAAGCCTAGTCCCCACGATACATACAACCGTGCCCGGTG
GGATTAATACGCCGGGGTCCCGTCGCGGGACATCTGGGATGGGAACACGTTCCGACCA
TATGCTCTCTGTCTGCATCTCTTGGGGAAATCCATTACGCTGCCGTTGACGTCGTTAAC
GTCACGCCACTAATACCGAACTAGGGGGGCCAAGCTATACCATGTAATCCGTGCGCCT
ACCCACTGTTGCGATTCAGGGTACGGGGCTGTGAGTAATGTACACACTCCTGGTGTGA
GCTTGTCCTCCGGACTTGAATGGCTACTCGCCGGGCTGGGACTTAGCAAACCACCCTGA
CGCATTCCAGGCCTCTGATCAGATCACAATTTTGCTTTCACCAGTTCCCAATTACGGTT
CCGCATCGTCGGGAGGCCTTCGACTAGATCTACTCGCATGGTTCGAGCGGTAATATCG
GGGCTGAAATCCCCTGACCCAACGGGAGGCGGTACGTGTGATCAGGATCTGCACCTTG
CGAGAGCCAAGCGTAATGACGGTGGCTCTTCCACAAGTAAAAGCTCGGGCGCGAGCG
GCGGGCTGGATGGTCCCCCGAATGTTCCCCCCTATGCCGAAGGCTGAACTCGAGGCTG
AGCCGTGGGCGCGCACCGTTGCCACGCTGGTGCACCGCGAGGTCCCTTGAAGGTCCCG
TCACGAAG

>TT_B26.1

GTCGGGATGCCTTCCCAACTCCGGCACGGCGCGGCGCCTTCGCGCACGGATCGGATAA
GCCTTCCCGTGGACGGCTTGTCTTCAAAAATACCTCGTAGCCTTCTCGTTGTTTACCCCA
ATGCCGCGCCGCGTCTGCTTAGATGCTGAATTGCGGAAGCTATTCGAGAGTCCTGTTGC
CCGGCCGGGGGCAGCACACAACCATGGGATCGGCGCAGCCCACCTCCGGCCAACGAG
AGTTACGGCGTCCCGGCCCGTTTTCCCGCGTCGGAGGCTGCCAACACGATAGTAACTG
AAATGGCCCAAACGTATTAATACGCACAGGGGGCGGGCCTCTGGCATGTCACTGGATC
AGGCCCGTGGCGTGCCCGCCTCGTCAGCGCCACCCATTGCTAAGCGCTGACAGTAATA
GACCCCTCCATAGTAGTTGCCGATGTTGATTTGGTCACCGGCCGAAACGTATGCGCTCA

GCACAGGGCAGGTACTACGGAGCGAAAGGTGGATGATTGGCAGGGGCCGCTGGCGCA
CCTACAAAACTATTCGTCCGCGCCTGCTGGAGCGACCAACTACGCTCTATAGCGTCCAG
TACCCGAGCAGTCCCTCAATTGGTCCGATGAGACTGTATACGCCGCCGTTGGGACGCG
GACTAAACAACCCCCTCATACCCATTCGCCCGTCCAGAGCGGAACGACTCCGCGGTGC
CGGCGTCAGGCCTCCTCGCCTAGGCTGCGCCATTATGTTGGGAGGTGCGTTGACTGGG
GGCCGGCGACCTGAGCTTGGTCGGCCCGTGAAGCCCATCACTCCCATGGAGACGTTTT
CTCCCACCCATTGGCCCCCGGGGTCCTCCACCAATTGCGTCGGAGTCTTGTCTAATATG
AAATTTATTCATCGTAAGGTATAAACCGCCCCCGCGTGGGTGCGGCTCGAAGTCCGGC
TTCCCAAGCTGGCTGCTAACACGGAAGTGTACCCGGTTCCACTCAGAGGGCCTGAGGC
AGTACCTACGGATCTCAAAATCCCTAGTCCCCATGATACGTACAGCTGGGCTAGGTGG
GATTAATACGTCGGGGTCCCATCGCGGGATGTTTGAGGGGGAGATACGTTCCAGCCAT
ATACTCCCTGTTCGCGCCCCTCAGAGAAGTCCATTACGCTGCCGTTGACGTCGTCAATG
CCGCGCCACTAACACCGGACTAGGGGGACCAAACCATACCATGTAATACGTGTACTTG
CCCATTGTCGCGGTTCAGAGTACGGGGCTGTGAGTAATGTACGCGTTCCTGGTGGGAA
CTTGTCCTCTGAACTTGAATAACAGCTCACTGGAATGGGATTTAGCAAACCATCCCAAC
ATATTCCAGGCCTCTGATCAGATCACAATTTTGCTTTCACCAGTTCCCAATTATGATTCC
GCATCGTCGGGAGGCCTTCGACTAGATCTGCTCGCATGGTTCGAGCGGTAATATCGGG
TCTGAAATCCCCTGACCCAACGGGAGGCGGTATGCGTGATCGGAATCTGCACCTTGCG
AGAGCTAATCGTGATGACAATGGCTCTTCCACAAGTGAGTGCGGGGGCGCGAGCGGCG
AACCAGCTGGTCCTCTGAATATTCCCCCCCGTGCCGAAGGCTGAACTCGAGGCGGGGC
CGTGGTCGCGCACCGTTACCACGCAGGTGCGTCGCGAGGCCCCTTGAAGGTCCCGTCA
TGAAG

>TT_B26.2

GTCGGGATGCCTTCCCAACTCTGGCACGGCGCGGCGCCTTCGCGCACGGATCGGATAA
GCTTTCCCGTGGACGGCTCGTCCTCGGAAATATCTCGTAGTCTTCTCGTTGGTTGCTCCA
ACGAAGCGACGAATCTACTTGGATACTGAACCGCGAGAGCTATTCAAGTGCTCTACTA
CTCGACCGGGGGAAGCATACAGCCGTGGGATCGGCGCGGCCCACCTCCGGCCAACGA
GAGTTACGAGTACCCGGCCCGTTTTCCCGCGTCGGAGGCTGCCAACACGATAGTAACT
GAACTGGCCCAAACGTATTGATACGCCCCGGGGGCGGGCCTCTGGCGCGCCACTGGAT
CAGGCCCGTGGCGTGCCCGCCTCGTCAGCGCCACCCATTGCTAAGCGCTGACAGTAAT
AGACCCCTCCATAGTAGTTGCCGATGTTAATTCGGTCACCGGCCGAAACGTATGAACG
CAGCACAGGGCAGGTACTACGGAGCGAAAGGTAGCTGATTGGCAGGGGCTGCTGGCG
CGCCTACCAACCTGTTCCTCCGCGCCTGCTGGAGCGACCAACTACGCCCCGCAGCGAC
CGGTACTTGAGCTGTCTCTCAACTGGTTCGATGAGACTGTATACACCGTCGTTGGGACG
CGGACTAAACAACCTCCTCATACCAATCCGCCCGTCCGGAGCGGAACGACTCGGCGGT
ACCGGCGTCAGGCCCCCTCGCCTAGGCTGCACCATTATGTTGGGAGGTGCGTCGACTG
GGGGCCGGCGACTTGAGCTCGGTCGTCCCGTGAAGCCCATCAGTCCCATGGAGACGTT
CTCTCCCATCCATTAGCCTCCGGGGCTCTCCACCAATCGCACCGGAGTCTTGTCTAGTG
TTAAATGTATTCATCGTGGGGTATAAACCGCCCCCGCGTGGGTGCGGCTCGAAGTCTG
GCCTCCCAGGCTGCTAGCTAACACGGAAGTGTGCCTCGTTTCGCTCAGAGGGGACGAG
ACACTCTCGACGGCTCTCAACAAGCCTAGTCCCCACGATACATACAACCGTGCCCGGT
GGGATTAATACACCGGGGTCCCGTCGCGGGACGTCTGGGATGGGACCATGTTCCGGCC
GTATACTCCCTGTTCGTGCCCCTCGGAGAAATCCATTACGCTGCCGTTGACGTCGTTAA
CGTCACGCCACTAACACCGAACTAGGGGGACCAAACTATACCATGTAATCCGTGCGCC
TACCCACTGTTGCGATTCAGGGTACGGGGCTGTGAGTAATGTACACACTCCTGGTGTGA
GCTTGTCCTCCGGACTTGAATGGCTACTCGCCGGGCTGGGACTTAGCAAACCACCCTGA
CGCATTCCAGGCCTCTGATCAGATCACAATTTTGCTTTCACCAGTTCCCAATTACGGTT
CCGCATCGTCGGGAGGCCTTCGACTAGATCTACTCGCATGGTTCGAGCGGTAATATCG
GGTCTGAAATCCCCTGACCCAACGGGAGGCGGTACGTGTGATCAGGATCTGCACCTTG
CGAGAGCCAAGCGTAATGACGGTGGCTCTTCCACAAGTAAAAGCTCGGGCGCGAGCG

GCGGGCTGGATGGTCCCCGAATGTTCCCCCTATGCCGAAGGCTGAACTCGAGGCTG
AGCCGTGGGCGCGCACCGTTGCCACGCAGGTGCACCGCGAGGTCCCTTGAAGGTCCCG
TCACGAAG

>TT_B40.1

GTCGGGATGCCCTCTCGATACTGGCACGGAGCGGCGCCTTCGCGCACGGATCGGATAA
GCTTTCCCGTAGATGGCTCGTCCTCGGAAATATCTCATAGTCTTCTCGTTGGTTACTCCA
ATGAAGCGCCTCGTCTGCGGGGGCACTAAACCGCGAGAGCTATTCAAGTGCTCTACTA
CTCGACCGGGGGCAGCTTACAACCGTGGGATCGGCGCGGCCCACCTCCGGTCAACGAG
AGTTACGAGTTCCCCGCCCGTTTTCCCGCGTCGGAGGTTGCCAACAGGATAGTAACTGA
ACTGGCCCAAACGTATTAATACGCCCCGGGGGCGGGCCTCTGGCGCGCCACTGGATCA
GGCCTGTGGCGTGCCCGCCTCGTCAGCGCCACCCATTGCTAAGCGCTGACAGTAATAG
ACCCCTCCATAGTAGTTGCCGATGTTGATTTGGTCACCGGCCGAAACGTATGCGCTCAG
CACAGGGCAGGTACTACGGAGCGAAAGGTGGATGATTGGCAGGGGCCGCTGGCGCAC
CTACAAAACTATTCGTCCGCGCCTGCTGGAGCGACCAACTACGCTCTATAGCGTCCAGT
ACCCGAGCAGTCCCTCAATTGGTCCGATGAGACTGTATACGCCGCCGTTGGGACGCGG
ACTCAACAACCCCCTCATACCCATTCGCCCGTCCAGAGCGGAACGACTCCGCGGTGCC
GGCGTCAGGCCTCCTCGCCTAGGCTGCGCCATTATGTTGGGAGGTGCGTTGACTGGGG
GCCGGCGACCTGAGCTTGGTCGGCCCGTGAAGCCCATCACTCCCATGGAGACGTTTTCT
CCCACCCATTGGCCCCCGGGGTCCTCCACCAATTGCGTCGGAGTCTTGTCTAATATGAA
ATTTATTCATCGTAAGGTATAAACCGCCCCCGCGTGGGTGCGGCTCGAAGTCCGGCTTC
CCAAGCTGGCTGCTAACACGGCAGTGTACCCGGTTCCACTCAGAGGGCCTGAGGCAGT
ACCTACGGATCTCAAAATCCCTAGTCCCCATGATACGTACAGCTGGGCTAGGTGGGAT
TAATACGTCGGGGTCCCATCGCGGGATGTTTGAGGGGGAGATACGTTCCAGCCAGATA
CTCCCTGTTCGCGCCCCTCAGAGAAGTCCATTACGCTGCCGTTGACGTCGTCAATGCCG
CGCCACTAACACCGGACTAGGGGGACCAAACCATACCATGTAATACGTGTACTTGCCC
ATTGTCGCGGTTCAGAGTACGGGGCTGTGAGTAATGTACGCGTTCCTGGTGGGAACTT
GTCCTCTGAACTTGAATAACAACTCACTGGAATGGGATTTAGCAAACCATCCCAACAT
ATTCCAGGCCTCTGATCAGATCACAATTTTGCTTTCACCAGTTCCCAATTATGATTCCGC
ATCGTCGGGAGGCCTTCGACTAGATCTGCTCGCATGGTTCGAGCGGTAATATCGGGTCT
GAAATCCCCTGACCCAACGGGAGGCGGTATGCGTGATCGGAATCTGCACCTTGCGAGA
GCTAATCGTGATGACAATGGCTCTTCCACAAGTGAGTGCGGGGGCGCGAGCGGCGAAC
CAGATGGTCCTCTGAATATTCCCCCCCGTGCCGAAGGCTGAACTCGAGGCGGGGCCGT
GGTCGCGCACCGTTACCACGCAGGTGCGTCGCGAGGCCCCTTGAAGGTCCCGTCATGA
AG

>TT_B40.2

GTCGGGATGCCTTCCCAACTCCGGCACGGCGCGGCGCCTTCGCGCACGGATCGGATAA
GCCTTCCCGTGGACGGCTTGTCTTCAAAAATACCTTGTAGCCTTCTCGTGGTTTACCCC
AATGCCGCGCCGCGTCTGCTTAGATGCTGAATTGCGGAAGCTATTCGAGAGTCCTGTTG
CCCGGCCGGGGGCAGCACACAACCATGGGATCGGCGCAGCCCACCTCCGGCCAACGA
GAGTTACGGCGTCCCGGCCCGTTTTCCCGCGTCGGAGGCTGCCAACACGATAGTAACT
GAAATGGCCCAAACGTATTAATACGCACAGGGGGCGGGCCTCTGGCATGTCACTGGAT
CAGGCCCGTGGCGTGCCCGCCTCGTCAGCGCCACCCATTGCTAAGCGCTGACAGTAAT
AGACCCCTCCATAGTAGTTGCCGATGTTGATTTGGTCACCGGCCGAAACGTATGCGCTC
AGCACAGGGCAGGTACTACGGAGCGAAAGGTGGATGATTGGCAGGGGCCGCTGGCGC
ACCTACAAAACTATTCGTCCGCGCCTGCTGGAGCGACCAACTACGCTCTATAGCGTCCA
GTACCCGAGCAGTCCCTCAATTGGTCCGATGAGACTGTATACGCCGCCGTTGGGACGC
GGACTAATCAACCCCCTCATACCCATTCGCCCGTCCAGAGCGGAACGACTCCGCGGTG
CCGGCGTGAGGCCTCCTCGCCTAGGCTGCGCCATTATGTTGGGAGGTGCGTTGACTGG

GGGCCGGCGACCTGAGCTTGGTCGGCCCGTGAAGCCCATCACTCCCATGGAGACGTTT
TCTCCCACCCATTGGCCCCCGGGGTCCTCCACCAATTGCGTCGGAGTCTTGTCTAATAT
GAAATTTATTCATCGTAAGGTATAAACCGCCCCCGCGTGGGTGCGGCTCGAAGTCCGG
CTTCCCAAGCTGGCTGCTAACACGGAAGTGTACCCGGTTCCACTCAGAGGGCCTGAGG
CAGTACCTACGGATCTCAAAATCCCTAGTCCCCATGATACGTACAGCTGGGCTAGGTG
GGATTAATACGTCGGGGTCCCATCGCGGGATGTTTGAGGGGGAGATACGTTCCAGCCA
TATACTCCCTGTTCGCGCCCCTCAGAGAAGTCCATTACGCTGCCGTTGACGTCGTCAAT
GCCGCGCCACTAACACCGGACTAGGGGGACCAAACCATACCATGTAATACGTGTACTT
GCCCATTGTCGCGGTTCAGAGTACGGGGCTGTGAGTAATGTACGCGTTCCTGGTGGGA
ACTTGTCCTCTGAACTTGAATAACAGCTCACTGGAATGGGATTTAGCAAACCATCCCAA
CATATTCCAGGCCTCTGATCAGATCACAATTTTGCTTTCACCAGTTCCCAATTATGATTC
CGCATCGTCGGGAGGCCTTCGACTAGATCTGCTCGCATGGTTCGAGCGGTAATATCGG
GTCTGAAATCCCCTGACCCAACGGGAGGCGGTATGCGTGATCGGAATCTGCACCTTGC
GAGAGCTAATCGTGATGACAATGGCTCTTCCACAAGTGAGTGCGGGGGCGCGAGCGGC
GAACCAGATGGTCCTCTGAATATTCCCCCCCGTGCCGAAGGCTGAACTCGAGGCGGGG
CCGTGGTCGCGCACCGTTACCACGCAGGTGCGTCGCGAGGCCCCTTGAAGGTCCCGTC
ATGAAG

>TT_MLZ7.1

GTCGGGTTGCCTCCTCGGCTCTGGCACGGCGCGGCGCTTTCGCGCACGGATCGGATAA
GCTTTTCCGTGGACCACTCGTCCTCAGAAATATCTCGTAGTCTTCTCGTTGGTTATTCCA
ATGAAGCGCCTCGTCTGTGGGGGCACTGAACCGCTAGAGCTATTCGAGTGCTCTACTA
CTCCACCGGGGGCAGCACGCAGCCGTGGGATCGGCACGGCCCACCTCCAGCCAACGAG
AGTTACGAGTACCCGACGCGTTTTCCCGCGTCGGAGGCTGCCAACACGATAGTAACTG
CACTGGCCCAAACGTATTAATACGCCCCGGGGGCGGGCCTCTGGCGCGCCACTGGATC
AGGCCCGTGGCGTGCCCGCCTCGTCAGCGCCACCCATTGCTAAGCGCTGACAGTAATA
GACCCCTCCAGAGTAGTTGCCGATGTTGATTCGGTCACCGACCGAAACGTATGCACTTA
GCACAGGGCAGGTACTACAAAGCGAGAGGGGGATGATTGGCAGGGGCTGCTGACGCG
CCTATCAGCCCGTTCCCCCGCGCCTGCTGTGGCGACCAACTACGCCCCGCAGCGTCCAG
TACCCGAGCAGTCTCTCAACTGGTCCGATTAGACTGTATACACCGCCGTTGGGACGCG
GACTAAACAACTCCCTCATACCCATCCGCCCGTCCGGAGCGGAACGACTCGGCGGTAC
CGGCGGCAGGCCCCCTCGCCTAGACTGCACCATTATGTTGGGAGGTGCGTCGACTGGG
GGCCGGCGACTTGATCCTGGTCGGCCCGTGAAGCCCATCAGTCCCATGGAGACGTTCT
CTCCCATCTATTGGCCTCCGGGGCTCTCCACCAATCGCACCGGAGTCTTGTCTAATATG
AAATTTAAACATCGTGAGGTATAAACCGCCCCCGAGTGGGGGCGGCTTGAAGTCCAGC
CCCCCAGGCCGCCTGCTAACACGGAAGTGTACCCGGTTCCACTCAGAGGGCATGAGGC
AGTACCTACGGATCTCAACAAGCCTAGTCCCCATGATACGTACAGCTGGGCCAGGTGG
GATTAATACGCCGGGGTTCCGTTGCGGAACGGCTAGAAGGTAAATACGCCTTGGCCAT
ATACTCTTTGTCCGCATCCCTTGGGGAAATCCATTATGCTGCCGTTGACGTCGTCAACG
TCACGCCACTAACACCGAACTAGGGGGACCAAACTATACCGTGTTATCCGTATACTTA
CCCACTGTTGCAGGTCAAGGTATGGGGCTGCAAGTAATGTACATACTCCTGGTGGGAA
CTTGTCCTCCGGACTTGAATGGCTACCTGCCGGGCTGGGACTTAGCAAACCACCCTGAC
GCATCCCAGGCCTTTGATCGGATCACAATTTTGCTTTCACCAGTTCCCAATTACGGTTC
CGCATCGTCGGGAGGCCTTCGACTAGATCTACTCGCATGGTTCGAGCAGTAATATCGG
GTCTGAAATCCCCTGACCCAACGGGGGGCAGTATGTGTGGTCGGAATCTGCACCTTGC
GAAAGATAATCGTAATGACGATGGACCTTCTACAAGTGAGTGCTGGGGCACGAGCGGC
TGACCAGATTGTCTTCCGAATGTTCCCCCCCGTGCCGAAGGCTGAACCCGAGGCGGAG
CCGGGGGCGTGCACCGTTGCCACGCAGGTGCACCGCGAGGCACCTTGAAGGTTCCGTC
ATGAAG

>TT_MLZ7.2

GTCGGGATGCCCTTTCGACACCAGCACGGCGCGACGCTTTCGCGCACGTATCAGATAA
GTTTTCCCGTGGACGGCTCGTCCTCAGAAATATCTCGTAGTCCGTTCGTTGGTTACTCC
AATGAAGCGCCTCGCCTGCGGGAGCACTAAACCGCGAGCGCCATTCGAGTGCTCTACA
ACTCGACCGGGGGCAGCACACAACCGTAGGATCGGCGCGGCTCATTTTCGGCTAGTAG
GGGGTCCGACGTCCCGGCCCGTTTTCTCGCGTCGGAGGCTGCCAACACGATAGTCACT
GAACTGGCCCAAACGTATTGATACGCCCAGGGGGCGGGTCTCTGGCGCGCCACTGGAT
CAGGCCCGTGGCGTGCCCGCCTCGTCAGCGCCACCCATTGCTAAGCGCTGACAGTCAT
AGACCCCTCCATAGTAGTTGCCGATGTTGATTCGGTCACCGGCTGAAACGTATGCACTT
AGCACAGGGCAGGTACTACAAAGCGAGAGGGGGATGATTGGCAGGGGCTGCTGACGC
GCCTATCAGCCCGTTCCCCCGCGCCTGCTGTGGCGACCAACTACGCCCCGCAGCGTCCA
GTACCCGAGCAGTCTCTCAACTGGTCCGATTAGACTGTATACACCGCCGTTGGGACGC
GGACTAAACAACTCCCTCATACCCATCCGCCCGTCCGGAGCGGAACGACTCGGCGGTA
CCGGCGTCAGGCCCCCTCGCCTAGACTGCACCATTATGTTGGGAGGTGCGTCGACTGG
GGGCCGGCGACTTGATCCTGGTCGGCCCGTGAAGCCCATCAGTCCCATGGAGACGGTC
TCTCCCATCTATTGGCCTCCGGGGCTCTCCACCAATCGCACCGGAGTCTTGTCTAATAT
GAAATTTATTCATCGTGAGGTATAAACCGCCCCCGAGTGGGTGCGGCTTGAAGTCCAG
CCCCCCAGGCCGCCTGCTAACACGGAAGTGTACCCGGTTCCACTCAGAGGGCATGAGG
CAGTACCTACGGATCTCAACAAGCCTAGTCCCCATGATACGTACAGCTGGGCCAGGTG
GGATTAATACGCCGGGGTTCCGTTGCGGAACGGCTAGAAGGTAAATACGCCTTGGCCA
TATACTCTTGGTCCGCATCCCTTGGGGAAATCCATTATGCTGCCGTTGACGTCGTCAAC
GTCACGCCACTAACACCGAACTAGGGGGACCAAACTATACCATGTTATCCGTATACTT
ACCCACTGTTGCAGGTCAAGGTATGGGGCTGCAAGTAATGTACATACTCCTGGTGGGA
ACTTGTCCTCCGGACGTGAATGGCTACCTGCCGGGCTGGGACTTAGCAAACCACCCTG
ACGCATCCCAGGCCTTTGATCGGATCACAATTTTGCTTTCACCAGTTCCCAATTACGGT
TCCGCATCGTCGGGAGTCCTTCGACTAGATCTACTCGCATGGTTCGAGCAGTAATATCG
GGTCTGAAATCCCCTGACCCAACGGGGGGCAGTATGTGTGGTCGGAATCTGCACCTTG
CGAGAGATAATCGTAATGACGATGGACCTTCTACAAGTGAGTGCTGGGGCACGAGCGG
CTGACCAGATTGTCTTCCGAATGTTCCCCCCCGTGCCGAAGGCTGAACCCGAGGCGGA
GCCGTGGGCGTGCACCGTTGCCACGCAGGTGCACCGCGAGGCACCTTGAAGGTCCCGT
CATGAAG

>TT_MLZ14.1

GTCGGGATGCCCTTTCGACACCAGCACGGCGCGACGCTTTCGCGCACGTATCAGATAA
GTTTTCCCGTGGACGGCTCGTCCTCAGAAATATCTCGTAGTCCTTTCGTTGGTTACTCCA
ATGAAGCGCCTCGCCTGCGGGAGCACTAAACCGCGAGCGCCAGTCGAGTGCTCTACAA
CTCGACCGGGGGCAGCACACAACCGTAGGATCGGCGCGGCTCATTGTCGGCTAGTAGG
GGGTCCGACGTTTCGGCGCGTTTTCCCGCGTCGGAGGCTGCCAACACGATAGTAACTG
AAATGGCCCAAACGTATTAATACGCACAGGGGGCGGGCCTCTGGCGCGCCACTGGATC
AGGCCCGTGGCGTGCCCGCCTCGTCAGCGCCACCCATTGCTAAGCGCTGACAGTAATA
GACCCCTCCATAGTAGTTGCCGATGTTGATTCGGTCACCGGTCGAAACGTATACACTCG
GTACGGGGTGGGTACCACGGAGCGAAAGGTGGATGATTGGCAGGAGCCGCTGGCGCA
CCTACAAAACTATTCGTTCGCGCCTGCTGGAATGACCAACTACGCTCTATAGCGTCCAG
TACTCGAGCAGTCCCTCAATTGGTCCGATGAGACTGTATACGCCGCCGTTGGGACGCG
GACTAAACAACCCCCTCATACCCATTCGCCCGTCCGGAGCGGAACGACTCGGCGGTGC
CGGCGTCAGGCCTCCTCGCCTAGGCTGCGCCATTATGTTGGGAGGTGCGTTGACTGGG
GGCCGGCGACCTGAGCTTGGTCGGCCCGTGAAGCCCATCACTCCCATGGAGACGTTTT
CTCCCACCCATTGGCCCCCGGGGTCCTCCATCAATCGCGTCGGAGTATCGTCTAATGTG
AAATTTATTCATCGTGAGGTATACACCGCCCCCGCGTGGGTGCGGCTCGAAGTCCGGC
CCCCCAGGCTGCCTGCTAACACGGAAGTATGCCTCGTTTAGCTCAGAAGGGACGAGAC
ACTCTAGACGGCTCTCAACAAGCCTAGTCCCCACGGTACATACTACCGTGCCAGGTGG
GATTGGTACGCCGGGGTCCCGTCGCAGGATGTCTAGGGGGGAGATACGTTCCGGTCAT

ATACTCTTTGTCCGCATCCCTTGGGAAAATCCATTACGCTGTCATTGACGTCGTCAACG
TCATGCCACTAACACCGAACTAGGGGGACCAAACTATACCATGTTATCCGTATGCCTA
CTCGTTGTCGCGGTTCAGAGTACGGGCCTGTGAGTAATGTACGCGCTCCGGGTGGGAA
CGTGTCCTCCGGACTTGAATGGTTACTCGCCGAGCTGGAACTTAGCAAACCACCCTGAT
GCATTCCAGGTATCAGATTAGAACACAATTTTGCTTTCACCAGTTCCCAATTGCGGGGC
CGTATCGTCGGGAGGCCTTCGACCGGGTCTACTCGCATAGTTCGAGCGGTAATATTGG
ATCTGAAATCTCCTGACCCAACGGGGGGCGGTACGTGTGATCGGGATCTGCACCTTGC
GAGCTCTAATCGTAATGACGATGGCTCTTCCACAAGTGAGTGCTGGGGCACGAGCGGC
GGACCAGATGGCCTTCCGAATGTTCCCCCCCGTGCCGAAGGCTTAACTCGAGGCGGAG
CCGTGGGCTCGCACCGTTGCCGCGCAGGTACACCGCGAGGTCCCTTGAAGGTCCCATC
ATGAAG

>TT_MLZ14.2

GTCGGGATGCCCTTTCGACACCAGCACGGCGCGACGCTTTCGCGCACGTATCAGATAA
GCTTTCCCGTGGACGGCTCGTCCTCAGAAATATCTCGTAGTCCTTTCGTTGGTTACTCCA
ATGAAGCGCCTCGCCTGCGGGAGCACTAAACCGCGAGCGCCATTCGAGTGCTCTACAA
CTCGACCGGGGGCAGCACACAACCGTAGGATCGGCGCGGCTCATTTTCGGCTAGTAGG
GGGTACGACGTCCCGGCCCGTTTTCTCGCGTCGGAGGCTGCCAACACGATAGTCACTG
AACTGGCCCAAACGTATTGATACGCCCAGGGGGCGGGCCTCTGGCGCGCCACGGGATC
AGGCCCGTGGCGTGCCCGCCTCGTCAGCGCCACCCATTGCTAAGCGCTGACAGTAATA
GACCCCTCCATAGTAGTTGCCGATGTTGATTTGGTCACCGGCCGAAACGTATGCGCTCA
GCACAGGGCAGGTACTACGGAGCGAAAGGTGGATGATTGGCAGGGGCCGCTGGCGCA
CCTACAAAACTATTCCTCCGCGCCTGTCGGAGCGACCAACCACGCTCTATAGCGTCCAG
TACCCGAGCAGTCCCTCAATTGGTCCGATGAGACTGTATACGCCGCCGTTGGGACGCG
GCCTAAATAACCCCTTTACGCACATCCGCCCATCCGGAGCGGAACGACTCGGCGGTAC
CGGCGTCAGGCCCCCTCGCCTAGACTGCACCATTATGTTGGGAGGTGCGTCGACTGGG
GGCCGGTGACTTGATCCTGGTCGGCCCGTGAGGCCCATCAGTCCTATGGAGACGTTCTC
TCCCATCCATTGGCCTCCTAGGCTTTCCACCAATTGCGTCAGGGTAATGCCAGATGTTA
GATGTATTCATCGTGAGGTATACACCGCCCCCGCGTGGGTGCGGCTCGAAGCCCGGCC
TCCCAGGCTGGCTGCTAACACGGAAGTGTGGCCGGTTCCACTCAGAAGGCATGAGGCA
GTACCTACGGATATCAACAAGCCTAGTCCCCATGATACGTACAGCTGGGCCAGGTGGG
ATCAATACGTCGGAAGCCCATCGCGGGATGTTTGAGGGGGAGATCCGTTCCGGCCATA
TACTCCCTGTCCGCATCCCTTGGGGTAATCCATTACGCCGCCGTTGACGTCGTCAACGT
CACGCCACTAACACCGAACTAGGGGGACCAAACTATACCATGTTATCCGTATACTTAC
CCACTGTTGCAGGTCAAGGTATGGGGCTGCAAGTAATGTACATACTCCTGGTGGGAAC
TTGTCCTCCGGACTTGAATGGCTACCTGCCGGGCTGGGACTTAGCAAACCACCCTGACG
CATCCCAGGCCTTTGATCGGATCACAATTTTGCTTTCACCAGTTCCCAATTACGGTTCC
GCATCGTCGGGAGGCCTTCGACTAGATCTACTCGCATGGTTCGAGCAGTAATATCGGG
TCTGAAATCCCCTGACCCAACGGGGGGCAGTATGTGTGGTCGGAATCTGCACCTTGCG
AGAGATAATCGTAATGACGATGGACCTTCTACAAGTGAGTGCTGGGGCACGAGCGGCT
GACCAGATTGTCTTCCGAATGTTCCCCCCCGTGCCGAAGGCTGAACCCGAGGCGGAGC
CGTGGGCGTGCACCGTTGCCACGCAGGTGCACCGCGAGGCACCTTGAAGGTTCCGTCA
TGAAG

>TT_MLZ30.1

GTCGGGTTGCCTCCTCGGCTCTGGCACGGCGCGGCGCTTTCGCGCACGGATCGGATAA
GCTTTTCCGCGGACCACTCGTCCTCAGAAATATCTCGTAGTCTTCTCGTTGGTTATTCCA
ATGAAGCGCCTCGTCTGTGGGGGCACTGAACCGCTAGAGCTATTCGAGTGCTCTACTA
CTCCACCGGGGGCAGCACGCAGCCGTGGGATCGGCACGGCCCACCTCCAGCCAACGAG
AGTTACGAGTACCCGACGCGTTTTCCCGCGTCGGAGGCTGCCAACACGATAGTAACTG

AACTGGCCCAAACGTATTAATACGCCCCGGGGGCGGGCCTCTGGCGCGCCACTGGATC
AGGCCCGTGGCGTGCCCGCCTCGTCAGCGCCACCCATTGCTAAGCGCTGACAGTAATA
GACCCCTCCAGAGTAGTTGCCGATGTTGATTCGGTCACCGGTCGAAAAGTATACACTC
GGTACGGGGTGGGTACCACGGAGCGAAAGGTGGATGATTGGCAGGAGCCGCTGGCGC
ACCTACAAAACTATTCGTTCGCGCCTGCTGGAATGACCAACTACGCTCTATAGCGTCCA
GTACTCGAGTAGTCCCTCAATTGGTCCGATGAGACTGTATACGCCGCCGTTGGGACGC
GGCCTAAATAACCCCTTTACGCACATCCGCCCATCCGGAGCGGAACGACTCGGCGGTA
CCGGCGTCAGGCCCCCTCGCCTAGACTGCACCATTATGTTGGGAGGTGCGTCGACTGG
GGGCCGGTGACTTGATCCTGGTCGGCCCGTGAGGCCCATCAGTCCTATGGAGACGTTCT
CTCCCATCCATTGGCCTCCTGGGCTTTCCACCAATTGCGTCAGGGTAATGCCAGATGTT
AGATGTATTCATCGTGAGGTATACACCGCCCCCGCGTGGGTGCGGCTCGAAGCCCGGC
CTCCCAGGCTGGCTGCTAACACGGAAGTGTAGCCGGTTCCACTCAGAGGGCACGAGGC
AGTACCTACGGATATCAACAAGCCTAGTCCCCATGATACGTACAGCTGGGCCAGGTGG
GATCAATACGTCGGAAGCCCATCGCGGGATGTTTGAGGGGGAGATCCGTTCCGGCCAT
ATACTCCCTGTCCGCATCCCTTGGGGTAATCCAGTACGCCGTCGTTGACGTTATCAATG
CCGCGCGATTAACACCGAGTTAATGGGACCAAAACATACCATGTAATCCGTGTACTTA
CCCATCGTTGCGGTACAGGATACGGGGCTGTGAGTAATGTACACACTCCTGGAGGGAG
CTTGTCCTCCAGACTTGAATGGCTACCTGCCGGGCTGGGACTTAGCAAACCACCCTGAC
GCATCCCAGGCCTTTGATCGGATCACAATTTTGCTTTCACCAGTTCCCAATTACGGTTC
CGCATCGTCGGGAGGCCTTCGACTAGATCTACTCGCATGGTTCGAGCAGTAATATCGG
GTCTGAAATCCCCTGACCCAACGGGGGGCAGTATGTGTGGTCGGAATCTGCACCTTGC
GAGCGATAATTGTAATGACGATGGACCTTCTACAAGTGAGTGCTGGGGCACGAGCGGC
GGACCAGATGGTCTTCTGAATGTTCCCTCCCGTGCCGAAGACCGAACCCGAGGCGGAG
CCGTGGGCGTGCACCGTTGCCACGCAGGTGCACCGCGAGGCACCTTGAAGGTCCCGTC
ATGACG

>TT_MLZ30.2

GTCGGGATGCCCTCTCGATACTGGCACGGAGCGGCGCCTTCGCGCACGGATCGGATAA
GCTTTCCCGTAGATGGCTCGTCCTCGGCAATATCTCATAGTCTTCTCGTTGGTTACTCCA
ATGAAGCGCCTCGTCTGCGGGGGCACTAAACCGCGAGAGCTATTCAAGTGCTCTACTA
CTCGACCGGGGGCAGCTTACAACCGTGGGATCGGCGCGGCCCACCGCCGGTCAACGAG
AGTTACGAGTTCCCCGCCCGTTTTCCCGCGTCGGAGGTTGCCAACAGGATAGTAACTGA
ACTGGCCCAAACGTATTAATACGCCCCGGGGGCGGGCCTCTGGCGCGCCACTGGATCA
GGCCCGTGGCGTGCCCGCCTCGTCAGCGCCACCCATTGCTAAGCGCTGACAGTAATAG
ACCCCTCCATAGTAGTTGCCGATGTTGATTTGGTCACCGGCCGAAACGTATGCGCTCAG
CACAGGGCAGGTACTACGGAGCGAAAGGTGGATGATTTGCAGGGGCCGCTGGCGCAC
CTACAAAACTATTCGTCCGCGCCTGCTGGAGCGACCAACTACGCTCTATAGCGTCCAGT
ACCCGAGCAGTCTCTCAACTGGTCCGATTAGACTGGATACACCGCCGTTGGGACGCGG
ACTAAACAATTCCCTCATACCCATCCGCCCGTCCGGAGCGGAACGACTCGACGGTACC
GGCGTCAGGCCCCCTCGCCTAGGCTGCACCATTATGTTGGAAGGTACATCGACTGGGG
GCCGGCAACTTGAGCTTGGTCGGCCCGTGAAACCCACCAGTCCCATGGAGAAGTTCTC
TCCCATCCATTGGCCTCCGGGGCTCTTCATCAATCGCGTCGGAGTATCGTCTAATGTGA
AATTTATTCATCGTGAGGTATACACCGCCCCCGCGTGGGTGCGGCTCGAAGCCCGGCTT
CCCAGGCTGGCAGCTAACACGGAAGTGTGCCTCGTTTCGCTCAGAAGGGACGGACAC
TCTCGACGGCCCTCAACAAGCCTAGTCCCCACGATACATACAACCGTGCCAGGTGGAA
TTAATACGTCGGGGTCCCATCGCGGGATGTTTGAGGGGGAGATACGTTCCGGCCATAT
ACTCCCGGTTCGCGCCCCTCAGAGAAGTCCATTACGCTGCCGTTGACGTCGTCAATGCC
GCGCCACTAACACCGGACTAGGGGGACCAAACCATACCATGTAATCCGTGTACTTGCC
CATTGTTACGGTTTAGGGAACGGGGCTGTGGCTAATAGACGCGCTCCTGGTGGGAGCT
TGTCCTCCGGACTCGAATGGCTACTCGCCGGGCTGGGACTTAGCAAACCACTCTGACG
CATCTACGGCCTCAGATTAGATCACAATTTTGCTTTCTCCATTTCCTAATTACCGTTCCG

CATCGTCGGGAGGCCTTCGACTAGATTTACTCGCATGGTTTGAGTGATAATATCGGGTC
GGGAATCCTCTGACCCAACGTGAAGCGGTATGCGCAATTGGGGTCTGTTCCTTGCAAG
AGCTAATCGTAATGACGATGGACCTTCTACAAGTGAGTGCTGTAGTGCCAGCAGCGGA
CCAGATTGTTTTCCGGATGTTCCCCCTCGTGCCGAAGGCTGAACTCAGGGCGGGGCCGT
GGGCGCGCATCGTTGCCACGCAGGTACACCGTGAGGCACCTTGAAGGTCCCGTCATGA
AC

>YNT_DQZ4.1

GTCGGGATGCCCTTTCGACACCAGCACGGCGCGACGCTTTCGCGCACGGATCGGATAA
GCTTTTCCGTGGACCACTCGTCCTCAGAAATATCTCGTAGTCTTCTCGTTGGTTATTCCA
ATGAAGCGCCTCGTCTGTGGGGGCACTGAACCGCTAGAGCTATTCGAGTGCTCTACTA
CTCCACCGGGGGCAGCACGCAGCCGTGGGATCGGCACGGCCCACCTCCAGCCAACGAG
AGTTACGAGTACCCGACGCGTTTTCCCGCGTCGGAGGCTGCCAACACGATAGTAACTG
AACTGGCCCAAACGTATTAATACGCCCCGGGGGCAGGCCTCTGGCGCGCCACTGGATC
AGGCCCGTGGCGTGCCCGCCTCGTCAGCGCCACCCATTGCTAAGCGCTGACAGTAATA
GACCCCTCCATAGTAGTTACCGATGTTAATTCGGTCACCGGCCGAAACGTGCGCGCTCA
GTGCAGGGCAGGTCCTGTAGAACGGAGGGTGGATATTTGGCAAAGGCTGCTGGTGCGC
CTACCAACTTGTCCCTCCGCGCTTGCTAGAGCGACCAACTACGCTCTGTAACGTCCAGT
ACCCGAGCAGTCTCTCAACTTGGCCGATGAGACTGTATACACCGCCGTTGGGACACGG
ACTAAACAACTCCCTCATACCCATCCGCCCGTCCGGAGCGAAAGGACTCGGCAATACC
GGCGTCAGGGCCCCTCGCCTGAACTGCATCATTATAATGGGAGGTGCGTCGACTGGGG
GCTGGCGACTTGATCCCGGTCGGCCCGTGAAGCCCATCAGTCTCATGGAGCCGTTCTCT
CCCATACGTTGGCCTCCTGGGCTTCCCATCAATTGTGTCGAAATATTGTCTAATGTGAA
ATTTATTCATCGTGAGGTGTAAACCGCCCCCGCGTGACTGTAGAGCGTAATCCGGCCTT
CTGGGCTAGCAGCTAACGAGGAAGTGTGCTTCATTTCGTTTGGAAAGGACGAAATGCT
CTAGGCGGCTCTCAACCAGCCTAGTCCCCACGATACATACAACCGTACCAGGTCGAAT
TAATCCGCCGAGGTCCCGTCGCGGGATGTTTGAGGGGGAGATACGTTCCGGCCATATA
CTTTTTGTCCGCATCCCTTGGGGAAATCCATTACGCTGCCGTTGATGTCGTTAACGTCA
CGCCACTAACACCGAACTAGGGGGACCAAACTATACCATGTTATCCGTATACTTACCC
ACTGCCGTGGTTCGGAGTACGCTGCTGTGAGTAATGTACGCGCTCCTCGTGGGGATTTG
TCGTCTGAACTTGAATAACAGCTCACTCGGATGGGATTTAGCAAACCATCCCAACATAT
TCCAGGCCTCTGATCAGATCACAATTTTGCTGTCACCAGTTTCCAATTACGGTTGTGCA
TCGCCGGGAGGCTTGCGATTAGATCTACTCGCATGATTCGAGCGGTAATATCGGGTCTG
AAATTCCCTAACCCAGCAGGAGGCGGTACGTGTGGTCGGAATCTGCACCTTGCGAGAG
CTAGTCGTAATGACGATGGCTTTTCCACAAGTGAGTGCTGGGGCGCGAGCGGCGGACC
AGATGGTCTTCCGAATGTTCCCCCCCGCGCTGAAGGCTGAACTCGATGTGCGACCGTA
GGCTCGCACCGTTGCCACGCATGCGCACCGCGAGGTCCCTTGAAGATCCCGTCATCAA
C

>YNT_DQZ4.2

GTCGGGATGCCTTCTCGACTCCGGGACGGCGTGGCACCTTCGTGCACGGATGGGATAA
GCTTTCCCGTAGATGGCTCGTTCTCAGAAATATCTCGTAGTCTTCTCGTTGGTTACTCCA
ATGAAGCGCCTCGTCTGCGGGGGCACTGAACCGCGAGAGCTATTCAAGTGCTCTACTA
CTCGACCGGGGGCAGCTTACAACCGCGGGATTGGCACGGTCTACCTCTGTCCAACAGG
GGGTACGACGACCCGGCCCGTTTTCCCGCGTCGGAGGCTGCCAACACGATAGTAACTG
AACTGGCCCAAACGTATTAATACGCCCCGGGGGCGGGCCTCTGGCGCGCCACTGGATC
AGGCCCGTGGCGTGCCCGCCTCGTCAGCGCCACCCATTGCTAAGCGCTGACAGTAATA
GACCCCTCCATAGTAGTTGCCGATGTTGATTTGGTCACCGGCCGAAACGTATGCGCTCA
GCACAGGGCAGGTACTACGGAGCGAAAGGTGGATGATTGGCAGGGGCCGCTGGCGCA
CCTACAAAACTATTCGTCCGCGCCTGCTGGAGCGACCAACTACGCTCTATAGCGTCCAG

TACCCGAGCAGTCCCTCAACTGATCCGATGAGACTTTATACACCGCCGGTGGGACGCG
GACTACACAACCCCCTCATACCCATTCGCCCGTCCGGAGCAGAACAACACGGCGGTAC
CGGCGTCAGGCCCCCTCGCCTAGGCTGCACCATTATGTTGGGAGGTGCGTCGATTGGG
GGTCGGCGACTTGATCCCGGTCGGCCCGTGAAGCCCATCAGTCCCGTGGAGACGTTCT
CTCCCATCCATTGGCCTCCGGAGCTCTTCATCAATCGCACCGGAGTATTGCCAGATGTT
AAGCGTATTCATCGTAGAGCACAAGCCGCCTTCGCATGGGTACAGCGCGTAATCCGGC
CCCCCAGGCTGCCTGCTAACACGGAAGTGTACCCGGTTCCACTCAGAGGGCATGAGGC
AGTACCTACGGATCTCAACAAGTCTAGCTCGCACAATATATATAACCATGCCCGGTGG
GATTAATACGTCGGGGTCTCATCGCGGGATGTTTGAGGGGGAGATACGTTCCAGCCAT
ATACTCCCTGTTCGCGCCCCTCAGAGAAGTCCATTACGCTGCCGTTGACGTCGTCAATG
CCGCGCCACTAACACCGGACTAGGGGGACCAAACCATACCATGTAATCCGTGTACTTG
CCCATTGTTGCGGTTCAGGGTACGGGGCTATGAGTAATGGGCACACTCCTGGTAGGAA
CTTGGTCCCCGGCTTTGTATGGCTACTCGCCGAGCTCGGCCTTAGCAAACCACCCTGAC
GCATCCCAGGCCTTTGATCGGATCACAATTTTGCTTTCACCAGTACCCAATTACGGTTC
CGCATCGTCGGGAGGCCTTCGACTAGATCTACTCGCATGGTTCGAGCGGTAATATCGG
GTCTGAAATCCCCCGACCCGACGGGAGGCGGTACGTGTGGTCGGGATCTGCACCTTGC
GAGAGCTAATCGTAATGACGATGGCTCTTCCACAAGTGAGTGCTGGGGCGCGAGCGGC
GGACCAGATTGTCTTCCGAATGTTTCCCCCCGTGCCGAAGGCTGAACTCGAGGCGGAG
CAGTGGGCGCGTACCGTCGACATGCAGGTGCACTATGAGGTCCCTTGAAGGTCCCGTC
ATGAAG

>YNT_DQZ12.1

GTCGGGACGCCCTCCCGGCTCTGGCACGGCGCGGTGGCTTTGCGCACGGATCGGATAA
ACTTTCCATTGGACGGCTCGTTCTCAGAAATATCTCGTAGTCTTCTCGTTGGTTACTCCA
ATGAAACGCCTCGTCTGCGGGGGCACTGCACCGCGAGAGCTATTCAAATGCTCTACTA
CTCGACCGGGGGCAGCTTACAACCGTGGGATCGGCGCGGCCCACCTCCGGTCAACGAG
AGTTACGAGTTCCCGGCCCGTTTTCTCGCGTCGGAGGCTGCCAACACGATAGTAACTGA
ACTGGCCCAAACGTATTAATACGCCCCGGGGGCGGGCCTCTGGCGCGCCACTGGATCA
GCCCCGTGGCGTGCCCGCCTCGTCAGCGCCACCCATTGCTAAGCGCTGACAGTAATAG
ACCCCTCCATAGTAGTTGCCGATGTTGATTTGGTCACCGGCCGAAACGTATGCGCTCAG
CACAGGGCAGGTACTACGGAGCGAAAGGTGGATGATTGGAAGGGGCCGCTGGCGCAC
CTACAAAACTATTCGTCCGCGCCTGTTGGAGCGACCACCTACGCTCTATAGCGTCCAGT
ACCCGAGCAGTCTCTTAACTGGTCCGGTGAGACTGTAAACATCCTTGTTGGGTCGTTGC
ATAAACAACCCACTCTTACCCATCCGCCCGTCCGGAGCAGAACGACTTGGCGGTACCG
ACGTCAGGGCCCCTCGCCTAGGCTGCATCATTATGTTGGGAGATGCGTCGACTGGGGG
CCGGCGACTTGATCCTGGTCGGCCCGTGAGGCCCATCAGTCCTATGGAGACGTTCTCTC
CCATCCATTGGCCCCCGGGGTCCTCTACCGATTGCGTCGGAGTCTTGTATAATATGAAA
TTTATTCATCGTGAGGCATAAACCGCCCCCGCGTGGGTGCGGCTCGAAGCCCGGCTTCC
CAGGTTGGCAGCTAACACGGAAGTGTGCCTCGTTTCGCTCAGGAGGGACGGGACACTC
TCGACGCCTCTCAACAAGCCTAGTCCCCACGATACATACAACCGTGCCAGGTGGAATT
AAGATGCCGGGGTCTCGTCGCGAGCCATCCAGGGGGGAGATACGTTCCGGCCATATAC
TCTCGGTCCACATCCCTTGAGGAAATCCATTACGCTGCCGTTGACGTCGTTAACGCCGC
TTCATTAACGTCGAACTAGGGAGACCAAACTATACCATGTTATCCGTATGCCCACCCAT
TTTTGCGGTTCAGAGTACGGGCCTGTGAGTAATGTACGCGCTCCTGGTGGGAACTTGTC
CTCCGGACTTGAATGGTTACTCGCCGAGCTGGAACTTAGCAAACCACCCTGATGCATTC
CAGGTATCAGATTAGAACACAATTTTGCTTTCACCAGTTCCCAATTGCGGGGCCGTATC
GTCGGGAGGCCTTCGACTAGATTTACTCGCATGGTTTAAGTGATAATATCAAGTCGGG
AATCCTCTGACCCAACGTGAAGCGGTATGCGCAATTGGGGTCTGTTCCTTGCAAGAGCT
AATCGTAATGACGATGGACCTTCTACAAGTGAGTGCTGTAGTGCCAGCAGCGGACCAG
ATTGTTTTCCGGATGTTCCCCCTCGTGCCGAAGGCTGAACTCAGGGCGGGGCCGTGGGC
GCGCATCGTTGCCACGCAGGTACACCGTGAGGCACCTTGAAGGTCCCGTCATGAAC

>YNT_DQZ12.2

GTCGGGATGCCCTTTCGACACCAGCACGGCGCGACGCTTTCGCGCACGGATCGGATAA
GCTTTTCCGTGGACCACTCGTCCTCAGAAATATCTCGTAGTCTTCTCGTTGGTTATTCCA
ATGAAGCGCCTCGTCTGTGGGGGCACTGAACCGCTAGAGCTATTCGAGTGCTCTACTA
CTCCACCGGGGGCAGCACGCAGCCGTGGGATCGGCACGGCCCACCTCCAGCCAACGAG
AGTTACGAGTACCCGACGCGTTTTCCCGCGTCGGAGGCTGCCAACACGATAGTAACTG
AACTGGCCCAAACGTATTAATACGCCCCGGGGGCAGGCCTCTGGCGCGCCACTGGCTC
AGGCCCGTGGCGTGCCCGCCTCGTCAGCGCCACCCATTGCTAAGCGCTGACAGTAATA
GACCCCTCCATAGTAGTTACCGATGTTAATTCGGTCACCGGCCGAAACGTGCGCGCTCA
GTGCAGGGCAGGTCCTGTAGAACGGAAGGTGGATATTTGGCAAAGGCTGCTGGTGCGC
CTACCAACTTGTTCCTCCGCGCTTGCTAGAGCGACCAACTACGCTCTGTAACGTCCAGT
ACCCGAGCAGTCTCTCAACTTGGCCGATGAGACGGTATACACCGCCGTTGGGACGCGG
ACTCAACAACTCCCTCATACCCATCCGCCCGTCCGGAGCAGAACGACACGGCGGTACC
GGCGTCAGGCCCCCTCGCCTGAACTGCATCATTATGAGGGGAGGTGCGTCGACTGGGG
GCCGGCGACTTGATCCTGGTCGGCCCGTGAAGCCCATCAGTCTCATGGAGCCGTTCTCT
CCCATACATTGGCCTCCTGGGCTTTCCATCAATTGTGTCGAAATATTGTCTAATGTGAA
ATTTATTCATCGTGAGGTGTAAACCGCCCCCGCGTGACTGTAGAGCGTAATCCGGCCTT
CTGGGCTAGCAGCTAACGAGGAAGTGTGCTTCATTTCGTTCGGAAAGGACGAAATGCT
CTAGGCGGCTCTCAACCAGCCTAGTCCCCACGATACATACAACCGTACCAGGTCGAAT
TAATCCGCCGAGGTCCCGTCGCGGGATGTTTGAGGGGGAGATACGTTCCGGCCATATA
CTTTTTGTCCGCATCCCTTGGGGAAATCCATTACGCTGCCGTTGATGTCGTTAACGTCA
CGCCACTAACACCGAACTAGGGGGACCAAACTATACCATGTTATCCGTATACTTACCC
ACTGCCGTGGTTCGGAGTACGCTGCTGTGAGTAATGTACGCGCTCCTCGTGGGGATTTG
TCGTCTGAACTTGAATAACAGCTCACTCGGATGGGATTTAGCAAACCATCCCAACATAT
TCCAGGCCTCTGATCAGATCACAATTTTGCTGTCACCAGTTTCCAATTACGGTTGTGCA
TCGCCGGGAGGCTTGCGATTAGATCTACTCGCATGATTCGAGCGGTAATATCGGGTCTG
AAATTCCCTAACCCAACAGGAGGCGGTACGTGTGGTCGGAATCTGCACCTTGCGAGAG
CTAGTCGTAATGACGATGGCTTTTCCACAAGTGAGTGCTGGGGCGCGAGCGGCGGACC
AGATGGTCTTCCGAATGTTCCCCCCCGCGCTGAAGGCTGAACTCGATGCGCGACCGTA
GGCTCGCACCGTTGCCACGCATGCGCACCGCGAGGTCCCTTGAAGATCCCGTCATCAA
C

>YNT_DQZ19.1

TTCAAGATGCCCTCCTGACTCTGCCACGACGCGGTGGCTTCACGTGTGGACCGGATAA
ACTTTCCATTGGACGGCTCGTTCTCAGAAATATCTCGTAGTCTTCTCGTTGGTTATTCCA
ATGAAGCGCCTCGTCTGTGGGGGCACTGAACCGCTAGAGCTATTCGAGTGCTCTACTA
CTCCACCGGGGGCAGCACGCAGCCGTGGGATCGGCACGGCCCACCTCCAGCCAACGAG
AGTTACGAGTACCCGACGCGTTTTCCCGCGTCGGAGGCTGCCAACACGATAGTAACTG
AACTGGCCCAAACGTATTAATACGCCCCGGGGGCGGGCCTCTGGCGCGCCACTGGATC
AGGCCCGTGGCGTGCCCGCCTCGTCAGCGCCACCCATTGCTAAGCGCTGACAGTAATA
GACCCCTCCATAGTAGTTGCCGATGCTAATTCGGTCACTGGCCGAAACGTATGCGCTCA
GCACAGGGCAGATACTACGGAGCGAAAGGTGGATGATTGGCAGGGGCCGCTGGCGCA
CCTACAAAACTATTCGTCCGCGCCTGCTGGAGCGACCAACTACGCTCTATAGCGTCCAG
TACCCGAGCAGTCCCTCAATTGGTCCGATGAGACTGTATACGCCGCCGTTGGGACGCG
GACTAAACAACCCCCTCATACCCATTCGCCCGTCCAGAGCGGAACGACTCCGCGGTGC
CGGCGTGAGGCCTCCTCGCCTAGGCTGCGCCATTATGTTGGGAGGTGCGTTGACTGGG
GGCCGGCGACCTGAGCTTGGTCGGCCCGTGAAGCCCATCAGTCCCATGGGGACGTTCT
CCCCCATCCATTGGCCTCTGGGGCTTTCCACCAATTGCACCAGAATAATGCCAGATGTT
AGATGTATTCATCGTGGGGCACGAGCCTTCCCCGCGTGAGTGCGGCTCGAAATCCGGT
CCCCCAGGCTGCCAGCTAACACGGAAGTGTGCCTCGTTTCGCTCAGAGGGGACGAGAC

ACTCTCGATGGCTCTCAACAAGCCTAGTCTCCACGATACATACAATCGTGCCAGGTGG
GATTGGTACGCCGGGGTCCCGTCGCAGGATGTCTAGGGGGGAGATACGTTCCGGTCAT
ATACTCTTTGTCCGCATCCCTTGGGGAAATCCATTACGCTGCCGTTGACGTCGTTAACG
CCGCTTCATTAACGTCGAACTAGGGAGACCAAACTATACCATGTAATCCGTGTACTTAC
CCATCGTTGCGGTACAGGATACGGGGCTGTGAGTAATGTACACACTCCTGGTGGGAAC
TTGTCCTCCGGACTTGAATGGTTACTCGCCGAGCTGGGACTTAGCAAACCACCCTGATG
CATTCCAGGTATCAGATTAGAACACAATTTTGCTTTCACCGGTTTCCCCTTACGGTTCC
GCATCGTCGAGGGGCCTTCAACTAAATCTACTCGCGTGGTTCGAGCGGTAATATCGGG
TCTGAAATCTCCTGACCTAACGGGAGGTGGCACGCGCGATCGGAATCTGCAGTGTGTG
GGAGCTAATCGTATTCATGATGACTCCTTCACAAGTGAGTGGTGTGACGCGAGCAGCG
GACCAGCTGATTTTCCGAATGTTCCCCTCCGTACCGAAGGCTTAACTTGAGGCGGAGCC
GTGGGCTCGCACTCCTGCTACGCAGGTGCACCGCGAGGCAACTTGAAGGTCTCGTCAT
GAAG

>YNT_DQZ19.2

GTCGGGATGCCTTCTCGACTCCGGCACGGCGTGGCACCTTCGTGCACGGATGGGATAA
GCTTTCCCGTAGATGGCTCGTTCTCAGAAATATCTCGTAGTCTTCTCGTTGGTTACTCCA
ATGAAGCGCCTCGTCTGCGGGGGCACTGAACCGCGAGAGCTATTCAAGTGCTCTACTA
CTCGACCGGGGGCAGCTTACAACCGCGGGATTGGCACGGTCTACCTCTGTCCAACAGG
GGGTACGACGACCCGGCCCGTTTTCCCGCGTCGGAGGCTGCCAACACAATAGTAACTG
AACTGGCCCAAACGTATTAATACGCCCCGGGGGCGGGCCTCTGGCGCGCCACTGGATC
AGGCCCGTGGCGTGCCCGCCTCGTCAGCGCCACCCATTGCTAAGCGCTGACAGTAATA
GACCCCTCCTTAGTAGTTGCCGATGTTGATTTGGTCACCGGCCGAAACCTATGCGCTCA
GCACAGGGCAGGTACTACGGAGCGAAAGGTGGATGATTTGCAGGGGCCGCTGGCGCA
CCTACAAAACTATTCGTCCGCGCCTGCTGGAGCGACCAACTACACTCTATAGCGTCCAG
TACCCGAGCAGTCTCTCAACTGGTCCGATTAGACTGTATACACCGCCGTTGGGACGCG
GACTAAACAATTCCCTCATACCCATCCGCCCGTCCGGAGCAGAACGACTCGACGGTAC
CGGCGTCAGGCCCCCTCACACAGGCTGAACCATTTTGTTGGGAGGTGCGTCGACTGGG
GGCCGGCAACTTGAGCTTGGTCGGCCCGTGAAACCCACCACTCCCATGGAGAAGTTCT
CTCCCATCCATTGGCCTCCGGGGCTCTTCATCAATCGCGTCGGAGTATCGTCTAATGTG
AAATTTATTCATCGTGAGGTATACACCGCCCCCGCGTGGGTGCGGCTCGAAGCCCGGC
TTCCCAGGCTGGCAGCTAACACGGAAGTGTGCCTCGTTTCGCTCAGAAGGGACGGGAC
ACTCTCGACGGCCCTCAACAAGCCTAGTCCCCACGATACATACAACCGTGCCAGGTGG
AATTAATACGTCGGGGTCCCATCGCGGGATGTTTGAGGGGGAGATACGTTCCGGCCAT
ATACTCCCTGTTCGCGCCCCTCAGAGAAGTCCATTACGCTGCCGTTGACGTCGTCAATG
CCGCGCCACTAACACCGGACTAGGGGGACCAAACCATACCATGTAATCCGTGTACTTG
CCCATTGTTACGGTTTAGGGAACGGGGCTGTGGCTAATAGACGCGCTCCTGGTGGGAG
CTTGTCCTCCGGACTCGAATGGCTACTCGCCGGGCTGGGACTTAGCAAACCACTCTGAC
GCATCTACGGCCTCAGATTAGATCACAATTGTGCTTTGTCCATTTCCTAACTACCGTTCC
GCATCGTCGGGAGGCCTTCGACTAGATTTACTCGCATGGTTTAAGTGATAATATCAAGT
CTGGAATCCTCTGACCCAACGTGAAGCGGTATGCGCAATTGGGGTCTGTTCCTTGCAAG
AGCTAATCGTAATGACGATGGACCTTCTACAAGTGAGTGCTGTAGCGCCAGCAGCGGA
CCAGATTGTTTTCCGGATGTTCCCCCTCGTGCCGAAGGCTGAACTCAGGGCGGGGCCGT
GGGCGCGCATCGTTGCCACGCAGGTACACCGTGAGGCACCTTGAAGGTCCCGTCATGA
AC

>YNT_DQZ24.1

GTCGGGATGCCCTTTCGACACCAGCACGGCGCGACGCTTTCGCGCACGTATCAGATAA
GTTTTCCCGTGGACGGCTCGTCCTCAGAAATATCTCGTAGTCCTTTCGTTGGTTACTCCA
ATGAAGCGCCTCGCCTGCGGGAGCACTAAACCGCGAGCGCCATTCGAGTGCTCTACAA

CTCGACCGGGGGCAGCACACAACCGTAGGATCGGCGCGGCTCATTTTCGGCTAGTAGG
GGGTCCGACGTCCCGGCCCGTTTTCTCGCGTCGGAGGCTGCCAACACGATAGTAACTG
AACTGGCCCAAACGTATTGATACCCCCAGAGGGCGGGTCTCTGGCGCGCCACTGGCTC
AGGCCCGTGGCGTGCCCGCCTCGTCAGCGCCACCCATTGCTAAGCGCTGACAGTAATA
GACCCCTCCATAGTAGTTGCCGATGTTGATTTGGTCACCGGCCGAAACGTATGCGCTCA
GCACAGGGCAGGTACTACGGAGCGAAAGGTGGATGATTGGCAGGGGCCGCTGGCGCA
CCTACAAAACTATTCGTCCGCGCCTGCTGGAGCGACCAACTACGCTCTATAGTGTCCAG
TACCCGAGCAGTCTCTCAACTGGTCCGATTAGACTGGATACACCGCCGTTGGGACGCG
GACTAAACAATTCCCTCATACCCATCCGCCCGTCCGGAGCGGAACGACTCGACGGTAC
CGGCGTCAGGCCCCCTCGCCTAGGCTGCACCATTATGTTGGAAGGTACATCGACTGGG
GGCCGGCAACTTGAGCTTGGTCGGCCCGTGAAACCCACCAGTCCCATGGAGAAGTTCT
CTCCCATCCATTGGCCTCCGGGGCTCTTCATCAAGCGCGTCGGAGTATCGTCTAATGTG
AAATTTATTCATCGTGAGGTATACACCGCCCCCGCGTGGGTGCGGCTCGAAGCCCGGC
TTCCCAGGCTGGCAGCTAACACGGAAGTGTGCCTCGTTTCGCTCAGAAGGGACGGGAC
ACTCTCGACGGCCCTCAACAAGCCTAGTCCCCACGATACATACAACCGTGCCAGGTGG
AATTAATACGTCGGGGTCCCATCGCGGGATGTTTGAGGGGGAGATACGTTCCGGCCAT
ATACTCCCTGTTCGCGCCCCTCAGAGAAGTCCATTACGCTGCCGTTGACGTCGTCAATG
CCGCGCCACTAACACCGGACTAGGGGGACAAAACCATACCATGTAATCCGTGTACTTG
CCCATTGTTACGGTTTAGGGAACGGGGCTGTGGCTAATAGACGCGCTCCTGGTGGGAG
CTTGTCCTCCGGACTCGAATGGCTACTCGCCGGGCTGGGACTTAGCAAACCACTCTGAC
GCATCTACGGCCTCAGATTAGATCACAATCTTGCTTTCTCCATTTCCTAATTACCGTTCC
GCATCGTCGGGAGGCCTTCGACTAGATTTACTCGCATGGTTCAAGTGATAATATCAAGT
CTGGAATCCTCTGACCCAACGTGAAGCGGTATGCGCAATCGGAATCTGCACCTTGCGA
GAGATAATTGTAATGACGATGGACCTTCTACAAGTGAGTGCTGGGGCACGAGCGGCGG
ACCAGATGGTCTTCTGAATGTTCCTTCCCGTGCCGAAGACCGAACCCGAGGCGGAGCC
GTGGGCGTGCACCGTTGCCACGCAGGTGCACCGCGAGGCACCTTGAAGGTCCCGTCAT
GAAG

>YNT_DQZ24.2

GGCGGGATGCCTTCCCAACTCTGGCACGGCGCGGCGCCTTCGCGCACGGACCGGATAA
GCTTTCCCGTGCACGGCTCGTTCTCAGAAATATCTCGAGGTTTTCCCGTTGGCCGCCTT
GATGCCGCGCCGCGTTTGCTTGGATGCTGACCCGCGAGAGCTATTCGAGTGCTCTACTA
CTCGACCGGGGGCAGCACACAGCCGTGGGATCGGCGCGGCCCATCTCCGGCCAACGAA
AGTTATGACGTCCCCGCCCGTTTTCCCGCGTCGGAGGCTGCCAACACGATAGTAACTGA
CCCAGCGCAAACGCATGGATTCGCCCAGAGGGCGGGCCTCTGGCGCGCCACTGGATCA
GGCCCGTGGCGTGCCCGCCTCGTCAGCGCCACCCATTGCTAAGCGCTGACAGTAATAG
ACCCCTCCATAGTAGTTGCCGATGTTGATTCGGTCACCGGCCGAAACGTGCGCGCTCAG
TGCAGGGCAGGTACTGTAGAGCGGAGGATGGATGACTGGCAGGGGCTGCTGACGCGC
CTATCAGCCCGTTCCCCCGCGCCTGCTGTGGCGACCAACTACGCCCCGCAGTGACCGGT
ACCCGAGCAGTCTCTCAACTGGTCCGATTAGACTGTATACACCGCCGTTGGGACGCGG
ACTAAACAACTCCCTCATACCCATCCGCCCGTCCGGAGCGGAACGACTTGGCGGAACC
GGCGTCAGACCCCCTTGCCTAGATGGCACTAATATGTTGGGAGATGCGTCGACTGGGA
TCTGACAACCTGAGCTTGGTCGGCCCGTGAAGCCCATCAGTCTCATGGAGACGTTCTCT
CCCATCCATTGGCCTCCTAGGCTTTCCACCAATTGCGTCAGGGTAATGCCAGATGTTAG
ATGTATTCATCGTGAGGTATACACCGCCCCCGCGTGGGTGCGGCTCGAAGCCCGGCCT
CCCAGGCTGGCTGCTAACACGGAAGTGTACCCGGTTCCACGCAGAGGGCATGAGGCAG
TACCTACGGATATCAACAAGCCTAGTCCCCATGATACGTACAGCTGGGCCAGGTGGGA
TCAATACGTCGGAAGCCCGTCGCGGGATGTCCAGGGGGGAGATACGTTCCGGCCATAT
ACTCCCTGTCCACATCCCTTGAGGAAATCCATTACGCTGCCGTTGACGTCGTTAACGCC
ACGCCACTAACACCGAACTAGGGGGACCAAACTATACCATGTTATCCGTATGCCCAGT
TGTTGTTGCGGTTCAGGGTACGGGGCTGTGAGGCACGGGCGCGCTCCTGGTGGGAACT

TGTCCTCCGGACTTTAATGGCTACTCGCCGGGCTGAGACTTAGCAGACCACCCTGACGC
ATTCCAGGCCTCTGATCAGATCACAATTTTGCTGTCACCAGTTTCCAATTACGGTTCCG
CATCGCCGGGAGGCCTGCGATTAGATCTACTCGCATGGTTCGAGCGGTAATATCGGGT
TTGAAATCCCCTGACCCAACGGGGGACGGTATGCGCAATCAGGATCTGCACCTTTCGG
GAGCTAATCATAATGACGATGGCTCTTCCACAAGTGAGTGGTGTAGCGCCAGCGGCGG
ACCAGACGGTCTTCCGAATGTTCCCCCCCGTGCCGAAGGCTGAACTCAAGGCGGGGCC
GTGGGCGTGCCCCGTTGCCACGCAGGTGCACCGCGAGGCACCTTGAAGGTCCCGTCAT
GAAG

>YNT_DQZ25.1

GTCGGGATGCCTTCTCGACTCCGGCACGGCGTGGCACCTTCGTGCACGGATGGGATAA
GCTTTCCCGTAGATGGCTCGTTCTCAGAAATATCTCGTAGTCTTCTCGTTGGTTACTCCA
ATGAAGCGCCTCGTCTGCGGGGGCACTGAACCGCGAGAGCTATTCAAGTGCTCTACTA
CTCGACCGGGGGCAGCTTACAACCGCGGATTTGCACGGTCTACCTCTGTCCAACAGG
GGGTACGACGACCCGGCCCGTTTTCCCGCGTCGGAGGCTGCCACCACGATAGTAACTG
AACTGGCCCAAACGTATTAATACGCCCCGGGGGCGGGCCTCTGGCGCGCCACTGGATC
AGGCCCGTGGCGTGCCCGCCTCGTCAGCGCCACCCATTGCTAAGCGCTGACAGTAATT
GACCCCTCCATAGTAGTTGCCGATGTTGATTTGGTCACCGGCCGAAACGTATGCGCTCA
GCACAGGGCAGGTACTACGGAGCGAAAGGTGGATGATTGGCAGGGGCCGCTGGCGCA
CCTACAAAACTATTCGTCCGCGCCTGCTGGAGCGACCAACTACGCTCTATAGCGTCCAG
TACCCGAGCAGTCCCTCAACTGATCCGATGAGACTTTATACACCGCCGGTGGGACGCG
GACTACACAACCCCCTCATACCCATTCGCCCGTCCGGAGCAGAACAACACGGCGGTAC
CGGCGTCAGGCCCCCTCGCCTAGGCTGCACCATTATATTGGGAGGTGCGTCGATTGGG
GGTTGGCGACTTGATCCCGGTCGGCCCGTGAAGCCCATCAGTCCCGTGGAGACGTTCTC
TCCCATCCATTGGCCTCCGGAGCTCTTCATCAATCGCACCGGAGTATTGCCAGATGTTA
AGCGTATTCATCGTAGAGCACAAGCCGCCTTCGCATGGGTACAGCGCGTAATCCGGCC
CCCCAGGCTGCCTGCTAACACGGATGTGTACCCGGTTCCACTCAGAGGGCATGAGGCA
GTACCTACGGATCTCAACAAGTCTAGCTCGCACAATACATATAACCATGCCCGGTGGG
ATTAATACGCCGGGGTCTCATCGCGGGATGTTTGAGGGGGAGATACGTTCCAGCCATA
TACTCCCTGTTCGCGCCCCTCAGAGAAGTCAATTACGCTGCCGTTGACGTCGTCAATGC
CGCGCCACTAACACCGGACTAGGGGGAGAAAACCATACCATGTAATCCGTGTACTTGC
CCATTGTTGCGGTTCAGGGTACGGGGCTATGAGTAATGGGCACACTCCTGGTAGGAAC
TTGGTCCCCGGCTTTGAATGGCTACTCGCCGAGCTCGGCCTTAGCAAACCACCCTGACG
CATCCCAGGCCTTTGATCGGATCACAATTTTGCTTTCACCAGTTCCCAATTACGGTTCC
GCATCGTCGGGAGGCCTTCGACTAGATCTACTCGCATGGTTCGAGCGGTAATATCGGG
TCTGAAATCCCCCGACCCGACGGGAGGCGGTACGTGTGGTCGGAATCTGCACCTTGCG
AGAGCTAATCGTAATGACGATGGCTCTTCCACAAGTGAGTGCTGGGGCGCGAGCGGCG
GACCAGATTGTCTTCCGAATGTTTCCCCCCGTGCCGAAGGCTGAACTCGAGGTGGAGC
AGTGGGCGCGTACCGTCGACATGCAGGTGCACTATGAGGTCCCTTGAAGGTCCCGTCA
TGAAG

>YNT_DQZ25.2

GTCGGGATGCCCTTTCGACACCAGCACGGCGCGACGCTTTCGCGCACGGATCGGATAA
GCTTTTCCGTGGACCACTCGTCCTCAGAAATATCTCGTAGTCTTCTCGTTGGTTATTCCA
ATGAAGCGCCTCGTCTGTGGGGGCACTGAACCGCTAGAGCTATTCGAGTGCTCTACTA
CTCCACCGGGGGCAGCACGCAGCCGTGGGATCGGCACGGCCCACCTCCAGCCAACGAG
AGTGACGAGTACCCGACGCGTTTTCCCGCGTCGGAGGCTGCCAACACGATAGTAACTG
AACTGGCCCAAACGTATTAATACGCCCCGGGGGCAGGCCTCTGGCGCGCCACTGGCTC
AGGCCCGTGGCGTGCCCGCCTCGTCAGCGCCACCCATTGCTAAGCGCTGACAGTAATA
GACCCCTCCATAGTAGTTACCGATGTTAATTCGGTCACCGGCCGAAACGTGCGCGCTCA

GTGCAGGGCAGGTCCTGTAGAACGGAAGGTGGATATTTGGCAAAGGCTGCTGGTGCGC
CTACCAACTTGTTCCTCCGCGCTTGCTAGAGCGACCAACTACGCTCTGTAACGTCCAGT
ACCCGAGCAGTCTCTCAACTTGGCCGATGAGACTGTATACACCGCCGTTGGGACACGG
ACTAAACAACTCCCTCATACCCATCCGCCCGTCCGGAGCGAAAGGACTCGGCAATACC
GACGTCAGGGCCCCTCGCCTGAACTGCATCATTATGATGGGAGGTGCGTCGACTGGGG
GCCGGCGACTTGATCCCGGTCGGCCCGTGAAGCCCATCAGTCTCATGGAGCCGTTCTCT
CCCATACGTTGGCCTCCTGGGCTTTCCATCAATTGTGTCGAAATATTGTCTAATGTGAA
ATTTATTCATCGTGAGGTGTAAACCGCCCCCGCGTGACTGTAGAGCGTAATCCGGCCTT
CTGGGCTAGCAGCTAACGAGGAAGTGTGCTTCATTTCGTTCGGAAAGGACGAAATGCA
CTAGGCGGCTCTCAACCAGCCTAGTCCCCACGATACATACAACCGTACCAGGTCGAAT
TAATCCGCCGAGGTCCCGTCGCGGGATGTTTGAGGGGGAGATACGTTCCGGCCATATA
CTTTTTGTCCGCATCCCTTGGGGAAATCCATTACGCTGCCGTTGATGTCGTTAACGTCA
CGCCACTAACACCGAACTAGGGGGACCAAACTATACCATGTTATCCGTATACTTACCC
ACTGCCGTGGTTCGGAGTACGCTGCTGTGAGTAATGTACGCGCTCCTCGTGGGGATTTG
TCGTCTGAACTTGAATAACAGCTCACTCGGATGGGATTTAGCAAACCATCCCAACATAT
TCCAGGCCTCTGATCAGATCACAATTTTGCTGTCACCAGTTTCCAATTACGGTTGTGCA
TCGCCGGGAGGCTTGCGATTAGATCTACTCGCATGATTCGAGCGGTAATATCGGGTCTG
AAATTCCCTAACCCAGCAGGAGGCGGTACGTGTGGTCGGAATCTGCACCTTGCGAGAG
CTAGTCGTAATGACGATGGCTTTTCCACAAGTGAGTGCTGGGGCGCGAGCGGCGGACC
AGATGGTCTTCCGAATGTTCCCCCCCGCGCTGAAGGCTGAACTCGATGCGCGACCGTA
GGCTCGCACCGTTGCCACGCATGCGCACCGCGAGGTCCCTTGAAGATCCCGTCATCAA
C

>YNT_DQZ26.1

GTCGGGACGCCCTCCCGGCTCTGGCATGGCGCGGTGGCTTTGCGCACGGATCGGATAA
ACTTTCCATTGGACGGCTCGTTCTCAGAAATATCTCGTAGTCTTCTCGTTGGTTACTCCA
ATGAAACGCCTCGTCTGCGGGGGCACTGAACCGCGAGAGCTATTCAAGTGCTCTACTA
CTCGACCGGAGGCAGCTTACAACCGTGGGATCGGCGCGGCCCACCTCCGGTCAACGAG
AGTTACGAGTACCTGGTCCGTTTTCCCGCGTCGGAGGCTACCAACACGATAGTAACTG
AACCAGCGCGAGAGCATTGTTACGCCTCGGGGGCGGGCCTCTGGCGCGCCACTGGATC
AGGCCCGTGGCGTGCCCGCCTAGTCAGCGCCACCCATTGCTAAGCGCCGACAGTAATA
GACCCCTCCATAGTAGTTGCCGATGTTGATTTGGTCACCGGCCAAAACGTATGCGCTCA
GCACAGGGCAGGTACTACGGAGCGAAAGGTGGATGATTGGCAGGGGCCGCTGGCGCA
CCTACAAAACTATTCGTCCGCGCCTGTCGGAGCGACCAACTACGCTCTATAGCGTCCAG
TACCCGAGCAGTCCCTCAATTGGTCCGATGAGACTGTATACGCCGCCGTTGGGACGCG
GCCTAAATAACCCCTTTACGCACATCCGCCCATCCGGAGCGGAACGACTCGGCGGTAC
CGGCGTCAGGCCCCCTCGCCTAGACTGCACCATTATGTTGGGAGGTGCGTCGACTGGG
GGCCGGTGACTTGATCCTGGTCGGCCCGTGAGGCCCATCAGTCCTATGGAGACGTTCTC
TCCCATCCATTGGCCTCCTAGGCTTTCCACCAAGCGCACCGGAGTCTTGTCTAATATGA
AATTTATTCATCGTGAGGTATAAACCGCCCCCGAGTGGGGGCGGCTTGAAGTCCAGCC
CCCCAGGCCGCCTGCTAACACGGAAGTGTACCCGGTTCCACTCAGAGGGCATGAGGCA
GTACCTACGGATCTCAACAAGCCTAGTCCCCATGATACGTACAGCTGGGCCAGGTGGG
ATTAATACGCCGGGGTTCCGTTGCGGAACGGCTAGAAGGTAAATACGCCTTGGCCATA
TACTCTTTGTCCGCATCCCTTGGGGAAATCCATTATGCTGCCGTTGACGTCGTCAACGT
CACGCCACTAACACCGAACTAGGGGGACCAAACTATACTATGTTATCCGTATACTTAC
CCACTGTTGCAGGTCAAGGTATGGGGCTGCAAGTAATGTACATACTCCTGGTGGGAAC
GTGTCCTCCGGACTTGAATGGCTACCTGCCGGGCTGGGACTTAGCAAACCACCCTGAC
GCATCCCAGGCCTTTGATCGGATCACAATTTTGCTTTCACCAGTTCCCAATTACGGTTC
CGCATCGTCGGGAGTCCTTCGACTAGATCTACTCGCATGGTTCGAGCAGTAATATCAAG
GCTGAAATCCCCTGACCCAACGGGGGGCAGTATGCGCAATCGGAATCTGCACCTTGCG
AGAGATAATTGTAATGACGATGGACCTTCTACAAGTGAGTGCTGGGGCACGAGCGGCG

GACCAGATGGTCTTCTGAATGTTCCTTCCCGTGCCGAAGACCGAACCCGAGGCGGAGC
CGTGGGCGTGCACCGTTGCCACGCAGGTGCACCGCGAGGCACCTTGAAGGTCCCGTCA
TGAAG

>YNT_DQZ26.2

GTCGGGATGCCCTTTCGACACCAGCACGGCGCGACGCTTTCGCGCACGTATCAGATAA
GTTTTCCCGTGGACGGCTCGTCCTCAGAAATATCTCGTAGTCCTTTCGTTGGTTACTCCA
ATGAAGCGCCTCGCCTGCGGGAGCACTAAACCGCGAGCGCCATTCGAGTGCTCTACAA
CTCGACCGGGGGCAGCACACAACCGTAGGATCGGCGCGGCTCATTTTCGGCTAGTAGG
GGGTACGACGTCCCGGCCCGTTTTCTCGCGTCGGAGGCTGCCAACACGATAGTCACTG
AACTGGCCCAAACGTATTGATACGCCCAGGGGGCGGGTCTCTGGCGCGCCACTGGATC
AGGCCCGTGGCGTGCCCGCCTCGTCAGCGCCCCCCATTGCTAAGCGCTGACAGTAATA
GACCCCTCCATAGTAGTTGCCGATGCTAATTCGGTCACTGGCCGAAACGTATGCGCTCA
GCACAGGGCAGGTACTACGGAGCGAAAGGTGGATGATTGGCAGGGGCCGCTGGCGCA
CCTACAAAACTATTCGTCCGCGCCTTCTGGAGCGACCAACTACGCTCTATAGCGTCCAG
TACCCGAGCAGTCTCTCAACTGGTCCGATTAGACTGGATACACCGCCGTTGGGACGCG
GACTAAACAATTCCCTCATACCCATCCGCCCGTCCGGAGCGGAACGACTCGACGGTAC
CGGCGTCAGGCCCCCTCGCCTAGGCTGCACCATTATGTTGGAAGGTACATCGACTGGG
GGCCGGCAACTTGAGCTTGGTCGGCCCGTGAAACCCACCAGTCCCATGGAGAAGTTCT
CTCCCATCCATTGGCCTCCGGGGCTCTTCATCAATCGCGTCGGAGTATCGTCTAATGTG
AAATTTATTCATCGTGAGGTATACACCGCCCCCGCGTGGGTGCGGCTCGAAGCCCGGC
TTCCCAGGCTGGCAGCTAACACGGAAGTGTGCCTCGTTTCGCTCAGAAGGGACGGGAC
ACTCTCGACGGCCCTCAACAAGCCTAGTCCCCACGATACATACAACCGTGCCAGGTGG
AGTTAATACGTCGGGGTCCCATCGCGGGATGTTTGAGGGGGAGATACGTTCCGGCCAG
ATACTCCCTGTTCGCGCCCCTCAGAGAAGTCCATTACGCTGCCGTTGACGTCGTCAATG
CCGCGCCACTAACACCGGACTAGGGGGACCAAACCATACCATGTAATCCGTGTACTTG
CCCATTGTTACGGTTTAGGGAACGGGGCTGTGGCTAATAGACGCGCTCCTGGTGGGAG
CTTGTCCTCCGGACTCGAATGGCTACTCGCCGGGCTGGGACTTAGCAAACAACTCTGAC
GCATCTACGGCCTCAGATTAGATCACAATTTTGCTTTCTCCATTTCCTAATTACCGTTCC
GCATCGTCGGGAGGCCTTCGACTAGATTTACTCGCATGGTTTAAGTGATAATATCGGGT
CGGGAATCCTCTGACCCAACGTGAAGCGGTATGCGCAATTGGGGTCTGTTCCTTGCAA
GAGCTAATCGTAATGACGATGGACCTTCTACAAGTGAGTGCTGTAGCGCCAGCAGCGG
ACCAGATTGTTTTCCGGATGTTCCCCCTCGTGCCGAAGGCTGAACTCAGGGCGGGGCC
GTGGGCGCGCATCGTTGCCACGCAGGTACACCGTGAGGCACCTTGAAGGTCCCGTCAT
GAAC

>SCT_LTZ201.1

GTCGGGACGCCCTCCCGGCTCTGGCACGGCGCGGTGGCTTTGCGCACGGATCGGATAA
ACTTTCCATTGCACCGCTCAACCCCGGAAATTCCCCGAGGTTTTCCCAGTGGTTACCCT
GATGCCGCGCAGCGTCTGCTTGGATGCTGAACCGCGAGAGCTATTCAGGTGCTCGACT
ACTCGACCGGGAGCAGCACGCAGCCGCGGGAACGACGCGGTCTACCTCCGGCCAGCA
GGGGGTACGACGTCCCGGCCCGTTTTCCCGCGTCGGAGGCTGCCAACACGATAGTAAC
TGAAATGGCCCAAACGTATTAATACGCACAGGGGGCGGGCCTCTGGCGCGCCATTGGA
TCAGGCCCGTGGCGTGCCCGCCTCGTCAGCGCCACCCATTGCTAAGCGCCGACAGTAA
TAGACCCCTCCATAGTAGTTGCCGATGTTGATTTGGTCACCGGCCGAAACGTATGCGCT
CAGCACAGGGCAGGTACTACGGAGCGAAAGGTGGATGATTGGCAGGGGCCGCTGGCG
CACCTACAAAACTATTCGTCCGCGCCTGCTGGAGCGACCAACTACGCTCTATAGCGTCC
AGTACCCGAGCAGTCTCTCAACTGGTCCGATTAGACTGTATACACCGCCGTTGGGACG
CGGACTAAACAATTCCCTCATACCCATCCGCCCGTCCGGAGCGGAACGACTCGACGGT
ACCGGCGTCAGGCCCCCTCGCCTAGGCTGCACCATTATGTTGGAAGGTACATCGACTG

GGGGCCGGCAACTTGAGCTTGGTCGGCCCGTGAAACCCCCCAGTCCCATGGAGAAGTT
CTCTCCCATCCATTGGCCTCCGGGGCTCTTCATCAATCGCGTCGGAGTATCGTCTAATG
TGAAATTTATTCATCGTGAGGTATACACCGCCCCCGCGTGGGTGCGGCTCGAAGCCCG
GCTTCCCAGGCTGGCAGCTAACACGGAAGTGTGCCTCGTTTCGCTCAGAAGGGACGGG
ACACTCTCGACGGCCCTCAACAAGCCTAGTCCCCACGATACATACAACCGTGCCAGGT
GGAATTAATACGTCGGGGTCCCATCGCGGGATGTTTGAGGGGGAGATACGTTCCGGCC
ATAGACTCCCTGATCGCGCCCCTCAGAGAAGTCCATTACGCTGCCGTTGACGTCGTCAA
TGCCGCGCCACTAACACCGGACTAGGGGGACCAAACCATACCATGTAATCCGTGTACT
TGCCCATTGTTACGGTTTAGGGAACGGGGCTGTGGCTAATAGACGCGCTCCTGGTGGG
AGCTTGTCCTCCGGACTCGAATGGCTACTCGCCGGGCTGGGACTTAGCAAACAGCTCT
GACGCATCTACGGCCTCAGATTAGATCACAATCGTGCTTTCTCCATTTCCTAATTACCG
TTCCGCATCGTCGGGAGGCCTTCGACTAGATTTACTCGCATGGTTTAAGTGATAATATC
GGGTCGGGAATCCTCTGACCCAACGTGAAGCGGTATGCGCAATTGGGGTCTGTTCCTT
GCAAGAGCTAATCGTAATGACGATGGACCTTCTACAAGTGAGTGCTGTAGCGCCAGCA
GCGGACCAGATTGTTTTCCGGATGTTCCCCCTCGTGCCGAAGGCTGAACTCAGGGCGG
GGCCGTGGGCGCGCATCGTTGCCACGCAGGTACACCGTGAGGCACCTTGAAGGTCCCG
TCATGAAC

>SCT_LTZ201.2

GTCGGGACGCCCTCCCGGCTCTGGCACGGCGCGGTGGCTTTGCGCACGGATCGGATAA
ACTTTCCATTGGACCGCTCAACCCCGGAAATACCCCGAGGTTTTCTCAGTGGTTACCCT
GATGCCGCGCAGCGTCTGCTTGGATGCTGAACCGCGAGAGCTATTCAGGTGCTCGACT
ACTCGACCGGGAGCAGCACGCAGCCGCGGGAACGACGCGGTCTACCTCCGGCCAGCA
GGGGGTACGACGTCCCGGCCCGTTTTCCCGCGTCGGAGGCTGCCAACACGATAGTAAC
TGAACTGGCCCAAACGTATTAATACGCACAGGGGGCGGGCCTCTGGCGCGCCACTGGA
TCAGGCCCGTGGCGTGCCCGCCTCGTCAGCGCCACCCATTGCTAAGCGCTGACAGTAA
TAGACCCCTCCATAGTAGTTGCCGATGTTGATTTGGTCACCGGCCGAAACGTATGCGCT
CAGCACAGGGCAGGTACTACGGAGCGAAAGGTGGATGATTGGCAGGGGCCGCTGGCG
CACCTACAAAACTATTCGTCCGCGCCTGCTGGAGCGACCAACTACGCTCTATAGCGTCC
AGTACCCGAGCAGTCTCTCAACTGGTCCGATTAGACTGTATACACCGCCGTTGGGACG
CGGACTAAACAATTCCCTCATACCCATCCGCCCGTCCGGAGCGGAACGACTCGACGGT
ACCGGCGTCAGGCCCCCTCGCCTAGGCTGCACCATTATGTTGGAAGGTACATCGACTG
GGGGCCGGCAACTTGAGCTTGGTCGGCCCGTGAAACCCACCAGTCCCATGGAGAAGTT
CTCTCCCATCCAGTGGCCTCCGGGGCTCTTCATCAATCGCGTCGGAGTATCGTCTAATG
TGAAATTTATTCATCGTGAGGTATACACCGCCCCCGCGTGGGTGCGGCTCGAAGCCCG
GCTTCCCAGGCTGGCAGCTAACACGGAAATGTGCCTCGTTTCGCTCAGAAGGGACGGG
ACACTCTCGACGGCCCTCAACAAGCCTAGTCCCCACGATACATACAACCGTGCCAGGT
GGAATTAATACGTCGGGGTCCCATCGCGGGATGTTTGAGGGGGAGATACGTTCCGGCC
ATATACTCCCTGTTCGCGCCCCTCAGAGAAGTCCATTACGCTGCCGTTGACGTCGTCAA
TGCCGCGCCACTAACACCGGACTAGGGGGACCAAACCATACCATGTAATCCGTGTACT
TGCCCATTGTTACGGTTTAGGGAACGGGGCTGTGGCTAATAGACGCGCTCCTGGTGGG
AGCTTGTCCTCCGGACTCGAATGGCTACTCGCCGGGCTGGGACTTAGCAAACCACTCTG
ACGCATCTACGGCCTCAGATTAGATCACAATTTTGCTTTCTCCATTTCCTAATTACCGTT
CCGCATCGTCGGGAGGCCTTCGACTAGATTTACTCGCATGGTTTAAGTGATAATGCCGG
GTCGGGAATCCTCTGACCCAACGTGAAGCGGTATGCGCAATTGGGGTCTGTTCCTTGCA
AGAGCTAATCGTAATGACGATGGACCTTCTACAAGTGAGTGCTGTAGCGCCAGCAGCG
GACCAGATTGTTTTCCGGATGTTCCCCCTCGTGCCGAAGGCTGAACTCAGGGCGGGGC
CGTGGGCGCGCACCGTTGCCACGCAGGTACACCGTGAGGCACCTTCAAGGTCCCGTCA
TGAAC

>SCT_LTZ203.1

GTCGGGACGCCCTCCCGGCTCTGGCACGGCGCGGTGGCTTTGCGCACGGATCGGATAA
ACTTTCCATTGGACGGCTCGTTCTCAGAAATATCTCGTAGTCTTCTCGTTGGTTACTCCA
ATGAAACGCCTCGTCTGCGGGGGCACTGAACCGCGAGAGCTATTCAAATGCTCTACTA
CTCGACCGGGGGCAGCTTACAACCGTGGGATCGGCGCGGCCCACCTCCGGTCAACGAG
AGTTACGAGTTCCCGGCCCGTTTTCTCGCGTCGGAGGCTGCCAACACGATAGTAACTGA
ACTGGCCCAAACGTATTAATACGCCCCGGGGGCGGGCCTCTGGCGCGCCACTGGATCA
GGCCCGTGGCGTGCCCGCCTCGTCAGCGCCACCCATTGCTAAGCGCTGACAGTAATAG
ACCCCTCCATAGTAGTTGCCGATGTTGATTCGGTCACCGGCCGAAACGTATGCACTTAG
CACAGGGCAGGTACTACAAAGCGAGAGGGGGATGATTGGCAGGGGCTGCTGACGCGC
CTATCAGCCCGTTCCCCCGCGCCTGCTGTGGCGACCAACTACGCCCCGCAGCGTCCAGT
ACCCGAGCAGTCTCTCAACTGGTCCGATTAGACTGTATACACCGCCGTTGGGACGCGG
ACTAAACAACTCCCTCATACCCATCCGCCCGTCCGGAGCGGAACGACTCGGCGGTACC
GGCGTCAGGCCCCCTCGCCTAGACTGCACCATTATGTTGGGAGGTGCGTCGACTGGGG
GCCGGCGACTTGATCCTGGTCGGCCCGTGAAGCCCATCAGTCCCATGGAGACGTTCTCT
CCCATCTATTGGCCTCCGGGGCTCTCCACCAATCGCACCGGAGTCTTGTCTAATATGAA
ATTTATTCATCGTGAGGTATAAACCGCCCCCGAGTGGGTGCGGCTTGAAGTCCAGCCCC
CCAGGCCGCCTGCTAACACGGAAGTGTACCCGGTTCCACTCAGAGGGCATGAGGCAGT
ACCTACGGATCTCAACAAGCCTAGTCCCCATGATACGTACAGCTGGGCCAGGTGGGAT
TAATACGCCGGGGTTCCGTTGCGGAACGGCTAGAAGGTAAATACGCCTTGGCCATATA
CTCTTTGTCCGCATCCCTTGGGGAAATCCATTATGCTGCCGTTGACGTCGTCAACGTCA
CGCCACTAACACCGAACTAGGGGGACCAAACTATACCATGTTATCCGTATACTTACCC
ACTGTTGCAGGTCAAGGTATGGGGCTGCAAGTAATGTACATACTCCTGGTGGGAACTT
GTCCTCCGGACTTGAATGGCTACCTGCCGGGCTGGGACTTAGCAAACCACCCTGACGC
ATCCCAGGCCTTTGATCGGATCACAATTTTGCTTTCACCAGTTCCCAATTACGGTTCCG
CATCGTCGGGAGGCCTTCGACTAGATCTACTCGCATGGTTCGAGCAGTAATATCGGGTC
TGAAATCCCCTGACCCAACGGGGGGCAGTATGTGTGGTCGGAATCTGCACCTTGCGAG
AGATAATCGTAATGACGATGGACCTTCTACAAGTGAGTGCTGGGGCACGAGCGGCTGA
CCAGATTGTCTTCCGAATGTTCCCCCCCGTGCCGAAGGCTGAACCCGAGGCGGAGCCG
TGGGCGTGCACCGTTGCCACGCAGGTGCACCGCGAGGCACCTTGAAGGTCCCGTCATG
AAG

>SCT_LTZ203.2

GTCGGGATGCCTTCCCAACTCCGGCACGGCGCGGCGCCTTCGCGCACGGATCAGATAA
GTTTTCTCGTGGACCGCTCAACCCCGGAAATACCCCGTAGTTTTCCCGTTGGTTACCCT
GATGCCGCGCCGCGTCTGCTTGGATGCTGAACCGCGAGAGCTATTCAGGTGCTCGACT
ACTCGACCGGGGGCAGCACGGCGCCGCGGGAACGACGCGGTCTACCTCCGGCCAGCA
GGGGGTACGACGTCCCGGCCCGTTTTCCCGCGTCGGAGGCTGCCAACACGATAGTAAC
TGAAATGGTCCAAACGTATTAATACGCACAGGGGGCGGGCCTCTGGCGCGCCACTGGA
TCAGGCCCGTGGCGTGCCCGCCTCGTCAGCGCCACCCATTGCTAAGCGCTGACAGTAA
TAGACCCCTCCATAGTAGTTGCCGATGTTGATTCGGTCACCGGCCGAAACGTATACACT
CGGTACGGGGTGGGTACCACGGAGCGAAAGGTGGATGATTGGCAGGAGCCGCTGGCG
CACCTACAAAACTATTCGTTCGCGCCTGCTGGAACGACCAACTACGCTCTATAGCGTCC
AGTACTCGAGTAGTCCCTCAATTGGTCCGATGAGACGGTATACGCCGCCGTTGGGACG
CGGCCTAAATAACCCCTTTACGCACATCCGCCCATCCGGCGCGGAACGACTCGGCGGT
ACCGGCGTCAGGCCCCCTCGCCTAGACTGCACCATTATGTTGGGAGGTGCGTCGACTG
GGGGCCGGTGACTTGATCCTGGTCGGCCCGTGAGGCCCATCAGTCCTATGGAGACGTT
CTCTCCCATCCATTGGCCTCCTAGGCTTTCCACCAATTGCGTCAGGGTAATGCCAGATG
TTAGATGTATTCATCGTGAGGTATACACCGCCCCCGCGTGGGTGCGGCTCGAAGCCCG
GCCTCCCAGGCTGGCTGCTAACACGGAAGTGTAGCCGGTTCCACTCAGAGGGCATGAG
GCAGTACCTACGGATATCAACAAGCCTAGTCCCCATGATACGTACAGCTGGGCCAGGT
GGGATCAATACGTCGGAAGCCCATCGCGGGATGTTTGAGGGGGAGATCCGTTCCGGCC

ATATACTCCCTGTCCGCATCCCGTGGGGTAATCCATTACGCCGTCGTTGACGTCATCAA
TGCCGCGCGATTAACACCGAGCTGATGGGACCAAAACATACCAGGTAATCCGTGTACT
TACCCATCGTTGCGGTACAGGATACGGGGCTGTGAGTAATGTACACACTCCTGGAGGG
AGCTTGTCCTCCAGACTTGAATGGCTACCTGCCGGGCTGGGACTTAGCAAACCACCCTG
ACGCATCCCAGGCCTTTGATCGGATCACAATTTTGCTTTCACCAGTTCCCAATTACGGT
TCCGCATCGTCGGGAGGCCTTCGACTAGATCTACTCGCATGGTTCGAGCAGTAATATCG
GGTCTGAAATCCCCTGACCCAACGGGGGGCAGTATGTGTGGTCGGAATCTTCAGTTTGT
GGGAGCTAATCGTAATGACGATGGACCTTCTTGAAGTGAGTGCTGTAGTGCCAGCAGC
GGACCAGATTGTTTTCCGGATGTTCCCCCTCGTGCCGAAGGCTGAACTCAGGGCGGGG
CCGTGGGCGCGCACCGTTGCCACGCAGGTACACCGTGAGGCACCTTGAAGGTCCCGTA
ATGAAC

>SCT_LTZ206.1

GTCGGGTTGCCCTTTCGACACCAGCACGGCGCGGCGCTTTCGCGCACGTATCAGATGG
GCTTTCCCGTGGACGGATTGTCCTCAGAAGCATTTCGAGGTTTTCTCAGTGGCCGCCCT
GATGCCGCGCAGCGTCTGCTTGGATGCTGAACCGCGAGAGCTATTCAGGTGCTCGACT
ACTCGACCGGGAGCAGCACGCAGCCGCGGAACGACGCGGTCTACCTCCGGCCAGCA
GGGGGTACGACGTCCCGGCCCGTTTTCCCGCGTCGGAGGCTGCCAACACGATAGTAAC
TGAAATGGCCCAAACGTATTAATACGCACAGGGGGCGGGCCTCTGGCGCGCCACTGGA
TCAGGCCCGTGGCGTGCCCGCCTCGTCAGCGCCACCCATTGCTAAGCGCCGACAGTAA
TAGACCCCTCCATAGTAGTTGCCGATGTTGATTTGGTCACCGGCCGAAACGTATGCGCT
CAGCACAGGGCAGGTACTACGGAGCGAAAGGTGGATGATTGGCAGGGGCCGCTGGCG
CACCTACAAAACTATTCGTCCGCGCCTGCTGGAGCGACCAACTACGCTCTATAGCGTCC
AGTACCCGAGCAGTCTCTCAACTGGTCCGATTAGACTGTATACACCGCCGTTGGGACG
CGGACTAAACAATTCCCTCATACCCATCCGCCCGTCCGGAGCGGAACGACTCGACGGT
ACCGGCGTCAGGCCCCCTCGCCTAGGCTGCACCATTATGTTGGAAGGTACATCGACTG
GGGGCCGGCAACTTGAGCTTGGTCGGCCCGTGAAACCCACCAGTCCCATGGAGAAGTT
CTCTCCCATCCAGTGGCCTCCGGGGCTCTTCATCAATCGCGTCGGAGTATCGTCTAATG
TGAAATTTATTCATCGTGAGGTATACACCGCCCCCGCGTGGGTGCGGCTCGAAGCCCG
GCTTCCCAGGCTGGCAGCTAACACGGAAGTGTGCCTCGTTTCGCTCAGAAGGGACGGG
ACACTCTCGACGGCCCTCAACAAGCCTAGTCCCCACGATACATACAACCGTGCCAGGT
GGAATTAATACGTCGGGGTCCCATCGCGGGATGTTTGAGGGGGAGATACGTTCCGGCC
ATAGACTCCCTGTTCGCGCCCCTCAGAGAAGTCCATTACGCTGCCGTTGACGTCGTCAA
TGCCGCGCCACTAACACCGGACTAGGGGGACAAAACCATACCATGTAATCCGTGTACT
TGCCCATTGTTACGGTTTAGGGAACGGGGCTGTGGCTAATAGACGCGCTCCTGGTGGG
AGCTTGTCCTCCGGACTCGAATGGCTACTCGCCGGGCTGGGACTTAGCAAACCACTCTG
ACGCATCTACGGCCTCAGATTAGATCACAATTTTGCTTTCTCCATTTCCTAATTACCGTT
CCGCATCGTCGGGAGGCCTTCGACTAGATTTACTCGCATGGTTTAAGTGATAATATCGG
GTCGGGAATCCTCTGACCCAACGTGAAGCGGTATGCGCAATTGGGGTCTGTTCCTTGCA
AGAGCTAATCGTAATGACGATGGACCTTCTACAAGTGAGTGCTGTAGCGCCAGCAGCG
GACCAGATTGTTTTCCGGATGTTCCCCCTCGTGCCGAAGGCTGCACTCAGGGCGGGGCC
GTGGGCGCGCATCGTTGCCACGCAGGTACACCGTGAGGCACCTTCAAGGTCCCGTCAT
GAAC

>SCT_LTZ206.2

GTCGGGACGCCCTCCCGGCTCTGGCACGGCGCGGTGGCCTTGCGCACGGATCGGATAA
ACTTTCCATTGGACCGCTCAACCCCGGAAATACCCCGAGGTTTTCTCAGTGGTTACCCT
GATGCCGCGCCGCGTTTGCTTGGATGCTGAACCGCGAGAGCCATTCGAGTGCTCTACA
ACTCGACCGGGGGCAGCACACAACCGTGGGATCGGTGCGACCCTCCTCCGGCCAACGA
GAGTTACGGCGTCCCGGCCCGTTTTCCCGCGTCGGCGGCTGCCACCCCGATAGTAACTG

AACTGGCCCAAACGTATTAATATGCCCCGGGGGCGGGCCTCTGGCGCGCCACTGGATC
AGGCCCGTGGCGTGCCCGCCTCGTTAGCGCCACCCATTGTTAAGCGCTGACAGTAATA
GACCCCTCCATAGTAGTTGCCGATGTTAATTCGGTCACCGGCCGAAACGTGCGCGCTCA
GTGCAGGGCAGGTCCTGTAGAACGGAGGGTGAATATTTGGCAAAGGCTGCTGGTGCGT
CTACCAACTTGTCCCTCCGCGCTTGCTAGAGCGACCAACTACGCTCTGTAACGTCCAGT
ACCCGAGCAGTCTCTCAACTTGGCCGATGAGACTGTATACACCGCCGTTGGGACGCGG
ACTAAACAACTCCCTCATACCCATCCGCCCGTCCGGAGCAGAACGACACGGCGGTACC
GGCGTCAGGCCCCCTCGCCTGAACTGCATCATTATGAGGGGAGGTGCGTCGACTGGGG
GCCGGCGACTTGATCCCGGTCGGCCCGTGAAGCCCATCAGTCCCATGGGGACGTTCTC
CCCCATCCATTGGCCTCTGGGGCTTTCCACCAATTGCACCAGAATAATGCCAGATGTTA
GATGTATTCATCGTGGGGCACGAGCCTTCCCCGCGTGAGTGCGGCTCGAAATCCGGTC
CCCCAGGCTGCCAGCTAACACGGAAGTGTGCCTCGTTTCGCTCAGAGGGGACGAGACA
CTCTCGATGGCTCGCAACAAGCCTAGTCTCCACGATACATACAATCGTGCCAGGTGGG
ATTAATACGCCGGAGGCCCATCGCGAGATGTTTGAGGGAGAGATACGTTCCGGCCATA
TACTCCCTGTCCGCATCCCTTGGGGAAATCCGTTACACTGCCGTTGACGTCGTTAACGC
CACGCCACTAACACCGAACTAGGAGGACCAAACTATACCATGTTATCCGTATGCCCAG
TTGTTGTTGCGGTTCAGGGTACGGGGCTGTGAGGCACGTACGCGTTCCTGGTGGGAACT
GGTCCTCTGAACTTGAATGACTGCTCACTGGAATGGGATTTAGCAAACCATCCCAACAT
ATTCCAGGCCTCTGATCAGATCACAAGCTTGCTTTCACCAGTTCCCAATTATGATTCCG
CATCGTCGGGAGGCCTTCGACTAGATCTACTCGCGTGGTTCGAGCGGGAATATCAAGT
CTGAAATCCCCTGACCTAACGGGAGGTGGCACGCGCGATCGGAATCTTCAGTTTGTGG
GAGCTAATCGTAATGACGATGGACCTTCTACAAGTGAGTGCTGTAGCGCCAGCAGCGG
ACCAGATTGTTTTCCGGATGTTCCCCCTCGTGCCGAAGGCTGACCTCAGGGCGGGGCCG
TGGGCGCGCACCGTTGCCACGCAGGTACACCGTGAGGCACCTTGAAGGTCCCGTAATG
AAC

>SCT_LTZ208.1

GTCGGGATGCCCTTTCGACACCAGCACGGCGCGACGCTTTCGCGCACGTATCAGATAA
GTTTTCCCGTGGACGGCTCGTCCTCAGAAATATCTCGTAGTCTTTTCGTTGGTTACTCCA
ATGAAGCGCCTCGCCTGCGGGAGCACTAAACCGCGAGCGCCATTCGAGTGCTCTACAA
CTCGACCGGGGGCAGCACACAACCGTAGGATCGGCGCGGCTCATTTTCGGCTAGTAGG
GGGTCCGACGTCCCGGCCCGTTTTCTCGCGTCGGAGGCTGCCAACACGATAGTAACTG
AACTGGCCCAAACGTATTGATACGCCCAGAGGGCGGGCCTCTGGCGCGCCACTGGATC
AGGCCCGTGGCGTGCCCGCCTCGTCAGCGCCACCCATTGCTAAGCGCTGACAGTAATA
GACCCCTCCATAGTAGTTGCCGATGTTGATTCGGTCACCGGCCGAAACGTATGCACTTA
GCACAGGGCAGGTACTACAAAGCGAGAGGGGGATGATTGGCAGGGGCTGCTGACGCG
CCTATCAGCCCGTTCCCCCGCGCCTGCTGTGGCGACCAACTACGCCCCGCAGCGTCCAG
TACCCGAGCAGTCTCTCAACTGGTCCGATTAGACTGTATACACCGCCGTTGGGACGCG
GACTAAACAACTCCCTCATACCCATCCGCCCGTCCGGAGCGGAACGACTCGGCGGTAC
CGGCGTCAGGCCCCCTCGCCTAGACGGCACCATTATGTTGGGAGGTGCGTCGACTGGG
GGCCGGCGACTTGATCCTGGTCGGCCCGTGAAGCCCATCAGTCCCATGGAGACGTTCT
CTCCCATCTATTGGCCTCCGGGGCTCTCCACCAATCGCACCGGAGTCTTGTCTAATATG
AAATTTAAACATCGTGAGGTATAAACCGCCCCCGAGTGGGTGCGGCTTGAAGTCCAGC
CCCCCAGGCCGCCTGCTAACACGGAAGTGTACCCGGTTCCACTCAGAGGGCATGAGGC
AGTACCTACGGATCTCAACAAGCCTAGTCCCCATGATACGTACAGCTGGGCCAGGTGG
GACTAATACGCCGGGGTTCCGTTGCGGAACGGCTAGAAGGTAAATACGCCTTGGCCAT
ATACTCTTTGTCCGCATCCCTTGGGGAAATCCATTATGCTGCCGTTGACGTCGTCAACG
TCACGCCACTAACACCGAACTAGGGGGACCAAACTATACCATGTTATCCGTATACTTA
CCCACTGTTGCAGGTCAAGGTATGGGGCTGCAAGTAATGTACATACTCCTGGTGGGAA
CTTGTCCTCCGGACTTGAATGGCTACCTGCCGGGCTGGGATTTAGCAAACCATCCCAAC
ATATTCCAGGCCTCTGATCAGATCACAATTTTGCTTTCACCAGTTCCCAATTATGATTCC

GCATCGTCGGGAGGCCTTCGACTAGATCTGCTCGCATGGTTCGAGCGGTAATATCGGG
TCTGAAATCCCCTGACCCAACGGGAGGCGGTATGCGTGATCGGAATCTGCACCTTGCG
AGAGCTAATCGTGATGACAATGGCTCTTCCACAAGTGAGTGCGGGGGCGCGAGCGGCG
AACCAGATGGTCCTCTGAATATTCCCCCCCGTGCCGAAGGCTGAACTCGAGGCGGGGC
CGTGGTCGCGCACCGTTACCACGCAGGTGCGTCGCGAGGCCCCTTGAAGGTCCCGTCA
TGAAG

>SCT_LTZ208.2

GTCGGGATGCCCTCTCGATACTGGCACGGAGCGGCGCCTTCGCGCACGGATCGGATAA
GCTTTCCCGTAGATGGCTCGTCCTCGGAAATATCTCGTAGTCTTCTCGTTGGTTACTCCA
ATGAAGCGCCTCGTCTGCGGGGGCACTAAACCGCGAGAGCTATTCAAGTGCTCTACTA
CTCGACCGGGGGCAGCTTACAACCGTGGGATCGGCGCGGCCCACCTCCGGTCAACGAG
AGTTACGAGTTCCCCGCCCGTTTTCCCGCGTCGGAGGTTGCCAACAGGATAGTAACTGA
ACTGGCCCAAACGTATTAATACGCCCCGGGGGCGGGCCTCTGGCGCGCCACTGGATCA
GGCCTGTGGCGTGCCCGCCTCGTCAGCGCCACCCATTGCTAAGCGCTGACAGTAATAG
ACCCCTCCATAGTAGTTGCCGATGTTGATTTGGTCACCGGCCGAAACGTATGCGCTCAG
CACAGGGCAGGTACTACGGAGCGAAAGGTGGATGATTGGCAGGGGCCGCTGGCGCAC
CTACAAAACTATTCGTCCGCGCCTGCTGGAGCGACCAACTACGCTCTATAGCGTCCAGT
ACCCGAGCAGTCCCTCAATTGGTCCGATGAGACTGTATACGCCGCCGTTGGGACGCGG
ACTACACAACCCCCTCATACCCATTCGCCCGTCCAGAGCGGAACGACTCCGCGGTGCC
GGCGTCAGGCCTCCTCGCCTAGGCTGCGCCATTATGTTGGGAGGTGCGTTGACTGGGG
GCCGGCGACCTGAGCTTGGTCGGCCCGTGAAGCCCATCACTCCCATGGAGACGTTTTCT
CCCACCCATTGGCCCCCGGGGTCCTCCACCAATTGCGTCGGAGTCTTGTCTAATATGAA
ATTTATTCATCGTAAGGTATAAACCGCCCCCGCGTGGGTGCGGCTCGAAGTCCGGCTTC
CCAAGCTGGCTGCTAACACGGAAGTGTACCCGGTTCCACTCAGAGGGCCTGAGGCAGT
ACCTACGGATCTCAAAATCCCTAGTCCCCATGATACGTACAGCTGGGCTAGGTGGGAT
TAATACGTCGGGGTCCCATCGCGGGATGTTTGAGGGGGAGATACGTTCCAGCTATATA
CTCCCTGATCGCGCCCCTCAGAGAAGTCCATTACGCTGCCGTTGACGTCGTCAATGCCG
CGCCACTAACACCGGACTAGGGGGACCAAACCATACCATGTAATACGTGTACTTGCCC
ATTGTCGCGGTTCAGAGTACGGGGCTGTGAGTAATGTACGCGTTCCTGGTGGGAACTT
GTCCTCTGAACTTGAATAACAGCTCACTGGGCTGGGACTTAGCAAACCACCCTGACGC
ATCCCAGGCCTTTGATCGGATCACAATTTTGCTTTCACCAGTTCCCAATTACGGTTCCG
CATCGTCGGGAGGCCTTCGACTAGATCTACTCGCATGGTTCGAGCAGTAATATCGGGTC
TGAAATCCCCTGACCCAACGGGGGGCAGTATGTGTGGTCGGAATCTGCACCTTGCGAG
AGATAATCGTAATGACGATGGACCTTCTACAAGTGAGTGCTGGGGCACGAGCGGCTGA
CCAGATTGTCTTCCGAATGTTCCCCCCCGTGCCGAAGGCTGAACCCGAGGCGGAGCCG
TGGGCGTGCACCGTTGCCACGCAGGTGCACCGCGAGGCACCTTGAAGGTCCCGTCATG
ACG

>SCT_LTZ2011.1

GTCGGGATGCCCTTTCGACACCAGCACGGCGCGACGCTTTCGCGCACGTATCAGATAA
GTTTTCCCGTGGACGGCTCGTCCTCAGAAATATCTCGTAGTCCTTTCGTTGGTTACTCCA
ATGAAGCGCCTCGCCTGCGGGAGCACTAAACCGCGAGCGCCATTCGAGTGCTCTACAA
CTCGACCGGGGGCAGCACACAACCGTAGGATCGGCGCGGCTCATTTTCGGCTAGTAGG
GGGTACGACGTCCCGGCCCGTTTTCTCGCGTCGGAGGCTGCCAACACGATAGTAACTG
AACTGGCCCAAACGTATTGATACCCCCAGGGGGCGGGCCTCTGGCGCGCCACTGGATC
AGGCCCGTGGCGTGCCCGCCTCGTCAGCGCCACCCATTGCTAAGCGCTGACAGTAATA
GACCCCTCCATAGTAGTTGCCGATGTTGATTCGGTCACCGGCCGAAACGTATGCACTTA
GCACAGGGCAGGTACTACAAAGCGAGAGGGGGATGATTGGCAGGGGCTGCTGACGCG
CCTATCAGCCCGTTCCCCCGCGCCTGCTGTGGCGACCAACTACGCCCCGCAGCGTCCAG

TACCCGAGCAGTCTCTCAACTGGTCCGATTAGACTGTATACACCGCCGTTGGGACGCG
GACTAAACAACTCCCTCATACCCATCCGCCCGTCCGGAGCGGAACGACTCGGCGGTAC
CGGCGTCAGGCCCCCTCGCCTAGACTGCACCATTATGTTGGGAGGTGCGTCGACTGGG
GGCCGGCGACTTGATCCTGGTCGGCCCGTGAAGCCCATCAGTCCCATGGAGACGTTCT
CTCCCATCTATTGGCCTCCGGGGCTCTCCACCAATCGCACCGGAGTCTTGTCTAATATG
AAATTTATTCATCGTGAGGTATAAACCGCCCCCGAGTGGGTGCGGCTTGAAGTCCAGC
CCCCCAGGCCGCCTGCTAACACGGAAGTGTACCCGGTTCCACTCAGAGGGCATGAGGC
AGTACCTACGGATCTCAACAAGCCTAGTCCCCATGATACGTACAGCTGGGCCAGGTGG
GATTAATACGCCGGGGTTCCGTTGCGGAACGGCTAGAAGGTAAATACGCCTTGGCCAT
ATACTCTTTGTCCGCATCCCTTGGGGAAATCCATTATGCTGCCGTTGACGTCGTCAACG
TCACGCCACTAACACCGAACTAGGGGGACCAAACTATACCATGTTATCCGTATACTTA
CCCACTGTTGCAGGTCAAGGTTTGGGGCTGCAAGTAATGTACATACTCCTGGTGGGAA
CTTGTCCTCCGGACTTGAATGGCTACCTGCCGGGCTGGGACTTAGCAAACCACCCTGAC
GCATCCCAGGCCTTTGATCGGATCACAATTTTGCTTTCACCAGTACCCAATTACGGTTC
CGCATCGTCGGGAGGCCTTCGACTAGATCTACTCGCATGGTTCGAGCAGTAATATCGG
GTCTGAAATCCCCTGACCCAACGGGGGGCAGTATGTGTGGTCGGAATCTGCACCTTGC
GAGAGATAATCGTAATGACGATGGACCTTCTACAAGTGAGTGCTGGGGCACGAGCGGC
TGACCAGATTGTCTTCCGAATGTTCCCCCCCGTGCCGAAGGCTGAACCCGAGGCGGAG
CCGTGGGCGTGCACCGTTGCCACGCAGGTGCACCGCGAGGCACCTTGAAGGTCCCGTC
ATGAAG

>SCT_LTZ2011.2

GTCGGGATGCCTTCCCAACTCCGGCACGGCGCGGCGCCTTCGCGCACGGATCAGATAA
GTTTTCTCGTGGACCGCTCAACCCCGGAAATTCCCCGAGGTTTTCCCAGTGGTTACCCT
GATGCCGCGCCGCGTCTGCTTGGATGCTGAACCGCGAGAGCTATTCAGGTGCTCGACT
ACTCGACCGGGGGCAGCACGCAGCCGCGGGAACGACGCGGTCTACCTCCGGCCAGCA
GGGGGTACGACGTCCCGGCCCGTTTTCCCGCGTCGGAGGCTGCCAACACGATAGTAAC
TGAAATGGTCCAAACGTATTAATACGCACAGGGGGCGGGCCTCTGGCGCGCCACTGGA
TCAGGCCCGTGGCGTGCCCGCCTCGTCAGCGCCACCCATTGCTAAGCGCTGACAGTAA
TAGACCCCTCCATAGTAGTTGCCGATGTTGATTCGGTCACCGGCCGAAACGTATACACT
CGGTACGGGGTGGGTACCACGGAGCGAAAGGTGGATGATTGGCAGGAGCCGCTGGCG
CACCTACAAAACTATTCGTTCGCGCCTGCTGGAATGACCAACTACGCTCTATAGCGTCC
AGTACTCGAGCAGTCCCTCAATTGGTCCGATGAGACTGTATACGCCGCCGTTGGGACG
CGGCCTAAATAACCCCTTTACGCACATCCGCCCATCCGGAGCGGAACGACTCGGCGGT
ACCGGCGTCAGGCCCCCTCGCCTAGACTGCACCATTATGTTGGGAGGTGCGTCGACTG
GGGGCCGGTGACTTGATCCTGGTCGGCCCGTGAGGCCCATCAGTCCTATGGAGACGTT
CTCTCCCATCCATTGGCCTCCTAGGCTTTCCACCAAGTGCGTCAGGGTAATGCCAGATG
TTAGATGTATTCATCGTGAGGTATACACCGCCCCCGCGTGGGTGCGGCTCGAAGCCCG
GCCTCCCAGGCTGGCTGCTAACACGGAAGTGTAGCCGGTTCCACTCAGAGGGCATGAG
GCAGTACCTACGGATATCAACAAGCCTAGTCCCCATGATACGTACAGCTGGGCCAGGT
GGGATCAATACGTCGGAAGCCCATCGCGGGATGTTTGAGGGGGAGATCCGTTCCGGCC
ATATACTCCCTGTCCGCATCCCTTGGGGTAATCCATTACGCCGTCGTTGACGTCATCAA
TGCCGCGCGATTAACACCGAGCTGATGGGACCAAAACATACCATGTAATCCGTGTACT
TACCCATCGTTGCGGTACAGGATACGGGGCTGTGAGTAATGTACACACTCCTGGAGGG
AGCTTGTCCTCCAGACTTGAATGGCTACCTGCCGGGCTGGGACTTAGCAAACCACCCTG
ACGCATCCCAGGCCTTTGATCGGATCACAATTTTGCTTTCACCAGTTCCCAATTACGGT
TCCGCATCGTCGGGAGGCCTTCGACTAGATCTACTCGCATGGTTCGAGCAGTAATATCG
GGTCTGAAATCCCCTGACCCAACGGGGGGCAGTATGTGTGGTCGGAATCTTCAGCTTG
TGGGAGCTAATCGTAATGACGATGGACCTTCTACAAGTGAGTGCTGTAGTGCCAGCAG
CGGACCAGATTGTTTTCCGGATGTTCCCCCTCGTGCCGAAGGCTGAACTCAGGGCGGG

GCCGTGGGCGCGCACCGTTGCCACGCAGGTACACCGTGAGGCACCTTGAAGGTCCCGT
AATGAAC

>SCT_LTZ2016.1

GTCGGGATGCCCTCTCGATACTGGCACGGAGCGGCGCCTTCGCGCACGGATCGGATAA
GCTTTCCCGTAGATGGCTCGTCCTCGGAAATATCTCGTAGTCTTCTCGTTGGTTACTCCA
ATGAAGCGCCTCGTCTGCGGGGGCACTAAACCGCGAGAGCTATTCAAGTGCTCTACTA
CTCGACCGGGGGCAGCTTACAACCGTGGGATCGGCGCGGCCCACCTCCGGTCAACGAG
AGTTACGAGTTCCCCGCCCGTTTTCCCGCGTCGGAGGTTGCCAACAGGATAGTAACTGA
ACTGGCCCAAACGTATTAATACGCCCCGGGGGCGGGCCTCTGGCGCGCCACTGGATCA
GGCCTGTGGCGTGCCCGCCTCGTCAGCGCCACCCATTGCTAAGCGCTGACAGTAATAG
ACCCCTCCATAGTAGTTGCCGATGTTGATTTGGTCACCGGCCGAAACGTATGCGCTCAG
CACAGGGCAGGTACTACGGAGCGAAAGGTGGATGATTGGCAGGGGCCGCTGGCGCAC
CTACAAAACTATTCGTCCGCGCCTGCTGGAGCGACCAACTACGCTTTATAGCATCCAGT
ACCCGAGCAAGCCCTCAATTGGTCCGATGAGACGGTATACGCCGCCGTTGGGACGCGG
ACTAATCAACCCCCTCATACCCATTCGCCCGTCCAGAGCGGAACGACTCCGCGGTGCC
GGCGTCAGGCCTCCTCGCCTAGGCTGCGCCATTATGTTGGGAGGTGCGTTGACTGGGG
GCCGGCGACCTGAGCTTGGTCGGCCCGTGAAGCCCATCACTCCCATGGAGACGTTTTCT
CCCACCCATTGGCCCCCGGGGTCCTCCACCAATTGCGTCGGAGTCTTGTCTAATATGAA
ATTTAATCATCGTAAGGTATAAACCGCCCCCGCGTGGGTGCGGCTCGAAGTCCGGCTTC
CCAAGCTGGCTGCTAACACGGAAGTGTACCCGGTTCCACTCAGAGGGCCTGAGGCAGT
ACCTACGGATCTCAAAAACCCTAGTCCCCATGATACGTACAGCTGGGCTAGGTGGGAT
TAATACGTCGGGGTCCCATCGCGGGATGTTTGAGGGGGAGATACGTTCCAGCTATATA
CTCCCTGTTCGCGCCCCTCAGAGAAGTCCATTACGCTGCCGTTGACGTCGTCAATGCCG
CGCCACTAACACCGGACTAGGGGGACCAAACCATACCATGTAATACGTGTACTTGCCC
ATTGTCGCGGTTCAGAGTACGGGGCTGTGAGTAATGTACGCGTTCCTGGTGGGAACTT
GTCCTCTGAACTTGAATAACAGCTCACTGGAATGGGATTTAGCAAACCATCCCAACAT
ATTCCAGGCCTCTGATCAGATCACAATTTTGCTTTCACCAGTTCCCAATTATGATTCCGC
ATCGTCGGGAGGCCTTCGACTAGATCTGCTCGCATGGTTCGAGCGGTAATATCGGGTCT
GAAATCCCCTGACCCAACGGGAGGCGGTATGCGTGATCGGAATCTGCACCTTGCGAGA
GCTAATCGTGATGACAATGGCTCTTCCACAAGTGAGTGCGGGGGCGCGAGCGGCGAAC
CAGCTGGTCCTCTGAATATTCCCCCCCGTGCCGAAGGCTGAACTCGAGGCGGGGCCGT
GGTCGCGCACCGTTACCACGCAGGTGCGTCGCGAGGCCCCTTGAAGGTCCCGTCATGA
AG

>SCT_LTZ2016.2

GTCGGGACGCCCTCCCGGCTCTGGCACGGCGCGGTGGCTTTGCGCACGGATCGGATAA
ACTTTCCATTGGACGGCTCGTTCTCAGAAATATCTCGTAGTCTTCTCGTTGGTTACTCCA
ATGAAACGCCTCGTCTGCGGGGGCACTGAACCGCGAGAGCTATTCAAATGCTCTACTA
CTCGACCGGGGGCAGCTTACAACCGTGGGATCGGCGCGGCCCACCTCCGGTCAACGAG
AGTTACGAGTTCCCGGCCCGTTTTCTCGCGTCGGAGGCTGCCAACACGATAGTAACTGA
ACTGGCCCAAACGTATTAATACGCCCCGGGGGCGGGCCTCTGGCGCGCCACTGGATCA
GGCCCGTGGCGTGCCCGCCTCGTCAGCGCCACCCATTGCTAAGCGCTGACAGTAATAG
ACCCCTCCATAGTAGTTGCCGAAGTTGATTCGGTTGCCGGCCGAAACGTATGCACTTAG
CACAGGGCAGGTACTACAAAGCGAGAGGTGGATGATTTGCAAGGGTTGTTGGCACGCC
TACAAAACTACTTCTCCGCTCCTGCTGGAGCGACCAACTACGCCCCGCAGCAACCGGT
ACTTGAGCTGTCTCTCAACTGGTCCGATTAGACTGTATACACCGCCGTTGGGACGCGGA
CTAAACAATTCCCTCATACCCATCCGCCCGTCCGGAGCGGAACGACTCGACGGTACCG
GCGTCAGGCCCCCTCGCCTAGGCTGCACCATTATGTTGGAAGGTACATCGACTGGGGG
CCGGCAACTTGAGCTTGGTCGGCCCGTGAAACCCACCAGTCCCATGGAGAAGGTCTCT

CCCATCCATTGGCCTCCGGGGCTCTTCATCAATCGCGTCGGAGTATCGTCTAATGTGAA
ATTTATTCATCGTGAGGTATACACCGCCCCCGCGTGGGTGCGGCTCGAAGCCCGGCTTC
CCAGGCTGGCAGCTAACACGGAAGTGTGCCTCGTTTCGCTCAGAAGGGACGGGACACT
CTCGACGGCCCTCAACAAGCCTAGTCCCCACGATACATACAACCGTGCCAGGTGGAAT
TAATACGTCGGGGTCCCATCGCGGGATGTTTGAGGGGGAGATACGTTCCGGCCATATA
CTCCCTGTTCGCGCCCCGCAGAGAAGTCCATTACGCTGCCGTTGACGTCGTCAATGCCG
CGCCACTAACACCGGACTAGGGGGACCAAACCATACCATGTAATCCGTGTACTTGCCC
ATTGTTACGGTTTAGGGAACGGGGCTGTGGCTAATAGACGCGCTCCTGGTGGGAGCTT
GTCCTCCGGACTCGAATGGCTACTCGCCGGGCTGGGACTTAGCAAACCACTCTGACGC
ATCTACGGCCTCAGATTAGATCACAAGCTTGCTTTCTCCATTTCCTAATTACCGTTCCGC
ATCGTCGGGAGGTCTTCGACTAGATTTACTCGCATGGTTTGAGTGATAATATCAAGTCT
GGAGTCCTCTGACCCAACGTGAAGCGGTATGCGCAATTGGGGTCTGTTCCTTGCAAGA
GCTAATCGTAATGACGATGGACCTTCTACAAGTGAGTGCTGTAGCGCCAGCAGCGGAC
CAGATTGTTTTCCGGATGTTCCCCCTCGTGCCGAAGGCTGAACTCAGGGCGGGGCCGTG
GGCGCGCATCGTTGCCACGCAGGTACACCGTGAGGCACCTTGAAGGTCCCGTCATGAA
C

>GST_R06.1

TTCGGGATGCCCTTTCGACTGTGCCGCGGCGCGGCGCCTCCGCGCACGGATCAGATAA
GCTTTCCCGTGGACGGCTCGTCCTCAGAAACATTTCGTAGTCTTCTCGTTGGTTACTCCA
CTGCCGCGCCGCGTCTGCTTGGATGCTGAACCACGAGATTTATTAGAGTACTCTACTAC
TCGACCGGGGGCAGCATACAACCGTGGGATCGGTGCGACCCTCCTCCGGCCCGCAGGG
AGTACGACGTCCCGGCCTGTTTTCCTGCGTCAGAGGCTGCCAACACGATAGTAACTGA
AATGGCCAAAACGTACTAATACGCCCCGGTTGCGGGCCTCTGGCGCGCCACTAAACTG
AGTCCAGGATGTGCCCGCCTCGCCATCGTCATTCATCGCTGTATATTAGAAAAATCAGA
TTCTCTCATAGTAGTTGCCGATGTTGATTCGGTCACCGGCCGAAACGTATGCACTTAGC
ACAGGGCAGGTACTACAAAGCGAGAGGGGGATGATTGGCAGGGGCTGCTGACGCGCC
TATCAGCCCGTTCCCCCGCGCCTGCTGTGGCGACCAACTACGCCCCGCAGCGTCCAGTA
CCCGAGCAGTCTCTCAACTGGTCCGATGAGACTGTATACACCGCCGTTGGGACGCGGA
CTAAACAACTCCCTCATACCCATCCGCCCGTCCGGAGCGGAACGACTCGGCGGTACCG
GCGTCAGGCCCCCTCGCCTAGACTGCACCATTATGTTGGGAGGTGCGTCGACTGGGGG
CCGGCGACTTGATCCTGGTCGGCCCGTGAAGCCCATCAGTCCCATGGAGACGTTCTCTC
CCATCTATTGGCCTCCGGGGCTCTCCACCAATCGCACCGGAGTCTTGTCTAATATGAAA
TTTATTCATCGTAAGGTATAAACCGCCCCCGAGTGGGTGCGGCTTGAAGTCCAGCCCCC
CAGGCCGCCTGCTAACACGGAAGTGTACCCGGTTCCACTCAGAGGGCATGAGGCAGTA
CCTACGGATCTCAACAAGCCTAGTCCCCATGATACGTACAGCTGGGCCAGGTGGGATT
AATACGCCGGGGTTCCGTTGCGGAACGGCTAGAAGGTAAATACGCCTTGGCCATATAC
TCTTTGTCCGCATCCCTTGGGGAAATCCATTATGCTGCCGTTGACGTCGTCAACGTCAC
GCCACTAACACCGAACTAGGGGGACCAAACTATACCATGTTATCCGTATACTTACCCA
CTGTTGCAGGTCAAGGTATGGGGCTGCAAGTAATGTACATACTCCTGGTGGGAACTTG
TCCTCCGGACTTGAATGGCTACCTGCCGGGCTGGGACTTAGCAAACCACCCTGACGCA
TCCCAGGCCTTTGATCGGATCACAATTTTGCTTTCACCAGTTCCCAATTACGGTTCCGC
ATCGTCGGGAGGCCTTCGACTAGATCTACTCGCATGGTTCGAGCAGTAATATCGGGTCT
GAAATCCCCTGACCCAACGGAGGGCAGTATGTGTGGTCGGAATCTGCACCTTGCGAGA
GATAATCGTAATGACGATGGACCTTCTACAAGTGAGTGCTGGGGCACGAGCGGCTGAC
CAGATTGTCTTCCGAATGTTCCCCCCCGTGCCGAAGGCTGAACCCGAGGCGGAGCCGT
GGGCGTGCACCGTTGCCACGCAGGTGCACCGCGAGGCACCTTGAAGGTCCCGTCATGA
AG

>GST_R06.2

GTCGGGATGCCTTCCCGACTCCGGCACGGCGCGGCGCCTTCGCGCACGGATCGGATAA
GCTTTCCCGTAGATGGCTCGTTCTCAGAAATATCTCGTAGTCTTCTCGTTGGTTACTCCA
ATGAAGCGCCTCGTCTGCGGGGGCACTGAACCGCGAGCGCTATTCAAGTGCTCTACTA
TTCCACCGGGGGCAGCACGCAGCCGTGGGATCGGTGCGACCCTCTTTCGGCTAGTAGG
GGGTACGACGTCCCGGCCCGTTTTCCCGCGTCGGAGGCTGCCAACACGGCAGTAACTG
AACTGGCCCAAACGTATTAATACGCACAGGGGGCGGGCCTCTGGCGCGCCACTGGATC
AGGCCCGTGGCGTGCCCGCCTCGTCAGCGCCACCCATTGCTAAGCGCTGACAGTAATA
GACCCCTCCAGAGTAGTTGCCGATGTTGATTTGGTCACCGGCCGAAACGTATGCGCTCA
GCACAGGGCAGGTACTACGGAGCGAAAGGTGGATGATTTGCAGGGGCCGCTGGCGCA
CCTACAAAACTATTCGTCCGCGCCTGCTGGAGCGACCAACTACGCTCTATAGCGTCCAG
TACCCGAGCAGTCCCTCAACTGGTCCGATTAGACTGTATACACCGCCGTTGGGACGCG
GACTAAACAACTCCCTCATACCCATCCGCCCGTCCGGAGCGGAACGACTCGGCGGTAC
CGGCGTCAGGCCCCCTCGCCTAGACTGCACCATTATGTTGGGAGGTGCGTCGACTGGG
GGCCGGCGACTTGAGGTCGGCCGTTTCGTGAAGCCCATTAGTCCCATGGAGACGTTCTC
TCCCATCCATTGGCCTCCGGGGCTCTTCATCAATCGCGTCGGAGTATCGTCTAATGTGA
AATTTATTCATCGTGAGGTATACACCGCCCCCGCGTGGGTGCGGCTCGAAGTCCGGCCC
CCCAGGCTGCCTGCTAACACGGCAGTATGCCTCGTTTAGCGCAGAAGGGACGAGACAC
TCTCGACGGCTCTCAACAAGCCTAGTCCCCACGGTACATACTACCGTGCCAGGTGGGA
TTGGTACGCCGGGGTCCCGTCACAGAACGGCTAGAAGGTAAATACGCCCTGGCCATAT
ACTCCTTGACCGCATCCCTTGGGGAAATCCATTACGCCGTCGTTGACGTCATCAATGCC
GCGCGATTAACACCGAGTTAGTGGGACCAAAACATACCATGTAATCCGTGTACTTACC
CATCGTTGCGGTACAGGATACGGGGCTGTGAGTAATGTACACACTCCTGGAGGGAGCT
TGTCCTCCAGACTTGAATGGCTACCTGCCGGGCTGGGACTTAGCAAACCACCCTGACG
CATCCCAGGCCTTTGATCGGATCACAATTTTGCTTTCACCAGTTCCCAATTACGGTTCC
GCATCGTCGGGAGGCCTTCGACTAGATCTACTCGCATGGTTCGAGCAGTAATATCGGG
TCTGAAATCCCCTGACCCAACGGGGGGCAGTATGTGTGGTCGGAATCTGCACCTTGCG
AGAGATAATCGTAATGACGATGGACCTTCTACAAGTGAGTGCTGGGGCACGAGCGGCG
GACCAGATTGTCTTTCGAATGTTCCCCCCCGTGCCGAAGGCTGAACCCGAGGCGGAGC
CGTGGGCGTGCACCGTTGCCACGCAGGTGCACCGCGAGGCACCTTGAATGTCCCGTCA
TGAAG

>GST_R11.1

TTCAAGATGCCCTCCTGACTCTGCCACGACGCGGTGGCTTCGCGCATGGACCGGATAA
GTTTTCCCGTGGACCGCTCAACCCCGGAAATACCTCGAGGTTTTCTCAGTGGTTACTCT
GATGCCGCGCCGCGTCTGCTTGGATGCTGAACCGCGAGAGCTATTCAGGTGCTCGACT
ACTCGACCGGGGGCAGCACGCAGCCGCGGGAACGACGCGGTCTACCTCCGGCCAGCA
GGGGGTACGACGTCCCGGCCCGTTTTCCCGCGTCGGAGGCTGCCAACACGATAGTAAC
TGAAATGGTCCAAACGTATTAATACGCACAGGGGGTGGGCCTCTGGCGCGCCACTGGA
TCAGGCCCGTGGCGTGCCCGCCTCGTCAGCGCCACCCATTGCTAAGCGCTGACAGTAA
TAGACCCCTCCATAGTAGTTGCCGATGTTGATTTGGTCACCGGCCGAAACGTATGCGCT
CAGCACAGGGCAGGTACTACGGAGCGAAAGGTGGATGATTGGCAGGGGCCGCTGGCG
CACCTACAAAACTATTCGTCCGCGCCTGCTGGAGCGACCAACTACGCCCTATAGCGTCC
AGTACCCGAGCAGTCCCTCAACTGGTCCGATTAGACTGTATACACCGCCGTTGGGACG
CGGACTAAACAACTCCCTCATACCCATCCGCCCGTCCGGAGCGGAACGACTCGGCGGT
ACCGGCGTCAGGCCCCCTCGCCTAGACTGCACCATTATGTTGGGAGGTGCGTCGACTG
GGGGCCGGCGACTTGAGGTCGGCCGTTTCGTGAAGCCCATTAGTCCCATGGAGACGTT
CTCTCCCATCCATTGGCCTCCGGGGCTCTTCATCAATCGCGTCGGAGTATCGTCTAATG
TGAAATTTATTCATCGTGAGGTATACACCGCCCCCGCGTGGGTGCGGCTCGAAGTCCG
GCCCCCCAGGCTGCCTGCTAACACGGAAGTATGCCTCGTTTAGCTCAGAAGGGACGAG
ACACTCTCGACGGCTCTCAACAAGCCTAGTCCCCACGGTACATACTACCGTGCCAGGT
GGGATTGGTACGCCGGGGTCCCGTCACAGAACGGCTAGAAGGTAAATACGCCCTGGCC

ATATACTCCTTGTCCGCATCCCTTGGGGAAATCCATTACGCCGTCGTTGACGTCATCAA
TGCCGCGCGATTAACACCGAGCTGATGGACCAAAACATACCATGTAATCCGTGTACT
TACCCATCGTTGCGGTACAGGATACGGGGCTGTGAGTAATGTACACACTCCTGGAGGG
AGCTTGTCCTCCAGACTTGAATGGCTACCTGCCGGGCTGGGACCTAGCAAACCACCCT
GACGCATCCCAGGCCTTTGATCGGATCACAATTTTGCTTTCACCAGTTCCCAATTACGG
TTCCGCATCGTCGGGAGGCCTTCGACTAGATCTACTCGCATGGTTCGAGCAGTAATATC
GGGTCTGAAATCCCCTGACCCAACGGGGGGCAGTATGTGTGGTCGGAATCTGCACCTT
GCGAGAGATAATCGTAATGACGATGGACCTTCTACAAGTGAGTGCTGGGGCACGAGCG
GCGGACCAGATTGTCTTTCGAATGTTCCCCCCCGTGCCGAAGGCTGAACCCGAGGCGG
AGCCGTGGGCGTGCACCGTTGCCACGCAGGTGCACCGCGAGGCACCTTGAATGTCCCG
TCATGAAG

>GST_R11.2

TTCGGGATGCCCTTTCGACTGTGCCGCGGCGCGGCGCCTCCGCGCACGGATCAGATAA
GCTTTCCCGTGGACGGCTCGTCCTCAGAAACATTTCGTAGTCTTCTCGTTGGTTACTCCA
CTGCCGCGCCGCGTCTGCTTGGATGCTGAACCACGAGATTTATTAAAGTACTCTACTAC
TCGACCGGGGGCAGCATACAACCGTGGGATCGGTGCGACCCTCCTCCGGCCCGCAGGG
AGTACGACGTCCCGGCCTGTTTTCCTGCGTCAGAGGCTGCCAACACGATAGTAACTGA
ACTGGCCAAAACGTACTAATACGCCCCGGTTGCGGGCCTCTGGCGCGCCACTAAACTG
AGTCCAGGATGTGCCCGCCTCGCCATCGTCATTCATCGCTGTATATTAGAAAAATCAGA
TTCTCTCATAGTAGTTGCCGGTGTTGATTCGGTCACCGGCCGAAACGTATGCACTTAGC
ACAGGGCAGGTACTACAAAGCGAGAGGGGGATGATTGGCAGGGGCTGCTGACGCGCC
TATCAGCCCGTTCCCCCGCGCCTGCTGTGGCGACCAACTACGCCCCGCAGCGTCCAGTA
CCCGAGCAGTCTCTCAACTGGTCCGATTAGACTGTATACACCGCCGTTGGGACGCGGA
CTAAACAACTCCCTCATACCCATCCGCCCGTCCGGAGCGGAACGACTCGGCGGTACCG
GCGTCAGGCCCCCTCGCCTAGACTGCACCATTATGTTGGGAGGTGCGTCGACTGGGGG
CCGGCGACTTGATCCTGGTCGGCCCGTGAAGCCCATCAGTCCCATGGAGACGTTCTCTC
CCATCTATTGGCCTCCGGGGCTCTCCACCAATCGCACCGGAGTCTTGTCTAATATGAAA
TTTATTCATCGTGAGGTATAAACCGCCCCCGAGTGGGTGCGGCTTGAAGTCCAGCCCCC
CAGGCCGCCTGCTAACACGGAAGTGTACCCGGTTCCACTCAGAGGGCATGAGGCAGTA
CCTACGGATCTCAACAAGCCTAGTCCCCATGATACGTACAGCTGGGCCAGGTGGGATT
AATACGCCGGGGTTCCGTTGCGGAACGGCTAGAAGGTAAATACGCCTTGGCCATATAC
TCTTTGTCCGCATCCCTTGGGGAAATCCATTATGCTGCCGTTGACGTCGTCAACGTCAC
GCCACTAACACCGAACTAGGGGGACCAAACTATACCATGTTATCCGTATACTTACCCA
CTGTTGCAGGTCAAGGTATGGGGCTGCAAGTAATGTACATACTCCTGGTGGGAACTTG
TCCTCCGGACTTGAATGGCTACCTGCCGGGCTGGGACTTAGCAAACCACCCTGACGCA
TCCCAGGCCTTTGATCGGATCACAATTTTGCTTTCACCAGTTCCCAATTACGGTTCCGC
ATCGTCGGGAGGCCTTCGACTAGATCTACTCGCATGGTTCGAGCAGTAATATCGGGTCT
GAAATCCCCTGACCCAACGGAGGGCAGTATGTGTGGTCGGAATCTGCACCTTGCGAGA
GATAATCGTAATGACGATGGACCTTCTACAAGTGAGTGCTGGGGCACGAGCGGCTGAC
CAGATTGTCTTCCGAATGTTCCCCCCCGTGCCGAAGGCTGAACCCGAGGCGGAGCCGT
GGGCGTGCACCGTTGCCACGCAGGTGCACCGCGAGGCACCTTGAAGGTCCCGTCATGA
AG

>GST_R02.1

GTCGGGATGCCCTTTCGACACCAGCACGGCGCGACGCTTTCGCGCACGTATCAGATAA
GTTTTCCCGTGGACGGCTCGTCCTCAGAAATATCTCGTAGTCCTTTCGTTGGTTACTCCA
ATGAAGCGCCTCGCCTGCGGGAGCACTAAACCGCGAGCGCCATTCGAGTGCTCTACAA
CTCGACCGGGGGCAGCACACAACCGTAGGATCGGCGCGGCTCATTTTCGGCTAGTAGG
GGGTACGACGTCCCGGCCCGTTTTCTCGCGTCGGAGGCTGCCAACACGATAGTAACTG

AACTGGCCCAAACGTATTGATACGCCCAGGGGGCGGCCTCTGGCGCGCCACTGGATC
AGGCCCGTGGCGTGCCCGCCTCGTCAGCGCCACCCATTGCTAAGCGCTGACAGTAATA
GACCCCTCCATCGTAGTTGCCGATGTTGATTTGGTCACCGGCCGAAACGTATGCGCTCA
GCACAGGGCAGGTACTACGGAGCGAAAGGTGGATGATTGGCAGGGGCCGCTGGCGCA
CCTACAAAACTATTCGTCCGCGCCTGCTGGAGCGACCAACTACGCTCTATAGCGTCCAG
TACCCGAGCAGTCCCTCAATTGGTCCGATGAGACTGTATACGCCGCCGTTGGGACGCG
GACTAAACAACCCCCTCATACCCATTCGCCCGTCCGGAGCGGAACGACTCCGCGGTGC
CGGCGTCAGGCCTCCTCGCCTAGGCTGCGCCATTATGTTGGGAGGTGCGTTGACTGGG
GGCCGGCGACCTGAGCTTGGTCGGCCCGTGAAGCCCATCACTCCCATGGAGACGTTTT
CTCCCACCCAGTGGCCCCCGGGGTCCTCCACCAATTGCGTCGGAGTCTTGTCTAATATG
AAATTTATTCATCGTAAGGTATAAACCGCCCCCGCGTGGGTGCGGCTCGAAGTCCGGC
TTCCCAAGCTGGCTGCTAACACGGAAGTGTACCCGGTTCCACGCAGAGGGCCTGAGGC
AGTACCTACGGATCTCAAAATCCCTAGTCCCCATGATACGTACAGCTGGGCTAGGTGG
GATTAATACGTCGGGGTTCCGTTGCGGAACGGCTAGAAGGTAAATACGCCTTGGCCAT
ATACTCTTTGTCCGCATCCCTTGGGGAAATCCATTATGCTGCCGTTGACGTCGTCAACG
TCACGCCACTAACACCGAACTAGGGGGACCAAACTATACCATGTTATCCGTATACTTA
CCCACTGTTGCAGGTCAAGGTATGGGGCTGCAAGTAATGTACATACTCCTGGTGGGAA
CTTGTCCTCCGGACTTGAATGGCTACCTGCCGGGCTGGGACTTAGCAAACCACCCTGAC
GCATCCCAGGCCTTTGATCGGATCACAATTTTGCTTTCACCAGTTCCCAATTACGGTTC
CGCATCGTCGGGAGGCCTTCGACTAGATCTACTCGCATGGTTCGAGCAGTAATATCGG
GTCTGAAATCCCCTGACCCAACGGGGGGCAGTATGTGTGGTCGGAATCTGCACCTTGC
GAAAGATAATCGTAATGACGATGGACCTTCTACAAGTGAGTGCTGGGGCACGAGCGGC
TGACCAGATTGTCTTCCGAATGTTCCCCCCCGTGCCGAAGGCTGACCCCGAGGCGGAG
CCGTGGGCGTGCACCGTTGCCACGCAGGTGCACCGCGAGGCACCTTGAAGGTTCCGTC
ATGAAG

>GST_R02.2

GTCGGGATGCCTTCCCAACTCTGGCACGGCGCGGCGCCTTCGCGCACGGATCGGATAA
GCTTTCCCGTGGACGGCTCGTCCTCGGAAATATCTCGTAGTCTTCTCGTTGGTTGCTCCA
ACGAAGCGACGAATCTACTTGGATACTGAACCGCGAGAGCTATTCAAGTGCTCTACTA
CTCGACCGGGGGCAGCATACAGCCGTGGGATCGGCGCGGCCCACCTCCGGCCAACGAG
AGTTACGAGTACCCGGCCCGTTTTCCCGCGTCGGAGGCTGCCAACACGATCGTAACTG
AACTGGCCCAAACGTATTGATACGCCCCGGGGGCGGCCTCTGGCGCGCCACTGGATC
AGGCCCGTGGCGTGCCCGCCTCGTCAGCGCCACCCGTTGCTAAGCGCTGACAGTAATA
GACCCCTCCATAGTAGTTGCCGATGTAGGTCTGGTCACCGGCCGAAACGTATGAACGC
AGCACAGGGCAGGTACTACGGAGCGAAAGGTAGCTGATTGGCAGGGGCTGCTGGCGC
GCCTACCAACCTGTTCCTCCGCGCCTGCTGGAGCGACCAACTACGCCCCGCAGCGACC
GGTACCCGAGCAGTCTCTCAACTGGTTCGATGAGACTGTATACACCGTCGTTGGGACG
CGGACTAAACCGCCCCCTCATACCCACCCGCCCGTCCGGAGCGGAACGACTCGGCGGT
ACCGGCGTCAGGCCCCCTCGCCTAGGCTGCACCATTATGTTGGGAGGTGCGTCGACTG
GGGGCCGGCGACTTGAGCTCGGTCGTCCCGTGAAGCCCATCAGTCCCAGGGAGACGTT
CTCTCCCATCCATTGGCCTCCGGGGCTCTCCACCAATCGCACCGGAGTCTTGTCTAGTG
TTAAATGTATTCATCGTGGGGTATAAACCGCCCCCGCGTAAGTGCGGCTCGAAGTCTG
GCCTCCCAGGCTGCTAGCAAACACGGAAGTGTGCCTCGTTTCGCTCAGAGGGGACAAG
ACACTCTCGACGGCTCTCAACAAGCCTAGTCCCCACGATACATACAACCGTGCCCGGT
GGGATTAATACGCCGGGGTCCCGTCGCGGGACATCTGGGATGGGAACACGTTCCGACC
ATATGCTCTCTGTCTGCATCTCTTGGGGAAATCCATTACGCTGCCGTTGACGTCGTTAA
CGTCACGCCACTAATACCGAACTAGGGGGGCCAAGCTATACCATGTAATCCGTGCGCC
TACCCACTGTTGCGATTCAGGGTACGGGGCTGTGAGTAATGTACACACTCCTGGTGTGA
GCTTGTCCTCCGGACTTGAATGGCTACTCGCCGGGCTGGGACTTAGCAAACCACCCTGA
CGCATTCCAGGCCTCTGATCAGATCACAATTTTGCTTTCACCAGTTCCCAATTACGGTT

CCGCATCGTCGGGAGGCCTTCGACTAGATCTACTCGCATGGTTCGAGCGGTAATATCG
GGTCTGAAATCCCCTGACCCAACGGGAGGCGGTACGTGTGATCAGGATCTGCACCTTG
CGAGAGCCAAGCGTAATGACGGTGGCTCTTCCACAAGTAAAAGCTCGGGCGCGAGCG
GCGGGCTGGATGGTCCCCCGAATGTTCCCCCCTATGCCGAAGGCTGAACTCGAGGCTG
AGCCGTGGGCGCGCACCGTTGCCACGCAGGTGCACCGCGAGGTCCCTTGAAGGTCCCG
TCACGAAG

>GST_R05.1

GTTGGGATCTCTTCTCGACTCTGGCACGGCGCGGCGCCTTCGCGCACGGATCGGATAA
GCTTTCCCGTGGACGGCTCGTCCTCGGAAATATCTCGTAGTCTTCTCGTTGGTTGCTCCA
ACGAAGCGACGAATCTACTTGGATACTGAACCGCGAGAGCTATTCAAGTGCTCTACTA
CTCGACCGGGGGCAGCATACAGCCGTGGGATCGGCGCGGCCCACCTCCGGCCAACGAG
AGGTACGAGTACCCGGCCCGTTTTCCCGCGTCGGAGGCTGCCAACACGATAGTAGCTG
AACTGGCCCAAACGTCTTGATACGCCCCGGGGGCGGGCCTCTGGCGCGCCACTGGATC
AGGCCCGTGGCGTGCCCGCCTCGTCAGCGCCCCCCATTGCTAAGCGCTGACAGTAATA
GACCCCTCCATAGTAGTTGCCGATGTTGATTTGGTCACCGGCCGAAACGTATGCGCTCA
GCACAGGGCAGGTACTACGGAGCGAAAGGTGGATGATTGGCAGGGGCCGCTGGCGCA
CCTACAAAACTATTCGTCCGCGCCTGCTGGAGCGACCAACTACGCTCTATAGCGTCCAG
TACCCGAGCAGTCCCTCAATTGGTCCGATGAGACTGTATACGCCGCCGTTGGGACGCG
GACTAAACAACCCCCTCATACCCATTCGCCCGTCCGGAGCGGTACGACTCCGCGGTGC
CGGCGTCAGGCCTCCTCGCCTAGGCTGCGCCATTATGTGGGGAGGTGCGTTGACTGGG
GGCCGGCGACCTGAGCTTGGTCGGCCCGTGAAGCCCATCACTCCCATGGAGACGTTTT
CTCCCATCCATTGGCCCCCGGGGTCCTCCACCAATTGCGTCGGAGTCTTGTCTAATATG
AAATTTAATCATCGTAAGGTATAAACCGCCCCCGCGTGGGTGCGGCTCGAAGTCCGGC
TTCCCAAGCTGGCTGCTAACACGGAAGTGTACCCGGTTCCACTCAGAGGGCCTGAGGC
AGTACCTACGGATCTCAAAATCCCTAGTCCCCATGATACGTACAGCTGGGCTAGGTGG
GATTAATACGTCGGGGTCCCATCGCGGGATGTTTGAGGGGGAGATACGTTCCAGCCAT
ATACTCCCTGTTCGCGCCCCTCAGAGAAGTCCATTACGCTGCCGTTGACGTCGTCAATG
CCGCGCCACTAACACCGGACTAGGGGGACCAAACCATACCATGTAATACGTGTACTTG
CCCATTGTCGCGGTTCAGAGTACGGGGCTGTGAGTAATGTACGCGTTCCTGGTGGGAA
CTTGTCCTCTGAACTTGAATAACAGCTCACTGGAATGGGATTTAGCAAACCATCCCAAC
ATATTCCAGGCCTCTGATCAGATCACAATTTTGCTTTCACCAGTTCCCAATTATGATTCC
GCATCGTCGGGAGGCCTTCGACTAGATCTGCTCGCATGGTTCGAGCGGTAATATCGGG
TCTGAAATCCCCTGACCCAACGGGAGGCGGTATGCGTGATCGGAATCTGCACCTTGCG
AGAGCTAATCGTGATGACAATGGCTCTTCCACAAGTGAGTGCGGGGGCGCGAGCGGCG
AACCAGATGGTCCTCTGAATATTCCCCCCCGTGCCGAAGGCTGAACTCGAGGCGGGGC
CGTGGGCGCGCACCGTTACCACGCAGGTGCGTCGCGAGGCCCCTTGAAGGTCCCGTCA
TGAAG

>GST_R05.2

GTCGGGATGCCCTCCCGGCTCTGGCACCGCGCGGTGGCTTCGCATGCGTATCGGATAA
GCTTTCCCGTGGACCGCTCAACCCCGGAAATACCCCGTAGTTTTCTCGTTGGTTACCCT
GATGCCGCGCCGCGTCTGCTTGGATGCTGAACCGCGAGAGCTATTCAGGTGCTCGACT
ACTCGACCGGGGGCAGCACGCAGCCGCGGGAACGACGCGGTCTACCTCCGGCCAGCA
GGGGGTACGACGTCCCGGCCCGTTTTCCCGCGTCGGAGGCTGCCAACACGATAGTAAC
TGAAATGGTCCAAACGTATTAATACGCACAGGGGGTGGGCCTCTGGCGCGCCACTGGA
TCAGGCCCGTGGCGTGCCCTCCCCCTCAGCGCCACCCATTGCTAAGCGCTGACAGTAAT
AGGCCCCTCCATAGTAGTTGCCGATGTTGATTCGGTCACCGGCCGAAACGTATGCACTT
AGCACAGGGCAGGTACTACAAAGCGAGAGGGGGATGATTGGCAGGGGCTGCTGACGC
GCCTATCAGCCCGTTCCCCCGCGCCTGCTGTGGCGACCAACTACGCCCCGCAGCGTCCA

GTACCCGAGCAGTCTCTCAACTGGTCCGATTAGACTGTATACACCGCCGTTGGGACGC
GGACTAAACAACTCCCTCATACCCATCCGCCCGTCCGGAGCGGAACGACTCGGCGGTA
CCGGCGTCAGGCCCCCTCGCCTAGACGGCACCATTATGTTGGGAGGTGCGTCGACTGG
GGGCCGGCGACTTGATCCTGGTCGGCCCGTGAAGCCCATCAGTCCCATGGAGACGTTC
TCTCCCATCTATTGGCCTCCGGGGCTCTCCACCAATCGCACCGGAGTCTTGTCTAATAT
GAAATTTATTCATCGTGAGGTATAAACCGCCCCCGAGTGGGTGCGGCTTGAAGTCCAG
CCCCCCAGGCCGCCTGCTAACACGGAAGTGTACCCGGTTCCACTCAGAGGGCATGAGG
CAGTACCTACGGATCTCAACAAGCCTAGTCCCCATGATACGTACAGCTGGGCCAGGTG
GGATTAATACGCCGGGGTTCCGTTGCGGAACGGCTAGAAGGTAAATACGCCTTGGCCA
TATACTCTTTGTCCGCATCCCTTGGGGAAATCCATTATGCTGCCGTTGACGTCGTCAAC
GTCACGCCACTAACACCGAACTAGGGGGACCAAACTATACCATGTTATCCGTATACTT
ACCCACTGTTGCAGGTCAAGGTATGGGGCTGCAAGTAATGTACATACCCCTGGTGGGA
ACTTGTCCTCCGGACTTGAATGGCTACCTGCCGGGCTGGGACTTAGCAAACCACCCTGA
CGCATCCCAGGCCTTTGATCGGATCACAATTTTGCTTTCACCAGTTCCCAATTACGGTT
CCGCATCGTCGGGAGGCCTTCGACTAGATCTACTCGCATGGTTCGAGCAGTAATATCG
GGTCTGAAATCCCCTGACCCAACGGAGGGCAGTATGTGTGGTCGGAATCTGCACCTTG
CGAGAGATAATCGTAATGACGATGGACCTTCTACAAGTGAGTGCTGGGGCACGAGCGG
CTGACCAGATTGTCTTCCGAATGTTCCCCCCCGTGCCGAAGGCTGAACCCGAGGCGGA
GCCGTGGGCGTGCACCGTTGCCACGCAGGTGCACCGCGAGGCACCTTGAAGGTCCCGT
CATGAAG

>HT_HTDE12.1

GTCGGGATGCCTTCTCGACTCCGGCACGGCGTGGCACCTTCGTGCACGGATGGGATAA
GCTTTCCCGTAGATGGCTCGTTCTCAGAAATATCTCGTAGTCTTCTCGTTGGTTACTCCA
ATGAAGCGCCTCGTCTGCGGGGGCACTGAACCGCGAGAGCTATTCAAGTGCTCTACTA
CTCGACCGGGGGCAGCTTACAACCGCGGGATCGGCACGGTCTACCTCTGTCCAACAGG
GGGTATGACGACCCGGCCCGTTTTCCCGCGTCGGAGGCTGCCAACACGATAGTAACTG
AACTGGCCCAAACGTATTAATACGCCCCGGGGGCGGGCCTCTGGCGCGCCACTGGATC
AGGCCCGTGGCGTGCCCGCCTCGTCAGCGCCACCCATTGCTAAGCGCTGACAGTAATA
GACCCCTCCATAGTAGTTGCCGATGTTGATTTGGTCACCGGCCGAAACGTATGCGCTCA
GCACAGGGCAGGTACTACGGAGCGAAAGGTGGATGATTGGCAGGGGCCGCTGGCGCA
CCTACAAAACTATTCGTCCGCGCCTGCTGGAGCGACCAACTACGCTCTATAGCGTCCAG
TACCCGAGCAGTCCCTCAACTGGTCCGATTAGACTGTATACACCGCCGTTGGGACGCG
GACTAAACAACTCCCTCATACCCATCCGCCCGTCCGGAGCGGAACGACTCGGCGGTAC
CGGCGTCAGGCCCCCTCGCCTAGACTGCACCATTATGTTGGGAGGTGCGTCGACTGGG
GGCCGGCGACTTGAGGTCGGCCGTTTCGTGAAGCCCATTAGTCCCATGGAGACGTTCTC
TCCCATCCATTGGCCTCCGGGGCTCTTCATCAATCGCGTCGGAGTATCGTCTAATGTGA
AATTTATTCATCGTGAGGTATACACCGCCCCCGCGTGGGTGCGGCTCGAAGTCCGGCCC
CCCAGGCTGCCTGCTAACACGGAAGTATGCCTCGTTTAGCTCAGAAGGGACGAGACAC
TCTCGACGGCTCTCAACAAGCCTAGTCCCCACGGTACATACTACCGTGCCAGGTGGGA
TTGGTACGCCGGGGTCCCGTCACAGAACGGCTAGAAGGTAAATACGCCCTGGCCATAT
ACTCCTTGTCCGCATCCCTTGGGGAAATCCATTACGCCGTCGTTGACGTCATCAATGCC
GCGCGATTAACACCGAGCTGATGGGACCAAAACATACCATGTAATCCGTGTACTTACC
CATCGTTGCGGTACAGGATACGGGGCTGTGAGTAATGTACACACTCCTGGAGGGAGCT
TGTCCTCCAGACTTGAATGGCTACCTGCCGGGCTGGGACCTAGCAAACCACCCTGACG
CATCCCAGGCCTTTGATCGGATCACAATTTTGCTTTCACCAGTTCCCAATTACGGTTCC
GCATCGTCGGGAGGCCTTCGACTAGATCTACTCGCATGGTTCGAGCAGTAATATCGGG
TCTGAAATCCCCTGACCCAACGGGGGGCAGTATGTGTGGTCGGAATCTGCACCTTGCG
AGAGATAATCGTAATGACGATGGACCTTCTACAAGTGAGTGCTGGGGCACGAGCGGCG
GACCAGATTGTCTTTCGAATGTTCCCCCCCGTGCCGAAGGCTGAACCCGAGGCGGAGC

CGTGGGCGTGCACCGTTGCCACGCAGGTGCACCGCGAGGCACCTTGAATGTCCCGTCA
TGACG

>HT_HTDE12.2

GTCGGGATGCCTTCCCAACTCCGGCACGGCGCGGCGCCTTCGCGCACGGATCGGATAA
GCTTTCCCGTAGATGGCTCGTTCTCAGAAATATCTCGTAGTCTTCTCGTTGGTTACTCCA
ATGAAGCGCCTCGTCTGCGGGGGCACTGAACCGCGAGAGCTATTCAAGTGCTCTACTA
TTCCACCGGGGGCAGCACGCAGCCGTGGGATCGGTGCGACCCTCTTTCGGCTAGTAGG
GGGTACGACGTCCCGGCCCGTTTTCCCGCGTCGGAGGCTGCCAACACGGCAGTAACTG
AACTGGCCCAAACGTATTAATACGCACAGGGGGCGGGCCTCTGGCGCGCCACTGGATC
AGGCCCGTGGCGTGCCCGCCTCGTCAGCGCCACCCATTGCTAAGCGCTGACAGTAATA
GACCCCTCCATAGTAGTTGCCGATGTTGATTTGGTCACCGGCCGAAACGTATGCGCTCA
GCACAGGGCAGGTACTACGGAGCGAAAGGTGGATGATTGCCAGGGGCCGCTGGCGCA
CCTACAAAACTATTCGTCCGCGCCTGCTGGAGCGACCAACTACGCTCTATAGCGTCCAG
TACCCGAGCAGTCCCTCAACTGGTCCGATTAGACTGTATACACCGCCGTTGGGACGCG
GACTAAACAACTCCCTCATACCCATCCGCCCGTCCGGAGCGGAACGACTCGGCGGTAC
CGGCGTCAGGCCCCCTCGCCTAGACTGCACCATTATGTTGGGAGGTGCGTCGACTGGG
GGCCGGCGACTTGAGGTCGGCCGTTTCGTGAAGCCCATTAGTCCCATGGAGACGTTCTC
TCCCATCCATTGGCCTCCGGGGCTCTTCATCAATCGCGTCGGAGTATCGTCTAATGTGA
AATTTATTCATCGTGAGGTATACACCGCCCCCGCGTGGGTGCGGCTCGAAGTCCGGCCC
CCCAGGCTGCCTGCTAACACGGAAGTATGCCTCGTTTAGCTCAGAAGGGACGAGACAC
TCTCGACGGCTCTCAACAAGCCTCGTCCCCACGGTACATACTACCGTGCCAGGTGGGAT
TGGTACGCCGGGGTCCCGTCACAGAACGGCTAGAAGGTAAATACGCCCTGGCCATATA
CTCCTTGACCGCATCCCTTGGGGAAATCCATTACGCCGTCGTTGACGTCATCAATGCCG
CGCGATTAACACCGAGCTGATGGGACCAAAACATACCAGGTAATCCGTGTACTTACCC
ATCGTTGCGGTACAGGATACGGGGCTGTGAGTAATGTACACACTCCTGGAGGGAGCTT
GTCCTCCAGACTTGAATGGCTACCTGCCGGGCTGGGACTCAGCAAACCACCCTGACGC
ATCCCAGGCCTTTGATCGGATCACAATTTTGCTTTCACCAGTTCCCAATTACGGTTCCG
CATCGTCGGGAGGCCTTCGACTAGATCTACTCGCATGGTTCGAGCAGTAATATCGGGTC
TGAAATCCCCTGACCCAACGGGGGGCAGTATGTGTGGTCGGAATCTGCACCTTGCGAG
AGATAATCGTAATGACGATGGACCTTCTTGAAGTGAGTGCTGGGGCACGAGCGGCGGA
CCAGATTGTCTTTCGAATGTTCCCCCCCGTGCCGAAGGCTGAACCCGAGGCGGAGCCG
TGGGCGTGCACCGTTGCCACGCAGGTGCACCGCGAGGCACCTTGAATGTCCCGTCATG
AAG

>HT_HTDE70.1

GTCGGGATGCCCTTTCGACACCAGCACGGCGCGACGCTTTCGCGCACGTATCAGATAA
GTTTTCCCGTGGACGGCTCGTCCTCAGAAATATCTCGTAGTCCTTTCGTTGGTTACTCCA
ATGAAGCGCCTCGCCTGCGGGAGCACTAAACCGCGAGCGCCATTCGAGTGCTCTACAA
CTCGACCGGGGGCAGCACACAACCGTAGGATCGGCGCGGCTCATTTTCGGCTAGTAGG
GGGTACGACGTCCCGGCCCGTTTTCTCGCGTCGGAGGCTGCCAACACGATAGTAACTG
AACTGGCCCAAACGTATTGATACGCCCAGGGGGCGGGCCTCTGGCGCGCCACTGGATC
AGGCCCGTGGCGTGCCCGCCTCGTCAGCGCCACCCATTGCTAAGCGCTGACAGTAATA
GACCCCTCCATAGTAGTTGCCGATGTTAATTCGGTCACCGGCCGAAACGTATGCACTTA
GCACAGGGCAGGTACTACAAAGCGAGAGGTGGACGATTGGCAAAGGCTGCTGGCGAG
CCTACCAACCTGTTCCTCCGCGCCTGCTGGAGCGACCAACTACGCCCCGCAGCGACCG
GTACTTGAGCTGTCTCTCAACTGGTTCGATGAGACTGTATACACCGTCGTTGGGACGCG
GACTAAACAACCTCCTCATACCAATCCGCCCGTCCGGAGCGGAACGACTCGGCGGTAC
CGGCGTCAGGCCCCCTCGCCTAGGCTACACCATTATGTTGGGAGGTGCGTCGACTGGG
GGCCGGCGACTTGAGCTCGGTCGTCCCGTGAAGCCCATCAGTCCCATGGAGACGTTCT

CTCTCATCCATTGGCCTCCGGGGCTCTCCACCAATCGCACCGGAGTCTTGTCTAGTGTT
AAATGTATTCATCGTGGGGTATAAACCGCCCCCGCGTGAGTGCGGCTCGAAGTCTGGC
CTCCCAGGCTGCTAGCTAACACGGAAGTGTGCCTCGTTTCGCTCAGAGGGGACGAGAC
ACTCTCGACGGCTCTCAACAAGCCTAGTCCCCACGATACATACAACCGTGCCCGGTGG
GATTAATACGCCGGGGTCCCGTCGCGGGACATCTGGGATGGGAACACGTTCCGGCCAT
ATGCTCTCTGTCCGCATCTCTTGGGGAAATCCATTACGCTGCCGTTGACGTCGTTAACG
TCACGCCACTAACACCGAACTAGGGGGACCAAACTATACCATGTAATCCGTGCGCCTA
CCCACTGTTGCGATTCAGGGTACGGGGCTGTGAGTAATGTACACACTCCTGGTGTGAG
CTTGTCCTCCGGACTTGAATGGCTACTCGCCGGGCTGGGACTTAGCAAACCACCCTGAC
GCATTCCAGGCCTCTGATCAGATCACAATTTTGCTTTCACCAGTTCCCAATTACGGTTC
CGCATCGTCGGGAGGCCTTCGACTAGATCTACTCGCATGGTTCGAGCGGTAATATCGG
GTCTGAAATCCCCTGACCCAACGGGAGGCGGTACGTGTGATCAGGATCTGCACCTTGC
GAGAGCCAAGCGTAATGACGGTGGCTCTTCCACCTCTAAAAGCTCGGGCGCGAGCGGC
GGGCTGGATGGTCCCCCGAATGTTCCCCCCTATGCCGAAGGCTGAACTCGAGGCTGAG
CCGTGGGCGCGCACCGTTGCCACGCAGGTGCACCGCGAGGTCCCTTGAAGGTCCCGTC
ACGAAG

>HT_HTDE70.2

GTCGGGATGCTTTCCCAACTCCGGCACGGCGCGGCGCCTTCGCGCACGGATCGGATAA
GCTTTCCCGTGGACGGCTCGTCCTCAGAAACATTTCGTAGTCTTCTCGTTGGTTACTCCA
CTGCCGCGCCGCGTCTGCTTGGATGTTGAACCGCGAGAGCTATTCAAGTGCTTTACTAC
TCGACCGAGGGCAGCTTACAACCGTGGGATCGGCGTGGCCCACCTCCGGCCAACGAGA
GTTACGAGTACCCGGCCCGTTTTCCCGCGTCGGAGGCTGCCAACACGATAGTAGCTGA
ACTGGCCCAGACGTATTGATACGCCCCGGGGGCGGGCCTCTGGCGCGCCACTGGATCA
GGCCCGTGGCGGGCCTGCCTCGTCAGCGCCACCCATTGCTAAGCGCTGACAGTAATAG
ACCCCTCCATAGTAGTTGCCGATGTTGATTTGGTCACCGGCCGAAACGTATGCGCTCAG
CACAGGGCAGGTACTACGGAGCGAAAGGTGGATGATTGGCAGGGGCCGCTGGCGCAC
CTACAAAACTATTCGTCCGCGCCTGCTGGAGCGACCAACTACGCTCTATAGCGTCCAGT
ACCCGAGCAGTCTCTCAACTGGTCCGATTAGACTGTATACACCGCCGTTGGGACGCGG
ACTAAACAATTCCCTCATACCCATCCGCCCGTCCGGAGCGGAACGACTCGACGGTACC
GGCGTCAGGCCCCCTCGCCTAGGCTGCACCATTATGTTGGAAGGTACATCGACTGGGG
GCCGGCAACTTGAGCTTGGTCGGCCCGTGAAACCCACCAGTCCCATGGAGAAGTTCTC
TCCCATCCAGTGGCCTCCGGGGCTCTTCATCAATCGCGTCGGAGTATCGTCTAATGTGA
AATTTATTCATCGTGAGGTATACACCGCCCCCGCGTGGGTGCGGCTCGAAGCCCGGCTT
CCCAGGCTGGCAGCTAACACGGAAGTGTGCCTCGTTTCGCTCAGAAGGGACGGACAC
TCTCGACGGCCCTCAACAAGCCTAGTCCCCACGATACATACAACCGTGCCAGGTGGAA
TTAATACGTCGGGGTCCCATCGCGGGATGTTTGAGGGGGAGATACGTTCCGGCCATAT
ACTCCCTGTTCGCGCCCCTCAGAGAAGTCCATTACGCTGCCGTTGACGTCGTCAATGCC
GCGCCACTAACACCGGACTAGGGGGACCAAACCATACCATGTAATCCGTGTACTTGCC
CATTGTTACGGTTTAGGGAACGGGGCTGTGGCTAATAGACGCGCTCCTGGTGGGAGCT
TGTCCTCCGGACTCGAATGGCTACTCGCCGGGCTGGGACTTAGCAAACCACTCTGACG
CATCTACGGCCTCAGATTAGATCACAATTTTGCTTTCTCCATTTCCTAATTACCGTTCCG
CATCGTCGGGAGGCCTTCGACTAGATTTACTCGCATGGTTTAAGTGATAATATCGGGTC
GGGAATCCTCTGACCCAACGTGAAGCGGTATGCGCAATTGGGGTCTGTTCCTTGCAAG
AGCTAATCGTAATGACGATGGACCTTCTACAAGTGAGTGCTGTAGCGCCAGCAGCGGA
CCAGATTGTTTTCCGGATGTTCCCCCTCGTGCCGAAGGCTGAACTCAGGGCGGGGCCGT
GGGCGCGCATCGTTGCCACGCAGGTACACCGTGAGGCACCTTGAAGGTCCCGTCATGA
AC

>HT_HTDE125.1

GTCGGGATGCCCTTTCGACACCAGCACGGCGCGACGCTTTCGCGCACGTATCGGATAA
ACTTTCCATTGGACGGCTCGTTCTCAGAAATATCTCGTAGTCTTCTCGTTGGTTACTCCA
ATGAAACGCCGCGTCTGCGGGGGCGCTGAACCGCGAGAGCTATTCAAATGCTCTACTA
CTCGACCGGGGGCAGCTTACAACCGTGGGATCGGCGCGGCCCACCTCCGGTCAACGAG
AGTTACGAGTTCCCGGCCCGTTTTCTCGCGTCGGAGGCTGCCAACACGATAGTAACTGA
ACTGGCCCAAACGTATTAATACGCCCCGGGGGCGGGCCTCTGGCGCGCCACTGGATCA
GGCCCGTGGCGTGCCCGCCTCGTCAGCGCCACCCATTGCTAAGCGCTGACAGTAATAG
ACCCCTCCATAGTAGTTGCCGATGTTGATTCGGTCACCGGCCGAAACGTATGCACTTAG
CACAGGGCAGGTACTACAAGGCGAGAGGGGGATGATTGGCAGGGGCTGCTGACGCGC
CTATCAACCCGTTCCCCCGCGTCTGCTGTGGCGACCAACTACGCCCCGCAGCGTCCAGT
ACCCGAGCAGTCTCTCAACTGGTCCGATTAGACGGTATACACCGCCGTTGGGACGCGG
ACTAAACAACTCCCTCATACCCATCCGCCCGTCCGGAGCGGAACGACTCGGCGGTACC
GGCGTCAGGCCCCCTCGCCTAGACTGCACCATTATGTTGGGAGGTGCGTCGACTGGGG
GCCGGCGACTTGATCCTGGTCGGCCCGTGAAGCCCATCAGTCCCATGGAGACGTTCTCT
CCCATCTATTGACCTCCGGGGCTCTCCACCAATCGCACCGGAGTCTTGTCTAATATGAA
ATTTATTCATCGTGAGGTATAAACCGCCCCCGAGTGGGTGCGGCTTGAAGTCCGGCCCC
CCAGGCCGCCTGCTAACACGGAAGTGTACCCGGTTCCACTCAGAGGGCATGAGGCAGT
ACCTACGGATCTCAACAAGCCTAGTCCCCATGATACGTACAGCTGGGCCAGGTGGGAT
TAATACGCCGGGGTCCCGCCGCGAAACGGCTAGAAGGTAAATACGCCCTGGCCATATA
CTCTTTGTCCGCATCCCTTGGGGACATCCATCATGCTGCCGTTGACGTCGTCAACGTCA
CGCCACTAACACCGAACTAGGGGGACCAAACTATACCATGTTATCCGTATACTTACCC
ACTGTTGCAGGTCAAGGTTTGGGGCTGCAAGTAATGTACATACTCCTGGTGGGAACTT
GTCCTCCGGACTTGAATGGCTACCTGCCGGGCTGGGACTTAGCAAACCACCCTGACGC
ATCCCAGGCCTTTGATCGGATCACAATTTTGATTCCACCAGTTCCCAATTACGGTTCCG
CATCGTTGGGAGTCCTTTGGCTAGATCTACCTGCATGGTTCGAGCGGTAATATCGGGTC
TGAAATCCCCTGACCCAACGGGAGGCAGTATGTGTGGTCGGAATCTGCACCTTGCGAG
AGATAATCGTAATGACGATGGACCTTCTACAAGTGAGTGCTGGGGCACGAGCGGCGGA
CCAGATGGTCTTCTGAATGTTCCTTCCCGTGCCGAAGGCTGAACTCGAGGCGGAGCAG
TGGGCGCGTACCGTCGACATGCAGGTGCACCGCGAGGTCCCTTGAAGATCCCGTCATC
AAC

>HT_HTDE125.2

GTCGGGATGCTTTCCCAACTCCGGCACGGCGCGGCGCCTTCGCGCACGGATCGGATAA
GCTTTCCCGTGGGCGGCTCGTCCTCAGAAACATTTCGTAGTCTTCTCGTTGGTTACTCCA
CTGCCGCGCCGCGTCTGCTTGGATGTTGACCCGCGAGAGCTATTCAAGTGCTTTACTAC
TCGACCGAGGGCAGCTTACAACCGTGGGATCGGCGTGGCCCACCTCCGGCCAACGAGA
GTTACGAGTACCCGGCCCGTTTTCCCGCGTCGGAGGCTGCCAACACGATAGTAGCTGA
ACTGGCCCAGACGTATTGATACGCCCCGGGGGCGGGCCTCTGGCGCGCCACTGGATCA
GGCCCGTGGCGTGCCTGCCTCGTCAGCGCCACCCATTGCTAAGCGCTGACAGTAATAG
ACCCCTCCATAGTAGTTGCCGATGTTAATTCGGTCACCGGCCGAAACGTATGCACTTAG
CATAGGGCAGGTACTACAAAGCGAGAGGTGGACGATTGGCAAAGGCTGCTGGCGAGC
CTACCAACCTGTTCCTCCGCGCCTGCTGGAGCGACCAACTACGCCCCGCAGCGACCGG
TACTTGAGCTGTCTCTCAACTGGTTCGATGAGACTGTATACACCGTCGTTGGGACGCGG
ACTAAACAACCTCCTCATACCAATCCGCCCGTCCGGAGCGGAACGACTCGGCGGTACC
GGCGTCAGGCCCCCTCGCCTAGGCTACACCATTATGTTGGGAGGTGCGTCGACTGGGG
GCCGGCGACTTGAGCTCGGTCGTCCCGTGAAGCCCATCAGTCCCATGGAGACGTTCTCT
CTCATCCATTGGCCTCCGGGGCTCTCCACCAATCGCACCGGAGTCTTGTCTAGTGTTAA
ATGTATTCATCGTGGGGTATAAACCGCCCCCGCGTGAGTGCGGCTCGAAGTCTGGCCTC
CCAGGCTGCTAGCTAACACGGAAGTGTGCCTCGTTTCGCTCAGAGGGGACGAGACACT
CTCGACGGCTCTCAACAAGCCTAGTCCCCACGATACATACAACCGTGCCCGGTGGGAT
TAATACGCCGGGGTCCCGTCGCGGGACATCTGGGATGGGAACACGTTCCGGCCATATG

CTCTCTGTCCGCATCTCTTGGGGAAATCCATTACGCTGCCGTTGACGTCGTTAACGTCA
CGCCACTAACACCGAACTAGGGGGACCAAACTATACCATGTAATCCGTGCGCCTACCC
ACTGTTGCGATTCAGGGTACGGGGCTGTGAGTAATGTACACACTCCTGGTGTGAGCTTG
TCCTCCGGACTTGAATGGCTACTCGCCGGGCTGGGACTTAGCAAACCACCCTGACGCA
TTCCAGGCCTCTGATCAGATCACAATTTTGCTTTCACCAGTTCCCAATTACGGTTCCGC
ATCGTCGGGAGGCCTTCGACTAGATCTACTCGCATGGTTCGAGCGGTAATATCGGGTCT
GAAATCCCCTGACCCAACGGGAGGCGGTACGTGTGATCAGGATCTGCACCTTGCGAGA
GCCAAGCGTAATGACGGTGGCTCTTCCACCTCTAAAAGCTCGGGCGCGAGCGGCGGGC
TGGATGGTCCCCCGAATGTTCCCCCCTATGCCGAAGGCTGAACTCGAGGCTGAGCCGT
GGGCGCGCACCGTTGCCACGCAGGTGCACCGCGAGGTCCCTTGAAGGCCCCGTCACGA
AG

>HT_HTDE201.1

GTCGGGATGCCCTTTCGACACCAGGACGGCGCGACGCTTTCGCGCACGTATCAGATAA
GTTTTCCCGTGGACGGCTCGTCCTCAGAAATATCTCGTAGTCCTTTCGTTGGTTACTCCA
ATGAAGCGCCTCGCCTGCGGGAGCACTAAACCGCGAGCGCCATTCGAGTGCTCTACAA
CTCGACCGGGGGCAGCACACAACCGTAGGATCGGCGCGGCTCATTTTCGGCTAGTAGG
GGGTACGACGTCCCGGCCCGTTTTCTCGCGTCGGAGGCTGCCAACACGATAGTAACTG
AACTGGCCCAAACGTATTGATTCGCCCAGGGGGCGGGCCTCTGGCGCGCCACTGGATC
AGGCCCGTGGCGTGCCCGCCTCGTCAGCGCCACCCATTGCTAAGCGCTGACAGTAATA
GACCCCTCCATAGTAGTTGCCGATGTTGATTTGGTCACCGGCCGAAACGTATGCGCTCA
GCACAGGGCAGGTACTACGGAGCGAAAGGTGGATGATTGGCAGGGGCCGCTGGCGCA
CCTACAAAACTATTCGTCCGCGCCTGCTGGAGCGACCAACTACGCTCTATAGCGTCCAG
TACCCGAGCAGTCTCTCAACTGGTCCGATTAGACTGTATACACCGCCGTTGGGACGCG
GACTAAACAATTCCCTCATACCCATCCGCCCGTCCGGAGCGGAACGACTCGACGGTAC
CGGCGTCAGGCCCCCTCGCCTAGGCTGCACCATTATGTTGGAAGGTACATCGACTGGG
GGCCGGCAACTTGAGCTTGGTCGGCCCGTGAAACCCACCAGTCCCATGGAGAAGTTCT
CTCCCATCCAGTGGCCTCCGGGGCTCTTCATCAATCGCGTCGGAGTATCGTCTAATGTG
AAATTTATTCATCGTGAGGTATACACCGCCCCCGCGTGGGTGCGGCTCGAAGCCCGGC
TTCCCAGGCTGGCAGCTAACACGGAAGTGTGCCTCGTTTCGCTCAGAAGGGACGGGAC
ACTCTCGACGGCCCTCAACAAGCCTAGTCCCCACGATACATACAACCGTGCCAGGTGG
AATTAATACGTCGGGGTCCCATCGCGGGATGTTTGAGGGGGAGATACGTTCCGGCCAT
ATACTCCCTGTTCGCGCCCCTCAGAGAAGTCCATTACGCTGCCGTTGACGTCGTCAATG
CCGCGCCACTAACACCGGACTAGGGGGACCAAACCATACCATGTAATCCGTGTACTTG
CCCATTGTTACGGTTTAGGGAACGGGGCTGTGGCTAATAGACGCGCTCCTGGTGGGAG
CTTGTCCTCCGGACTCGAATGGCTACTCGCCGGGCTGGGACTTAGCAAACCACTCTGAC
GCATCTACGGCCTCAGATTAGATCACAATTGTGCTTTCTCCATTTCCTAATTACCGTTCC
GCATCGTCGGGAGGCCTTCGACTAGATTTACTCGCATGGGTCGAGCGATAATATCAAG
TCTGGAATCCTCTGACCCAACGTGAAGCGGTATGCGCAATTGGGGTCTGTTCCTTGCAA
GAGCTAATCGTAATGACGATGGACCTTCTACAAGTGAGTGCTGTAGCGCCAGCAGCGG
ACCAGCTTGTTTTCCGGATGTTCCCCCTCGTGCCGAAGGCTGCACTCAGGGCGGGGCCG
TGGGCGCGCATCGTTGCCACGCAGGTACACCGTGAGGCACCTTGAAGGTCCCGTCATG
AAC

>HT_HTDE201.2

GTCGGGATGCCCTTTCGACACCAGCACGGCGCGACGCTTTCGCGCACGTATCGGATAA
ACTTTCCATTGGACGGCTCGTTCTCAGAAATATCTCGTAGTCTTCTCGTTGGTTACTCCA
ATGAAACGCCTCGTCTGCGGGGGCACTGAACCGCGAGAGCTATTCAAATGCTCTACTA
CTCGACCGGGGGCAGCTTACAACCGTGGGATCGGCGCGGCCCACCTCCGGTCAACGAG
AGTTACGAGTTCCCGGCCCGTTTTCTCGCGTCGGAGGCTGCCAACACGATAGTAACTGA

ACTGGCCCAAACGTATTAATACGCCCCGGGGGCGGGCCTCTGGCGCGCCACGGGATCA
GGCCCGTGGCGTGCCCGCCTCGTCAGCGCCACCCATTGCTAAGCGCTGACAGTCATAG
ACCCCTCCATAGTAGTTGCCGATGTTGATTCGGTCACCGGCCGAAACGTATGCACTTAG
CACAGGGCAGGTACTACAAGGCGAGAGGGGGATGATTGGCAGGGGCTGCTGACGCGC
CTATCAGCCCGTTCCCCCGCGTCTGCTGTGGCGACCAACTACGCCCCGCAGCGTCCAGT
ACCCGAGCAGTCTCTCAACTGGTCCGATTAGACTGTATACACCGCCGTTGGGACGCGG
ACTAAACAACTCCCTCATACCCATCCGCCCGTCCGGAGCGGAACGACTCGGCGGTACC
GGCGTCAGGCCCCCTCGCCTAGACTGCACCATTATGTTGGGAGGTGCGTCGACTGGGG
GCCGGCGACTTGATCCTGGTCGGCCCGTGAAGCCCATCAGTCCCATGGAGACGTTCTCT
CCCATCTATGGACCTCCGGGGCTCTCCACCAATCGCACCGGAGTCTTGTCTAATATGAA
ATTTATTCATCGTGAGGTATAAACCGCCCCCGAGTGGGTGCGGCTTGAAGTCCAGCCCC
CCAGGCCGCCTGCTAACACGGAAGTGTACCCGGTTCCACTCAGAGGGCATGAGGCAGT
ACCTACGGATCTCAACAAGCCTAGTCCCCATGATACGTACAGCTGGGCCAGGTGGGAT
TAATACGCCGGGGTCCCGCCGCGAAACGGCTAGAAGGTAAATACGCCCTGGCCATATA
CTCTTTGTCCGCATCCCTTGGGGAAATCCATTATGCTGCCGTTGACGTCGTCAACGTCA
CGCCACTAACACCGAACTAGGGGGACCAAACTATACCATGTTATCCGTATACTTACCC
ACTGTTGCAGGTCAAGGTATGGGGCTGCAAGTAATGTACATACTCCTGGTGGGAACTT
GTCCTCCGGACTTGAATGGCTACCTGCCGGGCTGGGACTTAGCAAACCACCCTGACGC
ATCCCAGGCCTTTGATCGGATCACAATTTTGATTCCACCAGTTCCCAATTACGGTTCCG
CATCGTTGGGAGGCCTTTGGCTAGATCTACCTGCATGGTTCGAGCGGTAATATCGGGTC
TGAAATCCCCTGACCCAACGGGAGGCAGTATGTGTGGTCGGAATCTGCACCTTGCGAG
AGATAATCGTAATGACGATGGACCTTCTACAAGTGAGTGCTGGGGCACGAGCGGCGGA
CCAGATGGTCTTCTGAATGTTCCTTCCCGTGCCGACGGCTGAACTCGAGGCGGAGCAGT
GGGCGCGTACCGTCGACATGCAGGTGCACCGCGAGGTCCCTTGAAGATCCCGTCATCA
AC

>HT_HTDE314.1

GTCGGGATGCCTTCTCGACTCCGGCACGGCGTGGCACCTTCGTGCACGGATGGGATAA
GCTTTCCCGTAGATGGCTCGTTCTCAGAAATATCTCGTAGTCTTCTCGTTGGTTACTCCA
ATGAAGCGCCTCGTCTGCGGGGGCACTGAACCGCGAGAGCTATTCAAGTGCTCTACTA
CTCGACCGGGGGCAGCTTACAACCGCGGGATTTGCACGGTCTACCTCTGTCCAACAGG
GGGTACGACGACCCGGCCCGTTTTCCCGCGTCGGAGGCTGCCAACACGATAGTAACTG
AACTGGCCCAAACGTATTAATACGCCCCGGGGGCGGGCCTCCGGCGCGCCACGGGATC
AGGCCCGTGGCGTGCCCGCCTCGTCAGCGCCACCCATTGCTAAGCGCTGACAGTAATA
GACCCCTCCATAGTAGTTGCCGATGTTGATTTGGTCACCGGCCGAAACGTATGCGCTCA
GCACAGGGCAGATACTACGGAGCGAAAGGTGGATGATTGGCAGGGGCCGCTGGCGCA
CCTACAAAACTATTCGTCCGCGCCTGCTGGAGCGACCAACTACGCTCTATAGCGTCCAG
TACCCGAGCAGTCCCTCAACTGGTCCGAGTAGACTGTATACACCGCCGTTGGGACGCG
GACTAAACAACTCCCTCATACCCATCCGCCCGTCCGGAGCGGAACGACTCGGCGGTAC
CGGCGTCAGGCCCCCTCGCCTAGACTGCACCATTATGTTGGGAGGTGCGTCGACTGGG
GGCCGGCGACTTGAGGTCGGCCGTTTCGTGAAGCCCATTAGTCCCATGGAGACGTTCTC
TCCCATCCATTGGCCTCCGGGGCTCTTCATCAATCGCGTCGGAGTATCGTCTAATGTGA
AATTTATTCATCGTGAGGTATACACCGCCCCCGCGTGGGTGCGGCTCGAAGTCCGGCCC
CCCAGGCTGCCTGCTAACACGGAAGTATGCCTCGTTTAGCTCAGAAGGGACGAGACAC
TCTCGACGGCTCTCAACAAGCCTAGTCCCCACGGTACATACTACCGTGCCAGGTGGGA
TTGGTACGCCGGGGTCCCGTCACAGAACGGCTAGAAGGTAAATACGCCCTGGCCAGAT
ACTCCTTGTCCGCATCCCTTGGGGAAATCCATTACGCCGTCGTTGACGTCATCAATGCC
GCGCGATTAACACCGAGCTGATGGGACCAAAACATACCATGTAATCCGTGTACTTACC
CATCGTTGCGGTACAGGATACGGGGCTGTGAGTAATGTACACACTCCTGGAGGGAGCT
TGTCCTCCAGACTTGAATGGCTACCTGCCGGGCTGGGACCTAGCAAACCACCCTGACG
CATCCCAGGCCTTTGATCGGATCACAATTTTGCTTTCACCAGTTCCCAATTACGGTTCC

GCATCGTCGGGAGGCCTTCGACTAGATCTACTCGCATGGTTCGAGCAGTAATATCGGG
TCTGAAATCCCCTGACCCAACGGGGGGCAGTATGTGTGGTCGGAATCTGCACCTTGCG
AGAGATAATCGTAATGACGATGGACCTTCTACAAGTGAGTGCTGGGGCACGAGCGGCG
GACCAGATTGTCTTTCGAATGTTCCCCCCCGTGCCGAAGGCTGAACCCGAGGCGGAGC
CGTGGGCGTGCACCGTTGCCACGCAGGTGCACCGCGAGGCACCTTGAATGTCCCGTCA
TGAAG

>HT_HTDE314.2

GTCGGGATGCCCTTTCGACACCAGCACGGCGCGACGCTTTCGCGCACGTATCGGATAA
ACTTTCCATTGGACGGCTCGTTCTCAGAAATATCTCGTAGTCTTCTCGTTGGTTACTCCA
ATGAAACGCCTCGTCTGCGGGGGCACTGAACCGCGAGAGCTATTCAAATGCTCTACTA
CTCGACCGGGGGCAGCTTACAACCGTGGGATCGGCGCGGCCCACCTCCGGTCAACGAG
AGTTACGAGTTCCCGGCCCGTTTTCTCGCGTCGGAGGCTGCCAACACGATAGTAACTGA
ACTGGCCCAAACGTATTAATACGCCCCGGGGGCGGGCCTCTGGCGCGCCACTGGATCA
GGCCCGTGGCGTGCCCGCCTCGTCAGCGCCACCCATTGCTAAGCGCTGACAGTAATAG
ACCCCTCCATAGTAGTTGCCGATGTTGATTCGGTCACCGGCCGAAACGTATGCACTTAG
CACAGGGCAGGTACTACAAAGCGAGAGGGGGATGATTGGCAGGGGCTGCTGACGCGC
CTATCAGCCCGTTCCCCCGCGTCTGCTGTGGCGACCAACTACGCCCCGCAGCGTCCAGT
ACCCGAGCAGTCTCTCAACTGGTCCGATTAGACTGTATACACCGCCGTTGGGACGCGG
ACTAAACAACTCCCTCATACCCATCCGCCCGTCCGGAGCGGAACGACTCGGCGGTACC
GGCGTCAGGCCCCCTCGCCTAGACTGCACCATTATGTTGGGAGGTGCGTCGACTGGGG
GCCGGCGACTTGATCCTGGTCGGCCCGTGAAGCCCATCAGTCCCATGGAGACGGTCTC
TCCCATCTATTGACCTCCGGGGCTCTCCACCAATCGCACCGGAGTCTTGTCTAATATGA
AATTTATTCATCGTGAGGTATAAACCGCCCCCGAGTGGGGGCGGCTTGAAGTCCAGCC
CCCCAGGCCGCCTGCTAACACGGAAGTGTACCCGGTTCCACTCAGAGGGCATGAGGCA
GTACCTACGGATCTCAACAAGCCTAGTCCCCATGATACGTACAGCTGGGCCAGGTGGG
ATTAATACGCCGGGGTCCCGCCGCGAAACGGCTAGAAGGTAAATACGCCCTGGCCATA
TACTCTTTGTCCGCATCCCTTGGGGAAATCCATTATGCTGCCGTTGACGTCGTCAACGT
CACGCCACTAACACCGAACTAGGGGGACCAAACTATACCATGTTATCCGTATACTTAC
CCACTGTTGCAGGTCAAGGTATGGGGCTGCAAGTAATGTACATACTCCTGGTGGGAAC
TTGTCCTCCGGACTTGAATGGCTACCTGCCGGGCTGGGACTTAGCAAACCACCCTGACG
CATCCCAGGCCTTTGATCGGATCACAATTTTGATTCCACCAGTTCCCAATTACGGTTCC
GCATCGTTGGGAGGCCTTTGGCTAGATCTACCTGCATGGTTCGAGCGGTAATATCGGGT
CTGAAATCCCCTGACCCAACGGAGGCAGTATGTGTGGTCGGAATCTGCACCTTGCGA
GAGATAATCGTAATGACGATGGACCTTCTACAAGTGAGTGCTGGGGCACGAGCGGCGG
ACCAGATGGTCTTCTGAATGTTCCTTCCCGTGCCGAAGGCTGAACTCGAGGCGGAGCA
GTGGGCGCGTACCGTCGACATGCAGGTGCACCGCGAGGTCCCTTGAAGATCCCGTCAT
CAAC

>HT_HTDE328.1

GTCGGGATGCCTTCTCGACTCCGGGACGGCGTGGCACCTTCGTGCACGGATCGGATAA
GCTTTCCCGTAGATGGCTCGTTCTCAGAAATATCTCGTAGTCTTCTCGTTGGTTACTCCA
ATGAAGCGCCTCGTCTGCGGGGGCACTGAACCGCGAGAGCTATTCAAGTGCTCTACTA
CTCGACCGGGGGCAGCTTACAACCGCGCGATTGGCACGGTCTACCTCTGTCCAACAGG
GGGTACGACGACCCGGCCCGTTTTCCCGCGTCGGAGGCTGCCAACACGATAGTAACTG
AACTGGCCCAAACGTATTAATACGCCCCGGGGGCGGGCCTCTGGCGCGCCACTGGATC
AGGCCCGTGGCGTGCCCGCCTCGTCAGCGCCACCCATTGCTAAGCGCTGACAGTAATA
GACCCCTCCATAGTAGTTGCCGATGTTGATTTGGTCACCGGCCGAAACGTATGCGCTCA
GCACAGGGCAGGTACTACGGAGCGAAAGGTGGATGATTGGCAGGGGCCGCTGGCGCA
CCTACAAAACTATTCGTCCGCGCCTGCTGGAGCGACCAACTACGCTCTATAGCGTCCAG

TACCCGAGCAGTCCCTCAACTGGTCCGATTAGACTGTATACACCGCCGTTGGGACGCG
GACTAAACAACTCCCTCATACCCATCCGCCCGTCCGGAGCGGAACGACTCGGCGGTAC
CGGCGTCAGGCCCCCTCGCCTAGACTGCACCATTATGTTGGGAGGTGCGTCGACTGGG
GGCCGGCGACTTGAGGTCGGCCGTTTCGTGAAGCCCATTAGTCCCATGGAGACGTTCTC
TCCCATCCATGGGCCTCCGGGGCTCTTCATCAATCGCGTCGGAGTATCGTCTAATGTGA
AATTTATTCATCGTGAGGTATACACCGCCCCCGCGTGGGTGCGGCTCGAAGTCCGGCCC
CCCAGGCTGCCTGCTAACACGGAAGTATGCCTCGTTTAGCTCAGAAGGGACGAGACAC
TCTCGACGGCTCTCGACAAGCCTAGTCCCCACGGTACATACTACCGTGCCAGGAGGGA
TTGGTACGCCGGGGTCCCGTCACAGAACGGCTAGAAGGTAAATACGCCCTGGCCATAT
ACTCCTTGTCCGCATCCCTTGGGGAAATCCATTACGCCGTCGTTGACGTCATCAATGCC
GCGCGATTAACACCGAGCTGATGGGACCAAAACATACCAGGTAATCCGTGTACTTACC
CATCGTTGCGGTACAGGATACGGGGCTGTGAGTAATGTACACACTCCTGGAGGGAGCT
TGTCCTCCAGACTTGAATGGCTACCTGCCGGGCTGGGACCTAGCAAACCACCCTGACG
CATCCCAGGCCTTTGATCGGATCACAATTTTGCTTTCACCAGTTCCCAATTACGGTTCC
GCATCGTCGGGAGGCCTTCGACTAGATCTACTCGCATGGTTCGAGCAGTAATATCGGG
TCTGAAATCCCCTGACCCAACGGGGGGCAGTATGTGTGGTCGGAATCTGCACCTTGCG
AGAGATAATCGTAATGACGATGGACCTTCTACAAGTGAGTGCTGGGGCACGAGCGGCG
GACCAGATTGTCTTTCGAATGTTCCCCCCCGTGCCGAAGGCTGAACCCGAGGCGGAGC
CGTGGGCGTGCCCCGTTGCCACGCAGGTGCACCGCGAGGCACCTTGAATGTCCCGTCA
TGAAG

>HT_HTDE328.2

GTCGGGATGCCTTCTCGACTCCGGCACGGCGTGGCACCTTCGTGCACGGATGGGATAA
GCTTTCCCGTAGATGGCTCGTTCTCAGAAATATCTCGTAGTCTTCTCGTTGGTTACTCCA
ATGAAGCGCCTCGTCTGCGGGGGCACTGAACCGCGAGAGCTATTCAAGTGCTCTACTA
CTCGACCGGGGGCAGCTTACAACCGCGGGATTTGCACGGTCTACCTCTGTCCAACAGG
GGGTACGACGACCCGGCCCGTTTTCCCGCGTCGGAGGCTGCCAACACGATAGTAACTG
AACTGGCCCAAACGTATTAATACGCCCCGGGGGCGGGCCTCTGGCGCGCCACTGGATC
AGCCCCGTGGCGTGCCCGCCTCGTCAGCGCCACCCATTGCTAAGCGCTGACAGTAATA
GACCCCTCCATAGTAGCTGCCGATGTTGATTTGGTCACCGGCCGAAACGTATGCGCTCA
GCACAGGGCAGATACTACGGAGCGAAAGGTGGATGATTGGCAGGGGCCGCTGGCGCA
CCTACAAAACTATTCGTCCGCGCCTGCTGGAGCGACCAACTACGCTCTATAGCGTCCAG
TACCCGAGCAGTCCCTCAACTGGTCCGATTAGACTGTATACACCGCCGTTGGGACGCG
GACTAAACAACTCCCTCATACCCATCCGCCCGTCCGGAGCGGAACGACTCGGCGGTAC
CGGCGTCAGGCCCCCTCGCCTAGACTGCACCATTATGTTGGGAGGTGCGTCGACTGGG
GGCCGGCGACTTGAGGTCGGCCGTTTCGTGAAGCCCATTAGTCCCATGGAGACGTTCTC
TCCCATCCATTGGCCTCCGGGGCTCTTCATCAATCGCGTCGGAGTATCGTCTAATGTGA
AATTTATTCATCGTGAGGTATACACCGCCCCCGCGTGGGTGCGGCTCGAAGTCCGGCCC
CCCAGGCTGCCTGCTAACACGGAAGTATGCCTCGTTTAGCTCAGAAGGGACGAGACAC
TCTCGACGGCTCTCAACAAGCCTAGTCCCCACGGTACATACTACCGTGCCAGGTGGGA
TTGGTACGCCGGGGTCCCGTCACAGAACGGCTAGAAGGTAAATACGCCCTGGCCATAT
ACTCCTTGTCCGCATCCCTTGGGGAAATCCATTACGCCGTCGTTGACGTCATCAATGCC
GCGCGATTAACACCGAGCTGATGGGACCAAAACATACCATGTAATCCGTGTACTTACC
CATCGTTGCGGTACAGGATACGGGGCTGTGAGTAATGTACACACTCCTGGAGGGAGCT
TGTCCTCCAGACTTGAATGGCTACCTGCCGGGCTGGGACCTAGCAAACCACCCTGACG
CATCCCAGGCCTTTGATCGGATCACAATTTTGCTTTCACCAGTTCCCAATTACGGTTCC
GCATCGTCGGGAGGCCTTCGACTAGATCTACTCGCATGGTTCGAGCAGTAATATCGGG
TCTGAAATCCCCTGACCCAACGGGGGGCAGTATGTGTGGTCGGAATCTGCACCTTGCG
AGAGATAATCGTAATGACGATGGACCTTCTACAAGTGAGTGCTGGGGCACGAGCGGCG
GACCAGATTGTCTTTCGAATGTTCCCCCCCGTGCCGAAGGCTGAACCCGAGGCGGAGC

CGGGGGCGTGCACCGTTGCCACGCAGGTGCACCGCGAGGCACCTTGAATGTCCCGTCA
TGAAG

>MIN_MZ-304-07.1

GTCGGGATGCCTTCCCAACTCCGGCACGGCGCGGCGCCTTCGCGCACGGATCGGATAA
GCTTACCCGTGGATGGCTCGTTCTCAGAAATATCTCGTAGTCTTCTCGTTGGTTACTCCA
ATGAAGCGCCTCGTCTGCGGGGGCACTGCACCGCGAGAGCTATTCAAGTGCTCTACTA
CTCGACCGGGGGCAGCTTACAACCGCGCGATTGGCACGGTCTACCTCCGTCCAACAGG
GGGTATGACGACCCGGTCCCCCCTCCCGCGTCGAAGGCTGCCAACACGATAGTAAGCG
AACTGGCCCAAACGTATTAATACGTACAGGGGGCGGGCCTCTGGCGCGCCACTGGATC
AGGCACGTGGCGTGCCCGCCTCGTCAGCGCCACCCATTGCTAAGCGCTGACAGTAATA
GACCCCTCCATAGTAGTTGCCGATGTTGATTTGGTCACCGGCCGAAACCTATGCGCTCA
GCACAGGGCAGGTACTACGGAGCGAAAGGTGGATGATTGGCAGGGGCCGCTGGCGCA
CCTACAAAACTATTCGTCCGCGCCTGCTGGAGCGACCAACTACACTCTATAGCGTCCAG
TACCCGAGCAGTCTCTCAACTGGTCCGATTAGACTGTATACACCGCCGTTGGGACGCG
GACTAAACAATTCCCTCATACCCATCCGCCCGTCCGGAGCAGAACGACTCGACGGTAC
CGGCGTCAGGCCCCCTCACACAGGCTGAACCATTTTGTTGGGAGGTGCGTCGACTGGG
GGCCGGCGACTTGAGCTTGGTCGGCCCGTGAAACCCATCAGTCCCATGGAGACGTTCT
CTCCCATCCATTGGCCTCCGGGGCTCTTCATCAATCGCGTCGGAGTATCGTCTAATGTG
AAATTTATTCATCGTGAGGTATACACCGCCCCCGCGTGGGTGCGGCTCGAAGTCCGGC
CCCCCAGGCTGCCTGCTAACACGAAAGTATGCCTCGTTTAGCTCAGAAGGGACGAGAC
ACTCTCGACGGCTCTCAACAAGCCTAGTCCCCACGGTACATACTACCGTGCCAGGTGG
GATTGATACGCCGGGGTCCCGTCGCAGGATGTCTAGGGGGGAGATACGTTCCGGTCAT
ATACTCTTTGTCCGCATCCCTTGGGAAAATCCATTACGCTGTCATTGACGTCGTCAACG
TCATGCCACTAACACCAAACTAGGGGGACCAAACTATACCATGTTATCCGTATGCCTA
CTCGTTGTCGCGGTTCAGAGTACGGGCCTGTGAGTAATGTACGCGCTCCTGGTGGGAA
CTTGTCCTCCGGACTTGAATGGTTACTCGCCGAGCTGGAACTTAGCAAACCACCCTGAT
GCATTCCAGGTATCAGATTAGAACACAATTTTGCTTTCACCAGTTCCCAATTGCGGGGC
CGTATCGTCGGGAGGCCTTCGACTAGATCTACTCGCATGGTTCGAGCGGTAATATCGG
GTCTGAAATCCCCTGACCCAACGGGAGGCGGTACGTGTGATCGGAATCTGCACCTTGC
GAGAGCTAATCGTAATGACGATGGACCTTCTACAAGTGAGTGCTGGGGCGTGAACGGG
GGACCAGATTGTCTTCCGAGCGTTCCCCCCCGTGCCGAACGATGAATTCGAGGCGGAG
GAATGGGCGCGCACCGTTGCCACGCAGGTGCACCGCGCAGCACCTTGAGGGTCCAGTC
ATGAAG

>MIN_MZ-304-07.2

GTCGGGATGCCTTCCCAACTCCGGCACGGCGCGGCGCCTTCGCGCACGGATCGGATAA
GCCTTCCCGTGGACGGCTTGTCTTCAAAAATACCTCGTAGCCTTCTCGTGGTTTACCCC
AATGCCGCGCCGCGTCTGCTTAGATGCTGAATTGCGGAAGCTATTCGAGAGTCCTGTTG
CCCGGCCGGGGGCAGCACACAACCATGGGATCGGCGCAGCCCACCTCCGGCCAACGA
GAGTTACGGCGTCCCGGCCCGTTTTCTCGCGTCGGAGGCTGCCACCACGATAGTAACTG
AACTGGCCCAAACGTATTAATACGCACAGGGGGCGGGCCTCTGGCGCGCCACTGGATC
AGGCCCGTGGCGTGCCCGCCTCGTCAGCGCCACCCATTGCTAAGCGCTGACAGTAATA
GACCCCTCCATAGTAGTTGCCGATGTTGATTCAGACACCGGCCGAAAAGTATGCACTC
AGCACAGGGCAGGTACCACGGAGCGAAAGGTGGATGATTGGCAGGGGCCGCTTGCGC
ACCTACAAAACTATTCGTCCGCGCCTGCTGGAGCGACCAACTACGCTCTATAGCGTTCA
GTACCCGAGCAGTCTCCTAACTGGTCCGGTGAGACTGTAAACATCCTTGTTGGGTTGTT
ACATAAACAACCCACTCTTACCCATCCGTCCGACCGGAGCGGAACGACTCCGCGGTAC
CGGCGTCAGGCCCCCTCGACTAGACTGCGCCGTTATGTTGGGAGGTGCGTCGACTGGG
GGCCGGCGACCTGAGGTCGGCCGTTTCGTGAAGCCCATTAGTCCCATGGACACGTTCTC

TCCCATCCATTGGCCTCCTGGACTTTCCATCAATCATGTCGAAATATTGTCTAATGTGA
AATTTATTCATCGTGAGGTGTAAACCGCCCCCGCGCGGGTGCGGCTCGTAATCCGGCCC
CCCAGGCTGCCTGCTAACACGGAAGTGTACCCGGTTCCACTCAGAGGGCATGAGGCAG
TACCTACGGATCTCAACAAGTCTAGCCCCCATGATACGTACAGCTGGGCCAGGTGGGA
TTAATACGTCGGGGTCCCATCGCGGGATGTTTGAGGGGGAGATCCGTTCCGGCCATAT
ACTCTCTGTCCGCATCCCTTGGGGAAAGCCATTACGCTGCCGTTGACGTTGTCAATGCC
GCTTCATTAACGTCGAACTAGGGAGACCAAAACATACCATGTAATCCGTGTACTTACC
CATTGTTGCGGTTCAGGGTACGGGGCTATGAGTAATGGGCACACTCCTGGTAGGAACT
TGGTCCCCGGCTTTGTATGGCTACTCGCCGAGCTCGGCCTTAGCAAACCACCCTGACGC
ATTCCAGGCCTCTGATCAGATCACAATTTTGCTTTCACCAGTTCCCAATTACGGTTCCG
CATCGTCGAGGAGCCCTCGACCGGGTCTACTCGCAAAGTTCGAGCGGTAATATTGGAT
CTGAAATCTCCTGACCCAACGGGGGGCAGTATGTGTGGTCGGAATCTGCACCTTGCGA
GAGATAATCGTAATGACGATGGACCTTCTACAAGTGAGTGCTGGGGCACGAGCGGCTG
ACCAGATTGTCTTCCGAATGTTCCCCCCCGTGCCGAAGGCTGACCCCGAGGCGGAGCC
GTGGGCGTGCACCGTTGCCACGCAGGTGCACCGCGAGGCACCTTCAAGGTCCCGTCAT
GAAG

>MIN_MZ-307-00.1

GTCGGGATGCCTTCCCAACTCCGGCACGGCGCGGCGCCTTCGCGCACGGATCGGATAA
GCCTTCCCGTGGACGGCTTGTCTTCAAAAATACCTCGTAGCCTTCTCGTGGTTTACCCC
AATGCCGCGCCGCGTCTGCTTAGATGCTGAATTGCGGAAGCTATTCGAGAGTCCTGTTG
CCCGGCCGGGGGCAGCACACAACCATGGGATCGGCGCAGCCCACCTCCGGCCAACGA
GAGTTACGGCGTCCCGGCCCGTTTTCTCGCGTCGGAGGCTGCCCACACGATAGTAACTG
AACTGGCCCAAACGTATTAATACGCACAGGGGGCGGGCCTCTGGCGCGCCACTGGATC
AGGCCCGTGGCGTGCCCGCCTCGTCAGCGCCACCCATTGCTAAGCGCTGACAGTAATA
GACCCCTCCATAGTAGTTGCCGATGTTGATTCAGACACCGGCCGAAAAGTATGCACTC
AGCACAGGGCAGGTACCACGGAGCGAAAGGTGGATGATTGGCAGGGGCCGCTTGCGC
ACCTACAAAACTATTCGTCCGCGCCTGCTGGAGCGACCAACTACGCTCTATAGCGTTCA
GTACCCGAGCAGTCTCCCAACTGGTCCGGTGAGACTGTAAACATCCTTGTTGGGTTGTT
ACATAAACAACCCACTCTTACCCATCCGTCCGACCGGAGCGGAACGACTCCGCGGTAC
CGGCGTCAGGCCCCCTCGACTAGACTGCGCCGTTATGTTGGGAGGTGCGTCGACTGGG
GGCCGGCGACCTGAGGTCGGCCGTTTCGTGAAGCCCATTAGTCCCATGGACACGTTCTC
TCCCATCCATTGGCCTCCTGGACTTTCCATCAATCGTGTCGAAATATTGTCTAATGTGA
AATTTATTCATCGTGAGGTGTAAACCGCCCCCGCGCGGGTGCGGCTCGTAATCCGGCCC
CCCAGGCTGCCTGCTAACACGGAAGTGTACCCGGTTCCACTCAGAGGGCATGAGGCAG
TACCTACGGATCTCAACAAGTCTAGCCCCCATGATACGTACAGCTGGGCCAGGTGGGA
TTAATACGTCGGGGTCCCATCGCGGGATGTTTGAGGGGGAGATCCGTTCCGGCCATAT
ACTCTCTGTCCGCATCCCTTGGGGAAAGCCATTACGCTGCCGTTGACGTCGTCAATGCC
GCTTCATTAACGTCGAACTAGGGAGACCAAAACATACCATGTAATCCGTGTACTTACC
CATTGTTGCGGTTCAGGGTACGGGGCTATGAGTAATGGGCACACTCCTGGTAGGAACT
TGGTCCCCGGCTTTGTATGGCTACTCGCCGAGCTCGGCCTTAGCAAACCACCCTGACGC
ATTCCAGGCCTCTGATCAGATCACAATTTTGCTTTCACCAGTTCCCAATTACGGTTCCG
CCTCGTCGAGGATCCCTCGACTAGATCTACTCGCAAGGTTCGAGCGGTAATATCGGGTC
TGAAATCCCCTGACCCAACGGGAGGCGGTACGTGTGATCGGAATCTGCACCTTGCGAG
AGCTAATCGTAATGACGATGGACCTTCTACAAGTGAGTGCTGGGGCGTGAACGGGGGA
CCAGATTGTCTTCCGAGCGTTCCCCCCCGTGCCGAAGGCTGCACCCGAGGCGGAGCCG
TGGGCGTGCACCGTTGCCACGCAGGTGCACCGCGAGGCACCTTGAAGGTCCCGTCATG
AAG

>MIN_MZ-307-00.2

GTCGGGATGCCTTCCCAACTCCGGCACGGCGCGGCGCCTTCGCGCACGGATCGGATAA
GCTTACCCGTGGATGGCTCGTTCTCAGAAATATCTCGTAGTCTTCTCGTTGGTTACTCCA
ATGAAGCGCCTCGTCTGCGGGGGCACTGAACCGCGAGAGCTATTCAAGTGCTCTACTA
CTCGACCGGGGGCAGCTTACAACCGCGGATCGGCACGGTCTACCTCCGTCCAACAGG
GGGTACGACGACCCGGTCCCCCCTCCCGCGTCGAAGGCTGCCAACACGATAGTAAGCG
AACTGGCCCAAACGTATTAATACGTACAGGGGGCGAGCCTCTGGCGCGCCACTGGATC
AGGCCCGTAGCGTGCCCGCCTCGTCAGCGCCACCCATTGCTAAGCGCTGACAGTAATA
GACCCCTCCATAGTAGTTGCCGATGTTGATTCGGTCACCGGCCGAAACGTATGCACTTA
GCACAGGGCAGGTACTACAAAGCGAGAGGGGGATGATTGGCAGGGGCTGCTGACGCG
CCTATCAGCCCGTTCCCCCGCGCCTGCTGTGGCGACCAACTACGCCCCGCAGCGTCCAG
TACCCGAGCAGTCTCTCAACTGGTCCGATTAGACTGTATACACCGCCGTTGGGACGCG
GACTAAACAATTCCCTCATACCCATCCGCCCGTCCGGAGCAGAACGACTCGACGGTAC
CGGCGTCAGGCCCCCTCACACAGGCTGAACCATTTTGTTGGGAGGTGCGTCGACTGGG
GGCCGGCGACTTGAGCTTGGTCGGCCCGTGAAACCCATCAGTCCCATGGAGACGTTCT
CTCCCATCCATTGGCCTCCGGGGCTCTTCATCAATCGCGTCGGAGTATCGTCTAATGTG
AAATTTATTCATCGTGAGGTATACACCGCCCCCGCGTGGGTGCGGCTCGAAGTCCGGC
CCCCCAGGCTGCCTGCTAACACGAAAGTATGCCTCGTTTAGCTCAGAAGGGACGAGAC
ACTCTCGACGGCTCTCAACAAGCCTAGTCCCCACGGTACATACTACCGTGCCAGGTGG
GATTGGTACGCCGGGGTCCCGTCGCAGGATGTCTAGGGGGGAGATACGTTCCGGTCAT
ATACTCTTTGTCCGCATCCCTTGGGAAAATCCATTACGCTGTCATTGACGTCGTCAACG
TCATGCCACTAACACCAAACTAGGGGGACCAAACTATACCATGTTATCCGTATGCCTA
CTCGTTGTCGCGGTTCAGAGTACTGGCCTGTGAGTAATGTACGCGCTCCTGGTGGGAAC
GTGTCCTCCGGACTTGAATGGTTACTCGCCGAGCTGGAACTTAGCGAACCACCCTGATG
CATTCCAGGTATCAGATTAGAACACAATTTTGCTTTCACCAGTTCCCAATTGCGGGGCC
GTATCGTCGGGAGGCCTTCGACCGGGTCTACTCGCATAGTTCGAGCGGTAATATTGGAT
CTGAAATCTCCTGACCCAACGGGGGGCAGTATGTGTGGTCGGAATCTGCACCTTGCGA
GAGATAATCGTAATGACGATGGACCTTCTACAAGTGAGTGCTGGGGCACGAGCGGCTG
ACCAGATTGTCTTCCGAATGTTCCCCCCCGTGCCGAACGATGAATTCGAGGCGGAGGA
ATGGGCGCGCACCGTTGCCACGCAGGTGCACCGCGCAGCACCTTGAGGGTCCAGTCAT
GAAG

>MIN_MZhu3126.1

TTCGGGATGCCCTTTCGACTGTGCCGCGGCGCGGCGCCTCCGCGCACGGATCAGATAA
GCTTTCCCGTGGACGGCTCGTCCTCAGAAACATTTCGTAGTCTTCTCGTTGGTTACTCCA
CTGCCGCGCCGCGTCTGCTTGGATGCTGAACCACGAGATTTATTAAAGTACTCTACTAC
TCGACCGGGGGCAGCATACAACCGTGGGATCGGTGCGACCCTCCTCCGGCCCGCAGGG
AGTACGACGTCCCGGCCTGTTTTCCTGCGTCAGAGGCTGCCAACACGATAGTAACCGA
ACTGGCCCAAACGTATTAATACGCACAGGGGGCGGACCTCTAAGGCGCCACTGGATCA
GGCCCGTGGCGTGCCCGCCTCGTCAGCGCCACCCATTGCAAAGCGCTGACAGTAATAG
ACCCCTCCATAGTAGTGGCCGATGTTGATTTGGTCACCGGCCGAAACGTATGCGCTCAG
CACAGGGCAGGTACTACGGAGCGAAAGGTGGATGATTGGCAGGGGCCGCTGGCGCAC
CTACAAAACTATTCGTCCGCGCCTGCTGGAGCGACCAACTACGCTCTATAGCGTCCAGT
ACCCGAGCAGTCCCTCAATTGGTCCGATGAGACTGTATACGCCGCCGTTGGGACGCGG
ACTAAACAACCCCCTCATACCCATTCGCCCGTCCAGAGCGGAACGACTCCGCGGTGCC
GGCGTCAGGCCTCCTCGCCTAGGCTGCGCCATTATGTTGGGAGGTGCGTTGACTGGGG
GCCGGCGACCTGAGCTTGGTCGGCCCGTGAAGCCCATCACTCCCATGGAGACGTTTTCT
CCCACCCATTGGCCCCCGGGGTCCTCCACCAATTGCGTCGGAGTCTTGTCTAATATGAA
ATTTATTCATCGTAAGGTATAAACCGCCCCCGCGTGGGTGCGGCTCGAAGTCCGGCTTC
CCAAGCTGGCTGCTAACACGGAAGTGTACCCGGTTCCACTCAGAGGGCCTGAGGCAGT
ACCTACGGATCTCAAAAACCCTAGTCCCCATGATACGTACAGCTGGGCTAGGTGGGAT
TAATACGTCGGGGTCCCATCGCGGGATGTTTGAGGGGGAGATACGTTCCAGCCATATA

CTCCCTGTTCGCGCCCCTCAGAGAAGTCCATTACGCTGCCGTTGACGTCGTCAATGCCG
CGCCACTAACACCGGACTAGGGGGAGAAAACCATACCATGTAATACGTGTACTTGCCC
ATTGTCGCGGTTCAGAGTACGGGGCTGTGAGTAATGTACGCGTTCCTGGTGGGAACTT
GTCCTCTGAACTTGAATAACAACTCACTGGAATGGGATTTAGCAAACCATCCCAACAT
ATTCCAGGCCTCTGATCAGATCACAATTTTGCTTTCACCAGTTCCCCATTATGATTCCGC
ATCGTCGGGAGGCCTTCGACTAGATCTGCTCGCATGGTTCGAGCGGTAATATCGGGTCT
GAAATCCCCTGACCCAACGGGAGGCGGTATGCGTGATCGGAATCTGCACCTTGCGAGA
GCTAATCGTGATGACAATGGCTCTTCCACAAGTGAGTGCGGGGGCGCGAGCGGCGAAC
CAGATGGTCCTCTGAATATTCCCCCCCGTGCCGAAGGCTGAACTCGAGGCGGGGCCGT
GGTCGCGCACCGTTACCACGCAGGTGCGTCGCGAGGCCCCTTGAAGGTCCCGTCATGA
AG

>MIN_MZhu3126.2

GTCGGGATGCCTTCCCAACTCCGGCACGGCGCGGCGCCTTCGCGCACGGATCGGATAA
GCTTACCCGTGGATGGCTCGTTCTCAGAAATATCTCGTAGTCTTCTCGTTGGTTACTCCA
ATGAAGCGCCTCGTCTGCGGGGGCACTGCACCGCGAGAGCTATTCAAGTGCTCTACTA
CTCGACCGGGGGCAGCTTACAACCGCGGGATTGGCACGGTCTACCTCCGTCCAACAGG
GGGTACGACGACCCGGTCCCCCCTCCCGCGTCGAAGGCCGCCAACACGATAGTAAGCG
AACTGGCCCAAACGTATTAATACGTACAGGGGGCGGGCCTCTGGCGCGCCACTGGATC
AGGCACGTGGCGTGCCCGCCTCGTCAGCGCCACCCATTGCTAAGCGCTGACAGTAATA
GACCCCTCCATAGTAGTTGCCGATGTTGATTTGGTCACCGGCCGAAACCTATGCGCTCA
GCACAGGGCAGGTACTACGGAGCGAAAGGTGGATGATTGGCAGGGGCCGCTGGCGCA
CCTACAAAACTATTCGTCCGCGCCTGCTGGAGCGACCAACTACGCTCTATAGCGTCCAG
TACCCGAGCAGTCTCTCAACTGGTCCGATTAGACTGTATACACCGCCGTTGGGACGCG
GACTAAACAATTCCCTCATACCCATCCGCCCGTCCGGAGCAGAACGACTCGACGGTAC
CGGCGTCAGGCCCCCTCACACAGGCTGAACCATTTTGTTGGGAGGTGCGTCGACTGGG
GGCCGGCGACTTGAGCTTGGTCGGCCCGTGAAACCCATCAGTCCCATGGAGACGTTCT
CTCCCATCCATTGGCCTCCGGGGCTCTTCATCAAGCGCGTCGGAGTATCGTCTAATGTG
AAATTTATTCATCGTGAGGTATACACCGCCCCCGCGTGGGTGCGGCTCGAAGTCCGGC
CCCCCAGGCTGCCTGCTAACACGGAAGTATGCCTCGTTTAGCTCAGAAGGGACGAGAC
ACTCTCGACGGCTCTCAACAAGCCTAGTCCCCACGGTACATACTACCGTGCCAGGTGG
GATTGGTACGCCGGGGTCCCGTCGCAGGATGTCTAGGGGGGAGATACGTTCCGGTCAT
ATACTCTTTGTCCGCATCCCTTGGGAAAATCCATTACGCTGTCATTGACGTCGTCAACG
TCATGCCACTAACACCAAACTAGGGGGACCAAACTATACCATGTTATCCGTATGCCTA
CTCGTTGTCGCGGTTCAGAGTACGGGCCTGTGAGTAATGTACGCGCTCCTGGTGGGAA
CTTGTCCTCCGGACTTGATAGGTTACTCGCCGAGCTGGAACTTAGCAAACCACCCTGAT
GCATTCCAGGTATCAGATTAGAACACAATTTTGCTTTCACCAGTTCCCAATTGCGGGGC
CGTATCGTCGGGAGGCCTTCGACCGGGTCTACTCGCATGGTTCGAGCGGTAATATTGG
ATCTGAAATCTCCTGCCCCAACGGGGGGCAGTATGTGTGGTCGGAATCTGCACCTTGC
GAGAGATAATCGTAATGACGATGGACCTTCTACAAGTGAGTGCTGGGGCACGAGCGGC
TGACCAGATTGTCTTCCGAATGTTCACCCCCGTGCCGAAGGCTGAACCCGAGGCGGAG
CCGTGGGCGTGCACCGTTGCCACGCAGGTGCACCGCGAGGCACCTTGAAGGTCCCGTC
ATGAAG

>MIN_MZhu3134.1

GTCGGGATGCCTTCCCAACTCCGGCACGGCGCGGCGCCTTCGCGCACGGATCGGATAA
GCTTACCCGTGGATGGCTCGTTCTCAGAAATATCTCGTAGTCTTCTCGTTGGTTACTCCA
ATGAAGCGCCTCGTCTGCGGGGGCACTGAACCGCGAGAGCTATTCAAGTGCTCTACTA
CTCGACCGGGGGCAGCTTACAACCGCGGGATTGGCACGGTCTACCTCCGTCCAACAGG
GGGTATGACGACCCGGTCCCCCCTCCCGCGTCGAAGGCTGCCAACACGATAGTAAGCG

AACTGGCCCAAACGTATTAATACGTACAGGGGGCGGCCTCTGGCGCGCCACTGGATC
AGGCACGTGGCGTGCCCGCCTCGTCAGCGCCACCCATTGCTAAGCGCTGACAGTAATA
GACCCCTCCATAGTAGTTGCCGATGTTGATTTGGTCACCGGCCGAAACCTATGCGCTCA
GCACAGGGCAGGTACTACGGAGCGAAAGGTGGATGATTGGCAGGGGCCGCTGGCGCA
CCTACAAAACTATTCGTCCGCGCCTGCTGGAGCGACCAACTACGCTCTATAGCGTCCAG
TACCCGAGCAGTCTCTCAACTGGTCCGATTAGACTGTATACACCGCCGTTGGGACGCG
GACTAAACAATTCCCTCATACCCATCCGCCCGTCCGGAGCAGAACGACTCGACGGTAC
CGGCGTCAGGCCCCCTCACACAGGCTGAACCATTTTGTTGGGAGGTGCGTCGACTGGG
GGCCGGCGACTTGAGCTTGGTCGGCCCGTGAAACCCATCAGTCCCATGGAGACGTTCT
CTCCCATCCATTGGCCTCCGGGGCTCTTCATCAATCGCGTCGGAGTATCGTCTAATGTG
AAATTTATTCATCGTGAGGTATACACCGCCCCCGCGTGGGTGCGGCTCGAAGTCCGGC
CCCCCAGGCTGCCTGCTAACACGGAAGTGTGCCTCGTTTAGCTCAGAAGGGACGAGAC
ACTCTCGACGGCTCTCAACAAGCCTAGTCCCCACGGTACATACTACCGTGCCAGGTGG
GATTGGTACGCCGGGGTCCCGTCGCAGGATGTCTAGGGGGGAGATACGTTCCGGTCAT
ATACTCTTTGTCCGCATCCCTTGGGAAAATCCATTACGCTGTCATTGACGGCGTCAACG
TCATGCCACTAACACCAAACTAGGGGGACCAAACTATACCATGTTATCCGTATGCCTA
CTCGTTGTCGCGGTTCAGAGTACGGGCCTGTGAGTAATGTACGCGCTCCTGGTGGAAA
CTTGTCCTCCGGACTTGAATGGTTACTCGCCGAGCTGGAACTTAGCAAACCACCCTGAT
GCATTCCAGGTATCAGATTAGAACACAATTTTGCTTTCACCAGTTCCCAATTGCGGGGC
CGTATCGTCGGGAGGCCTTCGACCGGGTCTACTCGCATAGTTCGAGCGGTAATATTGG
ATCTGAAATCTCCTGACCCAACGGGGGGCAGTATGTGTGGTCGGAATCTGCACCTTGC
GAGAGATAATCGTAATGACGATGGACCTTCTACAAGTGAGTGCTGGGGCACGAGCGGC
TGACCAGATTGTCTTCCGAATGTTCCCCCCCGTGCCGAAGGCTGAACCCGAGGCGGAG
CCGTGGGCGTGCACCGTTGCCACGCAGGTGCACCGCGAGGCACCTTGAAGGTCCCGTC
ATGAAG

>MIN_MZhu3134.2

GTCGGGATGCCCTTTCGACACCAGCACGGCGCGACGCTTTCGCGCACGTATCAGATAA
GTTTTCCCGTGGACGGCTCGTCCTCAGAAATATCTCGTAGTCCTTTCGTTGGTTACTCCA
ATGAAGCGCCTCGCCTGCGGGAGCACTAAACCGCGAGCGCCATTCGAGTGCTCTACAA
CTCGACCGGGGGCAGCACACAACCGTAGGATCGGCGCGGCTCATTTTCGGCTAGTAGG
GGGTACGACGTCCCGGCCCGTTTTCTCGCGTCGGAGGCTGCCAACACGATAGTAACTG
AACTGGCCCAAACGTATTGATACCCCCAGAGGGCGAGCCTCTGGCGCGCCACTGGATC
AGGCCCGTGGCGTGCCCGCCTCGTCAGCGCCACCCATTGCTAAGCGCTGACCGTAATA
GACCCCTCCATAGTAGTTGCCGATGTTGATTTGGTCACCGGCCGAAACGTATGCGCTCA
GCACAGGGCAGGTACTACGGAGCGAAAGGTGGATGATTGGCAGGGGCCGCTGGCGCA
CCTACAAAACTATTCGTCCGCGCCTGCTGGAGCGACCAACTACGCTCTATAGCGTCCAG
TACCCGAGCAGTCCCTCAATTGGTCCGATGAGACTGTATACGCCGCCGTTGGGACGCG
GACTCAACAACCCCCTCATACCCATTCGCCCGTCCAGAGCGGAACGACTCCGCGGTGC
CGGCGTCAGGCCTCCTCGCCTAGGCTGCGCCATTATGTTGGGAGGTGCGTTGACTGGG
GGCCGGCGACCTGAGCTTGGTCGGCCCGTGAAGCCCATCACTCCCATGGAGACGTTTT
CTCCCACCCATTGGCCCCCGGGGTCCTCCACCAATTGCGTCGGAGTCTTGTCTAATATG
AAATTTATTCATCGTAAGGTATAAACCGCCCCCGCGTGGGTGCGGCTCGAAGTCCGGC
TTCCCAAGCTGGCTGCTAACACGGCAGTGTACCCGGTTCCACTCAGAGGGCCTGAGGC
AGTACCTACGGATCTCAAAATCCCTAGTCCCCATGATACGTACAGCTGGGCTAGGTGG
GATTAATACGTCGGGGTCCCATCGCGGGATGTTTGAGGGGGAGATACGTTCCAGCCAT
ATACTCCCTGTTCGCGCCCCTCAGAGAAGTCCATTACGCTGCCGTTGACGTCGTCAATG
CCGCGCCACTAACACCGGACTAGGGGGACCAAACCATACCATGTAATACGTGTACTTG
CCCATTGTCGCGGTTCAGAGTACGGGGCTGTGAGTAATGTACGCGTTCCTGGTGGGAA
CTTGTCCTCTGAACTTGAATAACAGCTCACTGGAATGGGATTTAGCAAACCATCCCAAC
ATATTCCAGGCCTCTGATCAGATCACAATTTTGCTTTCACCAGTTCCCAATTATGATTCC

GCATCGTCGGGAGGCCTTCGACTAGATCTGCTCGCATGGTTCGAGCGGTAATATCGGG
TCTGAAATCCCCTGACCCAACGGGAGGCGGTATGCGTGATCGGAATCTGCACCTTGCG
AGAGCTAATCGTGATGACAATGGCTCTTCCACAAGTGAGTGCGGGGGCGCGAGCGGCG
AACCAGATGGTCCTCTGAATATTCCCCCCCGTGCCGAAGGCTGAACTCGAGGCGGGGC
CGTGGTCGCGCACCGTTACCACGCAGGTGCGTCGCGAGGCCCCTTGAAGGTCCCGTCA
TGAAG

>MIN_MZhu3252.1

GTCGGGATGCCCTTTCGACACCAGCACGGCGCGACGCTTTCGCGCACGTATCAGATAA
GTTTTCCCGTGGACGGCTCGTCCTCAGAAATATCTCGTAGTCCTTTCGTTGGTTACTCCA
ATGAAGCGCCTCGCCTGCGGGAGCACTAAACCGCGAGCGCCATTCGAGTGCTCTACAA
CTCGACCGGGGGCAGCACACAACCGTAGGATCGGCGCGGCTCATTTTCGGCTAGTAGG
GGGTACGACGTCCCGGCCCGTTTTCTCGCGTCGGAGGCTGCCAACACGATAGTAACTG
AACTGGCCCAAACGTATTGATACGCCCAGGGGGCGGGCCTCTGGCGCGCCACTGGATC
AGGCCCGTGGCGTGCCCGCCTCGTCAGCGCCACCCATTGCTAAGCGCTGACAGTAATA
GACCCCTCCATAGTAGTTGCCGATGTTGATTTGGTCACCGGCCGAAACGTATGCGCTCA
GCACAGGGCAGGTACTACGGAGCGAAAGGTGGATGATTGCCAGGGGCCGCTGGCGCA
CCTACAAAACTATTCGTCCGCGCCTGCTGGAGCGACCAACTACGCTCTATAGCGTCCAG
TACCCGAGCAGTCCCTCAATTGGTCCGATGAGACTGTATACGCCGCCGTTGGGACGCG
GACTAAACAACCCCCTCATACCCATTCGCCCGTCCAGAGCGGAACGACTCCGCGGTGC
CGGCGTCGGGCCTCCTCGCCTAGGCTGCGCCATTATGTTGGGAGGTGCGTTGACTGGG
GGCCGGCGACCTGAGCTTGGTCGGCCCGTGAAGCCCATCACTCCCATGGAGACGTTTT
CTCCCACCCATTGGCCCCCGGGGTCCTCCACCAATTGCGTCGGAGTCTTGTCTAATATG
AAATTTATTCATCGTAAGGTATAAACCGCCCCCGCGTGGGTGCGGCTCGAAGTCCGGC
TTCCCAAGCTGGCTGCTAACACGGAAGTGTACCCGGTTCCACTCAGAGGGCCTGAGGC
AGTACCTACGGATCGCAAAATCCCTAGTCCCCATGATACGTACAGCTGGGCTAGGTGG
GATTAATACGTCGGGGTCCCATCGCGGGATGTTTGAGGGGGAGATACGTTCCAGCCAT
ATACTCCCTGTTCGCGCCCCTCAGAGAAGTCCATTACGCTGCCGTTGACGTCGTCAATG
CCGCGCCACTAACACCGGACTAGGGGGACCAAACCATACCATGTAATACGTGTACTTG
CCCATTGTCGCGGTTCAGAGTACGGGGCTGTGAGTAATGTACGCGTTCCTGGTGGGAA
CTTGTCCTCTGAACTTGAATGACAGCTCACTGGAATGGGATTTAAAAAATCATCCCAAC
ATATTCCAGGCCTCTGATCAGATCACAATTTTGCTTTCACCAGTTCCCAATTATGATTCC
GCATCGTCGGGAGGCCTTCGACTAGATCTGCTCGCATGGTTCGAGCGGTAATATCGGG
TCTGAAATCCCCTGACCCAACGGGAGGCGGTATGCGTGATCGGAATCTGCACCTTGCG
AGAGCTAATCGTGATGACAATGGCTCTTCCACAAGTGAGTGCGGGGGCGCGAGCGGCG
AACCAGATGGTCCTCTGAATATTCCCCCCCGTGCCGAAGGCTGAACTCGAGGCGGGGC
CGTGGTCGCGCACCGTTACCACGCAGGTGCGTCGCGAGGCCCCTTGAAGGTCCCGTCG
TGAAG

>MIN_MZhu3252.2

TTCGGGATGCCCTTTCGACTGTGCCGCGGCGCGGCGCCTCCGCGCACGGATCAGATAA
GCTTTCCCGTGGACGGCTCGTCCTCAGAAACATTTCGTAGTCTTCTCGTTGGTTACTCCA
CTGCCGCGCCGCGTCTGCTTGGATGCTGAACCACGAGATTTATTAGAGTACTCTACTAC
TCGACCGGGGGCAGCATAGAACCGTGGGATCGGTGCGACCCTCCTCCGGCCCGCAGGG
AGTACGACGTCCCGGCCTGTTTTCCTGCGTCAGAGGCTGCCAACACGATAGTAACCGA
ACTGGCCCAAACGTATTAATACGCACAGGGGGCGGACCTCTAAGGCGCCACTGGATCA
GGCCCGTGGCGTGCCCGCCTCGTCAGCGCCACCCATTGCAAAGCGCTGACAGTAATAG
ACCCCTCCATAGTAGTTGCCGATGTTGATTTGGTCACCGGCCGAAACGTATGCGCTCAG
CACAGGGCAGGTACTACGGAGCGAAAGGTGGATGATTGGCAGGGGCCGCTGGCGCAC
CTACAAAACTATTCGTCCGCGCCTGCTGGAGCGACCAACTACGCTCTATAGCGTCCAGT

ACCCGAGCAGTCCCTCAATTGGTCCGATGAGACTGTATACGCCGCCGTTGGGACGCGG
ACTAAACAACCCCCTCATACCCATTCGCCCGTCCGGAGCGGTACGACTCCGCGGTGCC
GGCGTCAGGCCTCCTCGCCTAGGCTGCGCCATTATGTTGGGAGGTGCGTTGACTGGGG
GCCGGCGACCTGAGCTTGGTCGGCCCGTGAAGCCCATCACTCCCATGGAGACGTTTTCT
CCCACCCATTGGCCCCCGGGGTCCTCCACCAATTGCGTCGGAGTCTTGTCTAATATGAA
ATTTATTCATCGTAAGGTATAAACCGCCCCCGCGTGGGTGCGGCTCGAAGTCCGGCTTC
CCAAGCTGGCTGCTAACACGGAAGTGTACCCGGTTCCACTCAGAGGGCCTGAGGCAGT
ACCTACGGATCTCAAAAACCCTAGTCCCCATGATACGTACAGCTGGGCTAGGTGGGAT
TAATACGTCGGGGTCCCATCGCGGGATGTTTGAGGGGGAGATACGTTCCAGCCATATA
CTCCCTGTTCGCGCCCCTCAGAGAAGTCCATTACGCTGCCGTTGACGTTGTCAATGCCG
CGCCACTAACACCGAACTAGGGGGAGAAAACCATACCAGGTAATACGTGTACTTGCCC
ATTGTCGCGGTTCAGAGTACTGGGCTGTGAGTAATGTACGCGTTCCTGGTGGGAACTTG
TCCTCTGAACTTGAATAACAGCTCACTGGAATGGGATTTAGCAAACCATCCCAACATAT
TCCAGGCCTCTGATCAGATCACAATTTTGCTTTCACCAGTTCCCAATTATGATTCCGCCT
CGTCGGGAGTCCTTCGACTAGATCTGCTCGCATGGTTCGAGCGGTAATATCGGGTCTGA
AATCCCCTGACCCAACGGGAGGCGGTATGCGTGATCGGAATCTGCACCTTGCGAGAGC
TAATCGTGATGACAATGGCTCTTCCACAAGTGAGTGCGGGGGCGCGAGCGGCGAACCA
GATGGTCCTCTGAATATTCCCCCCCGTGCCGAAGGCTGAACTCGAGGCGGGGCCGTGG
TCGCGCACCGTTACCACGCAGGTGCGTCGCGAGGCCCCTTGAAGGTCCCGTCATGAAG

>MIN_MZhu3312.1

GTCGGGATGCCCTTTCGACACCGGCACGGCGCGGTGCTTTCGCGCACGGATGGGATAA
GCTTTCCCGTGGGTGGCTCGTCCCCGGAGATATCTCGTAGTCTTCTCGTTGGTTACTCCA
ATGAAGTTCCTCGTCTGCGGGGATGCTGAACCGCGAGATTTCTTAGAGTGCTCTACTAC
CTGGCCGGGGGCAGTATACAACCGTGGGATCGGCGCGGCCCACCTCCGGCCAACGAGG
GGTACAACGTTTCGGCGCGTTTTCCCGCGTCGGAGGCTGCCAACACGATAGTAACTGA
AATGGCCCAAACGTATTAATACGCACAGGGGGTGGGCCTCTGGCATGTCACTGGATCA
GGCCCGTGGCGTGCCCTCCCCCTCAGCGCCACCCATTGCTAAGCGCTGACAGTAATAG
ACCCCTCCATAGTAGTTGCCGATGTTGATTCGGTCACCGGCCGAAACGTATGCACTTAG
CACAGGGCAGGTACTACAAAGCGAGAGGGGGATGATTGGAAGGGGCTGCTGACGCGC
CTATCAGCCCGTTCCCCCGCGCCTGCTGTGGCGACCAACTACGCCCCGCAGCGTCCAGT
ACCCGAGCAGTCTCCCGACTGGTCTGATGAGACTGTATACACCGCCGTTGGGATGCGG
ACTAAACAACCCCCTCATACCCATCCGCTTGTCCGGAGCAGAACGACTTGGCGGTACC
GGCGTCAGGCCCACTCGCCTAGGCTGCACCATTATGTTGGGAGGTGCGTCAACTGGGG
GCCGGCAACCTGAGGTCGGCCGTTTCGTGAAGCCCATTAGTCCCATGGAGACGTTCTCT
CCCATCCATTGGCCTCCGGGGCTCTTCATCAATCGCGTCGGAGTATCGTCTAATGTGAA
ATTTATTCATCGTGAGGTATACACCGCCCCCGCGTGGGTGCGGCTCGAAGCCCGGCTTC
CCAGGCTGGCAGCTAACACGGAAGTGTGCCTCGTTCCACTCAGAGGGCATGAGGCAGT
ACCTACGGATCTCAACAAGCCTAGTCCCCATGATACGTTCAGCTGGGCCAGGTGGGAT
TAATACGCCGGAAGCCCGTCGCGGGATGTCTAGGGGGGAGATACGTTCCGGTCATATA
CTCTTTGTCCGCATCCCTTGGGAAAATCCATTACGCTGCCGTTGACGTCGTTAACGTCA
TGCCACTAACACCGAACTAGGGGGACCAAACTATACCATGCTATCCGTATGCCTACTC
GTTGTCGCGGTTCAGGGTACGGGGCTGTGAGGCATGGGCGCGCTCCTGGTGGGAACTT
GTCCTCCGGACTTGAATGGCTACTCGCCGGGCTGGGACTTAGCAAACCACTCTGACGC
ATTCCAGGCCTCTGATCAGATCACAATTTTGCTTTCACCAGGTCCCAATTACGGTGCCG
TATCGTCGGGAGGCCTTCGACTAGATTTACTCCCATGGTTCGAGCGGTAATATCGGGTC
TGAAATCCCCTGACCCAATGGGGGGCGGTACGTGTGATCGGAATCTGCACCTTGCGAG
ATCTAATCGTAATGACGATGGCTCTTCCACAAGTGAGTGCTGGGGCGCGAGCGGCGGA
CCAGATGGCCTTCCGAATGTTCCCCCCCGTGCCGAAGGCTTAACTCGAGGCGGAGCCG
TGGGCTCGCACCGTTGCCGCGCAGGTACACCGCGAGGTCCCTTGAAGGTCCCATCATG
AAG

>MIN_MZhu3312.2

GTCGGGATGCCTTCCCAACTCCGGCACGGCGCGGCGCCTTCGCGCACGGATCGGATAA
GCTTACCCGTGGATGGCTCGTTCTCAGAAATATCTCGTAGTCTTCTCGTTGGTTACTCCA
ATGAAGCGCCTCGTCTGCGGGGGCACTGAACCGCGAGAGCTATTCAAGTGCTCTACTA
CTCGACCGGGGGCAGCTTACAACCGCGGGATTGGCACGGTCTACCTCCGTCCAACAGG
GGGTACGACGACCCGGTCCCCCCTCCCGCGTCGAAGGCTGCCAACACGATAGTAAGCG
AACTGGCCCAAACGTATTAATACGTACAGGGGGCGGGCCTCTGGCGCGCCACTGGATC
AGGCCCGTGGCGTGCCCGCCTCGTCAGCGCCACCCATTGCTAAGCGCTGACAGTAATA
GACCCCTCCATAGTAGTTGCCGATGTTAATTCGGTCACCGGCCGAAACGTGCGCGCTCA
GTGCAGGGCAGGTCCTGTAGAACGGAGGGGGGATATTTGGCAAAGGCTGCTGGTGCGC
CTACCAACTTGTTCCTCCGCGCCTGCTGGAGCGACCAACTACGCCCCGCAGCGTCCAGT
ACCCGAGCAGTCTCTCAACTGGTCCGATTAGACTGTATACACCGCCGTTGGGACGCGG
ACTAAACAACTCCCTCATACCCATCCGCCCGTCCGGAGCGGAACGACTTGGCGGAACC
GGCGTCAGACCCCCTCGCCTAGGCTGCACCATTATATTAGGCGGTGCGTCGACTGAGG
GCTAGCGACTCGATCCCGGTCGGCCCGTGAAGCCCATCAGTTCCATGGAGACGTTCGTT
GCCGTCCATTGGCCTTCTGGGCCCTCCGCCAATTACGTCGGAGTATCGCCTAGGGTGAA
ATGCATTCAACGTGAGGTATAAACCGCCCCCGCGTGGGTGCGGCTCGATGTCTGGCCT
CCCAGGCTGCCTGCTAACACGAAAGTGTACCCGGTTCCACTCAGAGGGCATGAGGCAG
TACCTACGGATCTCAACAAGCCTAGTTCGCGCAATACACATAACCATGCCCGGTGGGA
TTAAGACGCCTGGGTCCCGCCGCGAAACGGCTAGAAGGTAAATACGCCCTGGCCATAT
ACTCTTTGTCCGCATCCCTTGGGGAAATCCATTATGCTGCCGTTGACGTCGTCAACGTC
ACGCCACTAACACCGAACTAGGGGGACCAAACTATACCATGTTATCCGTATACTTACC
CACTGTTGCAGGTCAAGGTATGGGGCTGCAAGTAATGTACATACTCCTGGTGGGAACT
TGTCCTCCGGACTTGAATGGCTACCTGCCGGGCTGGGACTTAGCAAACCACCCTGACG
CATCCCAGGCCTTTGATCGGATCACAATTTTGATTCCACCAGTTCCCAATTACGGTTCC
GCATCGTTGGGAGGCCTTTGGCTAGATCTACCTGCATGGTTCGAGCGGTAATATCGGGT
CTGAAATCCCCTGACCCAACGGGAGGCAGTATGTGTGGTCGGAATCTGCACCTTGCGA
GAGCTAATCGTAATGACGATGGCTCTTCCACAAGTGAGTGGTGGGGCGCGAACAGCGG
ATCAGACGGTCTTCCGAATGTTCCCCCCCGTGCCGAAGGCTGAACTCGAGGCGGAGCC
GTGGGCGCGCACCGTTGCCACGCAGGTGCACCGCGAGGCACCTCGAAGGTCCCGTCAT
GAAG

>BMX_BMX0001.1

GTCGGGATGCCCTCTCGATACTGGCACGGCGCGGCGCCTTCGCGCACGGATCAGATAA
GTTTTCTCGTGGACCGCTCAACCTCGGAAATATCCCGAGGTTTTCTCAGTGGTTACTCT
GATGCCGCGCCGCGTCTGCTTGGATGCTGAACCGAGAGAGCCATTCGAGTGCTCTACA
ACTCGACTGGGGACAGCACACAATTGTGGGATCGGCGCGGCCCACCTCCGGCCAACGA
GAGTTACGAGTACCCGGCCCGTTTCCCCGCATCGGAGGCTGCCAACACGATAGTAACT
GAACTGGCCCAAACGTATTAACACGCACAGGGGACGGGCCTCTGGCGCGCCACTGGAT
CAGGCCCGTGGCGTGCCCGCCTCGTCAGCGCCACCCATTGCTAAGCGCTGACAGTAAT
AGACCCCTCCATAGTAGTTGCCGGTGTTAATTCGGTCACCGGCCGAAACGTGCGCGCTC
AGTGCAGGGCAGGTCCTGTAGAACGGAGGATGAATATTTGGCAAAGGCTGCTGGTGCG
CCTACCAACTTGTTCCTCCGCGCCTGCTAGAGCGACCAACTACGCTCTGTAACGTCCAG
TACCCGAGCAGTCTCTCAACTTGGCCGATGAGACTGTATACACCGCCGTTGGTACGCG
GACTAAACAACTCCCTCATACCCATCCGCCCGTCCGGAGCAGAACGGTTTGGTGGTAC
CAGCGTCAGGCCCCTTCGCCTAGGCTGCACCATTATATTGGGAAACGCGACGGCCAGA
GGCCGGCGACCTAATCTCAGCTATTCTTCGGAGCCCATCACTCCCATGGAGACGTTTTC
TCCCACCCATTGGCCCCCGGGGTCCTCCACCAATTGCGTCGGAGTCTTGTCTAATATGA
AATTTATTCATCGTAAGGTATAAACCGCCCCCGCGTGGGTGCGGCTCGAAGTCCGGCTT
CCCAAGCTGGCTGCTAACACGGAAGTGTACCCGGTTCCACTCAGAGGGCCTGAGGCAG

TACCTACGGATCTCAAAAACCCTAGTCCCCATGATACGTACAGCTGGGCTAGGTGGGA
TTAATACGTCGGGGTCCCGTCGCGGGATGTCTAGGGGGGAGATACGTTCCGGCCATCT
ACTCTCTGTCCACATCCCTTGAGGAAATCCATTGCGCTGCCGTTGACGTCGTTAACGCC
GCTTCATTAACGTCGAACTAGGGAGACCAAACTACATCATGTTATCTGTATGCCCAGTT
GTTGTTGCGGTACAGGGTACGGGGCCGTGAGGCACGGGCGCGCTCCTGGTGGAAACTT
GTCCTCCGGACTTTAATGGCTACTCGCCGGGCTGAGACTTAGCAGACCACCCTGACGC
ATTCCAGGCCTCAGATTAGATCGCAATTTTGCTTTCATTAGTTCCCAATTACGGTTCCGC
ATCGTCAGGAGGCCTTCGACTAGATCTACTCGTATGGTTCGAGCGGTGATATCGGGTCT
AAAATCCCCTGACCCAATGGGGGGCGGTACGTGTGATCGGAATCTGCACCTTGCGAGA
TCTAATCGTAATGACGATGGCTCTTCCACAAGTGAGTGCTGGGGCGCGAGCGGCGGAC
CAGATGGCCTTCCGAATGTTCCCCCCCGTGCCGAAGGCTTAACTCGAGGCGGAGCCGT
GGGCTCGCACCGTTGCCGCGCAGGTACACCGCGAGGTCCCTTGAAGGTCCCATCATGA
AG

>BMX_BMX0001.2

GTCGGGACGCCCTCCCGGCTCTGGCACGGCGCGGTGGCTTTGCGCACGGATCGGATAA
ACTTTCCATTGGACGGCTCGTTCTCAGAAATATCTCGTAGTCTTCTCGTTGGTTACTCCA
ATGAAACGCCTCGTCTGCGGGGGCACTGAACCGCGAGAGCTATTCAAATGCTCTACTA
CTCGACCGGGGGCAGCTTACAACCGTGGGATCGGCGCGGCCCACCTCCGGTCAACGAG
AGTTACGAGTTCCCGGCCCGTTTTCTCGCGTCGGAGGCTGCCAACACGATAGTAACTGA
ACTGGCCCAAACGTATTAATACGCCCCGGGGGCGGGCCTCTGGCGCGCCACTGGATCA
GGCCCGTGGCGTGCCCGCCTCGTCAGCGCCACCCATTGTTAAGCGCTGACAGTAATAG
ACCCCTCCTTAGTAGTTGCCGATGTTGATTTGGTCACCGGCCGAAACGTATGCACTCAG
CACAGGGCAGGTACCACGGAGCGAAAGGTGGATGATTGGCAGGGGCCGCTTGCGCAC
CTACATAACTATTCGTCCGCGCCTGCTGGAGCGACCAACTACGCTCTATAGCGTTCAGT
ACCCGAGCAGTCTCCTAACCGGTCCGGTGAGACTGTAAACATCCTTGTTGGGTCGTTAC
ATAAACAACCCACTCTTACCCATCCGCCCGTCCGGAGCGGAACGACTTGGCGGAACCG
GCGTCAGACCCCCTTGCCTAGATTGCACTAATATGTTGGGAGATGCGTCGACTGGGATC
TGACAACCTGAGCTTGGTCGGCCCGTGAAGCCCATCAGTCTCATGGAGACGTTCTCTCC
CATCCATTGGCCTCCTAGGCTTTCCACCAATTGCGTCAGGGTAATGCCAGATGTTAGAT
GTATTCATCGTGAGGTATACACCGCCCCCGCGTGGGTGCGGCTCGAAGCCCGGCCTCC
CAGGCTGGCTGCTAACACGGAAGTGTACCCGGTTCCACTCAGAGGGCATGAGGCAGTA
CCTACGGATCTCAACAAGCCTAGTTCGCGCGATACACATAACCATGCCCGGTGGGATT
AAGACGCCGGGGTCCCGTCGCGGGACATCTGGGATGGGAACACGTTCCGGTCATATAC
TCTTTGTCCGCATCCCTTGGGGAAATCCATTACGCTGTCATTGACGTCGTCAACGTCAT
GCCACTAACACCGAACTAGGGGGACCAAACTATACCATGTTATCCGTATGCCTACTCG
TTGTCGCGGTTCAGAGTACGGGCCTGTGAGTAATGTACGCGCTCCTGGTGGAACTTGT
CCTCCGGACTTGAATGGTTACTCGCCGAGCTGGAACTTAGCAAACCACCCCAACATATT
CCAGGCCTCTGATCAGATCACAATTTTGCTTTCACCAGTTCCCAATTACGGTTCCGCAT
CGTCGGGAGGCCTTCGACTAAATCTACTCGCAAGGTTCGAGCGGTAATATCGGGTCTG
AAATCCCCTGCCCCAACGGGAGGCGGTACGTGTGATCGGAAGCTGCACCTCGCGAGAG
CCAAGCGGAATGACGATGGCTCTTCCACAAGTGAGTGGTGTAGTGCCAGCGGCGGACC
AGATGGTCTTCCGAATGTTCCCCCCCGTGCCGAAGGCTGAATTCGAGATGGAGCAGTG
GGCTCACACCGTTGCCACGCAGGTGCACCGCGAGGCAACTTGAAGGTCCAGTCATGAA
G

>BMX_BMX0005.1

TTCAAGATGCCCTCCTGACTCTGCCACGACGCGGTGGCTTCACGCATGGACCGGATAA
GTTTTCCCGTGGACCGCTCAACCCCGGAAATACCCCGAGGTTTTCTCAGTGGTTACCCT
GATGCCGCGCAGCGTCTGCTTGGATGCTGAACCGAGAGAGCCATTCGAGTGCTCTACA

ACTCGACTGGGGACAGCACACAATTGTGGGATCGGCGCGGCCCACCTCCGGCCAACGA
GAGTTACGAGTACCCGGCCCGTTTCCCCGCATCGGAGGCTGCCAACACGATAGTAACT
GAACTGGCCCAAACGTATTAACACGCACAGGGGACGGGCCTCTGGCGCGCCACTGGAT
CAGGCCCGTGGCGTGCCCGCCTCGTCAGCGCCACCCATTGCTAAGCGCTGACAGTAAT
AGACCCCTCCATAGTAGTTGCCGATGTTGATTTGGTCACCGGCCGAAACGTATGCGCTC
AGCACAGGGCAGGTACTACGGAGCGAAAGGTGGATGATTGGCAGGGGCCGCTGGCGC
ACCTACAAAACTATTCGTCCGCGCCTGCTGGAGCGACCAACTACGCTCTATAGCGTCCA
GTACCCGAGCAGTCCCTCAATTGGTCCGATGAGACTGTATACGCCGCCGTTGGGACGC
GGACTAAACAACCCCCTCATACCCATTCGCCCGTCCGGCGCGGAACGACTCCGCGGTG
CCGGCGTCAGGCCTCCTCGCCTAGGCTGCGCCATTATGTGGGGAGGTGCGTTGACTGG
GGGCCGGCGACCTGAGCTTGGTCGGCCCGTGAAGCCCATCACTCCCATGGAGACGTTT
TCTCCCACCCATTGGCCCCCGGGGTCCTCCACCAATTGCGTCGGAGTCTTGTCTAATAT
GAAATTTATTCATCGTAAGGTATACACCGCCCCCGCGTGGGTGCGGCTCGAAGTCCGG
CTTCCCAAGCTGGCTGCTAACACGGAAGTGTACCCGGTTCCGCTCAGAGGGGACGAGA
CACTCTCGATGGCTCTCAACAAGCCTAGTCTCCACGATACATACAATCGTGCCAGGTGG
GATTAATACGCCGGAAGCCCATCGCGAGATGTTTGAGGGAGAGATACGTTCCGGCCAT
ATACTCCCTGTCCGCATCCCTTGGGGAAATCCGTTACACTGCCGTTGACGTCGTTAACG
CCACGCCACTAACACCGAACTAGGAGGACCAAACTATACCATGTTATCCGTATGCCCA
GTTGTTGTTGCGGTTCAGGGTACGGGGCTGTGAGGCACGTACGCGTTCCTGGTGGGAA
CTTGTCCTCTGAACTTGATAAACAGCTCACTGGAATGGGATTTAGCAAACCATCCCAAC
ATATTCCAGGCCTCTGATCAGATCACAATTTTGCTTTCACCAGTTCCCAATTATGATTCC
GCATCGTCGGGAGGCCTTCGACTAGATCTACTCGCATGGTTTGAGCGATAATATCAAGT
CTGGAATCCTCTGACCCAACGTGAAGCGGTATGCGCAATTGGGGTCTGTTCCTTGCAAG
AGCTAATCGTAATGACGATGGACCTTCTACAAGTGAGTGCTGTAGCGCCAGCGGCTGA
CCAGATTGTCTTCCGAATGTTCCCCCCCGTGCCGAAGGCTGAACCCGAGGCGGAGCCG
TGGGCGTGCACCGTTGCCACGCAGGTGCACCGCGAGGCACCTTGAAGGTCCCGTCATG
AAG

>BMX_BMX0005.2

GTCGGGATGCCTTCCCAACTCCGGCACGGCGCGGCGCCTTCGCGCACGGATCGGATAA
GCTTACCCGTGGACCGCTCAACCCCGGAAATTCCCCGAGGTTTTCTCAGTGGTTACCCT
GATGCCGCGCAGCGTCTGCTTGGATGCTGAACCGCGAGAGCTATTCAGGTGCTCGACT
ACTCGACCGGGAGCAGCACGCAGCCGCGGGAACGACGCGGTCTACCTCCGGCCAGCA
GGGGGTACGACGTCCCGGCCCGTTTTCCCGCGTCGGAGGCTGCCAACACGATAGTAAC
TGAAATGGCCCAAACGTATTAATACGCACAGGGGGCGGGCCTCTGGCGCGCCACTGGA
TCAGGCCCGTGGCGTGCCCGCGTCGTCAGCGCCACCCATTGCTAAGCGCTGACAGTAA
TAGACCCCTCCATAGTATTTGCCGATGTTGATTCGGTCACCGGCCGAAACGTATGCACT
CAGCACAGGGCAGGTACCACGGAGCGAAAGGTGGATGATTGGCAGGGGCCGCTTGCG
CACCTACATAACTATTCGTCCGCGCCTGCTGGAGCGACCAACTACGCTCTATAGCGTTC
AGTACCCGAGCAGTCTCCTAACTTGGCCGATTCTATTGTATAAACAGTCGTTGGGACGC
GGACTAAACAACCTCCTCATACCCATCCTCCCGTTCGCAGCAGAACGACTCGACGGTA
CCGGCGTCAAGCGCCTCCGCCTAGGCTGCATCATTATGTTGGGAGATGCGTCGACTGG
GGGCCGGCGACCTGAGCTCGGTCGTCCCGTGAAGCCAATCAGTCTCATGGAGCCGTTC
TCTCCCATACATTGGCCTCCTGGGCTTTCCATCAATTGTGTCGAAATATTGTCTAATGTG
AAATTTATTCATCGTGAGGTGTAAACCGCCCCCGCGTGACTGTAGAGCGTAATCCGGC
CTTCTGGGCTAGCAGCTAACGAGGAAGTGTGCTTCATTTCGTTCGGAAAAGACGAAAT
GCTCTAGGCGGCTCTCAACCAGCCTAGTCCCCACGATATATACAACCGTACCAGGTCG
AATTAATCCGCCGGGGTCCCGTCGTGGGATGTTTGAGGGGGAGATACGTTCCGGCCAT
ATACTTTTTGTCCGCATCCCTTGGGGAAATCCATTACGCTGCCGTTGATGTCGTTAACG
TCACGCCACTAACACCGAACTAGGGGGACCAAACTATACCATGTTATCCGTATACTTA
CCCACTGCCGCGGTTCAGAGTACGGGCCTGTGAGTAATGTACGCGCTCCTGGTGGGAA

CTTGTCCTCCGGACTTGAATGGTTACTCGCCGAGCTGGAACTTAGCAAACCACCCTGAT
GCATTCCAGGTATCAGATTAAAACACAATTTTGCTTTCACCAGTTCCCAATTGCGGGGC
CGTATCGTCGGGAGGCCTTCGACTAGATCTGCTCGCATGGTTCGAGCGGTAATATCGG
GTCTGAAATCCCCTGACCCAACGGGAGGCGGTATGCGTGATCGGAATCTGCACCTTGC
GAGCGCTAATCGTGATGACGATGGCTCTTCCACAAGTGAGTGCTGGGGCGCGAGCGGC
GAACCAGATGGTCCTCTGAATGTTCCCCCCCGTGCCGAAGGCTGAACTCGAGGCGGGG
CCGTGGTCGCGCACCGTTACCACGCAGGTGCGTCGCGAGTCAACTTGAAGGTCCAGTC
ATGAAG

>BMX_BMX0012.1

TTCAAGATGCCCTCCTGACTCTGCCACGACGCGGTGGCTTCACGCATGGACCGGATAA
GTTTTCCCGTGGACCGCTCAACCCCGGAAATACCCCGAGGTTTTCTCAGTGGTTACCTT
GATGCCGCGCAGCGTCTGCTTGGATGCTGAACCGCGAGAGCTATTCAGGTGCTCGACT
ACTCGACCGGGAGCAGCACGCAGCCGCGGGAACGACGCGGTCTACCTCCGGCCAGCA
GGGGGTACGACGTCCCGGCCCGTTTTCCCGCGTCGGAGGCTGCCAACACGATAGTAAC
TGAAATGGCCCAAACGTATTAATACGCACAGGGGGCGGGCCTCTGGCGCGCCATTGGA
TCAGGCCCGTGGCGTGCCCGCGTCGTCAGCGCCACCCATTGCTAAGCGCTGACAGTAA
TAGACCCCTCCATAGTATTTGCCGATGTTGATTCGGTCACCGGCCGAAAAGTATGCACT
CAGCACAGGGCAGGTACCACGGAGCGAAAGGTGGATGATTTCCAGGGGCCGCTTGCG
CACCTACATAACTATTCGTCCGCGCCTGCTGGAGCGACCAACCACGCTCTATAGCGTTC
AGTACCCGAGCAGTCTCCTAACTTGGCCGATTCTATTGTATAAACAGTCGTTGGGACGC
GGACTAAACAACCTCCTCATACCCATCCTCCCGTTCGCAGCAGAACGACTCGACGGTA
CCGGCGTCAAGCGCCTCCGCCTAGGCTGCATCATTATGTTGGGAGATGCGTCGACTGG
GGGCCGGCGACCTGAGCTCGGTCGTCCCGTGAAGCCAATCAGTCTCATGGAGCCGTTC
TCTCCCATACATTGGCCTCCTGGGCTTTCCATCAATTGTGTCGAAATATTGTCTAATGTG
AAATTTATTCATCGTGAGGTGTAAACCGCCCCCGCGTGACTGTAGAGCGTAATCCGGC
CTTCTGGGCTAGCAGCTAACGAGGAAGTGTGCTTCATTTCGTTTGGAAAAGACGAAAT
GCTCTAGGCGGCTCTCAACCAGCCTAGTCCCCACGATACATACAACCGTACCAGGTCG
AATTAATCCGCCGGGGTCCCGTCGTGGGATGTTTGAGGGGGAGATACGTTCCGGCCAT
ATACTTTTTGTCCGCATCCCTTGGGGAAATCCATTACGCTGCCGTTGATGTCGTTAACG
TCACGCCACTAACACCGAACTAGGGGGACCAAACTATACCATGTTATCCGTATACTTA
CCCACTGCCGCGGTTCAGAGTACGGGCCTGTGAGTAATGTACGCGCTCCTGGTGGGAA
CTTGTCCTCCGGACTTGAATGGTTACTCGCCGAGCTGGAACTTAGCAAACCACCCTGAT
GCATTCCAGGTATCAGATTAAAACACAATTTTGCTTTCACCAGTTCCCAATTGCGGGGC
CGTATCGTCGGGAGGCCTTCGACTAGATCTGCTCGCATGGTTCGAGCGGTAATATCGG
GTCTGAAATCCCCTGACCCAACGGGAGGCGGTATGCGTGATCGGAATCTGCACCTTGC
GAGAGCTAATCGTGATGACGATGGCTCTTCCACAAGTGAGTGCTGGGGCGCGAGCGGC
GAACCAGATGGTCCTCTGAATGTTCCCCCCCGTGCCGAAGGCTGAACTCGAGGCGGGG
CCGTGGTCGCGCACCGTTACCACGCAGGTGCGTCGCGAGTCAACTTGAAGGTCCAGTC
ATGAAG

>BMX_BMX0012.2

GTTGGGATCTCTTCTCGACTCTGGCACGGCGCGGTGGCTTCGCGCACGGATCGGATAA
GCTTTCCCGTGCATCGATTGTCCTCAGAAGCATTTTGAGGTTTTCTCAGTGGCCGCCTTG
ATGCCGCGCCGCGTCTGCTTGGATGCTGAACCGCGAGATTTATTAGAGTGCTCTACTAC
TCGACCGGGGGCAGCATACAACCGTGGGATCGGCGCGGCCCACCTCCGGCCAACGAG
AGTTACGAGTACCCGGCCCGTTTTCCCGCGTCGGAGGCTGCCAACACAATAGTAACTG
AACTGGCCCAAACGTATTAATACGCCCCGGGGGCGGGCCTCTGGCGCGCCACTGGATC
AGGCCCGTGGCGTGCCCGCCTCGTCAGCGCCACCCATTACTAAGCGCTGACAGTAATA
GACCCCTCCATAGTAGCTGCCGATGCTAATTCGGTCACTGGCCGAAACGTATGCGCTCA

GCACAGGGCAGGTACTACGGAGCGAAAGGTGGATGATTGGCAGGGGCCGCTGGCGCA
CCTACAAAACTATTCGTCCGCGCCTGCTGGAGCGACCAACTACGCTCTATAGCGTCCAG
TACCCGAGCAGTCCCTCAATTGGTCCGATGAGACTGTATACGCCGCCGTTGGGACGCG
GACTACACAACCCCCTCATACCCATTCGCCCGTCCAGAGCGGAACGACTCCGCGGTGC
CGGCGTCAGGCCTCCTCGCCTAGGCTGCGCCATTATGTTGGGAGGTGCGTTGACTGGG
GGCCGGCGACCTGAGCTTGGTCGGCCCGTGAAGCCCATCAGTCCCATGGAGACGTTCT
CTCCCATCTATTGGCCTCCGGGGCTCTCCACCAATCGCACCGGAGTCTTGTCTAATATG
AAATTTATTCATCGTGAGGTATAAACCGCCCCCGAGTGGGTGCGGCTTGAAGTCCAGC
CCCCCAGGCCGCCTGCTAACACGGATGTGTACCCGGTTCCACTCAGAGGGCATGAGGC
AGTACCTACGGATCTCAACAAGCCTAGTCCCCATGATACGTACAGCTGGGCCAGGTGG
GATTAATACGCCGGGGTTCCGTTGCGGAACGGCTAGAAGGTAAATACGCCTTGGCCAT
ATACTCTTTGTCCGCATCCCTTGGGGAAATCCATCATGCTGCCGTTGACGTCGTCAACG
TCACGCCACTAACACCGAACTAGGGGGACCAAACTATACCATGTTATCCGTATACTTA
CCCACTGTTGCAGGTCAAGGTATGGGGCTGCAAGTAATGTACATACTCCTGGTGGGAA
CTTGTCCTCCGGACTTGAATGGCTACCTGCCGGGCTGGGACTTAGCAAACCACCCTGAC
GCATCCCAGGCCTTTGATCGGATCACAATTTTGCTTTCACCAGTTCCCAATTACGGTTC
CGCCTCGTCGGGAGGCCTTCGACTAGATCTACTCGCATGGTTCGAGCAGTAATATCGG
GTCTGAAATCCCCTGACCCAACGGGGGGCAGTATGTGTGGTCGGAATCTGCACCTTGC
GAGAGATAATCGTAATGACGATGGACCTTCTACAAGTGAGTGCTGGGGCACGAGCGGC
TGACCAGATTGTCTTCCGAATGTTCCCCCCCGTGCCGAAGGCTGAACCCGAGGCGGAG
CCGTGGGCGTGCACCGTTGCCACGCAGGTGCACCGCGAGGCACCTTGAAGGTCCCGTC
ATGAAG

>BMX_BMX0013.1

GGCGGGATGCCCTTTCGACACTGGCACGGCACGGCGCCTTCGCGCACGGATCGGATAA
ACTTTCCATTGGACGGCTCGTTCTCAGAAATATCTCGTAGTCTTCTCGTTGGTTACTCCA
ATGAAACGCCTCGTCTGCGGGGGCACTGAACCGCGAGAGCTATTCAAATGCTCTACTA
CTCGACCGGGGGCAGCTTACAACCGTGGGATCGGCGCGGCCCACCTCCGGTCAACGAG
AGTTACGAGTTCCCGGCCCGTTTTCTCGCGTCGGAGGCTGCCAACACGATAGTAACTGA
ACTGGCCCAAACGTATTAATACGCCCCGGGGGCGGGCCTCTGGCGCGCCACTGGATCA
GGCCCGTGGCGTGCCCGCCTCGTCAGCGCCACCCATTGCTAAGCGCTGACAGTAATAA
ACCCCTCCATAGTAGTTGCCGATGTTGATTCGGTCACCGGCCGAAACGCGCGCGCTCA
GTGCAGGGCAGGTACTGTAGAGCGGAGGATGGATGATTTCCAGGGGTTGTTGGCACGC
CTACAAAACTATTTCTCCGCTCCCGCTGGAGCGACCAACTGCGCCTCGCAGCGACTGGT
ATCCGAACAGTCTATCAACTTGGCCGATTCTATTGTATAAACAGTCGTTGGGACGCGGA
CCAAACAACCCCCTCATACCCATCCGCCCGTCCGGAGTGGAACGACTTGGCGGTATTG
GCCTCAGGCCCACTCGCCTAGATTGCATCATTATGTTGGGAGGTACATCGACTGGGGG
CTGGCGACTTGATCCCGGTCGGCCCGTAAAGCCCATCAGTCCCATGGACACGTTCTCTC
CCATCCATTGGTCTCCTGGGCTTTCCACGAATTGCGTCGAAGTAATGCCAGATGTTAAG
CGTATTCATCGTGAGGTATAAACCGCCCCCGCGTGGGTGCGGCTCGAAGTTCGGCCCC
CCAGACTGCCAGCTAACACGGAAGTGTGCCTCGTTTCACTCAGAAGGGACGGGACACT
CTCGACGGCCCTCAACAAGCCTAGTCCCCACGATACATACAACCGTGCCAGGTGGAAT
TAATACGTCGGGGTCCCATCGCGGGATGTTTGAGGGGGAGATACGTTCCGGCCATATA
CTCTTTGTCCGCATCCCTTGGGGAAATCCATTACGCTGCCGTTGACGTCGTTAACGTCA
TGCCACTAACACCGAACTAGGGGGACCAAACTATACCATGCTATCCGTATGCCTACTC
GTTGTCGCGGTTCAGAGTACTGGGCTGTGAGTAATGTACGCGTTCCTGGTGGGAACTTG
TCCTCTGAACTTGAATAACAGCTCACTGGAATGGGATTTAGCGAACCATCCCAACATAT
TCCAGGCCTCTGATCAGATCACAATTTTGCTTTCACCAGTTCCCAATTACGATTCCGCA
TCGTCGGGAGGCCTTCGACTAGATCTGCTCGCATGGTTCGAGCGGTAATATCGGGTCTG
AAATCCCCTGACCCAACGGAGACGGTGTGCTCGACCGGGATCTGCACCTTGCGAGAG
CTAATCGTAATGACGATGGCTCTTCCACAAGTGCGTGCTGGGGCGCGAACGGCGGATC

ATATGGTCTTCCGAATGTTCCCCCTCGTGCCGAAGGCTGAACTCAGGGCGGGGCCGTG
GGCGCGCATCGTTGCCACGCAGGTACACCGTGAGGCACCTTGAAGGTCCCGTCATGAA
C

>BMX_BMX0013.2

GTCGGGACGCCCTCCCGGCTCTGGCACGGCGCGGTGGCTTTGCGCACGGATCGGATAA
ACTTTCCATTGGACGGCTCGTTCTCAGAAATATCTCGTAGTCTTCTCGTTGGTTACTCCA
ATGAAACGCCTCGTCTGCGGGGGCACTGAACCGCGAGAGCTATTCAAATGCTCTACTA
CTCGACCGGGGGCAGCTTACAACCGTGGGATCGGCGCGGCCCACCTCCGGTCAACGAG
AGTTACGAGTTCCCGGCCCGTTTTCTCGCGTCGGAGGCTGCCAACACGATAGTAACTGA
ACTGGCCCAAACGTATTAATACGCCCCGGGGGCGGGCCTCTGGCGCGCCACTGGATCA
GGCCCGTGGCGTGCCCGCCTCGTCAGCGCCACCCATTGCTAAGCGCTGACAGTAATAG
ACCCCTCCATAGTAGTTGCCGATGTTGATTCAGACACCGGCCGAAACGTGCGCGCTCA
GTGCAGGGCAGGTACTGTAGAGCGGAGGATGGATGATTGGCAGGGGTTGTTGGCACGC
CTACAAAACTATTTCTCCGCTCCCGCTGGAGCGACCAACTGCGCCTCGCAGCGACTGGT
ATCCGAACAGTCTATCAACTTGGCCGATTCTATTGTATAAACCGTCGTTGGGACGCGGA
CCAAACAACCCCCTCATACCCATCCGCCCGTCCGGAGTGGAACGACTTGGCGGTATTG
GCCTCAGGCCCACTCGCCTAGATTGCATCATTATGTTGGGAGATGCGTCGACTGGGGG
CCGGCGACCTGAGCTCGGTCGTCCCGTGGAGCCAATCAGTTCCATGGAGACGTTCGCT
GCCGTCCATTGGCCTTCTGGGCCCTTCACCAATTGCGTCAGGGTAATGCCAGATGTGAA
GCGTATTCATCATAGGGCACAAGCCGCGCCTTCGTGGGTACGGCTCGAAGCCCGACCC
CCCAGGCTGCCTAATGACACGGAAGTGTACCTGGTTCCACTCAAAAGGCATGAGGCAG
TCCCTACAGATCTTAGCAAGCTAAGTCCCCACGATACATACAACCGGGCCAGGTGGGA
TTAAGACGCCGGGGTCCCGTCGCGGGACATCTGGGATGGGAACACGTTCCGGTCATAT
ACTCTTTGTCCGCATCCCTTGGGGAAATCCATTACGCTGTCATTGACGTCGTCAACGTC
ATGCCACTAACACCGAACTAGGGGGACCAAACTATACCATGTTATCCGTATGCCTACT
CGTTGTCGCGGTTCAGAGTACGGGCCTGTGAGTAATGTACGCGCTCCTGGTGGGAACTT
GTCCTCCGGACTTGAATGGTTACTCGCCGAGCTGGAACTTAGCAAACCACCCCAACAT
ATTCCAGGCCTCTGATCAGATCACAATTTTGCTTTCACCAGTTCCCAATTACGGTTCCG
CATCGTCGGGAGGCCTTCGACTAAATCCACTCGCAAGGTTCGAGCGGTAATATCGGGT
CTGAAATCCCCTGACCCAACGGGAGGCGGTACGTGTGATCGGAAGCTGCACCTCGCGA
GAGCCAAGCGGAATGACGATGGCTCTTCCACAAGTGAGTGGTGTAGCGCCAGCGGCGG
ACCAGATGGTCTTCCGAATGTTCACCCCCGTGCCGAAGGCTGAACTCAAGGCGGGGCC
GTGGGTGCGCACCGTTACCACGCAGGTGCATCGCGAGGCACCTTGAAGGTCCAGTCAT
GAAG

>BMX_BMX0014.1

GTCGGGACGCCCTCCCGGCTCTGGCACGGCGCGGTGGCTTTGCGCACGGATCGGATAA
ACTTTCCATTGGACGGCTCGTTCTCAGAAATATCTCGTAGTCTTCTCGTTGGTTACTCCA
ATGAAACGCCTCGTCTGCGGGGGCACTGCACCGCGAGAGCTAGTCAAATGCTCTACTA
CTCGACCGGGGGCAGCTTACAACCGTGGGATCGGCGCGGCCCACCTCCGGTCAACGAG
AGTTACGAGTTCCCGGCCCGTTTTCTCGCGTCGGAGGCTGCCAACACGATAGTAACTGA
ACTGGCCCAAACGTATTAATACGCCCCGGGGGCGGGCCTCTGGCGCGCCACTGGATCA
GGCCCGTGGCGTGCCCGCCTCGTCAGCGCCACCCATTGCTAAGCGCTGACCGTAATAG
ACCCCTCCATAGTAGTTGCCGATGTTGATTTGGTCACCGGCCGAAACGTATGCGCTCAG
CACAGGGCAGGTACTACGGAGCGAAAGGTGGATGATTGGCAGGGGCCGCTGGCGCAC
CTACAAAACTATTCGTCCGCGCCTGCTGGAGCGACCAACTACGCTCTATAGCGTCCAGT
ACCCGAGCAGTCCCTCAATTGGTCCGATGAGACTGTATACGCCGCCGTTGGGACGCGG
ACTAAACAACCCCCTCATACCCATTCGCCCGTCCAGAGCGGAACGACTCCGCGGTGCC
GGCGTCAGGCCTCCTCGCCTAGGCTGCGCCATTATGTTGGGAGGTGCGTTGACTGGGG

GCCGGCGACTTGATCCCGGTCGGCCCGTGAAGCCCATCACTCCCATGGAGACGTTTTCT
CCCACCCATTGGCCCCCGGGGTCCTCCACCAATTGCGTCGGAGTCTTGTCTAATATGAA
ATTTATTCATCGTAAGGTATACACCGCCCCCGCGTGGGTGCGGCTCGAAGTCCGGCTTC
CCAAGCTGGCTGCTAACACGGAAGTGTACCCGGTTCCGCTCAGAGGGGACGAGACACT
CTCGATGGCTCTCAACAAGCCTAGTCTCCACGATACATACAATCGTGCCAGGTGGGATT
AATACGCCGGAAGCCCATCGCGAGATGTTTGAGGGGGAGATACGTTCCGGCCATATAC
TCCCTGTCCGCATCCCTTGGGGAAATCCGTTACACTGCCGTTGACGTCGTTAACGCCAC
GCCACTAACACCGAACTAGGAGGACCAAACTATACCATGTTATCCGTATGCCCAGTTG
TTGTTGCGGTTCAGGGTACGGGGCTGTGAGGCACGTACGCGTTCCTGGTGGGAACTTGT
CCTCTGAACTTGATAAACAGCTCACTGGAATGGGATTTAGCAAACCATCCCAACATATT
CCAGGCCTCTGATCAGATCACAATTTTGCTTTCACCAGTTCTCAATTATGATTCCGCATC
GTCGGGAGGCCTTCGACTAGATCTACTCGCATGGTTTGAGTGATAATATCAAGTCGGG
AATCCTCTGACCCAACGTGAAGCGGTATGCGCAATTGGGGTCTGTTCCTTGCAAGAGCT
AATCGTAATGACGATGGACCTTCTACAAGTGAGTGCTGTAGCGCCAGCGGCTGACCAG
ATTGTCTTCCGAATGTTCCCCCCCGTGCCGAAGGCTGAACCCGAGGCGGAGCCGTGGG
CGTGCACCGTTGCCACGCAGGTGCACCGCGAGGCACCTTGAAGGTCCCGTCATGAAG

>BMX_BMX0014.2

GTCGGGATGCCTTCCCAACTCCGGCACGGCGCGGCGCCTTCGCGCACGGATCGGATAA
GCTTACCCGTGGACCGCTCGTTCTCAGAAATATCTCGTAGTCTTCTCGTTGGTTACTCCA
ATGAAACGCCTCGTCTGCGGGGGCACTGAACCGCGAGAGCTATTCAAGTGCTCTACTA
CTCGACCGGAGGCAGCTTACAACCGTGGGATCGGCGCGGCCCACCTCCGGTCAACGAG
AGTTACGAGTACCTGGTCCGTTTTCCCGCGTCGGAAGCTACCAACACGATCGTAACTGA
ACCAGCGCGAGAATATGGATACGCCTCGGGGGCGGGCCTCTGGCGCGCCACTGGATCA
GGCCCGTGGCGTGCCCGCCTCGTCAGCGCCACCCATTGTTAAGCGCTGACAGTAATAG
ACCCCTCCTTAGTAGTTGCCGATGTTGATTTGGTCACCGGCCGAAACGTATGCACTCAG
CACAGGGCAGGTACCACGGAGCGAAAGGTGGATGATTGGCAGGGGCCGCTTGCGCAC
CTACATAACTATTCGTCCGCGCCTGCTGGAGCGACCAACTACGCTCTATAGCGTTCAGC
ACCCGAGCAGTCTCCCAACCGGTCCGGTGAGACTGTAAACATCCTTGTTGGGTCGTTAC
ATAAACAACCCACTCTTACCCATCCGCCCGTCCGGAGCGGAACGACTTGGCGGAACCG
GCGTCAGACCCCCTTGCCTAGATTGCACTAATATGTTGGGAGATGCGTCGACTGGGATC
TGACAACCTGAGCTTGGTCGGCCCGTGAAGCCCATCAGTCTCATGGAGACGTTCTCTCC
CATCCATTGGCCTCCTAGGCTTTCCACCAATTGCGTCAGGGTAATGCCAGATGTTAGAT
GTATTCATCGTGAGGTATACACCGCCCCCGCGTGGGTGCGGCTCGAAGCCCGGCCTCC
CAGGCTGGCTGCTAACACGGAAGTGTACCCGGTTCCACTCAGAGGGCATGAGGCAGTA
CCTACGGATCTCAACAAGCCTAGTTCGCGCGATACACATAACCATGCCCGGTGGGATT
AAGACGCCGGGGTCCCGTCGCGGGACATCTGGGATGGGAACACGTTCCGGTCATATAC
TCTTTGTCCGCATCCCTTGGGGAAATCCATTACGCTGTCATTGACGTTGTCAACGTCAT
GCCACTAACACCGAACTAGGGGGACCAAACTATACCATGTTATCCGTATGCCTACTCG
TTGTCGCGGTTCAGAGTACGGGCCTGTGAGTAATGTACGCGCTCCTGGTGGGAACTTGT
CCTCCGGACTTGAATGGTTACTCGCCGAGCTGGAACTTAGCAAACCACCCCAACATATT
CCAGGCCTCTGATCAGATCACAATTTTGCTTTCACCAGTTCCCAATTACGGTTCCGCAT
CGTCGGGAGGCCTTCGACTAAATCTACTCGCAAGGTTCGAGCGGTAATATCGGGTCTG
AAAACCCCTGACCCAACGGGAGGCGGTACGTGTGATCGGAAGCTGCACCTCGCGAGA
GCCAAGCGGAATGACGATGGCTCTTCCACAAGTGAGTGGTGTAGTGCCAGCGGCGGAC
CAGATGGTCTTCCGAATGTTCCCCCCCGTGCCGAAGGCTGAATTCGAGATGGAGCAGT
GGGCTCACACCGTTGCCACGCAGGTGCACCGCGAGGCAACTTGAAGGTCCAGTCATGA
AG

>BMX_BMX0015.1

TTCAAGATGCCCTCCTGACTCTGCCACGACGCGGTGGCTTCACGCATGGACCGGATAA
GTTTTCCCGTGGACCGCTCAACCCCGGAAATACCCCGAGGTTTTCTCAGTGGTTACCCT
GATGCCGCGCAGCGTCTGCTTGGATGCTGAACCGCGAGAGCTATTCAGGTGCTCGACT
ACTCGACCGGGAGCAGCACGCAGCCGCGGGAACGACGCGGTCTACCTCCGGCCAGCA
GGGGGTACGACGTCCCGGCCCGTTTTCCCGCGTCGGAGGCTGCCAACACGATAGTAAC
TGAAATGGCCCAAACGTATTAATACGCACAGGGGGCGGGCCTCTGGCGCGCCATTGGA
TCAGGCCCGTGGCGTGCCCGCGTCGTCAGCGCCACCCATTGCTAAGCGCTGACAGTAA
TAGACCCCTCCATAGTATTTGCCGATGTTGATTCGGTCACCGGCCGAAAAGTATGCACT
CAGCACAGGGCAGGTACCACGGAGCGAAAGGTGGATGATTTGCAGGGGCCGCTGGCG
CACCTACATAACTATTCGTCCGCGCCTGCTGGAGCGACCAACTACGCTCTATAGCGTTC
AGTACCCGAGCAGTCTCCTAACTTGGCCGATTCTATTGTATAAACAGTCGTTGGGACGC
GGACTAAACAACCTCCTCATACCCATCCTCCCGTTCGCAGCAGAACGACTCGACGGTA
CCGGCGTCAAGCGCCTCCGCCTAGGCTGCATCATTATGTTGGGAGATGCGTCGACTGG
GGGCCGGCGACCTGAGCTCGGTCGTCCCGTGAAGCCAATCAGTCTCATGGAGCCGTTC
TCTCCCATACATTGGCCTCCTGGGCTTCCCATCAATTGTGTCGAAATATTGTCTAATGTG
AAATTTATTCATCGTGAGGTGTAAACCGCCCCCGCGTGACTGTAGAGCGTAATCCGGC
CTTCTGGGCTAGCAGCTAACGAGGAAGTGTGCTTCATTTCGTTCGGAAAAGACGAAAT
GCTCTAGGCGGCTCTCAACCAGCCTAGTCCCCACGATATATACAACCGTACCAGGTCG
AATTAATCCGCCGGGGTCCCGTCGTGGGATGTTTGAGGGGGAGATACGTTCCGGCCAT
ATACTTTTTGTCCGCATCCCTTGGGGAAATCCATTACGCTGCCGTTGATGTCGTTAACG
TCACGCCACTAACACCGAACTAGGGGGACCAAACTATACCATGTTATCCGTATACTTA
CCCACTGCCGCGGTTCAGAGTACGGGCCTGTGAGTAATGTACGCGCTCCTGGTGGGAA
CTTGTCCTCCGGACTTGATAGGTTACTCGCCGAGCTGGAACTTAGCAAACCACCCTGAT
GCATTCCAGGTATCAGATTAAAACACAATTTTGCTTTCACCAGTTCCCAATTGCGGGGC
CGTATCGTCGGGAGGCCTTCGACTAGATCTGCTCGCATGGTTCGAGCGGTAATATCGG
GTCTGAAATCCCCTGACCCAACGGGAGGCGGTATGCGTGATCGGAATCTGCACCTTGC
GAGAGCTAATCGTGATGACGATGGCTCTTCCACAAGTGAGTGCTGGGGCGCGAGCGGC
GAACCAGATGGTCCTCTGAATGTTCCCCCCCGTGCCGAAGGCTGAACTCGAGGCGGAG
CCGTGGTCGCGCACCGTTACCACGCTGGTGCGTCGCGAGTCAACTTGAAGGTCCAGTC
ATGAAG

>BMX_BMX0015.2

TTCAAGATGCCCTCCTGACTCTGCCACGACGCGGTGGCTTCGCGCATGGACCGGATAA
GTTTTCCCGTGGACCGCTCAACCCCGGAAATACCCCGAGGTTTTCTCAGTGGTTACCTT
GATGCCGCGCAGCGTCTGCTTGGATGCTGAACCGCGAGAGCTATTCAGGTGCTCGACT
ACTCGACCGGGAGCAGCACGCAGCCGCGGGAACGACGCGGTCTACCTCCGGCCAGCA
GGGGGTACGACGTCCCGGCCCGTTTTCCCGCGTCGGAGGCTGCCAACACGATAGTAAC
TGAAATGGCCCAAACGTATTAATACGCACAGGGGGCGGGCCTCTGGCGCGCCACTGGA
TCAGCCCCGTGGCGTGCCCGCGTCGTCAGCGCCACCCATTGCTAAGCGCTGACAGTAA
TAGACCCCTCCATAGTATTTGCCGATGTTGATTCGGTCACCGGCCGAAACGTATGCACT
CAGCACAGGGCAGGTACCACGGAGCGAAAGGTGGATGATTGGCAGGGGCCGCTTGCG
CACCTACATAACTATTCGTCCGCGCCTGCTGGAGCGACCAACTACGCTCTATAGCGTTC
AGTACCCGAGCAGTCTCCTAACTTGGCCGATTCTATTGTATAAACAGTCGTTGGGACGC
GGACTAAACAACCTCCTCATACCCATCCTCCCGTTCGCAGCAGAACGACTCGACGGTA
CCGGCGTCAAGCGCCTCCGCCTAGGCTGCATCATTATGTTGGGAGATGCGTCGACTGG
GGGCCGGCGACCTGAGCTCGGTCGTCCCGTGAAGCCAATCAGTCTCATGGAGCCGTTC
TCTCCCATACATTGGCCTCCTGGGCTTTCCATCAATTGTGTCGAAATATTGTCTAATGTG
AAATTTATTCATCGTGAGGTGTAAACCGCCCCCGCGTGACTGTAGAGCGTAATCCGGC
CTTCTGGGCTAGCAGCTATTGAGGAAGTGTGCTTCATTTCGTTTGGAAAAGACGAAATG
CACTAGGCGGCTCTCAACCAGCCTAGTCCCCACGATACATACAACCGTACCAGGTCGA
ATTAATCCGCCGGGGTCCCGTCGTGGGATGTTTGAGGGGGAGATACGTTCCGGCCATA

TACTTTTTGTCCGCATCCCTTGGGGAAATCCATTACGCTGCCGTTGATGTCGTTAACGTC
ACGCCACTAACACCGAACTAGGGGGACCAAACTATACCATGTTATCCGTATACTTACC
CACTGCCGCGGTTCAGAGTACGGGCCTGTGAGTAATGTACGCGCTCCTGGTGGGAACT
TGTCCTCCGGACTTGAATGGTTACTCGCCGAGCTGGAACTTAGCAAACCACCCTGATGC
ATTCCAGGTATCAGATTAAAACACAATTTTGCTTTCACCAGTTCCCAATTGCGGGGCCG
TATCGTCGGGAGTCCTTCGACTAGATCCGCTCGCATGGTTCGAGCGGTAATATCGGGTC
TGAAATCCCCTGACCCAACGGGAGGCGGTATGCGTGATCGGAATCTGCACCTTGCGAG
AGCTAATCGTGATGACGATGGCTCTTCCACAAGTGAGTGCTGGGGCGCGAGCGGCGAA
CCAGATGGTCCTCTGAATGTTCCCCCCCGTGCCGAAGGCTGAACTCGAGGCGGGGCCG
TGGTCGCGCACCGTTACCACGCAGGTGCGTCGCGAGTCAACTTGAAGGTCCAGTCATG
AAG

>LWU_LWH0F.1

GTCGGGTTGCCTCCTCGGCTCTGGCACGGCGCGGCGCTTTCGCGCACGGATCGGATAA
GCTTTTCCGTGGACCACTCGTCCTCAGAAATATCTCGTAGTCTTCTCGTTGGTTATTCCA
ATGAAGCGCCTCGTCTGTGGGGGCACTGAACCGCTAGAGCTATTCGAGTGCTCTACTA
CTCCATCGGGGGCAGCACGCAGCCGTGGGATCGGCACGGCCCACCTCCAGCCAACGAG
AGTTACGAGTACCCGACGCGTTTTCCCGCGTCGGAGGCTGCCAACACGATAGTAACTG
AACTGGCCCAAACGTATTAATACGCCCCGGGGGCGGGCCTCTGGCGCGCCACTGGATC
AGGCCCGTGGCATGCCCGCCTCGTCAGCGCCACCCATTGCTAAGCGCTGACAGTAATA
GGCCCCTCCATAGTAGTTGCCGATGTTGATTCGGTCACCGGTCGAAACGTATACACTCG
GTACGGGGTGGGTACCACGGAGCGAAAGGTGGATGATTGGCAGGAGCCGCTGGCGCA
CCTACAAAACTATTCGTTCGCGCCTGCTGGAATGACCAACTACGCTCTATAGCGTCCAG
TACTCGAGTAGTCCCTCAATTGGTCCGATGAGACTGTATACGCCGCCGTTGGGACGCG
GCCTAAATAACCCCTTTACGCACATCCGCCCATCCGGAGCGGAACGACTCGGCGGTAC
CGGCGTCAGGCCCCCTCGCCTAGACTGCACCATTATGTTGGGAGGTGCGTCGACTGGG
GGCCGGTGACTTGATCCTGGTCGGCCCGTGAGGCCCATCAGTCCTATGGAGACGTTCTC
TCCCATCCATTGGCCTCCTAGGCTTTCCACCAATTGCGTCAGGGTAATGCCAGATGTTA
GATGTATTCATCGTGAGGTATACACCGCCCCCGCGTGGGTGCGGCTCGAAGCCCGGCC
TCCCAGGCTGGCTGCTAACACGGAAGTGTAGCCGGTTCCACTCAGAGGGCATGAGGCA
GTACCTACGGATATCAACAAGCCTAGTCCCCATGATACGTACAGCTGGGCCAGGTGGG
ATCAATACGTCGGAAGCCCATCGCGGGATGTTTGAGGGGGAGATCCGTTCCGGCCATA
TACTCCCTGTCCGCATCCCGTGGGGTAATCCATTACGCCGTCGTTGACGTCATCAATGC
CGCGCGATTAACACCGAGCTGATGGGACCAAAACATACCATGTAATCCGTGTACTTAC
CCATCGTTGCGGTACAGGATACGGGGCTGTGAGTAATGTACACACTCCTGGAGGGAGC
TTGTCCTCCAGACTTGAATGGCTACCTGCCGGGCTGGGACTTAGCAAACCACCCTGACG
CATCCCAGGCCTTTGATCGGATCACAATTTTGCTTTCACCAGTTCCCAATTACGGTTCC
GCCTCGTCGGGAGGCCTTCGACTAGATCTACTCGCATGGTTCGAGCAGTAATATCGGGT
CTGAAATCCCCTGACCCAACGGGGGGCAGTATGTGTGGTCGGAATCTGCACCTTGCGA
GAGATGATTGTAATGACGATGGACCTTCTACAAGTGAGTGCTGGGGCACGAGCGGCGG
ACCAGATGGTCTTCTGAATGTTCCTTCCCGTGCCGAAGACCGAACCCGAGGCGGAGCC
GTGGGCGTGCACCGTTGCCACGCAGGTGCACCGCGAGGCACCTTGAAGGTCCCGTCAT
GAAG

>LWU_LWH0F.2

GTCGGGATGCCTTCCCAACTCCGGCACGGCGCGGCGCCTTCGCGCACGGATCGGATAA
GCTTACCCGTGGATGGCTCGTTCTCAGAAATATCTCGTAGTCTTCTCGTTGGTTACTCCA
ATGAAGCGCCTCGTCTGCGGGGGCACTGAACCGCGAGAGCTATTCAAGTGCTCTACTA
CTCGACCGGGGGCAGCTTACAACCGCGGGATCGGCACGGTCTACCTCCGTCCAACAGG
GGGTACGACGACCCGGTCCCCCCTCCCGCGTCGAAGGCTGCCAACACGATAGTAAGCG

AACTGGCCCAAACGTATTAATACGTACAGGGGGCGGGCCTCTGGCGCGCCACTGGATC
AGGCCCGTGGCATGCCCGCCTCGTCAGCGCCACCCATTGCTAAGCGCTGACAGTAATA
GACCCCTCCATAGTAGTTGCCGATGTTGATTTGGTCACCGGCCGAAACGTATGCGCTCA
GCACAGGGCAGGTACTACGGAGCGAAAGGTGGATGATTGGCAGGGGCCGCTGGCGCA
CCTACAAAACTATTCGTCCGCGCCTGCTGGAGCGACCAACTACGCTCTATAGCGTCCAG
TACCCGAGCAGTCCCTCAATTGGTCCGATGAGACTGTATACGCCGCCGTTGGGACGCG
GACTAAACAACCCCCTCATACCCATTCGCCCGTCCAGAGCGGAACGACTCCGCGGTGC
CGGCGTCAGGCCTCCTCGCCTAGGCTGCGCCATTATGTTGGGAGGTGCGTTGACTGGG
GGCTGGCAATTTGATCCCGGTCGGCCCGTGAAGCCCATCAGTTCCATGGAGACATTCGT
TGCCGTCCATTGGCCTTCTGGGCCCTCCGCCAATTACGTCGGAGTATCGCCTAGGGTGA
AATGCATTCAACGTGAGGTATAAACCGCCCCCGCGTGGGTGCGGCTCGATGTCTGGCC
TCCCAGGCTGGCAGCTAACACGGAAGTGTGCCTCGTTCCACTCAGAGGGCATGAGGCA
GTACCTACGGATCTCAACAAGCCTAGTCCCCATGATACGTTCAGCTGGGCCAGGTGGG
ATTAATACGCCGGAAGCCCGTCGCGGGATGTTTGAGGGGGAGATCCGTTCCGGCCATA
TACTCTCTGTCCGCATCCCTTGGGGAAAGCCATTACGCTGCCGTTGACGTCGTTAACGC
CGCTTCATTAACGTCGAACTAGGGAGACCAAACTACATCATGTTATCTGTATGCCCAGT
TGTTGTTGCGGTTCAGGGTACGGGGCTGTGAGGCACGGGCGCGCTCCTGGTGGGAACT
TGTCCTCCGGACTTTAATGGCTACTCGCCGGGCTGAGACTTAGCAGACCACCCTGACGC
ATTCCAGGCCTCAGATTAGATCACAATTTTGCTTTCATTAGTTCCCAATTACGGTTCCGC
ATCGTCAGGAGGCCTTCGACTAGATCTACTCGTATGGTTCGAGCGGTGATATCGGGTCT
AAAATCCCCTGACCCAATGGGGGGCGGTACGTGTGATCGGAATCTGCACCTTGCGAGA
TCTAATCGTAATGACGATGGCTCTTCCACAAGTGAGTGCTGGGGCGCGAGCGGCGGAC
CAGATGGCCTTCCGAATGTTCCCCCCCGTGCCGCAGGCTTAACTCGAGGCGGAGCCGT
GGGCTCGCACCGTTGCCGCGCAGGTACACCGCGAGGTCCCTTGAAGGTCCCATCATGA
AG

>LWU_LWH0M.1

GTCGGGATGCTTTCCCAACTCCGGCACGGCGCGGCGCCTTCGCGCACGGATCGGATAA
GCTTTCCCGTGGACGGCTCGTCCTCAGAAACATTTCGTAGTCTTCTCGTTGGTTACTCCA
CTGCCGCGCCGCGTCTGCTTGGATGTTGAACCGCGAGAGCTATTCAAGTGCTTTACTAC
TCGACCGGGGGCAGCTTACAACCGTGGGATCGGCGTGGCCCACCTCCGGCCAACGAGA
GTTACGAGTACCCGGCCCGTTTTCCCGCGTCGGAGGCTGCCAACACGATAGTAGCTGA
ACTGGCCCAGACGTATTGATACGCCCCGGGGGCGGGCCTCTGGCGCGCTACTGGATCA
GGCCCGTGGCGGGCCTGCCTCGTCAGCGCCACCCATTGCTAAGCGCTGACAGTAATAG
ACCCCTCCATAGTTGTTGCCGATGTTAATTCGGTCACCGGCCGAAACGTATGCACTTAG
CACAGGGCAGGTACTACAAGGCGAGAGGTGGACGATTGGCAAAGGCTGCTGGCGAGC
CTACCAACCTGTTCCTCCGCGCCTGCTGGAGCGGCCAACTACGCCCCGCAGCGACCGG
TCCCCGAGCAGTCTCTCAACTGGTTCGATGAGACTGTATACACCGTTGTTGGGACGCGG
ACTAAACCGCCCCCTCATACCCACCCGCCCGTCCGGAACGGAACGACTCGGCGGTACC
GGCGTCAGGCCCCCTCGCCTAGGCTGCACCATTATGTTGGGAGGTGCGTCGACTGGGG
GCCGGCGACTTGAGCTCGGTCGTCCCGTGAAGCCCATCAGGCCCATGGAGACGTTCTC
TCCCATCCATTAGCCTCCGGGGCTCTCCACCAATCGCACCGGAGTCTTGTCTAGTGTTA
AATGTATTCATCGTGGGGTATAAACCGCCCCCGCGTGGGTGCGGCTCGAAGTCTGGCC
TCCCAGGCTGCTAGCTAACACGGAAGTGTGCCTCGTTTCGCTCAGAGGGGACGAGACA
CTCTCGACGGCTCTCAACAAGCCTAGTCCCCACGATACATACAACCGTGCCCGGTGGG
ATTAATACACCGGGGTCCCGTCGCGGGACGTCTGGGATGGGACCATGTTCCGGCCGTA
TACTCCCTGTTCGTGCCCCTCGGAGAAATCCATTACGCTGCCGTTGACGTCGTTAACGT
CACGCCACTAACACCGAACTAGGGGGACCAAACTATACCATGTAATCCGTGCGCCTAC
CCACTGTTGCGATTCAGGGTACGGGGCTGTGAGTAATGTACACACTCCTGGTGTGAGCT
TGTCCTCCGGACTTGAATGGCTACTCGCCGGGCTGGACTTAGCAAACCACCCTGACG
CATTCCAGGCCTCTGATCAGATCACAATTTTGCTTTCACCAGTTCCCAATTACGGTTCC

GCATCGTCGGGAGGCCTTCGACTAGATCTACTCGCATGGTTCGAGCGGTAATATCGGG
TCTGAAATCCCCTGACCCAACGGGAGGCGGTACGTGTGATCAGGATCTGCACCTTGCG
AGAGCCAAGCGTAATGACGGTGGCTCTTCCACCTCTAAAAGCTCGGGCGCGAGCGGCG
GGCTGGATGGTCCCCCGAATGTTCCCCCCTATGCCGAAGGCTGAACTCGAGGCTGAGC
CGTGGGCGCGCACCGTTGCCACGCAGGTGCACCGCGAGGTCCCTTGAAGGCCCCGTCA
CGAAG

>LWU_LWH0M.2

GTCGGGATGCCTTCCCAACTCTGGCACGGCGCGGCGCCTTCGCGCACGGATCGGATAA
GCTTTCCCGTGGACGGCTCGTCCTCGGAAATATCTCGTAGTCTTCTCGTTGGTTGCTCCA
ACGAAGCGACGAATCTACTTGGATACTGAACCGCGAGAGCTATTCAAGTGCTCTACTA
CTCGACCGGGGGCAGCATACAGCCGTGGGATCGGCGCGGCCCACCTCCGGCCAACGAG
AGTTACGAGTACCCGGCCCGTTTTCCCGCGTCGGAGGCTGCCAACACGATAGTAACTG
AACTGGCCCAAACGTATTGATACGCCCCGGGGGCGGGCCTCTGGCGCGCCACTGGATC
AGGCCCGTGGCGTGCCCGCCTCGTCAGCGCCACCCATTGCTAAGCGCTGACAGTAATA
GACCCCTCCATAGTAGTTGCCGATGTTAATTCGGTCACCGGCCGAAACGTATGAACGC
AGCACAGGGCAGGTACTACGGAGCGAAAGGTAGCTGATTGGCAGGGGCTGCTGGCGC
GCCTACCAACCTGTTCCTCCGCGCCTGCTGGAGCGACCAACTACGCCCCGCAGCGACC
GGTACTTGAGCTGTCTCTCAACTGGTTCGATGAGACTGTATACACCGTCGTTGGGACGC
GGACTAAACAACCTCCTCATACCAATCCGCCCGTCCGGAGCGGAACGACTCGGCGGTA
CCGGCGTCAGGCCCCCTCGCCTAGGCTGCACCATTATGTTGGGAGGTGCGTCGACTGG
GGGCCGGCGACTTGAGCTCGGTCGTCCCGTGAAGCCCATCAGTCCCATGGAGACGTTC
TCTCCCATCCATTAGCCTCCGGGGCTCTCCACCAATCGCACCGGAGTCTTGTCTAGTGT
TAAATGTATTCATCGTGGGGTATAAACCGCCCCCGCGTGGGTGCGGCTCGAAGTCTGG
CCTCCCAGGCTGCTAGCTAACACGGAAGTGTGCCTCGTTTCGCTCAGAGGGGACGAGA
CACTCTCGACGGCTCTCAACAAGCCTAGTCCCCACGATACATACAACCGTGCCCGGTG
GGATTAATACACCGGGGTCCCGTCGCGGGACGTCTGGGATGGGACCATGTTCCGGCCG
TATACTCCCTGTTCGTGCCCCTCGGAGAAATCCATTACGCTGCCGTTGACGTCGTTAAC
GTCACGCCACTAACACCGAACTAGGGGGACCAAACTATACCATGTAATCCGTGCGCCT
ACCCACTGTTGCGATTCAGGGTACGGGGCTGTGAGTAATGTACACACTCCTGGTGTGA
GCTTGTCCTCCGGACTTGAATGGCTACTCGCCGGGCTGGGACTTAGCAAACCACCCTGA
CGCATTCCAGGCCTCTGATCAGATCACAATTTTGCTTTCACCAGTTCCCAATTACGGTT
CCGCATCGTCGGGAGGCCTTCGACTAGATCTACTCGCATGGTTCGAGCGGTAATATCG
GGTCTGAAATCCCCTGACCCAACGGGAGGCGGTACGTGTGATCAGGATCTGCACCTTG
CGAGAGCCAAGCGTAAGGACGGTGGCTCTTCCACAAGTAAAAGCTCGGGCGCGAGCG
GCGGGCTGGATGGTCCCCCGAATGTTCCCCCCTATGCCGAAGGCTGAACTCGAGGCTG
AGCCGTGGGCGCGCACCGTTGCCACGCAGGTGCACCGCGAGGTCCCTTGAAGGTCCCG
TCACGAAG

>LWU_LWH13.1

GTCGGGTTGCCTCCTCGGCTCTGGCACGGCGCGGCGCTTTCGCGCACGGATCGGATAA
GCTTTTCCGTGGACCACTCGTCCTCAGAAATATCTCGTAGTCTTCTCGTTGGTTATTCCA
ATGAAGCGCCTCGTCTGTGGGGGCACTGAACCGCTAGAGCTAGTCGAGTGCTCTACTA
CTCCACCGGGGGCAGCACGCAGCCGTGGGATCGGCACGGCCCACCTCCAGCCAACGAG
AGTTACGAGTACCCGACGCGTTTTCCCGCGTCGGAGGCTGCCAACACGATAGTAACTG
AACTGGCCCAAACGTATTATTACGCCCCGGGGGCGGGCCTCTGGCGCGCCACTGGATC
AGGCACGTGGCGTGCCCGCCTCGTCAGCGCCACCCATTGCTAAGCGCTGACAGTAATA
GACCCCTCCATAGTAGTTGCCGATGTTGATTCGGTCACCGGCCGAAACGTATGCACTTA
GCACAGGGCAGGTACTACAAAGCGAGAGGGGGATGATTGGCAGGGGCTGCTGACGCG
CCTATCAGCCCGTTCCCCCGCGCCTGCTGTGGCGACCAACTACGCCCCGCAGCGTCCAG

TACCCGAGCAGTCTCTCAACTGGTCCGATTAGACTGTATACACCGCCGTTGGGACGCG
GACTAATCAACTCCCTCATACCCATCCGCCCGTCCGGAGCGGAACGACTCGGCGGTAC
CGGCGTCAGGCCCCCTCGCCTAGACTGCACCATTATGTTGGGAGGTGCGTCGACTGGG
GGCCGGCGACTTGATCCTGGTCGGCCCGTGAAGCCCATCAGTCCCATGGAGACGTTCT
CTCCCATCTATTGGCCTCCGGGGCTCTCCACCAATCGCACCGGAGTCTTGTCTAATATG
AAATTTAAACATCGTGAGGTATAAACCGCCCCCGAGTGGGTGCGGCTTGAAGTCCAGC
CCCCCAGGCCGCCTGCTAACACGGAAGTGTACCCGGTTCCACTCAGAGGGCATGAGGC
AGTACCTACGGATCTCAACAAGCCTAGTCCCCATGATACGTACAGCTGGGCCAGGTGG
GATTAATACGCCGGGGTTCCGTTGCGGAACGGCTAGAAGGTAAATACGCCTTGGCCAT
ATACTCTTTGTCCGCATCCCTTGGGGAAATCCATTATGCTGCCGTTGACGTCGTCAACG
TCACGCCACTAACACCGAACTAGGGGGACCAAACTATACCATGTTATCCGTATACTTA
CCCACTGTTGCAGGTCAAGGTATGGGGCTGCAAGTAATGTACATACTCCTGGTGGGAA
CTTGTCCTCCGGACTTGAATGGCTACCTGCCGGGCTGGGACTTAGCAAACCACCCTGAC
GCATCCCAGGCCTTTGATCGGATCACAATTTTGCTTTCACCAGTACCCAATTACGGTTC
CGCATCGTCGGGAGGCCTTCGACTAGATCTACTCGCATGGTTCGAGCAGTAATATCGG
GTCTGAAATCCCCTGACCCAACGGGGGGCAGTATGTGTGGTCGGAATCTGCACCTTGC
GAGAGATAATCGTAATGACGATGGACCTTCTACAAGTGAGTGCTGGGGCACGAGCGGC
TGACCAGATTGTCTTCCGAATGTTCCCCCCCGTGCCGAAGGCTGAACCCGAGGCGGAG
CCGTGGGCGTGCACCGTTGCCACGCAGGTGCACCGCGAGGCACCTTGAAGGTCCCGTC
ATGAAG

>LWU_LWH13.2

GTCGGGTTGCCTCCTCGGCTCTGGCACGGCGCGGCGCTTTCGCGCACGGATCGGATAA
GCTTTTCCGTGGACCACTCGTCCTCAGAAATATCTCGTAGTCTTCTCGTTGGTTATTCCA
ATGAAGCGCCTCGTCTGTGGGGGCACTGAACCGCTAGAGCTATTCGAGTGCTCTACTA
CTCCATCGGGGGCAGCACGCAGCCGTGGGATCGGCACGGCCCACCTCCAGCCAACGAG
AGTTACGAGTACCCGACGCGTTTTCCCGCGTCGGAGGCTGCCAACACGATAGTAACTG
AACTGGCCCAAACGTATTAATACGCCCCGGGGGCGGGCCTCTGGCGCGCCACTGGATC
AGGCCCGTGGCGTGCCCTCCCCCTTAGCGCCACCCATTGCTAAGCGCTGACAGTAATA
GACCCCTCCATAGTAGTTGCCGATTTTGATTCGGTCACCGGCCGAAACGTATGCACTTA
GCACAGGGCAGGTACTACAAAGCGAGAGGGGGATGATTGGCAGGGGCTGCTGACGCG
CCTATCAGCCCGTTCCCCCGCGCCTGTTGTGGCGACCGACTACGCCCCGCAGTGACCGG
TACCCGAGCAGTCTCCCATCTGGTCTGATGAGACTGTATACACCGCCGTTGGGATGCGG
ACTAAACAACCCCCTCATACCCATCCGCTTGTCCGCAGCAGAACGACTCGGCGGTACT
GGCGTCAGGCCCCCTCGCCTAGACTGCACCATTATGTTGGGAGGTGCGTCGACTGGGG
GCTGGCAACCTGAGGTCGGCCGTTTCGTGAAGCCCATTAGTCCCATGGAGACGTTCTCT
CCCATCCATTGGCCTCCGGGGCTCTTCATCAATCGCGTCGGAGTATCGTCTAATGTGAA
ATTTATTCATCGTGAGGTATACACCGCCCCCGCGTGGGTGCGGCTCGAAGCCCGGCTTC
CCAGGCTGGCAGCTAACACGGAAGTGTGCCTCGTTCCACTCAGAGGGCATGAGGCAGT
ACCTACGGATCTCAACAAGCCTAGTCCCCATGATACGTTCAGCTGGGCCAGGTGGGAT
TAATACGCCGGGGTCCCGTCGCGGAACGGCTAGAAGGTAAATACGCCCTGGCCATATA
CTCTTTGTCCGCATCCCTTGGGGAAATCCATTATGCTGCCGTTGACGTCGTCAACGTCA
CGCCACTAACACCGAACTAGGGGGACCAAACTATACCGTGTTATCCGTATACTTACCC
ACTGTTGCAGGTCAAGGTATGGGGCTGCAAGTAATGTACATACTCCTGGTGGGAACTT
GTCCTCCGGACTTGAATGGCTACCTGCCGGGCTGGGACTTAGCAAACCACCCTGACGC
ATCCCAGGCCTTTGATCGGATCACAATTTTGCTTTCACCAGTTCCCAATTACGGTTCCG
CATCGTCGGGAGGCCTTCGACTAGATCTACTCGCATGGTTCGAGCAGTAATATCGGGTC
TGAAATCCCCTGACCCAACGGGGGGCAGTATGTGTGGTCGGAATCTGCACCTTGCGAG
AGATAATCGTAATGACGATGGACCTTCTACAAGTGAGTGCTGGGGCACGAGCGGCTGA
CCAGATTGTCTTCCGAATGTTCCCCCCCGTGCCGAAGGCTGAACCCGAGGCGGAGCCG

TGGGCGTGCACCGTTGCCACGCAGGTGCACCGCGAGGCACCTTCAAGGTCCCGTCATG
AAG

>LWU_LWH181.1

GTCGGGATGCTTTCCCAACTCCGGCACGGCGCGGCGCCTTCGCGCACGGATCGGATAA
GCTTTCCCGTGGACGGCTCGTCCTCAGAAACATTTCGTAGTCTTCTCGTTGGTTACTCCA
CTGCCGCGCCGCGTCTGCTTGGATGTTGAACCGCGAGAGCTATTCAAGTGCTTTACTAC
TCGACCGGGGGCAGCTTACAACCGTGGGATCGGCGTGGCCCACCTCCGGCCAACGAGA
GTTACGAGTACCCGGCCCGTTTTCCCGCGTCGGAGGCTGCCAACACGATAGTAGCTGA
ACTGGCCCAGACGTATTGATACGCCCCGGGGGCGGGCCTCTGGCGCGCTACTGGATCA
GGCCCGTGGCGGGCCCGCCTCGTCAGCGCCACCCATTGCTAAGCGCTGACAGTAATAG
ACCCCTCCATAGTTGTTGCCGATGTTAATTCGGTCACCGGCCGAAACGTATGCACTTAG
CATAGGGCAGGTACTACAAAGCGAGAGGTGGACGATTGGCAAAGGCTGCTGGCGAGC
CTACCAACCTGTTCCTCCGCGCCTGCTGGAGCGGCCAACTACGCCCCGCAGCGACCGG
TCCCCGAGCAGTCTCTCAACTGGTTCGATGAGACTGTATACACCGTTGTTGGGACGCGG
ACTAAACCGCCCCCTCATACCCACCCGCCCGTCCGGAACGGACCGACTCGGCGGTACC
GGCGTCAGGCCCCCTCGCCTAGGCTGCACCATTATGTTGGGAGGTGCGTCGACTGGGG
GCCGGCGACTTGAGCTCGGTCGTCCCGTGAAGCCCATCAGTCCCATGGAGACGTTCTCT
CCCATCCAGTAGCCTCCGGGGCTCTCCACCAATCGCACCGGAGTCTTGTCTAGTGTTAA
ATGTATTCATCGTGGGGTATAAACCGCCCCCGCGTGGGTGCGGCTCGAAGTCTGGCCTC
CCAGGCTGCTAGCTAACACGGAAGTGTGCCTCGTTTCGCTCAGAGGGGACGAGACACT
CTCGACGGCTCTCAACAAGCCTAGTCCCCACGATACATACAACCGTGCCCGGTGGGAT
TAATACACCGGGGTCCCGTCGCGGGACGTCTGGGATGGGACCATGTTCCGGCCGTATA
CTCCCTGACCGTGCCCCTCGGAGAAATCCATTACGCTGCCGTTGACGTCGTTAACGTCA
CGCCACTAACACCGAACTAGGGGGACCAAACTATACCATGTAATCCGTGCGCCTACCC
ACTGTTGCGATTCAGGGTACGGGGCTGTGAGTAATGTACACACTCCTGGTGTGAGCTTG
TCCTCCGGACGTGAATGGCTACTCGCCGGGCTGGGACTTAGCAAACCACCCTGACGCA
TTCCAGGCCTCTGATCAGATCACAATTTTGCTTTCACCAGTTCCCAATTACGGTTCCGC
ATCGTCGGGAGGCCTTCGACTAGATCTACTCGCATGGTTCGAGCGGTAATATCGGGTCT
GAAATCCCCTGACCCAACGGGAGGCGGTACGTGTGATCAGGATCTGCACCTTGCGAGA
GCCAAGCGTAAGGACGGTGGCTCTTCCACAAGTAAAAGCTCGGGCGCGAGCGGCGGG
CTGGATGGTCCCCCGAATGTTCCCCCCTATGCCGAAGGCTGAACTCGAGGCTGAGCCG
TGGGCGCGCACCGTTGCCACGCAGGTGCACCGCGAGGTCCCTTGAAGGTCCCGTCACG
AAG

>LWU_LWH181.2

GTCGGGATGCCTTCCCAACTCTGGCACGGCGCGGCGCCTTCGCGCACGGATCGGATAA
GCTTTCCCGTGGACGGCTCGTCCTCGGAAATATCTCGTAGTCTTCTCGTTGGTTGCTCCA
ACGAAGCGACGAATCTACTTGGATACTGAACCGCGAGAGCTATTCAAGTGCTCTACTA
CTCGACCGGGGGCAGCATACAGCCGTGGGATCGGCGCGGCCCACCTCCGGCCAACGAG
AGTTACGAGTACCCGGCCCGTTTTCCCGCGTCGGAGGCTGCCAACACGATAGTAACTG
AACTGGCCCAAACGTATTGATACGCCCCGGGGGCGGGCCTCTGGCGCGCCACTGGATC
AGGCCCGTGGCGTGCCCGCCTCGTCAGCGCCACCCATTGCTAAGCGCTGACAGTAATA
GACCCCTCCATAGTAGTTGCCGATGTTAATTCGGTCACCGGCCGAAACGTATGAACGC
AGCACAGGGCAGGTACTACGGAGCGAAAGGTAGCTGATTGGCAGGGGCTGCTGGCGC
GCCTACCAACCTGTTCCTCCGCGCCTGCTGGAGCGACCAACTACGCCCCGCAGCGACC
GGTACTTGAGCTGTCTCTCAACTGGTTCGATGAGACTGTATACACCGTCGTTGGGACGC
GGACTAAACAACCTCCTCATACCAATCCGCCCGTCCGGAGCGGAACGACTCGGCGGTA
CCGGCGTCAGGCCCCCTCGCCTAGGCTGCACCATTATGTTGGGAGGTGCGTCGACTGG
GGGCCGGCGACTTGAGCTCGGTCGTCCCGTGAAGCCCATCAGTCCCATGGAGACGTTC

TCTCCCATCCATTAGCCTCCGGGGCTCTCCACCAATCGCACCGGAGTCTTGTCTAGTGT
TAAATGTATTCATCGTGGGTATAAACCGCCCCCGCGTGGGTGCGGCTCGAAGTCTGG
CCTCCCAGGCTGCTAGCTAACACGGAAGTGTGCCTCGTTTCGCTCAGAGGGGACGAGA
CACTCTCGACGGCTCTCAACAAGCCTAGTCCCCACGATACATACAACCGTGCCCGGTG
GGATTAATACACCGGGGTCCCGTCGCGGGACGTCTGGGATGGGACCATGTTCCGGCCG
TATACTCCCTGTTCGTGCCCCTCGGAGAAATCCATTACGCTGCCGTTGACGTCGTTAAC
GTCACGCCACTAACACCGAACTAGGGGGACCAAACTATACCATGTAATCCGTGCGCCT
ACCCACTGTTGCGATTCAGGGTACGGGGCTGTGAGTAATGTACACACTCCTGGTGTGA
GCTTGTCCTCCGGACTTGAATGGCTACTCGCCGGGCTGGGACTTAGCAAACCACCCTGA
CGCATTCCAGGCCTCTGATCAGATCACAATTTTGCTTTCACCAGTTCCCAATTACGGTT
CCGCATCGTCGGGAGGCCTTCGACTAGATCTACTCGCATGGTTCGAGCGGTAATATCG
GGTCTGAAATCCCCTGACCCAACGGGAGGCGGTACGTGTGATCAGGATCTGCACCTTG
CGAGAGCCAAGCGTAATGACGGTGGCTCTTCCACAAGTAAAAGCTCGGGCGCGAGCG
GCGGGCCGGCTGGTCCCCCGAATGTTCCCCCCTATGCCGAAGGCTGAACTCGAGGCTG
AGCCGTGGGCGCGCACCGTTGCCACGCAGGTGCACCGCGAGGTCCCTTGAAGGTCCCG
TCACGAAG

>LWU_LWH879.1

GTCGGGATGCTTTCCCAACTCCGGCACGGCGCGGCGCCTTCGCGCACGGATCGGATAA
GCTTTCCCGTGGACGGCTCGTCCTCAGAAACATTTCGTAGTCTTCTCGTTGGTTACTCCA
CTGCCGCGCCGCGTCTGCTTGGATGTTGAACCGCGAGAGCTATTCAAGTGCTTTACTAC
TCGACCGGGGGCAGCTTACAACCGTGGGATCGGCGTGGCCCACCTCCGGCCAACGAGA
GTTACGAGTACCCGGCCCGTTTTCCCGCGTCGGAGGCTGCCAACACGATAGTAGCTGA
ACTGGCCCAGACGTATTGATACGCCCCGGGGGCGGGCCTCTGGCGCGCTACTGGATCA
GGCCCGTGGCGTGTCCGCCTCGTCAGCGCCACCCATTGCTAAGCGCTGACAGTAATAG
ACCCCTCCATAGTTGTTGCCGATGTTAATTCGGTCACCGGCCGAAACGTATGCACTTAG
CACAGGGCAGGTACTACAAAGCGAGAGGTGGACGATTGGCAAAGGCTGCTGGCGAGC
CTACCAACCTGTTCCTCCGCGCCTGCTGGAGCGGCCAACTACGCCCCGCAGCGACCGG
TACCCGAGCAGTCTCTCAACTGGTTCGATGAGACTGTATACACCGTTGTTGGGACGCGG
ACTAAACCGCCCCCTCATACCCACCCGCCCGTCCGGAACGGAACGACTCGGCGGTACC
GGCGTCAGGCCCCCTCGCCTAGGCTGCACCATTATGTGGGGAGGTGCGTCGACTGGGG
GCCGGCGACTTGAGCTCGGTCGTCCCGTGAAGCCCATCAGTCCCATGGAGACGTTCTCT
CCCATCCATTAGCCTCCGGGGCTCTCCACCAATCGCACCGGAGTCTTGTCTAGTGTTAA
ATGTATTCATCGTGGGTATAAACCGCCCCCGCGTGGGTGCGGCTCGAAGTCTGGCCTC
CCAGGCTGCTAGCTAACACGGAAGTGTGCCTCGTTTCGCTCAGAGGGGACGAGACACT
CTCGACGGCTCGCAACAAGCCTAGTCCCCACGATACATACAACCGTGCCCGGTGGGAT
TAATACACCGGGGTCCCGTCGCGGGACGTCTGGGATGGGACCATGTTCCGGCCGTATA
CTCCCTGTTCGTGCCCCTCGGAGAAATCCATTACGCTGCCGTTGACGTCGTTAACGTCA
CGCCACTAACACCGAACTAGGGGGACCAAACTATACCATGTAATCCGTGCGCCTACCC
ACTGTTGCGATTCAGGGTACGGGGCTGTGAGTAATGTACACACTCCTGGTGTGAGCTTG
TCCTCCGGACTTGAATGGCTACTCGCCGGGCTGGGACTTAGCAAACCACCCTGACGCA
TTCCAGGCCTCTGATCAGATCACAATTTTGCTTTCACCAGTTCCCAATTACGGTTCCGC
ATCGTCGGGAGGCCTTCGACTAGATCTACTCGCATGGTTCGAGCGGTAATATCGGGTCT
GAAATCCCCTGACCCAACGGGAGGCGGTACGTGTGATCAGGATCTGCACCTTGCGAGA
GCCAAGCGTAATGACGGTGGCTCTTCCACAAGTAAAAGCTCGGGCGCGAGCGGCGGGC
TGGATGGTCCCCCGAATGTTCCCCCCTATGCCGAAGGCTGAACTCGAGGCTGAGCCGT
GGGCGCGCACCGTTGCCACGCAGGTGCACCGCGAGGTCCCTTGAAGGTCCCGTCACGA
AG

>LWU_LWH879.2

GTCGGGATGCCTTCCCAACTCTGGCACGGCGCGGCGCCTTCGCGCACGGATCGGATAA
GCTTTCCCGTGGACGGCTCGTCCTCGGAAATATCTCGTAGTCTTCTCGTTGGTTGCTCCA
ACGAAGCGACGAATCTACTTGGATACTGAACCGCGAGAGCTATTCAAGTGCTCTACTA
CTCGACCGGGGGCAGCATACAGCCGTGGGATCGGCGCGGCCCACCTCCGGCCAACGAG
AGTTACGAGTACCCGGCCCGTTTTCCCGCGTCGGAGGCTGCCAACACGATAGTCACTG
AACTGGCCCAAACGTATTGATACGCCCCGGGGGCGGGCCTCTGGCGCGCCACTGGATC
AGGCCCGTGGCGTGCCCGCCTCGTCAGCGCCACCCATTGCTAAGCGCTGACAGTAATA
GACCCCTCCATCGTAGTTGCCGATGTTAATTCGGTCACCGGCCGAAACGTATGAACGC
AGCACAGGGCAGGTACTACGGAGCGAAAGGTAGCTGATTGGCAGGGGCTGCTGGCGC
GCCTACCAACCTGTTCCTCCGCGCCTGCTGGAGCGACCAACTACGCCCCGCAGCGACC
GGTACTTGAGCTGTCTCTCAACTGGTTCGATGAGACTGTATACACCGTCGTTGGGACGC
GGACTAAACAACCTCCTCATACCAATCCGCCCGTCCGGAGCGGAACGACTCGGCGGTA
CCGGCGTCAGGCCCCCTCGCCTAGGCTGCACCATTATGTTGGGAGGTGCGTCGACTGG
GGGCCGGCGACTTGAGCTCGGTCGTCCCGTGAAGCCCATCAGTCCCATGGAGACGTTC
TCTCCCATCCATTAGCCTCCGGGGCTCTCCACCAATCGCACCGGAGTCTTGTCTAGTGT
TAAATGTATTCATCGTGGGGTATAAACCGCCCCCGCGTGGGGGCGGCTCGAAGTCTGG
CCTCCCAGGCTGCTAGCTAACACGGAAGTGTGCCTCGTTTCGCTCAGAGGGGACGAGA
CACTCTCGACGGCTCTCAACAAGCCTAGTCCCCACGATACATACAACCGTGCCCGGTG
GGATTAATACACCGGGGTCCCGTCGCGGGACGTCTGGGATGGGACCATGTTCCGGCCG
TATACTCCCTGTCCGTGCCCCTCGGAGAAATCCATTACGCTGCCGTTGACGTCGTTAAC
GTCACGCCACTAACACCGAACTAGGGGGACCAAACTATACCATGTAATCCGTGCGCCT
ACCCACTGTTGCGATTCAGGGTACGGGGCTGTGAGTAATGTACACACTCCTGGTGTGA
GCTTGTCCTCCGGACTTGAATGGCTACTCGCCGGGCTGGGACTTAGCAAACCACCCTGA
CGCATTCCAGGCCTCTGATCAGATCACAATTTTGCTTTCACCAGTTCCCAATTACGGTT
CCGCATCGTCGGGAGGCCTTCGACTAGATCTACTCGCATGGTTCGAGCGGTAATATCG
GGTCTGAAATCCCCTGACCCAACGGGAGGCGGTACGTGTGATCAGGATCTGCACCTTG
CGAGAGCCAAGCGTAATGACGGTGGCTCTTCCACAAGTAAAAGCTCGGGCGCGAGCG
GCGGGCTGGATGGTCCCCCGAATGTTCCCCCCTATGCCGAAGGCTGAACTCGAGGCTG
AGCCGGGGGCGCGCACCGTTGCCACGCAGGTGCACCGCGAGGTCCCTTGAAGGTCCCG
TCACGAAG

>LWU_LWH1183.1

GTCGGGTTGCCTCCTCGGCTCTGGCACGGCGCGGCGCTTTCGCGCACGGATCGGATAA
GCTTTTCCGTGGACCACTCGTCCTCAGAAATATCTCGTAGTCTTCTCGTTGGTTATTCCA
ATGAAGCGCCTCGTCTGTGGGGGCACTGAACCGCTAGAGCTATTCGAGTGCTCTACTA
CTCCATCGGGGGCAGCACGCAGCCGTGGGATCGGCACGGCCCACCTCCAGCCAACGAG
AGTTACGAGTACCCGACGCGTTTTCCCGCGTCGGAGGCTGCCAACACGATAGTAACTG
AACTGGCCCAAACGTATTAATACGCCCCGGGGGCGGGCCTCTGGCGCGCCACTGGATC
AGGCCCGTGGCATGCCCGCCTCGTCAGCGCCACCCATTGCTAAGCGCTGACAGTAATA
GACCCCTCCATAGTAGTGGCCGATGTTGATTTGGTCACCGGCCGAAACGTATGCGCTCA
GCACAGGGCAGGTACTACGGAGCGAAAGGTGGATGATTGGCAGGGGCCGCTGGCGCA
CCTACAAAACTATTCGTCCGCGCCTGCTGGAGCGACCAACCACGCTCTATAGCGTCCA
GTACCCGAGCAGTCCCTCAATTGGTCCGATGAGACTGTATACGCCGCCGTTGGGACGC
GGACTAAACAACCCCCTCATACCCATTCGCCCGTCCAGAGCGGAACGACTCCGCGGTG
CCGGCGGCAGGCCTCCTCGCCTAGGCTGCGCCATTATGTTGGGAGGTGCGTTGACTGG
GGGCTGGCAATTTGATCCCGGTCGGCCCGTGAAGCCCATCAGTTCCATGGAGACGTTCT
TTCCCGTCCATTGGCCTTCTGGGCCCTCCGCCAATTACGTCGGAGTATCGCCTAGGGTG
AAATGCATTCAACGTGAGGTATAAACCGCCCCCGCGTGGGTGCGGCTCGATGTCTGGC
CTCCCAGGCTGGCAGCTAACACGGAAATGTGCCTCGTTCCACTCAGAGGGCATGAGGC
AGTACCTACGGATCTCAACATGCCTAGTCCCCATGATACGTTCAGCTGGGCCAGGTGG
GATTAATACGCCGGAAGCCCGTCGCGGGATGTTTGAGGGGGAGATCCGTTCCGGCCAT

ATACTCTCTGTCCGCATCCCTTGGGGAAAGCCATTACGCTGCCGTTGACGTCGTTAACG
CCGCTTCATTAACGTCGAACTAGGGAGACCAAACTACATCATGTTATCTGTATGCCCAG
TTGTTGTTGCGGTTCAGGGTACGGGGCTGTGAGGCACGGGCGCGCTCCTGGTGGGAAC
TTGTCCTCCGGACTTTAATGGCTACTCGCCGGGCTGAGACTTAGCAGACCACCCTGACG
CATTCCAGGCCTCAGATTAGATCACAATTTTGCTTTCATTAGTTCCCAATTACGGTTCCG
CATCGTCAGGAGGCCTTCGACTAGATCTACTCGTATGGTTCGAGCGGTGATATCGGGTC
TAAAATCCCCTGACCCAATGGGGGGCGGTACGTGTGATCGGAATCTGCACCTTGCGAG
ATCTAATCGTAATGACGATGGCTCTTCCACAAGTGAGTGCTGGGGCGCGAGCGGCGGA
CCAGATGGCCTTCCGAATGTTCCCCCCCGTGCCGAAGGCTTAACTCGAGGCGGAGCCG
TGGGCTCGCACCGTTGCCGCGCAGGTACACCGCGAGGTCCCTTGAAGGTCCCATCATG
GAG

>LWU_LWH1183.2

GTCGGGTTGCCTCCTCGGCTCTGGCACGGCGCGGCGCTTTCGCGCACGGATCGGATAA
GCTTTTCCGTGGACCACTCGTCCTCAGAAATATCTCGTAGTCTTCTCGTTGGTTATTCCA
ATGAAGCGCCTCGTCTGTGGGGGCACTGAACCGCTAGAGCTATTCGAGTGCTCTACTA
CTCCACCGGGGGCAGCACGGAGCCGTGGGATCGGCACGGCCCACCTCCAGCCAACGA
GAGTTACGAGTACCCGACGCGTTTTCCCGCGTCGGAGGCTGCCAACACGATAGTAACT
GAACTGGCCCAAACGTATTAATACGCCCCGGGGGCGGGCCTCTGGCGCGCCACTGGAT
CAGGCCCGTGGCATGCCCGCCTCGTCAGCGCCACCCATTGCTAAGCGCTGACAGTAAT
AGACCCCTCCATAGTAGTTGCCGATGTTGATTTGGTCACCGGCCGAAACGTATGCGCTC
AGCACAGGGCAGGTACTACGGAGCGAAAGGTGGATGATTGGCAGGGGCCGCTGGCGC
ACCTACAAAACTATTCGTCCGCGCCTGCTGGAGCGACCAACTACGCTCTATAGCGTCCA
GTACCCGAGCAGTCCCTCAATTGGTCCGATGAGACTGTATACGCCGCCGTTGGGACGC
GGACTAAACAACCCCCTCATACCCATTCGCCCGTCCAGAGCGGAACGACTCCGCGGTG
CCGGCGTCGGGCCTCCTCGCCTAGGCTGCGCCATTATGTTGGGAGGTGCGTTGACTGGG
GGCTGGCAATTTGATCCCGGTCGGCCCGTGAAGCCCATCAGTTCCATGGAGACATTCGT
TGCCGTCCATTGGCCTTCTGGGCCCTCCGCCAATTACGTCGGAGTATCGCCTAGGGTGA
AATGCATTCAACGTGAGGTATAAACCGCCCCCGCGTGGGTGCGGCTCGATGTCTGGCC
TCCCAGGCTGGCAGCTAACACGGAAGTGTGCCTCGTTCCACTCAGAGGGCATGAGGCA
GTACCTACGGATCTCAACAAGCCTAGTCCCCATGATACGTTCAGCTGGGCCAGGTGGG
ATTAATACGCCGGAAGCCCGTCGCGGGATGTTTGAGGGGGAGATCCGTTCCGGCCATA
TACTCTCTGTCCGCATCCCTTGGGGAAAGCCATTACGCTGCCGTTGACGTCGTTAACGC
CGCTTCATTAACGTCGAACTAGGGAGACCAAACTACATCATGTTATCTGTATGCCCAGT
TGTTGTTGCGGTTCAGGGTACGGGGCTGTGAGGCACGGGCGCGCTCCTGGTGGGAACT
TGTCCTCCGGACTTTAATGGCTACTCGCCGGGCTGAGACTTAGCAGACCACCCTGACGC
ATTCCAGGCCTCAGATTAGATCACAATTTTGCTTTCATTAGTTCCCAATTACGGTTCCGC
ATCGTCAGGAGGCCTTCGACTAGATCTACTCGTATGGTTCGAGCGGTGATATCGGGTCT
AAAATCCCCTGACCCAATGGGGGGCGGTACGTGTGATCGGAATCTGCACCTTGCGAGA
TCTAATCGTAATGACGATGGCTCTTCCACAAGTGAGTGCTGGGGCGCGAGCGGCGGAC
CAGATGGCCTTCCGAATGTTCCCCCCCGTGCCGAAGGCTTAACTCGAGGCGGAGCCGT
GGGCTCGCACCGTTGCCGCGCAGGTACACCGCGAGGTCCCTTGAAGGTCCCATCATGA
AG

>DRC_ERR173170.1

GTCGGGATGCCTTCCCAACTCTGGCACGGCGCGGCGCCTTCGCGCACGGATCGGATAA
GCTTTCCCGTGGACGGCTCGTCCTTGGAAATATCTCGTAGTCTTCTCGTTGGTTGCTCCA
ACGAAGCGACGAATCTACTTGGATACTGAACCGCGAGAGCTATTCAAGTGCTCTACTA
CTCGACCGGGGGCGGCATACAGCCGTGGGATCGGCGCGGCCCACCTCCGGCCAACGAG
AGTTACGAGTACCCGACGCGTTTTCCCGCGTCGGAGGCTGCCAACACGATAGTAACTG

AACTGGCCCAAACGTATTGATACGCCCCGGGGGCGGGCCTCTGGCGCGCCACTGGATC
AGGCCCGTGGCGTGCCCGCCTCGTCAGCGCCACCCATTGCTAAGCGCTGACAGTAATA
GACCCCTCCATAGTAGTTGCCGATGTAGGTCTGGTCACCGGCCGAAACGTATGAACGC
AGCACAGGGCAGGTACTACGGAGCGAAAGGTAGCTGATTGGCAGGGGCTGCTGGCGC
GCCTACCAACCTGTTCCTCCGCGCCTGCTGGAGCGACCAACTACGCCCCGCAGCGACC
GGTACCCGAGCAGTCTCTCAACTGGTTCGATGAGACTGTATACACCGTCGTTGGGACG
CGGACTAAACCGCCCCCTCATACCCACCCGCCCGTCCGGAGCGGAACGACTCGGCGGT
ACCGGCGTCAGGCCCCCTCGCCTAGGCTGCACCATTATGTTGGGAGGTGCGTCGACTG
GGGGCCGGCGACTTGAGCTCGGTCGTCCCGTGAAGCCCATCAGTCCCATGGAGACGTT
CTCTCCCATCCATTGGCCTCCGGGGCTCTCCACCAATCGCACCGGAGTCTTGTCTAGTG
TTAAATGTATTCATCGTGGGGTATAAACCGCCCCCGCGTGAGTGCGGCTCGAAGTCTG
GCCTCCCAGGCTGCTAGCTAACACGGAAGTGTGCCTCGTTTCGCTCAGAGGGGACGAG
ACACTCTCGACGGCTCTCAACAAGCCTAGTCCCCACGATACATACAACCGTGCCCGGT
GGGATTAATACGCCGGGGTCCCGTCGCGGGACATCTGGGATGGGAACACGTTCCGGCC
ATATGCTCTCTGTCCGCATCTCTTGGGGAAATCCATTACGCTGCCGTTGACGTCGTTAA
CGTCACGCCACTAATACCGAACTAGGGGGACCAAACTATACCATGTAATCCGTGCGCC
TACCCACTGTTGCGATTCAGGGTACGGGGCTGTGAGTAATGTACACACTCCTGGTGTGA
GCTTGTCCTCCGGACTTGAATGGCTACTCGCCGGGCTGGGACTTAGCAAACCACCCTGA
CGCATTCCAGGCCTCTGATCAGATCACAATTTTGCTTTCACCAGTTCCCAATTACGGTT
CCGCATCGTCGGGAGGCCTTCGACTAGATCTACTCGCATGGTTCGAGCGGTAATATCG
GGTCTGAAATCCCCTGACCCAACGGGAGGCGGTACGTGTGATCGGAATCTGCACCTTG
CGAGAGCTAATCGTAATGCCGATGGCTCTTCCACAAGTGAGTGCTGGGGCGCGAGTGG
CGGACCAGATTGTCTTCCGAATGTTCCCCCCCGTGTCGAAGGCTGAACTCGAGGCGGA
GCCGTGAGCGCGCACCGTTGCCACGCAGGTGCACCGCGAGGCACCTCGAAGGTCCCGT
CATGAAG

>DRC_ERR173170.2

GTCGGGATGCCTTCCCAACTCTGGCACGGCGCGGCGCCTTCGCGCACGGATCGGATAA
GCTTTCCCGTGGACGGCTCGTCCTCGGAAATATCTCGTAGTCTTCTCGTTGGTTGCTCCA
ACGAAGCGACGAATCTACTTGGATACTGAACCGCGAGAGCTATTCAAGTGCTCTACTA
CTCGACCGGGGGCAGCATACAGCCGTGGGATCGGCGCGGCCCACCTCCGGCCAACGAG
AGTTACGAGTACCCGGCCCGTTTTCCCGCGTCGGAGGCTGCCAACACGATAGTAACTG
AACTGGCCCAAACGTATTGATACGCCCCGGGGGCGGGCCTCTGGCGCGCCACTGGATC
AGGCCCGTGGCGTGCCCGCCTCGTCAGCGCCACCCATTGCTAAGCGCTGACAGTAATA
GACCCCTCCATAGTAGTTGCCGATGTAGGTCTGGTCACCGGCCGAAACGTATGAACGC
AGCACAGGGCAGGTACTACGGAGCGAAAGGTAGCTGATTGGCAGGGGCTGCTGGCGC
GCCTACCAACCTGTTCCTCCGCGCCTGCTGGAGCGACCAACTACGCCCCGCAGCGACC
GGTACCCGAGCAGTCTCTCAACTGGTTCGATGAGACTGTATACACCGTCGTTGGGACG
CGGACTAAACCGCCCCCTCATACCCACCCGCCCGTCCGGAGCGGAACGACTCGGCGGT
ACCGGCGTCAGGCCCCCTCGCCTAGGCTGCACCATTATGTTGGGAGGTGCGTCGACTG
GGGGCCGGCGACTTGAGCTCGGTCGTCCCGTGAAGCCCATCAGTCCCATGGAGACGTT
CTCTCCCATCCATTGGCCTCCGGGGCTCTCCACCAATCGCACCGGAGTCTTGTCTAGTG
TTAAATGTATTCATCGTGGGGTATAAACCGCCCCCGCGTGAGTGCGGCTCGAAGTCTG
GCCTCCCAGGCTGCTAGCTAACACGGAAGTGTGCCTCGTTTCGCTCAGAGGGGACGAG
ACACTCTCGACGGCTCTCAACAAGCCTAGTCCCCACGATACATACAACCGTGCCCGGT
GGGATTAATACGCCGGGGTCCCGTCGCGGGACATCTGGGATGGGAACACGTTCCGGCC
ATATGCTCTCTGTCCGCATCTCTTGGGGAAATCCATTACGCTGCCGTTGACGTCGTTAA
CGTCACGCCACTAACACCGAACTAGGGGGGCCAAGCTATACCATGTAATCCGTGCGCC
TACCCACTGTTGCGATTCAGGGTACGGGGCTGTGAGTAATGTACACACTCCTGGTGGG
AGCTTGTCCTTCGGACTTGAATGGCTACTCGCCGGGCTGGGACTTAGCAAACCACCCTG
ACGCATTCCAGGCCTCTGATCAGATCACAATTTTGCTTTCACCAGTTCCCAATTACGGT

TCCGCATCGTCGGGAGGCCTTCGACTAGATCTACTCGCATGGTTCGAGCGGTAATATCG
GGTCTGAAATCCCCTGACCCAACGGGAGGCGGTACGTGTGATCGGAATCTGCACCTTG
CGAGAGCTAATCGTAATGACGATTGCTCTTCCACAAGTGAGTGCTGGGGCGCGAGTGG
CGGACCAGATTGTCTTCCGAATGTTCCCCCCCGTGCCGAAGGCTGAACTCGAGGCGGA
GCCGTGGGCGCGCACCGTTGCCACGCAGCTGCACCGCGAGGCACCTCGAAGGTCCCGC
CATGAAG

>DRC_ERR173171.1

GTCGGGATGCTTTCCCAACTCCGGCACGGCGCGGCGCCTTCGCGCACGGATCGGATAA
GCTTTCCCGTGGACGGCTCGTCCTCAGAAACATCTCGTAGTCTTCTCGTTGGTTGCTCC
AACGAAGCGACGAATCTACTTGGATACTGAACCGCGAGAGCTATTCAAGTGCTCTACT
ACTCGACCGGGGGCAGCATACAGCCGTGGGATCGGCGCGGCCCACCTCCGGCCAACGA
GAGTTACGAGTACCCGGCCCGTTTTCCCGCGTCGGAGGCTGCCAACACGATAGTAACT
GAACTGGCCCAAACGTATTGATACGCCCCGGGGGCGGGCCTCTGGCGCGCCACTGGAT
CAGGCCCGTGGCGTGCCCGCCTCGTCAGCGCCACCCATTGCTAAGCGCTGACAGTAAT
AGACCCCTCCATAGTAGTTGCCGATGTTAATTCGGTCACCGGCCGAAACGTATGAACG
CAGCACAGGGCAGGTACTACGGAGCGAAAGGTAGCTGATTGGCAGGGGCTGCTGGCG
CGCCTACCAACCTGTTCCTCCGCGCCTGCTGGAGCGACCAACTACGCCCCGCAGCGAC
CGGTACCCGAGCAGTCTCTCAACTGGTTCGATGAGACTGTATACACCGTCGTTGGGAC
GCGGACTAAACCGCCCCCTCATACCCACCCGCCCGTCCGGAGCGGAACGACTCGGCGG
TACCGGCGTCAGGCCCCCTCGCCTAGGCTGCACCATTATGTTGGGAGGTGCGTCGACTG
GGGGCCGGCGACTTGAGCTCGGTCGTCCCGTGAAGCCCATCAGTCCCATGGAGACGTT
CTCTCCCATCCATTGGCCTCCGGGGCTCTCCACCAATCGCACCGGAGTCTTGTCTAGTG
TTAAATGTATTCATCGTGGGGTATAAACCGCCCCCGCGTGAGTGCGGCTCGAAGTCTG
GCCTCCCAGGCTGCTAGCTAACACGGAAGTGTGCCTCGTTTCGCTCAGAGGGGACGAG
ACACTCTCGACGGCTCTCAACAAGCCTAGTCCCCACGATACATACAACCGTGCCCGGT
GGGATTAATACGCCGGGGTCCCGTCGCGGGACATCTGGGATGGGAACACGTTCCGACC
ATATGCTCTCTGTCCGCATCTCTTGGGGAAATCCATTACGCTGCCGTTGACGTCGTTAA
CGTCACGCCACTAATACCGAACTAGGGGGACCAAACTATACCATGTAATCCGTGCGCC
TACCCACTGTTGCGATTCAGGGTACGGGGCTGTGAGTAATGTACACACTCCTGATGGG
AGCTTGTCCTTCGGACTTGAATGGCTACTCGCCGGGCTGGGACTTAGCAAACCACCCTG
ACGCATTCCAGGCCTCTGATCAGATCACAATTTTGCTTTCACCAGTTCCCAATTACGGT
TCCGCATCGTCGGGAGGCCTTCGACTAGATCTACTCGCATGGTTCGAGCGGTAATATCG
GGTCTGAAATCCCCTGACCCAACGGGAGGCGGTACGTGTGATCGGAATCTGCACCTTG
CGAGAGCTAATCGTAATGACGGTGGCTCTTCCACAAGTGAGTGCTGGGGCGCGAGTGG
CGGACCAGATTGTCTTCCGAATGTTCCCCCCCGTGCCGAAGGCTGAACTCGAGGCGGA
GCCGTGGGCGCGCACCGTTGCCACGCAGCTGCACCGCGAGGCACCTCGAAGGTCCCGC
CATGAAG

>DRC_ERR173171.2

GTCGGGATGCCTTCCCAACTCTGGCACGGCGCGGCGCCTTCGCGCACGGATCGGATAA
GCTTTCCCGTGGACGGCTCGTCCTCGGAAATATCTCGTAGTCTTCTCGTTGGTTGCTCCA
ACGAAGCGACGAATCTACTTGGATACTGAACCGCGAGAGCTATTCAAGTGCTCTACTA
CTCGACCGGGGGCAGCATACAGCCGTGGGATCGGCGCGGCCCACCTCCGGCCAACGAG
AGTTACGAGTACCCGGCCCGTTTTCCCGCGTCGGAGGCTGCCAACACGATAGTAACTG
AACTGGCCCAAACGTATTGATACGCCCCGGGGGCGGGCCTCTGGCGCGCCACTGGATC
AGGCCCGTGGCGTGCCCGCCTCGTCAGCGCCACCCATTGCTAAGCGCTGACAGTAATA
GACCCCTCCATAGTAGTTGCCGATGTTAATTCGGTCACCGGCCGAAACGTATGAACGC
AGCACAGGGCAGGTACTACGGAGCGAAAGGTAGCTGATTGGCAGGGGCTGCTGGCGC
GCCTACCAACCTGTTCCTCCGCGCCTGCTGGAGCGACCAACTATGCCCCGCAGCGACC

GGTACTTGAGCTGTCTCTCAACTGGTTCGATGAGACTGTATACACCGTCGTTGGGACGC
GGACTAAACAACCTCCTCATACCAATCCGCCCGTCCGGAGCGGAACGACTCGGCGGTA
CCGGCGTCAGGCCCCCTCGCCTAGGCTGCACCATTATGTTGGGAGGTGCGTCGACTGG
GGGCCGGCGACTTGAGCTCGGTCGTCCCGTGAAGCCCATCAGTCCCATGGAGACGTTC
TCTCCCATCCATTGGCCTCCGGGGCTCTCCACCAATCGCACCGGAGTCTTGTCTAGTGT
TAAATGTATTCATCGTGGGGTATAAACCGCCCCCGCGTGAGTGCGGCTCGAAGTCTGG
CCTCCCAGGCTGCTAGCTAACACGGAAGTGTGCCTCGTTTCGCTCAGAGGGGACGAGA
CACTCTCGACGGCTCTCAACAAGCCTAGTCCCCACGATACATACAACCGTGCCCGGTG
GGATTAATACGCCGGGGTCCCGTCGCGGGACATCTGGGATGGGAACACGTTCCGGCCA
TATGCTCTCTGTCCGCATCTCTTGGGGAAATCCATTACGCTGCCGTTGACGTCGTTAAC
GTCACGCCACTAATACCGAACTAGGGGGACCAAACTATACCATGTAATCCGTGCGCCT
ACCCACTGTTGCGATTCAGGGTACGGGGCTGTGAGTAATGTACACACTCCTGGTGGGA
GCTTGTCCTTCGGACTTGAATGGCTACTCGCCGGGCTGGGACTTAGCAAACCACCCTGA
CGCATTCCAGGCCTCTGATCAGATCACAATTTTGCTTTCACCAGTTCCCAATTACGGTT
CCGCATCGTCGGGAGGCCTTCGACTAGACCTACTCGCATGGTTCGAGCGGTAATATCG
GGTCTGAAATCCCCTGACCCAACGGGAGGCGGTACGTGTGATCGGAATCTGCACCTTG
CGAGAGCTAATCGTAATGACGATTGCTCTTCCACAAGTGAGTGCTGGGGCGCGAGTGG
CGGACCAGATTGTCTTCCGAATGTTCCCCCCCGTGCCGAAGGCTGAACTCGAGGCGGA
GCCGTGGGCGCGCACCGTTGCCACGCAGCTGCACCGCGAGGCACCTCGAAGGTCCCGC
CATGAAG

>DRC_ERR173172.1

GTCGGGATGCCTTCCCAACTCTGGCACGGCGCGGCGCCTTCGCGCACGGATCGGATAA
GCTTTCCCGTGGACGGCTCGTCCTCGGAAATATCTCGTAGTCTTCTCGTTGGTTGCTCCA
ACGAAGCGACGAATCTACTTGGATACTGAACCGCGAGAGCTATTCAAGTGCTCTACTA
CTCGACCGGGGGCAGCATACAGCCGTGGGATCGGCGCGGCCCACCTCCGGCCAACGAG
AGTTACGAGTACCCGGCCCGTTTTCCCGCGTCGGAGGCTGCCAACACGATAGTAACTG
AACTGGCCCAAACGTATTGATACGCCCCGGGGGCGGGCCTCTGGCGCGCCACTGGATC
AGGCCCGTGGCGTGCCCGCCTCGTCAGCGCCACCCATTGCTAAGCGCTGACAGTAATA
GACCCCTCCATAGTAGTTGCCGATGTAGGTCTGGTCACCGGCCGAAACGTATGAACGC
AGCACAGGGCAGGTACTACGGAGCGAAAGGTAGCTGATTGGCAGGGGCTGCTGGCGC
GCCTACCAACCTGTTCCTCCGCGCCTGCTGGAGCGACCAACTACGCCCCGCAGCGACC
GGTACCCGAGCAGTCTCTCAACTGGTTCGATGAGACTGTATACACCGTCGTTGGGACG
CGGACTAAACCGCCCCCTCATACCCACCCGCCCGTCCGGAGCGGAACGACTCGGCGGT
ACCGGCGTCAGGCCCCCTCGCCTAGGCTGCACCATTATGTTGGGAGGTGCGTCGACTG
GGGGCCGGCGACTTGAGCTCGGTCGTCCCGTGAAGCCCATCAGTCCCATGGAGACGTT
CTCTCCCATCCATTGGCCTCCGGGGCTCTCCACCAATCGCACCGGAGTCTTGTCTAGTG
TTAAATGTATTCATCGTGGGGTATAAACCGCCCCCGCGTGAGTGCGGCTCGAAGTCTG
GCCTCCCAGGCTGCTAGCTAACACGGAAGTGTGCCTCGTTTCGCTCAGAGGGGACGAG
ACACTCTCGACGGCTCTCAACAAGCCTAGTCCCCACGATACATACAACCGTGCCCGGT
GGGATTAATACGCCGGGGTCCCGTCGCGGGACATCTGGGATGGGAACACGTTCCGGCC
ATATGCTCTCTGTCCGCATCTCTTGGGGAAATCCATTACGCTGCCGTTGACGTCGTTAA
CGTCACGCCACTAATACCGAACTAGGGGGACCAAACTATACCATGTAATCCGTGCGCC
TACCCACTGTTGCGATTCAGGGTACGGGGCTGTGAGTAATGTACACACTCCTGGTGGG
AGCTTGTCCTTCGGACTTGAATGGCTACTCGCCGGGCTGGGACTTAGCAAACCACCCTG
ACGCATTCCAGGCCTCTGATCAGATCACAATTTTGCTTTCACCAGTTCCCAATTACGGT
TCCGCATCGTCGGGAGGCCTTCGACTAGATCTACTCGCATGGTTCGAGCGGTAATATCG
GGTCTGAAATCCCCTGACCCAACGGGAGGCGGTACGTGTGATCGGAATCTGCACCTTG
CGAGAGCTAATCGTAATGACGATTGCTCTTCCACAAGTGAGTGCTGGGGCGCGAGTGG
CGGACCAGATTGTCTTCCGAATGTTCCCCCCCGTGCCGAAGGCTGAACTCGAGGCGGA

GCCGTGGGCGCGCACCGTTGCCACGCAGGTGCACCGCGAGGCACCTCGAAGGTCCCGT
CATGAAG

>DRC_ERR173172.2

GTCGGGATGCCTTCCCAACTCTGGCACGGCGCGGCGCCTTCGCGCACGGATCGGATAA
GCTTTCCCGTGGACGGCTCGTCCTCGGAAATATCTCGTAGTCTTCTCGTTGGTTGCTCCA
ACGAAGCGACGAATCTACTTGGATACTGAACCGCGAGAGCTATTCAAGTGCTCTACTA
CTCGACCGGGGGCAGCATACAGCCGTGGGATCGGCGCGGCCCACCTCCGGCCAACGAG
AGTTACGAGTACCCGGCCCGTTTTCCCGCGTCGGAGGCTGCCAACACGATAGTAACTG
AACTGGCCCAAACGTATTGATACGCCCCGGGGGCGGGCCTCTGGCGCGCCACTGGATC
AGGCCCGTGGCGTGCCCGCCTCGTCAGCGCCACCCATTGCTAAGCGCTGACAGTAATA
GACCCCTCCATAGTAGTTGCCGATGTAGGTCTGGTCACCGGCCGAAACGTATGAACGC
AGCACAGGGCAGGTACTACGGAGCGAAAGGTAGCTGATTGGCAGGGGCTGCTGGCGC
GCCTACCAACCTGTTCCTCCGCGCCTGCTGGAGCGACCAACTACGCCCCGCAGCGACC
GGTACCCGAGCAGTCTCTCAACTGGTTCGATGAGACTGTATACACCGTCGTTGGGACG
CGGACTAAACCGCCCCCTCATACCCACCCGCCCGTCCGGAGCGGAACGACTCGGCGGT
ACCGGCGTCAGGCCCCCTCGCCTAGGCTGCACCATTATGTTGGGAGGTGCGTCGACTG
GGGGCCGGCGACTTGAGCTCGGTCGTCCCGTGAAGCCCATCAGTCCCATGGAGACGTT
CTCTCCCATCCATTGGCCTCCGGGGCTCTCCACCAATCGCACCGGAGTCTTGTCTAGTG
TTAAATGTATTCATCGTGGGGTATAAACCGCCCCCGCGTGAGTGCGGCTCGAAGTCTG
GCCTCCCAGGCTGCTAGCTAACACGGAAGTGTGCCTCGTTTCGCTCAGAGGGGACGAG
ACACTCTCGACGGCTCTCAACAAGCCTAGTCCCCACGATACATACAACCGTGCCCGGT
GGGATTAATACGCCGGGGTCCCGTCGCGGGACATCTGGGATGGGAACACGTTCCGGCC
ATATGCTCTCTGTCCGCATCTCTTGGGGAAATCCATTACGCTGCCGTTGACGTCGTTAA
CGTCACGCCACTAATACCGAACTAGGGGGACCAAACTATACCATGTAATCCGTGCGCC
TACCCACTGTTGCGATTCAGGGTACGGGGCTGTGAGTAATGTACACACTCCTGGTGGG
AGCTTGTCCTTCGGACTTGAATGGCTACTCGCCGGGCTGGGACTTAGCAAACCACCCTG
ACGCATTCCAGGCCTCTGATCAGATCACAATTTTGCTTTCACCAGTTCCCAATTACGGT
TCCGCATCGTCGGGAGGCCTTCGACTAGATCTACTCGCATGGTTCGAGCGGTAATATCG
GGTCTGAAATCCCCTGACCCAACGGGAGGCGGTACGTGTGATCGGAATCTGCACCTTG
CGAGAGCTAATCGTAATGACGATTGCTCTTCCACAAGTGAGTGCTGGGGCGCGAGTGG
CGGACCAGATTGTCTTCCGAATGTTCCCCCCCGTGCCGAAGGCTGAACTCGAGGCGGA
GCCGTGGGCGCGCACCGTTGCCACGCAGGTGCACCGCGAGGCACCTCGAAGGTCCCGT
CATGAAG

>DRC_ERR173173.1

GTCGGGATGCCTTCCCAACTCTGGCACGGCGCGGCGCCTTCGCGCACGGATCGGATAA
GCTTTCCCGTGGACGGCTCGTCCTCGGAAATATCTCGTAGTCTTCTCGTTGGTTGCTCCA
ACGAAGCGACGAATCTACTTGGATACTGAACCGCGAGAGCTATTCAAGTGCTCTACTA
CTCGACCGGGGGCAACATACAGCCGTGGGATCGGCGCGGCCCACCTCCGGCCAACGAG
AGTTACGAGTACCCGGCCCGTTTTCCCGCGTCGGAGGCTGCCAACACGATAGTAACTG
AACTGGCCCAAACGTATTGATACGCCCCGGGGGCGGGCCTCTGGCGCGCCACTGGATC
AGGCCCGTGGCGTGCCCGCCTCGTCAGCGCCACCCATTGCTAAGCGCTGACAGTAATA
GACCCCTCCATAGTAGTTGCCGATGTAGGTCTGGTCACCGGCCGAAACGTATGAACGC
AGCACAGGGCAGGTACTACGGAGCGAAAGGTAGCTGATTGGCAGGGGCTGCTGGCGC
GCCTACCAACCTGTTCCTCCGCGCCTGCTGGAGCGACCAACTACGCCCCGCAGCGACC
GGTACCCGAGCAGTCTCTCAACTGGTTCGATGAGACTGTATACACCGTCGTTGGGACG
CGGACTAAACCGCCCCCTCATACCAATCCGCCCGTCCGGAGCGGAACGACTCGGCGGT
ACCGGCGTCAGGCCCCCTCGCCTAGGCTGCACCATTATGTTGGGAGGTGCGTCGACTG
GGGGCCGGCGACTTGAGCTCGGTCGTCCCGTGAAGCCCATCAGTCCCATGGAGACGTT

CTCTCCCATCCATTGGCCTCCGGGGCTCTCCACCAATCGCACCGGAGTCTTGTCTAGTG
TTAAATGTATTCATCGTGGGGTATAAACCGCCCCCGCGTGAGTGCGGCTCGAAGTCTG
GCCTCCCAGGCTGCTAGCTAACACGGAAGTGTGCCTCGTTTCGCTCAGAGGGGACGAG
ACACTCTCGACGGCTCTCAACAAGCCTAGTCCCCACGATACATACAACCGTGCCCGGT
GGGATTAATACGCCGGGGTCCCGTCGCGGGACATCTGGGATGGGAACACGTTCCGGCC
ATATGCTCTCTGTCCGCATCTCTTGGGGAAATCCATTACGCTGCCGTTGACGTCGTTAA
CGTCACGCCACTAATACCGAACTAGGGGGACCAAACTATACCATGTAATCCGTGCGCC
TACCCACTGTTGCGATTCAGGGTACGGGGCTGTGAGTAATGTACACACTCCTGGTGGG
AGCTTGTCCTTCGGACTTGAATGGCTACTCGCCGGGCTGGGACTTAGCAAACCACCCTG
ACGCATTCCAGGCCTCTGATCAGATCACAATTTTGCTTTCACCAGTTCCCAATTACGGT
TCCGCATCGTCGGGAGGCCTTCGACTAGATCTACTCGCATGGTTCGAGCGGTAATATCG
GGTCTGAAATCCCCTGACCCAACGGGAGGCGGTACGTGTGATCGGAATCTGCACCTTG
CGAGAGCTAATCGTAATGACGATTGCTCTTCCACAAGTGAGTGCTGGGGCGCGAGTGG
CGGACCAGATTGTCTTCCGAATGTTCCCCCCCGTGCCGAAGGCTGAACTCGAGGCGGA
GCCGTGGGCGCGCACCGTTGCCACGCAGCTGCACCGCGAGGCCCCTCGAAGGTCCCGC
CATGAAG

>DRC_ERR173173.2

GTCGGGATGCCTTCCCAACTCTGGCACGGCGCGGCGCCTTCGCGCACGGATCGGATAA
GCTTTCCCGTGGACGGCTCGTCCTCGGAAATATCTCGTAGTCTTCTCGTTGGTTGCTCCA
ACGAAGCGACGAATCTACTTGGATACTGAACCGCGAGAGCTATTCAAGTGCTCTACTA
CTCGACCGGGGGCAGCATACAGCCGTGGGATCGGCGCGGCCCACCTCCGGCCAACGAG
AGTTACGAGTACCCGGCCCGTTTTCCCGCGTCGGAGGCTGCCAACACGATAGTAACTG
AACTGGCCCAAACGTATTGATACGCCCCGGGGGCGGGCCTCTGGCGCGCCACTGGATC
AGGCCCGTGGCGTGCCCGCCTCGTCAGCGCCACCCATTGCTAAGCGCTGACAGTAATA
GACCCCTCCATAGTAGTTGCCGATGTTAATTCGGTCACCGGCCGAAACGTATGAACGC
AGCACAGGGCAGGTACTACGGAGCGAAAGGTAGCTGATTGGCAGGGGCTGCTGGCGC
GCCTACCAACCTGTTCCTCCGCGCCTGCTGGAGCGACCAACTACGCCCCGCAGCGACC
GGTACTTGAGCTGTCTCTCAACTGGTTCGATGAGACTGTATACACCGTCGTTGGGACGC
GGACTAAACAACCTCCTCATACCAATCCGCCCGTCCGGAGCGGAACGACTCGGCGGTA
CCGGCGTCAGGCCCCCTCGCCTAGGCTGCACCATTATGTTGGGAGGTGCGTCGACTGG
GGGCCGGCGACTTGAGCTCGGTCGTCCCGTGAAGCCCATCAGTCCCATGGAGACGTTC
TCTCCCATCCATTAGCCTCCGGGGCTCTCCACCAATCGCACCGGAGTCTTGTCTAGTGT
TAAATGTATTCATCGTGGGGTATAAACCGCCCCCGCGTGGGTGCGGCTCGAAGTCTGG
CCTCCCAGGCTGCTAGCTAACACGGAAGTGTGCCTCGTTTCGCTCAGAGGGGACGAGA
CACTCTCGACGGCTCTCAACAAGCCTAGTCCCCACGATACATACAACCGTGCCCGGTG
GGATTAATACACCGGGGTCCCGTCGCGGGACGTCTGGGATGGGACCATGTTCCGGCCA
TATACTCCCTGTTCGTGCCCCTCGGAGAAATCCATTACGCTGCCGTTGACGTCGTTAAC
GTCACGCCACTAACACCGAACTAGGGGGACCAAACTATACCATGTAATCCGTGCGCCT
ACCCACTGTTGCGATTCAGGGTACGGGGCTGTGAGTAATGTACACACTCCTGGTGTGA
GCTTGTCCTCCGGACTTGAATGGCTACTCGCCGGGCTGGGACTTAGCAAACCACCCTGA
CGCATTCCAGGCCTCTGATCAGATCACAATTTTGCTTTCACCAGTTCCCAATTACGGTT
CCGCATCGTCGGGAGGCCTTCGACTAGATCTACTCGCATGGTTCGAGCGGTAATATCG
GGTCTGAAATCCCCTGACCCAACGGGAGGCGGTACGTGTGATCAGGATCTGCACCTTG
CGAGAGCCAAGCGTAATGACGGTGGCTCTTCCACAAGTAAAAGCTGGGGCGCGAGCG
GCGGGCTGGATGGTCCCCCGAATGTTCCCCCCTATGCCGAAGGCTGAACTCGAGGCTG
AGCCGTGGGCGCGCACCGTTGCCACGCAGGTGCACCGCGAGGTCCCTTGAAGGTCCCG
TCACGAAG

>HAMS_ERR173174.1

GTCGGGATGCCTTCCCAACTCTGGCACGGCGCGGCGCCTTCGCGCACGGATCGGATAA
GCTTTCCCGTGGACGGCTCGTCCTTGGAAATATCTCGTAGTCTTCTCGTTGGTTGCTCCA
ACGAAGCGACGAATCTACTTGGATACTGAACCGCGAGAGCTATTCAAGTGCTCTACTA
CTCGACCGGGGGCAGCATACAGCCGTGGGATCGGCGCGGCCCACCTCCGGCCAACGAG
AGTTACGAGTACCCGACGCGTTTTCCCGCGTCGGAGGCTGCCAACACGATAGTAACTG
AACTGGCCCAAACGTATTGATACGCCCCGGGGGCGGGCCTCTGGCGCGCCACTGGATC
AGGCCCGTGGCGTGCCCGCCTCGTCAGCGCCACCCATTGCTAAGCGCTGACAGTAATA
GACCCCTCCATAGTAGTTGCCGATGTTAATTCGGTCACCGGCCGAAACGTATGCACTTA
GCACAGGGCAGGTACTACAAAGCGAGAGGTGGACGATTGGCCAAGGCTGCTGGCGAG
CCTACCAACCTGTTCCTCCGCGCCTGCTGGAGCGACCAACTACGCCCCGCAGCGACCG
GTACTTGAGCTGTCTCTCAACTGGTTCGATGAGACTGTATACACCGTCGTTGGGACGCG
GACTAAACCGCCCCCTCATACCCACCCGCCCGTCCGGAACGGAACGACTCGGCGGTAC
CGGCGTCAGGCCCCCTCGCCTAGGCTGCACCATTATGTTGGGAGGTGCGTCGACTGGG
GGCCGGCGACTTGAGCTCGGTCGTCCCGTGAAGCCCATCAGTCCCATGGAGACGTTCT
CTCCCATCCATTGGCCTCCGGGGCTCTCCACCAATCGCACCGGAGTCTTGTCTAGTGTT
AAATGTATTCATCGTGGGGTATAAACCGCCCCCGCGTAAGTGCGGCTCGAAGTCTGGC
CTCCCAGGCTGCTAGCAAACACGGAAGTGTGCCTCGTTTCGCTCAGAGGGGACAAGAC
ACTCTCGACGGCTCTCAACAAGCCTAGTCCCCACGATACATACAACCGTGCCCGGTGG
GATTAATACGCCGGGGTCCCGTCGCGGGACATCTGGGATGGGAACACGTTCCGACCAT
ATGCTCTCTGTCTGCATCTCTTGGGGAAATCCATTACGCTGCCGTTGACGTCGTTAACG
TCACGCCACTAATACCGAACTAGGGGGACCAAACTATACCATGTTATCCGTGCGCCTA
CCCACTGTTGCGATTCAGGGTACGGGGCTGTGAGTAATGTACACACTCCTGGTGGGAG
CTTGTCCTCCGGACTTGAATGGCTACTCGCCGGGCTGGGACTTAGCAAACCACCCTGAC
GCATTCCAGGCCTCTGATCAGATCACAATTTTGCTTTCACCAGTTCCCAATTACGGTTC
CGCATCGTCGGGAGGCCTTCGACTAAATCTACTCGCATGGTTCGAGCGGTAATATCGG
GTCTGAAATCCCCTGACCCAACGGGAGGCGGTACGTGTGATCAGGATCTGCACCTTGC
GAGAGCCAAGCGTAATGACGGTGGCTCTTCCACAAGTAAAAGCTCGGGCGCGAGCGG
CGGGCTGGATGGTCCTCCGAATGTTCCCCCCTATGCCGAAGGCTGAACTCGAGGCTGA
GCCGTGGGCGCGCACCGTTGCCACGCAGGTGCACCGCGAGGTCCCTTGAAGGTCCCGT
CACGAAG

>HAMS_ERR173174.2

GTCGGGATGCCTTCCCAACTCTGGCACGGCGCGGCGCCTTCGCGCACGGATCGGATAA
GCTTTCCCGTGGACGGCTCGTCCTCGGAAATATCTCGTAGTCTTCTCGTTGGTTGCTCCA
ACGAAGCGACGAATCTACTTGGATACTGAACCGCGAGAGCTATTCAAGTGCTCTACTA
CTCGACCGGGGGCAGCATACAGCCGTGGGATCGGCGCGGCCCACCTCCGGCCAACGAG
AGTTACGAGTACCCGGCCCGTTTTCCCGCGTCGGAGGCTGCCAACACGATAGTAACTG
AACTGGCCCAAACGTATTGATACGCCCCGGGGGCGGGCCTCTGGCGCGCCACTGGATC
AGGCCCGTGGCGTGCCCGCCTCGTCAGCGCCACCCGTTGCTAAGCGCTGACAGTAATA
GACCCCTCCATAGTAGTTGCCGATGTAGGTCTGGTCACCGGCCGAAACGTATGAACGC
AGCACAGGGCAGGTACTACGGAGCGAAAGGTAGCTGATTGGCAGGGGCTGCTGGCGC
GCCTACCAACCTGTTCCTCCGCGCCTTCTGGAGCGACCAACTACGCCCCGCAGCGACCG
GTACCCGAGCAGTCTCTCAACTGGTTCGATGAGACTGTATACACCGTCGTTGGGACGC
GGACTAAACCGCCCCCTCATACCCACCCGCCCGTCCGGAGCGGAACGACTCGGCGGTA
CCGGCGTCAGGCCCCCTCGCCTAGGCTGCACCATTATGTTGGGAGGTGCGTCGACTGG
GGGCCGGCGACTTGAGCTCGGTCGTCCCGTGAAGCCCATCAGTCCCATGGAGACGTTC
TCTCCCATCCATTGGCCTCCGGGGCTCTCCACCAATCGCACCGGAGTCTTGTCTAGTGT
TAAATGTATTCATCGTGGGGTATAAACCGCCCCCGCGTAAGTGCGGCTCGAAGTCTGG
CCTCCCAGGCTGCTAGCAAACACGGAAGTGTGCCTCGTTTCGCTCAGAGGGGACAAGA
CACTCTCGACGGCTCTCAACAAGCCTAGTCCCCACGATACATACAACCGTGCCCGGTG
GGATTAATACGCCGGGGTCCCGTCGCGGGACATCTGGGATGGGAACACGTTCCGACCA

TATGCTCTCTGTCTGCATCTCTTGGGGAAATCCATTACGCTGCCGTTGACGTCGTTAAC
GTCACGCCACTAATACCGAACTAGGGGGGCCAAGCTATACCATGTAATCCGTGCGCCT
ACCCACTGTTGCGATTCAGGGTACGGGGCTGTGAGTAATGTACACACTCCTGGTGTGA
GCTTGTCCTCCGGACTTGAATGGCTACTCGCCGGGCTGGGACTTAGCAAACCACCCTGA
CGCATTCCAGGCCTCTGATCAGATCACAATTTTGCTTTCACCAGTTCCCAATTACGGTT
CCGCATCGTCGGGAGGCCTTCGACTAGATCTACTCGCATGGTTCGAGCGGTAATATCG
GGTCTGAAATCCCCTGACCCAACGGGAGGCGGTACGTGTGATCAGGATCTGCACCTTG
CGAGAGCCAAGCGTAATGACGGTGGCTCTTCCACAAGTAAAAGCTCGGGCGCGAGCG
GCGGGCTGGATGGTCCCCCGAATGTTCCCCCCTATGCCGAAGGCTGAACTCGAGGCTG
AGCCGTGGGCGCGCACCGTTGCCACGCAGGTGCACCGCGAGGTCCCTTGAAGGTCCCG
TCACGAAG

>HAMS_ERR173175.1

GTCGGGATGCCTTCCCAACTCTGGCACGGCGCGGCGCCTTCGCGCACGGATCGGATAA
GCTTTCCCGTGGACGGCTCGTCCTTGGAAATATCTCGTAGTCTTCTCGTTGGTTGCTCCA
ACGAAGCGACGAATCTACTTGGATACTGAACCGCGAGAGCTATTCAAGTGCTCTACTA
CTCGACCGGGGGCGGCATACAGCCGTGGGATCGGCGCGGCCCACCTCCGGCCAACGAG
AGTTACGAGTACCCGACGCGTTTTCCCGCGTCGGAGGCTGCCAACACGATAGTAACTG
AACTGGCCCAAACGTATTGATACGCCCCGGGGGCGGGCCTCTGGCGCGCCACTGGATC
AGGCCCGTGGCGTGCCCGCCTCGTCAGCGCCACCCATTGCTAAGCGCTGACAGTAATA
GACCCCTCCATAGTAGTTGCCGATGTTAATTCGGTCACCGGCCGAAACGTATGCACTTA
GCATAGGGCAGGTACTACAAAGCGAGAGGTGGACGATTGGCAAAGGCTGCTGGCGAG
CCTACCAACCTGTTCCTCCGCGCCTGCTGGAGCGACCAACTACGCCCCGCAGCGACCG
GTACTTGAGCTGTCTCTCAACTGGTTCGATGAGACTGTATACACCGTCGTTGGGACGCG
GACTAAACCGCCCCCTCATACCCACCCGCCCGTCCGGAACGGAACGACTCGGCGGTAC
CGGCGTCAGGCCCCCTCGCCTAGGCTGCACCATTATGTTGGGAGGTGCGTCGACTGGG
GGCCGGCGACTTGAGCTCGGTCGTCCCGTGAAGCCCATCAGTCCCATGGAGACGTTCT
CTCCCATCCATTGGCCTCCGGGGCTCTCCACCAATCGCACCGGAGTCTTGTCTAGTGTT
AAATGTATTCATCGTGGGGTATAAACCGCCCCCGCGTAAGTGCGGCTCGAAGTCTGGC
CTCCCAGGCTGCTAGCAAACACGGAAGTGTGCCTCGTTTCGCTCAGAGGGGACAAGAC
ACTCTCGACGGCTCTCAACAAGCCTAGTCCCCACGATACATACAACCGTGCCCGGTGG
GATTAATACGCCGGGGTCCCGTCGCGGGACATCTGGGATGGGAACACGTTCCGACCAT
ATGCTCTCTGTCTGCATCTCTTGGGGAAATCCATTACGCTGCCGTTGACGTCGTTAACG
TCACGCCACTAATACCGAACTAGGGGGACCAAACTATACCATGTTATCCGTGCGCCTA
CCCACTGTTGCGATTCAGGGTACGGGGCTGTGAGTAATGTACACACTCCTGGTGGGAG
CTTGTCCTCCGGACTTGAATGGCTACTCGCCGGGCTGGGACTTAGCAAACCACCCTGAC
GCATTCCAGGCCTCTGATCAGATCACAATTTTGCTTTCACCAGTTCCCAATTACGGTTC
CGCATCGTCGGGAGGCCTTCGACTAAATCTACTCGCATGGTTCGAGCGGTAATATCGG
GTCTGAAATCCCCTGACCCAACGGGAGGCGGTACGTGTGATCAGGATCTGCACCTTGC
GAGAGCCAAGCGTAATGACGGTGGCTCTTCCACAAGTAAAAGCTCGGGCGCGAGCGG
CGGGCTGGATGGTCCTCCGAATGTTCCCCCCTATGCCGAAGGCTGAACTCGAGGCTGA
GCCGTGGGCGCGCACCGTTGCCACGCAGGTGCACCGCGAGGTCCCTTGAAGGTCCCGT
CACGAAG

>HAMS_ERR173175.2

GTCGGGATGCCTTCCCAACTCTGGCACGGCGCGGCGCCTTCGCGCACGGATCGGATAA
GCTTTCCCGTGGACGGCTCGTCCTCGGAAATATCTCGTAGTCTTCTCGTTGGTTGCTCCA
ACGAAGCGACGAATCTACTTGGATACTGAACCGCGAGAGCTATTCAAGTGCTCTACTA
CTCGACCGGGGGCAGCATACAGCCGTGGGATCGGCGCGGCCCACCTCCGGCCAACGAG
AGTTACGAGTACCCGGCCCGTTTTCCCGCGTCGGAGGCTGCCAACACGATAGTAACTG

AACTGGCCCAAACGTATTGATACGCCCCGGGGGCGGGCCTCTGGCGCGCCACTGGATC
AGGCCCGTGGCGTGCCCGCCTCGTCAGCGCCACCCATTGCTAAGCGCTGACAGTAATA
GACCCCTCCATAGTAGTTGCCGATGTAGGTTTGGTCACCGGCCGAAACGTATGAACGC
AGCACAGGGCAGGTACTACGGAGCGAAAGGTAGCTGATTGGCAGGGGCTGCTGGCGC
GCCTACCAACCTGTTCCTCCGCGCCTGCTGGAGCGACCAACTACGCCCCGCAGCGACC
GGTACCCGAGCAGTCTCTCAACTGGTTCGATGAGACTGTATACACCGTCGTTGGGACG
CGGACTAAACCGCCCCCTCATACCCACCCGCCCGTCCGGAGCGGAACGACTCGGCGGT
ACCGGCGTCAGGCCCCCTCGCCTAGGCTGCACCATTATGTTGGGAGGTGCGTCGACTG
GGGGCCGGCGACTTGAGCTCGGTCGTCCCGTGAAGCCCATCAGTCCCATGGAGACGTT
CTCTCCCATCCATTGGCCTCCGGGGCTCTCCACCAATCGCACCGGAGTCTTGTCTAGTG
TTAAATGTATTCATCGTGGGGTATAAACCGCCCCCGCGTAAGTGCGGCTCGAAGTCTG
GCCTCCCAGGCTGCTAGCAAACACGGAAGTGTGCCTCGTTTCGCTCAGAGGGGACAAG
ACACTCTCGACGGCTCTCAACAAGCCTAGTCCCCACGATACATACAACCGTGCCCGGT
GGGATTAATACGCCGGGGTCCCGTCGCGGGACATCTGGGATGGGAACACGTTCCGACC
ATATGCTCTCTGTCTGCATCTCTTGGGGAAATCCATTACGCTGCCGTTGACGTCGTTAA
CGTCACGCCACTAATACCGAACTAGGGGGGCCAAGCTATACCATGTAATCCGTGCGCC
TACCCACTGTTGCGATTCAGGGTACGGGGCTGTGAGTAATGTACACACTCCTGGTGTGA
GCTTGTCCTCCGGACTTGAATGGCTACTCGCCGGGCTGGGACTTAGCAAACCACCCTGA
CGCATTCCAGGCCTCTGATCAGATCACAATTTTGCTTTCACCAGTTCCCAATTACGGTT
CCGCATCGTCGGGAGGCCTTCGACTAGATCTACTCGCATGGTTCGAGCGGTAATATCG
GGTCTGAAATCCCCTGACCCAACGGGAGGCGGTACGTGTGATCAGGATCTGCACCTTG
CGAGAGCCAAGCGTAATGACGGTGGCTCTTCCACAAGTAAAAGCTCGGGCGCGAGCG
GCGGGCTGGATGGTCCCCCGAATGTTCCCCCCTATGCCGAAGGCTGAACTCGAGGCTG
AGCCGTGGGCGCGCACCGTTGCCACGCAGGTGCACCGCGAGGTCCCTTGAAGGTCCCG
TCACGAAG

>AWB_ERR173176.1

GTCGGGATGCCCTTTCGACACTGGCACGGCGCGGCGCCTTCGCGCACGGATCGGATAA
ACTTTCCCGTGGACGGCTCGTCCTCAGAAACATTTCGTAGTCTTCTCGTTGGTTACTCCA
CTGCCGCGCCGCGTCTGCTTGGATGCTGAACCACGAGATTTATTAGAGTACTCTACTAC
TCGACCGGGGGCAGTATACAACCGTGGAAACGACGCGGCCCACCTCCGGTCAACGAG
AGTTACGAGTACCCGGCCCGTTTTCCCGTGTCGGAGGCTGCCAACACGATAGTAACTG
AACTGGCCCAAACGTATTAATATGCCCCGGGGGCGGGCCTCTGGCGCGCCACTGGATC
AGGCCCGTGGCGTGCCCGCCTCGTCAGCGCCACCCATTGCTAAGCGCTGACAGTAATA
GACCCCTCCATAGTAGTTGCCGATGTTGATTCGGTCACCGGCCGAAACGTATGCACTCA
GCACAGGGCAGGTACTACGGAGCGAAAGGTGGATAATTGGCCGGGGCTGCTGGCGCG
CCTACCAACCTGTTCCCCCGCGCCTGCTGGAGCGACTAACTACGCCCTGTGGCGACCGA
TACTTGAGCTGTCTCTCAACTGGGTCAATTAGATTGTATACATCCTTGTTGGGTCGCTG
CCTAAACAACCTCCTCATACCCATCCGCCCGTCTGGAGCGGAACGACTCGGCGGTACC
GGCGTCAGGCCCCCTCGCCTAGGCTGCGCCATTATGTTGGGAGGTGCGTCGACTGGGG
GCCGGCAACCTGTGGTCGGCCGTTTCGTGAAGCCCATCACTCCCATGGAGACGTTTTCT
CCCATCCATTGGCCCCCGGGGTCCTCCACCGATTGCGTCGGAGTCTTGTCTAATGTGAA
ATTTATTCATCGTGAGGTATAAACCGCCCCCGCGTGGGTGCGGCTCGAAGTCCGGCCTC
CCAAGCTGCCTGCTAACACGGAAGTGTACCCGGTTCCACTCAGAGGGCATGAGGCAGT
ACCTACGGATCTCAACAAGCCTAGTCCCCATGATACGTACAGCTGGGCCAGGTGGGAT
TAATACGTCGGGGTCCCGTCGCGGGATGTTTGAGGGGGAGATACGTTCCGGCCATATA
CTCTTTGTCCGCATCCCTTGGGGAAATCCATTACGCTGCCGTTGACGTCGTTAACGTCA
CGCCACTAACACCGAACTAGGGGGACCAAACTATACCATGTTATCCGTATGCCTACTC
GTTGTTGCGGTTCAGAGTACGGGGCTGTGAGTAATGTACGCGCTCCTGGTGGGAACTT
GTCCTCCGGACTTGAATAACAGCTCACTGGGATGGGATTTCGCAAACCATCCCAACAT
ATTCCAGGCCTCTGATCAGATCATAATTTTGCTGTCACCAGTTCCCAATTACGGTTCTG

CATCGTCGGGAGGCCTTCGACTAGATCTACTCGCATGGTTCGAGCGGTAATATCGGGTC
TGAAATCCCCTGACCCAACGGGAGGCGGTATGTGTGATCGGGATCTGCACCTTGCGAG
AGCTAAGCGTAATGACGATGGCTCTTCCACAAGTGAGTGCTGGGGCGCGAGCAGCGGA
CCAGATTGTCTTCCAAATGTTCCCCCCCGTGCCGAAGGCTGAACTCAAGGCGGGGCCG
TGGGCGCGCACCGTTGACACGCAGGTGCACCGCGAGGCACCTTGAAGGTCCCGTCATG
AAG

>AWB_ERR173176.2

GTCGGGATGCCTCCTCGGCTCTGGCACGGCGCGGTGCCTTCGTGCACGGATCGGCCAA
GCTTTCCCGTGGACGGCTCGTCCTCAGAAACATTTCGTAGTCTTCTCGTTGGTTACTCCA
CTGCCGCGCCGCGTCTGCTTGGATGCTGAACTGCGAGATTTCTTAGAGTGCTCTACTAC
CCGACCGGGGGCAGCATAGCACCGTGGGGTCGGCGCGGCCCACCTCCGGCCAACGAG
AGTTACGAGTACCCGGCCCGTTTTCCCGCGTCGGAGGCTGCCAACACGATAGTAACTG
AACTGGCCCAAACGTATTAATACGCCCCGGGGGCGGGCCTCTGGCGCGCCACTGGATC
AGGCCCGTGGCGTGCCCGCCTCGTCAGCGCCACCCATTGCTAAGCGCTGACAGTAATA
GACCCCTCCATAGTAGTTGCCGATGTTGATTCGGTCACCGGCCGAAACGTATGCACTCA
GCACAGGGCAGGTACTACGGAGCGAAAGGTGGATAATTGGCAGGGGCTGCTGGCGCG
CCTACCAACCTGTTCCCCCGCGCCTTCTGGAGCGACCAACTACGCCCTGTGGCGACCGA
TACTTGAGCTGTCTCTCAACTGGGTCAATTAGGTTGTATACATCCTTGTTGGGTCGCTG
CCTAAACAACCTCCTCATACCCATCCGCCCGTCCGGAGCGGAACGACTCGGCGGTACC
GGCGTCAGGCCCCCTCGCCTAGGCTGCGCCATTATGTTGGGAGGTGCGTCGACTGGGG
GCCGGCAACCTGTGGTCGGCCGTTTCGTGAAGCCCATCACTCCCATGGAGACGTTTTCT
CCCATCCATTGGCCCCCGGGGTCCTCCACCGATTGCGTCGGAGTCTTGTCTAATGTGAA
ATTTATTCATCGTGAGGTATAAACCGCCCCCGCGTGGGTGCGGCTCGAAGTCCGGCCTC
CCAAGCTGCCTGCTAACACGGAAGTGTACCCGGTTCCACTCAGAGGGCATGAGGCAGT
ACCTACGGATCTCAACAAGCCTAGTCCCCATGATACGTACAGCTGGGCCAGGTGGGAT
TAATACGTCGGGGTCCCGTCGCGGGATGTTTGAGGGGGAGATACGTTCCGGCCATATA
CTCTTTGTCCGCATCCCTTGGGGAAATCCATTACGCTGCCGTTGACGTCGTTAACGTCA
CGCCACTAACACCGAACTAGGGGGACCAAACTATACCATGTTATCCGTATGCCTACTC
GTTGTTGCGGTTCAGAGTACGGGGCTGTGAGTAATGTACGCGCTCCTGGTGGGAACTT
GTCCTCCGGACTTGAATAACAGCTCACTGGGATGGGATTTAGCAAACCATCCCAACAT
ATTCCAGGCCTCTGATCAGATCATAATTTTGCTGTCACCAGTTCCCAATTACGGTTCCG
CATCGTCGGGAGGCCTTCGACTAGATCTACTCGCATGGTTCGAGCGGTAATATCGGGTC
TGAAATCCCCTGACCCAACGGGAGGCGGTATGTGTGATCGGGATCTGCACCTTGCGAG
AGCTAAGCGTAATGACGATGGCTCTTCCACAAGTGAGTGCTGGGGCGCGAGCAGCGGA
CCAGATTGTCTTCCAAATGTTCCCCCCCGTGCCGAAGGCTGAACTCAAGGCGGGGCCG
TGGTTGCGCACCGTTACCACGCAGGTGCACCGCGAGGCCCCTTGAAGGTCCCGTCATG
AAG

>BP_ERR173177.1

GTCGGAATGCCCCCTCGACTCTGGCACGGCGCGGCGCCTTCGCGCACGGATCGGATAA
GCTTTCCCTTGGACGGAGCGTCCTCAGCGACATCTTGTAGTCTTCTCGTTAGTTGCCCC
GATAACGCGCCGCGTCCGCTTGGATACCGAACCGCGAGAGCTATTCGAGAGTTCTGTT
ACCCGACCGGGGGCAGCACACAACCGTGGGATCGGCGCGGCCCACCTCCGGCCAACG
AGAGTTACGAGTACCCGGCGCGTTTTCCCGCGCCGGAGGCTGCCAACACGATAGTAAC
TGAACTGGCCCGAGCGTATTGATACGCCCCGGGGGCGGGCCTCTGGCGCGCCACTGGA
TCAGGCCCGTGGCGTGCCCGCCTCGTCAGCGCCACCCATTGCTAAGCGCTGACAGTAA
TAGACCCCTCCATAGTAGTTGCCGATGTTGACCCGATCACCGGCCGGTGCGTATGCACT
CAGCACAGGGCAGGTACCACAGAGCGAAAAGTGGATGATTGGCAGGGGCTGCTGACG
CGCTTGCAAGCCTGTTCCCCCGGGCCTGCTGGAGCGACCAACTACGCCCCGCAGCGAC

CGGTACCCGAGCAGTCTCTCAACTGGTCCGATGAGACTGTGTACATCGTCGTCGGGAC
GCGGACTAAACAACCCCCTCATACCCATCCGCCCGTCCGGAGCGGAACGACTCGGCGG
TACCGGTGTCAGGCCCCCTCGCCTAGGCTGCACCATAATGTTGTGCGGCGTGTCGACTG
GGGGCCGGCGACTTGAGCTCGGTCGTCCCGTGAAGCCCATCAGTCCCATGGAGACGTT
CTCTCCCATCCATTGGCCTCCGGGGCTCTCCACCAATCGCACCGGAGTCTTGTCTAGTG
TGAAATGTATTCATCGTGGGGCACAAGCCGCCCCCGCATGAGTGCAGCTCGAAGTTCG
GCCTCCCGGGCTGCCAGCTAACACGGAAGTGTGCCTCGTTTCGCTCAGAGGGGACGAA
ATGCTCTCGACGGCTCTCAACAAGCCTAGTCCCCACGATACATACAACCATGCCCGGT
GGGATTAATACGTCGGGGTCCCGCTGCGAGACGTCTGAGAGGGAGATACGTTCCGGCC
ATATACTCTCTGTCCGCGTACTTCGGAGAAATCCATTACGTTGCCGCCGACGTCGTTAA
CGTCACGCCATTAATACCGAACCAGGGGGACCAAACCTTGCCATGTAATCCCTGTATCT
ACCCACTGTTGCGGTTCAGGGAACGGGGGTGTGAGTAATGTACGCACTTCTGGTGGGA
ACTTGGCCTCCGGACTTGAATGGCTAGTCGTCGGGCTGGAATTTAGCAAACCACCTTGG
CGCTTTCCAGGCCTCTGATTAGGTTACTATTTAACGTTGACCGGGTCCCAATTACGGTT
CCGCATCGTCGGGAGGCCTTCGACTAGATCTACTCGCATGGTTCGAGCGGTAATATCG
GGTCTGAAATCCCCTGACCCAACGGGAGGCGGTATGTGTGATCGGGATCTGCACCTTG
CGAGAGCTAAGCGTAATGACGATGGCTCTTCCACAAGTAAGTGCTGGGGCGCGAGCGG
CGGACCAGATGGTCTTCCGAATGTTCCCCCCCGTGCCGAAGGCTGAACTCGAGGCGGA
GCCGTGGGCGCGCACCGTTGCTACGCAGGTGCACCACGAGGCCCCTTGAAGGTCCCGT
CATGAAG

>BP_ERR173177.2

GTCGGGATGCCTTCCCAACTCTGGCACGGCGCGGCGCCTTCGCGCACAGTTCGTCTAAG
CTCTCCCTTGGACGGATCGTCCTCAGCGACATCTCGTAGTCTTCTCGTTGGTTGCCCCG
ATAACGCGCCGCGTCCGCTTGGATACCGAACCGCGAGAGCTATACGAGAGTTCTGTTA
CCCGACCGGGGGCAGCACACAACCGTGGGATCGGCGCGGCCCACCTCCGGCCAACGA
GAGTTACGAGTACCCGGCGCGTTTTCCCGCGTCGGAGGCTGCCAACACGATAGTAACT
AAACTGGCCCAAGCGTATTGATACGCCCCAGGGGCGGGCCTCTGGCGCGCCACTGGAT
CAGGCCCGTGGCGTGCCCGCCTCGTCAGCGCCACCCATTGCTAAGCGCTGACAGTAAT
AGACCCCTCCATAGTAGTTGCCGATGTTGACCCAATCACCGGCCGAAACGTATGCACT
CAGCACAGAGCAGGCATCACGGAGCGAAAAGTGGATGATTGGCAGGGGCTGCTGGCG
CGCCTATCAACCTGTTCCCCTCGGCCTGCTGGAGCGACCAACTACGCCCCGCAGCGACC
GGTACCCGAGCAGTCTCTCAACTGGTCCGATGAGACTGTGTTCATCGTCGTCGGGACGC
GGACTAAACAACCCCCTCATACCCATCCGCCCGTCTGGAGCGGAACGACTCGGCGGTG
CCGGCGTCAGGCCCCCTCGCCTAGGCTGCACCATTATGTTGGGAGGTGCGTCGACCGG
GGGCCGGCGACTTGAGCTCGATCGTCCCGTGAAGCCCATCGGTCCCATGGAGACGTTC
TCTCCCATCCATTGGCCTCCGGGGCTCTCCACCAATCGCGCTGGAGTCTTGTCTAGTGT
TAAATGTATTCATCGTGGGGTATAAACCGCCCCCGCGTGGGTGCAACTCATAATCCGG
CCCCTCGGGCTGCCAGCTAACACAGAAGTGTGCCTCGCTTCGCTCAGAGGGGACGAGA
CACTCTCGACGGCTCTCAACAAGCCTAGTCCCTACGATACATACAACCATGCCCGGTG
GGATTAATACGTCGGGGTCCCGTCGCGGGATGTCTAGGATGGGAACACGTTCCGGCCA
TATACTCTCTTTCCGCGTACTTCGGAGAAATCCATTACGTTGCTGCCAACATCGTCCCC
ACGACGCCACGGACGCTGAGTTAAGGGAACCGGACCTTGCCATTTAGCCCCTATATCT
ACCCACTGTTGCGGTTCAGGGAACGGGGGTGTGAGTAGTGTAGGCACTTCTGGTGGGA
ACTTGGCCTCCGGACTTGAATGGCTAGTCGTCGGGCTGGAATTTAGCAAACCACCTTGG
CGCTCTCCACACCTCTTATTAGGTTACTATTTAACGTTGACCAGTTCCCACTTACGGTTC
CGCAGCGTCGGTAGGCCTTCGACTAGATCTACTCCCATGGTTCGAGCGGTAATATCGG
GTCTGGTGTCCCCTGATCCAACGGGAGGCGGTATGTGTGATCGGGATCTGCACCTTGCG
AGAGCTAAGCGTAATGACGATGGCTCTTCCACAAGTGAGTGCTGGGGCGCGAGCGGCG
GACCAGATGGTCTTCCGAATGTTCCCCCCCGTGCCGAAGGCTGAACTCGAGGCGGAGC

CGTGGGCGCGCACCGTTGCTACGCAGGTGCACCGCGAGGCCCCGTGGAGGTCCCGTCA
TGAAG

>AWB_ERR173178.1

GTCGGGATGCCTCCCCGGCTCTGGCACGGCGCGGTGCCTCCGTGCACGGATCGGATAA
GCTTTCCCGTGGACGGCTCGTCCTCAGAAACATTTCGTAGTCTTCTCGTTGGTTACTCCA
CTGCCGCGCCGCGTCTGCTTGGATGCTGAACCACGAGATTTCTTAGAGTATTCTACTAC
CTGACCAGGGGCAGTATACAACCGTGGAAACGACGCGGCCCACCTCCGGTCAACGAG
AGTTACGAGTACCCGGCCCGTTTTCCCGTGTCGGAGGCTGCCAACACGATAGTAACTG
AACTGGCCCAAACGTATTAATATGCCCAGGGGGCGGGCCTCTGGCGCGCCACTGGATC
AGGCCCGTGGCGTGCCCGCCTCGTCAGCGCCACCCATTGCTAAGCGCTGACAGTAATA
GACCCCTCCATAGTAGTTGCCGATGTTGATTCGGTCACCGGCCGAAACGTATGCACTCA
GTACAGGGCAGGTACTACGGAGCGAAAGGTGGATAATTGGCAGGGGCTGCTGGCGCG
CCTACCAACCTGTTCCCCCGCGCCTGCTGGAGCGACTAACTACGCCCTGTGGCGACCGA
TACTTGAGCTGTCTCTCAACTGGGTCAATTAGGTTGTATACATCCTTGTTGGGTCGCTG
CCTAAACAACCTCCTCATACCCATCCGCCCGTCCGGAGCGGAACGACTCGGCGGTACC
GGTGTCAGGCCCCCTCGCCTAGGCTGCGCCATAATGTTGGGAGGTGCGTCGACTGGGG
GCCGGCAACCTGAGGTCGGCCGTTTCGTGAAGCCCATCACTCCCATGGAGACGTTTTCT
CCCATCCATTGGCCCCCGGGGTCCTCCACCGATTGCGTCGGAGTATTGTCTAATGTGAA
ATTTATTCATCGTGAGGTATAAACCGCCCCCGCGTGGGTGCGGCTCGAAGTCCGGCCTC
CCAAGCTGCCTGCTAACACGGAAGTGTACCCGGTTCCACTCAGAGGGCATGAGGCAGT
ACCTACGGATCTCAACAAGCCTAGTCCCTATGATACGTACAGCTGGGCCAGGTGGGAT
TAATACGTCGGGGTCCCGTCGCGGGATGTTTGAGGGGGAGATACGTTCCGGCCATATA
CTCTTTGTCCGCATCCCTTGGGGAAATCCATTACGTTGCCGTTGACGTCGTTAACGTCA
CGCCACTAACACCGAACTAGGGGGACCAAACTATACCATGTTATCCGTATGCCTACTC
GTTGTTGCGGTTCAGAGTACGGGGCTGTGAGTAATGTACGCGCTCCTGGTGGGAACTT
GTCCTCCGGACTTGAATAACAGCTCACTGGGATGGGATTTAGCAAACCATCCCAACAT
ATTCCAGGCCTCTGATCAGATCATAATTTTGCTGTCACCAGTTCCCAATTACGGTTCTG
CATCGTCGGGAGGCCTTCGACTAGATCTACTCGCATGGTTCGAGCGGTAATATCGGGTC
TGAAATCCCCTGACCCAACGGGAGGCGGTATGTGTGATCGGGATCTGCACCTTGCGAG
AGCTAAGCGTAATGACGATGGCTCTTCCACAAGTGAGTGCTGGGGCGCGAGCAGCGGA
CCAGATTGTCTTCCAAATGTTCCCCCCCGTGCCGAAGGCTGAACTCAAGGCGGGGCCG
TGGTCGCGCACCGTTACCACGCAGGTGCACCGCGAGGCCCCTTGAAGGTCCCGTCATG
AAG

>AWB_ERR173178.2

GTCGGGATGCCTCCCCGGCTCTGGCACGGCGCGGTGCCTTCGTGCACGGATCGGCCAA
GCTTTCCCGTGGACGGCTCGTCCTCAGAAACATTTCGTAGTCTTCTCGTTGGTTACTCCA
CTGCCGCGCCGCGTCTGCTTGGATGCTGAACTACGAGATTTCTTAGAGTACTCTACTAC
CCGACCGGGGGCAGTATACAACCGTGGGAACGACGCGGCCCACCTCCGGTCAACGAG
AGTTACGAGTACCCGGCCCGTTTTCCCGCGTCGGAGGCTGCCAACACGATAGTAACTG
AACTGGCCCAAACGTATTATTACGCCCAGGGGGCGGGCCTCTGGCGCGCCACTGGATC
AGGCCCGTGGCGTGCCCGCCTCGTCAGCGCCACCCATTGCTAAGCGCTGACAGTAATA
GACCCCTCCATAGTAGTTGCCGATGTTGATTCGGTCACCGGCCGAAACGTATGCACTCA
GCACAGGGCAGGTACTACGGAGCGAAAGGTGGATAATTGGCCGGGGCTGCTGGCGCG
CCTACCAACCTGTTCCCCTGCGCCTGCTGGAGCGACCAACTACGCCCCGCAGCGACCG
GTACCCGAGCAGTCTCTCAACTGGTCCGATTCTATTGTATAAACAGTCGTTGGGACGCG
GACTAAACAACCCCCTCATACCCATCCGCCCGTCTGGAGCGGAACGACTCGGCGGTAC
CGGCGTCAGGCCCCCTCGCCTAGGCTGCACCATTATGTTGGGAGGTGCGTCGACTGGG
GGCCGGCAACCTGTGGTCGGCCGTTTCGTGAAGCCCATCACTCCCATGGAGACGTTTTC

TCCCATCCATTGGCCCCCGGGGTCCTCCACCGATTGCGTCGGAGTCTTGTCTAATGTTA
AATTTATTCATCGTGAGGTATAAACCGCCCCCGCGTGGGTGCGGCTCGAAGTCCGGCCT
CCCAAGCTGCCTGCTAACACGGAAGTGTACCCGGTTCCACTCAGAGGGCATGAGGCAG
TACCTACGGATCTCAACAAGCCTAGTCCCCATGATACGTACAGCTGGGCCAGGTGGGA
TTAATACGTCGGGGTCCCGTCGCGGGATGTTTGAGGGGGAGATACGTTCCGGCCATAT
ACTCTTTGTCCGCATCCCTTGGGGAAATCCATTACGCTGTCGTTGACGTCGTTAACGTC
ACGCCACTAACACCGAACTAGGGGGACCAAACTATACCATGTTATCCGTATGCCTACT
CGTTGTTGCGGTTCAGAGTACGGGGCTGTGAGTAATGTACGCGCTCCTGGTGGGAACTT
GTCCTCCGGACTTGAATAACAGCTCACTGGGATGGGATTTAGCAAACCATCCCAACAT
ATTCCAGGCCTCTGATCAGATCATAATTTTGCTGTCACCAGTTCCCAATTACGGTTCTG
CATCGTCGGGAGGCCTTCGACTAGATCTACTCGCATGGTTCGAGCGGTAATATCGGGTC
TGAAATCCCCTGACCCAACGGGAGGCGGTATGTGTGATCGGGATCTGCACCTTGCGAG
AGCTAAGCGTAATGACGATGGCTCTTCCACAAGTGAGTGCTGGGGCGCGAGCAGCGGA
CCAGATTGTCTTCCAAATGTTCCCCCCCGTGCCGAAGGCTGAACTCAAGGCGGGGCCG
TGGTCGCGCACCGTTACCACGCAGGTGCACCGCGAGGCCCCTTGAAGGTCCCGTCATG
AAG

>JQH_ERR173179.1

GTCGGGACGCCCTCCCGGCTCTGGCACGGCGCGGTGGCTTTGCGCACGGATCGGATAA
ACTTTCCATTGGACGGCTCGTTCTCAGAAATATCTCGTAGTCTTCTCGTTGGTTACTCCA
ATGAAACGCCTCGTCTGCGGGGGCACTGAACCGCGAGAGCTATTCAAATGCTCTACTA
CTCGACCGGGGGCAGCTTACAACCGTGGGATCGGCGCGGCCCACCTCCGGTCAACGAG
AGTTACGAGTTCCCGGCCCGTTTTCCCGCGTCGGAGGCTGCCAACACGATAGTAACTG
AACTGGCCCAAACGTATTAATACGCCCCGGGGGCGGGCCTCTGGCGCGCCACTGGATC
AGGCCCGTGGCGTGCCCGCCTCGTCAGCGCCACCCATTGCTAAGCGCTGACAGTAATA
GACCCCTCCATAGTAGTTGCCGATGTTGATTTGGTCACCGGCCGAAACGTATGCGCTCA
GCACAGGGCAGGTACTACGGAGCGAAAGGTGGATGATTGGCAGGGGCCGCTGGCGCA
CCTACAAAACTATTCGTCCGCGCCTGCTGGAGCGACCAACTACGCTCTATAGCGTCCAG
TACCCGAGCAGTCTCTCAATTGGTCCGATGAGACTGTATACGCCGCCGTTGGGACGCG
GACTAAACAACCCCCTCATACCCATTCGCCCGTCCAGAGCGGAACGACTCCGCGGTGC
CGGCGTCAGGCCTCCTCGCCTAGGCTGCGCCATTATGTTGGGAGGTGCGTTGACTGGG
GGCTGGCAATTTGATCCCGGTCGGCCCGTGAAGCCCATCAGTTCCATGGAGACGTTCTT
TCCCGTCCATTGGCCTCCGGGGCCCTCCACCAATTACGTCGGAGTATTGCCTAGGGTGA
AATGCATTCATCGTGAGGTATAAACCGCCCCCGCGTGGGTGCGGCTCGATGTCTGGCCT
CCCAGGCTGGCAGCTAACACGGAAGTGTGCCTCGTTCCACTCAGAGGGCATGAGGCAG
TACCTACGGATCTCAACAAGCCTAGTCCCCATGATACGTACAGCTGGGCCAGGTGGGA
TTAATACGCCGGGGTCCCGTCGCGGGATGTTTGAGGGGGAGATACGTTCCGGCCATAT
ACTTTTTGTCCGCATCCCTTGGGGAAATCCATTACGCTGCCGTTGACGTCGGTAACGCC
ACGCCATTAACACCGAACCAGGGAGACCAAACTACACCATGTTATCCGTATGCCCAGT
TGTTGTTGCGGTTCAGGGTACGGGGCTGTGAGGCACGGGCGCGCTCCTGGTGGGAACT
TGTCCTCCGGACTTTAATGGCTACTCGCCGGGCTGAGACTTAGCAGACCACCCTGACGC
ATTCCAGGCCTCAGATTAGATCACAATTTTGCTTTCATTAGTTCCCAATTACGGTTCCGC
ATCGTCAGGAGGCCTTCGACTAGATCTACTCGTATGGTTCGAGCGGTGATATCGGGTCT
AAAATCCCCTGACCCAACGGGGGGCGGTACGTGTGATCGGAATGTGCACCTTGCGAGA
TCTAATCGTAATGACGATGGCTCTTCCACAAGTGAGTGCTGGGGCGCGAGCGGCGGAC
CAGATGGCCTTCCGAATGTTCCCCCCCGTGCCGAAGGCTTAACTCGAGGCGGAGCCGT
GGGCTCGCACCGTTGCCGCGCAGGTACACCGCGAGGTCCCTTGAAGGTCCCGTCATGA
AG

>JQH_ERR173179.2

GTCGGGATGCCTTCCCAACTCTGGCACGGCGCGGCGCCTTCGCGCACGGATCGGATAA
GCTTTCCCGTGGACGGCTCGTCCTCGGAAATATCTCGTAGTCTTCTCGTTGGTTGCTCCA
ACGAAGCGACGAATCTACTTGGATACTGAACCGCGAGAGCTATTCAAGTGCTCTACTA
CTCGACCGGGGGCAGCATACAGCCGTGGGATCGGCGCGGCCCACCTCCGGCCAACGAG
AGTTACGAGTACCCGGCCCGTTTTCCCGCGTCGGAGGCTGCCAACACGATAGTAACTG
AACTGGCCCAAACGTATTGATACGCCCCGGGGGCGGGCCTCTGGCGCGCCACTGGATC
AGGCCCGTGGCGTGCCCGCCTCGTCAGCGCCACCCATTGCTAAGCGCTGACAGTAATA
GACCCCTCCATAGTAGTTGCCGATGTTAATTCGGTCACCGGCCGAAACGTATGAATGC
AGCACAGGGCAGGTACTACGGAGCGAAAGGTAGCTGATTGGCAGGGGCTGCTGGCGC
GCCTACCAACCTGTTCCTCCGCGCCTGCTGGAGCGACCAACTACGCCCCGCAGCGACC
GGTACCCGAGCAGTCTCTCAACTGGTTCGATGAGACTGTATACACCGTCGTTGGGACG
CGGACTAAACAACCTCCTCATACCAATCCGCCCGTCCGGAGCGGAACGACTCGGCGGT
ACCGGCGTCAGGCCCCCTCGCCTAGGCTGCACCATTAGGTTGGGAGGTGCGTCGACTG
GGGGCCGGCGACTTGAGCTCGGTCGTCCCGTGAAGCTCATCAGTCCCATGGAGACGTT
CTCTCCCATCCATTGGCCTCCGGGGCTCTCCACCAATCGCACCGGAGTCTTGTCTAGTG
TTAAATGTATTCATCGTGGGGTATAAATCGCCCCCGCGTGAGTGCGGCTCGAAGTCTGG
CCTCCCAGGCTGCTAGCTAACACGGAAGTGTGCCTCGTTTCGCTCAGAGGGGACGAGA
CACTCTCGACGGCTCTCAACAAGCCTAGTCCCCACGATACATACAACCGTGCCCGGTG
GGATTAATACGCCGGGGTCCCGTCGCGGGACATCTGGGATGGGAACACGTTCCGGCCA
TATGCTCTCTGTCCGCATCTCTTGGGGAAATCCATTACGCTGCCGTTGACGTCGTTAAC
GTCACGCCACTAATACCGAACTAGGGGGACCAAACTATACCATGTTATCCGTGCGCCT
ACCCACTGTTGCGATTCAGGGTACGGGGCTGTGAGTAATGTACACACTCCTGGTGGGA
GCTTGTCCTCCGGACTTGAATGGCTACTCGCCGGGCTGGGACTTAGCAAACCACCCTGA
CGCATTCCAGGCCTCTGATCAGATCACAATTTTGCTTTCACCAGTTCCCAATTACGGTT
CCGCATCGTCGGGAGGCCTTCGACTAGATCTACTCGCATGGTTCGAGCGGTAATATCG
GGTCTGAAATCCCCTGACCCAACGGGAGGCGGTACGTGTGATCGGAATCTGCACCTTG
CGAGAGCTAATCGTAATGACGATTGCTCTTCCACAAGTGAGTGCTGGGGCGCGAGTGG
CGGACCAGATTGTCTTCCGAATGTTCCCCCCCGTGTCGAAGGCTGAACTCGAGGCGGA
GCCGTGAGCGCGCACCGTTGCCACGCAGGTGCACCGCGAGGCACCTCGAAGGTCCCGT
CATGAAG

>LR_ERR173180.1

GTCGGGATGCTTTCCCAACTCCGGCACGGCGCGGCGCCTTCGCGCACGGATCGGATAA
GCTTTCCCGTGGACGGCTCGTCCTCAGAAACATTTCGTAGTCTTCTCGTTGGTTACTCCA
CTGCAGCGCCGCGTCTGCTTGGATGCTGAACCGCGAGAGCTATTCAAGTGCTTTACTAC
TCGACCGGGGGCAGCTTACAACCGTGGGATCGGCGTGGCCCACCTCCGGCCAACGAGA
GTTACGAGTACCCGGCCCGTTTTCCCGCGTCGGAGGCTGCCAACACGATAGTAGCTGA
ACTGGCCCAGACGTATTGATACGCCCCGGGGGCGGGCCTCTGGCGCGCCACTGGATCA
GGCCCGTGGCGTGCCCGCCTCGTCAGCGCCACCCATTGCTAAGCGCTGACAGTAATAG
ACCCCTCCATAGTAGTTGCCGATGTTAATTCGGTCACCGGCCGAAACGTATGCACTTAG
CACAGGGCAGGTACTACAAAGCGAGAGGTGGACGATTGGCAAAGGCTGCTGGCGAGC
CTACCAACCTGTTCCTCCGCGCCTGCTGGAGCGGCCAACTACGCCCCGCAGCGACCGG
TACCCGAGCAGTCTCTCAACTGGTTCGATGAGACTGTATACACCGTCGTTGGGACGCG
GACTAAACCGCCCCCTCATACCCACCCGCCCGTCCGGAACGGAACGACTCGGCGGTAC
CGGCGTCAGGCCCCCTCGCCTAGGCTGCACCATTATGTTGGGAGGTGCGTCGACTGGG
GGCCGGCGACTTGAGCTCGGTCGTCCCGTGAAGCCCATCAGTCCCATGGAGACGTTCT
CTCCCATCCATTGGCCTCCGGGGCTCTCCACCAATCGCACCGGAGTCTTGTCTAGTGTT
AAATGTATTCATCGTGGGGTATAAACCGCCCCCGCGTAAGTGCGGCTCGAAGTCTGGC
CTCCCAGGCTGCTAGCAAACACGGAAGTGTGCCTCGTTTCGCTCAGAGGGGACAAGAC
ACTCTCGACGGCTCTCAACAAGCCTAGTCCCCACGATACATACAACCGTGCCCGGTGG
GATTAATACGCCGGGGTCCCGTCGCGGGACATCTGGGATGGGAACACGTTCCGACCAT

ATGCTCTCTGTCTGCATCTCTTGGGGAAATCCATTACGCTGCCGTTGACGTCGTTAACG
TCACGCCACTAATACCGAACTAGGGGGGCCAAGCTATACCATGTAATCCGTGCGCCTA
CCCACTGTTGCGATTCAGGGTACGGGGCTGTGAGTAATGTACACACTCCTGGTGTGAG
CTTGTCCTCCGGACTTGAATGGCTACTCGCCGGGCTGGGACTTAGCAAACCACCCTGAC
GCATTCCAGGCCTCTGATCAGATCACAATTTTGCTTTCACCAGTTCCCAATTACGGTTC
CGCATCGTCGGGAGGCCTTCGACTAGATCTACTCGCATGGTTCGAGCGGTAATATCGG
GTCTGAAATCCCCTGACCCAACGGGAGGCGGTACGTGTGATCAGGATCTGCACCTTGC
GAGAGCCAAGCGTAATGACGGTGGCTCTTCCACAAGTAAAAGCTCGGGCGCGAGCGG
CGGGCTGGATGGTCCCCCGAATGTTCCCCCCTATGCCGAAGGCTGAACTCGAGGCTGA
GCCGTGGGCGCGCACCGTTGCCACGCAGGTGCACCGCGAGGTCCCTTGAAGGTCCCGT
CACGAAG

>LR_ERR173180.2

GTCGGGATGCCTTCCCAACTCTGGCACGGCGCGGCGCCTTCGCGCACGGATCGGATAA
GCTTTCCCGTGGACGGCTCGTCCTCGGAAATATCTCGTAGTCTTCTCGTTGGTTGCTCCA
ACGAAGCGACGAATCTACTTGGATACTGAACCGCGAGAGCTATTCAAGTGCTCTACTA
CTCGACCGGGGGCAGCATACAGCCGTGGGATCGGCGCGGCCCACCTCCGGCCAACGAG
AGTTACGAGTACCCGGCCCGTTTTCCCGCGTCGGAGGCTGCCAACACGATAGTAACTG
AACTGGCCCAAACGTATTGATACGCCCCGGGGGCGGGCCTCTGGCGCGCCACTGGATC
AGGCCCGTGGCGTGCCCGCCTCGTCAGCGCCACCCATTGCTAAGCGCTGACAGTAATA
GACCCCTCCATAGTAGTTGCCGATGTAGGTCTGGTCACCGGCCGAAACGTATGAACGC
AGCACAGGGCAGGTACTACGGAGCGAAAGGTAGCTGATTGGCAGGGGCTGCTGGCGC
GCCTACCAACCTGTTCCTCCGCGCCTGCTGGAGCGACCAACTACGCCCCGCAGCGACC
GGTACCCGAGCAGTCTCTCAACTGGTTCGATGAGACTGTATACACCGTCGTTGGGACG
CGGACTAAACCGCCCCCTCATACCCACCTGCCCGTCCGGAGCGGAACGACTCGGCGGT
ACCGGCGTCAGGCCCCCTCGCCTAGGCTGCACCATTATGTTGGGAGGTGCGTCGACTG
GGGGCCGGCGACTTGAGCTCGGTCGTCCCGTGAAGCCCATCAGTCCCATGGAGACGTT
CTCTCCCATCCATTGGCCTCCGGGGCTCTCCACCAATCGCACCGGAGTCTTGTCTAGTG
TTAAATGTATTCATCGTGGGGTATAAACCGCCCCCGCGTAAGTGCGGCTCGAAGTCTG
GCCTCCCAGGCTGCTAGCAAACACGGAAGTGTGCCTCGTCTCGCTCAGAGGGGACAAG
ACACTCTCGACGGCTCTCAACAAGCCTAGTCCCCACGATACATACAACCGTGCCCGGT
GGGATTAATACGCCGGGGTCCCGTCGCGGGACATCTGGGATGGGAACACGTTCCGGCC
ATATGCTCTCTGTCCGCATCTCTTGGGGAAATCCATTACGCTGCCGTTGACGTCGTTAA
CGTCACGCCACTAATACCGAACTAGGGGGACCAAACTATACCATGTAATCCGTGCGCC
TACCCACTGTTGCGATTCAGGGTACGGGGCTGTGAGTAGTGTACACACTCTTGGTGGG
AGCTTATCCTCCGGACTTGAATGGCTACTCGCCGGGCTGGGACTTAGCAAACCACCCTG
ACGCATTCCAGGCCTCTGATCAGATCACAGTTTTGCTTTCACCAGTTCCCAATTACGGT
TCCGCATCGTCGGGAGGCCTTCGACTAGACCTACTCGCATGGTTCGAGCGGTAATATCG
GGTCTGAAATCCCTTGACCCAACGGGAGGCGGTACGTGTGATCGGAATCTGCACCTTG
CGAGAGCTAATCGTAATGCCGATGGCTCTTCCACAAGTGAGTGCTGGGGCGCGAGTGG
CGGACCAGATTGTCTTCCGAATGTTCCCCCCCGTGTCGAAGGCTGAACTCGAGGCGGA
GCCGTGAGCGCGCACCGTTGCCACGCAGGTGCACCGCGAGGCACCTCGAAGGTCCCGT
CATGAAG

>LR_ERR173181.1

GTCGGGATGCCTTCCCAACTCTGGCACGGCGCGGCGCCTTCGCGCACGGATCGGATAA
GCTTTCCCGTGGACGGCTCGTCCTCGGAAATATCTCGTAGTCTTCTCGTTGGTTGCTCCA
ACGAAGCGACGAATCTACTTGGATACTGAACCGCGAGAGCTATTCAAGTGCTCTACTA
CTCGACCGGGGGCAGCATACAGCCGTGGGATCGGCGCGGCCCACCTCCGGCCAACGAG
AGTTACGAGTACCCGGCCCGTTTTCCCGCGTCGGAGGCTGCCAACACGATAGTAACTG

AACTGGCCCAAACGTATTGATACGCCCCGGGGGCGGGCCTCTGGCGCGCCACTGGATC
AGGCCCGTGGCGTGCCCGCCTCGTCGGCGCCACCCATTGCTAAGCGCTGACAGTAATA
GACCCCTCCATAGTAGTTGCCGATGTAGGTCTGGTCACCGGCCGAAACGTATGAACGC
AGCACAGGGCAGGTACTACGGAGCGAAAGGTAGCTGATTGGCAGGGGCTGCTGGCGC
GCCTACCAACCTGTTCCTCCGCGCCTGCTGGAGCGACCAACTACGCCCCGCAGCGACC
GGTACCCGAGCAGTCTCTCAACTGGTTCGATGAGACTGTATACACCGTCGTTGGGACG
CGGACTAAACCGCCCCCTCATACCCACCCGCCCGTCCGGAGCGGAACGACTCGGCGGT
ACCGGCGTCAGGCCCCCTCGCCTAGGCTGCACCATTATGTTGGGAGGTGCGTCGACTG
GGGGCCGGCGACTTGAGCTCGGTCGTCCCGTGAAGCCCATCAGTCCCATGGAGACGTT
CTCTCCCATCCATTGGCCTCCGGGGCTCTCCACCAATCGCACCGGAGTCTTGTCTAGTG
TTAAATGTATTCATCGTGGGGTATAAACCGCCCCCGCGTAAGTGCGGCTCGAAGTCTG
GCCTCCCAGGCTGCTAGCAAACACGGAAGTGTGCCTCGTTTCGCTCAGAGGGGACAAG
ACACTCTCGACGGCTCTCAACAAGCCTAGTCCCCACGATACATACAACCGTGCCCGGT
GGGATTAATACGCCGGGGTCCCGTCGCGGGACATCTGGATGGGAACACGTTCCGACC
ATATGCTCTCTGTCTGCATCTCTTGGGGAAATCCATTACGCTGCCGTTGACGTCGTTAA
CGTCACGCCACTAATACCGAACTAGGGGGGCCAAGCTATACCATGTAATCCGTGCGCC
TACCCACTGTTGCGATTCAGGGTACGGGGCTGTGAGTAATGTACACACTCCTGGTGTGA
GCTTGTCCTCCGGACTTGAATGGCTACTCGCCGGGCTGGGACTTAGCAAACCACCCTGA
CGCATTCCAGGCCTCTGATCAGATCACAATTTTGCTTTCACCAGTTCCCAATTACGGTT
CCGCATCGTCGGGAGGCCTTCGACTAGATCTACTCGCATGGTTCGAGCGGTAATATCG
GGTCTGAAATCCCCTGACCCAACGGGAGGCGGTACGTGTGATCAGGATCTGCACCTTG
CGAGAGCCAAGCGTAATGACGGTGGCTCTTCCACAAGTAAAAGCTCGGGCGCGAGCG
GCGGGCTGGATGGTCCCCCGAATGTTCCCCCCTATGCCGAAGGCTGAACTCGAGGCTG
AGCCGTGGGCGCGCACCGTTGCCACGCAGGTGCACCGCGAGGTCCCTTGAAGGTCCCG
TCACGAAG

>LR_ERR173181.2

GTCGGGATGCCTTCCCAACTCTGGCACGGCGCGGCGCCTTCGCGCACGGATCGGATAA
GCTTTCCCGTGGACGGCTCGTCCTCGGAAATATCTCGTAGTCTTCTCGTTGGTTGCTCCA
ACGAAGCGACGAATCTACTTGGATACTGAACCGCGAGAGCTATTCAAGTGCTCTACTA
CTCGACCGGGGGCAGCATACAGCCGTGGGATCGGCGCGGCCCACCTCCGGCCAACGAG
AGTTACGAGTACCCGGCCCGTTTTCCCGCGTCGGAGGCTGCCAACACGATAGTAACTG
AACTGGCCCAAACGTATTGATACGCCCCGGGGGCGGGCCTCTGGCGCGCCACTGGATC
AGGCCCGTGGCGTGCCCGCCTCGTCAGCGCCACCCATTGCTAAGCGCTGACAGTAATA
GACCCCTCCATAGTAGTTGCCGATGTAGGTCTGGTCACCGGCCGAAACGTATGAACGC
AGCACAGGGCAGGTACTACGGAGCGAAAGGTAGCTGATTGGCAGGGGCTGCTGGCGC
GCCTACCAACCTGTTCCTCCGCGCCTGCTGGAGCGACCAACTACGCCCCGCAGCGACC
GGTACCCGAGCAGTCTCTCAACTGGTTCGATGAGACTGTATACACCGTCGTTGGGACG
CGGACTAAACCGCCCCCTCATACCCACCCGCCCGTCCGGAGCGGAACGACTCGGCGGT
ACCGGCGTCAGGCCCCCTCGCCTAGGCTGCACCATTATGTTGGGAGGTGCGTCGACTG
GGGGCCGGCGACTTGAGCTCGGTCGTCCCGTGAAGCCCATCAGTCCCATGGAGACGTT
CTCTCCCATCCATTGGCCTCCGGGGCTCTCCACCAATCGCACCGGAGTCTTGTCTAGTG
TTAAATGTATTCATCGTGGGGTATAAACCGCCCCCGCGTAAGTGCGGCTCGAAGTCTG
GCCTCCCAGGCTGCTAGCAAACACGGAAGTGTGCCTCGTTTCGCTCAGAGGGGACAAG
ACACTCTCGACGGCTCTCAACAAGCCTAGTCCCCACGATACATACAACCGTGCCCGGT
GGGATTAATACGCCGGGGTCCCGTCGCGGGACATCTGGATGGGAACACGTTCCGACC
ATATGCTCTCTGTCTGCATCTCTTGGGGAAATCCATTACGCTGCCGTTGACGTCGTTAA
CGTCACGCCACTAATACCGAACTAGGGGGGCCAAGCTATACCATGTAATCCGTGCGCC
TACCCACTGTTGCGATTCAGGGTACGGGGCTGTGAGTAATGTACACACTCCTGGTGTGA
GCTTGTCCTCCGGACTTGAATGGCTACTCGCCGGGCTGGGACTTAGCAAACCACCCTGA
CGCATTCCAGGCCTCTGATCAGATCACAATTTTGCTTTCACCAGTTCCCAATTACGGTT

CCGCATCGTCGGGAGGCCTTCGACTAGATCTACTCGCATGGTTCGAGCGGTAATATCG
GGTCTGAAATCCCCTGACCCAACGGGAGGCGGTACGTGTGATCAGGATCTGCACCTTG
CGAGAGCCAAGCGTAATGACGGTGGCTCTTCCACAAGTAAAAGCTCGGGCGCGAGCG
GCGGGCTGGATGGTCCCCCGAATGTTCCCCCCTATGCCGAAGGCTGAACTCGAGGCTG
AGCCGTGGGCGCGCACCGTTGCCACGCAGGTGCACCGCGAGGTCCCTTGAAGGTCCCG
TCACGAAG

>LR_ERR173182.1

GTCGGGATGCCTTCCCAACTCTGGCACGGCGCGGCGCCTTCGCGCACGGATCGGATAA
GCTTTCCCGTGGACGGCTCGTCCTCGGAAATATCTCGTAGTCTTCTCGTTGGTTGCTCCA
ACGAAGCGACGAATCTACTTGGATACTGAACCGCGAGAGCTATTCAAGTGCTCTACTA
CTCGACCGGGGGCAGCATACAGCCGTGGGATCGGCGCGGCCCACCTCCGGCCAACGAG
AGTTACGAGTACCCGGCCCGTTTTCCCGCGTCGGAGGCTGCCAACACGATAGTAACTG
AACTGGCCCAAACGTATTGATACGCCCCGGGGGCGGGCCTCTGGCGCGCCACTGGATC
AGGCCCGTGGCGTGCCCGCCTCGTCAGCGCCACCCATTGCTAAGCGCTGACAGTAATA
GACCCCTCCATAGTAGTTGCCGATGTAGGTCTGGTCACCGGCCGAAACGTATGAACGC
AGCACAGGGCAGGTACTACGGAGCGAAAGGTAGCTGATTGGCAGGGGCTGCTGGCGC
GCCTACCAACCTGTTCCTCCGCGCCTTCTGGAGCGACCAACTACGCCCCGCAGCGACCG
GTACCCGAGCAGTCTCTCAACTGGTTCGATGAGACTGTATACACCGTCGTTGGGACGC
GGACTAAACCGCCCCCTCATACCCACCCGCCCGTCCGGAGCGGAACGACTCGGCGGTA
CCGGCGTCAGGCCCCCTCGCCTAGGCTGCACCATTATGTTGGGAGGTGCGTCGACTGG
GGGCCGGCGACTTGAGCTCGGTCGTCCCGTGAAGCCCATCAGTCCCATGGAGACGTTC
TCTCCCATCCATTGGCCTCCGGGGCTCTCCACCAATCGCACCGGAGTCTTGTCTAGTGT
TAAATGTATTCATCGTGGGGTATAAACCGCCCCCGCGTAAGTGCGGCTCGAAGTCTGG
CCTCCCAGGCTGCTAGCAAACACGGAAGTGTGCCTCGTTTCGCTCAGAGGGGACAAGA
CACTCTCGACGGCTCTCAACAAGCCTAGTCCCCACGATACATACAACCGTGCCCGGTG
GGATTAATACGCCGGGGTCCCGTCGCGGGACATCTGGGATGGGAACACGTTCCGACCA
TATGCTCTCTGTCTGCATCTCTTGGGGAAATCCATTACGCTGCCGTTGACGTCGTTAAC
GTCACGCCACTAATACCGAACTAGGGGGGCCAAGCTATACCATGTAATCCGTGCGCCT
ACCCACTGTTGCGATTCAGGGTACGGGGCTGTGAGTAATGTACACACTCCTGGTGTGA
GCTTGTCCTCCGGACTTGAATGGCTACTCGCCGGGCTGGGACTTAGCAAACCACCCTGA
CGCATTCCAGGCCTCTGATCAGATCACAATTTTGCTTTCACCAGTTCCCAATTACGGTT
CCGCATCGTCGGGAGGCCTTCGACTAGATCTACTCGCATGGTTCGAGCGGTAATATCG
GGTCTGAAATCCCCTGACCCAACGGGAGGCGGTACGTGTGATCAGGATCTGCACCTTG
CGAGAGCCAAGCGTAATGACGGTGGCTCTTCCACAAGTAAAAGCTCGGGCGCGAGCG
GCGGGCTGGATGGTCCCCCGAATGTTCCCCCCTATGCCGAAGGCTGAACTCGAGGCTG
AGCCGTGGGCGCGCACCGTTGCCACGCAGGTGCACCGCGAGGTCCCTTGAAGGTCCCG
TCACGAAG

>LR_ERR173182.2

GTCGGGATGCCTTCCCAACTCTGGCACGGCGCGGCGCCTTCGCGCACGGATCGGATAA
GCTTTCCCGTGGACGGCTCGTCCTCGGAAATATCTCGTAGTCTTCTCGTTGGTTGCTCCA
ACGAAGCGACGAATCTACTTGGATACTGAACCGCGAGAGCTATTCAAGTGCTCTACTA
CTCGACCGGGGGCAGCATACAGCCGTGGGATCGGCGCGGCCCACCTCCGGCCAACGAG
AGTTACGAGTACCCGGCCCGTTTTCCCGCGTCGGAGGCTGCCAACACGATAGTAACTG
AACTGGCCCAAACGTATTGATACGCCCCGGGGGCGGGCCTCTGGCGCGCCACTGGATC
AGGCCCGTGGCGTGCCCGCCTCGTCAGCGCCACCCATTGCTAAGCGCTGACAGTAATA
GACCCCTCCATAGTAGTTGCCGATGTAGGTCTGGTCACCGGCCGAAACGTATGAACGC
AGCACAGGGCAGGTACTACGGAGCGAAAGGTAGCTGATTGGCAGGGGCTGCTGGCGC
GCCTACCAACCTGTTCCTCCGCGCCTGCTGGAGCGACCAACTACGCCCCGCAGCGACC

GGTACCCGAGCAGTCTCTCAACTGGTTCGATGAGACTGTATACACCGTCGTTGGGACG
CGGACTAAACCGCCCCCTCATACCCACCCGCCCGTCCGGAGCGGAACGACTCGGCGGT
ACCGGCGTCAGGCCCCCTCGCCTAGGCTGCACCATTATGTTGGGAGGTGCGTCGACTG
GGGGCCGGCGACTTGAGCTCGGTCGTCCCGTGAAGCCCATCAGTCCCATGGAGACGTT
CTCTCCCATCCATTGGCCTCCGGGGCTCTCCACCAATCGCACCGGAGTCTTGTCTAGTG
TTAAATGTATTCATCGTGGGGTATAAACCGCCCCCGCGTAAGTGCGGCTCGAAGTCTG
GCCTCCCAGGCTGCTAGCAAACACGGAAGTGTGCCTCGTTTCGCTCAGAGGGGACAAG
ACACTCTCGACGGCTCTCAACAAGCCTAGTCCCCACGATACATACAACCGTGCCCGGT
GGGATTAATACGCCGGGGTCCCGTCGCGGGACATCTGGGATGGGAACACGTTCCGACC
ATATGCTCTCTGTCTGCATCTCTTGGGGAAATCCATTACGCTGCCGTTGACGTCGTTAA
CGTCACGCCACTAATACCGAACTAGGGGGGCCAAGCTATACCATGTAATCCGTGCGCC
TACCCACTGTTGCGATTCAGGGTACGGGGCTGTGAGTAATGTACACACTCCTGGTGTGA
GCTTGTCCTCCGGACTTGAATGGCTACTCGCCGGGCTGGGACTTAGCAAACCACCCTGA
CGCATTCCAGGCCTCTGATCAGATCACAATTTTGCTTTCACCAGTTCCCAATTACGGTT
CCGCATCGTCGGGAGGCCTTCGACTAGATCTACTCGCATGGTTCGAGCGGTAATATCG
GGTCTGAAATCCCCTGACCCAACGGGAGGCGGTACGTGTGATCAGGATCTGCACCTTG
CGAGAGCCAAGCGTAATGACGGTGGCTCTTCCACAAGTAAAAGCTCGGGCGCGAGCG
GCGGGCTGGATGGTCCCCCGAATGTTCCCCCCTATGCCGAAGGCTGAACTCGAGGCTG
AGCCGTGGGCGCGCACCGTTGCCACGCAGGTGCACCGCGAGGTCCCTTGAAGGTCCCG
TCACGAAG

>LR_ERR173183.1

GTCGGGATGCTTTCCCAACTCCGGCACGGCGCGGCGCCTTCGCGCACGGATCGGATAA
GCTTACCCGTGGACGGCTCGTCCTCAGAAACATTTCGTAGTCTTCTCGTTGGTTACTCC
ACTGCAGCGCCGCGTCTGCTTGGATGCTGAACCGCGAGAGCTATTCAAGTGCTTTACTA
CTCGACCGGGGGCAGCTTACAACCGTGGGATCGGCGTGGCCCACCTCCGGCCAACGAG
AGTTACGAGTACCCGGCCCGTTTTCCCGCGTCGGAGGCTGCCAACACGATAGTAGCTG
AACTGGCCCAGACGTATTGATACGCCCCGGGGGCGGGCCTCTGGCGCGCCACTGGATC
AGGCCCGTGGCGTGCCCGCCTCGTCAGCGCCACCCATTGCTAAGCGCTGACAGTAATA
GACCCCTCCATAGTAGTTGCCGATGTAGGTCTGGTCACCGGCCGAAACGTATGAACGC
AGCACAGGGCAGGTACTACGGAGCGAAAGGTAGCTGATTGGCAGGGGCTGCTGGCGC
GCCTACCAACCTGTTCCTCCGCGCCTGCTGGAGCGACCAACTACGCCCCGCAGCGACC
GGTACCCGAGCAGTCTCTCAACTGGTTCGATGAGACTGTATACACCGTCGTTGGGACG
CGGACTAAACCGCCCCCTCATACCCACCCGCCCGTCCGGAGCGGAACGACTCGGCGGT
ACCGGCGTCAGGCCCCCTCGCCTAGGCTGCACCATTATGTTGGGAGGTGCGTCGACTG
GGGGCCGGCGACTTGAGCTCGGTCGTCCCGTGAAGCCCATCAGTCCCATGGAGACGTT
CTCTCCCATCCATTGGCCTCCGGGGCTCTCCACCAATCGCACCGGAGTCTTGTCTAGTG
TTAAATGTATTCATCGTGGGGTATAAACCGCCCCCGCGTAAGTGCGGCTCGAAGTCTG
GCCTCCCAGGCTGCTAGCAAACACGGAAGTGTGCCTCGTTTCGCTCAGAGGGGACAAG
ACACTCTCGACGGCTCTCAACAAGCCTAGTCCCCACGATACATACAACCGTGCCCGGT
GGGATTAATACGCCGGGGTCCCGTCGCGGGACATCTGGGATGGGAACACGTTCCGACC
ATATGCTCTCTGTCTGCATCTCTTGGGGAAATCCATTACGCTGCCGTTGACGTCGTTAA
CGTCACGCCACTAATACCGAACTAGGGGGGCCAAACTATACCATGTTATCCGTGCGCC
TACCCACTGTTGCGATTCAGGGTACGGGGCTGTGAGTAGTGTACACACTCTTGATGGG
AGCTTATCCTCCGGACTTGAATGGCTACTCGCCGGGCTGGGACTTAGCAAACCACCCTG
ACGCATTCCAGGCCTCTGATCAGATCACAGTTTTGCTTTCACCAGTTCCCAATTACGGT
TCCGCATCGTCGGGAGGCCTTCGACTAGACCTACTCGCATGGTTCGAGCGGTAATATCG
GGTCTGAAATCCCCTGACCCAACGGGAGGCGGTACGTGTGATCGGAATCTGCACCTTG
CGAGAGCTAATCGTAATGCCGATGGCTCTTCCACAAGTGAGTGCTGGGGCGCGAGTGG
CGGACCAGATTGTCTTCCGAATGTTCCCCCCCGTGTCGAAGGCTGAACTCGAGGCGGA

GCCGTGAGCGCGCACCGTTGCCACGCAGGTGCACCGCGAGGCACCTCGAAGGTCCCGT
CATGAAG

>LR_ERR173183.2

GTCGGGATGCCTTCCCAACTCTGGCACGGCGCGGCGCCTTCGCGCACGGATCGGATAA
GCTTTCCCGTGGACGGCTCGTCCTCGGAAATATCTCGTAGTCTTCTCGTTGGTTGCTCCA
ACGAAGCGACGAATCTACTTGGATACTGAACCGCGAGAGCTATTCAAGTGCTCTACTA
CTCGACCGGGGGCAGCATACAGCCGTGGGATCGGCGCGGCCCACCTCCGGCCAACGAG
AGTTACGAGTACCCGGCCCGTTTTCCCGCGTCGGAGGCTGCCAACACGATAGTAACTG
AACTGGCCCAAACGTATTGATACGCCCCGGGGGCGGGCCTCTGGCGCGCCACTGGATC
AGGCCCGTGGCGTGCCCGCCTCGTCAGCGCCACCCATTGCTAAGCGCTGACAGTAATA
GACCCCTCCATAGTAGTTGCCGATGTTAATTCGGTCACCGGCCGAAACGTATGAACGC
AGCACAGGGCAGGTACTACGGAGCGAAAGGTAGCTGATTGGCAGGGGCTGCTGGCGC
GCCTACCAACCTGTTCCTCCGCGCCTGCTGGAGCGACCAACTACGCCCCGCAGCGACC
GGTACCCGAGCAGTCTCTCAACTGGTTCGATGAGACTGTATACACCGTCGTTGGGACG
CGGACTAAACAACCTCCTCATACCAATCCGCCCGTCCGGAGCGGAACGACTCGGCGGT
ACCGGCGTCAGGCCCCCTCGCCTAGGCTGCACCATTATGTTGGGAGGTGCGTCGACTG
GGGGCCGGCGACTTGAGCTCGGTCGTCCCGTGAAGCCCATCAGTCCCATGGAGACGTT
CTCTCCCATCCATTGGCCTCCGGGGCTCTCCACCACTCGCACCGGAGTCTTGTCTAGTG
TTAAATGTATTCATAGTGGGGTATAAACCGCCCCCGCGTGAGTGCGGCTCGAAGTCTG
GCCTCCCAGGCTGCTAGCTAACACGGAAGTGTGCCTCGTTTCGCTCAGAGGGGACGAG
ACACTCTCGACGGCTCTCAACAAGCCTAGTCCCCACGATACATACAACCGTGCCCGGT
GGGATTAATACGCCGGGGTCCCGTCGCGGGACATCTGGATGGGAACACGTTCCGGCC
ATATGCTCTCTGTCCGCATCTCTTGGGGAAATCCATTACGCTGCCGTTGACGTCGTTAA
CGTCACGCCACTAATACCGAACTAGGGGGACCAAACTATACCATGTAATCCGTGCGCC
TACCCACTGTTGCGATTCAGGGTACGGGGCTGTGAGTAATGTACACACTCCTGGTGTGA
GCTTGTCCTCCGGACTTGAATGGCTACTCGCCGGGCTGGGACTTAGCAAACCACCCTGA
CGCATTCCAGGCCTCTGATCAGATCACAATTTTGCTTTCACCAGTTCCCAATTACGGTT
CCGCATCGTCGGGAGGCCTTCGACTAGATCTACTCGCATGGTTCGAGCGGTAATATCG
GGTCTGAAATCCCCTGACCCAACGGGAGGCGGTACGTGTGATCAGGATCTGCACCTTG
CGAGAGCCAAGCGTAATGACGGTGGCTCTTCCACAAGTAAAAGCTCGGGCGCGAGCG
GCGGGCTGGATGGTCCCCCGAATGTTCCCCCCTATGCCGAAGGCTGAACTCGAGGCTG
AGCCGTGGGCGCGCACCGTTGCCACGCAGGTGCACCGCGAGGTCCCTTGAAGGTCCCG
TCATGAAG

>LR_ERR173184.1

GTCGGGATGCTTTCCCAACTCCGGCACGGCGCGGCGCCTTCGCGCACGGATCGGATAA
GCTTACCCGTGGACGGCTCGTCCTCAGAAACATTTCGTAGTCTTCTCGTTGGTTACTCC
ACTGCAGCGCCGCGTCTGCTTGGATGTTGAACCGCGAGAGCTATTCAAGTGCTTTACTA
CTCGACCGGGGGCAGCTTACAACCGTGGGATCGGCGTGGCCCACCTCCGGCCAACGAG
AGTTACGAGTACCCGGCCCGTTTTCCCGCGTCGGAGGCTGCCAACACGATAGTAGCTG
AACTGGCCCAGACGTATTGATACGCCCCGGGGGCGGGCCTCTGGCGCGCCACTGGATC
AGGCCCGTGGCGTGCCCGCCTCGTCAGCGCCACCCATTGCTAAGCGCTGACAGTAATA
GACCCCTCCATAGTAGTTGCCGATGTAGGTCTGGTCACCGGCCGAAACGTATGAACGC
AGCACAGGGCAGGTACTACGGAGCGAAAGGTAGCTGATTGGCAGGGGCTGCTGGCGC
GCCTACCAACCTGTTCCTCCGCGCCTGCTGGAGCGACCAACTACGCCCCGCAGCGACC
GGTACCCGAGCAGTCTCTCAACTGGTTCGATGAGACTGTATACACCGTCGTTGGGACG
CGGACTAAACAACCTCCTCATACCAATCCGCCCGTCCGGAGCGGAACGACTCGGCGGT
ACCGGCGTCAGGCCCCCTCGCCTAGGCTGCACCATTATGTTGGGAGGTGCGTCGACTG
GGGGCCGGCGACTTGAGCTCGGTCGTCCCGTGAAGCCCATCAGTCCCATGGAGACGTT

CTCTCCCATCCATTGGCCTCCGGGGCTCTCCACCAATCGCACCGGAGTCTTGTCTAGTG
TTAAATGTATTCATCGTGGGGTATAAACCGCCCCCGCGTGAGTGCGGCTCGAAGTCTG
GCCTCCCAGGCTGCTAGCTAACACGGAAGTGTGCCTCGTTTCGCTCAGAGGGGACGAG
ACACTCTCGACGGCTCTCAACAAGCCTAGTCCCCACGATACATACAACCGTGCCCGGT
GGGATTAATACGCCGGGGTCCTGTCGCGGGACATCTGGATGGGAACACGTTCCGGCC
ATATGTTCTCTGTCCGCATCTCTTGGGGAAATTCATTACGCTGCCGTTGACGTCGTTAA
CGTCACGCCACTAATACCGAACTAGGGGGACCAAACTATACCATGTAATCCGTGCGCC
TACCCACTGTTGCGATTCAGGGTACGGGGCTGTGAGTAATGTACACACTCCTGGTGTGA
GCTTGTCCTCCGGACTTGAATGGCTACTCGCCGGGCTGGGACTTAGCAAACCACCCTGA
CGCATTCCAGGCCTCTGATCAGATCACAATTTTGCTTTCACCAGTTCCCAATTACGGTT
CCGCATCGTCGGGAGGCCTTCGACTAGATCTACTCGCATGGTTCGAGCGGTAATATCG
GGTCTGAAATCCCCTGACCCAACGGGAGGCGGTACGTGTGATCAGGATCTGCACCTTG
CGAGAGCCAAGCGTAATGACGGTGGCTCTTCCACAAGTAAAAGCTCGGGCGCGAGCG
GCGGGCTGGATGGTCCCCCGAATGTTCCCCCCTATGCCGAAGGCTGAACTCGAGGCTG
AGCCGTGGGCGCGCACCGTTGCCACGCAGGTGCACCGCGAGGTCCCTTGAAGGTCCCG
TCACGAAG

>LR_ERR173184.2

GTCGGGATGCCTTCCCAACTCTGGCACGGCGCGGCGCCTTCGCGCACGGATCGGATAA
GCTTTCCCGTGGACGGCTCGTCCTCGGAAATATCTCGTAGTCTTCTCGTTGGTTGCTCCA
ACGAAGCGACGAATCTACTTGGATACTGAACCGCGAGAGCTATTCAAGTGCTCTACTA
CTCGACCGGGGGCAGCATACAGCCGTGGGATCGGCGCGGCCCACCTCCGGCCAACGAG
AGTTACGAGTACCCGGCCCGTTTTCCCGCGTCGGAGGCTGCCAACACGATAGTAACTG
AACTGGCCCAAACGTATTGATACGCCCCGGGGGCGGGCCTCTGGCGCGCCACTGGATC
AGGCCCGTGGCGTGCCCGCCTCGTCAGCGCCACCCATTGCTAAGCGCTGACAGTAATA
GACCCCTCCATAGTAGTTGCCGATGTTAATTCGGTCACCGGCCGAAACGTATGAACGC
AGCACAGGGCAGGTACTACGGAGCGAAAGGTAGCTGATTGGCAGGGGCTGCTGGCGC
GCCTACCAACCTGTTCCTCCGCGCCTGCTGGAGCGACCAACTACGCCCCGCAGCGACC
GGTACCCGAGCAGTCTCTCAACTGGTTCGATGAGACTGTATACACCGTCGTTGGGACG
CGGACTAAACAACCTCCTCATACCAATCCGCCCGTCCGGAGCGGAACGACTCGGCGGT
ACCGGCGTCAGGCCCCCTCGCCTAGGCTGCACCATTATGTTGGGAGGTGCGTCGACTG
GGGGCCGGCGACTTGAGCTCGGTCGTCCCGTGAAGCCCATCAGTCCCATGGAGACGTT
CTCTCCCATCCATTGGCCTCCGGGGCTCTCCACCAATCGCACCGGAGTCTTGTCTAGTG
TTAAATGTATTCATCGTGGGGTATAAACCGCCCCCGCGTAAGTGCGGCTCGAAGTCTG
GCCTCCCAGGCTGCTAGCAAACACGGAAGTGTGCCTCGTTTCGCTCAGAGGGGACAAG
ACACTCTCGACGGCTCTCAACAAGCCTAGTCCCCACGATACATACAACCGTGCCCGGT
GGGATTAATACGCCGGGGTCCCGTCGCGGGACATCTGGATGGGAACACGTTCCGACC
ATATGCTCTCTGTCTGCATCTCTTGGGGAAATCCATTACGCTGCCGTTGACGTCGTTAA
CGTCACGCCACTAATACCGAACTAGGGGGGCCAAACTATACCATGTTATCCGTGCGCC
TACCCACTGTTGCGATTCAGGGTACGGGGCTGTGAGTAGTGTACACACTCTTGATGGG
AGCTTATCCTCCGGACTTGAATGGCTACTCGCCGGGCTGGGACTTAGCAAACCACCCTG
ACGCATTCCAGGCCTCTGATCAGATCACAGTTTTGCTTTCACCAGTTCCCAATTACGGT
TCCGCATCGTCGGGAGGCCTTCGACTAGACCTACTCGCATGGTTCGGTCGGTAATATCG
GGTCTGAAATCCCTTGACCCAACGGGAGGCGGTACGTGTGATCGGAATCTGCACCTTG
CGAGAGCTAATCGTAATGCCGATGGCTCTTCCACAAGTGAGTGCTGGGGCGCGAGTGG
CGGACCAGATTGTCTTCCGAATGTTCCCCCCCGTGTCGAAGGCTGAACTCGAGGCGGA
GCCGTGAGCGCGCACCGTTGCCACGCAGGTGCACCGCGAGGCACCTCGAAGGTCCCGT
CATGAAG

>LW_ERR173185.1

GTCGGGATGCCTTCCCAACTCTGGCACGGCGCGGCGCCTTCGCGCACGGATCGGATAA
GCTTTCCCGTGGACGGCTCGTCCTCGGAAATATCTCGTAGTCTTCTCGTTGGTTGCTCCA
ACGAAGCGACGAATCTACTTGGATACTGAACCGCGAGAGCTATTCAAGTGCTCTACTA
CTCGACCGGGGGCAGCATACAGCCGTGGGATCGGCGCGGCCCACCTCCGGCCAACGAG
AGTTACGAGTACCCGGCCCGTTTTCCCGCGTCGGAGGCTGCCAACACGATAGTAACTG
AACTGGCCCAAACGTATTGATACGCCCCGGGGGCGGGCCTCTGGCGCGCCACTGGATC
AGGCCCGTGGCGTGCCCGCCTCGTCAGCGCCACCCATTGCTAAGCGCTGACAGTAATA
GACCCCTCCATAGTAGTTGCCGATGTTAATTCGGTCACCGGCCGAAACGTATGAACGC
AGCACAGGGCAGGTACTACGGAGCGAAAGGTAGCTGATTGGCAGGGGCTGCTGGCGC
GCCTACCAAACTGTTCCTCCGCGCCTGCTGGAGCGACCAACTACGCCCCGCAGCGACC
GGTACCCGAGCAGTCTCTCAACTGGTTCGATGAGACTGTATACACCGTCGTTGGGACG
CGGACTAAACAACCTCCTCATACCAATCCGCCCGTCCGGAGCGGAACGACTCGGCGGT
ACCGGCGTCAGGCCCCCTCGCCTAGGCTGCACCATTAGGTTGGGAGGTGCGTCGACTG
GGGGCCGGCGACTTGAGCTCGGTCGTCCCGTGAAGCTCATCAGTCCCATGGAGACGTT
CTCTCCCATCCATTGGCCTCCGGGGCTCTCCACCAATCGCACCGGAGTCTTGTCTAGTG
TTAAATGTATTCATCGTGGGGTATAAACCGCCCCCGCGTGGGTGCGGCTCGAAGTCTG
GCCTCCCAGGCTGCTAGCTAACACGGAAGTGTGCCTCGTTTCGCTCAGAGGGGACGAG
ACACTCTCGACGGCTCTCAACAAGCCTAGTCCCCACGATACATACAACCGTGCCCGGT
GGGATTAATACGCCGGGGTCCCGTCGCGGGACATCTGGGATGGGACCATGTTCCGGCC
GTATACTCCCTGTCCGTATCTCTTGGGGAAATCCATTACGCTGCCGTTGACGTCGTTAA
CGTCACGCCACTAATACCGAACTAGGGGGACCAAACTATACCATGTTATCCGTGCGCC
TACCCACTGTTGCGATTCAGGGTACGGGGCTGTGAGTAATGTACACACTCCTGGTGGG
AGCTTGTCCTCCGGACTTGAATGGCTACTCGCCGGGCTGGGACTTAGCAAACCACCCTG
ACGCATTCCAGGCCTCTGATCAGATCACAATTTTGCTTTCACCAGTTCCCAATTACGGT
TCCGCATCGTCGGGAGGCCTTCGACTAGATCTACTCGCATGGTTCGAGCGGTAATATCG
GGTCTGAAATCCCCTGACCCAACGGGAGGCGGTACGTGTGATCGGAATCTGCACCTTG
CGAGAGCTAATCGTAATGACGATTGCTCTTCCACAAGTGAGTGCTGGGGCGCGAGTGG
CGGACCAGATTGTCTTCCGAATGTTCCCCCCCGTGTCGAAGGCTGAACTCGAGGCGGA
GCCGTGAGCGCGCACCGTTGCCACGCAGGTGCACCGCGAGGCACCTCGAAGGTCCCGT
CATGAAG

>LW_ERR173185.2

GTCGGGATGCCTTCCCAACTCTGGCACGGCGCGGCGCCTTCGCGCACGGATCGGATAA
GCTTTCCCGTGGACGGCTCGTCCTCGGAAATATCTCGTAGTCTTCTCGTTGGTTGCTCCA
ACGAAGCGACGAATCTACTTGGATACTGAACCGCGAGAGCTATTCAAGTGCTCTACTA
CTCGACCGGGGGCAGCATACAGCCGTGGGATCGGCGCGGCCCACCTCCGGCCAACGAG
AGTTACGAGTACCCGGCCCGTTTTCCCGCGTCGGAGGCTGCCAACACGATAGTAACTG
AACTGGCCCAAACGTATTGATACGCCCCGGGGGCGGGCCTCTGGCGCGCCACTGGATC
AGGCCCGTGGCGTGCCCGCCTCGTCAGCGCCACCCATTGCTAAGCGCTGACAGTAATA
GACCCCTCCATAGTAGTTGCCGATGTTAATTCGGTCACCGGCCGAAACGTATGAATGC
AGCACAGGGCAGGTACTACGGAGCGAAAGGTAGCTGATTGGCAGGGGCTGCTGGCGC
GCCTACCAACCTGTTCCTCCGCGCCTGCTGGAGCGACCAACTACGCCCCGCAGCGACC
GGTACCCGAGCAGTCTCTCAACTGGTTCGATGAGACTGTATACACCGTCGTTGGGACG
CGGACTAAACAACCTCCTCATACCAATCCGCCCGTCCGGAGCGGAACGACTCGGCGGT
ACCGGCGTCAGGCCCCCTCGCCTAGGCTGCACCATTAGGTTGGGAGGTGCGTCGACTG
GGGGCCGGCGACTTGAGCTCGGTCGTCCCGTGAAGCCCATCAGTCCCATGGAGACGTT
CTCTCCCATCCATTAGCCTCCGGGGCTCTCCACCAATCGCACCGGAGTCTTGTCTAGTG
TTAAATGTATTCATCGTGGGGTATAAATCGCCCCCGCGTGAGTGCGGCTCGAAGTCTGG
CCTCCCAGGCTGCTAGCTAACACGGAAGTGTGCCTCGTTTCGCTCAGAGGGGACGAGA
CACTCTCGACGGCTCTCAACAAGCCTAGTCCCCACGATACATACAACCGTGCCCGGTG
GGATTAATACGCCGGGGTCCCGTCGCGGGACATCTGGGATGGGAACACGTTCCGGCCA

TATGCTCTCTGTCCGCATCTCTTGGGGAAATCCATTACGCTGCCGTTGACGTCGTTAAC
GTCACGCCACTAATACCGAACTAGGGGGACCAAACTATACCATGTTATCCGTGCGCCT
ACCCACTGTTGCGATTCAGGGTACGGGGCTGTGAGTAATGTACACACTCCTGGTGGGA
GCTTGTCCTCCGGACTTGAATGGCTACTCGCCGGGCTGGGACTTAGCAAACCACCCTGA
CGCATTCCAGGCCTCTGATCAGATCACAATTTTGCTTTCACCAGTTCCCAATTACGGTT
CCGCATCGTCGGGAGGCCTTCGACTAGATCTACTCGCATGGTTCGAGCGGTAATATCG
GGTCTGAAATCCCCTGACCCAACGGGAGGCGGTACGTGTGATCGGAATCTGCACCTTG
CGAGAGCTAATCGTAATGACGATTGCTCTTCCACAAGTGAGTGCTGGGGCGCGAGTGG
CGGACCAGATTGTCTTCCGAATGTTCCCCCCCGTGTCGAAGGCTGAACTCGAGGCGGA
GCCGTGAGCGCGCACCGTTGCCACGCAGGTGCACCGCGAGGCACCTCGAAGGTCCCGT
CATGAAG

>LW_ERR173186.1

GTCGGGATGCCTTCCCAACTCTGGCACGGCGCGGCGCCTTCGCGCACGGATCGGATAA
GCTTTCCCGTGGACGGCTCGTCCTCGGAAATATCTCGTAGTCTTCTCGTTGGTTGCTCCA
ACGAAGCGACGAATCTACTTGGATACTGAACCGCGAGAGCTATTCAAGTGCTCTACTA
CTCGACCGGGGGCAGCATACAGCCGTGGGATCGGCGCGGCCCACCTCCGGCCAACGAG
AGTTACGAGTACCCGGCCCGTTTTCCCGCGTCGGAGGCTGCCAACACGATAGTAACTG
AACTGGCCCAAACGTATTGATACGCCCCGGGGGCGGGCCTCTGGCGCGCCACTGGATC
AGGCCCGTGGCGTGCCCGCCTCGTCAGCGCCACCCGTTGCTAAGCGCTGACAGTAATA
GACCCCTCCATAGTAGTTGCCGATGTAGGTCTGGTCACCGGCCGAAACGTATGAACGC
AGCACAGGGCAGGTACTACGGAGCGAAAGGTAGCTGATTGGCAGGGGCTGCTGGCGC
GCCTACCAACCTGTTCCTCCGCGCCTGCTGGAGCGACCAACTACGCCCCGCAGCGACC
GGTACCCGAGCAGTCTCTCAACTGGTTCGATGAGACTGTATACACCGTCGTTGGGACG
CGGACTAAACCGCCCCCTCATACCCATCCGCCCGTCCGGAGCGGAACGACTCGGCGGT
ACCGGCGTCAGGCCCCCTCGCCTAGGCTGCACCATTATGTTGGGAGGTGCGTCGACTG
GGGGCCGGCGACTTGAGCTCGGTCGTCCCGTGAAGCCCATCAGTCCCATGGAGACGTT
CTCTCCCATCCATTGGCCTCCGGGGCTCTCCACCAATCGCACCGGAGTCTTGTCTAGTG
TTAAATGTATTCATCGTGGGGTATAAACCGCCCCCGCGTAAGTGCGGCTCGAAGTCTG
GCCTCCCAGGCTGCTAGCAAACACGGAAGTGTGCCTCGTCTCGCTCAGAGGGGACAAG
ACACTCTCGACGGCTCTCAACAAGCCTAGTCCCCACGATACATACAACCGTGCCCGGT
GGGATTAATACGCCGGGGTCCCGTCGCGGGACATCTGGATGGGAACACGTTCCGACC
ATATGCTCTCTGTCTGCATCTCTTGGGGAAATCCATTACGCTGCCGTTGACGTCGTTAA
CGTCACGCCACTAATACCGAACTAGGGGGGCCAAGCTATACCATGTAATCCGTGCGCC
TACCCACTGTTGCGATTCAGGGTACGGGGCTGTGAGTAATGTACACACTCCTGGTGTGA
GCTTGTCCTCCGGACTTGAATGGCTACTCGCCGGGCTGGGACTTAGCAAACCACCCTGA
CGCATTCCAGGCCTCTGATCAGATCACAATTTTGCTTTCACCAGTTCCCAATTACGGTT
CCGCATCGTCGGGAGGCCTTCGACTAGATCTACTCGCATGGTTCGAGCGGTAATATCG
GGTCTGAAATCCCCTGACTCAACGGGAGGCGGTACGTGTGATCAGGATCTGCACCTTG
CGAGAGCCAAGCGTAATGACGGTGGCTCTTCCACAAGTAAAAGCTCGGGCGCGAGCG
GCGGGCTGGATGGTCCTCCGAATGTTCCCCCCTATGCCGAAGGCTGAACTCGAGGCTG
AGCCGTGGGCGCGCACCGTTGCCACGCAGGTGCACCGCGAGGCCCCTTGAAGGTCCCG
TCACGAAG

>LW_ERR173186.2

GTCGGGATGCCTTCCCAACTCTGGCACGGCGCGGCGCCTTCGCGCACGGATCGGATAA
GCTTTCCCGTGGACGGCTCGTCCTCGGAAATATCTCGTAGTCTTCTCGTTGGTTGCTCCA
ACGAAGCGACGAATCTACTTGGATACTGAACCGCGAGAGCTATTCAAGTGCTCTACTA
CTCGACCGGGGGCAGCATACAGCCGTGGGATCGGCGCGGCCCACCTCCGGCCAACGAG
AGTTACGAGTACCCGGCCCGTTTTCCCGCGTCGGAGGCTGCCAACACGATAGTAACTG

AACTGGCCCAAACGTATTGATACGCCCCGGGGGCGGGCCTCTGGCGCGCCACTGGATC
AGGCCCGTGGCGTGCCCGCCTCGTCAGCGCCACCCATTGCTAAGCGCTGACAGTAATA
GACCCCTCCATAGTAGTTGCCGATGTTAATTCGGTCACCGGCCGAAACGTATGAATGC
AGCACAGGGCAGGTACTACGGAGCGAAAGGTAGCTGATTGGCAGGGGCTGCTGGCGC
GCCTACCAACCTGTTCCTCCGCGCCTGCTGGAGCGACCAACTACGCCCCGCAGCGACC
GGTACCCGAGCAGTCTCTCAACTGGTTCGATGAGACTGTATACACCGTCGTTGGGACG
CGGACTAAACAACCTCCTCATACCAATCCGCCCGTCCGGAGCGGAACGACTCGGCGGT
ACCGGCGTCAGGCCCCCTCGCCTAGGCTGCACCATTAGGTTGGGAGGTGCGTCGACTG
GGGGCCGGCGACTTGAGCTCGGTCGTCCCGTGAAGCTCATCAGTCCCATGGAGACGTT
CTCTCCCATCCATTGGCCTCCGGGGCTCTCCACCAATCGCACCGGAGTCTTGTCTAGTG
TTAAATGTATTCATCGTGGGGTATAAATCGCCCCCGCGTGAGTGCGGCTCGAAGTCTGG
CCTCCCAGGCTGCTAGCTAACACGGAAGTGTGCCTCGTTTCGCTCAGAGGGGACGAGA
CACTCTCGACGGCTCTCAACAAGCCTAGTCCCCACGATACATACAACCGTGCCCGGTG
GGATTAATACGCCGGGGTCCCGTCGCGGGACATCTGGGATGGGAACACGTTCCGGCCA
TATGCTCTCTGTCCGCATCTCTTGGGGAAATCCATTACGCTGCCGTTGACGTCGTTAAC
GTCACGCCACTAATACCGAACTAGGGGGACCAAACTATACCATGTTATCCGTGCGCCT
ACCCACTGTTGCGATTCAGGGTACGGGGCTGTGAGTAATGTACACACTCCTGGTGGGA
GCTTGTCCTCCGGACTTGAATGGCTACTCGCCGGGCTGGGACTTAGCAAACCACCCTGA
CGCATTCCAGGCCTCTGATCAGATCACAATTTTGCTTTCACCAGTTCCCAATTACGGTT
CCGCATCGTCGGGAGGCCTTCGACTAGATCTACTCGCATGGTTCGAGCGGTAATATCG
GGTCTGAAATCCCCTGACCCAACGGGAGGCGGTACGTGTGATCGGAATCTGCACCTTG
CGAGAGCTAATCGTAATGACGATTGCTCTTCCACAAGTGAGTGCTGGGGCGCGAGTGG
CGGACCAGATTGTCTTCCGAATGTTCCCCCCCGTGTCGAAGGCTGAACTCGAGGCGGA
GCCGTGAGCGCGCACCGTTGCCACGCAGGTGCACCGCGAGGCACCTCGAAGGTCCCGT
CATGAAG

>LW_ERR173187.1

GTCGGGATGCCTTCCCAACTCTGGCACGGCGCGGCGCCTTCGCGCACGGATCGGATAA
GCTTTCCCGTGGACGGCTCGTCCTCGGAAATATCTCGTAGTCTTCTCGTTGGTTGCTCCA
ACGAAGCGACGAATCTACTTGGATACTGAACCGCGAGAGCTATTCAAGTGCTCTACTA
CTCGACCGGGGGCAGCATACAGCCGTGGGATCGGCGCGGCCCACCTCCGGCCAACGAG
AGTTACGAGTACCCGGCCCGTTTTCCCGCGTCGGAGGCTGCCAACACGATAGTAACTG
AACTGGCCCAAACGTATTGATACGCCCCGGGGGCGGGCCTCTGGCGCGCCACTGGATC
AGGCCCGTGGCGTGCCCGCCTCGTCAGCGCCACCCATTGCTAAGCGCTGACAGTAATA
GACCCCTCCATAGTAGTTGCCGATGTTAATTCGGTCACCGGCCGAAACGTATGAATGC
AGCACAGGGCAGGTACTACGGAGCGAAAGGTAGCTGATTGGCAGGGGCTGCTGGCGC
GCCTACCAACCTGTTCCTCCGCGCCTGCTGGAGCGACCAACTACGCCCCGCAGCGACC
GGTACCCGAGCAGTCTCTCAACTGGTTCGATGAGACTGTATACACCGTCGTTGGGACG
CGGACTAAACAACCTCCTCATACCAATCCGCCCGTCCGGAGCGGAACGACTCGGCGGT
ACCGGCGTCAGGCCCCCTCGCCTAGGCTGCACCATTAGGTTGGGAGGTGCGTCGACTG
GGGGCCGGCGACTTGAGCTCGGTCGTCCCGTGAAGCTCATCAGTCCCATGGAGACGTT
CTCTCCCATCCATTGGCCTCCGGGGCTCTCCACCAATCGCACCGGAGTCTTGTCTAGTG
TTAAATGTATTCATCGTGGGGTATAAATCGCCCCCGCGTGAGTGCGGCTCGAAGTCTGG
CCTCCCAGGCTGCTAGCTAACACGGAAGTGTGCCTCGTTTCGCTCAGAGGGGACGAGA
CACTCTCGACGGCTCTCAACAAGCCTAGTCCCCACGATACATACAACCGTGCCCGGTG
GGATTAATACGCCGGGGTCCCGTCGCGGGACATCTGGGATGGGAACACGTTCCGGCCA
TATGCTCTCTGTCCGCATCTCTTGGGGAAATCCATTACGCTGCCGTTGACGTCGTTAAC
GTCACGCCACTAATACCGAACTAGGGGGACCAAACTATACCATGTTATCCGTGCGCCT
ACCCACTGTTGCGATTCAGGGTACGGGGCTGTGAGTAATGTACACACTCCTGGTGGGA
GCTTGTCCTCCGGACTTGAATGGCTACTCGCCGGGCTGGGACTTAGCAAACCACCCTGA
CGCATTCCAGGCCTCTGATCAGATCACAATTTTGCTTTCACCAGTTCCCAATTACGGTT

CCGCATCGTCGGGAGGCCTTCGACTAGATCTACTCGCATGGTTCGAGCGGTAATATCG
GGTCTGAAATCCCCTGACCCAACGGGAGGCGGTACGTGTGATCGGAATCTGCACCTTG
CGAGAGCTAATCGTAATGACGATTGCTCTTCCACAAGTGAGTGCTGGGGCGCGAGTGG
CGGACCAGATTGTCTTCCGAATGTTCCCCCCCGTGTCGAAGGCTGAACTCGAGGCGGA
GCCGTGAGCGCGCACCGTTGCCACGCAGGTGCACCGCGAGGCACCTCGAAGGTCCCGT
CATGAAG

>LW_ERR173187.2

GTCGGGATGCCTTCCCAACTCTGGCACGGCGCGGCGCCTTCGCGCACGGATCGGATAA
GCTTTCCCGTGGACGGCTCGTCCTCGGAAATATCTCGTAGTCTTCTCGTTGGTTGCTCCA
ACGAAGCGACGAATCTACTTGGATACTGAACCGCGAGAGCTATTCAAGTGCTCTACTA
CTCGACCGGGGGCAACATACAGCCGTGGGATCGGCGCGGCCCACCTCCGGCCAACGAG
AGTTACGAGTACCCGGCCCGTTTTCCCGCGTCGGAGGCTGCCAACACGATAGTAACTG
AACTGGCCCAAACGTATTGATACGCCCCGGGGGCGGGCCTCTGGCGCGCCACTGGATC
AGGCCCGTGGCGTGCCCGCCTCGTCAGCGCCACCCATTGCTAAGCGCTGACAGTAATA
GACCCCTCCATAGTAGTTGCCGATGTAGGTCTGGTCACCGGCCGAAACGTATGAACGC
AGCACAGGGCAGGTACTACGGAGCGAAAGGTAGCTGATTGGCAGGGGCTGCTGGCGC
GCCTACCAACCTGTTCCTCCGCGCCTGTCGGAGCGACCAACTACGCCCCGCAGCGACC
GGTACCCGAGCAGTCTCTCAACTGGTTCGATGAGACTGTATACACCGTCGTTGGGACG
CGGACTAAACCGCCCCCTCATACCCACCCGCCCGTCCGGAGCGGAACGACTCGGCGGT
ACCGGCGTCAGGCCCCCTCGCCTAGGCTGCACCATTATGTTGGGAGGTGCGTCGACTG
GGGGCCGGCGACTTGAGCTCGGTCGTCCCGTGAAGCCCATCAGTCCCATGGAGACGTT
CTCTCCCATCCATTGGCCTCCGGGGCTCTCCACCAATCGCACCGGAGTCTTGTCTAGTG
TTAAATGTATTCATCGTGGGGTATAAACCGCCCCCGCGTAAGTGCGGCTCGAAGTCTG
GCCTCCCAGGCTGCTAGCAAACACGGAAGTGCGCCTCGTCTCGCTCAGAGGGGACAAG
ACACTCTCGACGGCTCTCAACAAGCCTAGTCCCCACGATACATACAACCGTGCCCGGT
GGGATTAATACGCCGGGGTCCCGTCGCGGGACATCTGGGATGGGAACACGTTCCGACC
ATATGCTCTCTGTCTGCATCTCTTGGGGAAATCCATTACGCTGCCGTTGACGTCGTTAA
CGTCACGCCACTAATACCGAACTAGGGGGGCCAAGCTATACCATGTAATCCGTGCGCC
TACCCACTGTTGCGATTCAGGGTACGGGGCTGTGAGTAATGTACACACTCCTGGTGTGA
GCTTGTCCTCCGGACTTGAATGGCTACTCGCCGGGCTGGGACTTAGCAAACCACCCTGA
CGCATTCCAGGCCTCTGATCAGATCACAATTTTGCTTTCACCAGTTCCCAATTACGGTT
CCGCATCGTCGGGAGGCCTTCGACTAGATCTACTCGCATGGTTCGAGCGGTAATATCG
GGTCTGAAATCCCCTGACCCAACGGGAGGCGGTACGTGTGATCAGGATCTGCACCTTG
CGAGAGCCAAGCGTAATGACGGTGGCTCTTCCACAAGTAAAAGCTCGGGCGCGAGCG
GCGGGCTGGATGGTCCCCCGAATGTTCCCCCCTATGCCGAAGGCTGAACTCGAGGCTG
AGCCGTGGGCGCGCACCGTTGCCACGCAGGTGCACCGCGAGGTCCCTTGAAGGTCCCG
TCACGAAG

>LW_ERR173188.1

GTCGGGATGCCTTCCCAACTCTGGCACGGCGCGGCGCCTTCGCGCACGGATCGGATAA
GCTTTCCCGTGGACGGCTCGTCCTCGGAAATATCTCGTAGTCTTCTCGTTGGTTGCTCCA
ACGAAGCGACGAATCTACTTGGATACTGAACCGCGAGAGCTATTCAAGTGCTCTACTA
CTCGACCGGGGGCAGCATACAGCCGTGGGATCGGCGCGGCCCACCTCCGGCCAACGAG
AGTTACGAGTACCCGGCCCGTTTTCCCGCGTCGGAGGCTGCCAACACGATAGTAACTG
AACTGGCCCAAACGTATTGATACGCCCCGGGGGCGGGCCTCTGGCGCGCCACTGGATC
AGGCCCGTGGCGTGCCCGCCTCGTCAGCGCCACCCGTTGCTAAGCGCTGACAGTAATA
GACCCCTCCATAGTAGTTGCCGATGTAGGTCTGGTCACCGGCCGAAACGTATGAACGC
AGCACAGGGCAGGTACTACGGAGCGAAAGGTAGCTGATTGGCAGGGGCTGCTGGCGC
GCCTACCAACCTGTTCCTCCGCGCCTGCTGGAGCGACCAACTACGCCCCGCAGCGACC

GGTACCCGAGCAGTCTCTCAACTGGTTCGATGAGACTGTATACACCGTCGTTGGGACG
CGGACTAAACCGCCCCCTCATACCCACCCGCCCGTCCGGAGCGGAACGACTCGGCGGT
ACCGGCGTCAGGCCCCCTCGCCTAGGCTGCACCATTATGTTGGGAGGTGCGTCGACTG
GGGGCCGGCGACTTGAGCTCGGTCGTCCCGTGAAGCCCATCAGTCCCATGGAGACGTT
CTCTCCCATCCATTGGCCTCCGGGGCTCTCCACCAATCGCACCGGAGTCTTGTCTAGTG
TTAAATGTATTCATCGTGGGGTATAAACCGCCCCCGCGTAAGTGCGGCTCGAAGTCTG
GCCTCCCAGGCTGCTAGCAAACACGGAAGTGCGCCTCGTCTCGCTCAGAGGGGACAAG
ACACTCTCGACGGCTCTCAACAAGCCTAGTCCCCACGATACATACAACCGTGCCCGGT
GGGATTAATACGCCGGGGTCCCGTCGCGGGACATCTGGGATGGGAACACGTTCCGACC
ATATGCTCTCTGTCTGCATCTCTTGGGGAAATCCATTACGCTGCCGTTGACGTCGTTAA
CGTCACGCCACTAATACCGAACTAGGGGGGCCAAGCTATACCATGTAATCCGTGCGCC
TACCCACTGTTGCGATTCAGGGTACGGGGCTGTGAGTAATGTACACACTCCTGGTGTGA
GCTTGTCCTCCGGACTTGAATGGCTACTCGCCGGGCTGGGACTTAGCAAACCACCCTGA
CGCATTCCAGGCCTCTGATCAGATCACAATTTTGCTTTCACCAGTTCCCAATTACGGTT
CCGCATCGTCGGGAGGCCTTCGACTAGATCTACTCGCATGGTTCGAGCGGTAATATCG
GGTCTGAAATCCCCTGACCCAACGGGAGGCGGTACGTGTGATCAGGATCCGCACCTTG
CGAGAGCCAAGCGTAATGACGGTGGCTCTTCCACAAGTAAAAGCTCGGGCGCGAGCG
GCGGGCTGGATGGTCCCCCGAATGTTCCCCCCTATGCCGAAGGCTGAACTCGAGGCTG
AGCCGTGGGCGCGCCCCGTTGCCACGCAGGTGCACCGCGAGGTCCCTTGAAGGTCCCG
TCACGAAG

>LW_ERR173188.2

GTCGGGATGCCTTCCCAACTCTGGCACGGCGCGGCGCCTTCGCGCACGGATCGGATAA
GCTTTCCCGTGGACGGCTCGTCCTCGGAAATATCTCGTAGTCTTCTCGTTGGTTGCTCCA
ACGAAGCGACGAATCTACTTGGATACTGAACCGCGAGAGCTATTCAAGTGCTCTACTA
CTCGACCGGGGGCAGCATACAGCCGTGGGATCGGCGCGGCCCACCTCCGGCCAACGAG
AGTTACGAGTACCCGGCCCGTTTTCCCGCGTCGGAGGCTGCCAACACGATAGTAACTG
AACTGGCCCAAACGTATTGATACGCCCCGGGGGCGGGCCTCTGGCGCGCCACTGGATC
AGGCCCGTGGCGTGCCCGCCTCGTCAGCGCCACCCATTGCTAAGCGCTGACAGTAATA
GACCCCTCCATAGTAGTTGCCGATGTAGGTCTGGTCACCGGCCGAAACGTATGAACGC
AGCACAGGGCAGGTACTACGGAGCGAAAGGTAGCTGATTGGCAGGGGCTGCTGGCGC
GCCTACCAACCTGTTCCTCCGCGCCTGCTGGAGCGACCAACTACGCCCCGCAGCGACC
GGTACCCGAGCAGTCTCTCAACTGGTTCGATGAGACTGTATACACCGTCGTTGGGACG
CGGACTAAACCGCCCCCTCATACCCACCCGCCCGTCCGGAGCGGAACGACTCGGCGGT
ACCGGCGTCAGGCCCCCTCGCCTAGGCTGCACCATTATGTTGGGAGGTGCGTCGACTG
GGGGCCGGCGACTTGAGCTCGGTCGTCCCGTGAAGCCCATCAGTCCCATGGAGACGTT
CTCTCCCATCCATTGGCCTCCGGGGCTCTCCACCAATCGCACCGGAGTCTTGTCTAGTG
TTAAATGTATTCATCGTGGGGTATAAACCGCCCCCGCGTAAGTGCGGCTCGAAGTCTG
GCCTCCCAGGCTGCTAGCAAACACGGAAGTGTGCCTCGTTTCGCTCAGAGGGGACAAG
ACACTCTCGACGGCTCTCAACAAGCCTAGTCCCCACGATACATACAACCGTGCCCGGT
GGGATTAATACGCCGGGGTCCCGTCGCGGGACATCTGGGATGGGAACACGTTCCGACC
ATATGCTCTCTGTCTGCATCTCTTGGGGAAATCCATTACGCTGCCGTTGACGTCGTTAA
CGTCACGCCACTAATACCGAACTAGGGGGGCCAAGCTATACCATGTAATCCGTGCGCC
TACCCACTGTTGCGATTCAGGGTACGGGGCTGTGAGTAATGTACACACTCCTGGTGTGA
GCTTGTCCTCCGGACTTGAATGGCTACTCGCCGGGCTGGGACTTAGCAAACCACCCTGA
CGCATTCCAGGCCTCTGATCAGATCACAATTTTGCTTTCACCAGTTCCCAATTACGGTT
CCGCATCGTCGGGAGGCCTTCGACTAGATCTACTCGCATGGTTCGAGCGGTAATATCG
GGTCTGAAATCCCCTGACCCAACGGGAGGCGGTACGTGTGATCAGGATCTGCACCTTG
CGAGAGCCAAGCGTAATGACGGTGGCTCTTCCACAAGTAAAAGCTCGGGCGCGAGCG
GCGGGCTGGATGGTCCCCCGAATGTTCCCCCCTATGCCGAAGGCTGAACTCGAGGCTG

AGCCGTGGGCGCGCACCGTTGCCACGCAGGTGCACCGCGAGGTCCCTTGAAGGTCCCG
TCACGAAG

>LW_ERR173189.1

GTCGGGATGCCTTCCCAACTCTGGCACGGCGCGGCGCCTTCGCGCACGGATCGGATAA
GCTTTCCCGTGGACGGCTCGTCCTCGGAAATATCTCGTAGTCTTCTCGTTGGTTGCTCCA
ACGAAGCGACGAATCTACTTGGATACTGAACCGCGAGAGCTATTCAAGTGCTCTACTA
CTCGACCGGGGGCAACATACAGCCGTGGGATCGGCGCGGCCCACCTCCGGCCAACGAG
AGTTACGAGTACCCGGCCCGTTTTCCCGCGTCGGAGGCTGCCAACACGATAGTAACTG
AACTGGCCCAAACGTATTGATACGCCCCGGGGGCGGGCCTCTGGCGCGCCACTGGATC
AGGCCCGTGGCGTGCCCGCCTCGTCAGCGCCACCCATTGCTAAGCGCTGACAGTAATA
GACCCCTCCATAGTAGTTGCCGATGTTAATTCGGTCACCGGCCGAAACGTATGCACTTA
GCATAGGGCAGGTACTACAAAGCGAGAGGTGGACGATTGGCAGAGGCTGCTGGCGAG
CCTACCAACCTGTTCCTCCGCGCCTGCTGGAGCGACCAACTACGCCCCGCAGCGACCG
GTACTTAAGCTGTCTCTCGACTGGTCCGATTAGATTGTATAAACAGTCGTTAAGACGCG
GACTAAACAACCTCCTCATACCAGTCCGCCCGTCCGGAGCGGAACGACTCGGCGGTAC
CGGCGTCAGGCCCCCTCGCCTAGGCTACACCATTATGTTGGGAGGTGCGTCGACTGGG
GGCCGGCGACTTGAGCTCGGTCGTCCCGTGAAGCCCATCAGTCCCATGGAGACGTTCT
CTCCCATCCATTGGCCTCCGGGGCTCTCCACCAATCGCACCGGAGTCTTGTCTAGTGTT
AAATGTATTCATCGTGGGGTATAAACCGCCCCCGCGTAAGTGCGGCTCGAAGTCTGGC
CTCCCAGGCTGCTAGCAAACACGGAAGTGTGCCTCGTTTCGCTCAGAGGGGACAAGAC
ACTCTCGACGGCTCTCAACAAGCCTAGTCCCCACGATACATACAACCGTGCCCGGTGG
GATTAATACGCCGGGGTCCCGTCGCGGGACATCTGGGATGGGAACACGTTCCGACCAT
ATGCTCTCTGTCTGCATCTCTTGGGGAAATCCATTACGCTGCCGTTGACGTCGTTAACG
TCACGCCACTAATACCGAACTAGGGGGGCCAAACTATACCATGTTATCCGTGCGCCTA
CCCACTGTTGCGATTCAGGGTACGGGGCTGTGAGTAGTGTACACACTCTTGATGGGAG
CTTATCCTCCGGACTTGAATGGCTACTCGCCGGGCTGGGACTTAGCAAACCACCCTGAC
GCATTCCAGGCCTCTGATCAGATCACAGTTTTGCTTTCACCAGTTCCCAATTACGGTTC
CGCATCGTCGGGAGGCCTTCGACTAGACCTACTCGCATGGTTCGGTCGGTAATATCGG
GTCTGAAATCCCTTGACCCAACGGGAGGCGGTACGTGTGATCGGAATCTGCACCTTGC
GAGAGCTAATCGTAATGCCGATGGCTCTTCCACAAGTGAGTGCTGGGGCGCGAGTGGC
GGACCAGATTGTCTTCCGAATGTTCCCCCCCGTGTCGAAGGCTGAACTCGAGGCGGAG
CCGTGAGCGCGCACCGTTGCCACGCAGGTGCACCGCGAGGCACCTCGAAGGTCCCGTC
ATGAAG

>LW_ERR173189.2

GTCGGGATGCCTTCCCAACTCTGGCACGGCGCGGCGCCTTCGCGCACGGATCGGATAA
GCTTTCCCGTGGACGGCTCGTCCTCGGAAATATCTCGTAGTCTTCTCGTTGGTTGCTCCA
ACGAAGCGACGAATCTACTTGGATACTGAACCGCGAGAGCTATTCAAGTGCTCTACTA
CTCGACCGGGGGCAGCATACAGCCGTGGGATCGGCGCGGCCCACCTCCGGCCAACGAG
AGTTACGAGTACCCGGCCCGTTTTCCCGCGTCGGAGGCTGCCAACACGATAGTAACTG
AACTGGCCCAAACGTATTGATACGCCCCGGGGGCGGGCCTCTGGCGCGCCACTGGATC
AGGCCCGTGGCGTGCCCGCCTCGTCAGCGCCACCCATTGCTAAGCGCTGACAGTAATA
GACCCCTCCATAGTAGTTGCCGATGTTAATTCGGTCACCGGCCGAAACGTATGAACGC
AGCACAGGGCAGGTACTACGGAGCGAAAGGTAGCTGATTGGCAGGGGCTGCTGGCGC
GCCTACCAACCTGTTCCTCCGCGCCTGCTGGAGCGACCAACTACGCCCCGCAGCGACC
GGTACCCGAGCAGTCTCTCAACTGGTTCGATGAGACTGTATACACCGTCGTTGGGACG
CGGACTAAACAACCTCCTCATACCAATCCGCCCGTCCGGAGCGGAACGACTCGGCGGT
ACCGGCGTCAGGCCCCCTCGCCTAGGCTGCACCATTATGTTGGGAGGTGCGTCGACTG
GGGGCCGGCGACTTGAGCTCGGTCGTCCCGTGAAGCCCATCAGTCCCATGGAGACGTT

CTCTCCCATCCATTAGCCTCCGGGGCTCTCCACCAATCGCACCGGAGTCTTGTCTAGTG
TTAAATGTATTCATCGTGGGGTATAAACCGCCCCCGCGTGGGTGCGGCTCGAAGTCTG
GCCTCCCAGGCTGCTAGCTAACACGGAAGTGTGCCTCGTTTCGCTCAGAGGGGACGAG
ACACTCTCGACGGCTCTCAACAAGCCTAGTCCCCACGATACATACAACCGTGCCCGGT
GGGATTAATACACCGGGGTCCCGTCGCGGGACGTCTGGGATGGGACCATGTTCCGGCC
GTATACTCCCTGTTCGTGCCCCTCGGAGAAATCCATTACGCTGCCGTTGACGTCGTTAA
CGTCACGCCACTAACACCGAACTAGGGGGACCAAACTATACCATGTTATCCGTGCGCC
TACCCACTGTTGCGATTCAGGGTACGGGGCTGTGAGTAATGTACACACTCCTGGTGGG
AGCTTGTCCTCCGGACTTGAATGGCTACTCGCCGGGCTGGGACTTAGCAAACCACCCTG
ACGCATTCCAGGCCTCTGATCAGATCACAATTTTGCTTTCACCAGTTCCCAATTACGGT
TCCGCATCGTCGGGAGGCCTTCGACTAGATCTACTCGCATGGTTCGAGCGGTAATATCG
GGTCTGAAATCCCCTGACCCAACGGGAGGCGGTACGTGTGATCGGAATCTGCACCTTG
CGAGAGCTAATCGTAATGACGATTGCTCTTCCACAAGTGAGTGCTGGGGCGCGAGTGG
CGGACCAGATTGTCTTCCGAATGTTCCCCCCCGTGTCGAAGGCTGAACTCGAGGCGGA
GCCGTGAGCGCGCACCGTTGCCACGCAGGTGCACCGCGAGGCACCTCGAAGGTCCCGT
CATGAAG

>LW_ERR173190.1

GTCGGGATCCCTTCTCGACTCTGGCACGGCGCGGCGGCTTCGCGCACGGATCGGATAA
GCTTTCCCGTGGACGGCTCGTCCTCGGAAATATCTCGTAGTCTTCTCGTTGGTTGCTCCA
ACGAAGCGACGAATCTACTTGGATACTGAACCGCGAGAGCTATTCAAGTGCTCTACTA
CTCGACCGGGGGCAGCATACAGCCGTGGGATCGGCGCGGCCCACCTCCGGCCAACGAG
AGGTACGAGTACCCGGCCCGTTTTCCCGCGTCGGAGGCTGCCAACACGATAGTAGCTG
AACTGGCCCAAACGTCTTGATACGCCCCGGGGGCGGGCCTCTGGCGCGCCACTGGATC
AGGCCCGTGGCGTGCCCGCCTCGTCAGCGCCACCCATTGCTAAGCGCTGACAGTAATA
GACCCCTCCATAGTAGTTGCCGATGTTGATTCGGTCACCGGCCGAAACGTGCGCGCTCA
GCACAGGGCAGGTACCACGGAGCAAAAGGTGGATGATTGGCAGGGACTGCTGGCGAG
CCTACCAACCTGTTCCTCCGCGCCTGCTGGAGCGACCAACTACGCCCCGCAGCGACCG
GTACTTGAGCTGTCTCTCGACTGGTCCGATTAGATTGTATAAACAGTCGTTAAGACGCG
GACTAAACAGCCCCCTCATACCCACCCGCCCGTCCGGAGCGGAACGACTCGGCGGTAC
CGGCGTCAGGCCCCCTCGCCTAGGCTGCACCATTATGTTGGGAGGTGCGTCGACTGGG
GGCCGGCGACTTGAGCTCGGTCGTCCCGTGAAGCCCATCAGTCCCATGGAGACGTTCT
CTCCCATCCATTAGCCTCCGGGGCTCTCCACCAATCGCACCGGAGTCTTGTCTAGTGTT
AAATGTATTCATCGTGGGGTATAAACCGCCCCCGCGTGGGTGCGGCTCGAAGTCTGGC
CTCCCAGGCTGCTAGCTAACACGGAAGTGTGCCTCGTTTCGCTCAGAGGGGACGAGAC
ACTCTCGACGGCTCTCAACAAGCCTAGTCCCCACGATACATACAACCGTGCCCGGTGG
GATTAATACGCCGGGGTCCTGTCGCGGGACATCTGGGATGGGAACACGTTCCGGCCAT
ATGTTCTCTGTCCGCATCTCTTGGGGAAATTCATTACGCTGCCGTTGACGTCGTTAACG
TCACGCCACTAATACCGAACTAGGGGGACCAAACTATACCATGTAATCCGTGCGCCTA
CCCACTGTTGCGATTCAGGGTACGGGGCTGTGAGTAATGTACACACTCCTGGTGTGAG
CTTGTCCTCCGGACTTGAATGGCTACTCGCCGGGCTGGGACTTAGCAAACCACCCTGAC
GCATTCCAGGCCTCTGATCAGATCACAATTTTGCTTTCACCAGTTCCCAATTACGGTTC
CGCATCGTCGGGAGGCCTTCGACTAGATCTACTCGCATGGTTCGATCGGTAATATCGGG
TCTGAAATCCCCTGACCCAACGGGAGGCGGTACGTGTGATCAGGATCTGCACCTTGCG
AGAGCCAAGCGTAATGACGGTGGCTCTTCCACAAGTAAAAGCTCGGGCGCGAGCGGC
GGGCTGGATGGTCCCCCGAATGTTCCCCCCTATGCCGAAGGCTGAACTCGAGGCGGAG
CCGTGGGCGCGCACCGTTGCCACGCAGCTGCACCGCGAGGCACCTCGAAGGTCCCGCC
ATGAAG

>LW_ERR173190.2

GTCGGGATGCCTTCCCAACTCTGGCACGGCGCGGCGCCTTCGCGCACGGATCGGATAA
GCTTTCCCGTGGACGGCTCGTCCTCGGAAATATCTCGTAGTCTTCTCGTTGGTTGCTCCA
ACGAAGCGACGAATCTACTTGGATACTGAACCGCGAGAGCTATTCAAGTGCTCTACTA
CTCGACCGGGGGCAGCATACAGCCGTGGGATCGGCGCGGCCCACCTCCGGCCAACGAG
AGTTACGAGTACCCGGCCCGTTTTCCCGCGTCGGAGGCTGCCAACACGATAGTAACTG
AACTGGCCCAAACGTATTGATACGCCCCGGGGGCGGGCCTCTGGCGCGCCACTGGATC
AGGCCCGTGGCGTGCCCGCCTCGTCAGCGCCACCCATTGCTAAGCGCTGACAGTAATA
GACCCCTCCATAGTAGTTGCCGATGTTAATTCGGTCACCGGCCGAAACGTATGAATGC
AGCACAGGGCAGGTACTACGGAGCGAAAGGTAGCTGATTGGCAGGGGCTGCTGGCGC
GCCTACCAACCTGTTCCTCCGCGCCTGCTGGAGCGACCAACTACGCCCCGCAGCGACC
GGTACCCGAGCAGTCTCTCAACTGGTTCGATGAGACTGTATACACCGTCGTTGGGACG
CGGACTAAACAACCTCCTCATACCAATCCGCCCGTCCGGAGCGGAACGACTCGGCGGT
ACCGGCGTCAGGCCCCCTCGCCTAGGCTGCACCATTAGGTTGGGAGGTGCGTCGACTG
GGGGCCGGCGACTTGAGCTCGGTCGTCCCGTGAAGCTCATCAGTCCCATGGAGACGTT
CTCTCCCATCCATTGGCCTCCGGGGCTCTCCACCAATCGCACCGGAGTCTTGTCTAGTG
TTAAATGTATTCATCGTGGGGTATAAATCGCCCCCGCGTGAGTGCGGCTCGAAGTCTGG
CCTCCCAGGCTGCTAGCTAACACGGAAGTGTGCCTCGTTTCGCTCAGAGGGGACGAGA
CACTCTCGACGGCTCTCAACAAGCCTAGTCCCCACGATACATACAACCGTGCCCGGTG
GGATTAATACGCCGGGGTCCCGTCGCGGGACATCTGGGATGGGAACACGTTCCGGCCA
TATGCTCTCTGTCCGCATCTCTTGGGGAAATCCATTACGCTGCCGTTGACGTCGTTAAC
GTCACGCCACTAATACCGAACTAGGGGGACCAAACTATACCATGTTATCCGTGCGCCT
ACCCACTGTTGCGATTCAGGGTACGGGGCTGTGAGTAATGTACACACTCCTGGTGGGA
GCTTGTCCTCCGGACTTGAATGGCTACTCGCCGGGCTGGGACTTAGCAAACCACCCTGA
CGCATTCCAGGCCTCTGATCAGATCACAATTTTGCTTTCACCAGTTCCCAATTACGGTT
CCGCATCGTCGGGAGGCCTTCGACTAGATCTACTCGCATGGTTCGAGCGGTAATATCG
GGTCTGAAATCCCCTGACCCAACGGGAGGCGGTACGTGTGATCGGAATCTGCACCTTG
CGAGAGCTAATCGTAATGACGATTGCTCTTCCACAAGTGAGTGCTGGGGCGCGAGCGG
CGGACCAGATTGTCTTCCGAATGTTCCCCCCCGTGTCGAAGGCTGAACTCGAGGCGGA
GCCGTGAGCGCGCACCGTTGCCACGCAGGTGCACCGCGAGGCACCTCGAAGGTCCCGT
CATGAAG

>LW_ERR173191.1

GTCGGGATGCCTTCCCAACTCTGGCACGGCGCGGCGCCTTCGCGCACGGATCGGATAA
GCTTTCCCGTGGACGGCTCGTCCTCGGAAATATCTCGTAGTCTTCTCGTTGGTTGCTCCA
ACGAAGCGACGAATCTACTTGGATACTGAACCGCGAGAGCTATTCAAGTGCTCTACTA
CTCGACCGGGGGCAGCATACAGCCGTGGGATCGGCGCGGCCCACCTCCGGCCAACGAG
AGTTACGAGTACCCGGCCCGTTTTCCCGCGTCGGAGGCTGCCAACACGATAGTAACTG
AACTGGCCCAAACGTATTGATACGCCCCGGGGGCGGGCCTCTGGCGCGCCACTGGATC
AGGCCCGTGGCGTGCCCGCCTCGTCAGCGCCACCCATTGCTAAGCGCTGACAGTAATA
GACCCCTCCATAGTAGTTGCCGATGTTAATTTGGTCACCGGCCGAAACGTATGAACGC
AGCACAGGGCAGGTACTACGGAGCGAAAGGTAGCTGATTGGCAGGGGCTGCTGGCGC
GCCTACCAACCTGTTCCTCCGCGCCTGCTGGAGCGACCAACTACGCCCCGCAGCGACC
GGTACCCGAGCAGTCTCTCAACTGGTTCGATGAGACTGTATACACCGTCGTTGGGACG
CGGACTAAACCGCCCCCTCATACCCACCCGCCCGTCCGGAGCGGAACGACTCGGCGGT
ACCGGCGTCAGGCCCCCTCGCCTAGGCTGCACCATTATGTTGGGAGGTGCGTCGACTG
GGGGCCGGCGACTTGAGCTCGGTCGTCCCGTGAAGCCCATCAGTCCCATGGAGACGTT
CTCTCCCATCCATTGGCCTCCGGGGCTCTCCACCAATCGCACCGGAGTCTTGTCTAGTG
TTAAATGTATTCATCGTGGGGTATAAACCGCCCCCGCGTAAGTGCGGCTCGAAGTCTG
GCCTCCCAGGCTGCTAGCAAACACGGAAGTGTGCCTCGTCTCGCTCAGAGGGGACAAG
ACACTCTCGACGGCTCTCAACAAGCCTAGTCCCCACGATACATACAACCGTGCCCGGT
GGGATTAATACGCCGGGGTCCCGTCGCGGGACATCTGGGATGGGAACACGTTCCGACC

ATATGCTCTCTGTCTGCATCTCTTGGGGAAATCCATTACGCTGCCGTTGACGTCGTTAA
CGTCACGCCACTAATACCGAACTAGGGGGCCAAGCTATACCATGTAATCCGTGCGCC
TACCCACTGTTGCGATTCAGGGTACGGGGCTGTGAGTAATGTACACACTCCTGGTGTGA
GCTTGTCCTCCGGACTTGAATGGCTACTCGCCGGGCTGGGACTTAGCAAACCACCCTGA
CGCATTCCAGGCCTCTGATCAGATCACAATTTTGCTTTCACCAGTTCCCAATTACGGTT
CCGCATCGTCGGGAGGCCTTCGACTAGATCTACTCGCATGGTTCGAGCGGTAATATCG
GGTCTGAAATCCCCTGACCCAACGGGAGGCGGTACGTGTGATCAGGATCTGCACCTTG
CGAGAGCCAAGCGTAATGACGGTGGCTCTTCCACAAGTAAAAGCTCGGGCGCGAGCG
GCGGGCTGGATGGTCCCCCGAATGTTCCCCCCTATGCCGAAGGCTGAACTCGAGGCTG
AGCCGTGGGCGCGCACCGTTGCCACGCAGGTGCACCGCGAGGTCCCTTGAAGGTCCCG
TCACGAAG

>LW_ERR173191.2

GTCGGGATGCCTTCCCAACTCTGGCACGGCGCGGCGCCTTCGCGCACGGATCGGATAA
GCTTTCCCGTGGACGGCTCGTCCTCGGAAATATCTCGTAGTCTTCTCGTTGGTTGCTCCA
ACGAAGCGACGAATCTACTTGGATACTGAACCGCGAGAGCTATTCAAGTGCTCTACTA
CTCGACCGGGGGCAGCATACAGCCGTGGGATCGGCGCGGCCCACCTCCGGCCAACGAG
AGTTACGAGTACCCGGCCCGTTTTCCCGCGTCGGAGGCTGCCAACACGATAGTAACTG
AACTGGCCCAAACGTATTGATACGCCCCGGGGGCGGGCCTCTGGCGCGCCACTGGATC
AGGCCCGTGGCGTGCCCGCCTCGTCAGCGCCACCCATTGCTAAGCGCTGACAGTAATA
GACCCCTCCATAGTAGTTGCCGATGTTAATTCGGTCACCGGCCGAAACGTATGAATGC
AGCACAGGGCAGGTACTACGGAGCGAAAGGTAGCTGATTGGCAGGGGCTGCTGGCGC
GCCTACCAACCTGTTCCTCCGCGCCTGCTGGAGCGACCAACTACGCCCCGCAGCGACC
GGTACCCGAGCAGTCTCTCAACTGGTTCGATGAGACTGTATACACCGTCGTTGGGACG
CGGACTAAACAACCTCCTCATACCAATCCGCCCGTCCGGAGCGGAACGACTCGGCGGT
ACCGGCGTCAGGCCCCCTCGCCTAGGCTGCACCATTAGGTTGGGAGGTGCGTCGACTG
GGGGCCGGCGACTTGAGCTCGGTCGTCCCGTGAAGCTCATCAGTCCCATGGAGACGTT
CTCTCCCATCCATTGGCCTCCGGGGCTCTCCACCAATCGCACCGGAGTCTTGTCTAGTG
TTAAATGTATTCATCGTGGGGTATAAATCGCCCCCGCGTGAGTGCGGCTCGAAGTCTGG
CCTCCCAGGCTGCTAGCTAACACGGAAGTGTGCCTCGTTTCGCTCAGAGGGGACGAGA
CACTCTCGACGGCTCTCAACAAGCCTAGTCCCCACGATACATACAACCGTGCCCGGTG
GGATTAATACGCCGGGGTCCCGTCGCGGGACATCTGGGATGGGAACACGTTCCGGCCA
TATGCTCTCTGTCCGCATCTCTTGGGGAAATCCATTACGCTGCCGTTGACGTCGTTAAC
GTCACGCCACTAATACCGAACTAGGGGGACCAAACTATACCATGTTATCCGTGCGCCT
ACCCACTGTTGCGATTCAGGGTACGGGGCTGTGAGTAATGTACACACTCCTGGTGGGA
GCTTGTCCTCCGGACTTGAATGGCTACTCGCCGGGCTGGGACTTAGCAAACCACCCTGA
CGCATTCCAGGCCTCTGATCAGATCACAATTTTGCTTTCACCAGTTCCCAATTACGGTT
CCGCATCGTCGGGAGGCCTTCGACTAGATCTACTCGCATGGTTCGAGCGGTAATATCG
GGTCTGAAATCCCCTGACCCAACGGGAGGCGGTACGTGTGATCGGAATCTGCACCTTG
CGAGAGCTAATCGTAATGACGATTGCTCTTCCACAAGTGAGTGCTGGGGCGCGAGTGG
CGGACCAGATTGTCTTCCGAATGTTCCCCCCCGTGTCGAAGGCTGAACTCGAGGCGGA
GCCGTGAGCGCGCACCGTTGCCACGCAGGTGCACCGCGAGGCACCTCGAAGGTCCCGT
CATGAAG

>LW_ERR173192.1

GTCGGGATGCCTTCCCAACTCTGGCACGGCGCGGCGCCTTCGCGCACGGATCGGATAA
GCTTTCCCGTGGACGGCTCGTCCTCGGAAATATCTCGTAGTCTTCTCGTTGGCTGCTCC
AACGAAGCGACGAATCTACTTGGATACTGAACCGCGAGAGCTATTCAAGTGCTCTACT
ACTCGACCGGGGGCAGCATACAGCCGTGGGATCGGCGCGGCCCACCTCCGGCCAACGA
GAGTTACGAGTACCCGGCCCGTTTTCCCGCGTCGGAGGCTGCCAACACGATAGTAACT

GAACTGGCCCAAACGTATTGATACGCCCCGGGGGCGGGCCTCTGGCGCGCCACTGGAT
CAGGCCCGTGGCGTGCCCGCCTCGTCAGCGCCACCCATTGCTAAGCGCTGACAGTAAT
AGACCCCTCCATAGTAGTTGCCGATGTTAATTCGGTCACCGGCCGAAACGTATGAACG
CAGCACAGGGCAGGTACTACGGAGCGAAAGGTAGCTGATTGGCAGGGGCTGCTGGCG
CGCCTACCAACCTGTTCCTCCGCGCCTGCTGGAGCGACCAACTACGCCCCGCAGCGAC
CGGTACCCGAGCAGTCTCTCAACTGGTTCGATGAGACTGTATACACCGTCGTTGGGAC
GCGGACTAAACAACCTCCTCATACCAATCCGCCCGTCCGGAGCGGAACGACTCGGCGG
TACCGGCGTCAGGCCCCCTCGCCTAGGCTGCACCATTATGTTGGGAGGTGCGTCGACTG
GGGGCCGGCGACTTGAGCTCGGTCGTCCCGTGAAGCCCATCAGTCCCATGGAGACGTT
CTCTCCCATCCATTGGCCTCCGGGGCTCTCCACCAATCGCACCGGAGTCTTGTCTAGTG
TTAAATGTATTCATCGTGGGGTATAAATCGCCCCCGCGTGAGTGCGGCTCGAAGTCTGG
CCTCCCAGGCTGCTAGCTAACACGGAAGTGTGCCTCGTTTCGCTCAGAGGGGACGAGA
CACTCTCGACGGCTCTCAACAAGCCTAGTCCCCACGATACATACAACCGTGCCCGGTG
GGATTAATACGCCGGGGTCCCGTCGCGGGACATCTGGGATGGGAACACGTTCCGGCCA
TATGCTCTCTGTCCGCATCTCTTGGGGAAATCCATTACGCTGCCGTTGACGTCGTTAAC
GTCACGCCACTAATACCGAACTAGGGGGACCAAACTATACCATGTTATCCGTGCGCCT
ACCCACTGTTGCGATTCAGGGTACGGGGCTGTGAGTAATGTACACACTCCTGGTGGGA
GCTTGTCCTCCGGACTTGAATGGCTACTCGCCGGGCTGGGACTTAGCAAACCACCCTGA
CGCATTCCAGGCCTCTGATCAGATCACAATTTTGCTTTCACCAGTTCCCAATTACGGTT
CCGCATCGTCGGGAGGCCTTCGACTAGACCTACTCGCATGGTTCGAGCGGTAATATCG
GGTCTGAAATCCCCTGACCCAACGGGAGGCGGTACGTGTGATCGGAATCTGCACCTTG
CGAGAGCTAATCGTAATGACGGTTGCTCTTCCACAAGTGAGTGCTGGGGCGCGAGTGG
CGGACCAGATTGTCTTCCGAATGTTCCCCCCCGTGTCGAAGGCTGAACTCGAGGCGGA
GCCGTGAGCGCGCACCGTTGCCACGCAGGTGCACCGCGAGGCACCTCGAAGGTCCCGT
CATGAAG

>LW_ERR173192.2

GTCGGGATGCCTTCCCAACTCTGGCACGGCGCGGCGCCTTCGCGCACGGATCGGATAA
GCTTTCCCGTGGACGGCTCGTCCTCGGAAATATCTCGTAGTCTTCTCGTTGGTTGCTCCA
ACGAAGCGACGAATCTACTTGGATACTGAACCGCGAGAGCTATTCAAGTGCTCTACTA
CTCGACCGGGGGCAGCATACAGCCGTGGGATCGGCGCGGCCCACCTCCGGCCAACGAG
AGTTACGAGTACCCGGCCCGTTTTCCCGCGTCGGAGGCTGCCAACACGATAGTAACTG
AACTGGCCCAAACGTATTGATACGCCCCGGGGGCGGGCCTCTGGCGCGCCACTGGATC
AGGCCCGTGGCGTGCCCGCCTCGTCAGCGCCACCCATTGCTAAGCGCTGACAGTAATA
GACCCCTCCATAGTAGTTGCCGATGTTAATTCGGTCACCGGCCGAAACGTATGAACGC
AGCACAGGGCAGGTACTACGGAGCGAAAGGTAGCTGATTGGCAGGGGCTGCTGGCGC
GCCTACCAACCTGTTCCTCCGCGCCTGCTGGAGCGACCAACTACGCCCCGCAGCGACC
GGTACCCGAGCAGTCTCTCAACTGGTTCGATGAGACTGTATACACCGTCGTTGGGACG
CGGACTAAACAACCTCCTCATACCAGTCCGCCCGTCCGGAGCGGAACGACTCGGCGGT
ACCGGCGTCAGGCCCCCTCGCCTAGGCTACACCATTATGTTGGGAGGTGCGTCGACTG
GGGGCCGGCGACTTGAGCTCGGTCGTCCCGTGAAGCCCATCAGTCCCATGGAGACGTT
CTCTCCCATCCATTGGCCTCCGGGGCTCTCCACCAATCGCACCGGAGTCTTGTCTAGTG
TTAAATGTATTCATCGTGGGGTATAAACCGCCCCCGCGTGGGTGCGGCTCGAAGTCTG
GCCTCCCAGGCTGCTAGCTAACACGGAAGTGTGCCTCGTTTCGCTCAGAGGGGACGAG
ACACTCTCGACGGCTCTCAACAAGCCTAGTCCCCACGATACATACAACCGTGCCCGGT
GGGATTAATACACCGGGGTCCCGTCGCGGGACGTCTGGGATGGGACCATGTTCCGGCC
GTATACTCCCTGTTCGTGCCCCTTGGAGAAATCCATTACGCTGCCGTTGACGTCGTTAA
CGTCACGCCACTAATACCGAACTAGGGGGACCAAACTATACCATGTAATCCGTGCGCC
TACCCACTGTTGCGATTCAGGGTACGGGGCTGTGAGTAATGTACACACTCCTGGTGTGA
GCTTGTCCTCCGGACTTGAATGGCTACTCGCCGGGCTGGGACTTAGCAAACCACCCTGA
CGCATTCCAGGCCTCTGATCAGATCACAATTTTGCTTTCACCAGTTCCCAATTACGGTT

CCGCATCGTCGGGAGGCCTTCGACTAGATCTACTCGCATGGTTCGAGCGGTAATATCG
GGTCTGAAATCCCCTGACCCAACGGGAGGCGGTACGTGTGATCAGGATCTGCACCTTG
CGAGAGCTAAGCGTAATGACGGTGGCTCTTCCACAAGTAAAAGCTCGGGCGCGAGCGG
CGGGCTGGATGGTCCCCCGAATGTTCCCCCCTATGCCGAAGGCTGAACTCGAGGCTGA
GCCGTGGGCGCGCACCGTTGCCACGCAGGTGCACCGCGAGGTCCCTTGAAGGTCCCGT
CACGAAG

>LW_ERR173193.1

GTCGGGATGCCTTCCCAACTCTGGCACGGCGCGGCGCCTTCGCGCACGGATCGGATAA
GCTTTCCCGTGGACGGCTCGTCCTTGGAAATATCTCGTAGTCTTCTCGTTGGTTGCTCCA
ACGAAGCGACGAATCTACTTGGATACTGAACCGCGAGAGCTATTCAAGTGCTCTACTA
CTCGACCGGGGGCAGCATACAGCCGTGGGATCGGCGCGGCCCACCTCCGGCCAACGAG
AGTTACGAGTACCCGACGCGTTTTCCCGCGTCGGAGGCTGCCAACACGATAGTAACTG
AACTGGCCCAAACGTATTGATACGCCCCGGGGGCGGGCCTCTGGCGCGCCACTGGATC
AGGCCCGTGGCGTGCCCGCCTCGTCAGCGCCACCCATTGCTAAGCGCTGACAGTAATA
GACCCCTCCATAGTAGTTGCCGATGTAGGTCTGGTCACCGGCCGAAACGTATGAACGC
AGCACAGGGCAGGTACTACGGAGCGAAAGGTAGCTGATTGGCAGGGGCTGCTGGCGC
GCCTACCAACCTGTTCCTCCGCGCCTGCTGGAGCGACCAACTACGCCCCGCAGCGACC
GGTACCCGAGCAGTCTCTCAACTGGTTCGATGAGACTGTATACACCGTCGTTGGGACG
CGGACTAAACCGCCCCCTCATACCCACCCGCCCGTCCGGAGCGGAACGACTCGGCGGT
ACCGGCGTCAGGCCCCCTCGCCTAGGCTGCACCATTATGTTGGGAGGTGCGTCGACTG
GGGGCCGGCGACTTGAGCTCGGTCGTCCCGTGAAGCCCATCAGTCCCATGGAGACGTT
CTCTCCCATCCATTGGCCTCCGGGGCTCTCCACCAATCGCACCGGAGTCTTGTCTAGTG
TTAAATGTATTCATCGTGGGGTATAAACCGCCCCCGCGTGAGTGCGGCTCGAAGTCTG
GCCTCCCAGGCTGCTAGCTAACACGGAAGTGTGCCTCGTTTCGCTCAGAGGGGACGAG
ACACTCTCGACGGCTCTCAACAAGCCTAGTCCCCACGATACATACAACCGTGCCCGGT
GGGATTAATACGCCGGGGTCCCGTCGCGGGACATCTGGGATGGGAACACGTTCCGGCC
ATATGCTCTCTGTCCGCATCTCTTGGGGAAATCCATTACGCTGCCGTTGACGTCGTTAA
CGTCACGCCACTAATACCGAACTAGGGGGACCAAGCTATACCATGTAATCCGTGCGCC
TACCCACTGTTGCGATTCAGGGTACGGGGCTGTGAGTAATGTACACACTCCTGGTGTGA
GCTTGTCCTCCGGACTTGAATGGCTACTCGCCGGGCTGGGACTTAGCAAACCACCCTGA
CGCATTCCAGGCCTCTGATCAGATCACAATTTTGCTTTCACCAGTTCCCAATTACGGTT
CCGCATCGTCGGGAGGCCTTCGACTAGATCTACTCGCATGGTTCGAGCGGTAATATCG
GGTCTGAAATCCCCTGACCCAACGGGAGGCGGTACGTGTGATCAGGATCTGCACCTTG
CGAGAGCCAAGCGTAATGACGGTGGCTCTTCCACAAGTAAAAGCTCGGGCGCGAGCG
GCGGGCTGGATGGTCCCCCGAATGTTCCCCCCTATGCCGAAGGCTGAACTCGAGGCTG
AGCCGTGGGCGCGCACCGTTGCCACGCAGGTGCACCGCGAGGTCCCTTGAAGGTCCCG
TCACGAAG

>LW_ERR173193.2

GTCGGGATGCCTTCCCAACTCTGGCACGGCGCGGCGCCTTCGCGCACGGATCGGATAA
GCTTTCCCGTGGACGGCTCGTCCTCGGAAATATCTCGTAGTCTTCTCGTTGGTTGCTCCA
ACGAAGCGACGAATCTACTTGGATACTGAACCGCGAGAGCTATTCAAGTGCTCTACTA
CTCGACCGGGGGAAGCATACAGCCGTGGGATCGGCGCGGCCCACCTCCGGCCAACGA
GAGTTACGAGTACCCGGCCCGTTTTTCCGCGTTGGAGGCTGCCAACCCGATAACAACT
GAACTGGCCCAAACGTATTGATACGCCCCGGGGGCGGGCCTCTGGCGCGCCACTGGAT
CAGGCCCGTGGCGTGCCCGCCTCGTCAGCGCCACCCATTGCTAAGCGCTGACAGTAAT
AGACCCCTCCATAGTAGTTGCCGATGTTAATTCGGTCACCGGCCGAAACGTATGCACTT
AGCACAGGGCAGGTACTACAAAGCGAGAGGTGGACGATTGGCAAGGGCTGCTGGCGA
GCCTACCAACCTGTTCCTCCGCGCCTGCTGGAGCGACCAACTACGCCCCGCAGCGACC

GGTACTTGAGCTGTCTCTCAACTGGTTCGATGAGACTGTATACACCGTCGTTGGGACGC
GGACTAAACCGCCCCCTCATACCCACCCGCCCGTCCGGAGCGGAACGACTCGGCGGTA
CCGGCGTCAGGCCCCCTCGCCTAGGCTGCACCATTATGTTGGGAGGTGCGTCGACTGG
GGGCCGGCGACTTGAGCTCGGTCGTCCCGTGAAGCCCATCAGTCCCATGGAGACGTTC
TCTCCCATCCATTAGCCTCCGGGGCTCTCCACCAATCGCACCGGAGTCTTGTCTAGTGT
TAAATGTATTCATCGTGGGGTATAAACCGCCCCCGCGTGGGTGCGGCTCGAAGTCTGG
CCTCCCAGGCTGCTAGCTAACACGGAAGTGTGCCTCGTTTCGCTCAGAGGGGACGAGA
CACTCTCGACGGCTCTCAACAAGCCTAGTCCCCACGATACATACAACCGTGCCCGGTG
GGATTAATACGCCGGGGTCCTGTCGCGGGACATCTGGGATGGGAACACGTTCCGGCCA
TATGTTCTCTGTCCGCATCTCTTGGGGAAATTCATTACGCTGCCGTTGACGTCGTTAAC
GTCACGCCACTAATACCGAACTAGGGGGACCAAACTATACCATGTAATCCGTGCGCCT
ACCCACTGTTGCGATTCAGGGTACGGGGCTGTGAGTAATGTACACACTCCTGGTGTGA
GCTTGTCCTCCGGACTTGAATGGCTACTCGCCGGGCTGGGACTTAGCAAACCACCCTGA
CGCATTCCAGGCCTCTGATCAGATCACAATTTTGCTTTCACCAGTTCCCAATTACGGTT
CCGCATCGTCGGGAGGCCTTCGACTAGATCTACTCGCATGGTTCGAGCGGTAATATCG
GGTCTGAAATCCCCTGACCCAACGGGAGGCGGTACGTGTGATCAGGATCTGCACCTTG
CGAGAGCCAAGCGTAAGGACGGTGGCTCTTCCACAAGTAAAAGCTCGGGCGCGAGCG
GCGGGCTGGATGGTCCCCCGAATGTTCCCCCCTATGCCGAAGGCTGAACTCGAGGCTG
AGCCGTGGGCGCGCACCGTTGCCACGCAGGTGCACCGCGAGGTCCCTTGAAGGTCCCG
TCACGAAG

>LW_ERR173194.1

GTCGGGATGCCTTCCCAACTCTGGCACGGCGCGGCGCCTTCGCGCACGGATCGGATAA
GCTTTCCCGTGGACGGCTCGTCCTCGGAAATATCTCGTAGTCTTCTCGTTGGTTGCTCCA
ACGAAGCGACGAATCTACTTGGATACTGAACCGCGAGAGCTATTCAAGTGCTCTACTA
CTCGACCGGGGGCAGCATACAGCCGTGGGATCGGCGCGGCCCACCTCCGGCCAACGAG
AGTTACGAGTACCCGGCCCGTTTTCCCGCGTCGGAGGCTGCCAACACGATAGTAACTG
AACTGGCCCAAACGTATTGATACGCCCCGGGGGCGGGCCTCTGGCGCGCCACTGGATC
AGGCCCGTGGCGTGCCCGCCTCGTCAGCGCCACCCATTGCTAAGCGCTGACAGTAATA
GACCCCTCCATAGTAGTTGCCGATGTTAATTCGGTCACCGGCCGAAACGTATGAACGC
AGCACAGGGCAGGTACTACGGAGCGAAAGGTAGCTGATTGGCAGGGGCTGCTGGCGC
GCCTACCAACCTGTTCCTCCGCGCCTGCTGGAGCGACCAACTACGCCCCGCAGCGACC
GGTACCCGAGCAGTCTCTCAACTGGTTCGATGAGACTGTATACACCGTCGTTGGGACG
CGGACTAAACAACCTCCTCATACCAGTCCGCCCGTCCGGAGCGGAACGACTCGGCGGT
ACCGGCGTCAGGCCCCCTCGCCTAGGCTACACCATTATGTTGGGAGGTGCGTCGACTG
GGGGCCGGCGACTTGAGCTCGGTCGTCCCGTGAAGCCCATCAGTCCCATGGAGACGTT
CTCTCCCATCCATTGGCCTCCGGGGCTCTCCACCAATCGCACCGGAGTCTTGTCTAGTG
TTAAATGTATTCATCGTGGGGTATAAACCGCCCCCGCGTGGGTGCGGCTCGAAGTCTG
GCCTCCCAGGCTGCTAGCTAACACGGAAGTGTGCCTCGTTTCGCTCAGAGGGGACGAG
ACACTCTCGACGGCTCTCAACAAGCCTAGTCCCCACGATACATACAACCGTGCCCGGT
GGGATTAATACACCGGGGTCCCGTCGCGGGACGTCTGGGATGGGACCATGTTCCGGCC
GTATACTCCCTGTTCGTGCCCCTCGGAGAAATCCATTACGCTGCCGTTGACGTCGTTAA
CGTCACGCCACTAATACCGAACTAGGGGGACCAAACTATACCATGTAATCCGTGCGCC
TACCCACTGTTGCGATTCAGGGTACGGGGCTGTGAGTAATGTACACACTCCTGGTGTGA
GCTTGTCCTCCGGACTTGAATGGCTACTCGCCGGGCTGGGACTTAGCAAACCACCCTGA
CGCATTCCAGGCCTCTGATCAGATCACAATTTTGCTTTCACCAGTTCCCAATTACGGTT
CCGCATCGTCGGGAGGCCTTCGACTAGATCTACTCGCATGGTTCGAGCGGTAATATCG
GGTCTGAAATCCCCTGACCCAACGGGAGGCGGTACGTGTGATCAGGATCTGCACCTTG
CGAGAGCCAAGCGTAATGACGGTGGCTCTTCCACAAGTAAAAGCTCGGGCGCGAGCG
GCGGGCTGGATGGTCCCCCGAATGTTCCCCCCTATGCCGAAGGCTGAACTCGAGGCTG

AGCCGTGGGCGCGCACCGTTGCCACGCAGGTGCACCGCGAGGTCCCTTGAAGGTCCCG
TCACGAAG

>LW_ERR173194.2

GTCGGGATGCCTTCCCAACTCTGGCACGGCGCGGCGCCTTCGCGCACGGATCGGATAA
GCTTTCCCGTGGACGGCTCGTCCTCGGAAATATCTCGTAGTCTTCTCGTTGGTTGCTCCA
ACGAAGCGACGAATCTACTTGGATACTGAACCGCGAGAGCTATTCAAGTGCTCTACTA
CTCGACCGGGGGCAGCATACAGCCGTGGGATCGGCGCGGCCCACCTCCGGCCAACGAG
AGTTACGAGTACCCGGCCCGTTTTCCCGCGTCGGAGGCTGCCAACACGATAGTAACTG
AACTGGCCCAAACGTATTGATACGCCCCGGGGGCGGGCCTCTGGCGCGCCACTGGATC
AGGCCCGTGGCGTGCCCGCCTCGTCAGCGCCACCCATTGCTAAGCGCTGACAGTAATA
GACCCCTCCATAGTAGTTGCCGATGTTAATTCGGTCACCGGCCGAAACGTATGAATGC
AGCACAGGGCAGGTACTACGGAGCGAAAGGTAGCTGATTGGCAGGGGCTGCTGGCGC
GCCTACCAACCTGTTCCTCCGCGCCTGCTGGAGCGACCAACTACGCCCCGCAGCGACC
GGTACCCGAGCAGTCTCTCAACTGGTTCGATGAGACTGTATACACCGTCGTTGGGACG
CGGACTAAACAACCTCCTCATACCAATCCGCCCGTCCGGAGCGGAACGACTCGGCGGT
ACCGGCGTCAGGCCCCCTCGCCTAGGCTGCACCATTAGGTTGGGAGGTGCGTCGACTG
GGGGCCGGCGACTTGAGCTCGGTCGTCCCGTGAAGCTCATCAGTCCCATGGAGACGTT
CTCTCCCATCCATTGGCCTCCGGGGCTCTCCACCAATCGCACCGGAGTCTTGTCTAGTG
TTAAATGTATTCATCGTGGGGTATAAATCGCCCCCGCGTGAGTGCGGCTCGAAGTCTGG
CCTCCCAGGCTGCTAGCTAACACGGAAGTGTGCCTCGTTTCGCTCAGAGGGGACGAGA
CACTCTCGACGGCTCTCAACAAGCCTAGTCCCCACGATACATACAACCGTGCCCGGTG
GGATTAATACGCCGGGGTCCCGTCGCGGGACATCTGGGATGGGAACACGTTCCGGCCA
TATGCTCTCTGTCCGCATCTCTTGGGGAAATCCATTACGCTGCCGTTGACGTCGTTAAC
GTCACGCCACTAATACCGAACTAGGGGGACCAAACTATACCATGTTATCCGTGCGCCT
ACCCACTGTTGCGATTCAGGGTACGGGGCTGTGAGTAATGTACACACTCCTGGTGGGA
GCTTGTCCTCCGGACTTGAATGGCTACTCGCCGGGCTGGGACTTAGCAAACCACCCTGA
CGCATTCCAGGCCTCTGATCAGATCACAATTTTGCTTTCACCAGTTCCCAATTACGGTT
CCGCATCGTCGGGAGGCCTTCGACTAGATCTACTCGCATGGTTCGAGCGGTAATATCG
GGTCTGAAATCCCCTGACCCAACGGGAGGCGGTACGTGTGATCGGAATCTGCACCTTG
CGAGAGCTAATCGTAATGACGATTGCTCTTCCACAAGTGAGTGCTGGGGCGCGAGTGG
CGGACCAGATTGTCTTCCGAATGTTCCCCCCCGTGTCGAAGGCTGAACTCGAGGCGGA
GCCGTGAGCGCGCACCGTTGCCACGCAGGTGCACCGCGAGGCACCTCGAAGGTCCCGT
CATGAAG

>LW_ERR173195.1

GTCGGGATGCCTTCCCAACTCTGGCACGGCGCGGCGCCTTCGCGCACGGATCGGATAA
GCTTTCCCGTGGACGGCTCGTCCTCGGAAATATCTCGTAGTCTTCTCGTTGGTTGCTCCA
ACGAAGCGACGAATCTACTTGGATACTGAACCGCGAGAGCTATTCAAGTGCTCTACTA
CTCGACCGGGGGCAGCATACAGCCGTGGGATCGGCGCGGCCCACCTCCGGCCAACGAG
AGTTACGAGTACCCGGCCCGTTTTCCCGCGTCGGAGGCTGCCAACACGATAGTAACTG
AACTGGCCCAAACGTATTGATACGCCCCGGGGGCGGGCCTCTGGCGCGCCACTGGATC
AGGCCCGTGGCGTGCCCGCCTCGTCAGCGCCACCCATTGCTAAGCGCTGACAGTAATA
GACCCCTCCATAGTAGTTGCCGATGTTAATTCGGTCACCGGCCGAAACGTATGAATGC
AGCACAGGGCAGGTACTACGGAGCGAAAGGTAGCTGATTGGCAGGGGCTGCTGGCGC
GCCTACCAACCTGTTCCTCCGCGCCTGCTGGAGCGACCAACTACGCCCCGCAGCGACC
GGTACCCGAGCAGTCTCTCAACTGGTTCGATGAGACTGTATACACCGTCGTTGGGACG
CGGACTAAACAACCTCCTCATACCAATCCGCCCGTCCGGAGCGGAACGACTCGGCGGT
ACCGGCGTCAGGCCCCCTCGCCTAGGCTGCACCATTAGGTTGGGAGGTGCGTCGACTG
GGGGCCGGCGACTTGAGCTCGGTCGTCCCGTGAAGCTCATCAGTCCCATGGAGACGTT

CTCTCCCATCCATTGGCCTCCGGGGCTCTCCACCAATCGCACCGGAGTCTTGTCTAGTG
TTAAATGTATTCATCGTGGGGTATAAATCGCCCCCGCGTGAGTGCGGCTCGAAGTCTGG
CCTCCCAGGCTGCTAGCTAACACGGAAGTGTGCCTCGTTTCGCTCAGAGGGGACGAGA
CACTCTCGACGGCTCTCAACAAGCCTAGTCCCCACGATACATACAACCGTGCCCGGTG
GGATTAATACGCCGGGGTCCCGTCGCGGGACATCTGGATGGGAACACGTTCCGGCCA
TATGCTCTCTGTCCGCATCTCTTGGGGAAATCCATTACGCTGCCGTTGACGTCGTTAAC
GTCACGCCACTAATACCGAACTAGGGGGACCAAACTATACCATGTTATCCGTGCGCCT
ACCCACTGTTGCGATTCAGGGTACGGGGCTGTGAGTAATGTACACACTCCTGGTGGGA
GCTTGTCCTCCGGACTTGAATGGCTACTCGCCGGGCTGGGACTTAGCAAACCACCCTGA
CGCATTCCAGGCCTCTGATCAGATCACAATTTTGCTTTCACCAGTTCCCAATTACGGTT
CCGCATCGTCGGGAGGCCTTCGACTAGATCTACTCGCATGGTTCGAGCGGTAATATCG
GGTCTGAAATCCCCTGACCCAACGGGAGGCGGTACGTGTGATCGGAATCTGCACCTTG
CGAGAGCTAATCGTAATGACGATTGCTCTTCCACAAGTGAGTGCTGGGGCGCGAGTGG
CGGACCAGATTGTCTTCCGAATGTTCCCCCCCGTGTCGAAGGCTGAACTCGAGGCGGA
GCCGTGAGCGCGCACCGTTGCCACGCAGGTGCACCGCGAGGCACCTCGAAGGTCCCGT
CATGAAG

>LW_ERR173195.2

GTCGGGATGCCTTCCCAACTCTGGCACGGCGCGGCGCCTTCGCGCACGGATCGGATAA
GCTTTCCCGTGGACGGCTCGTCCTCGGAAATATCTCGTAGTCTTCTCGTTGGTTGCTCCA
ACGAAGCGACGAATCTACTTGGATACTGAACCGCGAGAGCTATTCAAGTGCTCTACTA
CTCGACCGGGGGCAGCATACAGCCGTGGGATCGGCGCGGCCCACCTCCGGCCAACGAG
AGTTACGAGTACCCGGCCCGTTTTCCCGCGTCGGAGGCTGCCAACACGATAGTAACTG
AACTGGCCCAAACGTATTGATACGCCCCGGGGGCGGGCCTCTGGCGCGCCACTGGATC
AGGCCCGTGGCGTGCCCGCCTCGTCAGCGCCACCCATTGCTAAGCGCTGACAGTAATA
GACCCCTCCATAGTAGTTGCCGATGTTAATTCGGTCACCGGCCGAAACGTATGAACGC
AGCACAGGGCAGGTACTACGGAGCGAAAGGTAGCTGATTGGCAGGGGCTGCTGGCGC
GCCTACCAACCTGTTCCTCCGCGCCTGCTGGAGCGACCAACTACGCCCCGCAGCGACC
GGTACCCGAGCAGTCTCTCAACTGGTTCGATGAGACTGTATACACCGTCGTTGGGACG
CGGACTAAACAACCTCCTCATACCAGTCCGCCCGTCCGGAGCGGAACGACTCGGCGGT
ACCGGCGTCAGGCCCCCTCGCCTAGGCTACACCATTATGTTGGGAGGTGCGTCGACTG
GGGGCCGGCGACTTGAGCTCGGTCGTCCCGTGAAGCCCATCAGTCCCATGGAGACGTT
CTCTCCCATCCATTGGCCTCCGGGGCTCTCCACCAATCGCACCGGAGTCTTGTCTAGTG
TTAAATGTATTCATCGTGGGGTATAAACCGCCCCCGCGTGGGTGCGGCTCGAAGTCTG
GCCTCCCAGGCTGCTAGCTAACACGGAAGTGTGCCTCGTTTCGCTCAGAGGGGACGAG
ACACTCTCGACGGCTCTCAACAAGCCTAGTCCCCACGATACATACAACCGTGCCCGGT
GGGATTAATACACCGGGGTCCCGTCGCGGGACGTCTGGATGGGACCATGTTCCGGCC
GTATACTCCCTGTTCGTGCCCCTCGGAGAAATCCATTACGCTGCCGTTGACGTCGTTAA
CGTCACGCCACTAATACCGAACTAGGGGGACCAAACTATACCATGTAATCCGTGCGCC
TACCCACTGTTGCGATTCAGGGTACGGGGCTGTGAGTAATGTACACACTCCTGGTGTGA
GCTTGTCCTCCGGACTTGAATGGCTACTCGCCGGGCTGGGACTTAGCAAACCACCCTGA
CGCATTCCAGGCCTCTGATCAGATCACAATTTTGCTTTCACCAGTTCCCAATTACGGTT
CCGCATCGTCGGGAGGCCTTCGACTAGATCTACTCGCATGGTTCGAGCGGTAATATCG
GGTCTGAAATCCCCTGACCCAACGGGAGGCGGTACGTGTGATCAGGATCTGCACCTTG
CGAGAGCCAAGCGTAATGACGGTGGCTCTTCCACAAGTAAAAGCTCGGGCGCGAGCG
GCGGGCTGGATGGTCCCCCGAATGTTCCCCCCCGTGCCGAAGGCTGAACTCGAGGCTG
AGCCGTGGGCGCGCACCGTTGCCACGCAGGTGCACCGCGAGGTCCCTTGAAGGTCCCG
TCACGAAG

>LW_ERR173196.1

GTCGGGATGCCTTCCCAACTCTGGCACGGCGCGGCGCCTTCGCGCACGGATCGGATAA
GCTTTCCCGTGGACGGCTCGTCCTCGGAAATATCTCGTAGTCTTCTCGTTGGTTGCTCCA
ACGAAGCGACGAATCTACTTGGATACTGAACCGCGAGAGCTATTCAAGTGCTCTACTA
CTCGACCGGGGGCAGCATACAGCCGTGGGATCGGCGCGGCCCACCTCCGGCCAACGAG
AGTTACGAGTACCCGGCCCGTTTTCCCGCGTCGGAGGCTGCCAACACGATAGTAACTG
AACTGGCCCAAACGTATTGATACGCCCCGGGGGCGGGCCTCTGGCGCGCCACTGGATC
AGGCCCGTGGCGTGCCCGCCTCGTCAGCGCCACCCATTGCTAAGCGCTGACAGTAATA
GACCCCTCCATAGTAGTTGCCGATGTTAATTCGGTCACCGGCCGAAACGTATGAATGC
AGCACAGGGCAGGTACTACGGAGCGAAAGGTAGCTGATTGGCAGGGGCTGCTGGCGC
GCCTACCAACCTGTTCCTCCGCGCCTGCTGGAGCGACCAACTACGCCCCGCAGCGACC
GGTACCCGAGCAGTCTCTCAACTGGTTCGATGAGACTGTATACACCGTCGTTGGGACG
CGGACTAAACAACCTCCTCATACCAATCCGCCCGTCCGGAGCGGAACGACTCGGCGGT
ACCGGCGTCAGGCCCCCTCGCCTAGGCTGCACCATTAGGTTGGGAGGTGCGTCGACTG
GGGGCCGGCGACTTGAGCTCGGTCGTCCCGTGAAGCTCATCAGTCCCATGGAGACGTT
CTCTCCCATCCATTGGCCTCCGGGGCTCTCCACCAATCGCACCGGAGTCTTGTCTAGTG
TTAAATGTATTCATCGTGGGGTATAAATCGCCCCCGCGTGAGTGCGGCTCGAAGTCTGG
CCTCCCAGGCTGCTAGCTAACACGGAAGTGTGCCTCGTTTCGCTCAGAGGGGACGAGA
CACTCTCGACGGCTCTCAACAAGCCTAGTCCCCACGATACATACAACCGTGCCCGGTG
GGATTAATACGCCGGGGTCCCGTCGCGGACATCTGGGATGGGAACACGTTCCGGCCA
TATGCTCTCTGTCCGCATCTCTTGGGGAAATCCATTACGCTGCCGTTGACGTCGTTAAC
GTCACGCCACTAATACCGAACTAGGGGGACCAAACTATACCATGTTATCCGTGCGCCT
ACCCACTGTTGCGATTCAGGGTACGGGGCTGTGAGTAATGTACACACTCCTGGTGGGA
GCTTGTCCTCCGGACTTGAATGGCTACTCGCCGGGCTGGGACTTAGCAAACCACCCTGA
CGCATTCCAGGCCTCTGATCAGATCACAATTTTGCTTTCACCAGTTCCCAATTACGGTT
CCGCATCGTCGGGAGGCCTTCGACTAGATCTACTCGCATGGTTCGAGCGGTAATATCG
GGTCTGAAATCCCCTGACCCAACGGGAGGCGGTACGTGTGATCGGAATCTGCACCTTG
CGAGAGCTAATCGTAATGACGATTGCTCTTCCACAAGTGAGTGCTGGGGCGCGAGTGG
CGGACCAGATTGTCTTCCGAATGTTCCCCCCCGTGTCGAAGGCTGAACTCGAGGCGGA
GCCGTGAGCGCGCACCGTTGCCACGCAGGTGCACCGCGAGGCACCTCGAAGGTCCCGT
CATGAAG

>LW_ERR173196.2

GTCGGGATGCCTTCCCAACTCTGGCACGGCGCGGCGCCTTCGCGCACGGATCGGATAA
GCTTTCCCGTGGACGGCTCGTCCTCGGAAATATCTCGTAGTCTTCTCGTTGGTTGCTCCA
ACGAAGCGACGAATCTACTTGGATACTGAACCGCGAGAGCTATTCAAGTGCTCTACTA
CTCGACCGGGGGCAGCATACAGCCGTGGGATCGGCGCGGCCCACCTCCGGCCAACGAG
AGTTACGAGTACCCGGCCCGTTTTCCCGCGTCGGAGGCTGCCAACACGATAGTAACTG
AACTGGCCCAAACGTATTGATACGCCCCGGGGGCGGGCCTCTGGCGCGCCACTGGATC
AGGCCCGTGGCGTGCCCGCCTCGTCAGCGCCACCCATTGCTAAGCGCTGACAGTAATA
GACCCCTCCATAGTAGTTGCCGATGTTAATTCGGTCACCGGCCGAAACGTATGAACGC
AGCACAGGGCAGGTACTACGGAGCGAAAGGTAGCTGATTGGCAGGGGCTGCTGGCGC
GCCTACCAACCTGTTCCTCCGCGCCTGCTGGAGCGACCAACTACGCCCCGCAGCGACC
GGTACTTGGGCAGTCTCTCAACTGGTTCGATGAGACTGTATACACCGTCGTTGGGACGC
GGACTAAACAACCTCCTCATACCAATCCGCCCGTCCGGAGCGGAACGACTCGGCGGTA
CCGGCGTCAGGCCCCCTCGCCTAGGCTGCACCATTATGTTGGGAGGTGCGTCGACTGG
GGGCCGGCGACTTGAGCTCGGTCGTCCCGTGAAGCCCATCAGTCCCATGGAGACGTTC
TCTCCCATCCATTAGCCTCCGGGGCTCTCCACCAATCGCACCGGAGTCTTGTCTAGTGT
TAAATGTATTCATCGTGGGGTATAAACCGCCCCCGCGTGGGTGCGGCTCGAAGTCTGG
CCTCCCAGGCTGCTAGCTAACACGGAAGTGTGCCTCGTTTCGCTCAGAGGGGACGAGA
CACTCTCGACGGCTCTCAACAAGCCTAGTCCCCACGATACATACAACCGTGCCCGGTG
GGATTAATACACCGGGGTCCCGTCGCGGACGTCTGGGATGGGACCATGTTCCGGCCG

TATACTCCCTGTTCGTGCCCCTCGGAGAAATCCATTACGCTGCCGTTGACGTCGTTAAC
GTCACGCCACTAACACCGAACTAGGGGGACCAAACTATACCATGTAATCCGTGCGCCT
ACCCACTGTTGCGATTCAGGGTACGGGGCTGTGAGTAATGTACACACTCCTGGTGTGA
GCTTGTCCTCCGGACTTGAATGGCTACTCGCCGGGCTGGGACTTAGCAAACCACCCTGA
CGCATTCCAGGCCTCTGATCAGATCACAATTTTGCTTTCACCAGTTCCCAATTACGGTT
CCGCATCGTCGGGAGGCCTTCGACTAGATCTACTCGCATGGTTCGAGCGGTAATATCG
GGTCTGAAATCCCCTGACCCAACGGGAGGCGGTACGTGTGATCAGGATCTGCACCTTG
CGAGAGCCAAGCGTAATGACGGTGGCTCTTCCACAAGTAAAAGCTCGGGCGCGAGCG
GCGGGCTGGATGGTCCCCCGAATGTTCCCCCCTATGCCGAAGGCTGAACTCGAGGCTG
AGCCGTGGGCGCGCACCGTTGCCACGCAGGTGCACCGCGAGGTCCCTTGAAGGTCCCG
TCACGAAG

>LW_ERR173197.1

GTCGGGATGCCTTCCCAACTCTGGCACGGCGCGGCGCCTTCGCGCACGGATCGGATAA
GCTTTCCCGTGGACGGCTCGTCCTCGGAAATATCTCGTAGTCTTCTCGTTGGTTGCTCCA
ACGAAGCGACGAATCTACTTGGATACTGAACCGCGAGAGCTATTCAAGTGCTCTACTA
CTCGACCGGGGGCAGCATACAGCCGTGGGATCGGCGCGGCCCACCTCCGGCCAACGAG
AGTTACGAGTACCCGGCCCGTTTTCCCGCGTCGGAGGCTGCCAACACGATAGTAACTG
AACTGGCCCAAACGTATTGATACGCCCCGGGGGCGGGCCTCTGGCGCGCCACTGGATC
AGGCCCGTGGCGTGCCCGCCTCGTCAGCGCCACCCATTGCTAAGCGCTGACAGTAATA
GACCCCTCCATAGTAGTTGCCGATGTTAATTCGGTCACCGGCCGAAACGTATGAACGC
AGCACAGGGCAGGTACTACGGAGCGAAAGGTAGCTGATTGGCAGGGGCTGCTGGCGC
GCCTACCAACCTGTTCCTCCGCGCCTGCTGGAGCGACCAACTACGCCCCGCAGCGACC
GGTACCCGAGCAGTCTCTCAACTGGTTCGATGAGACTGTATACACCGTCGTTGGGACG
CGGACTAAACAACCTCCTCATACCAGTCCGCCCGTCCGGAGCGGAACGACTCGGCGGT
ACCGGCGTCAGGCCCCCTCGCCTAGGCTACACCATTATGTTGGGAGGTGCGTCGACTG
GGGGCCGGCGACTTGAGCTCGGTCGTCCCGTGAAGCCCATCAGTCCCATGGAGACGTT
CTCTCCCATCCATTGGCCTCCGGGGCTCTCCACCAATCGCACCGGAGTCTTGTCTAGTG
TTAAATGTATTCATCGTGGGGTATAAACCGCCCCCGCGTGGGTGCGGCTCGAAGTCTG
GCCTCCCAGGCTGCTAGCTAACACGGAAGTGTGCCTCGTTTCGCTCAGAGGGGACGAG
ACACTCTCGACGGCTCTCAACAAGCCTAGTCCCCACGATACATACAACCGTGCCCGGT
GGGATTAATACACCGGGGTCCCGTCGCGGGACGTCTGGGATGGGACCATGTTCCGGCC
GTATACTCCCTGTTCGTGCCCCTCGGAGAAATCCATTACGCTGCCGTTGACGTCGTTAA
CGTCACGCCACTAATACCGAACTAGGGGGACCAAACTATACCATGTAATCCGTGCGCC
TACCCACTGTTGCGATTCAGGGTACGGGGCTGTGAGTAATGTACACACTCCTGGTGTGA
GCTTGTCCTCCGGACTTGAATGGCTACTCGCCGGGCTGGGACTTAGCAAATCACCCTGA
CGCATTCCAGGCCTCTGATCAGATCACAATTTTGCTTTCACCAGTTCCCAATTACGGTT
CCGCATCGTCGGGAGGCCTTCGACTAGATCTACTCGCATGGTTCGAGCGGTAATATCG
GGTCTGAAATCCCCTGACCCAACGGGAGGCGGTACGTGTGATCAGGATCTGCACCTTG
CGAGAGCCAAGCGTAATGACGGTGGCTCTTCCACAAGTAAAAGCTCGGGCGCGAGCG
GCGGGCTGGATGGTCCCCCGAATGTTCCCCCCTATGCCGAAGGCTGAACTCGAGGCTG
AGCCGTGGGCGCGCACCGTTGCCACGCAGGTGCACCGCGAGGTCCCTTGAAGGTCCCG
TCACGAAG

>LW_ERR173197.2

GTCGGGATGCCTTCCCAACTCTGGCACGGCGCGGCGCCTTCGCGCACGGATCGGATAA
GCTTTCCCGTGGACGGCTCGTCCTCGGAAATATCTCGTAGTCTTCTCGTTGGTTGCTCCA
ACGAAGCGACGAATCTACTTGGATACTGAACCGCGAGAGCTATTCAAGTGCTCTACTA
CTCGACCGGGGGCAGCATACAGCCGTGGGATCGGCGCGGCCCACCTCCGGCCAACGAG
AGTTACGAGTACCCGGCCCGTTTTCCCGCGTCGGAGGCTGCCAACACGATAGTAACTG

AACTGGCCCAAACGTATTGATACGCCCCGGGGGCGGGCCTCTGGCGCGCCACTGGATC
AGGCCCGTGGCGTGCCCGCCTCGTCAGCGCCACCCATTGCTAAGCGCTGACAGTAATA
GACCCCTCCATAGTAGTTGCCGATGTTAATTCGGTCACCGGCCGAAACGTATGAACGC
AGCACAGGGCAGGTACTACGGAGCGAAAGGTAGCTGATTGGCAGGGGCTGCTGGCGC
GCCTACCAACCTGTTCCTCCGCGCCTGCTGGAGCGACCAACTACGCCCCGCAGCGACC
GGTACCCGAGCAGTCTCTCAACTGGTTCGATGAGACTGTATACACCGTCGTTGGGACG
CGGACTAAACAACCTCCTCATACCAGTCCGCCCGTCCGGAGCGGAACGACTCGGCGGT
ACCGGCGTCAGGCCCCCTCGCCTAGGCTACACCATTATGTTGGGAGGTGCGTCGACTG
GGGGCCGGCGACTTGAGCTCGGTCGTCCCGTGAAGCCCATCAGTCCCATGGAGACGTT
CTCTCCCATCCATTGGCCTCCGGGGCTCTCCACCAATCGCACCGGAGTCTTGTCTAGTG
TTAAATGTATTCATCGTGGGGTATAAACCGCCCCCGCGTGGGTGCGGCTCGAAGTCTG
GCCTCCCAGGCTGCTAGCTAACACGGAAGTGTGCCTCGTTTCGCTCAGAGGGGACGAG
ACACTCTCGACGGCTCTCAACAAGCCTAGTCCCCACGATACATACAACCGTGCCCGGT
GGGATTAATACACCGGGGTCCCGTCGCGGGACGTCTGGATGGACCATGTTCCGGCC
GTATACTCCCTGTCCGTGCCCCTCGGAGAAATCCATTACGCTGCCGTTGACGTCGTTAA
CGTCACGCCACTAATACCGAACTAGGGGGACCAAACTATACCATGTAATCCGTGCGCC
TACCCACTGTTGCGATTCAGGGTACGGGGCTGTGAGTAATGTACACACTCCTGGTGTGA
GCTTGTCCTCCGGACTTGAATGGCTACTCGCCGGGCTGGGACTTAGCAAACCACCCTGA
CGCATTCCAGGCCTCTGATCAGATCACAATTTTGCTTTCACCAGTTCCCAATTACGGTT
CCGCATCGTCGGGAGGCCTTCGACTAGATCTACTCGCATGGTTCGAGCGGTAATATCG
GGTCTGAAATCCCCTGACCCAACGGGAGGCGGTACGTGTGATCAGGATCTGCACCTTG
CGAGAGCCAAGCGTAATGACGGTGGCTCTTCCACAAGTAAAAGCTCGGGCGCGAGCG
GCGGGCTGGATGGTCCCCCGAATGTTCCCCCCTATGCCGAAGGCTGAACTCGAGGCTG
AGCCGTGGGCGCGCACCGTTGCCACGCAGGTGCACCGCGAGGTCCCTTGAAGGTCCCG
TCACGAAG

>LW_ERR173198.1

GTCGGGATGCCTTCCCAACTCTGGCACGGCGCGGCGCCTTCGCGCACGGATCGGATAA
GCTTTCCCGTGGACGGCTCGTCCTCGGAAATATCTCGTAGTCTTCTCGTTGGTTGCTCCA
ACGAAGCGACGAATCTACTTGGATACTGAACCGCGAGAGCTATTCAAGTGCTCTACTA
CTCGACCGGGGGCAGCATACAGCCGTGGGATCGGCGCGGCCCACCTCCGGCCAACGAG
AGTTACGAGTACCCGGCCCGTTTTCCCGCGTCGGAGGCTGCCAACACGATAGTAACTG
AACTGGCCCAAACGTATTGATACGCCCCGGGGGCGGGCCTCTGGCGCGCCACTGGATC
AGGCCCGTGGCGTGCCCGCCTCGTCAGCGCCACCCATTGCTAAGCGCTGACAGTAATA
GACCCCTCCATAGTAGTTGCCGATGTTAATTCGGTCACCGGCCGAAACGTATGAATGC
AGCACAGGGCAGGTACTACGGAGCGAAAGGTAGCTGATTGGCAGGGGCTGCTGGCGC
GCCTACCAACCTGTTCCTCCGCGCCTGCTGGAGCGACCAACTACGCCCCGCAGCGACC
GGTACCCGAGCAGTCTCTCAACTGGTTCGATGAGACTGTATACACCGTCGTTGGGACG
CGGACTAAACAACCTCCTCATACCAATCCGCCCGTCCGGAGCGGAACGACTCGGCGGT
ACCGGCGTCAGGCCCCCTCGCCTAGGCTGCACCATTAGGTTGGGAGGTGCGTCGACTG
GGGGCCGGCGACTTGAGCTCGGTCGTCCCGTGAAGCTCATCAGTCCCATGGAGACGTT
CTCTCCCATCCATTGGCCTCCGGGGCTCTCCACCAATCGCACCGGAGTCTTGTCTAGTG
TTAAATGTATTCATCGTGGGGTATAAATCGCCCCCGCGTGAGTGCGGCTCGAAGTCTGG
CCTCCCAGGCTGCTAGCTAACACGGAAGTGTGCCTCGTTTCGCTCAGAGGGGACGAGA
CACTCTCGACGGCTCTCAACAAGCCTAGTCCCCACGATACATACAACCGTGCCCGGTG
GGATTAATACGCCGGGGTCCCGTCGCGGGACATCTGGATGGGAACACGTTCCGGCCA
TATGCTCTCTGTCCGCATCTCTTGGGGAAATCCATTACGCTGCCGTTGACGTCGTTAAC
GTCACGCCACTAATACCGAACTAGGGGGACCAAACTATACCATGTTATCCGTGCGCCT
ACCCACTGTTGCGATTCAGGGTACGGGGCTGTGAGTAATGTACACACTCCTGGTGGGA
GCTTGTCCTCCGGACTTGAATGGCTACTCGCCGGGCTGGGACTTAGCAAACCACCCTGA
CGCATTCCAGGCCTCTGATCAGATCACAATTTTGCTTTCACCAGTTCCCAATTACGGTT

CCGCATCGTCGGGAGGCCTTCGACTAGATCTACTCGCATGGTTCGAGCGGTAATATCG
GGTCTGAAATCCCCTGACCCAACGGGAGGCGGTACGTGTGATCGGAATCTGCACCTTG
CGAGAGCTAATCGTAATGACGATTGCTCTTCCACAAGTGAGTGCTGGGGCGCGAGTGG
CGGACCAGATTGTCTTCCGAATGTTCCCCCCCGTGTCGAAGGCTGAACTCGAGGCGGA
GCCGTGAGCGCGCACCGTTGCCACGCAGGTGCACCGCGAGGCACCTCGAAGGTCCCGT
CATGAAG

>LW_ERR173198.2

GTCGGGATGCCTTCCCAACTCTGGCACGGCGCGGCGCCTTCGCGCACGGATCGGATAA
GCTTTCCCGTGGACGGCTCGTCCTCGGAAATATCTCGTAGTCTTCTCGTTGGTTGCTCCA
ACGAAGCGACGAATCTACTTGGATACTGAACCGCGAGAGCTATTCAAGTGCTCTACTA
CTCGACCGGGGGCAGCATACAGCCGTGGGATCGGCGCGGCCCACCTCCGGCCAACGAG
AGTTACGAGTACCCGGCCCGTTTTCCCGCGTCGGAGGCTGCCAACACGATAGTAACTG
AACTGGCCCAAACGTATTGATACGCCCCGGGGGCGGGCCTCTGGCGCGCCACTGGATC
AGGCCCGTGGCGTGCCCGCCTCGTCAGCGCCACCCATTGCTAAGCGCTGACAGTAATA
GACCCCTCCATAGTAGTTGCCGATGTTAATTCGGTCACCGGCCGAAACGTATGAACGC
AGCACAGGGCAGGTACTACGGAGCGAAAGGTAGCTGATTGGCAGGGGCTGCTGGCGC
GCCTACCAACCTGTTCCTCCGCGCCTGCTGGAGCGACCAACTACGCCCCGCAGCGACC
GGTACTTGAGCTGTCTCTCAACTGGTTCGATGAGACTGTATACACCGTCGTTGGGACGC
GGACTAAACAACCTCCTCATACCAATCCGCCCGTCCGGAGCGGAACGACTCGGCGGTA
CCGGCGTCAGGCCCCCTCGCCTAGGCTGCACCATTATGTTGGGAGGTGCGTCGACTGG
GGGCCGGCGACTTGAGCTCGGTCGTCCCGTGAAGCCCATCAGTCCCATGGAGACGTTC
TCTCCCATCCATTAGCCTCCGGGGCTCTCCACCAATCGCACCGGAGTCTTGTCTAGTGT
TAAATGTATTCATCGTGGGGTATAAACCGCCCCCGCGTGGGTGCGGCTCGAAGTCTGG
CCTCCCAGGCTGCTAGCTAACACGGAAGTGTGCCTCGTTTCGCTCAGAGGGGACGAGA
CACTCTCGACGGCTCTCAACAAGCCTAGTCCCCACGATACATACAACCGTGCCCGGTG
GGATTAATACACCGGGGTCCCGTCGCGGGACGTCTGGGATGGGACCATGTTCCGGCCG
TATACTCCCTGTTCGTGCCCCTCGGAGAAATCCATTACGCTGCCGTTGACGTCGTTAAC
GTCACGCCACTAACACCGAACTAGGGGGACCAAACTATACCATGTAATCCGTGCGCCT
ACCCACTGTTGCGATTCAGGGTACGGGGCTGTGAGTAATGTACACACTCCTGGTGTGA
GCTTGTCCTCCGGACTTGAATGGCTACTCGCCGGGCTGGGACTTAGCAAACCACCCTGA
CGCATTCCAGGCCTCTGATCAGATCACAATTTTGCTTTCACCAGTTCCCAATTACGGTT
CCGCATCGTCGGGAGGCCTTCGACTAGATCTACTCGCATGGTTCGAGCGGTAATATCG
GGTCTGAAATCCCCTGACCCAACGGGAGGCGGTACGTGTGATCAGGATCTGCACCTTG
CGAGAGCCAAGCGTAATGACGGTGGCTCTTCCACAAGTAAAAGCTCGGGCGCGAGCG
GCGGGCTGGATGGTCCCCCGAATGTTCCCCCCTATGCCGAAGGCTGAACTCGAGGCTG
AGCCGTGGGCGCGCACCGTTGCCACGCAGGTGCACCGCGAGGTCCCTTGAAGGTCCCG
TCACGAAG

>MS_ERR173199.1

GTCGGGATGCCCTTTCGACACCAGCACGGCGCGACGCTTTCGCGCACGTATCGGATAA
ACTTTCCATTGGACGGCTCGTTCTCAGAAATATCTCGTAGTCTTCTCGTTGGTTACTCCA
ATGAAACGCCTCGTCTGCGGGGGCACTGAACCGCGAGAGCTATTCAAATGCTCTACTA
CTCGACCGGGGGCAGCTTACAACCGTGGGATCGGCGCGGCCCACCTCCGGTCAACGAG
AGTTACGAGTTCCCGGCCCGTTTTCTCGCGTCGGAGGCTGCCAACACGATAGTAACTGA
ACTGGCCCAAACGTATTAATACGCCCCGGGGGCGGGCCTCTGGCGCGCCACTGGATCA
GGCCCGTGGCGTGCCCGCCTCGTCAGCGCCACCCATTGCTAAGCGCTGACAGTAATAG
ACCCCTCCATAGTAGTTGCCGATGTTGATTCGGTCACCGGCCGAAACGTATGCACTTAG
CACAGGGCAGATACTACAAAGCGAGAGGGGGATGATTGGCAGGGGCTGCCGACGCGC
CTATCAGCCCGTTCCCCCGCGCCTGCTGTGGCGACCAACTACGCCCCGCAGCGTCCAGT

ACCCGAGCAGTCTCTCAACTGGTCCGATTAGACTGTATACACCGCCGTTGGGACGCGG
ACTAAACAACTCCCTCATACCCATCCGCCCGTCCGGAGCGGAACGACTCGGCGGTACC
GGCGTCAGGCCCCCTCGCCTAGACTGCACCATTATGTTGGGAGGTGCGTCGACTGGGG
GCCGGCGACTTGATCCTGGTCGGCCCGTGAAGCCCATCAGTCCCATGGAGACGTTCTCT
CCCATCTATTGACCTCCGGGGCTCTCCACCAATCGCACCGGAGTCTTGTCTAATATGAA
ATTTATTCATCGTGAGGTATAAACCGCCCCCGAGTGGGTGCGGCTTGAAGTCCAGCCCC
CCAGGCCGCCTGCTAACACGGAAGTGTACCCGGTTCCACTCAGAGGGCATGAGGCAGT
ACCTACGGATCTCAACAAGCCTAGTCCCCATGATACGTACAGCTGGGCCAGGTGGGAT
TAATACGCCGGGGTCCCGCCGCGAAACGGCTAGAAGGTAAATACGCCCTGGCCATATA
CTCTTTGTCCGCATCCCTTGGGGAAATCCATTATGCTGCCGTTGACGTCGTCAACGTCA
CGCCACTAACACCGAACTAGGGGGACCAAACTATACCATGTTATCCGTATACTTACCC
ACTGTTGCAGGTCAAGGTATGGGGCTGCAAGTAATGTACATACTCCTGGTGGGAACTT
GTCCTCCGGACTTGAATGGCTACCTGCCGGGCTGGGACTTAGCAAACCACCCTGACGC
ATCCCAGGCCTTTGATCGGATCACAATTTTGATTCCACCAGTTCCCAATTACGGTTCCG
CATCGTTGGGAGGCCTTTGGCTAGATCTACCTGCATGGTTCGAGCGGTAATATCGGGTC
TGAAATCCCCTGACCCAACGGGAGGCAGTATGTGTGGTCGGAATCTGCACCTTGCGAG
AGATAATCGTAATGACGATGGACCTTCTACAAGTGAGTGCTGGGGCACGAGCGGCGGA
CCAGATGGTCTTCTGAATGTTCCTCCCCGTGCCGAAGGCTGAACTCGAGGCGGAGCAG
TGGGCGCGTACCGTCGACATGCAGGTGCACCGCGAGGTCCCTTGAAGATCCCGTCATC
AAC

>MS_ERR173199.2

GTCGGGATGCCTTCCCAACTCCGGCACGGCGCGGCGCCTTCGCGCACGGATCGGATAA
GCTTACCCGTGGATGGCTCGTTCTCAGAAATATCTCGTAGTCTTCTCGTTGGTTACTCCA
ATGAAGCGCCTCGTCTGCGGGGGCACTGAACCGCGAGAGCTATTCAAGTGCTCTACTA
CTCGACCGGGGGCAGCTTACAACCGCGGGATTGGCACGGTCTACCTCCGTCCAACAGG
GGTTACGACGACCCGGTCCCCCCTCCCGCGTCGAAGGCTGCCAACACGATAGTAAGCG
AACTGGCCCAAACGTATTAATACGTACAGGGGGCGGGCCTCTGGCGCGCCACTGGATC
AGGCCCGTGGCGTGCCCGCCTCGTCAGCGCCACCCATTGCTAAGCGCTGACAGTAATA
GACCCCTCCATAGTAGTTGCCGATGTTGATTCGGTCACCGGCCGAAACGCGCGCGCTC
AGCACAAGGCAGGTACCACGGAGCGAAAGGTGGATGATTGGCAGGGGCTGCTGACGC
GCCTATCAGCCCGTTCCCCCGCGCCTGCTGTGGCGACCAACTACGCCCCGCAGCGTCCA
GTACCCGAGCAGTCTCTCAACTGGTCCGATTAGACTGTATACACCGCCGTTGGGACGC
GGACTAAACAACTCCCTCATACCCATCCGCCCGTCCGGAGCGGAACGACTCGGCGGTA
CCGGCGTCAGGCCCCCTCGCCTAGACTGCACCATTATGTTGGGAGGTGCGTCGACTGG
GGGCCGGCGACTTGATCCTGGTCGGCCCGTGAAGCCCATCAGTCCCATGGAGACGTTC
TCTCCCATCTATTGGCCTCCGGGGCTCTCCACCAATCGCACCGGAGTCTTGTCTAATAT
GCAATTTATTCATCGTGAGGTATAAACCGCCCCCGAGTGGGTGCGGCTTGAAGTCCAG
CCCCCCAGGCCGCCTGCTAACACGGAAGTGTACCCGGTTCCACTCAGAGGGCATGAGG
CAGTACCTACGGATCTCAACAAGCCTAGTCCCCATGATACGTACAGCTGGGCCAGGTG
GGATTAATACGCCGGGGTCCCGTTGCGGGATGTCTAAGGGGGAGATACGTTCCGGTCA
TATACTCTTTGTCCGCATCCCTTGGGGAAATCCATTATGCTCCCGTTGACGTCGTCAAC
GTCACGCCACTAACACCGAACTAGGGGGACCAAACTATACCATGTTATCCGTATACTT
ACCCACTGTTGCAGGTCAAGGTATGGGGCTGCAAGTAATGTACATACTCCTGGTGGGA
GCTTGTCCTCCGGACTCGAATGGCTACTCGCCGGGCTGGGACTTAGCAAACCACTCTGA
CGCATCTACGGCCTCAGATTAGATCACAATTTTGCTTTCTCCATTTCCTAATTACCGTTC
CGCATCGTCGGGAGGCCTTCGACTAGATTTACTCGCATGGTTTAAGCGATAATATCAAG
TCGGGAATCCTCTGACCCAACGTGAAGCGGTATGCGCAATTGGGGTCTGTTCCTTGCAA
GAGCTAATCGTAATGACGATGGACCTTCTACAAGTGAGTGCTGTAGCGCCAGCAGCGG
ACCAGATTGTTTTCCGGATGTTCCCCCTCGTGCCGAAGGCTGAACTCAGGGCGGGGCC

GTGGGCGCGCATCGTTGCCACGCAGGTACACCGTGAGGCACCTTGAAGGTCCCGTCAT
GAAG

>MS_ERR173200.1

GTCGGGATGCCCTTTCGACACCAGCACGGCGCGACGCTTTCGCGCACGTATCGGATAA
ACTTTCCATTGGACGGCTCGTTCTCAGAAATATCTCGTAGTCTTCTCGTTGGTTACTCCA
ATGAAACGCCTCGTCTGCGGGGGCACTGAACCGCGAGAGCTATTCAAATGCTCTACTA
CTCGACCGGGGGCAGCTTACAACCGTGGGATCGGCGCGGCCCACCTCCGGTCAACGAG
AGTTACGAGTTCCCGGCCCGTTTTCTCGCGTCGGAGGCTGCCAACACGATAGTAACTGA
ACTGGCCCAAACGTATTAATACGCCCCGGGGGCGGGCCTCTGGCGCGCCACTGGATCA
GGCCCGTGGCGTGCCCGCCTCGTCAGCGCCACCCATTGCTAAGCGCTGACAGTAATAG
ACCCCTCCATAGTAGTTGCCGATGTTGATTCGGTCACCGGCCGAAACGTATGCACTTAG
CACAGGGCAGGTACTACAAAGCGAGAGGGGGATGATTGGCAGGGGCTGCTGACGCGC
CTATCAGCCCGTTCCCCCGCGTCTGCTGTGGCGACCAACTACGCCCCGCAGCGTCCAGT
ACCCGAGCAGTCTCTCAACTGGTCCGATTAGACTGTATACACCGCCGTTGGGACGCGG
ACTAAACAACTCCCTCATACCCATCCGCCCGTCCGGAGCGGAACGACTCGGCGGTACC
GGCGTCAGGCCCCCTCGCCTAGACTGCACCATTATGTTGGGAGGTGCGTCGACTGGGG
GCCGGCGACTTGATCCTGGTCGGCCCGTGAAGCCCATCAGTCCCATGGAGACGTTCTCT
CCCATCTATTGACCTCCGGGGCTCTCCACCAATCGCACCGGAGTCTTGTCTAATATGAA
ATTTATTCATCGTGAGGTATAAACCGCCCCCGAGTGGGTGCGGCTTGAAGTCCAGCCTC
CCAGGCCGCCTGCTAACACGGAAGTGTACCCGGTTCCACTCAGAGGGCATGAGGCAGT
ACCTACGGATCTCAACAAGCCTAGTCCCCATGATACGTACAGCTGGGCCAGGTGGGAT
TAATACGCCGGGGTCCCGCCGCGAAACGGCTAGAAGGGAAATACGCCCTGGCCATATA
CTCTTTGTCCGCATCCCTTGGGGAAATCCATTATGCTGCCGTTGACGTCGTCAACGTCA
CGCCACTAACACCGAACTAGGGGGACCAAACTATACCATGTTATCCGTATACTTACCC
ACTGTTGCAGGTCAAGGTATGGGGCTGCAAGTAATGTACATACTCCTGGTGGGAACTT
GTCCTCCGGACTTGAATGGCTACCTGCCGGGCTGGGACTTAGCAAACCACCCTGACGC
ATCCCAGGCCTTTGATCGGATCACAATTTTGATTCCACCAGTTCCCAATTACGGTTCCG
CATCGTTGGGAGGCCTTTGGCTAGATCTACCTGCATGGTTCGAGCGGTAATATCGGGTC
TGAAATCCCCTGACCCAACGGGAGGCAGTATGTGTGGTCGGAATCTGCACCTTGCGAG
AGATAATCGTAATGACGATGGACCTTCTACAAGTGAGTGCTGGGGCACGAGCGGCGGA
CCAGATGGTCTTCTGAATGTTCCTTCCCGTGCCGAAGGCTGAACTCGAGGCGGAGCAG
TGGGCGCGCACCGTCGACATGCAGGTGCACCGCGAGGTCCCTTGAAGATCCCGTCATC
AAC

>MS_ERR173200.2

GTCGGGACGCCCTCCCGGCTCTGGCACGGCGCGGTGGCTTTGCGCACGGATCGGATAA
ACTTTCCATTGGACGGCTCGTTCTCAGAAATATCTCGTAGTCTTCTCGTTGGTTACTCCA
ATGAAACGCCTCGTCTGCGGGGGCACTGAACCGCGAGAGCTATTCAAATGCTCTACTA
CTCGACCGGGGGCAGCTTACAACCGTGGGATCGGCGCGGCCCACCTCCGGTCAACGAG
AGTTACGAGTTCCCGGCCCGTTTTCTCGCGTCGGAGGCTGCCAACACGATAGTAACTGA
ACTGGCCCAAACGTATTAATACGCCCCGGGGGCGGGCCTCTGGCGCGCCACTGGATCA
GGCCCGTGGCGTGCCCGCCTCGTCAGCGCCACCCATTGCTAAGCGCTGACAGTAATAG
ACCCCTCCATAGTAGTTGCCGATGTTGATTTGGTCACCGGCCGAAACGTATGCGCTCAG
CACAGGGCAGGTACTACGGAGCGAAAGGTGGATGATTGGCAGGGGCCGCTGGCGCAC
CTACAAAACTATTCGTCCGCGCCTGCTGGAGCGACCAACTACGCTCTATAGCGTCCAGT
ACCCGAGCAGTCTCTCAATTGGTCCGATGAGACTGTATACGCCGCCGTTGGGACGCGG
ACTAAACAACCCCCTCATACCCATTCGCCCGTCCAGAGCGGAACGACTCCGCGGTGCC
GGCGTCAGGCCTCCTCGCCTAGGCTGCGCCATTATGTTGGGAGGTGCGTTGACTGGGG
GCTGGCAATTTGATCCCGGTCGGCCCGTGAAGCCCATCAGTTCCATGGAGACATTCTTT

GCCGTCCATTGGCCTTCTGGGCTCTCCGCCAATTACGTCGGAGTATTGCCTAGTGTGAA
ATGCATTCATCGTGAGGTATAAACCGCCCCCGCGTGGGTGCGGCTCGATGTCTGGCCTC
CCAGGCTGGCAGCTAACACGGAAGTGTGCCTCGTTCCACTCAGAGGGCATGAGGCAGT
ACCTACGGATCTCAACAAGCCTAGTCCCCATGATACGTTCAGCTGGGCCAGGTGGGAT
TAATACGCCGGGGTCCCGTCGCGGGATGTTTGAGGGGGAGATACGTTCCGGCCATATA
CTTTTTGTCCGCATCCCTTGGGGAAATCCATTACGCTGCCGTTGACGTCGGTAACGCCG
CTTCATTAACGTCGAACCAGGGGGACCAAACTACACCATGTTATCTGTATGCCCAGTTG
TTGTTGCGGTTCAGGGTACGGGGCTGTGAGGCACGGGCGCGCTCCTGGTGGGAACTTG
TCCTCCGGACTTTAATGGCTACTCGCCGGGCTGAGACTTAGCAGACCACCCTGACGCAT
TCCAGGCCTCAGATTAGATCACAATTTTGCTTTCATTAGTTCCCAATTACGGTTCCGCAT
CGTCAGGAGGCCTTCGACTAGATCTACTCGTATGGTTCGAGCGGTGATATCGGGTCTAA
AATCCCCTGACCCAACGGGGGGCGGTACGTGTGATCGGAATGTGCACCTTGCGAGATC
TAATCGTAATGACGATGGCTCTTCCACAAGTGAGTGCTGGGGCGCGAGCGGCGGACCA
GATGGCCTTCCGAATGTTCCCCCCCGTGCCGAAGGCTTAACTCGAGGCGGAGCCGTGG
GCTCGCACCGTTGCCGCGCAGGTACACCGCGAGGTCCCTTGAAGGTCCCATCATGAAG

>MS_ERR173201.1

GTCGGGACGCCCTCCCGGCTCTGGCATGGCGCGGTGGCTTTGCGCACGGATCGGATAA
ACTTTCCATTGGACGGCTCGTTCTCAGAAATATCTCGTAGTCTTCTCGTTGGTTACTCCA
ATGAAACGCCTCGTCTGCGGGGGCACTGAACCGCGAGAGCTATTCAAGTGCTCTACTA
CTCGACCGGGGGCAGCTTACAACCGTGGGATCGGCGCGGCCCACCTCCGGTCAACGAG
AGTTACGAGTACCTGGTCCGTTTTCCCGCGTCGGAAGCTACCAACACGATAGTAACTG
AACCAGCGCGAGAGCATTGATACGCCTCGGGGGCGGGCCTCTGGCGCGCCACTGGATC
AGGCCCGTGGCGTGCCCGCCTCGTCAGCGCCACCCATTGCTAAGCGCTGACAGTAATA
GACCCCTCCATAGTAGTTGCCGATGTTGATTCGGTCACCGGCCGAAACGTATGCACTTA
GCACAGGGCAGGTACTACAAAGCGAGAGGGGGATGATTGGCAGGGGCTGCTGACGCG
CCTATCAGCCCGTTCCCCCGCGCCTGCTGTGGCGACCAACTACGCCCCGCAGCGTCCAG
TACCCGAGCAGTCTCTCAACTGGTCCGATTAGACTGTATACACCGCCGTTGGGACGCG
GACTAAACAACTCCCTCATACCCATCCGCCCGTCCGGAGCGGAACGACTCGGCGGTAC
CGGCGTCAGGCCCCCTCGCCTAGACTGCACCATTATGTTGGGAGGTGCGTCGACTGGG
GGCCGGCGACTTGATCCTGGTCGGCCCGTGAAGCCCATCAGTCCCATGGAGACGTTCT
CTCCCATCTATTGGCCTCCGGGGCTCTCCACCAATCGCACCGGAGTCTTGTCTAATATG
AAATTTATTCATCGTGAGGTATAAACCGCCCCCGAGTGGGTGCGGCTTGAAGTCCAGC
CCCCCAGGCCGCCTGCTAACACGGAAGTGTACCCGGTTCCACTCAGAGGGCCTGAGGC
AGTACCTACGGATCTCAAAAACCCTAGTCCCCATGATACGTACAGCTGGGCTAGGTGG
GATTAATACGTCGGGGTCCCATCGCGGGATGTTTGAGGGGGAGATACGTTCCAGCCAT
ATACTCCCTGTTCGCGCCCCTCAGAGAAGTCCATTACGCTGCCGTTGACGTCGTCAATG
CCGCGCCACTAACACCGGACTAGGGGGACCAAACCATACCATGTAATACGTGTACTTG
CCCATTGTCGCGGTTCAGAGTACGGGGCTGTGAGTAATGTACGCGTTCCTGGTGGGAA
CTTGTCCTCTGAACTTGAATAGCTACTCGCCGGAATGGGATTTAGCAAACCACCCCAAC
ATATTCCAGGCCTCTGATCAGATCACAATTTTGCTTTCACCAGTTCCCAATTATGGTTCC
GCATCGTCGGGAGGCCTTCGACTAGATCTGCTCGCATGGTTCGAGCGGTAATATCGGG
TCTGAAATCCCCTGACCCAACGGGAGGCGGTATGCGTGATCGGAATCTGCACCTTGCG
AGAGCTAATCGTGATGACAATGGCTCTTCCACAAGTGAGTGCGGGGGCGCGAGCGGCG
AACCAGATGGTCCTCTGAATATTCCCCCCCGTGCCGAAGGCTGAACTCGAGGCGGGGC
CGTGGTCGCGCACCGTTACCACGCAGGTGCGTCGCGAGGCCCCTTGAAGGTCCCGTCA
TGAAG

>MS_ERR173201.2

GTCGGGATGCCCTTTCGACACCAGCACGGCGCGACGCTTTCGCGCACGTATCAGATAA
GTTTTCCCGTGGACGGCTCGTCCTCAGAAATATCTCGTAGTCCTTTCGTTGGTTACTCCA
ATGAAGCGCCTCGCCTGCGGGGGCACTAAACCGCGAGCGCCATTCGAGTGCTCTACAA
CTCGACCGGGGGCAGCATACAACCGTAGGATCGGCGCGGCTCATTTTCGGCTAGTAGG
GGGTACGACGTCCCGGCCCGTTTTCTCGCGTCGGAGGCTGCCAACACGATAGTAACTG
AACTGGCCCAAACGTATTGATACGCCCAGAGGGCGGGCCTCTGGCGCGCCACTGGATC
AGGCCCGTGGCGTGCCCGCCTCGTCAGCGCCACCCATTGCTAAGCGCTGACAGTAATA
GACCCCTCCATAGTAGTTGCCGATGTTGATTCGGTCACCGGCCGAAACGTATGCACTTA
GCACAGGGCAGGTACTACAAAGCGAGAGGGGGATGATTGGCAGGGGCTGCTGACGCG
CCTATCAGCCCGTTCCCCCGCGCCTGCTGTGGCGACCAACTACGCCCCGCAGCGTCCAG
TACCCGAGCAGTCTCTCAACTGGTCCGATTAGACTGTATACACCGCCGTTGGGACGCG
GACTAAACAACTCCCTCATACCCATCCGCCCGTCCGGAGCGGAACGACTCGGCGGTAC
CGGCGTCAGGCCCCCTCGCCTAGACTGCACCATTATGTTGGGAGGTGCGTCGACTGGG
GGCCGGCGACTTGATCTTGGTCGGCCCGTGAAGCCCATCACTCCCATGGAGACGTTTTC
TCCCACCCATTGGCCCCCGGGGTCCTCCACCAATTGCGCCGGAGTCTTGTCTAATATGA
AATTTATTCATCGTAAGGTATAAACCGCCCCCGCGTGGGTGCGGCTCGAAGTCCGGCTT
CCCAAGCTGGCTGCTAACACGGAAGTGTACCCGGTTCCACTCAGAGGGCATGAGGCAG
TACCTACGGATCTCAACAAGCCTAGTCCCCATGATACGTACAGCTGGGCCAGGTGGGA
TTAATACGCCGGGGTCCCGCCGCGAAACGGCTAGAAGGTAAATACGCCCTGGCCATAT
ACTCTTTGTCCGCATCCCTTGGGGAAATCCATTATGCTGCCGTTGACGTCGTCAACGTC
ACGCCACTAACACCGAACTAGGGGGACCAAACTATACCATGTTATCCGTATACTTACC
CACTGTTGCAGGTCAAGGTATGGGGCTGCAAGTAATGTACATACTCCTGGTGGGAACT
TGTCCTCCGGACTTGAATGGCTACCTGCCGGGCTGGGACTTAGCAAACCACCCTGACG
CATCCCAGGCCTTTGATCGGATCACAATTTTGATTCCACCAGTTCCCAATTACGGTTCC
GCATCGTTGGGAGGCCTTTGGCTAGATCTACCTGCATGGTTCGAGCGGTAATATCGGGT
CTGAAATCCCCTGACCCAACGGGAGGCAGTATGTGTGGTCGGAATCTGCACCTTGCGA
GAGATAATCGTAATGACGATGGACCTTCTACAAGTGAGTGCTGGGGCACGAGCGGCGG
ACCAGATGGTCTTCTGAATGTTCCTTCCCGTGCCGAAGGCTGAACTCGAGGCGGAGCA
GTGGGCGCGTACCGTTGCCACGCAGGTGCACCGCGAGGTCCCTTGAAGATCCCGTCAT
CAAC

>MS_ERR173202.1

GTCGGGACGCCCTCCCGGCTCTGGCATGGCGCGGTGGCTTTGCGCACGGATCGGATAA
ACTTTCCATTGGACGGCTCGTTCTCAGAAATATCTCGTAGTCTTCTCGTTGGTTACTCCA
ATGAAACGCCTCGTCTGCGGGGGCACTGAACCGCGAGAGCTATTCAAGTGCTCTACTA
CTCGACCGGGGGCAGCTTACAACCGCGGGATTGGCACGGTCTACCTCCGTCCAACAGG
AGTTACGACGACCCGGTCCCCCCTCCCGCGTCGAAGGCTGCCAACACGATAGTAAGCG
AACTGGCCCAAACGTATTAATACGTACAGGGGGCGGGCCTCTGGCGCGCCACTGGATC
AGGCCCGTGGCGTGCCCGCCTCGTCAGCGCCACCCATTGCTAAGCGCTGACAGTAATA
GACCCCTCCATAGTAGTTGCCGATGTTGATTCGGTCACCGGCCGAAACGCGCGCGCTC
AGCACAAGGCAGGTACCACGGAGCGAAAGGTGGATGATTGGCAGGGGCTGCTGACGC
GCCTATCAGCCCGTTCCCCCGCGCCTGCTGTGGCGACCAACTACGCCCCGCAGCGTCCA
GTACCCGAGCAGTCTCTCAACTGGTCCGATTAGACTGTATACACCGCCGTTGGGACGC
GGACTAAACAACTCCCTCATACCCATCCGCCCGTCCGGAGCGGAACGACTCGGCGGTA
CCGGCGTCAGGCCCCCTCGCCTAGACTGCACCATTATGTTGGGAGGTGCGTCGACTGG
GGGCCGGCGACTTGATCCTGGTCGGCCCGTGAAGCCCATCAGTCCCATGGAGACGTTC
TCTCCCATCTATTGGCCTCCGGGGCTCTCCACCAATCGCACCGGAGTCTTGTCTAATAT
GCAATTTATTCATCGTGAGGTATAAACCGCCCCCGAGTGGGTGCGGCTTGAAGTCCAG
CCCCCCAGGCCGCCTGCTAACACGGAAGTGTACCCGGTTCCACTCAGAGGGCATGAGG
CAGTACCTACGGATCTCAACAAGCCTAGTCCCCATGATACGTACAGCTGGGCCAGGTG
GGATTAATACGCCGGGGTCCCGTTGCGGGATGTCTAGGGGGGAGATACGTTCCGGCCA

TATACTCTTTGTCCGCATCCCTTGGGGAAATCCATTATGCTCCCGTTGACGTCGTCAAC
GTCACGCCACTAACACCGAACTAGGGGGACCAAACTATACCATGTTATCCGTATACTT
ACCCACTGTTGCAGGTCAAGGTATGGGGCTGCAAGTAATGTACATACTCCTGGTGGGA
GCTTGTCCTCCGGACTCGAATGGCTACTCGCCGGGCTGGGACTTAGCAAACCACTCTGA
CGCATCTCCGGCCTCAGATTAGATCACAATTTTGCTTTCTCCATTTCCTAATTACCGTTC
CGCATCGTCGGGAGGCCTTCGACTAGATTTACTCGCATGGTTTGAGTGATACAATCAAG
TCTGAAATCCTCTGACCCAACGTGAAGCGGTATGCGCAGTCGGGATCTGCACCTTGCA
AGAGCTAATCGTAATGACGATGGACCTTCTACAAGTGAGTGCTGTAGCGCCAGCAGCG
GACCAGATTGTTTTCCGGATGTTCCCCCTCGTGCCGAAGGCTGAACTCAGGGCGGGGC
CGTGGGCGCGCATCGTTGCCACGCAGGTACACCGTGAGGCACCTTGAAGGTCCCGTCA
TGAAG

>MS_ERR173202.2

GTCGGGATGCCTTCCCAACTCCGGCACGGCGCGGCGCCTTCGCGCACGGATCGGATAA
GCTTACCCGTGGATGGCTCGTTCTCAGAAATATCTCGTAGTCTTCTCGTTGGTTACTCCA
ATGAAGCGCCGCGTCTGCGTGGGCACTGAACCGCGAGAGCTATTCAAGTGCTCTACTA
CTCGACCGGGGGCAGCTTACAACCGTGGGATCGGCGCGGCCCACCTCCGGTCAACGAG
AGTTACGAGTACCTGGTCCGTTTTCCCGCGTCGGAAGCTACCAACACGATAGTAACTG
AACTAGCGCAAACGCATTGATACGCCTCGGGGGCGGGCCTCTGGCGCGCCACTGGATC
AGGCCCGTGGCGTGCCCGCCTCGTCAGCGCCACCCATTGCTAAGCGCTGACAGTAATA
GACCCCTCCATAGTAGTTGCCGATGTTGATTCGGTCACCGGCCGAAACGTATGCACTTA
GCACAGGGCAGGTACTACGGAGCGAGAGGGGGATGATTGGCAGGGGCTGCTGACGCG
CCTATCAGCCCGTTCCCCCGCGCCTGCTGTGGCGACCAACTATGCCCCGCAGCGTCCAG
TACCCGAGCAGTCTCTCAACTGGTCCGATTAGACTGTATACACCGCCGTTGGGACGCG
GACTAAACAACTCCCTCATACCCATCCGCCCGTCCGGAGCGGAACGACTCGGCGGTAC
CGGCGTCAGGCCCCCTCGCCTAGACTGCACCATTATGTTGGGAGGTGCGTCGACTGGG
GGCCGGCGACTTGATCCTGGTCGGCCCGTGAAGCCCATCAGTCCCATGGAGACGTTCT
CTCCCATCTATTGGCCTCCGGGGCTCTCCACCAATCGCACCGGAGTCTTGTCTAATATG
AAATTTATTCATCGTGAGGTATAAACCGCCCCCGAGTGGGTGCGGCTTGAAGTCCAGC
CCCCCAGGCCGCCTGCTAACACGGAAGTGTACCCGGTTCCACTCAGAGGGCATGAGGC
AGTACCTACGGATCTCAACAAGCCTAGTCCCCATGATACGTACAGCTGGGCCAGGTGG
GATTAATACGCCGGGGTCCCGCCGCGAAACGGCTAGAAGGTAAATACGCCCTGGCCAT
ATACTCTTTGTCCGCATCCCTTGGGGAAATCCATTATGCTGCCGTTGACGTCGTCAACG
TCACGCCACTAACACCGAACTAGGGGGACCAAACTATACCATGTTATCCGTATACTTA
CCCACTGTTGCAGGTCAAGGTATGGGGCTGCAAGTAATGTACATACTCCTGGTGGGAA
CTTGTCCTCCGGACTTGAATGGCTACCTGCCGGGCTGGGACTTAGCAAACCACCCTGAC
GCATCCCAGGCCTTTGATCGGATCACAATTTTGATTCCACCAGTTCCCAATTACGGTTC
CGCATCGTTGGGAGGCCTTTGGCTAGATCTACCTGCATGGTTCGAGCGGTAATATCGGG
TCTGAAATCCCCTGACCCAACGGGAGGCAGTATGTGTGGTCGGAATCTGCACCTTGCG
AGAGATAATCGTAATGACGATGGACCTTCTACAAGTGAGTGCTGGGGCACGAGCGGCG
GACCAGATGGTCTTCTGAATGTTCCTTCCCGTGCCGAAGGCTGAACTCGAGGCGGAGC
AGTGGGCGCGTACCGTCGACATGCAGGTGCACCGCGAGGTCCCTTGAAGATCCCGTCA
TCAAC

>AWH_ERR173203.1

GTCGGGATGCCTTCCCAACTCTGGCACGGCGCGGCGCCTTCGCGCATGGATCGTATAA
GCTTTCCCTTGGACGGCTCATCCTCGGAAATATCTCGTAGTCTTCTCGTTGGTTGCTCCA
ATGCCGCGCCGCGTCTGCTTGGATACTGAACCGCGAGAGCTATTCAAGTGCTCTACTAC
TCGACCGGGGGCAGCATACAGCCGTGGGATCGGCGCGGCCCACCTCCGGCCAGCGGG
AGTTACGAGTACCCGGCCCGTTTTCCCGTGTCGGAGGCTGCCAACACGATAGTAACTG

AACTGGCCCAAACGTATTGATACGCCCCGGGGGCGGCCTCTGGCGCGCCACTGGATC
AGGCCCGTGGCGTGCCCGCCTCGTCAGCGCCACCCATTGCTAAGCGCTGACAGTAATA
GACCCCTCCATAGTAGTTGCCGATGTTGACCCGATCACCGGCCGAAACGTATGCACTC
AGCACAGGGCAGGCACCACGGAGCGAAAGGTGGATGATGGGCAGGGGCTGCTGGCGC
GCCTACCAAACTGTTCCTCCGCGCCTGCTGGAGCGACCAACTACGCCCCGCGGCGACC
GGTACCCGAGCAGTCTCTCAACTGGTTCGATTAGACTGTGTACACCGCCGTTGGGACGC
GGACTAAACAACTCCCTCATACCCATCCGCCCGTCCGGAGCGGAACGACTCGGCGGTA
CCGGCGTCAGGCCCCCTCGCCTAGGCTGCACCATTATGTTGGGAGGTGCGTCGACTGG
GGGCCGGCGACTTGAGCTCGATCGTCCCGTGAAGCCCATCGGTCCCATGGAGACGTTC
TCTCCCATCCATTGGCTTCCGGGGCTCTCCACCAATCGCACCGGAGCATTGCCTAATGT
GAAATGTGTTCATCGTGGGGTATAAACCGCCCCCGCGTGAGTGCGGCTCGAAATCCGG
CCCCTCGGACTGCCAGCTAACACGGAAGTGTGCCTCGTTTCGCTCGGAGGGGACGAGA
CACTCTAGACGGATCTCAACAAGCCTATTCCCCACGATACATACAACCGTGCCCGGTG
GGATTAATACGCCGGGGTCCCGCCGCGGGACGTCTGGGAGGGGAACACGTTCCGGCCA
TATGCTCTCTGTCCGCATCCCTTGGGGAAATCCATTACGCTGCCGTTGACGTCGTTAAC
GCCACGCCACTAATACCGAACTAGGGGAACCAAACTTTACCATGTTGTCCGTGCATCT
ACCCACTGTTGCGATTCAGGGTACGGGGCTGTGAGTAATGTACACGCTCCTGGTGGGA
GCTTGTCCTCCGGACTTGAATGGCTACTCGCCGGGCTGGGACTTAGCAAACCACCCTGA
CGCATTCCAGGCCTCAGATCAGATCACAATTTTGCTTTCACCAGTTCCCAATTACGGTT
CCGCATCGTCGGGAGGCCTTCGACTAGATCTACTCGCATGGTTCGAGCGGTAATATTGG
GTCTGAAATCCCCTGACCCAACGGGAGGCGGTACGTGTGATCGGAATCTGCACCTTGC
GAGAGCTAATCGTAATGACGATTGCTCTTCCACAAGTGAGTGCTGGGGCGCGAGCGGC
GGGCTGGATGGTCCTCCGAATGTTCCCCCCTATGCCGAAGGCTGAACTCGAGGCTGAG
CCGTGGGCGCGCACCGTTGCCACGCAGGTGCACCGCGAGGTCCCTTGAAGGTCCCGTC
ATGAAG

>AWH_ERR173203.2

GTCGGGATGCCTTCCCAACTCTGGCACGGCGCGGCGCCTTCGCGCACGGATCGGATAA
GCTTTCCCTTGGACGGCTCGTCCTCGGAAATATCTCGTAGTCTTCTCGTTGGTTGCTCCA
ATGCCGCGCCGCGTCTGCTTGGATACTGAACCGCGAGAGCTATTCAAGAGCTCTACTA
CTCGACCGGGGGCAGCATACAGCCGTGGGATCGGCGCGGCCCACCTCCGGCCAGCGGG
AGTTACGAGTACCCGGCCCGTTTTCCCGCGTCGGAGGCTGCCAACACGATAGTAACTG
AACTGGCCCAAACGTATTGATACGCCCCGGGGGCGGCCTCTGGCGCGCCACTGGATC
AGGCCCGTGGCGTGCCCGCCTCGTCAGCGCCACCCATTGCTAAGCGCTGACAGTAATA
GACCCCTCCATAGTAGTTGCCGATGTTGACCCGATCACCGGCCGAAACGTATGCACTC
GACACAGGGCAGGTACCACGGAGCGAAAGGTGGATGATTGGCAGGGGCTGCTGGCGC
GCCTACCAAACTGTTCCTCCGCGCCTGCTGGAGCGACCAACTACGCCCCGCAGCGACC
GGTACCCGAGCAGTCTCTCAACTGGTTCGATTAGACTGTATACACCGCCGTTGGGACGC
GGACTAAACAACTCCCTCATACCCATCCGCCCGTCCGGAGCGGAACGACTCGGCGGTA
CCGGCGTCAGGCCCCCTCGCCTAGGCTGCACCATTATGTTGGGAGGTGCGTCGACTGG
GGGCCGGCGTCTTGAGCTCGGTCGTCCCGTGAAGCCCATCAGTCCCATGGAGACGTTCT
CTCCCATCCATTGGCCTCCGGGGCTCTCCACCAATCGCACCGGAGTATTGCCTAATGTG
AAATGTGTTCATCGTGGGGTATAAACCGCCCCCGCGTGAGTGCGGCTCGAAATCCGGC
CCCTCGGGCTGCCAGCTAACACGGAAGTGTGCCTCGTTTCGCTCAGAGGGGACGAGAC
ACTCTAGACGGATCTCAACAAGCCTAGTCCCCACGATACATACAACCGTGCCCGGTGG
GATTAATACGCCGGGGTCCCGCCGCGGGACGTCTGGGAGGGGAACACGTTCCGGCCAT
ATGCTCTCTGTCCGCATCCCTTGGGGAAATCCATTACGCTGCCGTTGACGTCGTTAACG
CCACGCCACTAATACCGAACTAGGGGGACCAAACTATACCATGTTATCCGTGCACCTA
CCCACTGTTGCGATTCAGGGTACGGGGCTGTGAGTAATGTACACGCTTCTGGTGGGAG
CTTGTCCCCCGGACTTGAATGGCTACTCGCCGGGCTGGGACTTAGCAAACCACCCTGAC
GCTTTCCAGACCCCAGATCAGATTACAGTTTTGCTTTCACCAGTTCCCACTTACGGTTCC

GCATCGTCGGGAGGCCTTCGACTAGATCTACTCGCATGGTTCGAGCGGTAATATTGGGT
CTGAAATCCCCTGACCCGACGGGAGGCGGTACGTGTGATCGGAATCTGCACCTTGCGA
GAGCTAATCGTAATGACGATTGCTCTTCCACCTCTGAGTGCTGGGGCGCGAGCGGCGG
GCTGGATGGTCCTCCAAATGTTCCCCCCTATGCCGAAGGCTGAACTCGAGGCTGAGCC
GTGGGCGCGCACCGTTGCCACGCAGGTGCACCGCGAGGTCCCTTGAAGGTCCCGTCAT
GAAG

>PT_ERR173204.1

GTCGGGATGCTTTCCCAACTCCGGCACGGCGCGGCGCCTTCGCGCACGGATCGGATAA
GCTTTCCCGTGGACGGCTCGTCCTCAGAAACATTTCGTAGTCTTCTCGTTGGTTACTCCA
CTGCCGCGCCGCGTCTGCTTGGATACTGAACCGCGAGAGCTATTCAAGTGCTCTACTAC
TCGACCGGGGGCAGCTTACAACCGTGGGATCGGCGTGGCCCACCTCCGGCCAACGAGA
GTTACGAGTACCCGGCCCGTTTTCCCGCGTCGGAGGCTGCCAACACGATAGTAGCTGA
ACTGGCCCAGACGTATTGATACGCCCCGGGGGCGGGCCTCTGGCGCGCCACTGGATCA
GGCCCGTGGCGTGCCCGCCTCGTCAGCGCCACCCATTGCTAAGCGCTGACAGTAATAG
ACCCCTCCATAGTAGTTGCCGATGTTAATTCGGTCACCGGCCGAAACGTATGCACTTAG
CACAGGGCAGGTACTACAAAGCGAGAGGTGGACGATTGGCAAAGGCTGCTGGCGAGC
CTACCAACCTGTTCCTCCGCGCCTGCTGGAGCGGCCAACTACGCCCCGCAGCGACCGG
TACCCGAGCAGTCTCTCAACTGGTTCGATGAGACTGTATACACCGTCGTTGGGACGCG
GACTAAACAACCTCCTCATACCAATCCGCCCGTCCGGAGCGGAACGACTCGGCGGTAC
CGGCGTCAGGCCCCCTCGCCTAGGCTGCACCATTATGTTGGGAGGTGCGTCGACTGGG
GGCCGGCGACTTGAGCTCGGTCGTCCCGTGAAGCCCATCAGTCCCATGGAGACGTTCT
CTCCCATCCATTGGCCTCCGGGGCTCTCCACCAATCGCACCGGAGTCTTGTCTAGTGTT
AAATGTATTCATCGTGGGGTATAAACCGCCCCCGCGTAAGTGCGGCTCGAAGTCTGGC
CTCCCAGGCTGCTAGCAAACACGGAAGTGTGCCTCGTCTCGCTCAGAGGGGACAAGAC
ACTCTCGACGGCTCTCAACAAGCCTAGTCCCCACGATACATACAACCGTGCCCGGTGG
GATTAATACGCCGGGGTCCCGTCGCGGGACATCTGGGATGGGAACACGTTCCGACCAT
ATGCTCTCTGTCTGCATCTCTTGGGGAAATCCATTACGCTGCCGTTGACGTCGTTAACG
TCACGCCACTAATACCGAACTAGGGGGGCCAAACTATACCATGTTATCCGTGCGCCTA
CCCACTGTTGCGATTCAGGGTACGGGGCTGTGAGTAGTGTACACACTCTTGATGGGAG
CTTATCCTCCGGACTTGAATGGCTACTCGCCGGGCTGGGACTTAGCAAACCACCCTGAC
GCATTCCAGGCCTCTGATCAGATCACAGTTTTGCTTTCACCAGTTCCCAATTACGGTTC
CGCATCGTCGGGAGGCCTTCGACTAGACCTACTCGCATGGTTCGAGCGGTAATATCGG
GTCTGAAATCCCCTGACCCAACGGGAGGCGGTACGTGTGATCGGAATCTGCACCTTGC
GAGAGCTAATCGTAATGCCGATGGCTCTTCCACAAGTGAGTGCTGGGGCGCGAGTGGC
GGACCAGATTGTCTTCCGAATGTTCCCCCCCATGTCGAAGGCTGAACTCGAGGCGGAG
CCGTGAGCGCGCACCGTTGCCACGCAGGTGCACCGCGAGGCACCTCGAAGGTCCCGTC
ATGAAG

>PT_ERR173204.2

GTCGGGATGCCTTCCCAACTCTGGCACGGCGCGGCGCCTTCGCGCACGGATCGGATAA
GCTTTCCCGTGGACGGCTCGTCCTCGGAAATATCTCGTAGTCTTCTCGTTGGTTGCTCCA
ACGAAGCGACGAATCTACTTGGATACTGAACCGCGAGAGCTATTCAAGTGCTCTACTA
CTCGACCGGGGGCAGCATACAGCCGTGGGATCGGCGCGGCCCACCTCCGGCCAACGAG
AGTTACGAGTACCCGGCCCGTTTTCCCGCGTCGGAGGCTGCCAACACGATAGTAACTG
AACTGGCCCAAACGTATTGATACGCCCCGGGGGCGGGCCTCTGGCGCGCCACTGGATC
AGGCCCGTGGCGTGCCCGCCTCGTCAGCGCCACCCATTGCTAAGCGCTGACAGTAATA
GACCCCTCCATAGTAGTTGCCGATGTAGGTCTGGTCACCGGCCGAAACGTATGAACGC
AGCACAGGGCAGGTACTACGGAGCGAAAGGTAGCTGATTGGCAGGGGCTGCTGGCGC
GCCTACCAACCTGTTCCTCCGCGCCTGCTGGAGCGACCAACTACGCCCCGCAGCGACC

GGTACCCGAGCAGTCTCTCAACTGGTTCGATGAGACTGTATACACCGTCGTTGGGACG
CGGACTAAACCACCCCCTCATACCCACCCGCCCGTCCGGAGCGGAACGACTCGGCGGT
ACCGGCGTCAGGCCCCCTCGCCTAGGCTGCACCATTATGTTGGGAGGTGCGTCGACTG
GGGGCCGGCGACTTGAGCTCGGTCGTCCCGTGAAGCCCATCAGTCCCATGGAGACGTT
CTCTCCCATCCATTGGCCTCCGGGGCTCTCCACCAATCGCACCGGAGTCTTGTCTAGTG
TTAAATGTATTCATCGTGGGGTATAAACCGCCCCCGCGTAAGTGCGGCTCGAAGTCTG
GCCTCCCAGGCTGCTAGCAAACACGGAAGTGTGCCTCGTTTCGCTCAGAGGGGACAAG
ACACTCTCGACGGCTCTCAACAAGCCTAGTCCCCACGATACATACAACCGTGCCCGGT
GGGATTAATACGCCGGGGTCCCGTCGCGGGACATCTGGGATGGAACACGTTCCGACC
ATATGCTCTCTGTCTGCATCTCTTGGGGAAATCCATTACGCTGCCGTTGACGTCGTTAA
CGTCACGCCACTAATACCGAACTAGGGGGGCCAAGCTATACCATGTAATCCGTGCGCC
TACCCACTGTTGCGATTCAGGGTACGGGGCTGTGAGTAATGTACACACTCCTGGTGTGA
GCTTGTCCTCCGGACTTGAATGGCTACTCGCCGGGCTGGGACTTAGCAAACCACCCTGA
CGCATTCCAGGCCTCTGATCAGATCACAATTTTGCTTTCACCAGTTCCCAATTACGGTT
CCGCATCGTCGGGAGGCCTTCGACTAGATCTACTCGCATGGTTCGAGCGGTAATATCG
GGTCTGAAATCCCCTGACCCAACGGGAGGCGGTACGTGTGATCAGGATCTGCACCTTG
CGAGAGCCAAGCGTAATGACGGTGGCTCTTCCACAAGTAAAAGCTCGGGCGCGAGCG
GCGGGCTGGATTGTCCCCCGAATGTTCCCCCCTATGCCGAAGGCTGAACTCGAGGCTG
AGCCGTGGGCGCGCACCGTTGCCACGCAGGTGCACCGCGAGGTCCCTTGAAGGTCCCG
TCACGAAG

>PT_ERR173205.1

GTCGGGATGCTTTCCCAACTCCGGCACGGCGCGGCGCCTTCGCGCACGGATCGGATAA
GCTTTCCCGTGGACGGCTCGTCCTCAGAAACATTTCGTAGTCTTCTCGTTGGTTACTCCA
CTGCCGCGCCGCGTCTGCTTGGATGTTGAACCGCGAGAGCTATTCAAGTGCTTTACTAC
TCGACCGAGGGCAGCTTACAACCGTGGGATCGGCGTGGCCCACCTCCGGCCAACGAGA
GTTACGAGTACCCGGCCCGTTTTCCCGCGTCGGAGGCTGCCAACACGATAGTAGCTGA
ACTGGCCCAGACGTATTGATACGCCCCGGGGGCGGGCCTCTGGCGCGCCACTGGATCA
GGCCCGTGGCGTGCCCGCCTCGTCAGCGCCACCCATTGCTAAGCGCTGACAGTAATAG
ACCCCTCCATAGTAGTTGCCGATGTTAATTCGGTCACCGGCCGAAACGTATGCACTTAG
CATAGGGCAGGTACTACAAAGCGAGAGGTGGACGATTGGCAAAGGCTGCTGGCGAGC
CTACCAACCTGTTCCTCCGCGCCTGCTGGAGCGGCCAACTACGCCCCGCAGCGACCGG
TACCCGAGCAGTCTCTCAACTGGTTCGATGAGACTGTATACACCGTCGTTGGGACGCG
GACTAAACAACCTCCTCATACCAATCCGCCCGTCCGGAGCGGAACGACTCGGCGGTAC
CGGCGTCAGGCCCCCTCGCCTAGGCTGCACCATTATGTTGGGAGGTGCGTCGACTGGG
GGCCGGCGACTTGAGCTCGGTCGTCCCGTGAAGCCCATCAGTCCCATGGAGACGTTCT
CTCCCATCCATTGGCCTCCGGGGCTCTCCACCAATCGCACCGGAGTCTTGTCTAGTGTT
AAATGTATTCATCGTGGGGTATAAACCGCCCCCGCGTAAGTGCGGCTCGAAGTCTGGC
CTCCCAGGCTGCTAGCAAACACGGAAGTGTGCCTCGTTTCGCTCAGAGGGGACAAGAC
ACTCTCGACGGCTCTCAACAAGCCTAGTCCCCACGATACATACAACCGTGCCCGGTGG
GATTAATACGCCGGGGTCCCGTCGCGGGACATCTGGGATGGGAACACGTTCCGACCAT
ATGCTCTCTGTCTGCATCTCTTGGGGAAATCCATTACGCTGCCGTTGACGTCGTTAACG
TCACGCCACTAATACCGAACTAGGGGGGCCAAACTATACCATGTTATCCGTGCGCCTA
CCCACTGTTGCGATTCAGGGTACGGGGCTGTGAGTAGTGTACACACTCTTGATGGGAG
CTTATCCTCCGGACTTGAATGGCTACTCGCCGGGCTGGGACTTAGCAAACCACCCTGAC
GCATTCCAGGCCTCTGATCAGATCACAGTTTTGCTTTCACCAGTTCCCAATTACGGTTC
CGCATCGTCGGGAGGCCTTCGACTAGACCTACTCGCATGGTTCGAGCGGTAATATCGG
GTCTGAAATCCCTTGACCCAACGGGAGGCGGTACGTGTGATCGGAATCTGCACCTTGC
GAGAGCTAATCGTAATGCCGATGGCTCTTCCACAAGTGAGTGCTGGGGCGCGAGTGGC
GGACCAGATTGTCTTCCGAATGTTCCCCCCCGTGTCGAAGGCTGAACTCGAGGCGGAG

CCGTGAGCGCGCACCGTTGCCACGCAGGTGCACCGCGAGGCACCTCGAAGGTCCCGTC
ATGAAG

>PT_ERR173205.2

GTCGGGATGCTTTCCCAACTCCGGCACGGCGCGGCGCCTTCGCGCACGGATCGGATAA
GCTTTCCCGTGGACGGCTCGTCCTCAGAAACATTTCGTAGTCTTCTCGTTGGTTACTCCA
CTGCCGCGCCGCGTCTGCTTGGATGTTGAACCGCGAGAGCTATTCAAGTGCTTTACTAC
TCGACCGGGGGCAGCTTACAACCGTGGGATCGGCGTGGCCCACCTCCGGCCAACGAGA
GTTACGAGTACCCGGCCCGTTTTCCCGCGTCGGAGGCTGCCAACACGATAGTAGCTGA
ACTGGCCCAGACGTATTGATACGCCCCGGGGGCGGGCCTCTGGCGCGCCACTGGATCA
GGCCCGTGGCGTGCCCGCCTCGTCAGCGCCACCCATTGCTAAGCGCTGACAGTAATAG
ACCCCTCCATAGTAGTTGCCGATGTTAATTCGGTCACCGGCCGAAACGTATGCACTTAG
CACAGGGCAGGTACTACAAAGCGAGAGGTGGACGATTGGCAAAGGCTGCTGGCGAGC
CTACCAACCTGTTCCTCCGCGCCTGCTGGAGCGGCCAACTACGCCCCGCAGCGACCGG
TACCCGAGCAGTCTCTCAACTGGTTCGATGAGACTGTATACACCGTCGTTGGGACGCG
GACTAAACAACCTCCTCATACCAATCCGCCCGTCCGGAGCGGAACGACTCGGCGGTAC
CGGCGTCAGGCCCCCTCGCCTAGGCTGCACCATTATGTTGGGAGGTGCGTCGACTGGG
GGCCGGCGACTTGAGCTCGGTCGTCCCGTGAAGCCCATCAGTCCCATGGAGACGTTCT
CTCCCATCCATTGGCCTCCGGGGCTCTCCACCAATCGCACCGGAGTCTTGTCTAGTGTT
AAATGTATTCATCGTGGGGTATAAACCGCCCCCGCGTAAGTGCGGCTCGAAGTCTGGC
CTCCCAGGCTGCTAGCAAACACGGAAGTGTGCCTCGTTTCGCTCAGAGGGGACAAGAC
ACTCTCGACGGCTCTCAACAAGCCTAGTCCCCACGATACATACAACCGTGCCCGGTGG
GATTAATACGCCGGGGTCCCGTCGCGGGACATCTGGGATGGGAACACGTTCCGACCAT
ATGCTCTCTGTCTGCATCTCTTGGGGAAATCCATTACGCTGCCGTTGACGTCGTTAACG
TCACGCCACTAATACCGAACTAGGGGGGCCAAACTATACCATGTTATCCGTGCGCCTA
CCCACTGTTGCGATTCAGGGTACGGGGCTGTGAGTAGTGTACACACTCTTGATGGGAG
CTTATCCTCCGGACTTGAATGGCTACTCGCCGGGCTGGGACTTAGCAAACCACCCTGAC
GCATTCCAGGCCTCTGATCAGATCACAGTTTTGCTTTCACCAGTTCCCAATTACGGTTC
CGCATCGTCGGGAGGCCTTCGACTAGACCTACTCGCATGGTCCGAGCGGTAATATCGG
GTCTGAAATCCCTTGACCCAACGGGAGGCGGTACGTGTGATCGGAATCTGCACCTTGC
GAGAGCTAATCGTAATGCCGATGGCTCTTCCACAAGTGAGTGCTGGGGCGCGAGTGGC
GGACCAGATTGTCTTCCGAATGTTCCCCCCCGTGTCGAAGGCTGAACTCGAGGCGGAG
CCGTGAGCGCGCACCGTTGCCACGCAGGTGCACCGCGAGGCACCTCGAAGGTCCCGTC
ATGAAG

>PT_ERR173206.1

GTCGGGATGCTTTCCCAACTCCGGCACGGCGCGGCGCCTTCGCGCACGGATCGGATAA
GCTTTCCCGTGGACGGCTCGTCCTCAGAAACATTTCGTAGTCTTCTCGTTGGTTACTCCA
CTGCCGCGCCGCGTCTGCTTGGATGTTGAACCGCGAGAGCTATTCAAGTGCTTTACTAC
TCGACCGGGGGCAGCTTACAACCGTGGGATCGGCGTGGCCCACCTCCGGCCAACGAGA
GTTACGAGTACCCGGCCCGTTTTCCCGCGTCGGAGGCTGCCAACACGATAGTAGCTGA
ACTGGCCCAGACGTATTGATACGCCCCGGGGGCGGGCCTCTGGCGCGCCACTGGATCA
GGCCCGTGGCGTGCCCGCCTCGTCAGCGCCACCCATTGCTAAGCGCTGACAGTAATAG
ACCCCTCCATAGTAGTTGCCGATGTTAATTCGGTCACCGGCCGAAACGTATGCACTTAG
CATAGGGCAGGTACTACAAAGCGAGAGGTGGACGATTGGCAGGGGCTGCTGGCGCGC
CTACCAACCTGTTCCTCCGCGCCTGCTGGAGCGGCCAACTACGCCCCGCAGCGACCGG
TACCCGAGCAGTCTCTCAACTGGTTCGATGAGACTGTATACACCGTCGTTGGGACGCG
GACTAAACAACCTCCTCATACCAATCCGCCCGTCCGGAGCGGAACGACTCGGCGGTAC
CGGCGTCAGGCCCCCTCGCCTAGGCTGCACCATTATGTTGGGAGGTGCGTCGACTGGG
GGCCGGCGACTTGAGCTCGGTCGTCCCGTGAAGCCCATCAGTCCCATGGAGACGTTCT

CTCCCATCCATTGGCCTCCGGGGCTCTCCACCAATCGCACCGGAGTCTTGTCTAGTGTT
AAATGTATTCATCGTGGGGTATAAACCGCCCCCGCGTGAGTGCGGCTCGAAGTCTGGC
CTCCCAGGCTGCTAGCTAACACGGAAGTGTGCCTCGTTTCGCTCAGAGGGGACGAGAC
ACTCTCGACGGCTCTCAACAAGCCTAGTCCCCACGATACATACAACCGTGCCCGGTGG
GATTAATACGCCGGGGTCCCGTCGCGGGACATCTGGGATGGGAACACGTTCCGGCCAT
ATGCTCTCTGTCCGCATCTCTTGGGGAAATCCATTACGCTGCCGTTGACGTCGTTAACG
TCACGCCACTAATACCGAACTAGGGGGACCAAACTATACCATGTTATCCGTGCGCCTA
CCCACTGTTGCGATTCAGGGTACGGGGCTGTGAGTAATGTACACACTCCTGGTGGGAG
CTTGTCCTCCGGACTTGAATGGCTACTCGCCGGGCTGGGACTTAGCAAACCACCCTGAC
GCATTCCAGGCCTCTGATCAGATCACAATTTTGCTTTCACCAGTTCCCAATTACGGTTC
CGCATCGTCGGGAGGCCTTCGACTAAATCTACTCGCATGGTTCGAGCGGTAATATCGG
GTCTGAAATCCCCTGACCCAACGGGAGGCGGTACGTGTGATCAGGATCTGCACCTTGC
GAGAGCCAAGCGTAATGACGGTGGCTCTTCCACAAGTAAAAGCTCGGGCGCGAGCGG
CGGGCTGGATGGTCCCCCGAATGTTCCCCCCTATGCCGAAGGCTGAACTCGAGGCTGA
GCCGTGGGCGCGCACCGTTGCCACGCAGGTGCACCGCGAGGTCCCTTGAAGGTCCCGT
CACGAAG

>PT_ERR173206.2

GTCGGGATGCCTTCCCAACTCTGGCACGGCGCGGCGCCTTCGCGCACGGATCGGATAA
GCTTTCCCGTGGACGGCTCGTCCTCGGAAATATCTCGTAGTCTTCTCGTTGGTTGCTCCA
ACGAAGCGACGAATCTACTTGGATACTGAACCGCGAGAGCTATTCAAGTGCTCTACTA
CTCGACCGGGGGCAGCATACAGCCGTGGGATCGGCGCGGCCCACCTCCGGCCAACGAG
AGTTACGAGTACCCGGCCCGTTTTCCCGCGTCGGAGGCTGCCAACACGATAGTAACTG
AACTGGCCCAAACGTATTGATACGCCCCGGGGGCGGGCCTCTGGCGCGCCACTGGATC
AGGCCCGTGGCGTGCCCGCCTCGTCAGCGCCACCCATTGCTAAGCGCTGACAGTAATA
GACCCCTCCATAGTAGTTGCCGATGTAGGTCTGGTCACCGGCCGAAACGTATGAACGC
AGCACAGGGCAGGTACTACGGAGCGAAAGGTAGCTGATTGGCAGGGGCTGCTGGCGC
GCCTACCAACCTGTTCCTCCGCGCCTGCTGGAGCGACCAACTACGCCCCGCAGCGACC
GGTACCCGAGCAGTCTCTCAACTGGTTCGATGAGACTGTATACACCGTCGTTGGGACG
CGGACTAAACCGCCCCCTCATACCCACCCGCCCGTCCGGAGCGGAACGACTCGGCGGT
ACCGGCGTCAGGCCCCCTCGCCTAGGCTGCACCATTATGTTGGGAGGTGCGTCGACTG
GGGGCCGGCGACTTGAGCTCGGTCGTCCCGTGAAGCCCATCAGTCCCATGGAGACGTT
CTCTCCCATCCATTGGCCTCCGGGGCTCTCCACCAATCGCACCGGAGTCTTGTCTAGTG
TTAAATGTATTCATCGTGGGGTATAAACCGCCCCCGCGTAAGTGCGGCTCGAAGTCTG
GCCTCCCAGGCTGCTAGCAAACACGGAAGTGTGCCTCGTCTCGCTCAGAGGGGACAAG
ACACTCTCGACGGCTCTCAACAAGCCTAGTCCCCACGATACATACAACCGTGCCCGGT
GGGATTAATACGCCGGGGTCCCGTCGCGGGACATCTGGGATGGGAACACGTTCCGACC
ATATGCTCTCTGTCTGCATCTCTTGGGGAAATCCATTACGCTGCCGTTGACGTCGTTAA
CGTCACGCCACTAATACCGAACTAGGGGGGCCAAGCTATACCATGTAATCCGTGCGCC
TACCCACTGTTGCGATTCAGGGTACGGGGCTGTGAGTAATGTACACACTCCTGGTGTGA
GCTTGTCCTCCGGACTTGAATGGCTACTCGCCGGGCTGGGACTTAGCAAACCACCCTGA
CGCATTCCAGGCCTCTGATCAGATCACAATTTTGCTTTCACCAGTTCCCAATTACGGTT
CCGCATCGTCGGGAGGCCTTCGACTAGATCTACTCGCATGGTTCGAGCGGTAATATCG
GGTCTGAAATCCCCTGACCCAACGGGAGGCGGTACGTGTGATCAGGATCCGCACCTTG
CGAGAGCCAAGCGTAATGACGGTGGCTCTTCCACAAGTAAAAGCTCGGGCGCGAGCG
GCGGGCTGGATGGTCCCCCGAATGTTCCCCCCTATGCCGAAGGCTGAACTCGAGGCTG
AGCCGTGGGCGCGCACCGTTGCCACGCAGGTGCACCGCGAGGTCCCTTGAAGGTCCCG
TCACGAAG

>PT_ERR173207.1

GTCGGGATGCCTTCCCAACTCTGGCACGGCGCGGCGCCTTCGCGCACGGATCGGATAA
GCTTTCCCGTGGACGGCTCGTCCTCGGAAATATCTCGTAGTCTTCTCGTTGGTTGCTCCA
ACGAAGCGACGAATCTACTTGGATACTGAACCGCGAGAGCTATTCAAGTGCTCTACTA
CTCGACCGGGGGCAGCATACAGCCGTGGGATCGGCGCGGCCCACCTCCGGCCAACGAG
AGTTACGAGTACCCGGCCCGTTTTCCCGCGTCGGAGGCTGCCAACACGATAGTAACTG
AACTGGCCCAAACGTATTGATACGCCCCGGGGGCGGGCCTCTGGCGCGCCACTGGATC
AGGCCCGTGGCGTGCCCGCCTCGTCAGCGCCACCCATTGCTAAGCGCTGACAGTAATA
GACCCCTCCATAGTAGTTGCCGATGTAGGTCTGGTCACCGGCCGAAACGTATGAACGC
AGCACAGGGCAGGTACTACGGAGCGAAAGGTAGCTGATTGGCAGGGGCTGCTGGCGC
GCCTACCAACCTGTTCCTCCGCGCCTGCTGGAGCGACCAACTACGCCCCGCAGCGACC
GGTACCCGAGCAGTCTCTCAACTGGTTCGATGAGACTGTATACACCGTCGTTGGGACG
CGGACTAAACCGCCCCCTCATACCCACCCGCCCGTCCGGAGCGGAACGACTCGGCGGT
ACCGGCGTCAGGCCCCCTCGCCTAGGCTGCACCATTATGTTGGGAGGTGCGTCGACTG
GGGGCCGGCGACTTGAGCTCGGTCGTCCCGTGAAGCCCATCAGTCCCATGGAGACGTT
CTCTCCCATCCATTGGCCTCCGGGGCTCTCCACCACTCGCACCGGAGTCTTGTCTAGTG
TTAAATGTATTCATCGTGGGGTATAAACCGCCCCCGCGTGAGTGCGGCTCGAAGTCTG
GCCTCCCAGGCTGCTAGCTAACACGGAAGTGTGCCTCGTTTCGCTCAGAGGGGACAAG
ACACTCTCGACGGCTCTCAACAAGCCTAGTCCCCACGATACATACAACCGTGCCCGGT
GGGATTAATACGCCGGGGTCCCGTCGCGGGACATCTGGATGGGAACACGTTCCGACC
ATATGCTCTCTGTCTGCATCTCTTGGGGAAATCCATTACGCTGCCGTTGACGTCGTTAA
CGTCACGCCACTAATACCGAACTAGGGGGGCCAAGCTATACCATGTAATCCGTGCGCC
TACCCACTGTTGCGATTCAGGGTACGGGGCTGTGAGTAATGTACACACTCCTGGTGTGA
GCTTGTCCTCCGGACTTGAATGGCTACTCGCCGGGCTGGGACTTAGCAAACCACCCTGA
CGCATTCCAGGCCTCTGATCAGATCACAATTTTGCTTTCACCAGTTCCCAATTACGGTT
CCGCATCGTCGGGAGGCCTTCGACTAGATCTACTCGCATGGTTCGAGCGGTAATATCG
GGTCTGAAATCCCCTGACCCAACGGGAGGCGGTACGTGTGATCGGAATCTGCACCTTG
CGAGAGCTAATCGTAATGCCGATGGCTCTTCCACAAGTGAGTGCTGGGGCGCGAGTGG
CGGACCAGATTGTCCCCCGAATGTTCCCCCCTATGCCGAAGGCTGAACTCGAGGCTGA
GCCGTGGGCGCGCACCGTTGCCACGCAGGTGCACCGCGAGGTCCCTTGAAGGTCCCGT
CACGAAG

>PT_ERR173207.2

GTCGGGATGCTTTCCCAACTCCGGCACGGCGCGGCGCCTTCGCGCACGGATCGGATAA
GCTTTCCCGTGGACGGCTCGTCCTCAGAAACATTTCGTAGTCTTCTCGTTGGTTACTCCA
CTGCCGCGCCGCGTCTGCTTGGATACTGAACCGCGAGAGCTATTCAAGTGCTCTACTAC
TCGACCGGGGGCAGCATACAGCCGTGGGATCGGCGCGGCCCACCTCCGGCCAACGAG
AGTTACGAGTACCCGGCCCGTTTTCCCGCGTCGGAGGCTGCCAACACGATAGTAGCTG
AACTGGCCCAGACGTATTGATACGCCCCGGGGGCGGGCCTCTGGCGCGCCACTGGATC
AGGCCCGTGGCGTGCCCGCCTCGTCAGCGCCACCCATTGCTAAGCGCTGACAGTAATA
GACCCCTCCATAGTAGTTGCCGATGTTAATTCGGTCACCGGCCGAAACGTATGAACGC
AGCACAGGGCAGGTACTACAAAGCGAGAGGTGGACGATTGGCAAAGGCTGCTGGCGA
GCCTACCAACCTGTTCCTCCGCGCCTGCTGGAGCGGCCAACTACGCCCCGCAGCGACC
GGTACCCGAGCAGTCTCTCAACTGGTTCGATGAGACTGTATACACCGTCGTTGGGACG
CGGACTAAACAACCTCCTCATACCAATCCGCCCGTCCGGAGCGGAACGACTCGGCGGT
ACCGGCGTCAGGCCCCCTCGCCTAGGCTGCACCATTATGTTGGGAGGTGCGTCGACTG
GGGGCCGGCGACTTGAGCTCGGTCGTCCCGTGAAGCCCATCAGTCCCATGGAGACGTT
CTCTCCCATCCATTGGCCTCCGGGGCTCTCCACCAATCGCACCGGAGTCTTGTCTAGTG
TTAAATGTATTCATCGTGGGGTATAAACCGCCCCCGCGTAAGTGCGGCTCGAAGTCTG
GCCTCCCAGGCTGCTAGCAAACACGGAAGTGTGCCTCGTTTCGCTCAGAGGGGACGAG
ACACTCTCGACGGCTCTCAACAAGCCTAGTCCCCACGATACATACAACCGTGCCCGGT
GGGATTAATACGCCGGGGTCCCGTCGCGGGACATCTGGATGGGAACACGTTCCGGCC

ATATGCTCTCTGTCTGCATCTCTTGGGGAAATCCATTACGCTGCCGTTGACGTCGTTAA
CGTCACGCCACTAATACCGAACTAGGGGGGCCAAGCTATACCATGTAATCCGTGCGCC
TACCCACTGTTGCGATTCAGGGTACGGGGCTGTGAGTAGTGTACACACTCCTGATGTGA
GCTTGTCCTCCGGACTTGAATGGCTACTCGCCGGGCTGGGACTTAGCAAACCACCCTGA
CGCATTCCAGGCCTCTGATCAGATCACAATTTTGCTTTCACCAGTTCCCAATTACGGTT
CCGCATCGTCGGGAGGCCTTCGACTAGATCTACTCGCATGGTTCGAGCGGTAATATCG
GGTCTGAAATCCCCTGACCCAACGGGAGGCGGTACGTGTGATCAGGATCTGCACCTTG
CGAGAGCCAAGCGTAATGACGGTGGCTCTTCCACAAGTAAAAGCTCGGGCGCGAGCG
GCGGGCTGGATGGTCCCCCGAATGTTCCCCCCTATGCCGAAGGCTGAACTCGAGGCGG
AGCCGTGAGCGCGCACCGTTGCCACGCAGGTGCACCGCGAGGCACCTTGAAGGTCCCG
TCACGAAG

>PT_ERR173208.1

GTCGGGATGCTTTCCCAACTCCGGCACGGCGCGGCGCCTTCGCGCACGGATCGGATAA
GCTTTCCCGTGGACGGCTCGTCCTCAGAAACATTTCGTAGTCTTCTCGTTGGTTACTCCA
CTGCCGCGCCGCGTCTGCTTGGATGTTGAACCGCGAGAGCTATTCAAGTGCTTTACTAC
TCGACCGGGGGCAGCTTACAACCGTGGGATCGGCGTGGCCCACCTCCGGCCAACGAGA
GTTACGAGTACCCGGCCCGTTTTCCCGCGTCGGAGGCTGCCAACACGATAGTAGCTGA
ACTGGCCCAGACGTATTGATACGCCCCGGGGGCGGGCCTCTGGCGCGCCACTGGATCA
GGCCCGTGGCGTGCCCGCCTCGTCAGCGCCACCCATTGCTAAGCGCTGACAGTAATAG
ACCCCTCCATAGTAGTTGCCGATGTTAATTCGGTCACCGGCCGAAACGTATGCACTTAG
CATAGGGCAGGTACTACAAAGCGAGAGGTGGACGATTGGCAAAGGCTGCTGGCGAGC
CTACCAACCTGTTCCTCCGCGCCTGCTGGAGCGGCCAACTACGCCCCGCAGCGACCGG
TACCCGAGCAGTCTCTCAACTGGTTCGATGAGACTGTATACACCGTCGTTGGGACGCG
GACTAAACAACCTCCTCATACCAATCCGCCCGTCCGGAGCGGAACGACTCGGCGGTAC
CGGCGTCAGGCCCCCTCGCCTAGGCTGCACCATTATGTTGGGAGGTGCGTCGACTGGG
GGCCGGCGACTTGAGCTCGGTCGTCCCGTGAAGCCCATCAGTCCCATGGAGACGTTCT
CTCCCATCCATTGGCCTCCGGGGCTCTCCACCAATCGCACCGGAGTCTTGTCTAGTGTT
AAATGTATTCATCGTGGGGTATAAACCGCCCCCGCGTAAGTGCGGCTCGAAGTCTGGC
CTCCCAGGCTGCTAGCAAACACGGAAGTGTGCCTCGTTTCGCTCAGAGGGGACAAGAC
ACTCTCGACGGCTCTCAACAAGCCTAGTCCCCACGATACATACAACCGTGCCCGGTGG
GATTAATACGCCGGGGTCCCGTCGCGGGACATCTGGGATGGGAACACGTTCCGACCAT
ATGCTCTCTGTCTGCATCTCTTGGGGAAATCCATTACGCTGCCGTTGACGTCGTTAACG
TCACGCCACTAATACCGAACTAGGGGGGCCAAGCTATACCATGTAATCCGTGCGCCTA
CCCACTGTTGCGATTCAGGGTACGGGGCTGTGAGTAATGTACACACTCCTGGTGTGAG
CTTGTCCTCCGGACTTGAATGGCTACTCGCCGGGCTGGGACTTAGCAAACCACCCTGAC
GCATTCCAGGCCTCTGATCAGATCACAATTTTGCTTTCACCAGTTCCCAATTACGGTTC
CGCATCGTCGGGAGGCCTTCGACTAGATCTACTCGCATGGTTCGAGCGGTAATATCGG
GTCTGAAATCCCCTGACCCAACGGGAGGCGGTACGTGTGATCAGGATCTGCACCTTGC
GAGAGCTAAGCGTAATGACGATGGCTCTTCCACAAGTAAAAGCTCGGGCGCGAGCGGC
GGGCTGGATGGTCCCCCGAATGTTCCCCCCTATGCCGAAGGCTGAACTCGAGGCTGAG
CCGTGGGCGCGCACCGTTGCCACGCAGGTGCACCGCGAGGTCCCTTGAAGGTCCCGTC
ACGAAG

>PT_ERR173208.2

GTCGGGATGCCTTCCCAACTCTGGCACGGCGCGGCGCCTTCGCGCACGGATCGGATAA
GCTTTCCCGTGGACGGCTCGTCCTCGGAAATATCTCGTAGTCTTCTCGTTGGTTGCTCCA
ACGAAGCGACGAATCTACTTGGATACTGAACCGCGAGAGCTATTCAAGTGCTCTACTA
CTCGACCGGGGGCAGCATACAGCCGTGGGATCGGCGCGGCCCACCTCCGGCCAACGAG
AGTTACGAGTACCCGGCCCGTTTTCCCGCGTCGGAGGCTGCCAACACGATAGTAACTG

AACTGGCCCAAACGTATTGATACGCCCCGGGGGCGGGCCTCTGGCGCGCCACTGGATC
AGGCCCGTGGCGTGCCCGCCTCGTCAGCGCCACCCATTGCTAAGCGCTGACAGTAATA
GACCCCTCCATAGTAGTTGCCGATGTAGGTCTGGTCACCGGCCGAAACGTATGAACGC
AGCACAGGGCAGGTACTACGGAGCGAAAGGTAGCTGATTGGCAGGGGCTGCTGGCGC
GCCTACCAACCTGTTCCTCCGCGCCTGCTGGAGCGACCAACTACGCCCCGCAGCGACC
GGTACCCGAGCAGTCTCTCAACTGGTTCGATGAGACTGTATACACCGTCGTTGGGACG
CGGACTAAACCGCCCCCTCATACCCACCCGCCCGTCCGGAGCGGAACGACTCGGCGGT
ACCGGCGTCAGGCCCCCTCGCCTAGGCTGCACCATTATGTTGGGAGGTGCGTCGACTG
GGGGCCGGCGACTTGAGCTCGGTCGTCCCGTGAAGCCCATCAGTCCCATGGAGACGTT
CTCTCCCATCCATTGGCCTCCGGGGCTCTCCACCAATCGCACCGGAGTCTTGTCTAGTG
TTAAATGTATTCATCGTGGGGTATAAACCGCCCCCGCGTAAGTGCGGCTCGAAGTCTG
GCCTCCCAGGCTGCTAGCAAACACGGAAGTGTGCCTCGTCTCGCTCAGAGGGGACAAG
ACACTCTCGACGGCTCTCAACAAGCCTAGTCCCCACGATACATACAACCGTGCCCGGT
GGGATTAATACGCCGGGGTCCCGTCGCGGGACATCTGGGATGGGAACACGTTCCGACC
ATATGCTCTCTGTCTGCATCTCTTGGGGAAATCCATTACGCTGCCGTTGACGTCGTTAA
CGTCACGCCACTAATACCGAACTAGGGGGGCCAAACTATACCATGTTATCCGTGCGCC
TACCCACTGTTGCGATTCAGGGTACGGGGCTGTGAGTAGTGTACACACTCTTGATGGG
AGCTTATCCTCCGGACTTGAATGGCTACTCGCCGGGCTGGGACTTAGCAAACCACCCTG
ACGCATTCCAGGCCTCTGATCAGATCACAGTTTTGCTTTCACCAGTTCCCAATTACGGT
TCCGCATCGTCGGGAGGCCTTCGACTAGACCTACTCGCATGGTTCGAGCGGTAATATCG
GGTCTGAAATCCCTTGACCCAACGGGAGGCGGTACGTGTGATCGGAATCTGCACCTTG
CGAGAGCTAATCGTAATGCCGATGGCTCTTCCACAAGTGAGTGCTGGGGCGCGAGTGG
CGGACCAGATTGTCTTCCGAATGTTCCCCCCCGTGTCGAAGGCTGAACTCGAGGCGGA
GCCGTGAGCGCGCACCGTTGCCACGCAGGTGCACCGCGAGGCACCTCGAAGGTCCCGT
CATGAAG

>VWP_ERR173209.1

GTCGGGATGCCCCCTCGACTCTGGCACGGCGCGGCGCCTTCGCGCACGGATCGGATAA
GCTTTCCCGTGGACGGCTCGTCCTCGGAAATATCTCGTAGTCTTCTCGTTGGTTGCCCC
GATAACGCGCCGCGTCCGCTTGGATACCGAACCGCGAGAGCTATTCGAGAGTTCTGTT
ACCCGACCGGGGGCAGCACACAACCGTGGGATCGGCGCGGCCCACCTCCGGCCAACG
AGAGTTACGAGTACCCGGCGCGTTTTCCCGCGCCGGAGGCTGCCAACACGATAGTAAC
TGAACTGGCCCAAGCGTATTGATACGCCCCAGGGGCGGGCCTCTGGCGCGCCACTGGA
TCAGGCCCGTGGCGTGCCCGCCTCGTCAGCGCCACCCATTGCTAAGCGCTGACAGTAA
TAGACCCCTCCATAGTAGTTGCCGATGTTGACCCAATCACCGGCCGAAACGTATGCACT
CAGCACAGAGCAGGTATCACAGAGCGAAAAGTGGATGATTGGCAGGGGCTGCTGGCG
CGCCTGCAAGCCTGTTCCCCCGCGCCTGCTGGAGCGACCAACTACGCCCCGCAGCGAC
CGGTACCCGAGCAGTCTCTCAACTGGTCCGATGAGACTGTGTACATCGTCGTCGGGAC
GCGGACTAAACAACCCCCCCATACCCATCCGCCCGTCCGGAGCGGAACGACTCGGCGG
TACCGGTGTCAGGCCCCCTCGCCTAGGCTGCACCATAATGTTGGGAGGTGCGTCAACT
GGGGGCCGGCGACCTGAGCTCGGCCGTCCCGTGAAGCCCATCAGTCCCATGGAGACGT
TCTCTCCCATCCATTGGCCTCCGGGGCTCTCCACCAATCGCACCGGAGTATTGCCTAAT
GTGAAATGTATTCAACGTGAGGTATAAACCGCCCCCGCGTGAGTGCGGCTCGAAATCC
GGCCCCTCGGGCTGCCAGCTAACACGGAAGTGTGCCTCGCTTCGCTCAGAGGGGACGA
GATACTCTCGACGGATCTCAACAAGCCTAGTCCCCACGAGGCATACAACCGTGCCCGG
TGGGATTAATACGCCGGGGTCCCGCTGCGAGACGTCTGAGAGGGAAATACGTTCCGGC
CATATACTCTCTGTCCGCGTACTTCGGAGAAATCCATTACGTTGCCGCCGACATCGTCC
CCACGACGCCACGGACGCTGAGCTAAGGGAACCGGACCTTGCCATGTAACCCCTGTAT
CTACCCACTGTTGCGGTTCAGGGAACGGGGGTGTGAGTAATGTACGCACTTCTGGTGG
GAACTTGGCCTCCGGACTTGAATGGCTAGTCGTCGGGCCGGAATTTAGCAAACCACCT
TGGCGCTCTCCAGGCCTCTGATTAGGTTACTATTTAACGTTGACCGGTTCCCAACCACG

GTTCCACATCGTCGGGAGGCCTTCAACTAGATCTACTCGCATGGTTCGAGCGGTAATAT
CGGGTCTGAAATCCCCTGATCCAACGGGGGGCGGCATGCGTGATCGGGATCTGCACCT
TGCGAGAGCTAATCATAATGACGACGACTCTTCCACAAGTAAGTGCTGGGGCGCGAGC
GGCGGACCAGATGGTCTTCCGAATGTTCCCCCCCGTGCCGAAGGCTGAACTCGAGGCG
GGGCCGTGGGCGCGCACCGTTGCCACGCAGGTGCACCGCGAGGCACCTTGGAGGTCCC
GTCATGAAG

>VWP_ERR173209.2

GTCGGGATGCCCCCTCGACTCTGGCACGGCGCGGCGCCTTCGCGCACGGATCGGATAA
GCTTTCCCTTGGACGGAGCGTCCTCAGCGACATCTCGTAGTCTTCTCGTTAGTTGCCCC
GATAACGCGCCGCGTCCGCTTGGATACCGAACCGCGAGAGCTATTCGAGAGTTCTGTT
ACCCGACCGGGGGCAGCACACAACCGTGGGATCGGCGCGGCCCACCTCCGGCCAACG
AGAGTTACGAGTACCCGGCGCGTTTTCCCGCGTCGGAGGCTGCCAACACGATAGTAAC
TGAACTGGCCCAAGCATATTGATACGCCCCAGGGGCGGGCCTCTGGCGCGCCACTGGA
TCAGGCCCGTGGCGTGCCCGCCTCGTCAGCGCCACCCATTGCTAAGCGCTGACAGTAA
TAGACCCCTCCATAGTAGTTGCCGATGTTGACCCGATCACCGGCCGGTGCGTATGCACT
CAGCACAGGGCAGGTACCACAGAGCGAAAAGTGGATGATTGGCAGGGGCTGCTGGCG
CGCCTGCAAGCCTGTTCCCCCGCGCCTGCTGGAGCGACCAACTACGCCCCGCAGCGAC
CGGTACCCGAGCAGTCTCTCAACTGGTCCGATGAGACTGTATACATCGTCGTCGGGAC
GCGGACTAAACAACCCCCCCATACCCATCCGCCCGTCCGGAGCGGAACGACTCGGCGG
TACCGGCGTCAGGCCCCCTCGCCTAGGCTGCACCATAATGTTGGGAGGTGCGTCGACT
GGGGGCCGGCGACCTGAGCTCGGCCGTCCCGTGAAGCCCATCGGTCCCATGGAGACGT
TCTCTCCCATCCATTGGCCTCCGGGGCTCTCCACCAATCGCACCGGAGTATTGCCTAAT
GTGAAATGTATTCATCGTGAGGTATAAACCGCCCCCGCGTGAGTGCGGCTCGAAATCC
GGCCCCTCGGGCTGCCAGCTAACACGGAAGTGTGCCTCGCTTCGCTCAGAGGGGACGA
GACACTCTCGACGGATCTCAACAAGCCTAGTCCCCACGAGGCATACAACCGTGGCCGG
TGGGATTAATACGCTGGGGTCCCGCTGCGAGACGTCTGAGAGGGAAATACGTTCCGGC
TATATACTCTCTGTCCGCGTACTTCGGAGAAATCCATTACGTTGCCGCCGACATCGTCC
CCACGACGCCACGGACGCTGAGCTAAGGGAACCGGACCTTGCCATGTAATCCCTGTAT
CTACCCACTGTTGCGGTTCAGGGAACGGGGGTGTGAGTAATGTACGCACTTCTGGTGG
GAACTTGGCCTCCGGACTTGAATGGCTAGTCGTCGGGCTGGAATTTAGCAAACCACCTT
GGCGCTCTCCACACCCCTTGCTAGGTTACTATTTAACGTTGACCGGTTCCCAACCACGG
TTCCGCATCGTCGGGAGGCCTTCGACTAGATCTACTCCCATGGTTCGAGCGGTAATATC
GGGTCTGAAATCCCCTGACCCAACGGGGGGCGGCATGTGTGATCGGGATCTGCACCTT
GCGAGAGCTAATCATAATGACGACGACTCTTCCACAAGCAAGTGCTGGGGCGCGAGCG
GCGGACCAGATGGTCTTCCGAATGTTCCCCCCCGTGCCGAAGGCTGAACTCGAGGCGG
GGCCGTGGGCGCGCACCGTTGCCACGCAGGTGCACCGCGAGGCACTTTGGAGGTCCCG
TCATGAAG

>CLWB_ERR173210.1

GTCGGAATGCCTCCTCGGCTCTGGCACGGCGCAGCGCCTTCGCGCACAGTTCGTATAA
GCTCTCCCTTGGACGGATCGTCCTCAGCGACATCTCGTAGTCTTCTCGTTAGTTGCCCC
GATAACGCGCCGCGTCCGCTTGGATACCGAACCGCGAGAGCTATACGAGTGTTCTGTT
ACCCGACCAGGGGCAGCACACAACCGTGGGATCGGCGCGGCCCACCTCCGGCCAACG
AGAGTTACGAGTACCCGGCGCGTTTTCCCGCGTCGGAGGCTGCCAACACGATAGTAAC
TGAACTGGCCCAAGCGTATTGATACGCCCCAGGGGCGGGCCTCTGGCGCGCCACTGGA
TCAGGCCCGTGGCGTGCCCGCCTCGTCAGCGCCACCCATTGCTAAGCGCTGACAGTAA
TAGACCCCTCCATAGTAGTTGCCGATGTTGACCCGATCACCGGCCGGTGCGTATGCACT
CAGCACAGGGCAGGCACCACAGAGCGAAAAGTGGATGATTGGCAGGGGCTGCTGGCG
CGCCTATCAACCTGTTCCCCTCGGCCTGCTGGAGCGATCAATTACACCCCGCAGCGACC

GGTACCTGAGCTGTTTCTCAACTGGTCCGATGAGACTGTGTTCATCGTCGTCGGGACGC
GGACTAAACAACCCCCCCATACCCATCCGCCCGTCCGGAGCGGAACGACTTGGCGGTA
CCGGCGTCAGGCCCCCTCGCCTAGGCTGCACCATAATGTTGTGCGGCGTGTCGACTGG
GGGCCGGCGTCCTGAGCTCAGCCGTCCCGTGAAGCCCATCAGTCCCATGGAGACGTTC
TCTCCCATCCATTGGCCTCCGGGGCTCTCCACCAATCGCGCTAGAACATTGCCTAATGA
GAAATGTATTCATCGTGGGGTACAAACCGCCCCCGCGTGGGTGCAACTCATAATTTGG
CCTCTCGGGCTGCCAGCTGATACGGAAGTGTGCCTCGCTTCGCTCAGAGGGGACGAGA
CACTCTCGACGGATCTCAACAAGCCTAGTCCCCACGAGGCATACAACCGTGGCCGGTG
GGATTAATACGTCGGGGTCCCGCTGCGGGATGTCTGGGAGGGGAACACGTTCCGGCCA
TATACTCTCTTTCCGCGTACTTCGGAGAAATCCATTACGTTGCCGCCAACATCGTCCCC
ACGACGCCACGGGCGCCGAGCTGGGGGGACCGAACCTTGCCATGTAATCCCTGTATCT
ACCCACTGTTGCGGTTCAGGGAACGGGGGTGTGAGTAATGTAGGCACTTCTGGTGGGA
ACTTGGCCTCCGGACTTGAATGGCTAGTCGTCGGGCCGGAATTTAGCAAACCACCTTG
GCGCTCTCCAGGCCCCTGATTAGGTTATTATTTAACGTTGACCGGTTCCCAACCACGGT
TCCACAGTTTCGGTAAGCCTTCAACTAGATCTATTCGCATGGTTCGAGCGGTACTGTCG
GGTCTGAAATCCCCTGACCCAACGGGGGGCGATGTGTGTGATCGGGATCTGCACCTTG
CGAGAGCTAATCGTAATGACGATGGCTCTTCCACAAGCAAGTTGTGGGGCGCGAGCGA
CGGACTAGATGGTCCTCCGAATGTCCCCCCCCGTACCGAAGGCTGAACTCGAGGCGGA
GCCGTGGGCGCGCACCGTTGCCACGCAGGTACACCGCGAGGCCCCGTGGAGGTCCCGT
CATGAAG

>CLWB_ERR173210.2

GTCGGAATGCCTCCTCGGCTCTGGCACGGCGCAGCGCCTTCGCGCACAGATCGTATAA
GCTCTCCCTTGGACGGATCGTCCTCAGCGACATCTCGTAGTCTTCTCGTTAGTTGCCCC
GATAACGCGCCGCATCCGCTTGGATACCGAACCGCGAGAGCTATTCGAGTGTTCTGTT
ACCCGACCGGGGGCAGCTCACAACCGTGGGATCGGCGCGGCCCACCTCCGGCCAACGA
GAGTTACGAGTACCCGGCGCGTTTTCCCGCGTCGGAGGCTGCCAACACGATAGTAACT
GAACTGGCCCAAGCGTATTGATACGCCCCAGGGGCGGGCCTCTGGCGCGCCACTGGAT
CAGGCCCGTGGCGTGCCCGCCTCGTCAGCGCCACCCATTGCTAAGCGCTGACAGTAAT
AGACCCCTCCATAGTAGTTGCCGATGTTGACCCAATCACCGGCCGAAACGTATGCACT
CAGCACAGAGCAGGCATCACGGAGCGAAAAGTGGATGATTGGCAGGGGCTGCTGGCG
CGCCTATCAACCTGTTCCCCTCGGCCTGCTGGAGCGACCAACTACGCCCCGCAGCGACC
GGTACCTGGGCAGTCTCTCAACTGGTCCGATGAGACTGTATACATCGTCGTCGGGTCGC
GGACTAAACAACCCCCCCATACCCATCCGCCCGTCTGGAGCGGAACGACTTGGCGGTA
CCGGCGTCAGGCCCCCTCGCCTAGGCTGCACCATAATGTTGTGCGGCGTGTCGACTGG
GGGCCGGCGTCCTGAGCTCGACCGTCCCGTGAAGCCCATCAGTCCCATGGAGACGTTC
TCTCCCATCCATTGGCCTCCGGGGCTCTCCACCAATCGCGCTAGAACATTGCCTAATGA
GAAATGTATTCATCGTGGGGCACAAGCCGCCCCCGCATGGGTGCAACTCATAATTTGG
CCTCTCGGGCTGCCAGCTGTCACGGAAGTGTGCCTCGCTTCGCTCAGAGGGGACGAGA
CACTCTCGACGGATCTCAACAAGCCTAGTCCCCACGAGGCATACAACCGTGGCCGGTG
GGATTAATACGTCGGGGTCCCGTCGCGGGATGTCTGGGAGGGGAACACGTTCCGGCCA
TATACTCTCTTTCCGCGTACTTCGGAGAAATCCATTACGTTGCCGCCAACATCGTCCCC
ACGACGCCACGGACGCTGAGTTAAGGGAACCGGACCTTGCCATTTAGCCCCTGTATCT
ACCCACTGTTGCGGTTCAGGGAACGGGGGTGTGAGTAGTGTAGGCACTCCTGGTGGGA
ACTTGGCCTCCGGACTTGAATGGCTAGTCGTCGGGCTGGAATTTAGCAAACCACCTTGG
CGCTCTCCACACCTCTTGCTAGGTTATTATTTAACGTTGACCGGTTCCCAACCACGGTTC
CACAGTTTCGGTAAGCCCTCGACTAGATCTATTCCCATGGTTCGAGCGGTAAAACCGG
GTCTGGTGTCCCCTGATCCAACGGGGGGCGATGTGTGTGATCGGGATCTGCACCTTGCG
AGAGCTAATCGTAATGACGATGGCTCTTCCACAAGCAAGTTGTGGGGCGCGAGCGACG
GACTAGATGGTCCTCCGAATGTCCCCCCCCGTACCGAAGGCTGAACTCGAGGCGGAGC

CGTGGGCGCGCACCGTTGCCACGCAGGTACACCGCGAGGCCCCGTGGAGGTCCCGTCA
TGAAG

>JVWP_ERR173211.1

GTCGGGATGTCCCCTCGACTCTGGCACGGCGCAGCGCCTTCGCGCACGGATCGGATAA
GCTTTCCCTTGGACGGATCGTCCTCAGCGACATCTCGTAGTCTTCTCGTTGGTTGCCCCG
ATAACGCGCCGCGTCCGCTTGGATACCGAACCGCGAGAGCTATACGAGAGTTCTGTTA
CCCGACCGGGGGCAGCACACAACCGTGGGATCGGCGCGGCCCACCTCCGGCCAACGA
GAGTTACGAGTACCCGGCGCGTTTTCCCGCGCCGGAGGCTGCCAACACGATAGTAACT
GAACTGGCCCAAGCGTATTGATACGCCCCAGGGGCGGGCCTCTGGCGCGCCACTGGAT
CAGGCCCGTGGCGTGCCCGCCTCGTCAGCGCCACCCATTGCTAAGCGCTGACAGTAAT
AGACCCCTCCATAGTAGTTGCCGATGTTGACCCGATCACCGGCCGGTGCGTATGCACTC
AGCACAGGGCAGGTACCACAGAGCGAAAAGTGGATGATTGGCAGGGGCTGCTGACGC
GCTTGCAAGCCTGTTCCCCCGGGCCTGCTGGAGCGACCAATTACGCCCCGCAGCGACC
GGTACCTGAGCTGTTTCTCAACTGGTCCGATGAGACTGTGTTCATCGTCGTTGGGACGC
GGACTAAACAACCCCCTCATACCCATCCGCCCGTCCGGAGCGGAACGACTCGGCGGTA
CCGGCGTCAGGCCCCCTCGCCTAGGCTGCACCATAATGTTGGGAGGCGCGTCGACTGG
GGGCCGGCGTCCCGAGCTCAGCCGTCCCGTGAAGCCCATCAGTCCCATGGAGACGTTC
TCTCCCATCCATTGGCCTCCGGGGCTCTCCACCAATCGCACCGGAGTATTGCCTAATGA
GAAATGTATTCAACGTGGGGCACAAGCCGCCCCCGCATGAGTGCAGCTCGAAGTTCGG
CCTCCCGGGCTGCCAGCTAACACGGAAGTGTGCCTCGTTTCGCTCAGAGGGGACGAGA
CACACTCGACGGCTCTCAACAAGCCTAGTCCCTACGATACATACAACCATGCCCGGTG
GGATTAATACGTCGGGGTCCCGCTGCGAGACGTCTGGGATGGGAACACGTTCCGGCCA
TATACTCTCTGTCCGCGTACTTCGGAGAAATCCATTACGTTGCCGCCGACATCGTCCCC
ACGACGCCACGGGCGCTGAGCTAAGGGAACCGAACCTTGCCATTTAATCCGCGCGCCT
ACCCACTGTTGCGGTTCAGGGAACGGGGGTGTAAGTAATGTACGCACTTCTGGTAGGA
ACTTGGCCTCCGGACTTGAATGGCTAGTCGTCGGGCTGGAATTTAGCAAACCACCTTGG
CGCTCTCCAGGCCTCTGATTAGGTTACTATTTAACGTTGACCGGTTCCCAACCACGGTT
CCGCAGTTTCGGTAAGCCTTCAACTAGATCTATTCGCATGGTTCGAGCGGTAATATCGG
GTCTGAAATCCCCTGACCCAACGGGGGGCGATGTGTGTGATCGGGATCTGCACCTTTC
GAGAGCTAATCGTAATGACGACGACTCTTCCACAAGTAAGTGCTGGGGCGCGAGCGAC
GGACCAGATGGTCTTCCGAATGTTCCCCCCCGTGCCGAAGGCTGAACTCGAGGCGGAG
CCGTGGGCGCGCACCGTTGCCACGCAGGTGCACCGCGAGGCACTTTGGAGGTCCCGTC
ATGAAG

>JVWP_ERR173211.2

GTCGGGATGCCCCCTCGACTCTGGCACGGCGCGGCGCCTTCGCGCACGGTTCGTATAA
GCTTTCCCTTGGACGGAGCGTCCTCAGCGACATCTCGTAGTCTTCTCGTTAGTTGCCCC
GATAACGCGCCGCGTCCGCTTGGATACCGAACCGCGAGAGCTATACGAGTGTTCTGTT
ACCCGGCCAGGGGCAGCACACAACCGTGGGATCGGCGCGGCCCACCTCCGGCCAACG
AGAGTTACGAGTACCCGGCGCGTTTTCCCGCGTCGGAGGCTGCCAACACGATAGTAAC
TGAACTGGCCCAAGCGTATTGATACGCCCCAGGGGCGGGCCTCTGGCGCGCCACTGGA
TCAGGCCCGTGGCGTGCCCGCCTCGTCAGCGCCACCCATTGCTAAGCGCTGACAGTAA
TAGACCCCTCCATAGTAGTTGCCGATGTTGACCCGATCACCGGCCGAAACGTATGCACT
CAGCACAGGGCAGGTACCACAGAGCGAAAAGTGGATGATTGGCAGGGGCTGCTGACG
CGCTTGCAAGCCTGTTCCCCCGCGCCTGCTGGAGCGACCAATTACGCCCCGCAGCGAC
CGGTACCTGAGCTGTTTCTCAACTGGTCCGATGAGACTGTATACATCGTCGTTGGGACG
CGGACTAAACAACCCCCTCATACCCATCCGCCCGTCCGGAGCGGAACGACTCGGCGGT
ACCGGCGTCAGGCCCCCTCGCCTAGGCTGCACCATAATGTTAGGAGGCGCGTCGACTG
GGGGCCGGCGTCCCGAGCTCAGCCGTCCCGTGAAGCCCATCAGTCCCATGGAGACGTT

CTCTCCCATCCATTGGCCTCCGGGGCTCTCCACCAATCGCACCGGAGTATTGCCTAATG
AGAAATGTATTCATCGTGGGGCACAAGCCGCCCCCGCATGAGTGCAGCTCGAAGTTCG
GCCTCCCGGGCTGCCAGCTAACACGGAAGTGTGCCTCGTTTCGCTCAGAGGGGACGAG
ACACTCTCGACGGCTCTCAACAAGCCTAGTCCCTACGATACATACAACCATGCCCGGT
GGGATTAATACGTCGGGGTCCCGCTGCGAGACGTCTGGGATGGGAACACGTTCCGGCC
ATATACTCTCTGTCCGCGTACTTCGGAGAAATCCATTACGTTGCCGCCGACATCGTCCC
CACGACGCCACGGGCGCTGAGCCAAGGGAACCGGACCTTGCCATTTAATCCGCGCGCC
TACCCACTGTTGCGGTTCAGGGAACGGGGGTGTAAGTAATGTACGCACTTCTGGTAGG
AACTTGGCCTCCGGACTTGAATGGCTAGTCGTCGGGCCGGAATTTAGCAAACCACCTT
GGCGCTCTCCAGGCCTCTTGCTAGGTTACTATTTAACGTTGACCGGTTCCCAACCACGG
TTCCGCATCGTCGGGAGGCCTTCGACTAGATCTACTCGCATGGTTCGAGCGGTAATATC
GGGTCTGAAATCCCCCGACCCAACGGGGGGCGATGTGTGTGATCGGGATCTGCACCGT
GCGAGAGCTAATCGTAATGACGACGACTCTTCCACAAGCAAGTGCTGGGGCGCGAGCG
ACGGACCAGATGGTCTTCCGAATGTTCCCCCCCGTGCCGAAGGCTGAACTCGAGGCGG
AGCCGTGGGCGCGCACCGTTGCCACGCAGGTGCACCGCGAGGCACTTTGGAGGTCCCG
TCATGAAG

>JWB_ERR173212.1

GTCGGGATGCCTTCCCAACTCTGGCACGGCGCGGCGCCTTCGCGCACGGATCGGATAA
GCTTTCCCGTGGACCACTCGTCCTCAGAAATATCTCGTAGTCTTCTCGTTGGTTATTCCA
ATGAAGCGCCTCGTCTGTGGGGGCACTGAACCGCTAGAGCTATTCGAGTGCTCTACTA
CTCCACCGGGGGCAGCACGCAGCCGTGGGATCGGCACGGCCCACCTCCAGCCAACGAG
AGTTACGACTACCCGGCCCGTTTTCTCGCGTCGGCGGCTGCCAACACGATAGTAAGTG
AACTGGCCCAAACGTATTAATACGCACAGGGGGCGGGCCTCTGGCGCGCCACTGGATC
AGGCCCGTGGCGTGCCCGCCTCGTCAGCGCCACCCATTGCTAAGCGCTGACAGTAATA
GACCCCTCCATAGTAGTTGCCGATGTTGATTCAGACACCGGCCGAAACGTATGCACTTA
GCACAGGGCAGGTACTACAAGGCGAGAGGGGGATGATTGGCAGGGGCTGCTGACGCG
CCTATCAGCCCGTTCCCCCGCGCCTGCTGTGGCGACCAACTACGCCCCGCAGTGACCGG
TACCCGAGCAGTCTCTCAACTGGTCCGATGAGACTGTATACACCGCCGTTGGGACGCG
GACTAAACAACTCCCTCATACCCATCCGCCCGTCCGGAGCGGAACGACTCGGCGGTAC
CGGCGTCAGGCCCCCTCGCCTAGACTGCACCATTATGTTGGGAGGTGCGTCGACTGGG
GGCCGGCGACTTGAGCTCGGTCGTCCCGTGAAGCCCATCAGTCCCATGGAGACGTTCT
CTCCCATCCATTGGCCTCCTAGGCTTTCCACCAATTGCGTCAGGGTAATGCCAGATGTT
AGATGTATTCATCGTGGGGTACAAACCGCCCCCGCGTGAGTGCGGCTCGTAGTCTGGC
CTCCCAGGCTGCCTGCTAACACGGAAGTGTACCCGGTTCCACTCAGAGGGCATGAGGC
AGTACCTACGGATCTCAACAAGCCTAGTCCCCATGATACGTACAGCTGGGCCAGGTGG
GATTAATACGCCGGGGTCCCGTCGCGGGATGTCTAGGGGGGAGATACGTTCCGGCCAT
ATACTCTCTGTCCACATCCCTTGAGGAAATCCATTACGCTGCCGTTGACGTCGTTAACG
CCACGCCACTAACACCGAACTAGGGGGACCAAACTATACCATGTTATCCGTATGCCCA
CTTGTTGTTGCGGTTCAGGGTACGGGGCTGTGAGGCACGGGCGCGCTCCTGGTGGGAA
CTTGTCCTCTGAACTTGAATAACAGCTCACTGGGATGGGACTTAAAAAACCATCCCAA
CATATTCCAGGCCTCTGATCAGATCACAATTTTGCTTTCACCAGTTCCCAATTATGATTC
CGCATCGTCGGGAGGCCTTCGACTAGATCTGCTCGCATGGTTCGAGCGGTAATATCGG
GTTTGAAATCCCCTGACCCAACGGGAGGCGGTACGTGTGATCGGAATCTGCACCTTGC
GAGAGCTAATCGTAATGACGATGGACCTTCTACAAGTGAGTGCTGGGGCACGAGCGGC
GGACCAGATGGTCTTCTGAATGTTCCTTCCCGTGCCGAAGGCTGAACTCGAGGCGGAG
CAGTGGGCGCGTACCGTCGCCACGCAGGTGCACTACGAGGTCCCTTGAAGGTCCCGTC
ATGAAG

>JWB_ERR173212.2

GTCGGGATGCCTTCCCAACTCTGGCACGGCGCGGCGCCTTCGCGCACGGATCGGATAA
GCTTTCCCGTGGACCACTCGTCCTCAGAAATATCTCGTAGTCTTCTCGTTGGTTATTCCA
ATGAAGCGCCTCGTCTGTGGGGGCACTGAACCGCTAGAGCTATTCGAGTGCTCTACTA
CTCCACCGGGGGCAGCACGCAGCCGTGGAATCGGCACGGCCCACCTCCAGCCAACGAG
AGTTACGACTACCCGGCCCGTTTTCTCGCGTCGGAGGCTGCCAACACGATAGTAACTG
AACTGGCCCAAACGTATTAATACGCCCCGGGGGCGGGCCTCTGGCGCGCCACTGGATC
AGGCCCGTGGCGTGCCCGCCTCGTCAGCGCCACCCATTGCTAAGCGCTGACAGTAATA
GACCCCTCCATAGTAGTTGCCGATGTTGATTCGGTCACCGGCCGAAACGTATGCACTTA
ACACAGGGCAGGTACTACAAAGCGAGAGGGGGATGATTGGCAGGGGCTGCTGACGCG
CCTATCAGCCCGTTCCCCCGCGCCTGCTGTGGCGACCAACTACGCCCCGCAGTGACCGG
TACCCGAGCAGTCTCTCAACTGGTCCGATTAGACTGTATACACCGCCGTTGGGACGCG
GACTAAACAACTCCCTCATACCCATCCGCCCGTCCGGAGCGGAACGACTCGGCGGTAC
CGGCGTCAGGCCCCCTCGCCTAGACTGCACCATTATGTTGGGAGGTGCGTCGACTGGG
GGCCGGCGACTTGAGCTCGGTCGTCCCGTGAAGCCCATCAGTCCCATGGAGACGTTCT
CTCCCATCCATTGGCCTCCGGGGCTCTCCACCAATTGCGTCAGGGTAATGCCAGATGTT
AGATGTATTCATCGTGGGGTACAAACCGCCCCCGCGTGAGTGCGGCTCGTAGTCTGGC
CTCCCAGGCTGCCTGCTAACACGGAAGTGTACCCGGTTCCACTCAGAGGGCATGAGGC
AGTACCTACGGATCTCAACAAGCCTAGTCCCCATGATACGTACAGCTGGGCCAGGTGG
GATTAATACGCCGGGGTCCCGTCGCGGGATGTCTAGGGGGGAGATACGTTCCGGCCAT
ATACTCTCTGTCCACATCCCTTGAGGAAATCCATTACGCTGCCGTTGACGTCGTTAACG
CCGCTTCATTAACGTCGAACCAGGGAGACCAAACTATACCATGTTATCCGTATGCCCAC
TTGTTGTTGCGGTTCAGGGTACGGGGCTGTGAGGCACGGGCGCGCTCCTGGTGGGAAC
TTGTCCTCTGAACTTGAATAACAGCTCACTGGAATGGGATTTAGCAAACCATCCCAACA
TATTCCAGGCCTCTGATCAGATCACAATTTTGCTTTCACCAGTTCCCAATTATGATTCCG
CATCGTCGGGAGGCCTTCGACTAGATCTGCTCGCATGGTTCGAGCGGTAATATCGGGTC
TGAAATCCCCTGACCCAACGGGAGGCGGTATGCGCAGTCAGGGTCTGCACCTTGCGAG
AGCTAATCGTAATGACGATGGCTCTTCCACAAGTGCGTGCTGGGGCGCGAGCGACGGA
CCAGATGATCTTCCGAATGTTTCCCCCCGTGCCGAAGGCCGAACCCGAGGCGGAGCCG
TGGGCGTGCACCGTTGCCACGCAGGTGCACCGTGAGGCACCTTGAAGGTCCCGTCATG
AAG

>WWB_ERR173213.1

GTCGGGATGCTTTCCCAACTCCGGCACGGCGCGGCGCCTTCGCGCACGGATCGGATAA
GCTTACCCGTGGACGGCTCGTCCTCAGAAACATTTCGTAGTCTTCTCGTTGGTTACTCC
ACTGCCGCGCCGCGTCTGCTTGGATGTTGAACCGCGAGAGCTATTCAAGTGCTTTACTA
CTCGACCGGGGGCAGCTTACAACCGTGGGATCGGCGTGGCCCACCTCCGGCCAACGAG
AGTTACGAGTACCCGGCCCGTTTTCCCGCGTCGGAGGCTGCCAACACGATAGTAGCTG
AACTGGCCCAAACGTCTTGATACGCCCCGGGGGCGGGCCTCTGGCGCGCCACTGGATC
AGGCCCGTGGCGTGCCCGCCTCGTCAGCGCCACCCATTGCTAAGCGCTGACAGTAATA
GACCCCTCCATAGTAGTTGCCGATGTTGATTCGGTCACCGGCCGAAACGTGCGCGCTCA
GCACAGGGCAGGTACCACGGAGTAAAAGGTGGATGATTGGCAGGGGCTGCTGGCGAG
CCTACCAACCTGTTCCTCCGCGCCTGCTGGAGCGACCAACTACGCCCCGCAGCGACCG
GTACTTGAGCTGTCTCTCGACTGGTCCGATTAGATTGTATAAACAGTCGTTAAGACGCG
GACTAAACAGCCCCCTCATACCCACCCGCCCGTCCGGAGCGGAACGACTCGGCGGTAC
CGGCGTCAGGCCCCCTCGCCTAGGCTGCACCATTATGTTGGGAGGTGCGTCGACTGGG
GGCCGGCGACTTGAGCTCGGTCGTCCCGTGAAGCCCATCAGTCCCATGGAGACGTTCT
CTCCCATCCATTAGCCTCCGGGGCTCTCCACCACTCGCACCGGAGTCTTGTCTAGTGTT
AAATGTATTCATAGTGGGGTATAAACCGCCCCCGCGTGAGTGCGGCTCGAAGTCTGGC
CTCCCAGGCTGCTAGCTAACACGGAAGTGTGCCTCGTTTCGCTCAGAGGGGACGAGAC
ACTCTCGACGGCTCTCAACAAGCCTAGTCCCCACGATACATACAACCGTGCCCGGTGG
GATTAATACGCCGGGGTCCCGTCGCGGGACATCTGGGATGGGAACACGTTCCGGCCAT

ATGCTCTCTGTCCGCATCTCTTGGGGAAATCCATTACGCTGCCGTTGACGTCGTTAACG
TCACGCCACTAATACCGAACTAGGGGGACCAAACTATACCATGTTATCCGTGCGCCTA
CCCACTGTTGCGATTCAGGGTACGGGGCTGTGAGTAATGTACACACTCCTGGTGGGAG
CTTGTCCTCCGGACTTGAATGGCTACTCGCCGGGCTGGGACTTAGCAAACCACCCTGAC
GCATTCCAGGCCTCTGATCAGATCACAATTTTGCTTTCACCAGTTCCCAATTACGGTTC
CGCATCGTCGGGAGGCCTTCGACTAGATCTACTCGCATGGTTCGGTCGGTAATATCGGG
TCTGAAATCCCTTGACCCAACGGGAGGCGGTACGTGTGATCGGAATCTGCACCTTGCG
AGAGCTAATCGTAATGCCGATGGCTCTTCCACAAGTGAGTGCTGGGGCGCGAGTGGCG
GACCAGATTGTCTTCCGAATGTTCCCCCCCGTGTCGAAGGCTGAACTCGAGGCGGAGC
CGTGGGCGCGCACCGTTGCCACGCAGGTGCACCGCGAGGCACCTCGAAGGTCCCGTCA
TGAAG

>WWB_ERR173213.2

GTCGGGATGCCTTCCCAACTCTGGCACGGCGCGGCGCCTTCGCGCACGGATCGGATAA
GCTTTCCCGTGGACGGCTCGTCCTTGGAAATATCTCGTAGTCTTCTCGTTGGTTGCTCCA
ACGAAGCGACGAATCTACTTGGATACTGAACCGCGAGAGCTATTCAAGTGCTCTACTA
CTCGACCGGGGGCGGCATACAGCCGTGGGATCGGCGCGGCCCACCTCCGGCCAACGAG
AGTTACGAGTACCCGACGCGTTTTTCCGCGTTGGAGGCTGCCAACCCGATAACAACTG
AACTGGCCCAAACGTATTAATACGCCCCGGGGGCGGGCCTCTGGCGCGCCACTGGATC
AGGCCCGTGGCGTGCCCGCCTCGTCAGCGCCACCCATTGCTAAGCGCTGACAGTAATA
GACCCCTCCATAGTAGTTGCCGATGTTAATTCGGTCACCGGCCGAAACGTATGCACTTA
GCATAGGGCAGGTACTACAAAGCGAGAGGTGGACGATTGGCAAAGGCTGCTGGCGAG
TCTACCAACCTGTTCCTCCGCGCCTGCTGGAGCGACCAACTACGCCCCGCAGCGACCG
GTACTTGGGCTGTCTCTCGACTGGTCCGATTAGATTGTATAAACAGTCGTTAAGACGCG
GACTAAACAACCTCCTCATACCAGTCCGCCCGTCCGGAGCGGAACGACTCGGCGGTAC
CGGCGTCAGGCCCCCTCGCCTAGGCTACACCATTATGTTGGGAGGTGCGTCGACTGGG
GGCCGGCGACTTGAGCTCGGTCGTCCCGTGAAGCCCATCAGTCCCATGGAGACGTTCT
CTCCCATCCATTGGCCTCCGGGGCTCTCCACCAATCGCACCGGAGTCTTGTCTAGTGTT
AAATGTATTCATCGTGGGGTATAAACCGCCCCCGCGTGGGTGCGGCTCGAAGTCTGGC
CTCCCAGGCTGCTAGCTAACACGGAAGTGTGCCTCGTTTCGCTCAGAGGGGACGAGAC
ACTCTCGACGGCTCTCAACAAGCCTAGTCCCCACGATACATACAACCGTGCCCGGTGG
GATTAATACACCGGGGTCCCGTCGCGGGACGTCTGGGATGGGACCATGTTCCGGCCGT
ATACTCCCTGTTCGTGCCCCTCGGAGAAATCCATTACGCTGCCGTTGACGTCGTTAACG
TCACGCCACTAACACCGAACTAGGGGGACCAAACTATACCATGTAATCCGTGCGCCTA
CCCACTGTTGCGATTCAGGGTACGGGGCTGTGAGTAGTGTACACACTCTTGGTGGGAG
CTTATCCTCCGGACTTGAATGGCTACTCGCCGGGCTGGGACTTAGCAAACCACCCTGAC
GCATTCCAGGCCTCTGATCAGATCACAATTTTGCTTTCACCAGTTCCCAATTACGGTTC
CGCATCGTCGGGAGGCCTTCGACTAGACCTACTCGCATGGTCCGAGCGGTAATATCGG
GTCTGAAATCCCCTGACCCAACGGGAGGCGGTACGTGTGATCGGAATCTGCACCTTGC
GAGAGCTAATCGTAATGCCGATGGCTCTTCCACAAGTGAGTGCTGGGGCGCGAGTGGC
GGACCAGATTGTCTTCCGAATGTTCCCCCCCGTGCCGAAGGCTGAACTCGAGGCGGAG
CCGTGGGCGCGCACCGTTGCCACGCAGCTGCACCGCGAGGCACCTCGAAGGTCCCGCC
ATGAAG

>WWB_ERR173214.1

GTCGGGATGCTTTCCCAACTCCGGCACGGCGCGGCGCCTTCGCGCACGGATCGGATAA
GCTTACCCGTGGACGGCTCGTCCTCAGAAACATTTCGTAGTCTTCTCGTTGGTTACTCC
ACTGCCGCGCCGCGTCTGCTTGGATGTTGAACCGCGAGAGCTATTCAAGTGCTTTACTA
CTCGACCGGGGGCAGCTTACAACCGTGGGATCGGCGTGGCCCACCTCCGGCCAACGAG
AGTTACGAGTACCCGGCCCGTTTTCCCGCGTCGGAGGCTGCCAACACGATAGTAGCTG

AACTGGCCCAAACGTATTGATACGCCCCGGGGGCGGGCCTCTGGCGCGCCACTGGATC
AGGCCCGTGGCGTGCCCGCCTCGTCAGCGCCACCCATTGCTAAGCGCTGACAGTAATA
GACCCCTCCATAGTAGTTGCCGATGTTGATTCGGTCACCGGCCGAAACGTGCGCGCTCA
GCACAGGGCAAGTACCACGGAGTGAAAGGTGGATGATTGGCAGGGGCTGCTGGCGAG
CCTACCAACCTGTTCCTCCGCGCCTGCTGGAGCGACCAACTACGCCCCGCAGCGACCG
GTACTTAAGCTGTCTCTCGACTGGTCCGATTAGATTGTATAAACAGTCGTTAAGACGCG
GACTAAACAACCTCCTCATACCAGTCCGCCCGTCCGGAGCGGAACGACTCGGCGGTAC
CGGCGTCAGGCCCCCTCGCCTAGGCTGCACCATTATGTTGGGAGGTGCGTCGACTGGG
GGCCGGCGACTTGAGCTCGGTCGTCCCGTGAAGCCCATCAGTCCCATGGAGACGTTTTC
TCCCATCCATTGGCCTCCGGGGCTCTCCACCAATCGCACCGGAGTCTTGTCTAGTGTTA
AATGTATTCATCGTGGGGTATAAACCGCCCCCGCGTAAGTGCGGCTCGAAGTCTGGCC
TCCCAGGCTGCTAGCTAACACGGAAGTGTGCCTCGTTTCGCTCAGAGGGGACGAGACA
CTCTCGACGGCTCTCAACAAGCCTAGTCCCCACGATACATACAACCGTGCCCGGTGGG
ATTAATACGCCGGGGTCCCGTCGCGGGACATCTGGGATGGGAACACGTTCCGGCCATA
TGCTCTCTGTCCGCATCTCTTGGGGAAATCCATTACGCTGCCGTTGACGTCGTTAACGT
CACGCCACTAACACCGAACTAGGGGGACCAAACTATACCATGTAATCCGTGCGCCTAC
CCACTGTTGCGATTCAGGGTACGGGGCTGTGAGTAATGTACACACTCCTGGTGGGAGC
TTGTCCTTCGGACTTGAATGGCTACTCGCCGGGCTGGGACTTAGCAAACCACCCTGACG
CATTCCAGGCCTCTGATCAGATCACAATTTTGCTTTCACCAGTTCCCAATTACGGTTCC
GCATCGTCGGGAGGCCTTCGACTAGATCTACTCGCATGGTTCGAGCGGTAATATCGGG
TCTGAAATCCCCTGACCCAACGGGAGGCGGTACGTGTGATCAGGATCTGCACCTTGCG
AGAGCCAAGCGTAATGACGGTGGCTCTTCCACATGTAAAAGCTCGGGCGCGAGCGGCG
GGCTGGATGGTCCCCCGAATGTTCCCCCCTATGCCGAAGGCTGAACTCGAGGCTGAGC
CGTGGGCGCGCACCGTTGCCACGCAGGTGCACCGCGAGGTCCCTTGAAGGTCCCGTCA
CGAAG

>WWB_ERR173214.2

GTCGGGATGCTTTCCCAACTCCGGCACGGCGCGGCGCCTTCGCGCACGGATCGGATAA
GCTTACCCGTGGACGGCTCGTCCTCAGAAACATTTCGTAGTCTTCTCGTTGGTTACTCC
ACTGCCGCGCCGCGTCTGCTTGGATGTTGAACCGCGAGAGCTATTCAAGTGCTTTACTA
CTCGACCGGGGGCAGCTTACAACCGTGGGATCGGCGTGGCCCACCTCCGGCCAACGAG
AGTTACGAGTACCCGGCCCGTTTTCCCGCGTCGGAGGCTGCCAACACGATAGTAGCTG
AACTGGCCCAAACGTATTGATACGCCCCGGGGGCGGGCCTCTGGCGCGCCACTGGATC
AGGCCCGTGGCGTGCCCGCCTCGTCAGCGCCACCCATTGCTAAGCGCTGACAGTAATA
GGCCCCTCCATAGTAGTTGCCGATGTTGATTCGGTCACCGGCCGAAACGTGCGCGCTCA
GCACAGGGCAGGTACCACGGAGTGAAAGGTGGATGATTGGCAGGGGCTGCTGGCGAG
CCTACCAACCTGTTCCTCCGCGCCTGCTGGAGCGACCAACTACGCCCCGCAGCGACCG
GTACTTGAGCTGTCTCTCGACTGGTCCGATTAGATTGTATAAACAGTCGTTAAGACGCG
GACTAAACAACCTCCTCATACCAGTCCGCCCGTCCGGAGCGGAACGACTCGGCGGTAC
CGGCGTCAGGCCCCCTCGCCTAGGCTGCACCATTATGTTGGGAGGTGCGTCGACTGGG
GGCCGGCGACTTGAGCTCGGTCGTCCCGTGAAGCCCATCAGTCCCATGGAGACGTTTTC
TCCCATCCATTGGCCTCCGGGGCTCTCCACCAATCGCACCGGAGTCTTGTCTAGTGTTA
AATGTATTCATCGTGGGGTATAAACCGCCCCCGCGTAAGTGCGGCTCGAAGTCTGGCC
TCCCAGGCTGCTAGCTAACACGGAAGTGTGCCTCGTTTCGCTCAGAGGGGACGAGACA
CTCTCGACGGCTCTCAACAAGCCTAGTCCCCACGATACATACAACCGTGCCCGGTGGG
ATTAATACGCCGGGGTCCCGTCGCGGGACATCTGGGATGGGAACACGTTCCGGCCATA
TGCTCTCTGTCCGCATCTCTTGGGGAAATCCATTACGCTGCCGTTGACGTCGTTAACGT
CACGCCACTAACACCGAACTAGGGGGACCAAACTATACCATGTAATCCGTGCGCCTAC
CCACTGTTGCGATTCAGGGTACGGGGCTGTGAGTAATGTACACACTCCTGGTGGGAGC
TTGTCCTTCGGACTTGAATGGCTACTCGCCGGGCTGGGACTTAGCAAACCACCCTGACG
CATTCCAGGCCTCTGATCAGATCACAATTTTGCTTTCACCAGTTCCCAATTACGGTTCC

GCATCGTCGGGAGGCCTTCGACTAGATCTACTCGCATGGTTCGAGCGGTAATATCGGG
TCTGAAATCCCCTGACCCAACGGGAGGCGGTACGTGTGATCAGGATCCGCACCTTGCG
AGAGCCAAGCGTAATGACGGTGGCTCTTCCACAAGTAAAAGCTCGGGCGCGAGCGGC
GGGCTGGATGGTCCCCGAATGTTCCCCCTATGCCGAAGGCTGAACTCGAGGCTGAG
CCGTGGGCGCGCACCGTTGCCACGCAGGTGCACCGCGAGGTCCCTTGAAGGTCCCGTC
ACGAAG

>WWB_ERR173215.1

GTCGGGATGCCTTCCCAACTCTGGCACGGCGCGGCGCCTTCGCGCACGGATCGGATAA
GCTTTCCCGTGGACGGCTCGTCCTCGGAAATATCTCGTAGTCTTCTCGTTGGTTGCTCCA
ACGAAGCGACGAATCTACTTGGATACTGAACCGCGAGAGCTATTCAAGTGCTCTACTA
CTCGACCGGGGGCAGCATACAGCCGTGGGATCGGCGCGGCCCACCTCCGGCCAACGAG
AGTTACGAGTACCCGGCCCGTTTTCCCGCGTCGGAGGCTGCCAACACGATAGTAACTG
AACTGGCCCAAACGTATTGATACGCCCCGGGGGCGGGCCTCTGGCGCGCCACTGGATC
AGGCCCGTGGCGTGCCCGCCTCGTCAGCGCCACCCATTGCTAAGCGCTGACAGTAATA
GACCCCTCCATAGTAGTTGCCGATGTTAATTCGGTCACCGGCCGAAACGTATGAACGC
AGCACAGGGCAGGTACTACGGAGCGAAAGGTAGCTGATTGGCAGGGACTGCTGGCGC
GCCTACCAACCTGTTCCTCCGCGCCTGCTGGAGCGACCAACTACGCCCCGCAGCGACC
GGTACCCGAGCAGTCTCTCAACTGGTTCGATGAGACTGTATACACCGTCGTTGGGACG
CGGACTAAACAACCTCCTCATACCAATCCGCCCGTCCGGAGCGGAACGACTCGGCGGT
ACCGGCGTCAGGCCCCCTCGCCTAGGCTGCACCATTATGTTGGGAGGTGCGTCGACTG
GGGGCCGGCGACTTGAGCTCGGTCGTCCCGTGAAGCCCATCAGTCCCATGGAGACGTT
CTCTCCCATCCATTGGCCTCCGGGGCTCTCCACCAATCGCACCGGAGTCTTGTCTAGTG
TTAAATGTATTCATCGTGGGGTATAAACCGCCCCCGCGTGAGTGCGGCTCGAAGTCTG
GCCTCCCAGGCTGCTAGCTAACACGGAAGTGTGCCTCGTTTCGCTCAGAGGGGACGAG
ACACTCTCGACGGCTCTCAACAAGCCTAGTCCCCACGATACATACAACCGTGCCCGGT
GGGATTAATACGCCGGGGTCCTGTCGCGGGACGTCTGGGATGGGACCATGTTCCGGCC
GTATACTCCCTGTCCGCATCCCTCGGAGAAATTCATTACGCTGCCGTTGACGTCGTTAA
CGTCACGCCACTAACACCGAACTAGGGGGACCAAACTATACCATGTAATCCGTGCGCC
TACCCACTGTTGCGATTCAGGGTACGGGGCTGTGAGTAATGTACACACTCCTGGTGGG
AGCTTGTCCTTCGGACTTGAATGGCTACTCGCCGGGCTGGGACTTAGCAAACCACCCTG
ACGCATTCCAGGCCTCTGATCAGATCACAATTTTGCTTTCACCAGTTCCCAATTACGGT
TCCGCATCGTCGGGAGGCCTTCGACTAGATCTACTCGCATGGTTCGAGCGGTAATATCG
GGTCTGAAATCCCCTGACCCAACGGGAGGCGGTACGTGTGATCGGAATCTGCACCTTG
CGAGAGCTAATCGTAATGACGATTGCTCTTCCACAAGTGAGTGCTGGGGCGCGAGCGG
CGGACCAGATTGTCTTCCGAATGTTCCCCCCCGTGCCGAAGGCTGAACTCGAGGCGGA
GCCGTGGGCGCGCACCGTTGCCACGCAGGTGCACCGCGAGGCACCTCGAAGGTCCCGT
CATGAAG

>WWB_ERR173215.2

GTCGGGATGCCTTCCCAACTCTGGCACGGCGCGGCGCCTTCGCGCACGGATCGGATAA
GCTTTCCCGTGGACGGCTCGTCCTCGGAAATATCTCGTAGTCTTCTCGTTGGTTGCTCCA
ACGAAGCGACGAATCTACTTGGATACTGAACCGCGAGAGCTATTCAAGTGCTCTACTA
CTCGACCGGGGGCAGCATACAGCCGTGGGATCGGCGCGGCCCACCTCCGGCCAACGAG
AGTTACGAGTACCCGGCCCGTTTTTCCGCGTTGGAGGCTGCCAACCCGATAACAACTG
AACTGGCCCAAACGTATTGATACGCCCCGGGGGCGGGCCTCTGGCGCGCCACTGGATC
AGGCCCGTGGCGTGCCCGCCTCGTCAGCGCCACCCATTGCTAAGCGCTGACAGTAATA
GACCCCTCCATAGTAGTTGCCGATGTTAATTCGGTCACCGGCCGAAACGTATGAACGC
AGCACAGGGCAGGTACTACGGAGCGAAAGGTAGCTGATTGGCAGGGGCTGCTGGCGC
GCCTACCAACCTGTTCCTCCGCGCCTGCTGGAGCGACCAACTACGCCCCGCAGCGACC

GGTACCCGAGCAGTCTCTCAACTGGTTCGATGAGACTGTATACACAGTCGTTGGGACG
CGGACTAAACAACCTCCTCATACCAATCCGCCCGTCCGGAGCGGAACGACTCGGCGGT
ACCGGCGTCAGGCCCCCTCGCCTAGGCTGCACCATTATGTTGGGAGGTGCGTCGACTG
GGGGCCGGCGACTTGAGCTCGGTCGTCCCGTGAAGCCCATCAGTCCCATGGAGACGTT
CTCTCCCATCCATTGGCCTCCGGGGCTCTCCACCAATCGCACCGGAGTCTTGTCTAGTG
TTAAATGTATTCATCGTGGGGTATAAACCGCCCCCGCGTGAGTGCGGCTCGAAGTCTG
GCCTCCCAGGCTGCTAGCTAACACGGAAGTGTGCCTCGTTTCGCTCAGAGGGGACGAG
ACACTCTCGACGGCTCTCAACAAGCCTAGTCCCCACGATACATACAACCGTGCCCGGT
GGGATTAATACGCCGGGGTCCCGTCGCGGGACATCTGGGATGGGAACACGTTCCGGCC
ATATGCTCTCTGTCCGCATCTCTTGGGGAAATCCATTACGCTGCCGTTGACGTCGTTAA
CGTCACGCCACTAATACCGAACTAGGGGGACCAAACTATACCATGTAATCCGTGCGCC
TACCCACTGTTGCGATTCAGGGTACGGGGCTGTGAGTAATGTACACACTCCTGGTGGG
AGCTTGTCCTTCGGACTTGAATGGCTACTCGCCGGGCTGGGACTTAGCAAACCACCCTG
ACGCATTCCAGGCCTCTGATCAGATCACAATTTTGCTTTCACCAGTTCCCAATTACGGT
TCCGCATCGTCGGGAGGCCTTCGACTAGATCTACTCGCATGGTTCGAGCGGTAATATCG
GGTCTGAAATCCCCTGACCCAACGGGAGGCGGTACGTGTGATCGGAATCTGCACCTTG
CGAGAGCTAATCGTAATGACGATTGCTCTTCCACAAGTGAGTGCTGGGGCGCGAGTGG
CGGACCAGATTGTCTTCCGAATGTTCCCCCCCGTGCCGAAGGCTGAACTCGAGGCGGA
GCCGTGGGCGCGCACCGTTGCCACGCAGCTGCACCGCGAGGCACCTCGAAGGTCCCGT
CATGAAG

>WWB_ERR173216.1

GTCGGGATGCCTTCCCAACTCTGGCACGGCGCGGCGCCTTCGCGCACGGATCGGATAA
GCTTTCCCGTGGACGGCTCGTCCTCGGAAATATCTCGTAGTCTTCTCGTTGGTTGCTCCA
ACGAAGCGACGAATCTACTTGGATACTGAACCGCGAGAGCTATTCAAGTGCTCTACTA
CTCGACCGGGGGCAGCATACAGCCGTGGGATCGGCGCGGCCCACCTCCGGCCAACGAG
AGTTACGAGTACCCGGCCCGTTTTCCCGCGTCGGAGGCTGCCAACACGATAGTAACTG
AACTGGCCCAAACGTATTGATACGCCCCGGGGGCGGGCCTCTGGCGCGCCACTGGATC
AGGCCCGTGGCGTGCCCGCCTCGTCAGCGCCACCCATTGCTAAGCGCTGACAGTAATA
GACCCCTCCATAGTAGTTGCCGATGTTGATTCGGTCACCGGCCGAAACGTGCGCGCTCA
GCACAGGGCAGGTACCACGGAGCAAAAGGTGGATGATTGGCAGGGACTGCTGGCGAG
CCTACCAACCTGTTCCTCCGCGCCTGCTGGAGCGACCAACTACGCCCCGCAGCGACCG
GTACTTGAGCTGTCTCTCAACTGGTCCGATTAGATTGTATAAACAGTCGTTAAGACGCG
GACTAAACAACCTCCTCATACCAGTCCGCCCGTCCGGAGCGGAACGACTCGGCGGTAC
CGGCGTCAGGCCCCCTCGCCTAGGCTGCACCATTATGTTGGGAGGTGCGTCGACTGGG
GGCCGGCGACTTGAGCTCGGTCGTCCCGTGAAGCCCATCAGTCCCATGGAGACGTTCT
CTCCCATCCATTGGCCTCCGGGGCTCTCCACCAATCGCACCGGAGTCTTGTCTAGTGTT
AAATGTATTCATCGTGGGGTATAAACCGCCCCCGCGTGAGTGCGGCTCGAAGTCTGGC
CTCCCAGGCTGCTAGCTAACACGGAAGTGTGCCTCGTTTCGCTCAGAGGGGACGAGAC
ACTCTCGACGGCTCTCAACAAGCCTAGTCCCCACGATACATACAACCGTGCCCGGTGG
GATTAATACGCCGGGGTCCCGTCGCGGGACATCTGGGATGGGAACACGTTCCGGCCAT
ATGCTCTCTGTCCGCATCTCTTGGGGAAATCCATTACGCTGCCGTTGACGTCGTTAACG
TCACGCCACTAATACCGAACTAGGGGGACCAAACTATACCATGTAATCCGTGCGCCTA
CCCACTGTTGCGATTCAGGGTACGGGGCTGTGAGTAATGTACACACTCCTGGTGGGAG
CTTGTCCTTCGGACTTGAATGGCTACTCGCCGGGCTGGGACTTAGCAAACCACCCTGAC
GCATTCCAGGCCTCTGATCAGATCACAATTTTGCTTTCACCAGTTCCCAATTACGGTTC
CGCATCGTCGGGAGGCCTTCGACTAGATCTACTCGCATGGTTCGAGCGGTAATATCGG
GTCTGAAATCCCCTGACCCAACGGGAGGCGGTACGTGTGATCGGAATCTGCACCTTGC
GAGAGCTAATCGTAATGACGATTGCTCTTCCACAAGTGAGTGCTGGGGCGCGAGTGGC
GGACCAGATTGTCTTCCGAATGTTCCCCCCCGTGCCGAAGGCTGAACTCGAGGCGGAG

CCGTGGGCGCGCACCGTTGCCACGCAGGTGCACCGCGAGGCACCTCGAAGGTCCCGTC
ATGAAG

>WWB_ERR173216.2

GTCGGGATGCTTTCCCAACTCCGGCACGGCGCGGCGCCTTCGCGCACGGATCGGATAA
GCTTACCCGTGGACGGCTCGTCCTCAGAAACATTTCGTAGTCTTCTCGTTGGTTACTCC
ACTGCCGCGCCGCGTCTGCTTGGATGTTGAACCGCGAGAGCTATTCAAGTGCTTTACTA
CTCGACCGGGGGCAGCTTACAACCGTGGGATCGGCGTGGCCCACCTCCGGCCAACGAG
AGTTACGAGTACCCGGCCCGTTTTCCCGCGTCGGAGGCTGCCAACACGATAGTAGCTG
AACTGGCCCAAACGTATTGATACGCCCCGGGGGCGGGCCTCTGGCGCGCCACTGGATC
AGGCCCGTGGCGTGCCCGCCTCGTCAGCGCCACCCATTGCTAAGCGCTGACAGTAATA
GACCCCTCCATAGTAGTTGCCGATGTTGATTCGGTCACCGGCCGAAACGTGCGCGCTCA
GCACAGGGCAAATACCACGGAGTGAAAGGTGGATGATTGGCAGGGGCTGCTGGCGAG
CCTACCAACCTGTTCCTCCGCGCCTGCTGGAGCGACCAACTACGCCCCGCAGCGACCG
GTACTTGAGCTGTCTCTCGACTGGTCCGATTAGATTGTATAAACAGTCGTTAAGACGCG
GACTAAACAGCCCCCTCATACCCACCCGCCCGTCCGGAGCGGAACGACTCGGCGGTAC
CGGCGTCAGGCCCCCTCGCCTAGGCTACACCATTATGTTGGGAGGTGCGTCGACTGGG
GGCCGGCGACTTGAGCTCGGTCGTCCCGTGAAGCCCATCAGTCCCATGGAGACGTTCT
CTCCCATCCATTAGCCTCCGGGGCTCTCCACCACTCGCACCGGAGTCTTGTCTAGTGTT
AAATGTATTCATAGTGGGGTATAAACCGCCCCCGCGTGGGTGCGGCTCGAAGTCTGGC
CTCCCAGGCTGCTAGCTAACACGGAAGTGTGCCTCGTTTCGCTCAGAGGGGACGAGAC
ACTCTCGACGGCTCTCAACAAGCCTAGTCCCCACGATACATACAACCGTGCCCGGTGG
GATTAATACACCGGGGTCCCGTCGCGGGACGTCTGGGATGGGACCATGTTCCGGCCGT
ATACTCCCTGTTCGTGCCCCTCGGAGAAATCCATTACGCTGCCGTTGACGTCGTTAACG
TCACGCCACTAACACCGAACTAGGGGGACCAAACTATACCATGTAATCCGTGCGCCTA
CCCACTGTTGCGATTCAGGGTACGGGGCTGTGAGTAGTGTACACACTCTTGGTGGGAG
CTTATCCTCCGGACTTGAATGGCTACTCGCCGGGCTGGGACTTAGCAAACCACCCTGAC
GCATTCCAGGCCTCTGATCAGATCACAATTTTGCTTTCACCAGTTCCCAATTACGGTTC
CGCATCGTCGGGAGGCCTTCGACTAGACCTACTCGCATGGTTCGAGCGGTAATATCGG
GTCTGAAATCCCCTGACCCAACGGGAGGCGGTACGTGTGATCGGAATCTGCACCTTGC
GAGAGCTAATCGTAATGACGATTGCTCTTCCACAAGTGAGTGCTGGGGCGCGAGTGGC
GGACCAGATTGTCTTCCGAATGTTCCCCCCCGTGCCGAAGGCTGAACTCGAGGCGGAG
CCGTGGGCGCGCACCGTTGCCACGCAGGTGCACCGCGAGGCACCTCGAAGGTCCCGTC
ATGAAG

>WWB_ERR173217.1

GTCGGGATGCCTTCCCAACTCTGGCACGGCGCGGCGCCTTCGCGCACGGATCGGATAA
GCTTTCCCGTGGACGGCTCGTCCTCGGAAATATCTCGTAGTCTTCTCGTTGGTTGCTCCA
ACGAAGCGACGAATCTACTTGGATACTGAACCGCGAGAGCTATTCAAGTGCTCTACTA
CTCGACCGGGGGCAGCATACAGCCGTGGGATCGGCGCGGCCCACCTCCGGCCAACGAG
AGGTACGAGTACCCGGCCCGTTTTCCCGCGTCGGAGGCTGCCAACACGATAGTAGCTG
AACTGGCCCAAACGTCTTGATACGCCCCGGGGGCGGGCCTCTGGCGCGCCACTGGATC
AGGCCCGTGGCGTGCCCGCCTCGTCAGCGCCACCCATTGCTAAGCGCTGACAGTAATA
GACCCCTCCATAGTAGTTGCCGATGTTAATTCGGTCACCGGCCGAAACGTATGCACTTA
GCATAGGGCAGGTACTACAAAGCGAGAGGTGGACGATTGGCAAAGGCTGCTGGCGAG
CCTACCAACCTGTTCCTCCGCGCCTGCTGGAGCGACCAACTACGCCCCGCAGCGACCG
GTACTTGAGCTGTCTCTCAACTGGTTCGATGAGACTGTATACACCGTCGTTGGGACGCG
GACTAAACCGCCCCCTCATACCCACCCGCCCGTCCGGAACGGAACGACTCGGCGGTAC
CGGCGTCAGGCCCCCTCGCCTAGGCTGCACCATTATGTTGGGAGGTGCGTCGACTGGG
GGCCGGCGACTTGAGCTCGGTCGTCCCGTGAAGCCCATCAGTCCCATGGAGACGTTCT

CTCCCATCCATTAGCCTCCGGGGCTCTCCACCACTCGCACCGGAGTCTTGTCTAGTGTT
AAATGTATTCATAGTGGGGTATAAACCGCCCCCGCGTGGGTGCGGCTCGAAGTCTGGC
CTCCCAGGCTGCTAGCTAACACGGAAGTGTGCCTCGTTTCGCTCAGAGGGGACGAGAC
ACTCTCGACGGCTCTCAACAAGCCTAGTCCCCACGATACATACAACCGTGCCCGGTGG
GATTAATACACCGGGGTCCCGTCGCGGGACGTCTGGGATGGGACCATGTTCCGGCCGT
ATACTCCCTGTTCGTGCCCCTCGGAGAAATCCATTACGCTGCCGTTGACGTCGTTAACG
TCACGCCACTAACACCGAACTAGGGGGACCAAACTATACCATGTAATCCGTGCGCCTA
CCCACTGTTGCGATTCAGGGTACGGGGCTGTGAGTAGTGTACACACTCTTGGTGGGAG
CTTATCCTCCGGACTTGAATGGCTACTCGCCGGGCTGGGACTTAGCAAACCACCCTGAC
GCATTCCAGGCCTCTGATCAGATCACAGTTTTGCTTTCACCAGTTCCCAATTACGGTTC
CGCATCGTCGGGAGGCCTTCGACTAGACCTACTCGCATGGTCCGAGCGGTAATATCGG
GTCTGAAATCCCTTGACCCAACGGGAGGCGGTACGTGTGATCGGAATCTGCACCTTGC
GAGAGCTAATCGTAATGCCGATGGCTCTTCCACAAGTGAGTGCTGGGGCGCGAGTGGC
GGACCAGATTGTCTTCCGAATGTTCCCCCCCGTGTCGAAGGCTGAACTCGAGGCGGAG
CCGTGAGCGCGCACCGTTGCCACGCAGGTGCACCGCGAGGCACCTCGAAGGTCCCGTC
ATGAAG

>WWB_ERR173217.2

GTCGGGATGCCTTCCCAACTCTGGCACGGCGCGGCGCCTTCGCGCACGGATCGGATAA
GCTTTCCCGTGGACGGCTCGTCCTCGGAAATATCTCGTAGTCTTCTCGTTGGTTGCTCCA
ACGAAGCGACGAATCTACTTGGATACTGAACCGCGAGAGCTATTCAAGTGCTCTACTA
CTCGACCGGGGGCAGCATACAGCCGTGGGATCGGCGCGGCCCACCTCCGGCCAACGAG
AGTTACGAGTACCCGGCCCGTTTTCCCGCGTCGGAGGCTGCCAACACGATAGTAACTG
AACTGGCCCAAACGTATTGATACGCCCCGGGGGCGGGCCTCTGGCGCGCCACTGGATC
AGGCCCGTGGCGTGCCCGCCTCGTCAGCGCCACCCATTGCTAAGCGCTGACAGTAATA
GACCCCTCCATAGTAGTTGCCGATGTTAATTCGGTCACCGGCCGAAACGTATGAACGC
AGCACAGGGCAGGTACTACGGAGCGAAAGGTAGCTGATTGGCAGGGGCTGCTGGCGC
GCCTACCAACCTGTTCCTCCGCGCCTGCTGGAGCGACCAACTACGCCCCGCAGCGACC
GGTACCCGAGCAGTCTCTCAACTGGTTCGATGAGACTGTATACACCGTCGTTAAGACG
CGGACTAAACAGCCCCCTCATACCCACCCGCCCGTCCGGAGCGGAACGACTCGGCGGT
ACCGGCGTCAGGCCCCCTCGCCTAGGCTACACCATTATGTTGGGAGGTGCGTCGACTG
GGGGCCGGCGACTTGAGCTCGGTCGTCCCGTGAAGCCCATCAGTCCCATGGAGACGTT
CTCTCTCATCCATTGGCCTCCGGGGCTCTCCACCAATCGCACCGGAGTCTTGTCTAGTG
TTAAATGTATTCATCGTGGGGTATAAACCGCCCCCGCGTGAGTGCGGCTCGAAGTCTG
GCCTCCCAGGCTGCTAGCTAACACGGAAGTGTGCCTCGTTTCGCTCAGAGGGGACGAG
ACACTCTCGACGGCTCTCAACAAGCCTAGTCCCCACGATACATACAACCGTGCCCGGT
GGGATTAATACGCCGGGGTCCCGTCGCGGGACATCTGGGATGGGAACACGTTCCGGCC
ATATGCTCTCTGTCCGCATCTCTTGGGGAAATCCATTACGCTGCCGTTGACGTCGTTAA
CGTCACGCCACTAATACCGAACTAGGGGGACCAAACTATACCATGTAATCCGTGCGCC
TACCCACTGTTGCGATTCAGGGTACGGGGCTGTGAGTAATGTACACACTCCTGGTGGG
AGCTTGTCCTCCGGACTTGAATGGCTACTCGCCGGGCTGGGACTTAGCAAACCACCCTG
ACGCATTCCAGGCCTCTGATCAGATCACAATTTTGCTTTCACCAGTTCCCAATTACGGT
TCCGCATCGTCGGGAGGCCTTCGACTAGATCTACTCGCATGGTTCGAGCGGTAATATCG
GGTCTGAAATCCCCTGACCCAACGGGAGGCGGTACGTGTGATCGGAATCTGCACCTTG
CGAGAGCTAATCGTAATGACGATGGCTCTTCCACAAGTGAGTGCTGGGGCGCGAGTGG
CGGACCAGATTGTCTTCCGAATGTTCCCCCCCGTGCCGAAGGCTGAACTCGAGGCGGA
GCCGTGGGCGCGCACCGTTGCCACGCAGCTGCACCGCGAGGCACCTCGAAGGTCCCGC
CATGAAG

>WWB_ERR173218.1

GTCGGGATGCTTTCCCAACTCCGGCACGGCGCGGCGCCTTCGCGCACGGATCGGATAA
GCTTACCCGTGGACGGCTCGTCCTCAGAAACATTTCGTAGTCTTCTCGTTGGTTACTCC
ACTGCCGCGCCGCGTCTGCTTGGATGTTGAACCGCGAGAGCTATTCAAGTGCTTTACTA
CTCGACCGGGGGCAGCTTACAACCGTGGGATCGGCGTGGCCCACCTCCGGCCAACGAG
AGTTACGAGTACCCGGCCCGTTTTCCCGCGTCGGAGGCTGCCAACACGATAGTAGCTG
AACTGGCCCAAACGTATTGATACGCCCCGGGGGCGGGCCTCTGGCGCGCCACTGGATC
AGGCCCGTGGCGTGCCCGCCTCGTCAGCGCCACCCATTGCTAAGCGCTGACAGTAATA
GACCCCTCCATAGTAGTTGCCGATGTTGATTCGGTCACCGGCCGAAACGTGCGCACTCA
GCACAGGGCAAGTACTACGGAGTAAAAGGTGGATGATTGGCAGGGACTGCTGGCGAG
CCTACCAACCTGTTCCTCCGCGCCTGCTGGAGCGACCAACTACGCCCCGCAGCGACCG
GTACTTGAGCTGTCTCTCGACTGGTCCGATTAGATTGTATAAACAGTCGTTAAGACGCG
GACTAAACAACCTCCTCATACCAGTCCGCCCGTCCGGAGCGGAACGACTCGGCGGTAC
CGGCGTCAGGCCCCCTCGCCTAGGCTACACCATTATGTTGGGAGGTGCGTCGACTGGG
GGCCGGCGACTTGAGCTCGGTCGTCCCGTGAAGCCCATCAGTCCCATGGAGACGTTCT
CTCCCATCCATTGGCCTCCGGGGCTCTCCACCAATCGCACCGGAGTCTTGTCTAGTGTT
AAATGTATTCATCGTGGGGTATAAACCGCCCCCGCGTAAGTGCGGCTCGAAGTCTGGC
CTCCCAGGCTGCTAGCAAACACGGAAGTGCGCCTCGTTTCGCTCAGAGGGGACAAGAC
ACTCTCGACGGCTCTCAACAAGCCTAGTCCCCACGATACATACAACCGTGCCCGGTGG
GATTAATACGCCGGGGTCCCGTCGCGGGACATCTGGGATGGGAACACGTTCCGGCCAT
ATGCTCTCTGTCTGCATCTCTTGGGGAAATCCATTACGCTGCCGTTGACGTCGTTAACG
TCACGCCACTAACACCGAACTAGGGGGACCAAACTATACCATGTAATCCGTGCGCCTA
CCCACTGTTGCGATTCAGGGTACGGGGCTGTGAGTAATGTACACACTCCTGGTGGGAG
CTTGTCCTCCGGACTTGAATGGCTACTCGCCGGGCTGGGACTTAGCAAACCACCCTGAC
GCATTCCAGGCCTCTGATCAGATCACAATTTTGCTTTCACCAGTTCCCAATTACGGTTC
CGCATCGTCGGGAGGCCTTCGACTAGATCTACTCGCATGGTTCGAGCGGTAATATCGG
GTCTGAAATCCCCTGACCCAACGGGAGGCGGTACGTGTGATCAGGATCTGCACCTTGC
GAGAGCCAAGCGTAATGACGGTGGCTCTTCCACAAGTGAAAGCTCGGGCGCGAGCGG
CGGGCTGGATGGTCCCCCGAATGTTCCCCCCTATGCCGAAGGCTGAACTCGAGGCTGA
GCCGTGGGCGCGCACCGTTGCCACGCAGGTGCACCGCGAGGTCCCTTGAAGGTCCCGT
CACGAAG

>WWB_ERR173218.2

GTCGGGATGCCTTCCCAACTCTGGCACGGCGCGGCGCCTTCGCGCACGGATCGGATAA
GCTTTCCCGTGGACGGCTCGTCCTCGGAAATATCTCGTAGTCTTCTCGTTGGTTGCTCCA
ACGAAGCGACGAATCTACTTGGATACTGAACCGCGAGAGCTATTCAAGTGCTCTACTA
CTCGACCGGGGGCAGCATACAGCCGTGGGATCGGCGCGGCCCACCTCCGGCCAACGAG
AGTTACGAGTACCCGGCCCGTTTTCCCGCGTCGGAGGCTGCCAACACGATAGTAACTG
AACTGGCCCAAACGTATTGATACGCCCCGGGGGCGGGCCTCTGGCGCGCCACTGGATC
AGGCCCGTGGCGTGCCCGCCTCGTCAGCGCCACCCATTGCTAAGCGCTGACAGTAATA
GACCCCTCCATAGTAGTTGCCGATGTTAATTCGGTCACCGGCCGAAACGTATGCACTTA
GCACAGGGCAGGTACTACAAAGCGAGAGGTGGACGATTGGCAAAGGCTGCTGGCGAG
CCTACCAACCTGTTCCTCCGCGCCTGCTGGAGCGACCAACTATGCCCCGCAGCGACTGG
TACTTAAGCTGTCTCTCGACTGGTCCGATTAGATTGTATAAACAGTCGTTAAGACGCGG
ACTAAACAACCTCCTCATACCAGTCCGCCCGTCCGGAACGGAACGACTCGGCGGTACC
GGCGTCAGGCCCCCTCGCCTAGGCTGCACCATTATGTTGGGAGGTGCGTCGACTGGGG
GCCGGCGACTTGAGCTCGGTCGTCCCGTGAAGCCCATCAGTCCCATGGAGACGTTCTCT
CTCATCCATTGGCCTCCGGGGCTCTCCACCAATCGCACCGGAGTCTTGTCTAGTGTTAA
ATGTATTCATCGTGGGGTATAAACCGCCCCCGCGTGAGTGCGGCTCGAAGTCTGGCCTC
CCAGGCTGCTAGCTAACACGGAAGTGTGCCTCGTTTCGCTCAGAGGGGACGAGACACT
CTCGACGGCTCTCAACAAGCCTAGTCCCCACGATACATACAACCGTGCCCGGTGGGAT
TAATACGCCGGGGTCCCGTCGCGGGACATCTGGGATGGGAACACGTTCCGGCCATATG

CTCTCTGTCCGCATCTCTTGGGGAAATCCATTACGCTGCCGTTGACGTCGTTAACGTCA
CGCCACTAATACCGAACTAGGGGGACCAAACTATACCATGTAATCCGTGCGCCTACCC
ACTGTTGCGATTCAGGGTACGGGGCTGTGAGTAATGTACACACTCCTGGTGGGAGCTT
GTCCTCCGGACTTGAATGGCTACTCGCCGGGCTGGGACTTAGCAAACCACCCTGACGC
ATTCCAGGCCTCTGATCAGATCACAATTTTGCTTTCACCAGTTCCCAATTACGGTTCCG
CATCGTCGGGAGGCCTTCGACTAGATCTACTCGCATGGTTCGAGCGGTAATATCGGGTC
TGAAATCCCCTGACCCAACGGGAGGCGGTACGTGTGATCGGAATCTGCACCTTGCGAG
AGCTAATCGTAATGACGATGGCTCTTCCACAAGTGAGTGCTGGGGCGCGAGTGGCGGA
CCAGATTGTCTTCCGAATGTTCCCCCCCGTGCCGAAGGCTGAACTCGAGGCGGAGCCG
TGGGCGCGCACCGTTGCCACGCAGCTGCACCGCGAGGCACCTCGAAGGTCCCGCCATG
AAG

>SCWB_ERR173219.1

GTCGGGATGCCCTCCCAACTCTGGCACGGCGCGGCGCCTTCGTGCACGGATCGGATAA
GCTTTCCCGTAGATGGCTCGTTCTCAGAAATATCTCGTAGTCTTCTCGTTGGTTACTCCA
ATGAAGCGCCTCGTCTGCGGGGGCACTGAACCGCGAGAGCTATTCGAGTGCTCTACTA
CTCGACCGGGGGCAGCATACAGCCGTGGGATCGGTGCGGCCCTCTTTCGGCTAGTAGG
GGGTACGACGTCCCGGCCCGTTTTCCCGCGTCGGAGGCTGCCAACACGGCAGTAACTG
AACTGGCCCAAACGCATTGATACGCCCCGGGGGCGGGCCTCTGGCGCGCCACTGGATC
AGGCCCGTGGCGTGTCCGCCTCGTCAGCGCCACCCATTGCTAAGCGCTGACAGTAATA
GACCCCTCCATAGTAGTTGCCGATGTTGATTTGGTCACCGGCCGAAACGTATGCGCTCA
GCACAGGGCAGGTACTACGGAGCGAAAGGGGGATGATTGGCAGGGGCCGCTGGCACA
CCTACAAAACTATTCGTCCGCGCCTGCTGGAGCGACCAACTACGCTCTATAGCGTCCAG
TACCCGAGCAGTCCCTCAACTGGTCCGATTAGACTGTATACACCGCCGTTGGGACACG
GACTAAACAACTCCCTCATACCCATCCGCCCGTCCGGAGCGGAACGACTCGGCGGTAC
CGGCGTCAGGGCCCCTCGCCTAGGCTGCACCATTATGTTGGGAGGTGCGTCGATTGGG
GGCCGGCGACTTGAGCTCGGTCGTCCCGTGAAGCCCATCAGTCCCATGGAGACGTTCT
CTCCCATCCATTGGCCTCCGGGGCTCTCCACCAATCGCGCCGGAGTCTTGTCTAGTGTG
AAATTTATTCATCGTAAAGTACACACCGCCTTCGCGTGAGTGCGGCTCGAAGTCTGGCC
CCCCAGGCTGGTAGCTAACACGGAAGTGTGCCTCGTTTCGCTCAGAGGGGACGAGACA
CTCTCGACGGCTCTCAACAAGCCTAGTCCCCACGATACATACTACCGTGCCCGGTGGA
ATTAATACGCCGGGGTCCCGTCGCGGGACATCTGGGATGGGAACACGTTCCGGCCATA
TGCCCTTTGTCCGCATCTCTTGGGGAAATCCATTACGCTGCCGTTGACGTCGTTAACGT
CACGCCACTAATACCGAACTAGGGGGACCAAACTATACCATGTAATCCGTGCGCCTAC
TCACTGTTGCGATTCAGGGTACGGGGCTGTGAGTAATGTACACACTCCTGGTGGGAGC
TTGTCCTTCGGACTTGAATGGCTACTCGCCGAGCTGGGACTTAGCAAACCACCCTGACG
CATTCCAGGCCTCTGATTAGAACACAATTTTACTTTCACCGGGTCCCAATTACGGTTCC
GCATCGTCGGGAGGCCTTCGACTAGGTCTACTCGCATAGTTCGAGCGGTAATATTGGGT
CTGAAATCTCCCGACCCAACGGGGGGCAGTATGTGTGGTCGGAATCTGCACCTTGTGG
GAGCTAATCGTAATGACGATGGACCTTCTACAAGTGAGTGCTGGGGCACGAGCGGCTG
ACCAGATTGTCTTCCGAATGTTCCCCCCCGTGCCGAAGGCTGAACCCGAGGCGGAGCC
GTGGGCGTGCACCGTTGCCACGCAGGTGCACCGCGAGGCAACTTGAAGGTCCCGTCAT
GAAG

>SCWB_ERR173219.2

GTCGGGATGCCTTCCCAACTCCGGCACGGCGCGGCGCCTTCGCGCACGGATCGGATAA
GCTTACCCGTGGACGGCTCGTCCCCGGAGATATCTCGTATTCTTCTTGTTGGTTACTCCA
ATGAAGCGCCTCGTCTGCGGGGGCACTGAACCGCGAGAGCTATTCAAGTGCTCTACTA
CTCGACCGGGGGCAGCTTACAACCGCGGGAACGGCGCGGCCTACCTCCGGCCAACGAG
AGTTACGAGTACCCGGCCCGTTTTCCCGCGTCGGAGGCTGCCAACACGATAGTAACTG

AACTGGCCCAAACGTATTAATACGCCCCGGGGGCGGGCCTCTGGCGCGCCACTGGATC
AGGCCCGTGGCGTGCCCGCCTCGTCAGCGCCACCCATTGCTAAGCGCTGACAGTAATA
GACCCCTCCATAGTAGTTGCCGATGTTGATTCGGTCACCGGCCGAAACGTGCGCACTCA
GCACAGGGCAGGTACCACGGAGCGAAAGGTGGATGACTGGCAGGGGCCGCTGGCGCA
CCTACAAAACTATTCCTCCGCGCCTGCTGGAGCGACCAACTACGGTTCGCAGCGACCG
GTACCCGAGCAAGCCCTCAACTGGTCCGATGAGACTGTATACGCCGCCGTTGGGACGC
GGACTAAACAACTCCCTCATACCCATCCGCCCGTCCGGAGCAAAAGGACTCGGCGATA
CCGGCGTCAGGCCCCCTCACACAGGCTGCACCATTATGTTGGGAGGTGCGTCGACTGG
GGGCCGGCGACTTGATCCCGGTCGTCCCGTGAAGCCCATCAGTCCCGTGGAGACGTTC
TCTCCCATCCATTGGCCTCCGGAGCTCTCCACCAATCGCACCGGAGTCTTGTCTAGTGT
TAAATGTATTCATCGTGGGGTATAAACCGCCCCCGCGTGAGTGCGGCTCGAAGTCTGG
CCTCCCAGGCTGCTAGCTAACACGGAAGTGTGCCTCGTTTAGCTCAGAGGGGACGAGA
CACTCTCGACGGCTCTCAACAAGCCTAGTCCCCACGATACATACAACCGTGCCCGGTG
GGATTAATACGCCGGGGTCCCGTCGCGGGACATCTGGGATGGGAACACGTTCCGGCCA
TATGCTCTCTGTCCGCATCTCTTGGGAAAATCCATTACGCTGCCGTTGACGTCGTCAAC
GTCACGCCACTAATACCAAACTAGGGGGACCAAACTATACCATGTAATCCGTGCGCCT
ACCCACTGTTGCGATTCAGGGTACGGGGCTGTGAGTAATGTACACACTCCTGGTGGGA
GCTTGTCCTTCGGACTTGAATGGCTACTCGCCGGGCTGGGACTTAGCAAACCACCCTGA
CGCATTCCAGGCCTCTGATCAGATCACAATTTTGCTTTCACCAGTTCCCAATTACGGTT
CCGCATCGTCGGGAGGCCTTCGACTAGATCTACTCGCATGGTTCGAGCGGTAATATCG
GGTCTGAAATCCCCTGACCCAACGGGAGGCGGTACGTGTGATCAGGATCTGCACCTTG
CGAGAGCTAATCGTAATGACGATGACTCTTCCACAAGTGAGTGCTGGGGCGCGAGTGG
CGGACCAGATTGTCTTCCGAATGTTCCCCCCCGTGCCGAAGGCTGAACTCGAGGCGGA
GCCGTGGGCGCGCACCGTTGCCACGCAGGTGCACCGCGAGGCACCTTGAAGGTCCCGT
CATGAAG

>SCWB_ERR173220.1

GTCGGGACGCCCTCCCGGCTCTGGCATGGCGCGGTGGCTTTGCGCACGGATCGGATAA
ACTTTCCATTGGACGGCTCGTTCTCAGAAATATCTCGTAGTCTTCTCGTTGGTTACTCCA
ATGAAACGCCTCGTCTGCGGGGGCACTGAACCGCGAGAGCTATTCAAGTGCTCTACTA
CTCGACCGGGGGCAGCTTACAACCGTGGGATCGGCGCGGCCCACCTCCGGTCAACGAG
AGTTACGAGTACCTGGTCCGTTTTCCCGCGTCGGAAGCTGCCAACACGATAGTAACTA
AACCAGCGCGAGAGCATTGATACGCCTCGGGGGCGGGCCTCTGGCGCGCCACTGGATC
AGGCCCGTGGCGTGCCCGCCTCGTCAGCGCCACCCATTGCTAAGCGCTGACAGTAATA
GACCCCTCCATAGTAGTTGCCGATGTTGATTCGGTCACCGGCCGAAACGTATGCACTTA
GCACAGGGCAGGTACTACAAAGCGAGAGGGGGATGATTGGCAGGGGCTGCCGACGCG
CCTATCAGCCCGTTCCCCCGCGCCTGCTGTGGCGACCGACTACGCCCCGCAGTGACCGG
TACCCGAGCAGTCTCCCAACTGGTCTGATGAGACTGTATACACCGCCGTTGGGATGCG
GACTAAACAACCCCCTCATACCCATCCGCTTGTCCGGAGCGGAACGACTCGACGGTAC
CGGCGTCAAGCGCCTCCGCCTAGGCTGCATCATTATGTTGGGAGATGCGTCGACTGGG
GGCCGGCGACCTGAGCTCGGTCGTCCCGTGAAGCCAATCAGTCTCATGGAGCCGTTCT
CTCCCATCCATTGGCCTCCTGGGCTTTCCACCAATTGCGTCGAAGTATTTTCTAATGTTA
AGTGTGTTTGTCGCGGGACACAAACTGCCCCCGCGCGGGTGCGGAGCGTAATCCGGCC
CCCCAGGCCGCCTGCTAACACGGAAGTGTACCCGGTTCCACTCAGAGGGCATGAGGCA
GTACCTACGGATCTCAACAAGCCTAGTCCCCATGATACGTACAGCTGGGCCAGGTGGG
ATTAATACGCCGGGGTCCCGTCGCGGGATGTCTGAGGGGGGGATACATTCCGGCCGTA
TACTCCCTGTCCGCATCCCTTGGGGTAATCCATTACGCCGTCGTTGACGTCGTCAATGC
CGCGCCACTAACACCGAGCTAGGGGGACCAAAACATACCATGTAATCCGTGTACTTAC
CCATCGTTGCGGTACAGGATACGGGGCTGTGAGTAATGTACACACTCCTGGAGGGAGC
TTGTCCTCCAGACTTTAATGGCTACTCGCCGGGCTGAGACTTAGCAGACCACCCTGACG
CATTCCAGGCCTCAGATTAGATCACAATTTTGCTTTCATTAGTTCCCAATTACGGTTCCG

CATCGTCAGGAGGCCTTCGACTAGATCTACTCGTATGGTTCGAGCGGTGATATCGGGTC
TAAAATCCCCTGACCCAACGGGGGGCGGTACGTGTGATCGGAATCTGCACCTTGCGAG
AGCTAATCGTAATGACGATGGCTCTTCCACAAGTGAGTGCTGGGGCGCGAGCGGCGGA
CCAGATTGTCTTCCGAATGTTCCCCCCCGTGCCGAAGGCTGAACTCGAGGCGGAGCCG
TGGGCGTGCACCGTTGCCACGCAGGTGCATCGCGAAGTCCCTTGAAGGTCCCGTCATG
AAG

>SCWB_ERR173220.2

GTCGGGATGCCCTTTCGACACCGGCACGGCGCGGCGCTTTCGCGCACGGATGGGATAA
GCTTTCCCGTGGATGGCTCGTCCCCGGAGATATCTCGTAGTCTTCTCGTTGGTTACTCCA
ATGAAGTTCCTCGTCTGCGGGGATGCTGAACCGCGAGAGCTCTTAGAGTGCTCTACTAC
CTGGCCGGGGGCAGTATACAACCGTGGGATCGGCGCGGCCCACCTCCGGCCAACGAGG
GGTACAACGTTTCGGCGCGTTTTCCCGCGTCGGAGGCTGCCAACACGATAGTAACTGA
AATGGCCCAAACGTATTAATACGCACAGGGGGCGGGCCTCTGGCGCGCCACTGGATCA
GGCCCGTGGCGTGTCCGCCTCGTCAGCGCCACCCATTGCTAAGCGCTGACAGTAATAG
ACCCCTCCATAGTAGTTGCCGATGTTGATTCGGTCACCGGCCGAAACGTGCGCGCTCAG
CGCAGGGCAGGTACTGTAGAACGAGAGGTGGATATTTGGCAAAGGCTGCTGGCGCGCC
TACCAACCTGTTCCTCCGCGCCTGCTGGAGCGACCAACTACGCCCCGCAGCGACCGGT
ACTTGAGCTGTCCCTCAACTGGTCCGATGAGACTGTATACACCGCCGTTGGGACGCGG
ACTAAACAACCCCCTCATACCCATCTGCCCGTCCGGAGCGGAACGACTCGGCGGTACT
GGCGTCAGGCCCCCTCGCCTAGACTGCACCATTATGTTGGGAGGTGCGTCGACTGGGG
GCCGGCAACTTGAGCTTGGTCGGCCCGTGAAACCCATCAGTCCCATGGAGACGTTCTCT
CCCATCCATTGGCCTCCGGGGCTCTCCATCAATCGCGTCGGAGTATCGTCTAATGTGAA
ATTTATTCATCGTGAGGTATACACCGCCCCCGCGTGGGTGCGGCTCGAAGTCCGGCCCC
CCAGGCTGCCTGCTAACACGGAAGTGTGCCTCGTTTAGCTCAGAAGGGACGAGACACT
CTCGACGGCTCTCAACAAGCCTAGTCCCCACGGTACATACTACCGTGCCAGGTGGGAT
TGGTACGCCGGGGTCCCGTCGCGGGACATCTGGGATGGGAACACGTTCCGGTCATATA
CTCTTTGTCCGCATCCCTTGGGGAAATCCATTACGCTGTCATTGACGTCGTCAACGTCA
TGCCACTAACACCGAACTAGGGGGACAAAACTATACCATGTTATCCGTATGCCTACTC
GTTGTCGCGGTTCAGAGTACGGGCCTGTGAGTAATGTACGCGCTCCTGGTGGGAACTT
GTCCTCCGGACTTGATAGGTTACTCGCCGAGCTGGAACTTAGCAAACCACCCTGATGC
ATTCCAGGTATCAGATTAGAACACAATTTTGCTTTCACCAGTTCCCAATTGCGGGGCCG
TATCGTCGGGAGGCCTTCGACCGGGTCTACTCGCATGGTTCGAGCGGTAATATTGGATC
TGAAATCTCCTGACCCAACGGGAGACGGTGTGCTTGACCGGGATCTGCACCTTGCGAG
AGCTAATCGTAATGACGATGACTCTCCCACAAGTGCGTGGTGTGGCGCGAACGGCGGA
TCATATGGTCTTCCGAATGTTCCCCCCCGTGCCGAAGGCTGAACTCGAGGCGGAGCAG
TGGGCGCGTACCGTCGACATGCAGGTGCACCGCGAGGTCCCTTGAAGATCCCGTCATG
AAG

>NCWB_ERR173221.1

GTCGGGATGCCCTTTCGACACCGGCACGGCGCGGCGCCTTCGCGCACGGATCGGATAA
GCTTACCCGTGGATGGCTCGTCCTCAGAAATATCTCGTAGTCTTCTCGTTGGTTACTCC
AATGAAGCGCCTCGTCTGCTTGGATGTTGAACCACGAGAGCTATTCAAGTGCTTTACTA
CTCGACCGGGGGCAGCTTACAACCGCGGGATTGGCACGGTCTACCTCCGTCCAACGGG
AGTTACGAGTACCCGGCCCGTTTTCCCGCGTCGGAGGCTGCCAACACGATAGTAACTG
AACTGGCCCAAACGTATTGATACGCCCCGGGGGCGGGCCTCTGGCGCGCCACTGGATC
AGGCCCGTGGCGTGCCCGCCTCGTCAGCGCCACCCATTGCTAAGCGCTGACAGTAATA
GACCCCTCCATAGTAGTTGCCGATGTTGATTCGGTCACCGGCCGAAACGTATGCACTTA
GCACAGGGCAGGTACTACAAAGCGAGAGGGGGATGATTGGCAGGGGCTGCTGGCGCG
CCTACCAAACTATTCCTCCGCGCCTGCTGGAGCGACCAACTACGGCCCGCAGCGTCCA

GTACCCGAGCAGGCTCTCAACTGGTCCGATTAGACTGTATACACCGCCGTTGGGACGC
GGACTAAACAACTCCCTCATACCCATCCGCCCGTCCGGAGCGGAACGACTCGGCGGTA
CCGGCGTCAGGCCCCCTCGCCTAGACTGCACCATTATGTTGGGAGGTGCGTCGACTGG
GGGCTGGCAACCTGAGCTCGGTCGTCCCGTGAAGCCCATCAGTCTCATGGAGACGTTC
TCTCCCATCCATTGGCCTCCGGGGCTCTCCACCAATCGCACCGGAGTCTTGTCTAGTGT
TAAATGTATTCATCGTGGGGTATAAACCGCCCCCGCGTGAGTGCGGCTCGAAGTCTGG
CCTCCCAGGCTGGTAGCTAACACGGAAGTGTGCCTCGTTTCGCTCAGAGGGGACGAGA
CACTCTCGACGGCTCTCAACAAGCCTAGTCCCCACGATACATACAACCGTGCCCGGTG
GGGTTAATATGCCTGGGTCCCGCCGCGAAATGTCTAGGATGGGAACACGTTCCGGCCA
TATGCTCTCTGTCCGCATCTCTTGGGGAAATCCATTACGCTGCCGTTGACGTCGTTAAC
GTCACGCCACTAATACCGAACTAGGGGGACCAAACTATACCATGTAATCCGTGCGCCT
ACCCACTGTTGCGATTCAGGGTACGGGGCTGTGAGTAATGTACACACTCCTGGTGTGA
GCTTGTCCTCCGGACTTGAATGGCTACTCGCCGGGCTGGGACTTAGCAAACCACCCTGA
CATATTCCAGGCCTCTGATCAGATCACAATTTTGCTTTCACCAGTTCCCAATTACGGTTC
CGCATCGTCGGGAGGCCTTCGACTAGATCTACTCGCATGGTTCGAGCGGTAATATCGG
GTCTGAAATCCCCTGACCCAACGGGAGGCGGTACGTGTGATCAGGATCTGCACCTTGC
GAGAGCTAAGCGTAATGACGGTGGCTCTTCCACAAGTAAAAGCTCGGGCGCGAGCGGC
GGGCTGGATTGTCTTCTGAATGTTCCCCCCCGTGCCGAAGGCTGAACCCGAGGCTGAG
CCGTGGGCGCGCACCGTTGCCACGCAGGTGCACCGCGAGGTCCCTTGAAGGTCCCGTC
ACGAAC

>NCWB_ERR173221.2

GTCGGGATGCCTTCCCAACTCTGGCACGGCGCGGCGCCTTCGCGCACGGATCGGATAA
ACTTTCCCGTGGACGGCTCGTCCTCAGAAACATTTCGTAGTCTTCTCGTTGGTTACTCCA
CTGCCGCGCCGCGTCTGCGGGGGCACTGAACCGCGAGAGCTATTAGAGTGCTCTACTA
CTCGACCGGGGGCAGCATAGCACCGTGGGGTCGGCGCGGCCCACCTCCGGCCAACGAG
AGTTACGAGTACCCGGCCCGTTTTCCCGCGTCGGCGGCTGCCAACACGATAGTAACTG
AACTGGCCCAAACGTATTGATACGCCCCGGGGGCGGGCCTCTGGCGCGCCACTGGATC
AGGCCCGTGGCGTGCCCGCCTCGTCAGCGCCACCCATTGCTAAGCGCTGACAGTAATA
GACCCCTCCATAGTAGTTGCCGATGTTGATTCGGTCACCGGCCGAAACGTATGAACTCA
GCACAGGGCAGGTACTACAAAGCGAGAGGGGGATGATTGGCAGGGGCTGCTGACGCG
CCTATCAGCCCGTTCCCCCGCGCCTGCTGTGGCGACCAACTACGCCCCGCAGTATCCAG
TACCCGAGCAGTCTCTCAACTGGTCCGATTAGACTGTATACACCGCCGTTGGGACGCG
GACTAAACAACTCCCTCATACCCATCCGTCCGTCCGGAGCGGAACGACTCGGCGGTAC
CGGCGTCAGGCCCCCTCGCACAGGCTGCACCATTATGTTGGGAGGTGCGTCGACTGGG
GGCCGGCGACTTGAGCTCGGTCGTCCCGTGAAGTCCACCAGTCCCATGGAGAAGTTCT
CTCCCATCCATTGGCCTCCTGGACTCTCCACCAATCGCACCGGAGTCTTGTCTAATATG
AAATTTATTCATCGTGGGGTATAAACCGCCCCCGCGTGAGTGCGGCTCGAAGTCTGGC
CTCCCAGGTTGGTAGCTAACACGGAAGTGTGCCTCGTTTCGCTCAGAGGGGACGAGAC
ACTCTCGACGGCCCTCAACAAGCCTAGTCCCCACGATACATACAACCGTGCCCGGTGG
GATTAATACGCCGGGGTCCCGTCGCGGGACATCTGGGATGGGAACACATTCCGGCCGT
ATACTCTTTGTCCGCATCTCTTGGGGAAATCCATTACGCTGCCGTTGACGTCGTTAACG
TCACGCCACTAATACCGAACTAGTGGGACCAAACTATACCATGTAATCCGTGCGCCTG
CCCACTGTTGCGATTCAGGGTACGGGGCTGTGAGTAATGTACACACTCCTGGAGTGAG
CTTGTCCTCCGAACTTGAATAGCTACTCGCCGGGCTGGGACTTAGCAAACCACCCTGAC
GCATTCCAGGCCTCTGATCAGATCACAATTTTGCTTTCACCAGTTCCCAATTACGGTTC
CGCATCGTCGGGAGGCCTTCGACTAGATCTACTCGCATGGTTCGAGCGGTAATATCGG
GTCTGAAATCCCCTGACCCAACGGGGGGCAGTATGTGTGGTCGGAATCTGCACCTTGC
GAGAGATAATCGTAATGACGATGGACCTTCTACAAGTAAAAGCTCGGGCGCGAGCGGC
GGGCTGGATGGTCCCCCGAATGTTCCCCCCTATGCCGAAGGCTGAACTCGAGGCTGAG

CCGTGGGCGCGCACCGTTGACACGCAGGTGCACCGCGAGGTCCCTTGAAGGTCCCGTC
ACGAAG

>NCWB_ERR173222.1

GTCGGGACGCCCTCCCGGCTCTGGCATGGCGCGGTGGCTTTGCGCACGGATCGGATAA
ACTTTCCATTGGACGGCTTGTCCTCAGAAGCATTTCGTAGTCTTCTCGTTGGCTACCCTG
ATGCCGCGCCGCGTCTGCTTGGATGCTGAACCGCGAGAGCTATTCGAGTGCTCTACTAT
TCGACCGGGGGCAGCACGCAGCCGTGGGATCGGTGCGACCCTCTTTCGGCTAGTAGGG
GGTACGACGTCCCGGCCCGTTTTCCCGCGTCGGAGGCTGCCAACACGGCAGTAACTGA
ACTGGCCCAAACGTATTAATACGCACAGGGGGCGGGCCTCTGGCGCGCCACTGGATCA
GGCCCGTGGCGTGCCCGCCTCGTCAGCGCCACCCATTGCTAAGCGCTGACAGTAATAG
ACCCCTCTATAGTAGTTGCCGATGTTGATTTGGTCACCGGCCGAAACGTATGCGCTCAG
CACAGGGCAGGTACTACGGAGCGAAAGGTGGATGATTGGCAGGGGCCGCTGGCGCAC
CTACAAAACTATTCGTCCGCGCCTGCTGGAGCGACCAACTACGCTCTGTAGCGTCCAGT
ACCCGAGCAGTCCCTCAATTGGTCCGATGAGACTGTATACGCCGCCGTTGGGACGCGG
ACTAAACAACCCCCTCATACCCATTCGCCCGTCCAGAGCGGAACGACTCCGCGGTGCC
GGCGTCAGGCCTCCTCGCCTAGGCTGCGCCATTATGTTGGGAGGTGCGTTGACTGGGG
GCTGGCAATTTGATCCCGGTCGGCCCGTGAAGCCCATCAGTTCCATGGAGACATTCTTT
GCCGTCCATTGGCCTTCTGGGCTCTCCGCCAATTACGTCGGAGTATCGCCTAGGGTGAA
ATGCATTCAACGTGAGGTATAAACCGCCCCCGCGTGGGTGCGGCTCGATGTCTGGCCT
CCCAGGCTGGCAGCTAACACGGAAGTGTGCCTCGTTCCACTCAGAGGGCATGAGGCAG
TACCTACGGATCTCAACAAGCCTAGTCCCCATGATACGTTCAGCTGGGCCAGGTGGGA
TTAAGATGCCTGGGTCCCGCCGCGAAACGGCTAGAAGGTAAATACGCCCTGGCCATAT
ACTCTTTGTCCGCATCCCTTGGGGAAATCCATTATGCTGCCGTTGACGTCGTCAACGTC
ACGCCACTAACACCGAACTAGGGGGACCAAACTATACCATGTTATCCGTATACTTACC
CACTGTTGCAGGTCAAGGTATGGGGCTGCAAGTAATGTACATACTCCTGGTGGGAACT
TGTCCTCCGGACTTGAATGGCTACCTGCCGGGCTGGGACTTAGCAAACCACCCTGACG
CATCCCAGGCCTTTGATCGGATCACAATTTTGATTCCACCAGTTCCCAATTACGGTTCC
GCATCGTTGGGAGGCCTTTGGCTAGATCTACCTGCATGGTTCGAGCGGTAATATCGGGT
CTGAAATCCCCTGACCCAACGGGAGGCAGTATGTGTGGTCGGAATCTGCACCTTGCGA
GAGATAATCGTAATGACGATGGACCTTCTACAAGTGAGTGCTGGGGCACGAGCGGCGG
ACCAGATGGTCTTCTGAATGTTCCTTCCCGTGCCGAAGGCTGAACTCGAGGCGGAGCA
GTGGGCGCGTACCGTCGACATGCAGGTGCACCGCGAGGTCCCTTGAAGATCCCGTCAT
CAAC

>NCWB_ERR173222.2

GTCGGGATGCCTTCCCAACTCCGGCACGGCGCGGCGCCTTCGCGCACGGATCGGATAA
GCTTACCCGTGGATGGCTCGTTCTCAGAAATATCTCGTAGTCTTCTCGTTGGTTACTCCA
ATGAAGCGCCTCGTCTGCGGGGGCACTGAACCGCGAGAGCTATTCAAGTGCTCTACTA
CTCGACCGGGGGCAGCTTACAACCGCGGGATCGGCACGGTCTACCTCCGTCCAACAGG
GGGTACGACGACCCGGTCCCCCCTCCCGCGTCGGAGGCTGCCAACACGATAGTAACTG
AACTGGCCCAAACGTATTAATACGCCCCGGGGGCGGGCCTCCGGCGCGCCACTGGATC
AGGCCCGTGGCGTGCCCGCCCCGTCAGCGCCACCCATTGCTAAGCGCTGACAGTAATA
GACCCCTCCATAGTAGTTGCCGATGTTGATTCGGTCACCGGCCGAAACGTATGCACTTA
ACACAGGGCAGGTACTACAAAGCGAGAGGGGGATGATTGGCAGGGGCTGCTGACGCG
CCTATCAGCCCGTTCCCCCGCGCCTGCTGTAGCGACCAACTACGCCCCGCAGTGACCGG
TACCCGAGCAGTCTCCCAATTGGTCCGATGAGACTGTATACGCCGCCGTTGGGACGCG
GACTAAACAACCCCCTCATACCCATTCGCCCGTCCGGAGCGGAACGACTCGGCGGTAC
CGGCGTCAGGCCCCCTCGCCTAGACTGCACCATTATGTTGGGAGGTGCGTCGACTGGG
GGCCGGCGACTTGATCCTGGTCGGCCCGTGAGGCCCATCAGTCCTATGGAGACGTTCTT

TCCCATCCATTGGCCCCCGGGGTCCTCTACCGATTGCGTCGGAGTCTTGTATAATATGA
AATTTATTCATCGTGAGGCATACACCGCCCCCGCGTGGGTGCGGCTCGAAGCCCGGCTT
CCCAGGTTGGCAGCTAACACGGAAGTGTGCCTCGTTTCGCTCAGGAGGGACGAGACAC
TCTCGACGGCTCTCAACAAGCCTAGTCCCCACGATACATACAACCGTGCCAGGTGGAA
TTGGTACGCCGGGGTCCCGTCGCAGGATGTCTAGAAGGTAAATACGCCCTGGCCATAT
ACTCTTTGTCCGCATCCCTTGGGGAAATCCATTATGCTGCCGTTGACGTCGTCAACGTC
ACGCCACTAACACCGAACTAGGGGGACCAAACTATACCATGTTATCCGTATACTTACC
CACTGTTGCAGGTCAAGGTATGGGGCTGCAAGTAATGTACATACTCCTGGTGGGAACT
TGTCCTCCGGACTTGAATGGCTACCTGCCGGGCTGGGACTTAGCAAACCACCCTGACG
CATCCCAGGCCTTTGATCGGATCACAATTTTGATTCCACCAGTTCCCAATTACGGTTCC
GCATCGTTGGGAGGCCTTTGGCTAGATCTACCTGCATGGTTCGAGCGGTAATATCGGGT
CTGAAATCCCCTGACCCAACGGGAGGCAGTATGTGTGGTCGGAATCTGCACCTTGCGA
GAGATAATCGTAATGACGATGGACCTTCTACAAGTGAGTGCTGGGGCACGAGCGGCGG
ACCAGATGGTCTTCTGAATGTTCCTTCCCGTGCCGAAGGCTGAACTCGAGGCGGAGCA
GTGGGCGCGTACCGTCGACATGCAGGTGCACCGCGAGGTCCCTTGAAGATCCCGTCAT
CAAC

>Xiang_ERR173223.1

GTCGGGACGCCCTCCCGGCTCTGGCACGGCGCGGTGGCTTTGCGCACGGATCGGATAA
ACTTTCCATTGGACGGCTCGTTCTCAGAAATATCTCGTAGTCTTCTCGTTGGTTACTCCA
ATGAAACGCCTCGTCTGCGGGGGCACTGAACCGCGAGAGCTATTCAAATGCTCTACTA
CTCGACCGGGGGCAGCTTACAACCGTGGGATCGGCGCGGCCCACCTCCGGTCAATAGG
AGTTACGACGTCCCGGCCCGTTTTCTCGCGTCGGAGGCTGCCAACACGATAGTAACTG
AACTGGCCCAAACGTATTAATACGCCCCGGGGGCGGGCCTCTGGCGCGCCACTGGATC
AGGCCCGTGGCGTGCCCGCCTCGTCAGCGCCACCCATTGCTAAGCGCTGACAGTAATA
GACCCCTCCATAGTAGTTGCCGATGTTGATTTGGTCACCGGCCGAAACGTATGCGCTCA
GCACAGGGCAGGTACTACGGAGCGAAAGGTGGATGATTGGCAGGGGCCGCTGGCGCA
CCTACAAAACTATTCGTCCGCGCCTGCTGGAGCGACCAACTACGCTCTATAGCGTCCAG
TACCCGAGCAGTCCCTCAACTGGTCCGATGAGACTGTATACACCGCCGTTGGGACGCG
GACTAAACAACCCCCTCATACCCATTCGCCCGTCCGGAGCGGAACGACTCCGCGGTGC
CGGCGTCAGGCCTCCTCGCCTAGGCTGCGCCATTATGTTGGGAGGTGCGTTGACTGGG
GGCCGGCGACCTGAGCTTGGTCGGCCCGTGAAGCCCATCAGTCCCATGGGGACGTTCT
CTCCCATCCATTGGCCTCTGGGGCTTTCCACCAATTGCACCAGAATAATGCCAGATGTT
AGATGTATTCATCGTGGGGCACGAGCCGCCCCCGCGTGAGTGCGGCTCGAAATCCGGT
CCCCCAGGCTGCCAGCTAACACGGAAGTGTGCCCGGTTCCACTCAGAGGGCATGAGGC
AGTACCTACGGATCTCAACAAGCCTAGTCCCCATGATACGTACAGCTGGGCCAGGTGG
GATTAATACGCCGGGGTCCCGTCGCGGGATGTCTAGGGGGGAGATACGTTCCGGCCAT
ATACTCTCTGTCCACATCCCTTGAGGAAATCCATTACGCTGCCGTTGACGTCGTTAACG
CCGCTTCATTAACGTCGAACCAGGGAGACCAAACTATACCATGTTATCCGTATGCCCAC
CCATTTTTGCGGTTCAGAGTACTGGCCTGTGAGTAATGTACGCGCTCCTGGTGGGAACT
TGTCCTCCGGACTTGATAGGTTACTCGCCGAGCTGGAACTTAGCAAACCACCCTGATGC
ATTCCAGGTATCAGATTAGAACACAATTTTGCTTTCACCAGTTCCCAATTGCGGGGCCG
TATCGTCGGGAGGCCTTCGACTAGATCTGCTCGCATGGTTCGAGCGGTAATATCGGGTC
TGAAATCCCCTGACCCAACGGGAGGCGGTATGCGTGATCGGAATCTGCACCTTGCGAG
AGCTAATCGTGATGACGATGGCTCTTCCACAAGTGAGTGCTGGGGCGCGAGCGGCGAA
CCAGATGGTCCTCTGAATGTTCCCCCCCGTGCCGAAGGCTGAACTCGAGGCGGGGCCG
TGGTCGCGCACCGTTACCACGCAGGTGCGTCGCGAGTCAACTTGAAGGTCCAGTCATG
AAG

>Xiang_ERR173223.2

GTCGGGATGCCCTTTCGACACCAGCACGGCGCGACGCTTTCGCGCACGTATCAGATAA
GTTTTCCCGTGGACGGCTCGTCCTCAGAAATATCTCGTAGTCTTTTCGTTGGTTACTCCA
ATGAAGCGCCTCGCCTGCGGGGGCACTAAACCGCGAGCGCCATTCGAGTGCTCTACAA
CTCGACCGGGGGCAGCACACAACCGTAGGATCGGCGCGGCTCATTTTCGGTCAACGAG
AGTTACGAGTTCCCGGCCCGTTTTCTCGCGTCGGAGGCTGCCAACACGATAGTAACTGA
ACTGGCCCAAACGTATTGATACGCCCCGGGGGCGGGCCTCTGGCGCGCCACTGGATCA
GGCCCGTGGCGTGCCCGCCTCGTCAGCGCCACCCATTGCTAAGCGCTGACAGTAATAG
ACCCCTCCATAGTAGTTGCCGATGTTGATTCGGTCACCGGCCGAAACGTATGCACTTAG
CACAGGGCAGGTACTACAAAGCGAGAGGGGGATGATTGGCAGGGGCTGCTGACGCGC
CTATCAGCCCGTTCCCCCGCGCCTGCTGTGGCGACCAACTACGCCCCGCAGCGTCCAGT
ACCCGAGCAGTCTCTCAACTGGTCCGATTAGACTGTATACACCGCCGTTGGGACGCGG
ACTAAACAACTCCCTCATACCCATCCGCCCGTCCGGAGCGGAACGACTCGGCGGTACC
GGCGTCAGGCCCCCTCGCCTAGACTGCACCATTATGTTGGGAGGTGCGTCGACTGGGG
GCCGGCGACTTGATCCTGGTCGGCCCGTGAAGCCCATCAGTCCCATGGAGACGTTCTCT
CCCATCTATTGGCCTCCGGGGCTCTCCACCAATCGCACCGGAGTCTTGTCTAATATGAA
ATTTATTCATCGTGAGGTATAAACCGCCCCCGCGTGGGTGCGGCTCGAAGTCCGGCTTC
CCAAGCTGGCTGCTAACACGGAAGTGTACCCGGTTCCACTCAGAGGGCCTGAGGCAGT
ACCTACGGATCTCAAAAACCCTAGTCCCCATGATACGTACAGCTGGGCTAGGTGGGAT
TAATACGTCGGGGTTCCGTTGCGGAACGGCTAGAAGGGAAATACGCCTTGGCCATATA
CTCTTTGTCCGCATCCCTTGGGGAAATCCATTATGCTGCCGTTGACGTCGTCAACGTCA
CGCCACTAACACCGAACTAGGGGGACCAAACTATACCATGTTATCCGTATACTTACCC
ACTGTTGCAGGTCAAGGTATGGGGCTGTAAGTAATGTACATACTCCTGGTGGGAACTT
GTCCTCCGGACTTGAATGGCTACCTGCCGGGCTGGGACTTAGCAAACCACCCTGACGC
ATCCCAGGCCTTTGATCGGATCACAATTTTGCTTTCACCAGTTCCCAATTACGGTTCCG
CATCGTCGGGAGGCCTTCGACTAGATCTACTCGCATGGTTCGAGCAGTAATATCGGGTC
TGAAATCCCCTGACCCAACGGGGGGCAGTATGTGTGGTCGGAATCTGCACCTTGCGAG
AGATAATCGTAATGACGATGGACCTTCTACAAGTGAGTGCTGGGGCACGAGCGGCTGA
CCAGATTGTCTTCCGAATGTTCCCCCCCGTGCCGAAGGCTGAACCCGAGGCGGAGCCG
TGGGCGTGCACCGTTGCCACGCAGGTGCACCGCGAGGCACCTTGAAGGTCCCGTCATG
AAG

>Xiang_ERR173224.1

GTCGGGATGCCCTTTCGACACCAGCACGGCGCGACGCTTTCGCGCACGTATCAGATAA
GTTTTCCCGTGGACGGCTCGTCCTCAGAAATATCTCGTAGTCCTTTCGTTGGTTACTCCA
ATGAAGCGCCTCGCCTGCGGGAGCACTAAACCGCGAGCGCCATTCGAGTGCTCTACAA
CTCGACCGGGGGCAGCACACAACCGTAGGATCGGCGCGGCTCATTTTCGGCTAACAGG
GGTTACGACGTCCCGGCCCGTTTTCTCGCGTCGGAGGCTGCCAACACGATAGTAACTG
AACTGGCCCAAACGTATTGATACGCCCAGGGGGCGGGCCTCTGGCGCGCCACTGGATC
AGGCCCGTGGCGTGCCCGCCTCGTCAGCGCCACCCATTGCTAAGCGCTGACAGTAATA
GACCCCTCCATAGTAGTTGCCGATGTTGATTTGGTCACCGGCCGAAACGTATGCGCTCA
GCACAGGGCAGGTACTACGGAGCGAAAGGTGGATGATTGGCAGGGGCCGCTGGCGCA
CCTACAAAACTATTCGTCCGCGCCTGCTGGAGCGACCAACTACGCTCTATAGCGTCCAG
TACCCGAGCAGTCCCTCAATTGGTCCGATGAGACTGTATACGCCGCCGTTGGGACGCG
GACTAAACAACCCCCTCATACCCATTCGCCCGTCCGGAGCGGAACGACTCCGCGGTGC
CGGCGTCAGGCCTCCTCGCCTAGGCTGCGCCATTATGTTGGGAGGTGCGTTGACTGGG
GGCCGGCGACCTGAGCTTGGTCGGCCCGTGAAGCCCATCAGTCCCATGGGGACGTTCT
CTCCCATCCATTGGCCTCTGGGGCTTTCCACCAATTGCACCAGAATAATGCCAGATGTT
AGATGTATTCATCGTGGGGCACGAGCCGCCCCCGCGTGAGTGCGGCTCGAAATCCGGT
CCCCCAGGCTGCCAGCTAACACGGAAGTGTGCCTCGTTCCGCTCAGAGGGGACGAGAC
ACTCTCGATGGCTCTCAACAAGCCTAGTCTCCACGATACATACAATCGTGCCAGGTGG
GATTAATACGCCGGGGTTCCGTTGCGGAACGGCTAGAAGGTAAATACGCCTTGGCCAT

ATACTCTTTGTCCGCATCCCTTGGGGAAATCCATTATGCTGCCGTTGACGTCGTCAACG
TCACGCCACTAACACCGGACTAGGGGGACCAAACCATACCATGTAATCCGTGTACTTG
CCCATTGTTACGGTTTAGGGAACGGGGCTGTGGCTAATAGACGCGCTCCTGGTGGGAG
CTTGTCCTCCGGACTCGAATGGCTACTCGCCGGGCTGGGACTTAGCAAACCGCTCTGAC
GCATCTACGGCCTCAGATTAGATCACAATTTTGCTTTCTCCATTTCCTAATTACCGTTCC
GCATCGTCGGGAGGCCTTCGACTAGATTTACTCGCATGGTTTAAGTGATACAGTCGGGT
CGGGAATCCTCTGACCCAACGTGAAGCGGTATGCGCAATTGGGGTCTGTTCCTTGCAA
GAGCTAATCGTAATGACGATGGACCTTCTACAAGTGAGTGCTGGGGCACGAGCGGCTG
ACCAGATTGTCTTCCGAATGTTCCCCCCCGTGCCGAAGGCTGAACCCGAGGCGGAGCC
GTGGGCGTGCACCGTTGCCACGCAGGTGCACCGCGAGGCACCTTGAAGGTCCCGTCAT
GAAG

>Xiang_ERR173224.2

GTCGGGATGCCTTCCCAACTCCGGCACGGCGCGGCGCCTTCGCGCACGGATCGGATAA
GCTTACCCGTGGACCGCTCGTCCTCGGAAATATCCCGAGGTTTTCCCAGTGGTTACCTT
GATGCCGCGCAGCGTCTGCTTGGATGCTGAACCGAGAGAGCCATTCGAGTGCTCTACA
ACTCGACCGGGGACAGCACACAATTGTGGGATCGGCGCGGCCCACCTCCGGCCAACGA
GAGTTACGAGTACCCGGCCCGTTTCCCCGCATCGGAGGCTGCCAACACGATAGTAACT
GAACTGGCCCAAACGTATTAACACGCACAGGGGGCGGGCCTCTGGCGCGCCACTGGAT
CAGGCCCGTGGCGTGCCCGCCTCGTCAGCGCCACCCATTGCTAAGCGCTGACAGTAAT
AGACCCCTCCATAGTAGTTGCCGATGTTGATTTGGTCACCGGCCGAAACGTATGCGCTC
AGCACAGGGCAGGTACTACGGAGCGAAAGGTGGATGATTGGCAGGGGCCGCTGGCGC
ACCTACAAAACTATTCGTCCGCGCCTGCTGGAGCGACCAACTACGCTCTATAGCGTCCA
GTACCCGAGCAGTCCCTCAATTGGTCCGATGAGACTGTATACGCCGCCGTTGGGACGC
GGACTAAACAACCCCCTCATACCCATTCGCCCGTCCGGAGCGGAACGACTCCGCGGTG
CCGGCGTCAGGCCTCCTCGCCTAGGCTGCGCCATTATGTTGGGAGGTGCGTTGACTGGG
GGCCGGCGACCTGAGCTTGGTCGGCCCGTGAAGCCCATCAGTCCCATGGGGACGTTCT
CTCCCATCCATTGGCCTCTGGGGCTTTCCACCAATTGCACCAGAATAATGCCAGATGTT
AGATGTATTCATCGTGGGGCACGAGCCGCCCCCGCGTGAGTGCGGCTCGAAATCCGGT
CCCCCAGGCTGCCAGCTAACACGGAAGTGTGCCTCGTTCCGCTCAGAGGGGACGAGAC
ACTCTCGATGGCTCTCAACAAGCCTAGTCTCCACGATACATACAATCGTGCCAGGTGG
GATTAATACGCCGGGGTTCCGTTGCGGAACGGCTAGAAGGTAAATACGCCTTGGCCAT
ATACTCTTTGTCCGCATCCCTTGGGGAAATCCATTATGCTGCCGTTGACGTCGTCAACG
TCACGCCACTAACACCGGACTAGGGGGACCAAACCATACCATGTAATCCGTGTACTTG
CCCATTGTTACGGTTTAGGGAACGGGGCTGTGGCTAATAGACGCGCTCCTGGTGGGAG
CTTGTCCTCCGGACTCGAATGGCTACTCGCCGGGCTGGGACTTAGCAAACCACTCTGAC
GCATCTACGGCCTCAGATTAGATCACAATTTTGCTTTCTCCATTTCCTAATTACCGTTCC
GCATCGTCGGGAGGCCTTCGACTAGATTTACTCGCATGGTTTAAGTGATAATACCGGGT
CGGGAATCCTCTGACCCAACGTGAAGCGGTATGCGCAATTGGGGTCTGTTCCTTGCAA
GAGCTAATCGTAATGACGATGGACCTTCTACAAGTGAGTGCTGGGGCACGAGCGGCTG
ACCAGATTGTCTTCCGAATGTTCCCCCCCGTGCCGAAGGCTGAACCCGAGGCGGAGCC
GTGGGCGTGCACCGTTGCCACGCAGGTGCACCGCGAGGCACCTTGAAGGTCCCGTCAT
GAAG

>EHL_0F0202.1

GTCGGGATGCCCTTTCGACACCAGCACGGCGCGACGCTTTCGCGCACGTATCAGATAA
GTTTTCCCGTGGACGGCTCGTCCTCAGAAATATCTCGTAGTCCTTTCGTTGGTTACTCCA
ATGAAGCGCCTCGCCTGCGGGAGCACTAAACCGCGAGCGCCATTCGAGTGCTCTACAA
CTCGACCGGGGGCAGCACACAACCGTAGGATCGGCGCGGCTCATTTTCGGCTAGTAGG
GGGTACGACGTCCCGGCCCGTTTTCTCGCGTCGGAGGCTGCCAACACGATAGTAACTG

AACTGGCCCAAACGTATTGATACCCCCAGGGGGCGGCCTCTGGCGCGCCACTGGATC
AGGCCCGTGGCGTGCCCGCCTCGTCAGCGCCACCCATTGCTAAGCGCTGACAGTAATA
GACCCCTCCATAGTAGTTGCCGATGTAGGTCTGGTCACCGGCCGAAACGTATGAACGC
AGCACAGGGCAGGTACTACGGAGCGAAAGGTAGCTGATTGGCAGGGGCTGCTGGCGC
GCCTACCAACCTGTTCCTCCGCGCCTGCTGGAGCGACCAACTACGCCCCGCAGCGACC
GGTACCCGAGCAGTCTCTCAACTGGTTCGATGAGACTGTATACACCGTCGTTGGGACG
CGGACTAAACCGCCCCCTCATACCCACCCGCCCGTCCGGAGCGGAACGACTCGGCGGT
ACCGGCGTCAGGCCCCCTCGCCTAGGCTGCACCATTATGTTGGGAGGTGCGTCGACTG
GGGGCCGGCGACTTGAGCTCGGTCGTCCCGTGAAGCCCATCAGTCCCATGGAGACGTT
CTCTCCCATCCATTGGCCTCCGGGGCTCTCCACCAATCGCACCGGAGTCTTGTCTAGTG
TTAAATGTATTCATCGTGGGGTATAAACCGCCCCCGCGTAAGTGCGGCTCGAAGTCTG
GCCTCCCAGGCTGCTAGCAAACACGGAAGTGTGCCTCGTCTCGCTCAGAGGGGACAAG
ACACTCTCGACGGCTCTCAACAAGCCTAGTCCCCACGATACATACAACCGTGCCCGGT
GGGATTAATACGCCGGGGTCCCGTCGCGGGACATCTGGGATGGGAACACGTTCCGACC
ATATGCTCTCTGTCTGCATCTCTTGGGGAAATCCATTACGCTGCCGTTGACGTCGTTAA
CGTCACGCCACTAATACCGAACTAGGGGGGCCAAGCTATACCATGTAATCCGTGCGCC
TACCCACTGTTGCGATTCAGGGTACGGGGCTGTGAGTAATGTACACACTCCTGGTGTGA
GCTTGTCCTCCGGACTTGAATGGCTACTCGCCGGGCTGGGACTTAGCAAACCACCCTGA
CGCATTCCAGGCCTCTGATCAGATCACAATTTTGCTTTCACCAGTTCCCAATTACGGTT
CCGCCTCGTCGGGAGGCCTTCGACTAGATCTACTCGCATGGTTCGAGCGGTAATATCGG
GTCTGAAATCCCCTGACCCAACGGGAGGCGGTACGTGTGATCAGGATCTGCACCTTGC
GAGAGCCAAGCGTAATGACGGTGGCTCTTCCACAAGTAAAAGCTCGGGCGCGAGCGG
CGGGCTGGATGGTCCCCCGAATGTTCCCCCCTATGCCGCAGGCTGAACTCGAGGCTGA
GCCGTGGGCGCGCACCGTTGCCACGCAGGTGCACCGCGAGGTCCCTTGAAGGTCCCGT
CACGAAG

>EHL_0F0202.2

GTCGGGATGCCTTCCCAACTCCGGCACGGCGCGGCGCCTTCGCGCACGGATCGGATAA
GCTTACCCGTGGATGGCTCGTTCTCAGAAATATCTCGTAGTCTTCTCGTTGGTTACTCCA
ATGAAGCGCCTCGTCTGCGGGGGCACTGAACCGCGAGAGCTATTCAAGTGCTCTACTA
CTCGACCGGGGGCAGCTTACAACCGCGGGATTGGCACGGTCTACCTCCGTCCAACAGG
GGGTACGACGACCCGGTCCCCCCTCCCGCGTCGAAGGCTGCCAACACGATAGTAAGCG
AACTGGCCCAAACGTATTAATACGTACAGAGGGCGAGCCTCTGGCGCGCCACTGGATC
AGGCCCGTGGCGTGCCCGCCTCGTCAGTGCCACCCATTGCTAAGCGCTGACAGTAATA
GACCCCTCCATAGTAGTTGCCGATGTTGATTCGGTCACCGGCCGAAACGTGCGCGCTCA
GCACAGGGCAGGTACCACGGAGCGAAAGGTGGATGATTGGCAGGGGCTGCTGACGCG
CCTATCAGCCCGTTCCCCCGCGCCTGCTGTGGCGACCAACTACGCCCCGCAGCGTCCAG
TACCCGAGCAGTCTCTCAACTGGTCCGATTAGACTGTATACACCGCCGTTGGGACGCG
GACTAAACAACTCCCTCATACCCATCCGCCCGTCCGGAGCGGAACGACTCGGCGGTAC
CGGCGTCAGGCCCCCTCGCCTAGACTGCACCATTATGTTGGGAGGTGCGTCGACTGGG
GGCCGGCGACTTGATCCTGGTCGGCCCGTGAAGCCCATCAGTCCCATGGAGACGTTCT
CTCCCATCTATTGGCCTCCGGGGCTCTCCACCAATCGCACCGGAGTCTTGTCTAATATG
CAATTTATTCATCGTGAGGTATAAACCGCCCCCGAGTGGGTGCGGCTTGAAGTCCAGC
CCCCCAGGCCGCCTGCTAACACGGAAGTGTACCCGGTTCCACTCAGAGGGCATGAGGC
AGTACCTACGGATCTCAACAAGCCTAGTCCCCATGATACGTACAGCTGGGCCAGGTGG
GATTAATACGCCGGGGTCCCGTTGCGGGATGTCTAGGGGGGAGATACGTTCCGGTCAT
ATACTCTTTGTCCGCATCCCTTGGGGAAATCCATTATGCTCCCGTTGACGTCGTCAACG
TCACGCCACTAACACCGAACTAGGGGGACCAAACTATACCATGTTATCCGTATACTTA
CCCACTGTTGCAGGTCAAGGTATGGGGCTGCAAGTAATGTACATACTCCTGGTGGGAG
CTTGTCCTCCGGACTCGAATGGCTACTCGCCGGGCTGGGACTTAGCAAACCACTCTGAC
GCATCTACGGCCTCAGATTAGATCACAAGCTTGCTTTCTCCATTTCCTAATTACCGTTCC

GCATCGTCGGGAGGCCTTCGACTAGATTTACTCGCATGGTTCAAGCGATAATATCAAGT
CTGGAATCCTCTGACCCAACGTGAAGCGGTATGCGCAATTGGGGTCTGTTCCTTGCAAG
AGCTAATCGTAATGACGATGGACCTTCTACAAGTGAGTGCTGTAGCGCCAGCAGCGGA
CCAGATTGTTTTCCGGATGTTCCCCCTCGTGCCGAAGGCTGAACTCAGGGCGGGGCCGT
GGGCGCGCATCGTTGCCACGCAGGTACACCGTGAGGCACCTTGAAGGTCCCGTCATGA
AG

>EHL_0F0090.1

GTCGGGATGCCCTCTCGATACTGGCACGGCGCGGCGCCTTCGCGCACGGATCGGATAA
GCTTTCCCGTAGATGGCTCGTCCTCGGAAATATCTCGTAGTCTTCTCGTTGGTTGCTCCA
ACGAAGCGACGAATCTACTTGGATACTGAACCGCGAGAGCTATTCAAGTGCTCTACTA
CTCGACCGGGGGCAGCATACAGCCGTGGGATCGGCGCGGCCCACCTCCGGCCAACGAG
GGGTACGACGTTTCGGCGCGTTTTCCCGCGTCGGAGGCTGCCAACACGATAGTAACTG
AAATGGCCCAAACGTATTAATACGCACAGGGGGCGGGCCTCTGGCGCGCCACTGGATC
AGGCCCGTGGCGTGCCCGCCTCGTCAGCGCCACCCATTGCTAAGCGCTGACAGTAATA
GACCCCTCCATAGTAGTTGCCGATGTTGATTTGGTCACCGGCCGAAACGTATGCGCTCA
GCACAGGGCAGGTACTACGGAGCGAAAGGTGGATGATTGGCAGGGGCCGCTGGCGCA
CCTACAAAACTATTCGTCCGCGCCTGCTGGAGCGACCAACTACGCTCTATAGCGTCCAG
TACCCGAGCAGTCCCTCAATTGGTCCGATGAGACTGTATACGCCGCCGTTGGGACGCG
GACTAAACAACCCCCTCATACCCATTCGCCCGTCCAGAGCGGAACGACTCCGCGGTGC
CGGCGTCAGGCCTCCTCGCCTAGGCTGCGCCATTATGTTGGGAGGTGCGTTGACTGGG
GGCCGGCGACCTGAGCTTGGTCGGCCCGTGAAGCCCATCACTCCCATGGAGACGTTTT
CTCCCACCCATTGGCCCCCGGGGTCCTCCACCAATTGCGTCGGAGTCTTGTCTAATATG
AAATTTATTCATCGTAAGGTATAAACCGCCCCCGCGTGGGTGCGGCTCGAAGTCCGGC
TTCCCAAGCTGGCTGCTAACACGGAAGTGTACCCGGTTCCACTCAGAGGGCCTGAGGC
AGTACCTACGGATCTCAAAAACCCTAGTCCCCATGATACGTACAGCTGGGCTAGGTGG
GATTAATACGTCGGGGTCCCATCGCGGGATGTTTGAGGGGGAGATACGTTCCAGCCAT
ATACTCCCTGTTCGCGCCCCTCAGAGAAGTCCATTACGCTGCCGTTGACGTCGTCAATG
CCGCGCCACTAACACCGGACTAGGGGGACCAAACCATACCATGTAATACGTGTACTTG
CCCATTGTCGCGGTTCAGAGTACGGGGCTGTGAGTAATGTACGCGTTCCTGGTGGGAA
CTTGTCCTCTGAACTTGAATAACAGCTCACTGGAATGGGATTTCAAAAACCACCCTGAC
GCATTCCAGGCCTCTGATCAGATCACAGTTTTGCTTTCACCAGTTCCCAATTACGGTTC
CGCATCGTCGGGAGGCCTTCGACTAGACCTACTCGCATGGTTCGAGCGGTAATATCGG
GTCTGAAATCCCTTGACCCAACGGGAGGCGGTACGTGTGATCGGAATCTGCACCTTGC
GAGAGCTAATCGTAATGCCGATGGCTCTTCCACAAGTGAGTGCTGGGGCGCGAGTGGC
GGACCAGATTGTCTTCCGAATGTTCCCCCCCGTGTCGAAGGCTGAACTCGAGGCGGAG
CCGTGAGCGCGCACCGTTGCCACGCAGGTGCACCGCGAGGCACCTCGAAGGTCCCGTC
ATGAAG

>EHL_0F0090.2

GTCGGGATGCCTTCCCAACTCTGGCACGGCGCGGCGCCTTCGCGCACGGATCGGATAA
GCTTTCCCGTGGACGGCTCGTCCTCGGAAATATCTCGTAGTCTTCTCGTTGGTTGCTCCA
ACGAAGCGACGAATCTACTTGGATACTGAACCGCGAGAGCTATTCAAGTGCTCTACTA
CTCGACCGGGGGCAGCATACAGCCGTGGGATCGGCGCGGCCCACCTCCGGCCAACGAG
AGTTACGAGTACCCGGCCCGTTTTCCCGCGTCGGAGGCTGCCAACACGATAGTAACTG
AACTGGCCCAAACGTATTGATACGCCCCGGGGGCGGGCCTCTGGCGCGCCACTGGATC
AGGCCCGTGGCGTGCCCGCCTCGTCAGCGCCACCCATTGCTAAGCGCTGACAGTAATA
GACCCCTCCATAGTAGTTGCCGATGTTAATTCGGTCACCGGCCAAAACGTATGCACTTA
GCACAGGGCAGGTACTACAAAGCGAGAGGTGGACGATTGGAAAAGGCTGCTGGCGAG
CCTACCAACCTGTTCCTCCGCGCCTGCTGGAGCGACCAACTACGCCCCGCAGCGACCG

GTACTTGAGCTGTCTCTCGACTGGTCCGATTAGATTGTATAAACAGTCGTTAAGACGCG
GACTAAACAACCTCCTCATACCAGTCCGCCCGTCCGGAGCGGAACGACTCGGCGGTAC
CGGCGTCAGGCCCCCTCGCCTAGGCTACACCATTATGTTGGGAGGTGCGTCGACTGGG
GGCCGGCGACTTGAGCTCGGTCGTCCCGTGAAGCCCATCAGTCCCATGGAGACGTTCT
CTCCCATCCATTGGCCTCCGGGGCTCTCCACCAATCGCACCGGAGTCTTGTCTAGTGTT
AAATGTATTCATCGTGGGGTATAAACCGCCCCCGCGTAAGTGCGGCTCGAAGTCTGGC
CTCCCAGGCTGCTAGCAAACACGGAAGTGCGCCTCGTTTCGCTCAGAGGGGACAAGAC
ACTCTCGACGGCTCTCAACAAGCCTAGTCCCCACGATACATACAACCGTGCCCGGTGG
GATTAATACGCCGGGGTCCCGTCGCGGGACATCTGGGATGGGAACACGTTCCGGCCAT
ATGCTCTCTGTCCGCATCTCTTGGGGAAATCCATTACGCTGCCGTTGACGTCGTTAACG
TCACGCCACTAATACCGAACTAGGGGGGCCAAACTATACCATGTTATCCGTGCGCCTA
CCCACTGTTGCGATTCAGGGTACGGGGCTGTGAGTAGTGTACACACTCTTGATGGGAG
CTTATCCTCCGGACTTGAATGGCTACTCGCCGGGCTGGGACTTAGCAAACCATCCCAAC
ATATTCCAGGCCTCTGATCAGATCACAATTTTGCTTTCACCAGTTCCCAATTATGATTCC
GCATCGTCGGGAGGCCTTCGACTAGATCTGCTCGCATGGTTCGAGCGGTAATATCGGG
TCTGAAATCCCCTGACCCAACGGGAGGCGGTATGCGTGATCGGAATCTGCACCTTGCG
AGAGCTAATCGTGATGACAATGGCTCTTCCACAAGTGAGTGCGGGGGCGCGAGCGGCG
AACCAGATGGTCCTCTGAATATTCCCCCCCGTGCCGAAGGCTGAACTCGAGGCGGGGC
CGTGGTCGCGCACCGTTACCACGCAGGTGCGTCGCGAGGCCCCTTGAAGGTCCCGTCA
TGAAG

>EHL_0F0094.1

GTCGGGATGCCCTTTCGACACCAGCACGGCGCGACGCTTTCGCGCACGTATCAGATAA
GTTTTCCCGTGGACGGCTCGTCCTCAGAAATATCTCGTAGTCCTTTCGTTGGTTACTCCA
ATGAAGCGCCTCGCCTGCGGGAGCACTAAACCGCGAGCGCCATTCGAGTGCTCTACAA
CTCGACCGGGGGCAGCACACAACCGTAGGATCGGCGCGGCTCATTTTCGGCTAGTAGG
GGGTACGACGTCCCGGCCCGTTTTCTCGCGTCGGAGGCTGCCAACACGATAGTAACTG
AACTGGCCCAAACGTATTGATACGCCCAGAGGGCGGGCCTCTGGCGCGCCACTGGATC
AGGCCCGTGGCGTGCCCGCCTCGTCAGTGCCACCCATTGCTAAGCGCTGACAGTAATA
GACCCCTCCATAGTAGTTGCCGATGTTGATTCGGTCACCGGCCGAAACGCGCGCGCTC
AGCACAAGGCAGGTACCACGGAGCGAAAGGTGGATGATTGGAAGGGGCTGCTGACGC
GCCTATCAGCCCGTTCCCCCGCGCCTGCTGTGGCGACCAACTACGCCCCGCAGCGTCCA
GTACCCGAGCAGTCTCTCAACTGGTCCGATTAGACTGTATACACCGCCGTTGGGACGC
GGACTAAACAACTCCCTCATACCCATCCGCCCGTCCGGAGCGGAACGACTCGGCGGTA
CCGGCGTCAGGCCCCCTCGCCTAGACTGCACCATTATGTTGGGAGGTGCGTCGACTGG
GGGCCGGCGACTTGATCCTGGTCGGCCCGTGAAGCCCATCAGTCCCATGGAGACGTTC
TCTCCCATCTATTGGCCTCCGGGGCTCTCCACCAATCGCACCGGAGTCTTGTCTAATAT
GCAATTTATTCATCGTGAGGTATAAACCGCCCCCGAGTGGGTGCGGCTTGAAGTCCAG
CCCCCCAGGCCGCCTGCTAACACGGAAGTGTACCCGGTTCCACTCAGAGGGCATGAGG
CAGTACCTACGGATCTCAACAAGCCTAGTCCCCATGATACGTACAGCTGGGCCAGGTG
GGATTAATACGCCGGGGTCCCGTTGCGGGATGTCTAGGGGGGAGATACGTTCCGGTCA
TATACTCTTTGTCCGCATCCCTTGGGGAAATCCATTATGCTCCCGTTGACGTCGTCAAC
GTCACGCCACTAACACCGAACTAGGGGGACCAAACTATACCATGTTATCCGTATACTT
ACCCACTGTTGCAGGTCAAGGTATGGGGCTGCAAGTAATGTACATACTCCTGGTGGGA
GCTTGTCCTCCGGACTCGAATGGCTACTCGCCGGGCTGGGACTTAGCAAACCACTCTGA
CGCATCTACGGCCTCAGATTAGATCACAATTTTGCTTTCTCCATTTCCTAATTACCGTTC
CGCATCGTCGGGAGGCCTTCGACTAGATTTACTCGCATGGTTTAAGCGATAATATCAAG
TCTGGAATCCTCTGACCCAACGTGAAGCGGTATGCGCAATTGGGGTCTGTTCCTTGCAA
GAGCTAATCGTAATGACGATGGACCTTCTACAAGTGAGTGCTGTAGCGCCAGCAGCGG
ACCAGATTGTTTTCCGGATGTTCCCCCTCGTGCCGAAGGCTGAACTCAGGGCGGGGCC

GTGGGCGCGCATCGTTGCCACGCAGGTACACCGTGAGGCACCTTGAAGGTCCCGTCAT
GAAG

>EHL_0F00904.2

GTCGGGATGCCTTCCCAACTCCGGCACGGCGCGGCGCCTTCGCGCACGGATCGGATAA
GCTTACCCGTGGATGGCTCGTTCTCAGAAATATCTCGTAGTCTTCTCGTTGGTTACTCCA
ATGAAGCGCCTCGTCTGCGGGGGCACTGAACCGCGAGAGCTATTCAAGTGCTCTACTA
CTCGACCGGGGGCAGCTTACAACCGCGGGATTGGCACGGTCTACCTCCGTCCAACAGG
GGGTACGACGACCCGGTCCCCCCTCCCGCGTCGAAGGCTGCCAACACGATAGTAAGCG
AACTGGCCCAAACGTATTAATACGTACAGGGGGCGGGCCTCTGGCGCGCCACTGGATC
AGGCCCGTGGCGTGCCCGCCTCGTCAGCGCCACCCATTGCTAAGCGCTGACAGTAATA
GACCCCTCCATAGTAGTTGCCGATGTTGATTTGGTCACCGGCCAAAACGTATGCGCTCA
GCACAGGGCAGGTACTACGGAGCGAAAGGTGGATGATTGGCAGGGGCCGCTGGCGCA
CCTACAAAACTATTCGTCCGCGCCTGCTGGAGCGACCAACTACGCTCTATAGCGTCCAG
TACCCGAGCAGTCCCTCAATTGGTCCGATGAGACTGTATACGCCGCCGTTGGGACGCG
GACTAAACAACCCCCTCATACCCATTCGCCCGTCCAGAGCGGAACGACTCCGCGGTGC
CGGCGTCAGGCCTCCTCGCCTAGGCTGCGCCATTATGTTGGGAGGTGCGTTGACTGGG
GGCTGGCAATTTGATCCCGGTCGGCCCGTGAAGCCCATCAGTTCCATAAAGACATTCGT
TGCCGTCCATTGGCCTTCTGGGCCCTCCGCCAATTACGCCGGAGTATCGCCTAGGGTGA
AATGCATTCAACGTGAGGTATAAACCGCCCCCGCGTGGGTGCGGCTCGATGTCTGGCC
TCCCAGGCTGGCAGCTAACACGGAAGTGTGCCTCGTTCCACTCAGAGGGCATGAGGCA
GTACCTACGGATCTCAACAAGCCTAGTCCCCATGATACGTTCAGCTGGGCCAGGTGGG
ATTAATACGCCGGGGTCCCGTCGCGGGATGTTTGAGGGGGAGATACGTTCCGGCCATA
TACTTTTTGTCCGCATCCCTTGGGGAAATCCATTACGCTGCCGTTGACGTCGGTAACGC
CGCTTCATTAACGTCGAACCAGGGAGACCAAACTACACCATGTTATCCGTATGCCCAG
TTGTTGTTGCGGTTCAGGGTACGGGGCTGTGAGGCACGGGCGCGCTCCTGGTGGGAAC
TTGTCCTCCGGACTTTAATGGCTACTCGCCGGGCTGAGACTTAGCAGACCACCCTGACG
CATTCCAGGCCTCAGATTAGATCACAATTTTGCTTTCATTAGTTCCCAATTACGGTTCCG
CATCGTCAGGAGGCCTTCGACTAGATCTACTCGTATGGTTCGAGCGGTGATATCGGGTC
TAAAATCCCCTGACCCAACGGGGGGCGGTACGTGTGATCGGAATGTGCACCTTGCGAG
ATCTAATCGTAATGACGATGGCTCTTCCACAAGTGAGTGCTGGGGCGCGAGCGGCGGA
CCAGATGGCCTTCCGAATGTTCCCCCCCGTGCCGAAGGCTTAACTCGAGGCGGAGCCG
TGGGCTCGCACCGTTGCCGCGCAGGTACACCGCGAGGTCCCTTGAAGGCCCCATCATG
GAG
